# Network Pharmacology-Based Investigation on the Mechanism of the JinGuanLan Formula in Treating Acne Vulgaris

**DOI:** 10.1155/2022/6944792

**Published:** 2022-07-13

**Authors:** Noha Saleh Gholais, Chunrui Shi, Jing Zhang, Bei Liao, Rowida A. Albarmaqi, Xiaolong Tang, Leyuan Mi

**Affiliations:** ^1^Department of Dermatology, The First Hospital of Lanzhou University, Lanzhou City, Gansu Province, China; ^2^The First Clinical Medical College, Lanzhou University, Lanzhou City, Gansu Province, China; ^3^Kunming Medical University First Affiliated Hospital, Kunming, China

## Abstract

**Background:**

JinGuanLan (JGL) formula is a traditional Chinese medicine (TCM) developed by the Department of Pharmacology at the First Hospital of Lanzhou University. The network pharmacology approach was applied to determine the potential active compounds, therapeutic targets, and main pathways of the JGL formula to evaluate its application value in acne vulgaris.

**Methods:**

Data on the active compounds and their related targets were obtained from the Traditional Chinese Medicine Systems Pharmacology Database and Analysis Platform (TCMSP). Acne vulgaris-related targets were searched from the Online Mendelian Inheritance in Man (OMIM) database, GeneCards Database, Comparative Toxicogenomics Database (CTD), Therapeutic Target Database (TTD), and DisGeNET Database. Targets intersecting between JGL- and acne vulgaris-related targets were chosen as potential therapeutic targets. The protein-protein interaction (PPI) network of potential therapeutic targets was visualized using Cytoscape software based on the PPI data collected from the STRING database. Three topological features, namely, “Degree,” “MCC,” and “EPC” of each node in the PPI network were calculated using the cytoHubba plugin of Cytoscape to excavate the core targets. R program was used for the Gene Ontology (GO) and Kyoto Encyclopedia of Genes and Genomes (KEGG) enrichment analysis of the potential therapeutic targets. Finally, the compound–target–pathway network was constructed.

**Result:**

Among the 148 active compounds that were identified, quercetin and kaempferol showed the highest degree of target interaction and thus may play essential roles in the pharmacological effect of the JGL formula for acne treatment. Among the 97 potential therapeutic targets that were screened out, the 6 core targets were TNF, JUN, IL6, STAT3, MAPK1, and MAPK3. A total of 2260 terms of GO enrichment analysis were obtained, including 2090 for biological processes (BP), 37 for cellular components (CC), and 133 for molecular function (MF). A total of 156 enriched KEGG pathways were identified, including TNF, IL-17, Th17 cell differentiation, MAPK, PI3K-Akt, T cell receptor, and Toll-like receptor signalling pathways.

**Conclusion:**

This work showed that the JGL formula might reverse the pathological changes associated with acne vulgaris through its antiinflammatory effect and regulate the excessive lipogenesis in sebaceous glands via different signalling pathways. This new drug has application value and is worthy of further research and development.

## 1. Introduction

Acne vulgaris is a chronic inflammatory cutaneous disorder affecting the pilosebaceous unit. Acne ranks eighth in the top ten most frequent diseases worldwide, and acne vulgaris has a prevalence rate of 94% according to the Global Burden of Disease Project [1, 2]. Although no mortality is associated with acne, physical comorbidities, such as permanent scarring and hyperpigmentation, and psychiatric comorbidities, such as poor self-image, depression, and anxiety, are commonly correlated with this disease [3]. Acne causes the greatest burden between the first and third decades of life [4], and its pathogenesis includes four key elements, namely, follicle colonization with *Propionibacterium acnes* (*P*. *acnes*), infundibular hyperkeratinisation, sebum production alteration, and inflammation [5]. This complex pathogenesis poses a massive challenge to the antiacne medication's effect [6]. The inflammatory reaction plays a significant role in the acne lesions' progression [7]. The major cause of the inflammatory response observed in acne vulgaris is *P*. *acnes* [8]. Most Chinese herbal medicines have antiinflammatory effects. In general, the therapeutic strategy of acne in Chinese herbal medicine is comparable with that in Western medicine therapy and focuses on the antiinflammatory and antibacterial mechanisms as well as a reduction in sebum production and hyperkeratinisation [5]. With the significant increase in antibiotic resistance, Traditional Chinese medicine (TCM) may provide a new way to solve this problem [9].

TCM is a comprehensive medical system that has been used in China for thousands of years and is also becoming popular in Western countries due to its therapeutic efficiency and few side effects [10, 11]. One of the main features of Chinese medicine is the synergistic effect of TCM by working at different levels on multiple targets, compounds, and pathways [12].

JinGuanLan (JGL) is a new herbal formula developed by the Pharmacology Department at the First Hospital of Lanzhou University. This formula comprises five medicinal herbs, namely, *Lonicerae Japonicae Flos* (Jinyinhua, JYH), *Licorice* (Gancao, GC), *Isatidis Radix* (Banlangen, BLG), *Fortunes Bossfern Rhizome* (Guanzhong, GZ) and *Hedysarum Multijugum Maxim or Astragali Radix* (Huangqi, HQ). Most existing Chinese medicines for acne treatment contain *Lonicerae Japonicae Flos*, *Licorice, or Astragali Radix* as monarch drugs [13]. GC is a famous Chinese herb with antiinflammatory, antioxidant, and antibacterial activities. The antiinflammatory and antiacne effects of GC are mainly attributed to its flavonoid compounds such as kaempferol, quercetin, naringin, formononetin, and luteolin [6]. JYH can exert a significant antiinflammatory activity and is the most favourable herb in Chinese medicine for acne treatment [13, 14]. HQ possesses antioxidant, antiinflammatory, and immune regulatory properties [15]. BLG has potent antiviral properties and antiinflammatory, antibacterial, and immunomodulatory effects and is considered a heat-clearing and detoxifying herb [16]. Reports on GZ are limited. At present, experimental studies on the JGL formula are in progress.

**Figure 1:**
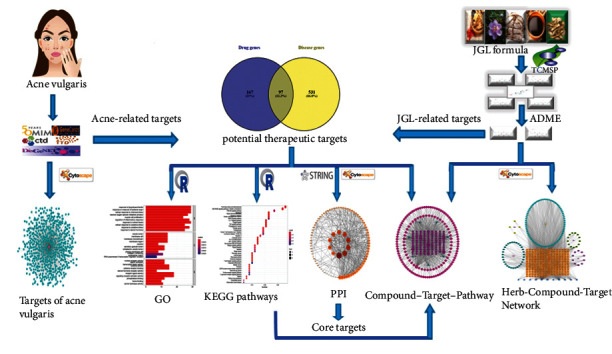
Graphical abstract of the network pharmacology approach.

Network pharmacology is an emerging approach that integrates network biology with poly-pharmacology [17]. This concept was presented for the first time in 2007 by pharmacologist Hopkins and was regarded as the next paradigm in drug discovery [18]. Network pharmacology efficiently overpasses the gap between TCM and Western medicine and facilitates the mechanistic studies of the synergistic effects of TCMs [19]. In this study, a comprehensive analysis was conducted on the JGL formula to determine its application for treating acne vulgaris. The graphical abstract of this network pharmacology approach is shown in (Figure 1).

## 2. Methods

### 2.1. Screening of Active Compounds and Targets

The Latin names of each herb were entered into the Traditional Chinese Medicine Systems Pharmacology Database and Analysis Platform version 2.3 TCMSP (https://old.tcmsp-e.com/tcmsp.php) to explore all the candidate compounds of the five herbal medicines in the JGL formula. Active compounds were screened out according to the pharmacokinetic activity parameters (ADME) and must meet the following standard screening criteria: oral bioavailability (OB) ≥ 30% and drug-likeness (DL) ≥ 0.18 [20–22].

### 2.2. Screening the Target Genes of the Selected Active Compounds

The corresponding targets of each active compound (JGL-related targets) were acquired from TCMSP. The UniProt database (https://www.uniprot.org/) was used to standardize the target gene names and remove invalid targets; only ‘Reviewed (Swiss-Prot)' and ‘Homo sapiens' target genes in UniProt were selected to ensure prediction accuracy [23].

### 2.3. Search Targets of Acne Vulgaris

‘Acne vulgaris' was used as a keyword to search for acne vulgaris-related targets in the following five databases: Online Mendelian Inheritance in Man database (OMIM) (https://www.omim.org/), GeneCards Human Gene database (https://www.genecards.org/), Comparative Toxicogenomics Database (CTD) (http://ctdbase.org/), Therapeutic Target Database (TTD) (http://bidd.nus.edu.sg/BIDD-Databases/TTD/TTD.asp), and DisGeNET Database (https://www.disgenet.org). All the search results were then merged, and the duplicate targets were removed to select all the acne vulgaris-related targets.

Targets intersecting between the JGL- and acne vulgaris-related targets were selected as the potential therapeutic targets of JGL in treating acne vulgaris using the Venny 2.1.0 online tool (http://bioinfogp.cnb.csic.es/tools/venny/index.html).

### 2.4. Protein-Protein Interaction (PPI) Network Construction and Topological Analysis

The potential therapeutic targets were uploaded into the Search Tool for the Retrieval of Interacting Genes database (STRING) version 11.0 b (https://string-db.org/) to obtain the data of the PPI of JGL formula and acne vulgaris targets. In STRING, the organism was set as ‘*Homo sapiens*', and the ‘highest confidence score of 0.9' was defined as a significant interaction score with hidden disconnected nodes in the network [24, 25].

Subsequently, the PPI data collected from the STRING database was used to visualize the PPI network via Cytoscape software version 3.8.1. Three topological parameters, namely, degree, edge percolated component (EPC), and maximal clique centrality (MCC), were analysed using the Cytoscape plugin CytoHubba to assess the Hub nodes and subnetworks within the PPI network. Finally, the top 10 nodes for each parameter were chosen as core targets that play an essential part in the PPI network [26].

### 2.5. GO and KEGG Enrichment Analysis

The names of the potential therapeutic targets were inputted into the R program (version 3.6.3) to perform Gene Ontology (GO) and Kyoto Encyclopedia of Genes and Genomes (KEGG) enrichment analysis using the *P* value cut-off < 0.05 and q-value cut-off < 0.05 as screening criteria. GO enrichment analysis included the following terms: biological processes (BP), cellular components (CC), and molecular function (MF). The results were presented in visual bubbles, bar charts, and data tables.

## 3. Results

### 3.1. Active Compounds and Potential Therapeutic Targets of the JGL Formula

A total of 802 compounds in the JGL formula were obtained from the TCMSP database. Among them, 181 were identified as potential active compounds using the screening criteria: OB ≥ 30% and DL ≥ 0.18. Out of the 181 potentially active compounds, 19 without corresponding targets were removed because they were not expected to interact with human targets. Finally, 162 active compounds were retrieved. The number of active compounds and associated targets in each herb of the JGL formula are listed in Table 1. Among these 162 compounds, eleven existed in more than one herb, namely, kaempferol, quercetin, mairin, jaranol, isorhamnetin, formononetin, calycosin, beta-sitosterol, stigmasterol, sitosterol, and DFV. Finally, 148 active compounds were retrieved after the duplicates were removed.

A total of 3123 potential targets were discovered for all compounds using TCMSP databases: 337, 1769, 106, 462, and 449 targets for BLG, GC, GZ, HQ, and JYH, respectively. UniProt was used to standardize the target gene names and yielded 3048 targets. Finally, 264 JGL-related targets were obtained after the redundant terms were eliminated. Basic information for all active compounds and related targets is shown in Supplementary file 1, Tables 1 and S2.

**Figure 2:**
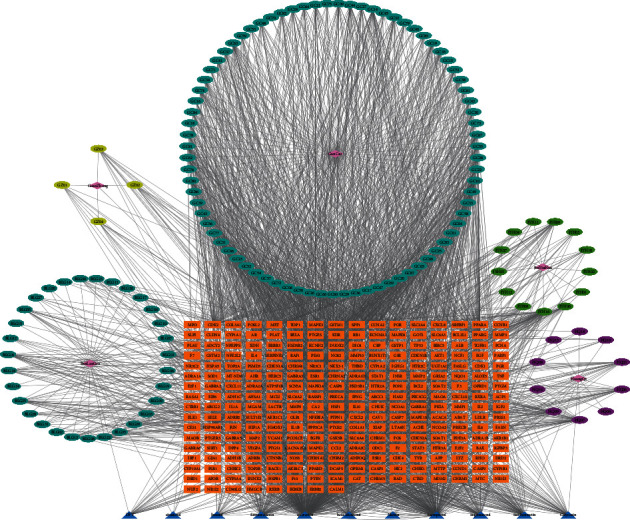
“Herb-compound-target” network of the JGL formula. The nodes that represent active compounds are polychrome circles. The orange squares represent the targets, and the blue triangles represent the compounds in the five herbs.

A total of 148 active compounds were screened, and 264 JGL-related targets were obtained. A visual ‘herb-compound-target' network was then constructed using Cytoscape 3.8.1 (Figure 2). The network contained 417 nodes (5 herbs, 148 compounds, and 264 targets) and 3210 edges. The ‘analyze network' tool in Cytoscape was applied to obtain the degree parameter of the network. Among the top 10 compounds of high degree nodes, quercetin displayed the most target interactions, followed by kaempferol (Table 2). Therefore, these compounds may play essential roles in the pharmacological effect of JGL.

### 3.2. Target Genes of Acne Vulgaris

A total of 732 targets related to acne vulgaris were identified using the MultiSource Database Integration Method. Among them, 524, 7, 95, 19, and 87 targets were from GeneCards, OMIM, CTD, TTD, and DisGeNET databases, respectively. After the redundant terms were eliminated, 628 potential targets related to acne vulgaris (acne vulgaris-related targets) were obtained. Detailed information for the potential target genes of acne vulgaris is listed in Supplementary file 2, Table S3, and Supplementary file 3, Figure S1.

**Figure 3:**
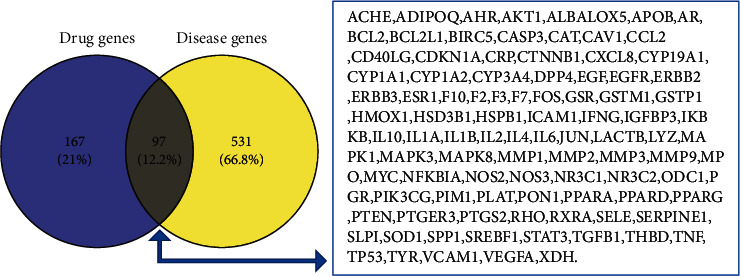
Venn diagram of the JGL- and acne vulgaris-related targets.

Afterward, 97 intersecting targets between 264 JGL-related targets and 628 acne vulgaris-related targets were identified as potential therapeutic targets, and a Venn diagram was drawn using the Venny 2.1.0 online tool (Figure 3).

### 3.3. PPI Network Construction and Core Target Identification

**Figure 4:**
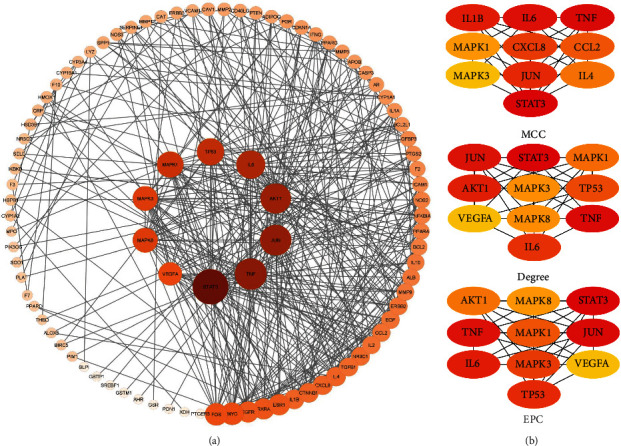
Protein-protein interaction (ppi) network. (a) Ppi network of 97 potential therapeutic targets. The change of node size or colour from red to yellow indicates the change in degree value from high to low. (b) Top 10 targets of three topological features, degree, mcc, and epc.

The 97 potential therapeutic targets were imported into the STRING database for PPI data analysis. The PPI network of potential therapeutic targets was visualized via the Cytoscape software using the PPI data collected from the STRING database (Figure 4(a)). The network contains 91 nodes and 400 edges. Three topological features, namely, “degree,” “MCC,” and “EPC,” of each node in the PPI network were calculated using the CytoHubba plugin of Cytoscape. Finally, the overlapped targets among the top 10 targets of three topological features were chosen as the core targets, and the subnetworks were constructed as shown in (Figure 4(b)). The core targets included TNF, JUN, IL6, STAT3, MAPK1, and MAPK3 as listed in (Table 3).

### 3.4. GO Functional Enrichment Analysis

**Figure 5:**
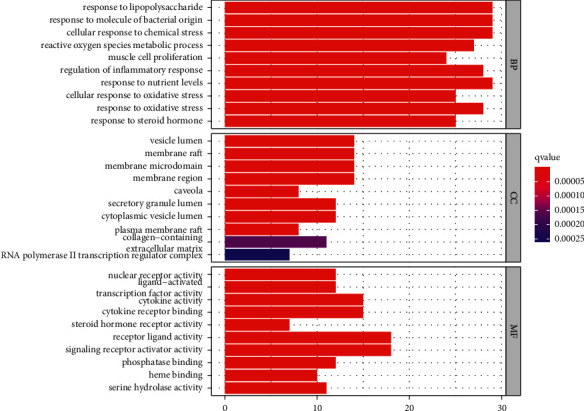
GO enrichment analysis of the potential therapeutic targets of the JGL formula against acne vulgaris.

The R program was used for the GO enrichment analysis of the 97 potential therapeutic targets to further understand the mechanism of the JGL formula in treating acne vulgaris. The results revealed 2090 terms for BP, 37 for CC, and 133 for MF. According to the *P* value cut-off < 0.05, the top 10 GO terms of each term were selected as significantly enriched terms (Table 4). The bar chart of the top 10 of each GO term is displayed in Figure 5. On the basis of the GO enrichment analysis, the antiacne activity of the JGL formula may result from the synergetic effects of the complex multibiological processes, cellular components, and molecular function. However, the effects of the JGL formula on BP, CC, and MF in acne vulgaris need further experimental study. Detailed information for the GO enrichment analysis of BP, CC, and MF can be found in Supplementary file 4, Tables 4, S5, and S6.

### 3.5. KEGG Pathway Enrichment Analysis

**Figure 6:**
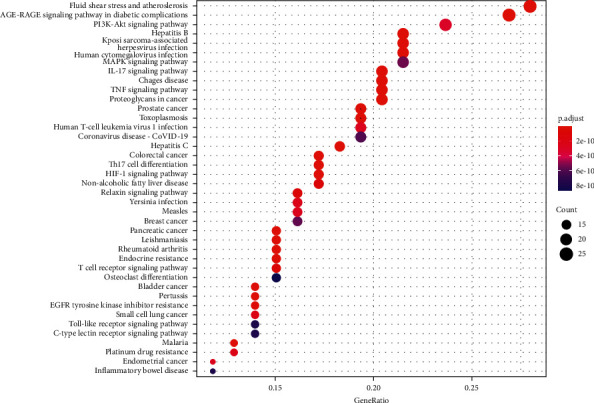
KEGG pathway enrichment analysis. The bubble size represents the number of target genes in the pathway, and the colour represents the *P* value.

KEGG pathway enrichment analysis for the 97 potential therapeutic targets was conducted using the *R* program. A total of 156 enriched pathways were collected, but only those with a *P* value <0.05 were considered significant. The top 40 significantly enriched pathways were selected according to their *P* values from small to large, and a bubble diagram was drawn as shown in (Figure 6). The significantly enriched pathways correlated with acne vulgaris are listed in (Table 5). Detailed information for screened KEGG pathways is provided in Supplementary file 5, Table S7.

### 3.6. Compound-Target-Pathway Network Construction and Analysis

**Figure 7:**
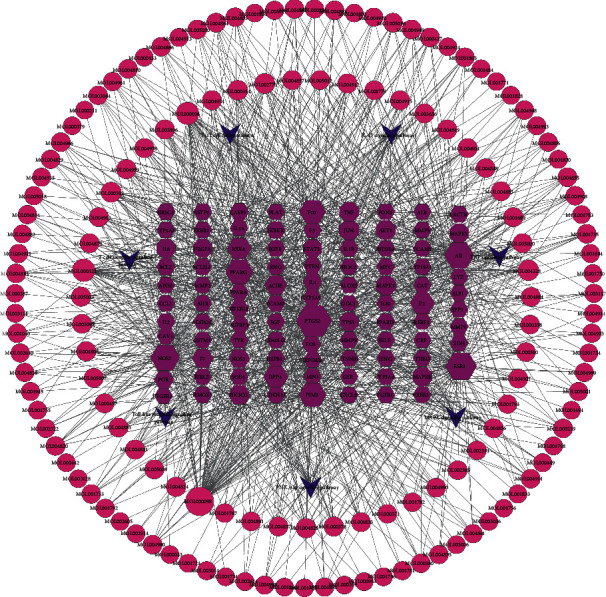
“Compound-Target-Pathway” network. The pink circles represent the active compounds, the purple hexagonal nodes represent the potential therapeutic targets, and the blue V shaped nodes represent the pathways.

The compound-target-pathway network constructed with the Cytoscape 3.8.1 software (Figure 7) had 246 nodes (142 compounds, 97 genes, and 7 pathways) and 1116 edges. Network analysis showed that out of the 148 active compounds in the JGL formula, 6 compounds, namely, ZINC03860434, icos-5-enoic acid, ethyl linolenate, gadelaidic acid, linarin, and isomucronulatol-7,2′-di-O-glucosiole had no connection with these 97 targets. Meanwhile, 3 compounds, namely, quercetin, kaempferol, and luteolin, had a high number of interactions and were linked to more than 20 targets. In addition, a single target might be targeted by many compounds. For example, PTGS2 was targeted simultaneously by 123 active compounds, ESR1 was targeted by 92 active compounds, and AR was targeted by 81 active compounds. The core targets, MAPK1 and MAPK3, were involved in seven selected pathways; TNF, JUN, and IL6 were present in five pathways; and STAT3 was involved in one pathway.

## 4. Discussion

The network pharmacology approach was applied to explore the active compounds, potential therapeutic targets, and significant pathways of the novel JGL formula and investigate its potential mechanism against acne vulgaris.

### 4.1. Therapeutic Effect of the Main Active Compounds of the JGL Formula on Acne Vulgaris

A total of 148 active compounds of the JGL formula were acquired from the TCMSP database using ADME criteria. According to the contractednetworks, the active compounds of the JGL such as quercetin, kaempferol, luteolin, naringenin, beta-carotene, and formononetin may form the primary material basis of its potential antiacne effects. Compared with the other compounds, quercetin had the highest degree value in the network. This compound also exhibits an inhibitory effect on proinflammatory cytokines and chemokines such as IL1*β*, IL8, IL6, and TNF-*α* from *P*. *acnes*-stimulated human keratinocytes and monocytes [27]. Kaempferol has a moderate antibacterial activity and an inhibitory effect on *P*. *acnes* growth [28, 29]. Luteolin exhibits antiinflammatory, antioxidant, and antiandrogenic effects [30]. Formononetin has significant antioxidant, antiadipogenicity, and estrogenic activities [31, 32]. In conclusion, the active compounds of the JGL formula showed multidrug compatibility and synergistic effects in the treatment of acne vulgaris.

Enrichment analysis and compound-target-pathway network analysis further indicated that the JGL might modify the pathological changes associated with acne vulgaris via essential pathways.

### 4.2. Antiinflammatory Effect of the JGL Formula through Toll-Like Receptor, TNF, Th17 Cell Differentiation, IL17, and MAPK Signalling Pathway

Inflammation is one of the key pathogenetic mechanisms of acne and manifests through all the phases of this illness [33, 34]. This condition mainly results from the immune response against *P*. *acnes* [35]. Specific representative pathways, which were widely reported as antiinflammatory, were chosen to clarify the antiinflammatory effect of the JGL formula on acne vulgaris.

*P*. *acnes* plays a direct role in the inflammatory response in acne by activating the innate immune system via the Toll-like receptor pathway (TLR2) on keratinocytes and the sebocytes of the pilosebaceous unit, thus activating signalling cascades and inducing the release of inflammatory mediators such as IL1*ß*, TNF-*α*, IL8 (CXCL8), and IL6 [35, 36]. The involvement of the Th17 pathway in acne pathogenesis can explain the histological findings and inflammation in acne [37]. Th17 cell differentiation pathway inhibition exhibits an antiacne effect by downregulating retinoic acid receptor alpha, the same mechanism exerted by some antiacne medicines that contain vitamins *D* and A [35]. TNF- *α* is one of the most critical proinflammatory cytokines and plays an essential role in the immune response during inflammation. TNF stimulates intracellular signalling cascades to activate mitogen-activated protein kinases (MAPKs), which have great activity and participation in the production of proinflammatory mediators. TNF could also activate matrix metalloprotease (MMPs) for tissue remodelling [38, 39]. MAPKs are grouped into three main families (ERK, JNK, and p38) and play an essential role in complex biological processes and cellular responses to external stimulants. Several external stimuli may activate MAPKs, including ROS and microbial infection (e.g., *P*. *acnes*). Activated MAPK pathways are involved in signalling cascades that activate several transcription factors, such as nuclear factor-kappa B. These three MAPK pathways are the potential targets of many antiinflammatory drugs because they regulate inflammatory mediators at the transcriptional and translational levels [40].

Network and enrichment analyses revealed that many compounds and targets have a role in the inflammatory and immune responses of acne vulgaris. Kaempferol, quercetin, formononetin, and luteolin could be the main compounds responsible for the antiinflammatory effect of the JGL formula. Quercetin significantly suppresses the secretion of proinflammatory cytokines IL8, IL1ß, IL6, and TNF-*α* in *P*. *acnes*-stimulated cells and the phosphorylation of JNK, ERK, and p38 MAPK signalling pathways and the production of TLR-2 in *P*.*acnes*-stimulated human keratinocytes and THP-1 cells. In vitro experiments proved that quercetin suppressed MMP-9 in two cell lines exposed to *P*. *acnes* [27]. Kaempferol exerts an inhibitory effect on ERK1/2 phosphorylation [41] and prostaglandin synthesis by suppressing COX-1 and COX-2 enzyme activity [28]. In vitro experiments showed that the main antiinflammatory effect of luteolin is suppressing NF-*κ*B and MAPK pathways [42]. The core targets and other potential therapeutic targets were involved in most of the inflammatory and immune response-related pathways. Thus, targeting these pathway targets and regulating the production of proinflammatory cytokines and chemokines might be the therapeutic strategies for treating acne using the JGL formula.

### 4.3. Inhibition of Lipogenesis through PI3K-AKT Pathway Regulation

In sebocytes, FoxO1 regulates SREBP-1, a major transcription factor of androgen receptor (AR) regulation. In acne pathogenesis, the activation of the PI3K-Akt signalling pathway inhibits FoxOs and therefore increases lipogenesis [43, 44]. Likewise, the PI3K-Akt/mTORC1 pathway activates SREBP-1, thus, increasing lipogenesis in the pilosebaceous unit [45, 46]. Furthermore, the TNF-*α* significantly induces lipogenesis in the sebaceous gland via JNK and PI3K/Akt pathways as reported in a previous in vitro study on inflammatory acne that TNF-*α* increases lipid droplet accumulation in the sebaceous gland cells [47].

The anti-acne effect of licorice flavonoids, such as kaempferol, quercetin, naringin, formononetin, and luteolin, might be mediated by the inhibition of the PI3K-Akt pathway, leading to an increase in FoxO1 expression level in the skin to suppress the mTORC1 biological activity, which in turn finally restrains SREBP-1 expression and lipid synthesis [6]. Luteolin may inhibit sebocyte growth by suppressing AKT1 and PI3K phosphorylation [48]. Luteolin and formononetin mediate anti-androgenic effects by downregulating androgen receptor AR, thereby reducing sebaceous gland activity [26, 30]. Our study also has some limitations due to the limitations of various databases and the popularity of some topics, and it lacked in vitro validation. Still, it provides a scientific basis for to further studies for in-depth investigation of the effect of the JGL on the treatment of acne vulgaris. In vitro and in vivo studies are needed to verify these results.

## 5. Conclusion

In the JGL formula, 148 active compounds, 97 potential therapeutic targets, and 6 core targets were identified by network pharmacology. Among the active compounds, quercetin and kaempferol showed the highest degree of target interaction and might play a critical role in the pharmacological effect of the JGL on acne vulgaris. The therapeutic effect of the JGL formula was closely related to inhibiting inflammation and regulating excessive lipogenesis in sebaceous glands through specific pathways, namely, Toll-like receptor, TNF, Th17 cell differentiation, IL17, MAPK, and PI3K-AKT signalling pathways. These pathways are among the most important pathogenetic mechanisms of acne vulgaris.

## Figures and Tables

**Table 1:** Number of active compounds and associated targets in each herb of the JGL formula.

Herbal Name	Compounds Number	Target genes number
Jinyinhua (JYH)	17	437
Gancao (GC)	88	1738
Banlangen (BLG)	35	322
Guanzhong (GZ)	5	101
Huangqi (HQ)	17	450

**Table 2:** Basic information of the top 10 active compounds in JGL according to their degree values.

MO ID	Compound Name	Degree	OB (%)	DL	Medicine	2D structure∗
MOL000098	Quercetin	456	46.43	0.28	JYH, GC, and HQ	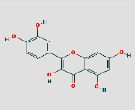
MOL000422	Kaempferol	248	41.88	0.24	JYH, GC, HQ, and GZ	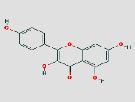
MOL000392	Formononetin	78	69.67	0.21	GC and HQ	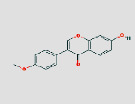
MOL000358	Beta-sitosterol	74	36.91	0.75	JYH and BLG	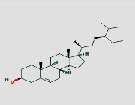
MOL000354	Isorhamnetin	72	49.6	0.31	GC and HQ	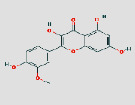
MOL000449	Stigmasterol	62	43.83	0.76	JYH and BLG	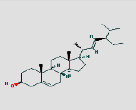
MOL000006	Luteolin	57	36.16	0.25	JYH	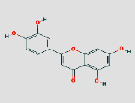
MOL000378	7-O-methylisomucronulatol	45	74.69	0.3	HQ	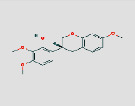
MOL000417	Calycosin	44	47.75	0.24	GC and HQ	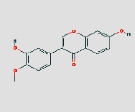
MOL003896	7-Methoxy-2-methyl isoflavone	43	42.56	0.2	GC	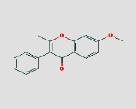

^*∗*^Cited from the DrugBank database.

**Table 3:** Core targets with related compounds.

Core targets	Related compounds	Degree	MCC	EPC
STAT3	Licochalcone a	35	25062	39.842
TNF	Kaempferol, quercetin, luteolin, isovitexin,	30	21311	38.838
JUN	Kaempferol, quercetin,beta-sitosterol, luteolin, formononetin, beta-carotene	29	18028	39.104
IL6	Quercetin, luteolin	26	21300	37.957
MAPK1	Quercetin, luteolin, naringenin, licochalcone a	23	13040	37.997
MAPK3	Naringenin	21	13038	37.577

**Table 4:** Top 10 terms of BP, CC, and MF GO enrichment analysis.

Category	Term	*P* value	*Q* value	Count
GO-BP	Response to lipopolysaccharide	6.85*E* − 28	1.09*E − *24	29
GO-BP	Response to molecule of bacterial origin	4.36*E − *27	3.01*E − *24	29
GO-BP	Cellular response to chemical stress	6.02*E − *27	3.01*E − *24	29
GO-BP	Reactive oxygen species metabolic process	7.59*E − *27	3.01*E − *24	27
GO-BP	Muscle cell proliferation	2.30*E − *24	7.30*E − *22	24
GO-BP	Regulation of inflammatory response	1.38*E − *23	3.32*E − *21	28
GO-BP	Response to nutrient levels	1.47*E − *23	3.32*E − *21	29
GO-BP	Cellular response to oxidative stress	3.27*E − *23	6.48*E − *21	25
GO-BP	Response to oxidative stress	1.06*E − *22	1.87*E − *20	28
GO-BP	Response to steroid hormone	4.95*E − *22	7.86*E − *20	25
GO-CC	Vesicle lumen	8.43*E − *10	5.09*E − *08	14
GO-CC	Membrane raft	8.77*E − *10	5.09*E − *08	14
GO-CC	Membrane microdomain	9.12*E − *10	5.09*E − *08	14
GO-CC	Membrane region	1.50*E − *09	6.29*E − *08	14
GO-CC	Caveola	7.19*E − *09	2.41*E − *07	8
GO-CC	Secretory granule lumen	6.59*E − *08	1.80*E − *06	12
GO-CC	Cytoplasmic vesicle lumen	7.54*E − *08	1.80*E − *06	12
GO-CC	Plasma membrane raft	9.11*E − *08	1.91*E − *06	8
GO-CC	Collagen-containing extracellular matrix	8.71*E − *06	0.000161924	11
GO-CC	RNA polymerase II transcription regulator complex	1.55*E − *05	0.00025964	7
GO-MF	Nuclear receptor activity	4.08*E − *17	4.34*E − *15	12
GO-MF	Ligand-activated transcription factor activity	4.08*E − *17	4.34*E − *15	12
GO-MF	Cytokine activity	1.59*E − *12	1.13*E − *10	15
GO-MF	Cytokine receptor binding	1.23*E − *11	6.55*E − *10	15
GO-MF	Steroid hormone receptor activity	5.60*E − *11	2.38*E − *09	7
GO-MF	Receptor ligand activity	7.57*E − *11	2.68*E − *09	18
GO-MF	Signalling receptor activator activity	8.94*E − *11	2.71*E − *09	18
GO-MF	Phosphatase binding	4.50*E − *10	1.20*E − *08	12
GO-MF	Heme binding	3.00*E − *09	7.09*E − *08	10
GO-MF	Serine hydrolase activity	5.31*E − *09	1.13*E − *07	11

**Table 5:** Potential targets of the JGL formula based on KEGG enrichment analysis.

Pathway	Number of pathway targets	*P* value
IL-17 signalling pathway	CASP3/CCL2/CXCL8/FOS/IFNG/IKBKB/IL1B/IL4/IL6/JUN/MAPK1/MAPK3/MAPK8/MMP1/MMP3/MMP9/NFKBIA/PTGS2/TNF	3.81*E − *19
TNF signalling pathway	AKT1/CASP3/CCL2/FOS/ICAM1/IKBKB/IL1B/IL6/JUN/MAPK1/MAPK3/MAPK8/MMP3/MMP9/NFKBIA/PTGS2/SELE/TNF/VCAM1	1.27*E − *17
Th17 cell differentiation	AHR/FOS/IFNG/IKBKB/IL1B/IL2/IL4/IL6/JUN/MAPK1/MAPK3/MAPK8/NFKBIA/RXRA/STAT3/TGFB1	4.65*E − *14
T cell receptor signalling pathway	AKT1/CD40LG/FOS/IFNG/IKBKB/IL10/IL2/IL4/JUN/MAPK1/MAPK3/MAPK8/NFKBIA/TNF	8.96*E − *12
PI3K-Akt signalling pathway	AKT1/BCL2/BCL2L1/CDKN1A/EGF/EGFR/ERBB2/ERBB3/IKBKB/IL2/IL4/IL6/MAPK1/MAPK3/MYC/NOS3/PIK3CG/PTEN/RXRA/SPP1/TP53/VEGFA	4.77*E − *11
MAPK signalling pathway	AKT1/CASP3/EGF/EGFR/ERBB2/ERBB3/FOS/HSPB1/IKBKB/IL1A/IL1B/JUN/MAPK1/MAPK3/MAPK8/MYC/TGFB1/TNF/TP53/VEGFA	8.37*E − *11
Toll-like receptor signalling pathway	AKT1/CXCL8/FOS/IKBKB/IL1B/IL6/JUN/MAPK1/MAPK3/MAPK8/NFKBIA/SPP1/TNF	1.37*E − *10

## Data Availability

The underlying data supporting the results of this study are included within the article and in the supplementary files. The authors obtained the active compounds and their related targets from the TCMSP Database version 2.3 (https://old.tcmsp-e.com/tcmsp.php). Acne vulgaris-related targets were obtained from OMIM (https://www.omim.org/), GeneCards Database (https://www.genecards.org/), CTD (http://ctdbase.org/), TTD Database (http://bidd.nus.edu.sg/BIDD-Databases/TTD/TTD.asp), and DisGeNET Database (https://www.disgenet.org). Target names were standardized by the UniProt Database (https://www.uniprot.org/). The intersection targets were determined by the Venny 2.1.0 online tool (http://bioinfogp.cnb.csic.es/tools/venny/index.html). The protein-protein interaction (PPI) network was constructed based on the PPI data collected from the STRING Database version 11.0 b (https://string-db.org/).

## Abbreviations

JGL: JinGuanLan formula
TCM: Traditional Chinese medicine
TCMSP: Traditional Chinese Medicine Systems Pharmacology Database and Analysis Platform
OMIM: Online Mendelian Inheritance in Man database
CTD: Comparative Toxicogenomics Database
TTD: Therapeutic Target Database
DisGeNET Database: A database of gene-disease associations
P. acnes: Propionibacterium acnes
PPI: Protein-protein interaction
JYH: Jinyinhua
GC: Gancao
BLG: Banlangen
GZ: Guanzhong
HQ: Huangqi,
ADME: Absorption, distribution, metabolism, and excretion
OB: Oral Bioavailability
DL: Drug-Likeness
EPC: Edge Percolated Component
MCC: Maximal Clique Centrality
GO: Gene Ontology
KEGG: Kyoto Encyclopedia of Genes and Genomes
BP: Biological processes
CC: Cellular components
MF: Molecular function
TNF: Tumor necrosis factor
MAPK3: Mitogen-activated protein kinase 3
JUN: Transcription factor AP-1
IL6: Interleukin-6 IL6
STAT3: Signal transducer and activator of transcription 3
MAPK1: Mitogen-activated protein kinase 1
SREBP-1: Sterol-regulatory element binding proteins 1.

## Supplementary Materials

Supplementary Materials Supplementary file 1, Tables S1 and S2: the basic information of all active compounds and related targets. Supplementary file 2, Table S3; and Supplementary file 3, Figure S1: the detailed information of the potential target genes of acne vulgaris. Supplementary file 4, Tables 4, S5, and S6: the detailed information of GO enrichment analysis for BP, CC, and MF. Supplementary file 5, Table S7: the detailed information of screened KEGG pathways.
