# Supplementary material for: Network Pharmacology-Based Investigation on the Mechanism of the JinGuanLan Formula in Treating Acne Vulgaris
Source: Evid Based Complement Alternat Med. 2022 Jul 13;2022:6944792. doi: 10.1155/2022/6944792 (PMC9300327; doi:10.1155/2022/6944792)

| **Supplementary file1: Table S1: The basic information of all active compounds** | | | | | | | | | |
| --- | --- | --- | --- | --- | --- | --- | --- | --- | --- |
| Latin name | | | Chinese name | Molecular ID | Molecular name | OB (%) | DL | CAS | InChIKey |
| *Fortunes Bossfern Rhizome* | | | Guanzhong | MOL001040 | (2R)-5,7-dihydroxy-2-(4-hydroxyphenyl)chroman-4-one | 42.36 | 0.21 | 480-41-1  14259-46-2  93602-28-9 | FTVWIRXFELQLPI-CYBMUJFWSA-N |
| *Fortunes Bossfern Rhizome* | | | Guanzhong | MOL002605 | 11-Hydroxynumantenine | 50.79 | 0.71 | N/A | VYZFRSLZSBLYIC-FGDSOQBESA-N |
| *Fortunes Bossfern Rhizome* | | | Guanzhong | MOL002610 | ZINC00035529 | 58.39 | 0.22 | 22964-77-8 | NETGEQWGGLFVRL-DOMZBBRYSA-N |
| *Fortunes Bossfern Rhizome* | | | Guanzhong | MOL002614 | Flavidin | 30.1 | 0.26 | 83924-98-5 | QMOLHJKSZMURCV-UHFFFAOYSA-N |
| *Fortunes Bossfern Rhizome* | | | Guanzhong | MOL000422 | kaempferol | 41.88 | 0.24 | 520-18-3 | IYRMWMYZSQPJKC-UHFFFAOYSA-N |
| *Licorice* | | | Gancao | MOL001484 | Inermine | 75.18 | 0.54 | 2035-15-6 | HUKSJTUUSUGIDC-ZBEGNZNMSA-N |
| *Licorice* | | | Gancao | MOL001792 | DFV | 32.76 | 0.18 | 578-86-9 16006-91-0 17002-53-8  2885-30-5 | FURUXTVZLHCCNA-AWEZNQCLSA-N |
| *Licorice* | | | Gancao | MOL000211 | Mairin | 55.38 | 0.78 | 472-15-1 | QGJZLNKBHJESQX-FZFNOLFKSA-N |
| *Licorice* | | | Gancao | MOL002311 | Glycyrol | 90.78 | 0.67 | 23013-84-5 | LWESBHWAOZORCQ-UHFFFAOYSA-N |
| *Licorice* | | | Gancao | MOL000239 | Jaranol | 50.83 | 0.29 | 3301-49-3 | BJBUTJQYZDYRMJ-UHFFFAOYSA-N |
| *Licorice* | | | Gancao | MOL002565 | Medicarpin | 49.22 | 0.34 | 32383-76-9 | NSRJSISNDPOJOP-BBRMVZONSA-N |
| *Licorice* | | | Gancao | MOL000354 | isorhamnetin | 49.6 | 0.31 | 480-19-3 | IZQSVPBOUDKVDZ-UHFFFAOYSA-N |
| *Licorice* | | | Gancao | MOL000359 | sitosterol | 36.91 | 0.75 | 83-46-5  149-91-7  474-58-8  915-05-9  5779-62-4  64997-52-0 | KZJWDPNRJALLNS-ZFVHJZABSA-N |
| *Licorice* | | | Gancao | MOL003656 | Lupiwighteone | 51.64 | 0.37 | 104691-86-3 | YGCCASGFIOIXIN-UHFFFAOYSA-N |
| *Licorice* | | | Gancao | MOL003896 | 7-Methoxy-2-methyl isoflavone | 42.56 | 0.2 | 19725-44-1  82517-12-2 | XRGWZIGGCNSFRY-UHFFFAOYSA-N |
| *Licorice* | | | Gancao | MOL000392 | formononetin | 69.67 | 0.21 | 485-72-3 | HKQYGTCOTHHOMP-UHFFFAOYSA-N |
| *Licorice* | | | Gancao | MOL000417 | Calycosin | 47.75 | 0.24 | 20575-57-9 | ZZAJQOPSWWVMBI-UHFFFAOYSA-N |
| *Licorice* | | | Gancao | MOL000422 | kaempferol | 41.88 | 0.24 | 520-18-3 | IYRMWMYZSQPJKC-UHFFFAOYSA-N |
| *Licorice* | | | Gancao | MOL004328 | naringenin | 59.29 | 0.21 | 153-18-4  478-01-3  480-41-1  529-55-5  10236-47-2  14259-46-2  81202-36-0  93602-28-9 | FTVWIRXFELQLPI-ZDUSSCGKSA-N |
| *Licorice* | | | Gancao | MOL004805 | (2S)-2-[4-hydroxy-3-(3-methylbut-2-enyl)phenyl]-8,8-dimethyl-2,3-dihydropyrano[2,3-f]chromen-4-one | 31.79 | 0.72 | 157414-03-4 | NEIURIYDQMKXIG-QHCPKHFHSA-N |
| *Licorice* | | | Gancao | MOL004806 | euchrenone | 30.29 | 0.57 | N/A | LOMFVXOLTBGKBK-CVUHLLMESA-N |
| *Licorice* | | | Gancao | MOL004808 | glyasperin B | 65.22 | 0.44 | 142488-54-8 | PDKHNRSRVURSHL-HNNXBMFYSA-N |
| *Licorice* | | | Gancao | MOL004810 | glyasperin F | 75.84 | 0.54 | N/A | CFCUNFSHJIQKLS-ZDUSSCGKSA-N |
| *Licorice* | | | Gancao | MOL004811 | Glyasperin C | 45.56 | 0.4 | 142474-53-1 | RCZMWVKBVFOCEE-ZDUSSCGKSA-N |
| *Licorice* | | | Gancao | MOL004814 | Isotrifoliol | 31.94 | 0.42 | N/A | OVLUQDOJWGTPHA-UHFFFAOYSA-N |
| *Licorice* | | | Gancao | MOL004815 | (E)-1-(2,4-dihydroxyphenyl)-3-(2,2-dimethylchromen-6-yl)prop-2-en-1-one | 39.62 | 0.35 | N/A | TUHJQMZJOMZXJO-XVNBXDOJSA-N |
| *Licorice* | | | Gancao | MOL004820 | kanzonols W | 50.48 | 0.52 | N/A | VFIXONREXXFDQV-UHFFFAOYSA-N |
| *Licorice* | | | Gancao | MOL004824 | (2S)-6-(2,4-dihydroxyphenyl)-2-(2-hydroxypropan-2-yl)-4-methoxy-2,3-dihydrofuro[3,2-g]chromen-7-one | 60.25 | 0.63 | N/A | GAUFLNQQCSXBPK-SFHVURJKSA-N |
| *Licorice* | | | Gancao | MOL004827 | Semilicoisoflavone B | 48.78 | 0.55 | 129280-33-7 | LWZACZCRAUQSLH-UHFFFAOYSA-N |
| *Licorice* | | | Gancao | MOL004828 | Glepidotin A | 44.72 | 0.35 | 42193-83-9 | WCSHKPNHOSDFGK-UHFFFAOYSA-N |
| *Licorice* | | | Gancao | MOL004829 | Glepidotin B | 64.46 | 0.34 | 87440-56-0 | ATJOIGKHVRPLSM-RBUKOAKNSA-N |
| *Licorice* | | | Gancao | MOL004833 | Phaseolinisoflavan | 32.01 | 0.45 | 40323-57-7 | UUJBHSNXZMGYBT-ZDUSSCGKSA-N |
| *Licorice* | | | Gancao | MOL004835 | Glypallichalcone | 61.6 | 0.19 | 146763-58-8 | CTBBBAQHUJVKNG-UXBLZVDNSA-N |
| *Licorice* | | | Gancao | MOL004838 | 8-(6-hydroxy-2-benzofuranyl)-2,2-dimethyl-5-chromenol | 58.44 | 0.38 | N/A | SJIZTMNAKZAOTA-UHFFFAOYSA-N |
| *Licorice* | | | Gancao | MOL004841 | Licochalcone B | 76.76 | 0.19 | 58749-23-8 | DRDRYGIIYOPBBZ-XBXARRHUSA-N |
| *Licorice* | | | Gancao | MOL004848 | licochalcone G | 49.25 | 0.32 | N/A | UYRRMZSSCNIGNC-RMKNXTFCSA-N |
| *Licorice* | | | Gancao | MOL004849 | 3-(2,4-dihydroxyphenyl)-8-(1,1-dimethylprop-2-enyl)-7-hydroxy-5-methoxy-coumarin | 59.62 | 0.43 | N/A | LCRIQVFKVCYUAO-UHFFFAOYSA-N |
| *Licorice* | | | Gancao | MOL004855 | Licoricone | 63.58 | 0.47 | 51847-92-8 | GGWMNTNDTRKETA-UHFFFAOYSA-N |
| *Licorice* | | | Gancao | MOL004856 | Gancaonin A | 51.08 | 0.4 | 27762-99-8 | JQNSUDIGIIGIOL-UHFFFAOYSA-N |
| *Licorice* | | | Gancao | MOL004857 | Gancaonin B | 48.79 | 0.45 | 124596-86-7 | YQEPOQVRUDADPH-UHFFFAOYSA-N |
| *Licorice* | | | Gancao | MOL004863 | 3-(3,4-dihydroxyphenyl)-5,7-dihydroxy-8-(3-methylbut-2-enyl)chromone | 66.37 | 0.41 | 129145-50-2 | WSOHPJFMARQRFD-UHFFFAOYSA-N |
| *Licorice* | | | Gancao | MOL004864 | 5,7-dihydroxy-3-(4-methoxyphenyl)-8-(3-methylbut-2-enyl)chromone | 30.49 | 0.41 | 129145-51-3 | DDLPIQXHEKZHQX-UHFFFAOYSA-N |
| *Licorice* | | | Gancao | MOL004866 | 2-(3,4-dihydroxyphenyl)-5,7-dihydroxy-6-(3-methylbut-2-enyl)chromone | 44.15 | 0.41 | 129145-53-5 | AFJYQKPCJLMHCC-UHFFFAOYSA-N |
| *Licorice* | | | Gancao | MOL004879 | Glycyrin | 52.61 | 0.47 | 66056-18-6 | FWWGXZYUURXJLK-UHFFFAOYSA-N |
| *Licorice* | | | Gancao | MOL004882 | Licocoumarone | 33.21 | 0.36 | 118524-14-4 | CNPMAFLUEHEXRE-UHFFFAOYSA-N |
| *Licorice* | | | Gancao | MOL004883 | Licoisoflavone | 41.61 | 0.42 | 66056-19-7 | KCUZCRLRQVRBBV-UHFFFAOYSA-N |
| *Licorice* | | | Gancao | MOL004884 | Licoisoflavone B | 38.93 | 0.55 | 66056-30-2 | KIZPADOTOCPASX-UHFFFAOYSA-N |
| *Licorice* | | | Gancao | MOL004885 | licoisoflavanone | 52.47 | 0.54 | 66067-26-3 | JNDPLDZUOFZXIG-ZDUSSCGKSA-N |
| *Licorice* | | | Gancao | MOL004891 | shinpterocarpin | 80.3 | 0.73 | 157414-04-5 | QGPHRCQDTPCIQI-KXBFYZLASA-N |
| *Licorice* | | | Gancao | MOL004898 | (E)-3-[3,4-dihydroxy-5-(3-methylbut-2-enyl)phenyl]-1-(2,4-dihydroxyphenyl)prop-2-en-1-one | 46.27 | 0.31 | N/A | XHTOWVVXFDSBGH-XBXARRHUSA-N |
| *Licorice* | | | Gancao | MOL004903 | liquiritin | 65.69 | 0.74 | 31564-20-2 551-15-5 100483-53-2 17654-21-6 30372-08-8 | DEMKZLAVQYISIA-ZRWXNEIDSA-N |
| *Licorice* | | | Gancao | MOL004904 | licopyranocoumarin | 80.36 | 0.65 | 1564-20-2 551-15-5 100483-53-2 17654-21-6 30372-08-8 | MOBCUWLJOZHPQL-OAQYLSRUSA-N |
| *Licorice* | | | Gancao | MOL004907 | Glyzaglabrin | 61.07 | 0.35 | N/A | GRMSSCUVELGNHC-UHFFFAOYSA-N |
| *Licorice* | | | Gancao | MOL004908 | Glabridin | 53.25 | 0.47 | 59870-68-7 | LBQIJVLKGVZRIW-ZDUSSCGKSA-N |
| *Licorice* | | | Gancao | MOL004910 | Glabranin | 52.9 | 0.31 | 41983-91-9 | DAWSYIQAGQMLFS-SFHVURJKSA-N |
| *Licorice* | | | Gancao | MOL004911 | Glabrene | 46.27 | 0.44 | 60008-03-9 | NGGYSPUAKQMTNP-UHFFFAOYSA-N |
| *Licorice* | | | Gancao | MOL004912 | Glabrone | 52.51 | 0.5 | 60008-02-8 | COLMVFWKLOZOOP-UHFFFAOYSA-N |
| *Licorice* | | | Gancao | MOL004913 | 1,3-dihydroxy-9-methoxy-6-benzofurano[3,2-c]chromenone | 48.14 | 0.43 | N/A | AAKHRTZXSZBLFQ-UHFFFAOYSA-N |
| *Licorice* | | | Gancao | MOL004914 | 1,3-dihydroxy-8,9-dimethoxy-6-benzofurano[3,2-c]chromenone | 62.9 | 0.53 | N/A | FBPDUHIOILHCFT-UHFFFAOYSA-N |
| *Licorice* | | | Gancao | MOL004915 | Eurycarpin A | 43.28 | 0.37 | N/A | NNCFAUGCNTZUIW-UHFFFAOYSA-N |
| *Licorice* | | | Gancao | MOL004924 | (-)-Medicocarpin | 40.99 | 0.95 | N/A | PVEMGMOWXQUWRD-NJAOXFEXSA-N |
| *Licorice* | | | Gancao | MOL004935 | Sigmoidin-B | 34.88 | 0.41 | 87746-47-2 | SFQIGPZCFNTPOD-KRWDZBQOSA-N |
| *Licorice* | | | Gancao | MOL004941 | (2R)-7-hydroxy-2-(4-hydroxyphenyl)chroman-4-one | 71.12 | 0.18 | 578-86-9  5088-75-5  41680-09-5 | FURUXTVZLHCCNA-CQSZACIVSA-N |
| *Licorice* | | | Gancao | MOL004945 | (2S)-7-hydroxy-2-(4-hydroxyphenyl)-8-(3-methylbut-2-enyl)chroman-4-one | 36.57 | 0.32 | 31524-62-6 | KYFBXCHUXFKMGQ-IBGZPJMESA-N |
| *Licorice* | | | Gancao | MOL004948 | Isoglycyrol | 44.7 | 0.84 | 23013-86-7 | CFWLRXJPRRCJTI-UHFFFAOYSA-N |
| *Licorice* | | | Gancao | MOL004949 | Isolicoflavonol | 45.17 | 0.42 | 94805-83-1 | PGCKDCPTJAQQSQ-UHFFFAOYSA-N |
| *Licorice* | | | Gancao | MOL004957 | HMO | 38.37 | 0.21 | N/A | LNIQZRIHAMVRJA-UHFFFAOYSA-N |
| *Licorice* | | | Gancao | MOL004959 | 1-Methoxyphaseollidin | 69.98 | 0.64 | N/A | YKTZRMXYANFKQR-YCRPNKLZSA-N |
| *Licorice* | | | Gancao | MOL004961 | Quercetin der. | 46.45 | 0.33 | 4382-17-6 | FMEHGPQTMOPUGM-UHFFFAOYSA-N |
| *Licorice* | | | Gancao | MOL004966 | 3'-Hydroxy-4'-O-Methylglabridin | 43.71 | 0.57 | N/A | PPBISUGOQDBBEL-ZDUSSCGKSA-N |
| *Licorice* | | | Gancao | MOL000497 | licochalcone a | 40.79 | 0.29 | 58749-22-7 | KAZSKMJFUPEHHW-DHZHZOJOSA-N |
| *Licorice* | | | Gancao | MOL004974 | 3'-Methoxyglabridin | 46.16 | 0.57 | N/A | SBQBKTSYEKPBJF-ZDUSSCGKSA-N |
| *Licorice* | | | Gancao | MOL004978 | 2-[(3R)-8,8-dimethyl-3,4-dihydro-2H-pyrano[6,5-f]chromen-3-yl]-5-methoxypheno | 36.21 | 0.52 | N/A | ZZAIPFIGEGQNHP-AWEZNQCLSA-N |
| *Licorice* | | | Gancao | MOL004980 | Inflacoumarin A | 39.71 | 0.33 | 158446-33-4 | RNBLSJGPSGNSIN-UHFFFAOYSA-N |
| *Licorice* | | | Gancao | MOL004985 | icos-5-enoic acid | 30.7 | 0.2 | N/A | FPAQLJHSZVFKES-FOCLMDBBSA-N |
| *Licorice* | | | Gancao | MOL004988 | Kanzonol F | 32.47 | 0.89 | N/A | SVHCNENPWOPFOI-BVZFJXPGSA-N |
| *Licorice* | | | Gancao | MOL004989 | 6-prenylated eriodictyol | 39.22 | 0.41 | N/A | KBEFFXBSHFUQLT-QGZVFWFLSA-N |
| *Licorice* | | | Gancao | MOL004990 | 7,2',4'-trihydroxy－5-methoxy-3－arylcoumarin | 83.71 | 0.27 | N/A | FTOHMMMSWYNATM-UHFFFAOYSA-N |
| *Licorice* | | | Gancao | MOL004991 | 7-Acetoxy-2-methylisoflavone | 38.92 | 0.26 | 3211-63-0 | DPIAJERHFDBLPT-UHFFFAOYSA-N |
| *Licorice* | | | Gancao | MOL004993 | 8-prenylated eriodictyol | 53.79 | 0.4 | N/A | XJUDJFVOICLOMY-GOSISDBHSA-N |
| *Licorice* | | | Gancao | MOL004996 | gadelaidic acid | 30.7 | 0.2 | 506-31-0 | LQJBNNIYVWPHFW-VAWYXSNFSA-N |
| *Licorice* | | | Gancao | MOL000500 | Vestitol | 74.66 | 0.21 | 20879-05-4 | XRVFNNUXNVWYTI-LLVKDONJSA-N |
| *Licorice* | | | Gancao | MOL005000 | Gancaonin G | 60.44 | 0.39 | 126716-34-5 | WLPHLDLTTPUDSI-UHFFFAOYSA-N |
| *Licorice* | | | Gancao | MOL005001 | Gancaonin H | 50.1 | 0.78 | 126716-35-6 | CCUZOQMNQONIMX-UHFFFAOYSA-N |
| *Licorice* | | | Gancao | MOL005003 | Licoagrocarpin | 58.81 | 0.58 | N/A | NYWUHXBEDBSRQB-UWJYYQICSA-N |
| *Licorice* | | | Gancao | MOL005007 | Glyasperins M | 72.67 | 0.59 | N/A | ZIDJFMZHWCAFPM-AWEZNQCLSA-N |
| *Licorice* | | | Gancao | MOL005008 | Glycyrrhiza flavonol A | 41.28 | 0.6 | N/A | UFWHTSBKDGUFOX-OAHLLOKOSA-N |
| *Licorice* | | | Gancao | MOL005012 | Licoagroisoflavone | 57.28 | 0.49 | N/A | CQXOSSCKEFRIBR-OAHLLOKOSA-N |
| *Licorice* | | | Gancao | MOL005016 | Odoratin | 49.95 | 0.3 | N/A | BYNYZQQDQIQLSO-UHFFFAOYSA-N |
| *Licorice* | | | Gancao | MOL005017 | Phaseol | 78.77 | 0.58 | N/A | FRXPSBUCIWPZMH-UHFFFAOYSA-N |
| *Licorice* | | | Gancao | MOL005018 | Xambioona | 54.85 | 0.87 | N/A | FGJUXFVUOCKRCY-QFIPXVFZSA-N |
| *Licorice* | | | Gancao | MOL005020 | dehydroglyasperins C | 53.82 | 0.37 | N/A | DRUAOMUKHSJILC-UHFFFAOYSA-N |
| *Licorice* | | | Gancao | MOL000098 | quercetin | 46.43 | 0.28 | 73123-10-1  74893-81-5  117-39-5 | REFJWTPEDVJJIY-UHFFFAOYSA-N |
| *Isatidis Radix* | | | Banlangen | MOL001689 | acacetin | 34.97 | 0.24 | 480-44-4 | DANYIYRPLHHOCZ-UHFFFAOYSA-N |
| *Isatidis Radix* | | | Banlangen | MOL002322 | isovitexin | 31.29 | 0.72 | 61838-34-4 61383-34-4  29702-25-8  38953-85-4 | MYXNWGACZJSMBT-VJXVFPJBSA-N |
| *Isatidis Radix* | | | Banlangen | MOL001721 | Isaindigodione | 60.12 | 0.41 | N/A | QQDWEMDBMCNRPU-VOJFVSQTSA-N |
| *Isatidis Radix* | | | Banlangen | MOL001722 | 2-O-beta-D-glucopyranosyl-2H-1,4-benzoxazin-3(4H)-one | 43.62 | 0.31 | N/A | PYQSUTLVBSTCSK-TWTZXXGESA-N |
| *Isatidis Radix* | | | Banlangen | MOL001733 | EUPATORIN | 30.23 | 0.37 | 855-96-9 | KLAOKWJLUQKWIF-UHFFFAOYSA-N |
| *Isatidis Radix* | | | Banlangen | MOL001734 | 3-[[(2R,3R,5R,6S)-3,5-dihydroxy-6-(1H-indol-3-yloxy)-4-oxooxan-2-yl]methoxy]-3-oxopropanoic acid | 85.87 | 0.47 | N/A | HZZHOGRFRUCYQW-QQDQCYIDSA-N |
| *Isatidis Radix* | | | Banlangen | MOL001735 | Dinatin | 30.97 | 0.27 | 1447-88-7 | IHFBPDAQLQOCBX-UHFFFAOYSA-N |
| *Isatidis Radix* | | | Banlangen | MOL001736 | (-)-taxifolin | 60.51 | 0.27 | 480-18-2  17654-26-1  29838-67-3 | CXQWRCVTCMQVQX-CABCVRRESA-N |
| *Isatidis Radix* | | | Banlangen | MOL001749 | ZINC03860434 | 43.59 | 0.35 | 117-81-7  8033-53-2  40120-69-2  50885-87-5  275818-89-8  607374-50-5 | BJQHLKABXJIVAM-WOJBJXKFSA-N |
| *Isatidis Radix* | | | Banlangen | MOL001750 | glucobrassicin | 66.02 | 0.48 | 4356-52-9 | DNDNWOWHUWNBCK-NJACVBLOSA-N |
| *Isatidis Radix* | | | Banlangen | MOL001755 | 24-Ethylcholest-4-en-3-one | 36.08 | 0.76 | 67392-96-5 | RUVUHIUYGJBLGI-XJZKHKOHSA-N |
| *Isatidis Radix* | | | Banlangen | MOL001756 | quindoline | 33.17 | 0.22 | 243-58-3 | QOAKRWLMTKEDDL-UHFFFAOYSA-N |
| *Isatidis Radix* | | | Banlangen | MOL001767 | hydroxyindirubin | 63.37 | 0.3 | N/A | IMMCEXDJRKGRBS-YPKPFQOOSA-N |
| *Isatidis Radix* | | | Banlangen | MOL001771 | poriferast-5-en-3beta-ol | 36.91 | 1.14 | 83-46-5 83-47-6  201-481-1  5779-62-4  19044-06-5 | KZJWDPNRJALLNS-FBZNIEFRSA-N |
| *Isatidis Radix* | | | Banlangen | MOL001774 | Ineketone | 37.14 | 0.3 | 62574-18-9 | BWRPYSJNBVBIRP-FLFBIERCSA-N |
| *Isatidis Radix* | | | Banlangen | MOL001779 | Sinoacutine | 49.11 | 0.46 | 17039-45-1 1936-18-1 | GVTRUVGBZQJVTF-YJYMSZOUSA-N |
| *Isatidis Radix* | | | Banlangen | MOL001781 | Indigo | 38.2 | 0.26 | 12000-74-7  12626-73-2  93660-98-1  68651-46-7  11129-41-2 | COHYTHOBJLSHDF-BUHFOSPRSA-N |
| *Isatidis Radix* | | | Banlangen | MOL001782 | (2Z)-2-(2-oxoindolin-3-ylidene)indolin-3-one | 48.4 | 0.26 | 479-41-4 | CRDNMYFJWFXOCH-YPKPFQOOSA-N |
| *Isatidis Radix* | | | Banlangen | MOL001783 | 2-(9-((3-methyl-2-oxopent-3-en-1-yl)oxy)-2-oxo-1,2,8,9-tetrahydrofuro[2,3-h]quinolin-8-yl)propan-2-yl acetate | 64 | 0.57 | N/A | FDQWKLKMJSBEJA-RAXKFLFSSA-N |
| *Isatidis Radix* | | | Banlangen | MOL001790 | Linarin | 39.84 | 0.71 | 480-36-4 | YFVGIJBUXMQFOF-PJOVQGMDSA-N |
| *Isatidis Radix* | | | Banlangen | MOL001792 | DFV | 32.76 | 0.18 | 578-86-9  16006-91-0  17002-53-8  2885-30-5 | FURUXTVZLHCCNA-AWEZNQCLSA-N |
| *Isatidis Radix* | | | Banlangen | MOL001793 | (E)-2-[(3-indole)cyanomethylene-]-3-indolinone | 54.59 | 0.32 | N/A | IHDMETHUVCEFTN-ZCXUNETKSA-N |
| *Isatidis Radix* | | | Banlangen | MOL001798 | neohesperidin_qt | 71.17 | 0.27 | 13241-33-3 | YNYGAMJOLICYES-AWEZNQCLSA-N |
| *Isatidis Radix* | | | Banlangen | MOL001800 | rosasterol | 35.87 | 0.75 | N/A | BBTIMXAYZRWPNG-ITNOYKGOSA-N |
| *Isatidis Radix* | | | Banlangen | MOL001803 | Sinensetin | 50.56 | 0.45 | 2306-27-6  27181-91-5 | LKMNXYDUQXAUCZ-UHFFFAOYSA-N |
| *Isatidis Radix* | | | Banlangen | MOL001804 | Stigmasta-5,22-diene-3beta,7alpha-diol | 43.04 | 0.82 | N/A | XAWFYPABHPYOBL-RSMPLCKASA-N |
| *Isatidis Radix* | | | Banlangen | MOL001810 | 6-(3-oxoindolin-2-ylidene)indolo[2,1-b]quinazolin-12-one | 45.28 | 0.89 | 97457-31-3 | DXENDDMPDZMHSQ-FMQUCBEESA-N |
| *Isatidis Radix* | | | Banlangen | MOL001814 | (E)-3-(3,5-dimethoxy-4-hydroxy-benzylidene)-2-indolinone | 57.18 | 0.25 | N/A | HXBOVHHWZNXQOG-NXVVXOECSA-N |
| *Isatidis Radix* | | | Banlangen | MOL001820 | (E)-3-(3,5-dimethoxy-4-hydroxyb-enzylidene)-2-indolinone | 65.17 | 0.25 | N/A | UWPXXGPTDSEESY-MRXNPFEDSA-N |
| *Isatidis Radix* | | | Banlangen | MOL001828 | 3-[(3,5-dimethoxy-4-oxo-1-cyclohexa-2,5-dienylidene)methyl]-2,4-dihydro-1H-pyrrolo[2,1-b]quinazolin-9-one | 51.84 | 0.56 | N/A | MGPNFYJFABOVFP-UHFFFAOYSA-N |
| *Isatidis Radix* | | | Banlangen | MOL001833 | Glucobrassicin-1-Sulfonate_qt | 42.52 | 0.24 | N/A | CKXSUNNEIDPPAW-UHFFFAOYSA-N |
| *Isatidis Radix* | | | Banlangen | MOL000358 | beta-sitosterol | 36.91 | 0.75 | 83-46-5 | KZJWDPNRJALLNS-VJSFXXLFSA-N |
| *Isatidis Radix* | | | Banlangen | MOL000359 | sitosterol | 36.91 | 0.75 | 83-46-5 149-91-7 474-58-8 915-05-9 5779-62-4 64997-52-0 | KZJWDPNRJALLNS-ZFVHJZABSA-N |
| *Isatidis Radix* | | | Banlangen | MOL000449 | Stigmasterol | 43.83 | 0.76 | 83-48-7 | HCXVJBMSMIARIN-PHZDYDNGSA-N |
| *Isatidis Radix* | | | Banlangen | MOL000953 | CLR | 37.87 | 0.68 | 80356-14-5  22243-67-0  57-88-5  209124-38-9  218965-24-3 | HVYWMOMLDIMFJA-DPAQBDIFSA-N |
| *Lonicerae Japonicae Flos* | | | Jinyinhua | MOL001494 | Mandenol | 42 | 0.19 | 544-35-4 | FMMOOAYVCKXGMF-MURFETPASA-N |
| *Lonicerae Japonicae Flos* | | | Jinyinhua | MOL001495 | Ethyl linolenate | 46.1 | 0.2 | 1191-41-9  34079-45-3 | JYYFMIOPGOFNPK-XSHSMGBESA-N |
| *Lonicerae Japonicae Flos* | | | Jinyinhua | MOL002914 | Eriodyctiol (flavanone) | 41.35 | 0.24 | 4049-38-1 | SBHXYTNGIZCORC-CYBMUJFWSA-N |
| *Lonicerae Japonicae Flos* | | | Jinyinhua | MOL003006 | (-)-(3R,8S,9R,9aS,10aS)-9-ethenyl-8-(beta-D-glucopyranosyloxy)-2,3,9,9a,10,10a-hexahydro-5-oxo-5H,8H-pyrano[4,3-d]oxazolo[3,2-a]pyridine-3-carboxylic acid_qt | 87.47 | 0.23 | N/A | CJISYUMYHKJZBF-HZOXHFDSSA-N |
| *Lonicerae Japonicae Flos* | | | Jinyinhua | MOL003014 | secologanic dibutylacetal_qt | 53.65 | 0.29 | N/A | MQMIOCHUWUGSPS-LLGFUMIMSA-N |
| *Lonicerae Japonicae Flos* | | | Jinyinhua | MOL002773 | beta-carotene | 37.18 | 0.58 | 7235-40-7  116-32-5  31797-85-0 | OENHQHLEOONYIE-JLTXGRSLSA-N |
| *Lonicerae Japonicae Flos* | | | Jinyinhua | MOL003036 | ZINC03978781 | 43.83 | 0.76 | 19716-26-8 | HCXVJBMSMIARIN-NKMAIEQZSA-N |
| *Lonicerae Japonicae Flos* | | | Jinyinhua | MOL003044 | Chryseriol | 35.85 | 0.27 | 491-71-4 | SCZVLDHREVKTSH-UHFFFAOYSA-N |
| *Lonicerae Japonicae Flos* | | | Jinyinhua | MOL003095 | 5-hydroxy-7-methoxy-2-(3,4,5-trimethoxyphenyl)chromone | 51.96 | 0.41 | 18103-41-8 | FLCVGMVLNHYJAW-UHFFFAOYSA-N |
| *Lonicerae Japonicae Flos* | | | Jinyinhua | MOL003111 | Centauroside_qt | 55.79 | 0.5 | N/A | LTDRLWUNCZGQEM-NBWBJBPESA-N |
| *Lonicerae Japonicae Flos* | | | Jinyinhua | MOL003117 | Ioniceracetalides B_qt | 61.19 | 0.19 | N/A | HOJAQTBWHRNJPB-XKJBLICVSA-N |
| *Lonicerae Japonicae Flos* | | | Jinyinhua | MOL003128 | dinethylsecologanoside | 48.46 | 0.48 | 25488-59-9 | STDANUIGVXEGDJ-VPNMNMBESA-N |
| *Lonicerae Japonicae Flos* | | | Jinyinhua | MOL000358 | beta-sitosterol | 36.91 | 0.75 | 83-46-5 | KZJWDPNRJALLNS-VJSFXXLFSA-N |
| *Lonicerae Japonicae Flos* | | | Jinyinhua | MOL000422 | kaempferol | 41.88 | 0.24 | 520-18-3 | IYRMWMYZSQPJKC-UHFFFAOYSA-N |
| *Lonicerae Japonicae Flos* | | | Jinyinhua | MOL000449 | Stigmasterol | 43.83 | 0.76 | 83-48-7 | HCXVJBMSMIARIN-PHZDYDNGSA-N |
| *Lonicerae Japonicae Flos* | | | Jinyinhua | MOL000006 | luteolin | 36.16 | 0.25 | 491-70-3 | IQPNAANSBPBGFQ-UHFFFAOYSA-N |
| *Lonicerae Japonicae Flos* | | | Jinyinhua | MOL000098 | quercetin | 46.43 | 0.28 | 73123-10-1  74893-81-5  117-39-5 | REFJWTPEDVJJIY-UHFFFAOYSA-N |
| *Hedysarum Multijugum Maxim* | | | Huangqi | MOL000211 | Mairin | 55.38 | 0.78 | 472-15-1 | QGJZLNKBHJESQX-FZFNOLFKSA-N |
| *Hedysarum Multijugum Maxim* | | | Huangqi | MOL000239 | Jaranol | 50.83 | 0.29 | 3301-49-3 | BJBUTJQYZDYRMJ-UHFFFAOYSA-N |
| *Hedysarum Multijugum Maxim* | | | Huangqi | MOL000296 | hederagenin | 36.91 | 0.75 | 465-99-6 474-58-8 | KZJWDPNRJALLNS-CQXWNKEUSA-N |
| *Hedysarum Multijugum Maxim* | | | Huangqi | MOL000033 | (3S,8S,9S,10R,13R,14S,17R)-10,13-dimethyl-17-[(2R,5S)-5-propan-2-yloctan-2-yl]-2,3,4,7,8,9,11,12,14,15,16,17-dodecahydro-1H-cyclopenta[a]phenanthren-3-ol | 36.23 | 0.78 | 64997-52-0 | KLEXDBGYSOIREE-UIFQYPGESA-N |
| *Hedysarum Multijugum Maxim* | | | Huangqi | MOL000354 | isorhamnetin | 49.6 | 0.31 | 480-19-3 | IZQSVPBOUDKVDZ-UHFFFAOYSA-N |
| *Hedysarum Multijugum Maxim* | | | Huangqi | MOL000371 | 3,9-di-O-methylnissolin | 53.74 | 0.48 | N/A | RFFNFQZKHNKOPO-BBRMVZONSA-N |
| *Hedysarum Multijugum Maxim* | | | Huangqi | MOL000378 | 7-O-methylisomucronulatol | 74.69 | 0.3 | N/A | BLHQCBJSTMDZQA-LBPRGKRZSA-N |
| *Hedysarum Multijugum Maxim* | | | Huangqi | MOL000379 | 9,10-dimethoxypterocarpan-3-O-β-D-glucoside | 36.74 | 0.92 | 94367-42-7 | PCIXSTFFMHVOMF-PBGSHFJYSA-N |
| *Hedysarum Multijugum Maxim* | | | Huangqi | MOL000380 | (6aR,11aR)-9,10-dimethoxy-6a,11a-dihydro-6H-benzofurano[3,2-c]chromen-3-ol | 64.26 | 0.42 | 73340-41-7  94367-42-7 | UOVGCLXUTLXAEC-WFASDCNBSA-N |
| *Hedysarum Multijugum Maxim* | | | Huangqi | MOL000387 | Bifendate | 31.1 | 0.67 | 73536-69-3 | JMZOMFYRADAWOG-UHFFFAOYSA-N |
| *Hedysarum Multijugum Maxim* | | | Huangqi | MOL000392 | formononetin | 69.67 | 0.21 | 485-72-3 | HKQYGTCOTHHOMP-UHFFFAOYSA-N |
| *Hedysarum Multijugum Maxim* | | | Huangqi | MOL000417 | Calycosin | 47.75 | 0.24 | 20575-57-9 | ZZAJQOPSWWVMBI-UHFFFAOYSA-N |
| *Hedysarum Multijugum Maxim* | | | Huangqi | MOL000422 | kaempferol | 41.88 | 0.24 | 520-18-3 | IYRMWMYZSQPJKC-UHFFFAOYSA-N |
| *Hedysarum Multijugum Maxim* | | | Huangqi | MOL000433 | FA | 68.96 | 0.71 | 33609-88-0 59-30-3 | OVBPIULPVIDEAO-LBPRGKRZSA-N |
| *Hedysarum Multijugum Maxim* | | | Huangqi | MOL000439 | isomucronulatol-7,2'-di-O-glucosiole | 49.28 | 0.62 | N/A | NHOPAJCVMDIGBN-MEPKZADGSA-N |
| *Hedysarum Multijugum Maxim* | | | Huangqi | MOL000442 | 1,7-Dihydroxy-3,9-dimethoxy pterocarpene | 39.05 | 0.48 | N/A | RVGZSUMTFIEORY-UHFFFAOYSA-N |
| *Hedysarum Multijugum Maxim* | | | Huangqi | MOL000098 | quercetin | 46.43 | 0.28 | 73123-10-1  74893-81-5  117-39-5 | REFJWTPEDVJJIY-UHFFFAOYSA-N |
|  | |  |  |  |  |  |  |  |  |
| Herbal Name | Compounds Number | | |  |  |  |  |  |  |
| Jinyinhua (JYH) | 17 | | |  |  |  |  |  |  |
| Gancao (GC) | 88 | | |  |  |  |  |  |  |
| Banlangen (BLG) | 35 | | |  |  |  |  |  |  |
| Guanzhong (GZ) | 5 | | |  |  |  |  |  |  |
| Huangqi (HQ) | 17 | | |  |  |  |  |  |  |


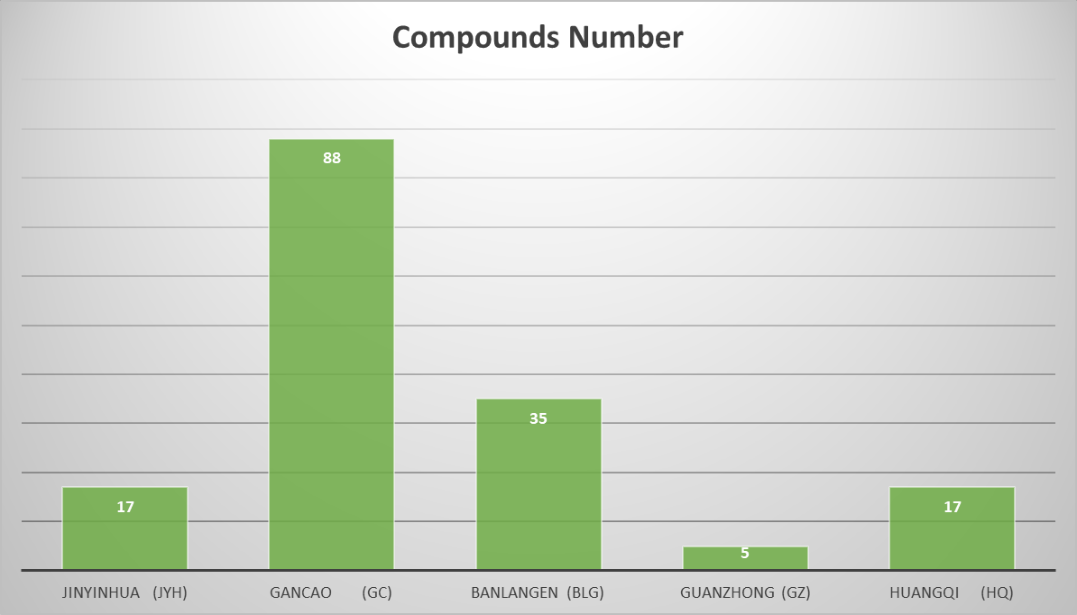


| **Supplementary file1: Table S2. all active compounds and related targets** | | | |  |
| --- | --- | --- | --- | --- |
| **Latin name** | **Chinese name** | **Molecular ID** | **Related targets** | **Symbol** |
| Fortunes Bossfern Rhizome | Guanzhong | MOL002614 | Acetylcholinesterase | ACHE |
| Fortunes Bossfern Rhizome | Guanzhong | MOL000422 | Acetylcholinesterase | ACHE |
| Fortunes Bossfern Rhizome | Guanzhong | MOL002614 | Alpha-1A adrenergic receptor | ADRA1A |
| Fortunes Bossfern Rhizome | Guanzhong | MOL002605 | Alpha-1B adrenergic receptor | ADRA1B |
| Fortunes Bossfern Rhizome | Guanzhong | MOL000422 | Alpha-1B adrenergic receptor | ADRA1B |
| Fortunes Bossfern Rhizome | Guanzhong | MOL002614 | Alpha-1D adrenergic receptor | ADRA1D |
| Fortunes Bossfern Rhizome | Guanzhong | MOL002610 | Beta-2 adrenergic receptor | ADRB2 |
| Fortunes Bossfern Rhizome | Guanzhong | MOL002614 | Beta-2 adrenergic receptor | ADRB2 |
| Fortunes Bossfern Rhizome | Guanzhong | MOL000422 | Aryl hydrocarbon receptor | AHR |
| Fortunes Bossfern Rhizome | Guanzhong | MOL000422 | Activator of 90 kDa heat shock protein ATPase homolog 1 | AHSA1 |
| Fortunes Bossfern Rhizome | Guanzhong | MOL000422 | Aldo-keto reductase family 1 member C3 | AKR1C3 |
| Fortunes Bossfern Rhizome | Guanzhong | MOL000422 | RAC-alpha serine/threonine-protein kinase | AKT1 |
| Fortunes Bossfern Rhizome | Guanzhong | MOL000422 | Arachidonate 5-lipoxygenase | ALOX5 |
| Fortunes Bossfern Rhizome | Guanzhong | MOL000422 | Androgen receptor | AR |
| Fortunes Bossfern Rhizome | Guanzhong | MOL000422 | Apoptosis regulator BAX | BAX |
| Fortunes Bossfern Rhizome | Guanzhong | MOL000422 | Apoptosis regulator Bcl-2 | BCL2 |
| Fortunes Bossfern Rhizome | Guanzhong | MOL000422 | Calmodulin | CALM1 |
| Fortunes Bossfern Rhizome | Guanzhong | MOL000422 | Caspase-3 | CASP3 |
| Fortunes Bossfern Rhizome | Guanzhong | MOL000422 | Cell division control protein 2 homolog | CDK1 |
| Fortunes Bossfern Rhizome | Guanzhong | MOL002605 | Muscarinic acetylcholine receptor M1 | CHRM1 |
| Fortunes Bossfern Rhizome | Guanzhong | MOL000422 | Muscarinic acetylcholine receptor M1 | CHRM1 |
| Fortunes Bossfern Rhizome | Guanzhong | MOL000422 | Muscarinic acetylcholine receptor M2 | CHRM2 |
| Fortunes Bossfern Rhizome | Guanzhong | MOL002605 | Muscarinic acetylcholine receptor M3 | CHRM3 |
| Fortunes Bossfern Rhizome | Guanzhong | MOL002610 | Muscarinic acetylcholine receptor M3 | CHRM3 |
| Fortunes Bossfern Rhizome | Guanzhong | MOL002614 | Muscarinic acetylcholine receptor M3 | CHRM3 |
| Fortunes Bossfern Rhizome | Guanzhong | MOL002605 | Muscarinic acetylcholine receptor M5 | CHRM5 |
| Fortunes Bossfern Rhizome | Guanzhong | MOL000422 | Cytochrome P450 1A1 | CYP1A1 |
| Fortunes Bossfern Rhizome | Guanzhong | MOL000422 | Cytochrome P450 1A2 | CYP1A2 |
| Fortunes Bossfern Rhizome | Guanzhong | MOL000422 | Cytochrome P450 1B1 | CYP1B1 |
| Fortunes Bossfern Rhizome | Guanzhong | MOL000422 | Cytochrome P450 3A4 | CYP3A4 |
| Fortunes Bossfern Rhizome | Guanzhong | MOL000422 | Type I iodothyronine deiodinase | DIO1 |
| Fortunes Bossfern Rhizome | Guanzhong | MOL000422 | Dipeptidyl peptidase IV | DPP4 |
| Fortunes Bossfern Rhizome | Guanzhong | MOL002605 | D(2) dopamine receptor | DRD2 |
| Fortunes Bossfern Rhizome | Guanzhong | MOL001040 | Estrogen receptor | ESR1 |
| Fortunes Bossfern Rhizome | Guanzhong | MOL002605 | Estrogen receptor | ESR1 |
| Fortunes Bossfern Rhizome | Guanzhong | MOL002610 | Estrogen receptor beta | ESR2 |
| Fortunes Bossfern Rhizome | Guanzhong | MOL000422 | Thrombin | F2 |
| Fortunes Bossfern Rhizome | Guanzhong | MOL000422 | Coagulation factor VII | F7 |
| Fortunes Bossfern Rhizome | Guanzhong | MOL002610 | Gamma-aminobutyric acid receptor subunit alpha-1 | GABRA1 |
| Fortunes Bossfern Rhizome | Guanzhong | MOL000422 | Gamma-aminobutyric acid receptor subunit alpha-1 | GABRA1 |
| Fortunes Bossfern Rhizome | Guanzhong | MOL000422 | Gamma-aminobutyric-acid receptor alpha-2 subunit | GABRA2 |
| Fortunes Bossfern Rhizome | Guanzhong | MOL000422 | Glutathione S-transferase Mu 1 | GSTM1 |
| Fortunes Bossfern Rhizome | Guanzhong | MOL000422 | Glutathione S-transferase Mu 2 | GSTM2 |
| Fortunes Bossfern Rhizome | Guanzhong | MOL000422 | Glutathione S-transferase P | GSTP1 |
| Fortunes Bossfern Rhizome | Guanzhong | MOL000422 | Hyaluronan synthase 2 | HAS2 |
| Fortunes Bossfern Rhizome | Guanzhong | MOL000422 | Heme oxygenase 1 | HMOX1 |
| Fortunes Bossfern Rhizome | Guanzhong | MOL001040 | Heat shock protein HSP 90 | HSP90AB1 |
| Fortunes Bossfern Rhizome | Guanzhong | MOL000422 | Heat shock protein HSP 90 | HSP90AB1 |
| Fortunes Bossfern Rhizome | Guanzhong | MOL002605 | 5-hydroxytryptamine 2A receptor | HTR2A |
| Fortunes Bossfern Rhizome | Guanzhong | MOL002605 | 5-hydroxytryptamine 2C receptor | HTR2C |
| Fortunes Bossfern Rhizome | Guanzhong | MOL000422 | Intercellular adhesion molecule 1 | ICAM1 |
| Fortunes Bossfern Rhizome | Guanzhong | MOL000422 | Inhibitor of nuclear factor kappa-B kinase subunit beta | IKBKB |
| Fortunes Bossfern Rhizome | Guanzhong | MOL000422 | Insulin receptor | INSR |
| Fortunes Bossfern Rhizome | Guanzhong | MOL000422 | Transcription factor AP-1 | JUN |
| Fortunes Bossfern Rhizome | Guanzhong | MOL001040 | Beta-lactamase | LACTB |
| Fortunes Bossfern Rhizome | Guanzhong | MOL000422 | Mitogen-activated protein kinase 8 | MAPK8 |
| Fortunes Bossfern Rhizome | Guanzhong | MOL000422 | Interstitial collagenase | MMP1 |
| Fortunes Bossfern Rhizome | Guanzhong | MOL002614 | Nuclear receptor coactivator 2 | NCOA2 |
| Fortunes Bossfern Rhizome | Guanzhong | MOL000422 | Nuclear receptor coactivator 2 | NCOA2 |
| Fortunes Bossfern Rhizome | Guanzhong | MOL002610 | Nitric oxide synthase, inducible | NOS2 |
| Fortunes Bossfern Rhizome | Guanzhong | MOL000422 | Nitric oxide synthase, inducible | NOS2 |
| Fortunes Bossfern Rhizome | Guanzhong | MOL002610 | Nitric-oxide synthase, endothelial | NOS3 |
| Fortunes Bossfern Rhizome | Guanzhong | MOL002614 | Nitric-oxide synthase, endothelial | NOS3 |
| Fortunes Bossfern Rhizome | Guanzhong | MOL000422 | Nitric-oxide synthase, endothelial | NOS3 |
| Fortunes Bossfern Rhizome | Guanzhong | MOL000422 | Nuclear receptor subfamily 1 group I member 2 | NR1I2 |
| Fortunes Bossfern Rhizome | Guanzhong | MOL000422 | Nuclear receptor subfamily 1 group I member 3 | NR1I3 |
| Fortunes Bossfern Rhizome | Guanzhong | MOL001040 | Glucocorticoid receptor | NR3C1 |
| Fortunes Bossfern Rhizome | Guanzhong | MOL001040 | Mineralocorticoid receptor | NR3C2 |
| Fortunes Bossfern Rhizome | Guanzhong | MOL002605 | Delta-type opioid receptor | OPRD1 |
| Fortunes Bossfern Rhizome | Guanzhong | MOL002605 | Mu-type opioid receptor | OPRM1 |
| Fortunes Bossfern Rhizome | Guanzhong | MOL002610 | Mu-type opioid receptor | OPRM1 |
| Fortunes Bossfern Rhizome | Guanzhong | MOL001040 | Progesterone receptor | PGR |
| Fortunes Bossfern Rhizome | Guanzhong | MOL000422 | Progesterone receptor | PGR |
| Fortunes Bossfern Rhizome | Guanzhong | MOL002610 | Phosphatidylinositol-4,5-bisphosphate 3-kinase catalytic subunit, gamma isoform | PIK3CG |
| Fortunes Bossfern Rhizome | Guanzhong | MOL000422 | Phosphatidylinositol-4,5-bisphosphate 3-kinase catalytic subunit, gamma isoform | PIK3CG |
| Fortunes Bossfern Rhizome | Guanzhong | MOL000422 | Peroxisome proliferator activated receptor gamma | PPARG |
| Fortunes Bossfern Rhizome | Guanzhong | MOL000422 | Peroxisome proliferator-activated receptor gamma | PPARG |
| Fortunes Bossfern Rhizome | Guanzhong | MOL000422 | Serine/threonine-protein phosphatase 2B catalytic subunit alpha isoform | PPP3CA |
| Fortunes Bossfern Rhizome | Guanzhong | MOL000422 | Trypsin-1 | PRSS1 |
| Fortunes Bossfern Rhizome | Guanzhong | MOL000422 | 26S proteasome non-ATPase regulatory subunit 3 | PSMD3 |
| Fortunes Bossfern Rhizome | Guanzhong | MOL001040 | Prostaglandin G/H synthase 1 | PTGS1 |
| Fortunes Bossfern Rhizome | Guanzhong | MOL002610 | Prostaglandin G/H synthase 1 | PTGS1 |
| Fortunes Bossfern Rhizome | Guanzhong | MOL002614 | Prostaglandin G/H synthase 1 | PTGS1 |
| Fortunes Bossfern Rhizome | Guanzhong | MOL000422 | Prostaglandin G/H synthase 1 | PTGS1 |
| Fortunes Bossfern Rhizome | Guanzhong | MOL001040 | Prostaglandin G/H synthase 2 | PTGS2 |
| Fortunes Bossfern Rhizome | Guanzhong | MOL002610 | Prostaglandin G/H synthase 2 | PTGS2 |
| Fortunes Bossfern Rhizome | Guanzhong | MOL002614 | Prostaglandin G/H synthase 2 | PTGS2 |
| Fortunes Bossfern Rhizome | Guanzhong | MOL000422 | Prostaglandin G/H synthase 2 | PTGS2 |
| Fortunes Bossfern Rhizome | Guanzhong | MOL000422 | Transcription factor p65 | RELA |
| Fortunes Bossfern Rhizome | Guanzhong | MOL002610 | Retinoic acid receptor RXR-alpha | RXRA |
| Fortunes Bossfern Rhizome | Guanzhong | MOL002605 | Sodium channel protein type 5 subunit alpha | SCN5A |
| Fortunes Bossfern Rhizome | Guanzhong | MOL002610 | Sodium channel protein type 5 subunit alpha | SCN5A |
| Fortunes Bossfern Rhizome | Guanzhong | MOL000422 | E-selectin | SELE |
| Fortunes Bossfern Rhizome | Guanzhong | MOL000422 | Solute carrier family 2, facilitated glucose transporter member 4 | SLC2A4 |
| Fortunes Bossfern Rhizome | Guanzhong | MOL000422 | Sodium-dependent noradrenaline transporter | SLC6A2 |
| Fortunes Bossfern Rhizome | Guanzhong | MOL000422 | Antileukoproteinase | SLPI |
| Fortunes Bossfern Rhizome | Guanzhong | MOL000422 | Signal transducer and activator of transcription 1-alpha/beta | STAT1 |
| Fortunes Bossfern Rhizome | Guanzhong | MOL000422 | Tumor necrosis factor | TNF |
| Fortunes Bossfern Rhizome | Guanzhong | MOL000422 | DNA topoisomerase II | TOP2B |
| Fortunes Bossfern Rhizome | Guanzhong | MOL000422 | Vascular cell adhesion protein 1 | VCAM1 |
| Fortunes Bossfern Rhizome | Guanzhong | MOL000422 | Xanthine dehydrogenase/oxidase | XDH |
| *Licorice* | Gancao | MOL004328 | 4-aminobutyrate aminotransferase, mitochondrial | ABAT |
| *Licorice* | Gancao | MOL004328 | Multidrug resistance-associated protein 1 | ABCC1 |
| *Licorice* | Gancao | MOL000098 | ATP-binding cassette sub-family G member 2 | ABCG2 |
| *Licorice* | Gancao | MOL000098 | Acetyl-CoA carboxylase 1 | ACACA |
| *Licorice* | Gancao | MOL000354 | Acetylcholinesterase | ACHE |
| *Licorice* | Gancao | MOL003896 | Acetylcholinesterase | ACHE |
| *Licorice* | Gancao | MOL000392 | Acetylcholinesterase | ACHE |
| *Licorice* | Gancao | MOL000422 | Acetylcholinesterase | ACHE |
| *Licorice* | Gancao | MOL004808 | Acetylcholinesterase | ACHE |
| *Licorice* | Gancao | MOL004811 | Acetylcholinesterase | ACHE |
| *Licorice* | Gancao | MOL004824 | Acetylcholinesterase | ACHE |
| *Licorice* | Gancao | MOL004827 | Acetylcholinesterase | ACHE |
| *Licorice* | Gancao | MOL004833 | Acetylcholinesterase | ACHE |
| *Licorice* | Gancao | MOL004856 | Acetylcholinesterase | ACHE |
| *Licorice* | Gancao | MOL004884 | Acetylcholinesterase | ACHE |
| *Licorice* | Gancao | MOL004885 | Acetylcholinesterase | ACHE |
| *Licorice* | Gancao | MOL004904 | Acetylcholinesterase | ACHE |
| *Licorice* | Gancao | MOL004908 | Acetylcholinesterase | ACHE |
| *Licorice* | Gancao | MOL004912 | Acetylcholinesterase | ACHE |
| *Licorice* | Gancao | MOL004924 | Acetylcholinesterase | ACHE |
| *Licorice* | Gancao | MOL004974 | Acetylcholinesterase | ACHE |
| *Licorice* | Gancao | MOL004978 | Acetylcholinesterase | ACHE |
| *Licorice* | Gancao | MOL004991 | Acetylcholinesterase | ACHE |
| *Licorice* | Gancao | MOL005003 | Acetylcholinesterase | ACHE |
| *Licorice* | Gancao | MOL005007 | Acetylcholinesterase | ACHE |
| *Licorice* | Gancao | MOL005008 | Acetylcholinesterase | ACHE |
| *Licorice* | Gancao | MOL000098 | Acetylcholinesterase | ACHE |
| *Licorice* | Gancao | MOL000098 | Prostatic acid phosphatase | ACP3 |
| *Licorice* | Gancao | MOL004328 | Adiponectin | ADIPOQ |
| *Licorice* | Gancao | MOL002565 | Alpha-1A adrenergic receptor | ADRA1A |
| *Licorice* | Gancao | MOL000392 | Alpha-1A adrenergic receptor | ADRA1A |
| *Licorice* | Gancao | MOL000500 | Alpha-1A adrenergic receptor | ADRA1A |
| *Licorice* | Gancao | MOL001484 | Alpha-1B adrenergic receptor | ADRA1B |
| *Licorice* | Gancao | MOL002565 | Alpha-1B adrenergic receptor | ADRA1B |
| *Licorice* | Gancao | MOL003896 | Alpha-1B adrenergic receptor | ADRA1B |
| *Licorice* | Gancao | MOL000422 | Alpha-1B adrenergic receptor | ADRA1B |
| *Licorice* | Gancao | MOL004815 | Alpha-1B adrenergic receptor | ADRA1B |
| *Licorice* | Gancao | MOL004829 | Alpha-1B adrenergic receptor | ADRA1B |
| *Licorice* | Gancao | MOL004833 | Alpha-1B adrenergic receptor | ADRA1B |
| *Licorice* | Gancao | MOL004835 | Alpha-1B adrenergic receptor | ADRA1B |
| *Licorice* | Gancao | MOL004857 | Alpha-1B adrenergic receptor | ADRA1B |
| *Licorice* | Gancao | MOL004891 | Alpha-1B adrenergic receptor | ADRA1B |
| *Licorice* | Gancao | MOL004908 | Alpha-1B adrenergic receptor | ADRA1B |
| *Licorice* | Gancao | MOL004945 | Alpha-1B adrenergic receptor | ADRA1B |
| *Licorice* | Gancao | MOL004959 | Alpha-1B adrenergic receptor | ADRA1B |
| *Licorice* | Gancao | MOL004966 | Alpha-1B adrenergic receptor | ADRA1B |
| *Licorice* | Gancao | MOL000497 | Alpha-1B adrenergic receptor | ADRA1B |
| *Licorice* | Gancao | MOL004974 | Alpha-1B adrenergic receptor | ADRA1B |
| *Licorice* | Gancao | MOL004978 | Alpha-1B adrenergic receptor | ADRA1B |
| *Licorice* | Gancao | MOL004991 | Alpha-1B adrenergic receptor | ADRA1B |
| *Licorice* | Gancao | MOL000500 | Alpha-1B adrenergic receptor | ADRA1B |
| *Licorice* | Gancao | MOL005003 | Alpha-1B adrenergic receptor | ADRA1B |
| *Licorice* | Gancao | MOL001484 | Alpha-1D adrenergic receptor | ADRA1D |
| *Licorice* | Gancao | MOL002565 | Alpha-1D adrenergic receptor | ADRA1D |
| *Licorice* | Gancao | MOL003896 | Alpha-1D adrenergic receptor | ADRA1D |
| *Licorice* | Gancao | MOL004891 | Alpha-1D adrenergic receptor | ADRA1D |
| *Licorice* | Gancao | MOL004959 | Alpha-1D adrenergic receptor | ADRA1D |
| *Licorice* | Gancao | MOL004991 | Alpha-1D adrenergic receptor | ADRA1D |
| *Licorice* | Gancao | MOL003896 | Beta-1 adrenergic receptor | ADRB1 |
| *Licorice* | Gancao | MOL001484 | Beta-2 adrenergic receptor | ADRB2 |
| *Licorice* | Gancao | MOL001792 | Beta-2 adrenergic receptor | ADRB2 |
| *Licorice* | Gancao | MOL002565 | Beta-2 adrenergic receptor | ADRB2 |
| *Licorice* | Gancao | MOL003896 | Beta-2 adrenergic receptor | ADRB2 |
| *Licorice* | Gancao | MOL000392 | Beta-2 adrenergic receptor | ADRB2 |
| *Licorice* | Gancao | MOL000417 | Beta-2 adrenergic receptor | ADRB2 |
| *Licorice* | Gancao | MOL004833 | Beta-2 adrenergic receptor | ADRB2 |
| *Licorice* | Gancao | MOL004835 | Beta-2 adrenergic receptor | ADRB2 |
| *Licorice* | Gancao | MOL004841 | Beta-2 adrenergic receptor | ADRB2 |
| *Licorice* | Gancao | MOL004857 | Beta-2 adrenergic receptor | ADRB2 |
| *Licorice* | Gancao | MOL004866 | Beta-2 adrenergic receptor | ADRB2 |
| *Licorice* | Gancao | MOL004891 | Beta-2 adrenergic receptor | ADRB2 |
| *Licorice* | Gancao | MOL004908 | Beta-2 adrenergic receptor | ADRB2 |
| *Licorice* | Gancao | MOL004911 | Beta-2 adrenergic receptor | ADRB2 |
| *Licorice* | Gancao | MOL004941 | Beta-2 adrenergic receptor | ADRB2 |
| *Licorice* | Gancao | MOL004945 | Beta-2 adrenergic receptor | ADRB2 |
| *Licorice* | Gancao | MOL004957 | Beta-2 adrenergic receptor | ADRB2 |
| *Licorice* | Gancao | MOL004959 | Beta-2 adrenergic receptor | ADRB2 |
| *Licorice* | Gancao | MOL004966 | Beta-2 adrenergic receptor | ADRB2 |
| *Licorice* | Gancao | MOL000497 | Beta-2 adrenergic receptor | ADRB2 |
| *Licorice* | Gancao | MOL004974 | Beta-2 adrenergic receptor | ADRB2 |
| *Licorice* | Gancao | MOL004978 | Beta-2 adrenergic receptor | ADRB2 |
| *Licorice* | Gancao | MOL004980 | Beta-2 adrenergic receptor | ADRB2 |
| *Licorice* | Gancao | MOL004991 | Beta-2 adrenergic receptor | ADRB2 |
| *Licorice* | Gancao | MOL000500 | Beta-2 adrenergic receptor | ADRB2 |
| *Licorice* | Gancao | MOL005003 | Beta-2 adrenergic receptor | ADRB2 |
| *Licorice* | Gancao | MOL005020 | Beta-2 adrenergic receptor | ADRB2 |
| *Licorice* | Gancao | MOL000098 | Beta-2 adrenergic receptor | ADRB2 |
| *Licorice* | Gancao | MOL000422 | Aryl hydrocarbon receptor | AHR |
| *Licorice* | Gancao | MOL000098 | Aryl hydrocarbon receptor | AHR |
| *Licorice* | Gancao | MOL000422 | Activator of 90 kDa heat shock protein ATPase homolog 1 | AHSA1 |
| *Licorice* | Gancao | MOL000098 | Activator of 90 kDa heat shock protein ATPase homolog 1 | AHSA1 |
| *Licorice* | Gancao | MOL000354 | Aldose reductase | AKR1B1 |
| *Licorice* | Gancao | MOL000098 | Aldose reductase | AKR1B1 |
| *Licorice* | Gancao | MOL004328 | Aldo-keto reductase family 1 member C1 | AKR1C1 |
| *Licorice* | Gancao | MOL000422 | Aldo-keto reductase family 1 member C3 | AKR1C3 |
| *Licorice* | Gancao | MOL000422 | RAC-alpha serine/threonine-protein kinase | AKT1 |
| *Licorice* | Gancao | MOL004328 | RAC-alpha serine/threonine-protein kinase | AKT1 |
| *Licorice* | Gancao | MOL000098 | RAC-alpha serine/threonine-protein kinase | AKT1 |
| *Licorice* | Gancao | MOL000422 | Arachidonate 5-lipoxygenase | ALOX5 |
| *Licorice* | Gancao | MOL000098 | Arachidonate 5-lipoxygenase | ALOX5 |
| *Licorice* | Gancao | MOL004328 | Apolipoprotein B-100 | APOB |
| *Licorice* | Gancao | MOL000239 | Androgen receptor | AR |
| *Licorice* | Gancao | MOL000354 | Androgen receptor | AR |
| *Licorice* | Gancao | MOL003656 | Androgen receptor | AR |
| *Licorice* | Gancao | MOL003896 | Androgen receptor | AR |
| *Licorice* | Gancao | MOL000392 | Androgen receptor | AR |
| *Licorice* | Gancao | MOL000417 | Androgen receptor | AR |
| *Licorice* | Gancao | MOL000422 | Androgen receptor | AR |
| *Licorice* | Gancao | MOL004805 | Androgen receptor | AR |
| *Licorice* | Gancao | MOL004808 | Androgen receptor | AR |
| *Licorice* | Gancao | MOL004810 | Androgen receptor | AR |
| *Licorice* | Gancao | MOL004811 | Androgen receptor | AR |
| *Licorice* | Gancao | MOL004814 | Androgen receptor | AR |
| *Licorice* | Gancao | MOL004815 | Androgen receptor | AR |
| *Licorice* | Gancao | MOL004820 | Androgen receptor | AR |
| *Licorice* | Gancao | MOL004824 | Androgen receptor | AR |
| *Licorice* | Gancao | MOL004827 | Androgen receptor | AR |
| *Licorice* | Gancao | MOL004828 | Androgen receptor | AR |
| *Licorice* | Gancao | MOL004833 | Androgen receptor | AR |
| *Licorice* | Gancao | MOL004835 | Androgen receptor | AR |
| *Licorice* | Gancao | MOL004841 | Androgen receptor | AR |
| *Licorice* | Gancao | MOL004848 | Androgen receptor | AR |
| *Licorice* | Gancao | MOL004849 | Androgen receptor | AR |
| *Licorice* | Gancao | MOL004855 | Androgen receptor | AR |
| *Licorice* | Gancao | MOL004856 | Androgen receptor | AR |
| *Licorice* | Gancao | MOL004857 | Androgen receptor | AR |
| *Licorice* | Gancao | MOL004863 | Androgen receptor | AR |
| *Licorice* | Gancao | MOL004864 | Androgen receptor | AR |
| *Licorice* | Gancao | MOL004866 | Androgen receptor | AR |
| *Licorice* | Gancao | MOL004879 | Androgen receptor | AR |
| *Licorice* | Gancao | MOL004882 | Androgen receptor | AR |
| *Licorice* | Gancao | MOL004883 | Androgen receptor | AR |
| *Licorice* | Gancao | MOL004884 | Androgen receptor | AR |
| *Licorice* | Gancao | MOL004885 | Androgen receptor | AR |
| *Licorice* | Gancao | MOL004891 | Androgen receptor | AR |
| *Licorice* | Gancao | MOL004898 | Androgen receptor | AR |
| *Licorice* | Gancao | MOL004904 | Androgen receptor | AR |
| *Licorice* | Gancao | MOL004907 | Androgen receptor | AR |
| *Licorice* | Gancao | MOL004908 | Androgen receptor | AR |
| *Licorice* | Gancao | MOL004911 | Androgen receptor | AR |
| *Licorice* | Gancao | MOL004912 | Androgen receptor | AR |
| *Licorice* | Gancao | MOL004914 | Androgen receptor | AR |
| *Licorice* | Gancao | MOL004915 | Androgen receptor | AR |
| *Licorice* | Gancao | MOL004948 | Androgen receptor | AR |
| *Licorice* | Gancao | MOL004949 | Androgen receptor | AR |
| *Licorice* | Gancao | MOL004957 | Androgen receptor | AR |
| *Licorice* | Gancao | MOL004959 | Androgen receptor | AR |
| *Licorice* | Gancao | MOL004961 | Androgen receptor | AR |
| *Licorice* | Gancao | MOL004966 | Androgen receptor | AR |
| *Licorice* | Gancao | MOL000497 | Androgen receptor | AR |
| *Licorice* | Gancao | MOL004974 | Androgen receptor | AR |
| *Licorice* | Gancao | MOL004978 | Androgen receptor | AR |
| *Licorice* | Gancao | MOL004980 | Androgen receptor | AR |
| *Licorice* | Gancao | MOL004988 | Androgen receptor | AR |
| *Licorice* | Gancao | MOL004990 | Androgen receptor | AR |
| *Licorice* | Gancao | MOL004991 | Androgen receptor | AR |
| *Licorice* | Gancao | MOL000500 | Androgen receptor | AR |
| *Licorice* | Gancao | MOL005000 | Androgen receptor | AR |
| *Licorice* | Gancao | MOL005001 | Androgen receptor | AR |
| *Licorice* | Gancao | MOL005003 | Androgen receptor | AR |
| *Licorice* | Gancao | MOL005007 | Androgen receptor | AR |
| *Licorice* | Gancao | MOL005008 | Androgen receptor | AR |
| *Licorice* | Gancao | MOL005012 | Androgen receptor | AR |
| *Licorice* | Gancao | MOL005016 | Androgen receptor | AR |
| *Licorice* | Gancao | MOL005017 | Androgen receptor | AR |
| *Licorice* | Gancao | MOL005020 | Androgen receptor | AR |
| *Licorice* | Gancao | MOL000098 | Androgen receptor | AR |
| *Licorice* | Gancao | MOL000392 | ATP synthase subunit beta, mitochondrial | ATP5F1B |
| *Licorice* | Gancao | MOL004806 | Beta-secretase | BACE1 |
| *Licorice* | Gancao | MOL004328 | Bcl2 antagonist of cell death | BAD |
| *Licorice* | Gancao | MOL000422 | Apoptosis regulator BAX | BAX |
| *Licorice* | Gancao | MOL000098 | Apoptosis regulator BAX | BAX |
| *Licorice* | Gancao | MOL000422 | Apoptosis regulator Bcl-2 | BCL2 |
| *Licorice* | Gancao | MOL004328 | Apoptosis regulator Bcl-2 | BCL2 |
| *Licorice* | Gancao | MOL000497 | Apoptosis regulator Bcl-2 | BCL2 |
| *Licorice* | Gancao | MOL000098 | Apoptosis regulator Bcl-2 | BCL2 |
| *Licorice* | Gancao | MOL000098 | Bcl-2-like protein 1 | BCL2L1 |
| *Licorice* | Gancao | MOL000098 | Baculoviral IAP repeat-containing protein 5 | BIRC5 |
| *Licorice* | Gancao | MOL004815 | Carbonic anhydrase II | CA2 |
| *Licorice* | Gancao | MOL004835 | Carbonic anhydrase II | CA2 |
| *Licorice* | Gancao | MOL004841 | Carbonic anhydrase II | CA2 |
| *Licorice* | Gancao | MOL000497 | Carbonic anhydrase II | CA2 |
| *Licorice* | Gancao | MOL001484 | Calmodulin | CALM1 |
| *Licorice* | Gancao | MOL000239 | Calmodulin | CALM1 |
| *Licorice* | Gancao | MOL002565 | Calmodulin | CALM1 |
| *Licorice* | Gancao | MOL000354 | Calmodulin | CALM1 |
| *Licorice* | Gancao | MOL003656 | Calmodulin | CALM1 |
| *Licorice* | Gancao | MOL003896 | Calmodulin | CALM1 |
| *Licorice* | Gancao | MOL000392 | Calmodulin | CALM1 |
| *Licorice* | Gancao | MOL000417 | Calmodulin | CALM1 |
| *Licorice* | Gancao | MOL000422 | Calmodulin | CALM1 |
| *Licorice* | Gancao | MOL004805 | Calmodulin | CALM1 |
| *Licorice* | Gancao | MOL004806 | Calmodulin | CALM1 |
| *Licorice* | Gancao | MOL004808 | Calmodulin | CALM1 |
| *Licorice* | Gancao | MOL004810 | Calmodulin | CALM1 |
| *Licorice* | Gancao | MOL004811 | Calmodulin | CALM1 |
| *Licorice* | Gancao | MOL004815 | Calmodulin | CALM1 |
| *Licorice* | Gancao | MOL004820 | Calmodulin | CALM1 |
| *Licorice* | Gancao | MOL004824 | Calmodulin | CALM1 |
| *Licorice* | Gancao | MOL004827 | Calmodulin | CALM1 |
| *Licorice* | Gancao | MOL004828 | Calmodulin | CALM1 |
| *Licorice* | Gancao | MOL004829 | Calmodulin | CALM1 |
| *Licorice* | Gancao | MOL004833 | Calmodulin | CALM1 |
| *Licorice* | Gancao | MOL004835 | Calmodulin | CALM1 |
| *Licorice* | Gancao | MOL004841 | Calmodulin | CALM1 |
| *Licorice* | Gancao | MOL004848 | Calmodulin | CALM1 |
| *Licorice* | Gancao | MOL004849 | Calmodulin | CALM1 |
| *Licorice* | Gancao | MOL004855 | Calmodulin | CALM1 |
| *Licorice* | Gancao | MOL004856 | Calmodulin | CALM1 |
| *Licorice* | Gancao | MOL004857 | Calmodulin | CALM1 |
| *Licorice* | Gancao | MOL004863 | Calmodulin | CALM1 |
| *Licorice* | Gancao | MOL004864 | Calmodulin | CALM1 |
| *Licorice* | Gancao | MOL004866 | Calmodulin | CALM1 |
| *Licorice* | Gancao | MOL004879 | Calmodulin | CALM1 |
| *Licorice* | Gancao | MOL004883 | Calmodulin | CALM1 |
| *Licorice* | Gancao | MOL004884 | Calmodulin | CALM1 |
| *Licorice* | Gancao | MOL004885 | Calmodulin | CALM1 |
| *Licorice* | Gancao | MOL004891 | Calmodulin | CALM1 |
| *Licorice* | Gancao | MOL004898 | Calmodulin | CALM1 |
| *Licorice* | Gancao | MOL004903 | Calmodulin | CALM1 |
| *Licorice* | Gancao | MOL004904 | Calmodulin | CALM1 |
| *Licorice* | Gancao | MOL004908 | Calmodulin | CALM1 |
| *Licorice* | Gancao | MOL004910 | Calmodulin | CALM1 |
| *Licorice* | Gancao | MOL004911 | Calmodulin | CALM1 |
| *Licorice* | Gancao | MOL004912 | Calmodulin | CALM1 |
| *Licorice* | Gancao | MOL004915 | Calmodulin | CALM1 |
| *Licorice* | Gancao | MOL004935 | Calmodulin | CALM1 |
| *Licorice* | Gancao | MOL004941 | Calmodulin | CALM1 |
| *Licorice* | Gancao | MOL004945 | Calmodulin | CALM1 |
| *Licorice* | Gancao | MOL004949 | Calmodulin | CALM1 |
| *Licorice* | Gancao | MOL004957 | Calmodulin | CALM1 |
| *Licorice* | Gancao | MOL004959 | Calmodulin | CALM1 |
| *Licorice* | Gancao | MOL004961 | Calmodulin | CALM1 |
| *Licorice* | Gancao | MOL004966 | Calmodulin | CALM1 |
| *Licorice* | Gancao | MOL000497 | Calmodulin | CALM1 |
| *Licorice* | Gancao | MOL004974 | Calmodulin | CALM1 |
| *Licorice* | Gancao | MOL004978 | Calmodulin | CALM1 |
| *Licorice* | Gancao | MOL004980 | Calmodulin | CALM1 |
| *Licorice* | Gancao | MOL004988 | Calmodulin | CALM1 |
| *Licorice* | Gancao | MOL004989 | Calmodulin | CALM1 |
| *Licorice* | Gancao | MOL004991 | Calmodulin | CALM1 |
| *Licorice* | Gancao | MOL004993 | Calmodulin | CALM1 |
| *Licorice* | Gancao | MOL005000 | Calmodulin | CALM1 |
| *Licorice* | Gancao | MOL005001 | Calmodulin | CALM1 |
| *Licorice* | Gancao | MOL005003 | Calmodulin | CALM1 |
| *Licorice* | Gancao | MOL005007 | Calmodulin | CALM1 |
| *Licorice* | Gancao | MOL005008 | Calmodulin | CALM1 |
| *Licorice* | Gancao | MOL005012 | Calmodulin | CALM1 |
| *Licorice* | Gancao | MOL005016 | Calmodulin | CALM1 |
| *Licorice* | Gancao | MOL005018 | Calmodulin | CALM1 |
| *Licorice* | Gancao | MOL005020 | Calmodulin | CALM1 |
| *Licorice* | Gancao | MOL000422 | Caspase-3 | CASP3 |
| *Licorice* | Gancao | MOL004328 | Caspase-3 | CASP3 |
| *Licorice* | Gancao | MOL000098 | Caspase-3 | CASP3 |
| *Licorice* | Gancao | MOL000098 | Caspase-8 | CASP8 |
| *Licorice* | Gancao | MOL000098 | Caspase-9 | CASP9 |
| *Licorice* | Gancao | MOL004328 | Catalase | CAT |
| *Licorice* | Gancao | MOL000098 | Caveolin-1 | CAV1 |
| *Licorice* | Gancao | MOL000098 | C-C motif chemokine 2 | CCL2 |
| *Licorice* | Gancao | MOL002311 | Cyclin-A2 | CCNA2 |
| *Licorice* | Gancao | MOL002565 | Cyclin-A2 | CCNA2 |
| *Licorice* | Gancao | MOL000354 | Cyclin-A2 | CCNA2 |
| *Licorice* | Gancao | MOL003656 | Cyclin-A2 | CCNA2 |
| *Licorice* | Gancao | MOL003896 | Cyclin-A2 | CCNA2 |
| *Licorice* | Gancao | MOL000392 | Cyclin-A2 | CCNA2 |
| *Licorice* | Gancao | MOL000417 | Cyclin-A2 | CCNA2 |
| *Licorice* | Gancao | MOL004808 | Cyclin-A2 | CCNA2 |
| *Licorice* | Gancao | MOL004810 | Cyclin-A2 | CCNA2 |
| *Licorice* | Gancao | MOL004811 | Cyclin-A2 | CCNA2 |
| *Licorice* | Gancao | MOL004814 | Cyclin-A2 | CCNA2 |
| *Licorice* | Gancao | MOL004815 | Cyclin-A2 | CCNA2 |
| *Licorice* | Gancao | MOL004820 | Cyclin-A2 | CCNA2 |
| *Licorice* | Gancao | MOL004824 | Cyclin-A2 | CCNA2 |
| *Licorice* | Gancao | MOL004828 | Cyclin-A2 | CCNA2 |
| *Licorice* | Gancao | MOL004833 | Cyclin-A2 | CCNA2 |
| *Licorice* | Gancao | MOL004835 | Cyclin-A2 | CCNA2 |
| *Licorice* | Gancao | MOL004841 | Cyclin-A2 | CCNA2 |
| *Licorice* | Gancao | MOL004848 | Cyclin-A2 | CCNA2 |
| *Licorice* | Gancao | MOL004856 | Cyclin-A2 | CCNA2 |
| *Licorice* | Gancao | MOL004857 | Cyclin-A2 | CCNA2 |
| *Licorice* | Gancao | MOL004863 | Cyclin-A2 | CCNA2 |
| *Licorice* | Gancao | MOL004864 | Cyclin-A2 | CCNA2 |
| *Licorice* | Gancao | MOL004866 | Cyclin-A2 | CCNA2 |
| *Licorice* | Gancao | MOL004882 | Cyclin-A2 | CCNA2 |
| *Licorice* | Gancao | MOL004883 | Cyclin-A2 | CCNA2 |
| *Licorice* | Gancao | MOL004884 | Cyclin-A2 | CCNA2 |
| *Licorice* | Gancao | MOL004885 | Cyclin-A2 | CCNA2 |
| *Licorice* | Gancao | MOL004891 | Cyclin-A2 | CCNA2 |
| *Licorice* | Gancao | MOL004898 | Cyclin-A2 | CCNA2 |
| *Licorice* | Gancao | MOL004904 | Cyclin-A2 | CCNA2 |
| *Licorice* | Gancao | MOL004907 | Cyclin-A2 | CCNA2 |
| *Licorice* | Gancao | MOL004908 | Cyclin-A2 | CCNA2 |
| *Licorice* | Gancao | MOL004912 | Cyclin-A2 | CCNA2 |
| *Licorice* | Gancao | MOL004913 | Cyclin-A2 | CCNA2 |
| *Licorice* | Gancao | MOL004915 | Cyclin-A2 | CCNA2 |
| *Licorice* | Gancao | MOL004949 | Cyclin-A2 | CCNA2 |
| *Licorice* | Gancao | MOL004957 | Cyclin-A2 | CCNA2 |
| *Licorice* | Gancao | MOL004959 | Cyclin-A2 | CCNA2 |
| *Licorice* | Gancao | MOL004966 | Cyclin-A2 | CCNA2 |
| *Licorice* | Gancao | MOL000497 | Cyclin-A2 | CCNA2 |
| *Licorice* | Gancao | MOL004974 | Cyclin-A2 | CCNA2 |
| *Licorice* | Gancao | MOL004978 | Cyclin-A2 | CCNA2 |
| *Licorice* | Gancao | MOL000500 | Cyclin-A2 | CCNA2 |
| *Licorice* | Gancao | MOL000500 | Calmodulin | CCNA2 |
| *Licorice* | Gancao | MOL005000 | Cyclin-A2 | CCNA2 |
| *Licorice* | Gancao | MOL005001 | Cyclin-A2 | CCNA2 |
| *Licorice* | Gancao | MOL005003 | Cyclin-A2 | CCNA2 |
| *Licorice* | Gancao | MOL005007 | Cyclin-A2 | CCNA2 |
| *Licorice* | Gancao | MOL005008 | Cyclin-A2 | CCNA2 |
| *Licorice* | Gancao | MOL005012 | Cyclin-A2 | CCNA2 |
| *Licorice* | Gancao | MOL005016 | Cyclin-A2 | CCNA2 |
| *Licorice* | Gancao | MOL005017 | Cyclin-A2 | CCNA2 |
| *Licorice* | Gancao | MOL005020 | Cyclin-A2 | CCNA2 |
| *Licorice* | Gancao | MOL000098 | G2/mitotic-specific cyclin-B1 | CCNB1 |
| *Licorice* | Gancao | MOL000497 | G1/S-specific cyclin-D1 | CCND1 |
| *Licorice* | Gancao | MOL000098 | G1/S-specific cyclin-D1 | CCND1 |
| *Licorice* | Gancao | MOL000098 | CD40 ligand | CD40LG |
| *Licorice* | Gancao | MOL000422 | Cell division control protein 2 homolog | CDK1 |
| *Licorice* | Gancao | MOL000098 | Cell division control protein 2 homolog | CDK1 |
| *Licorice* | Gancao | MOL000239 | Cell division protein kinase 2 | CDK2 |
| *Licorice* | Gancao | MOL002565 | Cell division protein kinase 2 | CDK2 |
| *Licorice* | Gancao | MOL000354 | Cell division protein kinase 2 | CDK2 |
| *Licorice* | Gancao | MOL003656 | Cell division protein kinase 2 | CDK2 |
| *Licorice* | Gancao | MOL003896 | Cell division protein kinase 2 | CDK2 |
| *Licorice* | Gancao | MOL000392 | Cell division protein kinase 2 | CDK2 |
| *Licorice* | Gancao | MOL000417 | Cell division protein kinase 2 | CDK2 |
| *Licorice* | Gancao | MOL004808 | Cell division protein kinase 2 | CDK2 |
| *Licorice* | Gancao | MOL004810 | Cell division protein kinase 2 | CDK2 |
| *Licorice* | Gancao | MOL004811 | Cell division protein kinase 2 | CDK2 |
| *Licorice* | Gancao | MOL004814 | Cell division protein kinase 2 | CDK2 |
| *Licorice* | Gancao | MOL004815 | Cell division protein kinase 2 | CDK2 |
| *Licorice* | Gancao | MOL004820 | Cell division protein kinase 2 | CDK2 |
| *Licorice* | Gancao | MOL004824 | Cell division protein kinase 2 | CDK2 |
| *Licorice* | Gancao | MOL004827 | Cell division protein kinase 2 | CDK2 |
| *Licorice* | Gancao | MOL004828 | Cell division protein kinase 2 | CDK2 |
| *Licorice* | Gancao | MOL004833 | Cell division protein kinase 2 | CDK2 |
| *Licorice* | Gancao | MOL004835 | Cell division protein kinase 2 | CDK2 |
| *Licorice* | Gancao | MOL004841 | Cell division protein kinase 2 | CDK2 |
| *Licorice* | Gancao | MOL004848 | Cell division protein kinase 2 | CDK2 |
| *Licorice* | Gancao | MOL004849 | Cell division protein kinase 2 | CDK2 |
| *Licorice* | Gancao | MOL004863 | Cell division protein kinase 2 | CDK2 |
| *Licorice* | Gancao | MOL004864 | Cell division protein kinase 2 | CDK2 |
| *Licorice* | Gancao | MOL004866 | Cell division protein kinase 2 | CDK2 |
| *Licorice* | Gancao | MOL004882 | Cell division protein kinase 2 | CDK2 |
| *Licorice* | Gancao | MOL004883 | Cell division protein kinase 2 | CDK2 |
| *Licorice* | Gancao | MOL004884 | Cell division protein kinase 2 | CDK2 |
| *Licorice* | Gancao | MOL004885 | Cell division protein kinase 2 | CDK2 |
| *Licorice* | Gancao | MOL004891 | Cell division protein kinase 2 | CDK2 |
| *Licorice* | Gancao | MOL004898 | Cell division protein kinase 2 | CDK2 |
| *Licorice* | Gancao | MOL004904 | Cell division protein kinase 2 | CDK2 |
| *Licorice* | Gancao | MOL004907 | Cell division protein kinase 2 | CDK2 |
| *Licorice* | Gancao | MOL004908 | Cell division protein kinase 2 | CDK2 |
| *Licorice* | Gancao | MOL004911 | Cell division protein kinase 2 | CDK2 |
| *Licorice* | Gancao | MOL004912 | Cell division protein kinase 2 | CDK2 |
| *Licorice* | Gancao | MOL004913 | Cell division protein kinase 2 | CDK2 |
| *Licorice* | Gancao | MOL004914 | Cell division protein kinase 2 | CDK2 |
| *Licorice* | Gancao | MOL004915 | Cell division protein kinase 2 | CDK2 |
| *Licorice* | Gancao | MOL004949 | Cell division protein kinase 2 | CDK2 |
| *Licorice* | Gancao | MOL004957 | Cell division protein kinase 2 | CDK2 |
| *Licorice* | Gancao | MOL004959 | Cell division protein kinase 2 | CDK2 |
| *Licorice* | Gancao | MOL004961 | Cell division protein kinase 2 | CDK2 |
| *Licorice* | Gancao | MOL004966 | Cell division protein kinase 2 | CDK2 |
| *Licorice* | Gancao | MOL000497 | Cell division protein kinase 2 | CDK2 |
| *Licorice* | Gancao | MOL004974 | Cell division protein kinase 2 | CDK2 |
| *Licorice* | Gancao | MOL004978 | Cell division protein kinase 2 | CDK2 |
| *Licorice* | Gancao | MOL004990 | Cell division protein kinase 2 | CDK2 |
| *Licorice* | Gancao | MOL004991 | Cell division protein kinase 2 | CDK2 |
| *Licorice* | Gancao | MOL000500 | Cell division protein kinase 2 | CDK2 |
| *Licorice* | Gancao | MOL005003 | Cell division protein kinase 2 | CDK2 |
| *Licorice* | Gancao | MOL005007 | Cell division protein kinase 2 | CDK2 |
| *Licorice* | Gancao | MOL005008 | Cell division protein kinase 2 | CDK2 |
| *Licorice* | Gancao | MOL005012 | Cell division protein kinase 2 | CDK2 |
| *Licorice* | Gancao | MOL005016 | Cell division protein kinase 2 | CDK2 |
| *Licorice* | Gancao | MOL005017 | Cell division protein kinase 2 | CDK2 |
| *Licorice* | Gancao | MOL005020 | Cell division protein kinase 2 | CDK2 |
| *Licorice* | Gancao | MOL000497 | Cell division protein kinase 4 | CDK4 |
| *Licorice* | Gancao | MOL000098 | Cyclin-dependent kinase inhibitor 1 | CDKN1A |
| *Licorice* | Gancao | MOL000098 | Cyclin-dependent kinase inhibitor 2A, isoforms 1/2/3 | CDKN2A |
| *Licorice* | Gancao | MOL004328 | Liver carboxylesterase 1 | CES1 |
| *Licorice* | Gancao | MOL002311 | Serine/threonine-protein kinase Chk1 | CHEK1 |
| *Licorice* | Gancao | MOL000239 | Serine/threonine-protein kinase Chk1 | CHEK1 |
| *Licorice* | Gancao | MOL000354 | Serine/threonine-protein kinase Chk1 | CHEK1 |
| *Licorice* | Gancao | MOL003656 | Serine/threonine-protein kinase Chk1 | CHEK1 |
| *Licorice* | Gancao | MOL003896 | Serine/threonine-protein kinase Chk1 | CHEK1 |
| *Licorice* | Gancao | MOL000392 | Serine/threonine-protein kinase Chk1 | CHEK1 |
| *Licorice* | Gancao | MOL000417 | Serine/threonine-protein kinase Chk1 | CHEK1 |
| *Licorice* | Gancao | MOL004811 | Serine/threonine-protein kinase Chk1 | CHEK1 |
| *Licorice* | Gancao | MOL004814 | Serine/threonine-protein kinase Chk1 | CHEK1 |
| *Licorice* | Gancao | MOL004815 | Serine/threonine-protein kinase Chk1 | CHEK1 |
| *Licorice* | Gancao | MOL004820 | Serine/threonine-protein kinase Chk1 | CHEK1 |
| *Licorice* | Gancao | MOL004824 | Serine/threonine-protein kinase Chk1 | CHEK1 |
| *Licorice* | Gancao | MOL004827 | Serine/threonine-protein kinase Chk1 | CHEK1 |
| *Licorice* | Gancao | MOL004828 | Serine/threonine-protein kinase Chk1 | CHEK1 |
| *Licorice* | Gancao | MOL004833 | Serine/threonine-protein kinase Chk1 | CHEK1 |
| *Licorice* | Gancao | MOL004835 | Serine/threonine-protein kinase Chk1 | CHEK1 |
| *Licorice* | Gancao | MOL004841 | Serine/threonine-protein kinase Chk1 | CHEK1 |
| *Licorice* | Gancao | MOL004849 | Serine/threonine-protein kinase Chk1 | CHEK1 |
| *Licorice* | Gancao | MOL004855 | Serine/threonine-protein kinase Chk1 | CHEK1 |
| *Licorice* | Gancao | MOL004856 | Serine/threonine-protein kinase Chk1 | CHEK1 |
| *Licorice* | Gancao | MOL004857 | Serine/threonine-protein kinase Chk1 | CHEK1 |
| *Licorice* | Gancao | MOL004863 | Serine/threonine-protein kinase Chk1 | CHEK1 |
| *Licorice* | Gancao | MOL004864 | Serine/threonine-protein kinase Chk1 | CHEK1 |
| *Licorice* | Gancao | MOL004866 | Serine/threonine-protein kinase Chk1 | CHEK1 |
| *Licorice* | Gancao | MOL004879 | Serine/threonine-protein kinase Chk1 | CHEK1 |
| *Licorice* | Gancao | MOL004883 | Serine/threonine-protein kinase Chk1 | CHEK1 |
| *Licorice* | Gancao | MOL004884 | Serine/threonine-protein kinase Chk1 | CHEK1 |
| *Licorice* | Gancao | MOL004907 | Serine/threonine-protein kinase Chk1 | CHEK1 |
| *Licorice* | Gancao | MOL004908 | Serine/threonine-protein kinase Chk1 | CHEK1 |
| *Licorice* | Gancao | MOL004912 | Serine/threonine-protein kinase Chk1 | CHEK1 |
| *Licorice* | Gancao | MOL004913 | Serine/threonine-protein kinase Chk1 | CHEK1 |
| *Licorice* | Gancao | MOL004914 | Serine/threonine-protein kinase Chk1 | CHEK1 |
| *Licorice* | Gancao | MOL004915 | Serine/threonine-protein kinase Chk1 | CHEK1 |
| *Licorice* | Gancao | MOL004957 | Serine/threonine-protein kinase Chk1 | CHEK1 |
| *Licorice* | Gancao | MOL004966 | Serine/threonine-protein kinase Chk1 | CHEK1 |
| *Licorice* | Gancao | MOL000497 | Serine/threonine-protein kinase Chk1 | CHEK1 |
| *Licorice* | Gancao | MOL004974 | Serine/threonine-protein kinase Chk1 | CHEK1 |
| *Licorice* | Gancao | MOL004978 | Serine/threonine-protein kinase Chk1 | CHEK1 |
| *Licorice* | Gancao | MOL004990 | Serine/threonine-protein kinase Chk1 | CHEK1 |
| *Licorice* | Gancao | MOL004991 | Serine/threonine-protein kinase Chk1 | CHEK1 |
| *Licorice* | Gancao | MOL000500 | Serine/threonine-protein kinase Chk1 | CHEK1 |
| *Licorice* | Gancao | MOL005000 | Serine/threonine-protein kinase Chk1 | CHEK1 |
| *Licorice* | Gancao | MOL005012 | Serine/threonine-protein kinase Chk1 | CHEK1 |
| *Licorice* | Gancao | MOL005016 | Serine/threonine-protein kinase Chk1 | CHEK1 |
| *Licorice* | Gancao | MOL005017 | Serine/threonine-protein kinase Chk1 | CHEK1 |
| *Licorice* | Gancao | MOL005020 | Serine/threonine-protein kinase Chk1 | CHEK1 |
| *Licorice* | Gancao | MOL000098 | Serine/threonine-protein kinase Chk2 | CHEK2 |
| *Licorice* | Gancao | MOL001484 | Muscarinic acetylcholine receptor M1 | CHRM1 |
| *Licorice* | Gancao | MOL002565 | Muscarinic acetylcholine receptor M1 | CHRM1 |
| *Licorice* | Gancao | MOL003896 | Muscarinic acetylcholine receptor M1 | CHRM1 |
| *Licorice* | Gancao | MOL000392 | Muscarinic acetylcholine receptor M1 | CHRM1 |
| *Licorice* | Gancao | MOL000422 | Muscarinic acetylcholine receptor M1 | CHRM1 |
| *Licorice* | Gancao | MOL004833 | Muscarinic acetylcholine receptor M1 | CHRM1 |
| *Licorice* | Gancao | MOL004835 | Muscarinic acetylcholine receptor M1 | CHRM1 |
| *Licorice* | Gancao | MOL004891 | Muscarinic acetylcholine receptor M1 | CHRM1 |
| *Licorice* | Gancao | MOL004908 | Muscarinic acetylcholine receptor M1 | CHRM1 |
| *Licorice* | Gancao | MOL004957 | Muscarinic acetylcholine receptor M1 | CHRM1 |
| *Licorice* | Gancao | MOL000497 | Muscarinic acetylcholine receptor M1 | CHRM1 |
| *Licorice* | Gancao | MOL004978 | Muscarinic acetylcholine receptor M1 | CHRM1 |
| *Licorice* | Gancao | MOL000500 | Muscarinic acetylcholine receptor M1 | CHRM1 |
| *Licorice* | Gancao | MOL005003 | Muscarinic acetylcholine receptor M1 | CHRM1 |
| *Licorice* | Gancao | MOL002565 | Muscarinic acetylcholine receptor M2 | CHRM2 |
| *Licorice* | Gancao | MOL000422 | Muscarinic acetylcholine receptor M2 | CHRM2 |
| *Licorice* | Gancao | MOL001484 | Muscarinic acetylcholine receptor M3 | CHRM3 |
| *Licorice* | Gancao | MOL002565 | Muscarinic acetylcholine receptor M3 | CHRM3 |
| *Licorice* | Gancao | MOL003896 | Muscarinic acetylcholine receptor M3 | CHRM3 |
| *Licorice* | Gancao | MOL004891 | Muscarinic acetylcholine receptor M3 | CHRM3 |
| *Licorice* | Gancao | MOL004978 | Muscarinic acetylcholine receptor M3 | CHRM3 |
| *Licorice* | Gancao | MOL005003 | Muscarinic acetylcholine receptor M3 | CHRM3 |
| *Licorice* | Gancao | MOL002565 | Muscarinic acetylcholine receptor M4 | CHRM4 |
| *Licorice* | Gancao | MOL000500 | Muscarinic acetylcholine receptor M4 | CHRM4 |
| *Licorice* | Gancao | MOL002565 | Muscarinic acetylcholine receptor M5 | CHRM5 |
| *Licorice* | Gancao | MOL003896 | Muscarinic acetylcholine receptor M5 | CHRM5 |
| *Licorice* | Gancao | MOL005003 | Muscarinic acetylcholine receptor M5 | CHRM5 |
| *Licorice* | Gancao | MOL002565 | Neuronal acetylcholine receptor protein, alpha-7 chain | CHRNA7 |
| *Licorice* | Gancao | MOL003896 | Neuronal acetylcholine receptor protein, alpha-7 chain | CHRNA7 |
| *Licorice* | Gancao | MOL004891 | Neuronal acetylcholine receptor protein, alpha-7 chain | CHRNA7 |
| *Licorice* | Gancao | MOL000098 | Inhibitor of nuclear factor kappa-B kinase subunit alpha | CHUK |
| *Licorice* | Gancao | MOL000098 | Claudin-4 | CLDN4 |
| *Licorice* | Gancao | MOL000098 | Collagen alpha-1(I) chain | COL1A1 |
| *Licorice* | Gancao | MOL000098 | Collagen alpha-1(III) chain | COL3A1 |
| *Licorice* | Gancao | MOL000098 | C-reactive protein | CRP |
| *Licorice* | Gancao | MOL000098 | Cathepsin D | CTSD |
| *Licorice* | Gancao | MOL000098 | C-X-C motif chemokine 10 | CXCL10 |
| *Licorice* | Gancao | MOL000098 | C-X-C motif chemokine 11 | CXCL11 |
| *Licorice* | Gancao | MOL000098 | C-X-C motif chemokine 2 | CXCL2 |
| *Licorice* | Gancao | MOL000098 | Interleukin-8 | CXCL8 |
| *Licorice* | Gancao | MOL004328 | Cytochrome P450 19A1 | CYP19A1 |
| *Licorice* | Gancao | MOL000422 | Cytochrome P450 1A1 | CYP1A1 |
| *Licorice* | Gancao | MOL000098 | Cytochrome P450 1A1 | CYP1A1 |
| *Licorice* | Gancao | MOL000422 | Cytochrome P450 1A2 | CYP1A2 |
| *Licorice* | Gancao | MOL000098 | Cytochrome P450 1A2 | CYP1A2 |
| *Licorice* | Gancao | MOL000422 | Cytochrome P450 1B1 | CYP1B1 |
| *Licorice* | Gancao | MOL000098 | Cytochrome P450 1B1 | CYP1B1 |
| *Licorice* | Gancao | MOL000422 | Cytochrome P450 3A4 | CYP3A4 |
| *Licorice* | Gancao | MOL000098 | Cytochrome P450 3A4 | CYP3A4 |
| *Licorice* | Gancao | MOL000098 | DDB1- and CUL4-associated factor 5 | DCAF5 |
| *Licorice* | Gancao | MOL000422 | Type I iodothyronine deiodinase | DIO1 |
| *Licorice* | Gancao | MOL000098 | Type I iodothyronine deiodinase | DIO1 |
| *Licorice* | Gancao | MOL000239 | Dipeptidyl peptidase IV | DPP4 |
| *Licorice* | Gancao | MOL002565 | Dipeptidyl peptidase IV | DPP4 |
| *Licorice* | Gancao | MOL000354 | Dipeptidyl peptidase IV | DPP4 |
| *Licorice* | Gancao | MOL003656 | Dipeptidyl peptidase IV | DPP4 |
| *Licorice* | Gancao | MOL003896 | Dipeptidyl peptidase IV | DPP4 |
| *Licorice* | Gancao | MOL000392 | Dipeptidyl peptidase IV | DPP4 |
| *Licorice* | Gancao | MOL000417 | Dipeptidyl peptidase IV | DPP4 |
| *Licorice* | Gancao | MOL000422 | Dipeptidyl peptidase IV | DPP4 |
| *Licorice* | Gancao | MOL004808 | Dipeptidyl peptidase IV | DPP4 |
| *Licorice* | Gancao | MOL004811 | Dipeptidyl peptidase IV | DPP4 |
| *Licorice* | Gancao | MOL004824 | Dipeptidyl peptidase IV | DPP4 |
| *Licorice* | Gancao | MOL004828 | Dipeptidyl peptidase IV | DPP4 |
| *Licorice* | Gancao | MOL004849 | Dipeptidyl peptidase IV | DPP4 |
| *Licorice* | Gancao | MOL004856 | Dipeptidyl peptidase IV | DPP4 |
| *Licorice* | Gancao | MOL004857 | Dipeptidyl peptidase IV | DPP4 |
| *Licorice* | Gancao | MOL004864 | Dipeptidyl peptidase IV | DPP4 |
| *Licorice* | Gancao | MOL004866 | Dipeptidyl peptidase IV | DPP4 |
| *Licorice* | Gancao | MOL004879 | Dipeptidyl peptidase IV | DPP4 |
| *Licorice* | Gancao | MOL004883 | Dipeptidyl peptidase IV | DPP4 |
| *Licorice* | Gancao | MOL004907 | Dipeptidyl peptidase IV | DPP4 |
| *Licorice* | Gancao | MOL004912 | Dipeptidyl peptidase IV | DPP4 |
| *Licorice* | Gancao | MOL004915 | Dipeptidyl peptidase IV | DPP4 |
| *Licorice* | Gancao | MOL004948 | Dipeptidyl peptidase IV | DPP4 |
| *Licorice* | Gancao | MOL004957 | Dipeptidyl peptidase IV | DPP4 |
| *Licorice* | Gancao | MOL004961 | Dipeptidyl peptidase IV | DPP4 |
| *Licorice* | Gancao | MOL004980 | Dipeptidyl peptidase IV | DPP4 |
| *Licorice* | Gancao | MOL004990 | Dipeptidyl peptidase IV | DPP4 |
| *Licorice* | Gancao | MOL004991 | Dipeptidyl peptidase IV | DPP4 |
| *Licorice* | Gancao | MOL000500 | Dipeptidyl peptidase IV | DPP4 |
| *Licorice* | Gancao | MOL005000 | Dipeptidyl peptidase IV | DPP4 |
| *Licorice* | Gancao | MOL005008 | Dipeptidyl peptidase IV | DPP4 |
| *Licorice* | Gancao | MOL005012 | Dipeptidyl peptidase IV | DPP4 |
| *Licorice* | Gancao | MOL005016 | Dipeptidyl peptidase IV | DPP4 |
| *Licorice* | Gancao | MOL000098 | Dipeptidyl peptidase IV | DPP4 |
| *Licorice* | Gancao | MOL002565 | Dopamine D1 receptor | DRD1 |
| *Licorice* | Gancao | MOL003896 | Dopamine D1 receptor | DRD1 |
| *Licorice* | Gancao | MOL000098 | Dual oxidase 2 | DUOX2 |
| *Licorice* | Gancao | MOL000098 | Transcription factor E2F1 | E2F1 |
| *Licorice* | Gancao | MOL000098 | Transcription factor E2F2 | E2F2 |
| *Licorice* | Gancao | MOL000098 | Pro-epidermal growth factor | EGF |
| *Licorice* | Gancao | MOL000098 | Epidermal growth factor receptor | EGFR |
| *Licorice* | Gancao | MOL000497 | Eukaryotic translation initiation factor 6 | EIF6 |
| *Licorice* | Gancao | MOL000098 | Eukaryotic translation initiation factor 6 | EIF6 |
| *Licorice* | Gancao | MOL000098 | ETS domain-containing protein Elk-1 | ELK1 |
| *Licorice* | Gancao | MOL000098 | Receptor tyrosine-protein kinase erbB-2 | ERBB2 |
| *Licorice* | Gancao | MOL000098 | Receptor tyrosine-protein kinase erbB-3 | ERBB3 |
| *Licorice* | Gancao | MOL001792 | Estrogen receptor | ESR1 |
| *Licorice* | Gancao | MOL002311 | Estrogen receptor | ESR1 |
| *Licorice* | Gancao | MOL002565 | Estrogen receptor | ESR1 |
| *Licorice* | Gancao | MOL000354 | Estrogen receptor | ESR1 |
| *Licorice* | Gancao | MOL003656 | Estrogen receptor | ESR1 |
| *Licorice* | Gancao | MOL003896 | Estrogen receptor | ESR1 |
| *Licorice* | Gancao | MOL000392 | Estrogen receptor | ESR1 |
| *Licorice* | Gancao | MOL000417 | Estrogen receptor | ESR1 |
| *Licorice* | Gancao | MOL004328 | Estrogen receptor | ESR1 |
| *Licorice* | Gancao | MOL004805 | Estrogen receptor | ESR1 |
| *Licorice* | Gancao | MOL004806 | Estrogen receptor | ESR1 |
| *Licorice* | Gancao | MOL004808 | Estrogen receptor | ESR1 |
| *Licorice* | Gancao | MOL004810 | Estrogen receptor | ESR1 |
| *Licorice* | Gancao | MOL004811 | Estrogen receptor | ESR1 |
| *Licorice* | Gancao | MOL004814 | Estrogen receptor | ESR1 |
| *Licorice* | Gancao | MOL004815 | Estrogen receptor | ESR1 |
| *Licorice* | Gancao | MOL004820 | Estrogen receptor | ESR1 |
| *Licorice* | Gancao | MOL004824 | Estrogen receptor | ESR1 |
| *Licorice* | Gancao | MOL004827 | Estrogen receptor | ESR1 |
| *Licorice* | Gancao | MOL004828 | Estrogen receptor | ESR1 |
| *Licorice* | Gancao | MOL004829 | Estrogen receptor | ESR1 |
| *Licorice* | Gancao | MOL004833 | Estrogen receptor | ESR1 |
| *Licorice* | Gancao | MOL004835 | Estrogen receptor | ESR1 |
| *Licorice* | Gancao | MOL004838 | Estrogen receptor | ESR1 |
| *Licorice* | Gancao | MOL004841 | Estrogen receptor | ESR1 |
| *Licorice* | Gancao | MOL004848 | Estrogen receptor | ESR1 |
| *Licorice* | Gancao | MOL004849 | Estrogen receptor | ESR1 |
| *Licorice* | Gancao | MOL004855 | Estrogen receptor | ESR1 |
| *Licorice* | Gancao | MOL004856 | Estrogen receptor | ESR1 |
| *Licorice* | Gancao | MOL004857 | Estrogen receptor | ESR1 |
| *Licorice* | Gancao | MOL004863 | Estrogen receptor | ESR1 |
| *Licorice* | Gancao | MOL004864 | Estrogen receptor | ESR1 |
| *Licorice* | Gancao | MOL004879 | Estrogen receptor | ESR1 |
| *Licorice* | Gancao | MOL004882 | Estrogen receptor | ESR1 |
| *Licorice* | Gancao | MOL004883 | Estrogen receptor | ESR1 |
| *Licorice* | Gancao | MOL004884 | Estrogen receptor | ESR1 |
| *Licorice* | Gancao | MOL004885 | Estrogen receptor | ESR1 |
| *Licorice* | Gancao | MOL004891 | Estrogen receptor | ESR1 |
| *Licorice* | Gancao | MOL004898 | Estrogen receptor | ESR1 |
| *Licorice* | Gancao | MOL004904 | Estrogen receptor | ESR1 |
| *Licorice* | Gancao | MOL004907 | Estrogen receptor | ESR1 |
| *Licorice* | Gancao | MOL004908 | Estrogen receptor | ESR1 |
| *Licorice* | Gancao | MOL004910 | Estrogen receptor | ESR1 |
| *Licorice* | Gancao | MOL004911 | Estrogen receptor | ESR1 |
| *Licorice* | Gancao | MOL004912 | Estrogen receptor | ESR1 |
| *Licorice* | Gancao | MOL004913 | Estrogen receptor | ESR1 |
| *Licorice* | Gancao | MOL004914 | Estrogen receptor | ESR1 |
| *Licorice* | Gancao | MOL004915 | Estrogen receptor | ESR1 |
| *Licorice* | Gancao | MOL004935 | Estrogen receptor | ESR1 |
| *Licorice* | Gancao | MOL004941 | Estrogen receptor | ESR1 |
| *Licorice* | Gancao | MOL004945 | Estrogen receptor | ESR1 |
| *Licorice* | Gancao | MOL004948 | Estrogen receptor | ESR1 |
| *Licorice* | Gancao | MOL004949 | Estrogen receptor | ESR1 |
| *Licorice* | Gancao | MOL004957 | Estrogen receptor | ESR1 |
| *Licorice* | Gancao | MOL004959 | Estrogen receptor | ESR1 |
| *Licorice* | Gancao | MOL004961 | Estrogen receptor | ESR1 |
| *Licorice* | Gancao | MOL004966 | Estrogen receptor | ESR1 |
| *Licorice* | Gancao | MOL000497 | Estrogen receptor | ESR1 |
| *Licorice* | Gancao | MOL004974 | Estrogen receptor | ESR1 |
| *Licorice* | Gancao | MOL004978 | Estrogen receptor | ESR1 |
| *Licorice* | Gancao | MOL004980 | Estrogen receptor | ESR1 |
| *Licorice* | Gancao | MOL004988 | Estrogen receptor | ESR1 |
| *Licorice* | Gancao | MOL004989 | Estrogen receptor | ESR1 |
| *Licorice* | Gancao | MOL004990 | Estrogen receptor | ESR1 |
| *Licorice* | Gancao | MOL004991 | Estrogen receptor | ESR1 |
| *Licorice* | Gancao | MOL004993 | Estrogen receptor | ESR1 |
| *Licorice* | Gancao | MOL000500 | Estrogen receptor | ESR1 |
| *Licorice* | Gancao | MOL005000 | Estrogen receptor | ESR1 |
| *Licorice* | Gancao | MOL005001 | Estrogen receptor | ESR1 |
| *Licorice* | Gancao | MOL005003 | Estrogen receptor | ESR1 |
| *Licorice* | Gancao | MOL005007 | Estrogen receptor | ESR1 |
| *Licorice* | Gancao | MOL005008 | Estrogen receptor | ESR1 |
| *Licorice* | Gancao | MOL005012 | Estrogen receptor | ESR1 |
| *Licorice* | Gancao | MOL005016 | Estrogen receptor | ESR1 |
| *Licorice* | Gancao | MOL005017 | Estrogen receptor | ESR1 |
| *Licorice* | Gancao | MOL005018 | Estrogen receptor | ESR1 |
| *Licorice* | Gancao | MOL005020 | Estrogen receptor | ESR1 |
| *Licorice* | Gancao | MOL000239 | Estrogen receptor beta | ESR2 |
| *Licorice* | Gancao | MOL002565 | Estrogen receptor beta | ESR2 |
| *Licorice* | Gancao | MOL000354 | Estrogen receptor beta | ESR2 |
| *Licorice* | Gancao | MOL003656 | Estrogen receptor beta | ESR2 |
| *Licorice* | Gancao | MOL003896 | Estrogen receptor beta | ESR2 |
| *Licorice* | Gancao | MOL000392 | Estrogen receptor beta | ESR2 |
| *Licorice* | Gancao | MOL000417 | Estrogen receptor beta | ESR2 |
| *Licorice* | Gancao | MOL004805 | Estrogen receptor beta | ESR2 |
| *Licorice* | Gancao | MOL004806 | Estrogen receptor beta | ESR2 |
| *Licorice* | Gancao | MOL004808 | Estrogen receptor beta | ESR2 |
| *Licorice* | Gancao | MOL004810 | Estrogen receptor beta | ESR2 |
| *Licorice* | Gancao | MOL004811 | Estrogen receptor beta | ESR2 |
| *Licorice* | Gancao | MOL004814 | Estrogen receptor beta | ESR2 |
| *Licorice* | Gancao | MOL004815 | Estrogen receptor beta | ESR2 |
| *Licorice* | Gancao | MOL004820 | Estrogen receptor beta | ESR2 |
| *Licorice* | Gancao | MOL004824 | Estrogen receptor beta | ESR2 |
| *Licorice* | Gancao | MOL004833 | Estrogen receptor beta | ESR2 |
| *Licorice* | Gancao | MOL004835 | Estrogen receptor beta | ESR2 |
| *Licorice* | Gancao | MOL004841 | Estrogen receptor beta | ESR2 |
| *Licorice* | Gancao | MOL004848 | Estrogen receptor beta | ESR2 |
| *Licorice* | Gancao | MOL004849 | Estrogen receptor beta | ESR2 |
| *Licorice* | Gancao | MOL004856 | Estrogen receptor beta | ESR2 |
| *Licorice* | Gancao | MOL004857 | Estrogen receptor beta | ESR2 |
| *Licorice* | Gancao | MOL004864 | Estrogen receptor beta | ESR2 |
| *Licorice* | Gancao | MOL004879 | Estrogen receptor beta | ESR2 |
| *Licorice* | Gancao | MOL004882 | Estrogen receptor beta | ESR2 |
| *Licorice* | Gancao | MOL004884 | Estrogen receptor beta | ESR2 |
| *Licorice* | Gancao | MOL004885 | Estrogen receptor beta | ESR2 |
| *Licorice* | Gancao | MOL004891 | Estrogen receptor beta | ESR2 |
| *Licorice* | Gancao | MOL004907 | Estrogen receptor beta | ESR2 |
| *Licorice* | Gancao | MOL004908 | Estrogen receptor beta | ESR2 |
| *Licorice* | Gancao | MOL004911 | Estrogen receptor beta | ESR2 |
| *Licorice* | Gancao | MOL004912 | Estrogen receptor beta | ESR2 |
| *Licorice* | Gancao | MOL004913 | Estrogen receptor beta | ESR2 |
| *Licorice* | Gancao | MOL004915 | Estrogen receptor beta | ESR2 |
| *Licorice* | Gancao | MOL004945 | Estrogen receptor beta | ESR2 |
| *Licorice* | Gancao | MOL004957 | Estrogen receptor beta | ESR2 |
| *Licorice* | Gancao | MOL004959 | Estrogen receptor beta | ESR2 |
| *Licorice* | Gancao | MOL004961 | Estrogen receptor beta | ESR2 |
| *Licorice* | Gancao | MOL004966 | Estrogen receptor beta | ESR2 |
| *Licorice* | Gancao | MOL000497 | Estrogen receptor beta | ESR2 |
| *Licorice* | Gancao | MOL004974 | Estrogen receptor beta | ESR2 |
| *Licorice* | Gancao | MOL004978 | Estrogen receptor beta | ESR2 |
| *Licorice* | Gancao | MOL004988 | Estrogen receptor beta | ESR2 |
| *Licorice* | Gancao | MOL004990 | Estrogen receptor beta | ESR2 |
| *Licorice* | Gancao | MOL000500 | Estrogen receptor beta | ESR2 |
| *Licorice* | Gancao | MOL005000 | Estrogen receptor beta | ESR2 |
| *Licorice* | Gancao | MOL005003 | Estrogen receptor beta | ESR2 |
| *Licorice* | Gancao | MOL005007 | Estrogen receptor beta | ESR2 |
| *Licorice* | Gancao | MOL005008 | Estrogen receptor beta | ESR2 |
| *Licorice* | Gancao | MOL005012 | Estrogen receptor beta | ESR2 |
| *Licorice* | Gancao | MOL005016 | Estrogen receptor beta | ESR2 |
| *Licorice* | Gancao | MOL005017 | Prostaglandin G/H synthase 2 | ESR2 |
| *Licorice* | Gancao | MOL005018 | Estrogen receptor beta | ESR2 |
| *Licorice* | Gancao | MOL005020 | Estrogen receptor beta | ESR2 |
| *Licorice* | Gancao | MOL003656 | Coagulation factor Xa | F10 |
| *Licorice* | Gancao | MOL004805 | Coagulation factor Xa | F10 |
| *Licorice* | Gancao | MOL004806 | Coagulation factor Xa | F10 |
| *Licorice* | Gancao | MOL004808 | Coagulation factor Xa | F10 |
| *Licorice* | Gancao | MOL004810 | Coagulation factor Xa | F10 |
| *Licorice* | Gancao | MOL004811 | Coagulation factor Xa | F10 |
| *Licorice* | Gancao | MOL004815 | Coagulation factor Xa | F10 |
| *Licorice* | Gancao | MOL004820 | Coagulation factor Xa | F10 |
| *Licorice* | Gancao | MOL004824 | Coagulation factor Xa | F10 |
| *Licorice* | Gancao | MOL004827 | Coagulation factor Xa | F10 |
| *Licorice* | Gancao | MOL004828 | Coagulation factor Xa | F10 |
| *Licorice* | Gancao | MOL004829 | Coagulation factor Xa | F10 |
| *Licorice* | Gancao | MOL004833 | Coagulation factor Xa | F10 |
| *Licorice* | Gancao | MOL004848 | Coagulation factor Xa | F10 |
| *Licorice* | Gancao | MOL004849 | Coagulation factor Xa | F10 |
| *Licorice* | Gancao | MOL004855 | Coagulation factor Xa | F10 |
| *Licorice* | Gancao | MOL004856 | Coagulation factor Xa | F10 |
| *Licorice* | Gancao | MOL004857 | Coagulation factor Xa | F10 |
| *Licorice* | Gancao | MOL004863 | Coagulation factor Xa | F10 |
| *Licorice* | Gancao | MOL004864 | Coagulation factor Xa | F10 |
| *Licorice* | Gancao | MOL004866 | Coagulation factor Xa | F10 |
| *Licorice* | Gancao | MOL004879 | Coagulation factor Xa | F10 |
| *Licorice* | Gancao | MOL004883 | Coagulation factor Xa | F10 |
| *Licorice* | Gancao | MOL004884 | Coagulation factor Xa | F10 |
| *Licorice* | Gancao | MOL004885 | Coagulation factor Xa | F10 |
| *Licorice* | Gancao | MOL004903 | Coagulation factor Xa | F10 |
| *Licorice* | Gancao | MOL004904 | Coagulation factor Xa | F10 |
| *Licorice* | Gancao | MOL004910 | Coagulation factor Xa | F10 |
| *Licorice* | Gancao | MOL004911 | Coagulation factor Xa | F10 |
| *Licorice* | Gancao | MOL004912 | Coagulation factor Xa | F10 |
| *Licorice* | Gancao | MOL004915 | Coagulation factor Xa | F10 |
| *Licorice* | Gancao | MOL004935 | Coagulation factor Xa | F10 |
| *Licorice* | Gancao | MOL004945 | Coagulation factor Xa | F10 |
| *Licorice* | Gancao | MOL004949 | Coagulation factor Xa | F10 |
| *Licorice* | Gancao | MOL004959 | Coagulation factor Xa | F10 |
| *Licorice* | Gancao | MOL004966 | Coagulation factor Xa | F10 |
| *Licorice* | Gancao | MOL000497 | Coagulation factor Xa | F10 |
| *Licorice* | Gancao | MOL004974 | Coagulation factor Xa | F10 |
| *Licorice* | Gancao | MOL004978 | Coagulation factor Xa | F10 |
| *Licorice* | Gancao | MOL004980 | Coagulation factor Xa | F10 |
| *Licorice* | Gancao | MOL004988 | Coagulation factor Xa | F10 |
| *Licorice* | Gancao | MOL004989 | Coagulation factor Xa | F10 |
| *Licorice* | Gancao | MOL004993 | Coagulation factor Xa | F10 |
| *Licorice* | Gancao | MOL005000 | Coagulation factor Xa | F10 |
| *Licorice* | Gancao | MOL005001 | Coagulation factor Xa | F10 |
| *Licorice* | Gancao | MOL005003 | Coagulation factor Xa | F10 |
| *Licorice* | Gancao | MOL005007 | Coagulation factor Xa | F10 |
| *Licorice* | Gancao | MOL005008 | Coagulation factor Xa | F10 |
| *Licorice* | Gancao | MOL005012 | Coagulation factor Xa | F10 |
| *Licorice* | Gancao | MOL005018 | Coagulation factor Xa | F10 |
| *Licorice* | Gancao | MOL005020 | Coagulation factor Xa | F10 |
| *Licorice* | Gancao | MOL000098 | Coagulation factor Xa | F10 |
| *Licorice* | Gancao | MOL002311 | Thrombin | F2 |
| *Licorice* | Gancao | MOL000354 | Thrombin | F2 |
| *Licorice* | Gancao | MOL003656 | Thrombin | F2 |
| *Licorice* | Gancao | MOL003896 | Thrombin | F2 |
| *Licorice* | Gancao | MOL000392 | Thrombin | F2 |
| *Licorice* | Gancao | MOL000422 | Thrombin | F2 |
| *Licorice* | Gancao | MOL004808 | Thrombin | F2 |
| *Licorice* | Gancao | MOL004811 | Thrombin | F2 |
| *Licorice* | Gancao | MOL004824 | Thrombin | F2 |
| *Licorice* | Gancao | MOL004827 | Thrombin | F2 |
| *Licorice* | Gancao | MOL004828 | Thrombin | F2 |
| *Licorice* | Gancao | MOL004849 | Thrombin | F2 |
| *Licorice* | Gancao | MOL004855 | Thrombin | F2 |
| *Licorice* | Gancao | MOL004856 | Thrombin | F2 |
| *Licorice* | Gancao | MOL004857 | Thrombin | F2 |
| *Licorice* | Gancao | MOL004863 | Thrombin | F2 |
| *Licorice* | Gancao | MOL004866 | Thrombin | F2 |
| *Licorice* | Gancao | MOL004879 | Thrombin | F2 |
| *Licorice* | Gancao | MOL004883 | Thrombin | F2 |
| *Licorice* | Gancao | MOL004884 | Thrombin | F2 |
| *Licorice* | Gancao | MOL004904 | Thrombin | F2 |
| *Licorice* | Gancao | MOL004912 | Thrombin | F2 |
| *Licorice* | Gancao | MOL004915 | Thrombin | F2 |
| *Licorice* | Gancao | MOL004949 | Thrombin | F2 |
| *Licorice* | Gancao | MOL004959 | Thrombin | F2 |
| *Licorice* | Gancao | MOL004980 | Thrombin | F2 |
| *Licorice* | Gancao | MOL004991 | Thrombin | F2 |
| *Licorice* | Gancao | MOL005000 | Thrombin | F2 |
| *Licorice* | Gancao | MOL005003 | Thrombin | F2 |
| *Licorice* | Gancao | MOL005012 | Thrombin | F2 |
| *Licorice* | Gancao | MOL005017 | Thrombin | F2 |
| *Licorice* | Gancao | MOL000098 | Thrombin | F2 |
| *Licorice* | Gancao | MOL000098 | Tissue factor | F3 |
| *Licorice* | Gancao | MOL000354 | Coagulation factor VII | F7 |
| *Licorice* | Gancao | MOL000422 | Coagulation factor VII | F7 |
| *Licorice* | Gancao | MOL004808 | Coagulation factor VII | F7 |
| *Licorice* | Gancao | MOL004824 | Coagulation factor VII | F7 |
| *Licorice* | Gancao | MOL004827 | Coagulation factor VII | F7 |
| *Licorice* | Gancao | MOL004828 | Coagulation factor VII | F7 |
| *Licorice* | Gancao | MOL004829 | Coagulation factor VII | F7 |
| *Licorice* | Gancao | MOL004849 | Coagulation factor VII | F7 |
| *Licorice* | Gancao | MOL004857 | Coagulation factor VII | F7 |
| *Licorice* | Gancao | MOL004866 | Coagulation factor VII | F7 |
| *Licorice* | Gancao | MOL004885 | Coagulation factor VII | F7 |
| *Licorice* | Gancao | MOL004903 | Coagulation factor VII | F7 |
| *Licorice* | Gancao | MOL004904 | Coagulation factor VII | F7 |
| *Licorice* | Gancao | MOL004966 | Coagulation factor VII | F7 |
| *Licorice* | Gancao | MOL004974 | Coagulation factor VII | F7 |
| *Licorice* | Gancao | MOL004989 | Coagulation factor VII | F7 |
| *Licorice* | Gancao | MOL004993 | Coagulation factor VII | F7 |
| *Licorice* | Gancao | MOL005007 | Coagulation factor VII | F7 |
| *Licorice* | Gancao | MOL005008 | Coagulation factor VII | F7 |
| *Licorice* | Gancao | MOL000098 | Coagulation factor VII | F7 |
| *Licorice* | Gancao | MOL004328 | Fatty acid synthase | FASN |
| *Licorice* | Gancao | MOL000098 | Proto-oncogene c-Fos | FOS |
| *Licorice* | Gancao | MOL000497 | Fos-related antigen 2 | FOSL2 |
| *Licorice* | Gancao | MOL000354 | Gamma-aminobutyric acid receptor subunit alpha-1 | GABRA1 |
| *Licorice* | Gancao | MOL003896 | Gamma-aminobutyric acid receptor subunit alpha-1 | GABRA1 |
| *Licorice* | Gancao | MOL000422 | Gamma-aminobutyric acid receptor subunit alpha-1 | GABRA1 |
| *Licorice* | Gancao | MOL004941 | Gamma-aminobutyric acid receptor subunit alpha-1 | GABRA1 |
| *Licorice* | Gancao | MOL004991 | Gamma-aminobutyric acid receptor subunit alpha-1 | GABRA1 |
| *Licorice* | Gancao | MOL000098 | Gamma-aminobutyric acid receptor subunit alpha-1 | GABRA1 |
| *Licorice* | Gancao | MOL000422 | Gamma-aminobutyric-acid receptor alpha-2 subunit | GABRA2 |
| *Licorice* | Gancao | MOL000098 | Gap junction alpha-1 protein | GJA1 |
| *Licorice* | Gancao | MOL004328 | Aspartate aminotransferase, cytoplasmic | GOT1 |
| *Licorice* | Gancao | MOL000354 | Glutamate receptor 2 | GRIA2 |
| *Licorice* | Gancao | MOL002311 | Glycogen synthase kinase-3 beta | GSK3B |
| *Licorice* | Gancao | MOL000354 | Glycogen synthase kinase-3 beta | GSK3B |
| *Licorice* | Gancao | MOL003656 | Glycogen synthase kinase-3 beta | GSK3B |
| *Licorice* | Gancao | MOL003896 | Glycogen synthase kinase-3 beta | GSK3B |
| *Licorice* | Gancao | MOL000392 | Glycogen synthase kinase-3 beta | GSK3B |
| *Licorice* | Gancao | MOL000417 | Glycogen synthase kinase-3 beta | GSK3B |
| *Licorice* | Gancao | MOL004805 | Glycogen synthase kinase-3 beta | GSK3B |
| *Licorice* | Gancao | MOL004808 | Glycogen synthase kinase-3 beta | GSK3B |
| *Licorice* | Gancao | MOL004810 | Glycogen synthase kinase-3 beta | GSK3B |
| *Licorice* | Gancao | MOL004811 | Glycogen synthase kinase-3 beta | GSK3B |
| *Licorice* | Gancao | MOL004814 | Glycogen synthase kinase-3 beta | GSK3B |
| *Licorice* | Gancao | MOL004815 | Glycogen synthase kinase-3 beta | GSK3B |
| *Licorice* | Gancao | MOL004820 | Glycogen synthase kinase-3 beta | GSK3B |
| *Licorice* | Gancao | MOL004824 | Glycogen synthase kinase-3 beta | GSK3B |
| *Licorice* | Gancao | MOL004827 | Glycogen synthase kinase-3 beta | GSK3B |
| *Licorice* | Gancao | MOL004828 | Glycogen synthase kinase-3 beta | GSK3B |
| *Licorice* | Gancao | MOL004833 | Glycogen synthase kinase-3 beta | GSK3B |
| *Licorice* | Gancao | MOL004835 | Glycogen synthase kinase-3 beta | GSK3B |
| *Licorice* | Gancao | MOL004841 | Glycogen synthase kinase-3 beta | GSK3B |
| *Licorice* | Gancao | MOL004848 | Glycogen synthase kinase-3 beta | GSK3B |
| *Licorice* | Gancao | MOL004849 | Glycogen synthase kinase-3 beta | GSK3B |
| *Licorice* | Gancao | MOL004856 | Glycogen synthase kinase-3 beta | GSK3B |
| *Licorice* | Gancao | MOL004857 | Glycogen synthase kinase-3 beta | GSK3B |
| *Licorice* | Gancao | MOL004863 | Glycogen synthase kinase-3 beta | GSK3B |
| *Licorice* | Gancao | MOL004864 | Glycogen synthase kinase-3 beta | GSK3B |
| *Licorice* | Gancao | MOL004882 | Glycogen synthase kinase-3 beta | GSK3B |
| *Licorice* | Gancao | MOL004884 | Glycogen synthase kinase-3 beta | GSK3B |
| *Licorice* | Gancao | MOL004885 | Glycogen synthase kinase-3 beta | GSK3B |
| *Licorice* | Gancao | MOL004891 | Glycogen synthase kinase-3 beta | GSK3B |
| *Licorice* | Gancao | MOL004898 | Glycogen synthase kinase-3 beta | GSK3B |
| *Licorice* | Gancao | MOL004907 | Glycogen synthase kinase-3 beta | GSK3B |
| *Licorice* | Gancao | MOL004908 | Glycogen synthase kinase-3 beta | GSK3B |
| *Licorice* | Gancao | MOL004911 | Glycogen synthase kinase-3 beta | GSK3B |
| *Licorice* | Gancao | MOL004912 | Glycogen synthase kinase-3 beta | GSK3B |
| *Licorice* | Gancao | MOL004913 | Glycogen synthase kinase-3 beta | GSK3B |
| *Licorice* | Gancao | MOL004914 | Glycogen synthase kinase-3 beta | GSK3B |
| *Licorice* | Gancao | MOL004915 | Glycogen synthase kinase-3 beta | GSK3B |
| *Licorice* | Gancao | MOL004948 | Glycogen synthase kinase-3 beta | GSK3B |
| *Licorice* | Gancao | MOL004949 | Glycogen synthase kinase-3 beta | GSK3B |
| *Licorice* | Gancao | MOL004957 | Glycogen synthase kinase-3 beta | GSK3B |
| *Licorice* | Gancao | MOL004959 | Glycogen synthase kinase-3 beta | GSK3B |
| *Licorice* | Gancao | MOL004961 | Glycogen synthase kinase-3 beta | GSK3B |
| *Licorice* | Gancao | MOL004966 | Glycogen synthase kinase-3 beta | GSK3B |
| *Licorice* | Gancao | MOL000497 | Glycogen synthase kinase-3 beta | GSK3B |
| *Licorice* | Gancao | MOL004974 | Glycogen synthase kinase-3 beta | GSK3B |
| *Licorice* | Gancao | MOL004978 | Glycogen synthase kinase-3 beta | GSK3B |
| *Licorice* | Gancao | MOL004990 | Glycogen synthase kinase-3 beta | GSK3B |
| *Licorice* | Gancao | MOL004991 | Glycogen synthase kinase-3 beta | GSK3B |
| *Licorice* | Gancao | MOL000500 | Glycogen synthase kinase-3 beta | GSK3B |
| *Licorice* | Gancao | MOL005000 | Glycogen synthase kinase-3 beta | GSK3B |
| *Licorice* | Gancao | MOL005003 | Glycogen synthase kinase-3 beta | GSK3B |
| *Licorice* | Gancao | MOL005007 | Glycogen synthase kinase-3 beta | GSK3B |
| *Licorice* | Gancao | MOL005008 | Glycogen synthase kinase-3 beta | GSK3B |
| *Licorice* | Gancao | MOL005012 | Glycogen synthase kinase-3 beta | GSK3B |
| *Licorice* | Gancao | MOL005016 | Glycogen synthase kinase-3 beta | GSK3B |
| *Licorice* | Gancao | MOL005017 | Glycogen synthase kinase-3 beta | GSK3B |
| *Licorice* | Gancao | MOL004328 | Glutathione reductase, mitochondrial | GSR |
| *Licorice* | Gancao | MOL000422 | Glutathione S-transferase Mu 1 | GSTM1 |
| *Licorice* | Gancao | MOL000098 | Glutathione S-transferase Mu 1 | GSTM1 |
| *Licorice* | Gancao | MOL000422 | Glutathione S-transferase Mu 2 | GSTM2 |
| *Licorice* | Gancao | MOL000098 | Glutathione S-transferase Mu 2 | GSTM2 |
| *Licorice* | Gancao | MOL000422 | Glutathione S-transferase P | GSTP1 |
| *Licorice* | Gancao | MOL004328 | Glutathione S-transferase P | GSTP1 |
| *Licorice* | Gancao | MOL000098 | Glutathione S-transferase P | GSTP1 |
| *Licorice* | Gancao | MOL000422 | Hyaluronan synthase 2 | HAS2 |
| *Licorice* | Gancao | MOL000098 | Hyaluronan synthase 2 | HAS2 |
| *Licorice* | Gancao | MOL000098 | Probable E3 ubiquitin-protein ligase HERC5 | HERC5 |
| *Licorice* | Gancao | MOL000098 | Hypoxia-inducible factor 1-alpha | HIF1A |
| *Licorice* | Gancao | MOL000098 | Hexokinase-2 | HK2 |
| *Licorice* | Gancao | MOL004328 | 3-hydroxy-3-methylglutaryl-coenzyme A reductase | HMGCR |
| *Licorice* | Gancao | MOL000422 | Heme oxygenase 1 | HMOX1 |
| *Licorice* | Gancao | MOL000098 | Heme oxygenase 1 | HMOX1 |
| *Licorice* | Gancao | MOL000392 | 3 beta-hydroxysteroid dehydrogenase/Delta 5-->4-isomerase type 1 | HSD3B1 |
| *Licorice* | Gancao | MOL000392 | 3 beta-hydroxysteroid dehydrogenase/Delta 5-->4-isomerase type 2 | HSD3B2 |
| *Licorice* | Gancao | MOL000098 | Heat shock factor protein 1 | HSF1 |
| *Licorice* | Gancao | MOL001484 | Heat shock protein HSP 90 | HSP90AB1 |
| *Licorice* | Gancao | MOL001792 | Heat shock protein HSP 90 | HSP90AB1 |
| *Licorice* | Gancao | MOL000239 | Heat shock protein HSP 90 | HSP90AB1 |
| *Licorice* | Gancao | MOL002565 | Heat shock protein HSP 90 | HSP90AB1 |
| *Licorice* | Gancao | MOL000354 | Heat shock protein HSP 90 | HSP90AB1 |
| *Licorice* | Gancao | MOL003656 | Heat shock protein HSP 90 | HSP90AB1 |
| *Licorice* | Gancao | MOL003896 | Heat shock protein HSP 90 | HSP90AB1 |
| *Licorice* | Gancao | MOL000392 | Heat shock protein HSP 90 | HSP90AB1 |
| *Licorice* | Gancao | MOL000417 | Heat shock protein HSP 90 | HSP90AB1 |
| *Licorice* | Gancao | MOL000422 | Heat shock protein HSP 90 | HSP90AB1 |
| *Licorice* | Gancao | MOL004328 | Heat shock protein HSP 90 | HSP90AB1 |
| *Licorice* | Gancao | MOL004808 | Heat shock protein HSP 90 | HSP90AB1 |
| *Licorice* | Gancao | MOL004810 | Heat shock protein HSP 90 | HSP90AB1 |
| *Licorice* | Gancao | MOL004811 | Heat shock protein HSP 90 | HSP90AB1 |
| *Licorice* | Gancao | MOL004814 | Heat shock protein HSP 90 | HSP90AB1 |
| *Licorice* | Gancao | MOL004827 | Heat shock protein HSP 90 | HSP90AB1 |
| *Licorice* | Gancao | MOL004828 | Heat shock protein HSP 90 | HSP90AB1 |
| *Licorice* | Gancao | MOL004829 | Heat shock protein HSP 90 | HSP90AB1 |
| *Licorice* | Gancao | MOL004835 | Heat shock protein HSP 90 | HSP90AB1 |
| *Licorice* | Gancao | MOL004838 | Heat shock protein HSP 90 | HSP90AB1 |
| *Licorice* | Gancao | MOL004841 | Heat shock protein HSP 90 | HSP90AB1 |
| *Licorice* | Gancao | MOL004848 | Heat shock protein HSP 90 | HSP90AB1 |
| *Licorice* | Gancao | MOL004849 | Heat shock protein HSP 90 | HSP90AB1 |
| *Licorice* | Gancao | MOL004856 | Heat shock protein HSP 90 | HSP90AB1 |
| *Licorice* | Gancao | MOL004857 | Heat shock protein HSP 90 | HSP90AB1 |
| *Licorice* | Gancao | MOL004863 | Heat shock protein HSP 90 | HSP90AB1 |
| *Licorice* | Gancao | MOL004864 | Heat shock protein HSP 90 | HSP90AB1 |
| *Licorice* | Gancao | MOL004866 | Heat shock protein HSP 90 | HSP90AB1 |
| *Licorice* | Gancao | MOL004882 | Heat shock protein HSP 90 | HSP90AB1 |
| *Licorice* | Gancao | MOL004883 | Heat shock protein HSP 90 | HSP90AB1 |
| *Licorice* | Gancao | MOL004885 | Heat shock protein HSP 90 | HSP90AB1 |
| *Licorice* | Gancao | MOL004898 | Heat shock protein HSP 90 | HSP90AB1 |
| *Licorice* | Gancao | MOL004907 | Heat shock protein HSP 90 | HSP90AB1 |
| *Licorice* | Gancao | MOL004910 | Heat shock protein HSP 90 | HSP90AB1 |
| *Licorice* | Gancao | MOL004911 | Heat shock protein HSP 90 | HSP90AB1 |
| *Licorice* | Gancao | MOL004913 | Heat shock protein HSP 90 | HSP90AB1 |
| *Licorice* | Gancao | MOL004914 | Heat shock protein HSP 90 | HSP90AB1 |
| *Licorice* | Gancao | MOL004915 | Heat shock protein HSP 90 | HSP90AB1 |
| *Licorice* | Gancao | MOL004935 | Heat shock protein HSP 90 | HSP90AB1 |
| *Licorice* | Gancao | MOL004941 | Heat shock protein HSP 90 | HSP90AB1 |
| *Licorice* | Gancao | MOL004945 | Heat shock protein HSP 90 | HSP90AB1 |
| *Licorice* | Gancao | MOL004949 | Heat shock protein HSP 90 | HSP90AB1 |
| *Licorice* | Gancao | MOL004959 | Heat shock protein HSP 90 | HSP90AB1 |
| *Licorice* | Gancao | MOL004961 | Heat shock protein HSP 90 | HSP90AB1 |
| *Licorice* | Gancao | MOL004966 | Heat shock protein HSP 90 | HSP90AB1 |
| *Licorice* | Gancao | MOL000497 | Heat shock protein HSP 90 | HSP90AB1 |
| *Licorice* | Gancao | MOL004974 | Heat shock protein HSP 90 | HSP90AB1 |
| *Licorice* | Gancao | MOL004980 | Heat shock protein HSP 90 | HSP90AB1 |
| *Licorice* | Gancao | MOL004989 | Heat shock protein HSP 90 | HSP90AB1 |
| *Licorice* | Gancao | MOL004990 | Heat shock protein HSP 90 | HSP90AB1 |
| *Licorice* | Gancao | MOL004991 | Heat shock protein HSP 90 | HSP90AB1 |
| *Licorice* | Gancao | MOL004993 | Heat shock protein HSP 90 | HSP90AB1 |
| *Licorice* | Gancao | MOL000500 | Heat shock protein HSP 90 | HSP90AB1 |
| *Licorice* | Gancao | MOL005000 | Heat shock protein HSP 90 | HSP90AB1 |
| *Licorice* | Gancao | MOL005001 | Heat shock protein HSP 90 | HSP90AB1 |
| *Licorice* | Gancao | MOL005003 | Heat shock protein HSP 90 | HSP90AB1 |
| *Licorice* | Gancao | MOL005007 | Heat shock protein HSP 90 | HSP90AB1 |
| *Licorice* | Gancao | MOL005008 | Heat shock protein HSP 90 | HSP90AB1 |
| *Licorice* | Gancao | MOL005016 | Heat shock protein HSP 90 | HSP90AB1 |
| *Licorice* | Gancao | MOL005017 | Heat shock protein HSP 90 | HSP90AB1 |
| *Licorice* | Gancao | MOL005020 | Heat shock protein HSP 90 | HSP90AB1 |
| *Licorice* | Gancao | MOL000098 | Heat shock protein HSP 90 | HSP90AB1 |
| *Licorice* | Gancao | MOL000098 | 78 kDa glucose-regulated protein | HSPA5 |
| *Licorice* | Gancao | MOL000098 | Heat shock protein beta-1 | HSPB1 |
| *Licorice* | Gancao | MOL002565 | 5-hydroxytryptamine 2A receptor | HTR2A |
| *Licorice* | Gancao | MOL000500 | 5-hydroxytryptamine 2A receptor | HTR2A |
| *Licorice* | Gancao | MOL001484 | 5-hydroxytryptamine receptor 3A | HTR3A |
| *Licorice* | Gancao | MOL004891 | 5-hydroxytryptamine receptor 3A | HTR3A |
| *Licorice* | Gancao | MOL000422 | Intercellular adhesion molecule 1 | ICAM1 |
| *Licorice* | Gancao | MOL000098 | Intercellular adhesion molecule 1 | ICAM1 |
| *Licorice* | Gancao | MOL000098 | Interferon gamma | IFNG |
| *Licorice* | Gancao | MOL000098 | Insulin-like growth factor II | IGF2 |
| *Licorice* | Gancao | MOL000098 | Insulin-like growth factor-binding protein 3 | IGFBP3 |
| *Licorice* | Gancao | MOL001484 | Ig gamma-1 chain C region | IGHG1 |
| *Licorice* | Gancao | MOL003896 | Ig gamma-1 chain C region | IGHG1 |
| *Licorice* | Gancao | MOL004828 | Ig gamma-1 chain C region | IGHG1 |
| *Licorice* | Gancao | MOL004829 | Ig gamma-1 chain C region | IGHG1 |
| *Licorice* | Gancao | MOL004848 | Ig gamma-1 chain C region | IGHG1 |
| *Licorice* | Gancao | MOL004908 | Ig gamma-1 chain C region | IGHG1 |
| *Licorice* | Gancao | MOL004957 | Ig gamma-1 chain C region | IGHG1 |
| *Licorice* | Gancao | MOL000422 | Inhibitor of nuclear factor kappa-B kinase subunit beta | IKBKB |
| *Licorice* | Gancao | MOL000098 | Interleukin-10 | IL10 |
| *Licorice* | Gancao | MOL000098 | Interleukin-1 alpha | IL1A |
| *Licorice* | Gancao | MOL000098 | Interleukin-1 beta | IL1B |
| *Licorice* | Gancao | MOL000098 | Interleukin-2 | IL2 |
| *Licorice* | Gancao | MOL000392 | Interleukin-4 | IL4 |
| *Licorice* | Gancao | MOL000098 | Interleukin-6 | IL6 |
| *Licorice* | Gancao | MOL000422 | Insulin receptor | INSR |
| *Licorice* | Gancao | MOL000098 | Insulin receptor | INSR |
| *Licorice* | Gancao | MOL000098 | Interferon regulatory factor 1 | IRF1 |
| *Licorice* | Gancao | MOL000392 | Transcription factor AP-1 | JUN |
| *Licorice* | Gancao | MOL000422 | Transcription factor AP-1 | JUN |
| *Licorice* | Gancao | MOL000098 | Transcription factor AP-1 | JUN |
| *Licorice* | Gancao | MOL004805 | Potassium voltage-gated channel subfamily H member 2 | KCNH2 |
| *Licorice* | Gancao | MOL004806 | Potassium voltage-gated channel subfamily H member 2 | KCNH2 |
| *Licorice* | Gancao | MOL004811 | Potassium voltage-gated channel subfamily H member 2 | KCNH2 |
| *Licorice* | Gancao | MOL004849 | Potassium voltage-gated channel subfamily H member 2 | KCNH2 |
| *Licorice* | Gancao | MOL004855 | Potassium voltage-gated channel subfamily H member 2 | KCNH2 |
| *Licorice* | Gancao | MOL004864 | Potassium voltage-gated channel subfamily H member 2 | KCNH2 |
| *Licorice* | Gancao | MOL004879 | Potassium voltage-gated channel subfamily H member 2 | KCNH2 |
| *Licorice* | Gancao | MOL004891 | Potassium voltage-gated channel subfamily H member 2 | KCNH2 |
| *Licorice* | Gancao | MOL004959 | Potassium voltage-gated channel subfamily H member 2 | KCNH2 |
| *Licorice* | Gancao | MOL004966 | Potassium voltage-gated channel subfamily H member 2 | KCNH2 |
| *Licorice* | Gancao | MOL004974 | Potassium voltage-gated channel subfamily H member 2 | KCNH2 |
| *Licorice* | Gancao | MOL004978 | Potassium voltage-gated channel subfamily H member 2 | KCNH2 |
| *Licorice* | Gancao | MOL005003 | Potassium voltage-gated channel subfamily H member 2 | KCNH2 |
| *Licorice* | Gancao | MOL005007 | Potassium voltage-gated channel subfamily H member 2 | KCNH2 |
| *Licorice* | Gancao | MOL000098 | Potassium voltage-gated channel subfamily H member 2 | KCNH2 |
| *Licorice* | Gancao | MOL004966 | Calcium-activated potassium channel subunit alpha 1 | KCNMA1 |
| *Licorice* | Gancao | MOL004974 | Calcium-activated potassium channel subunit alpha 1 | KCNMA1 |
| *Licorice* | Gancao | MOL004978 | Calcium-activated potassium channel subunit alpha 1 | KCNMA1 |
| *Licorice* | Gancao | MOL005007 | Calcium-activated potassium channel subunit alpha 1 | KCNMA1 |
| *Licorice* | Gancao | MOL002311 | Vascular endothelial growth factor receptor 2 | KDR |
| *Licorice* | Gancao | MOL004808 | Vascular endothelial growth factor receptor 2 | KDR |
| *Licorice* | Gancao | MOL004824 | Vascular endothelial growth factor receptor 2 | KDR |
| *Licorice* | Gancao | MOL004828 | Vascular endothelial growth factor receptor 2 | KDR |
| *Licorice* | Gancao | MOL004848 | Vascular endothelial growth factor receptor 2 | KDR |
| *Licorice* | Gancao | MOL004849 | Vascular endothelial growth factor receptor 2 | KDR |
| *Licorice* | Gancao | MOL004855 | Vascular endothelial growth factor receptor 2 | KDR |
| *Licorice* | Gancao | MOL004857 | Vascular endothelial growth factor receptor 2 | KDR |
| *Licorice* | Gancao | MOL004879 | Vascular endothelial growth factor receptor 2 | KDR |
| *Licorice* | Gancao | MOL004883 | Vascular endothelial growth factor receptor 2 | KDR |
| *Licorice* | Gancao | MOL004903 | Vascular endothelial growth factor receptor 2 | KDR |
| *Licorice* | Gancao | MOL004904 | Vascular endothelial growth factor receptor 2 | KDR |
| *Licorice* | Gancao | MOL004935 | Vascular endothelial growth factor receptor 2 | KDR |
| *Licorice* | Gancao | MOL004959 | Vascular endothelial growth factor receptor 2 | KDR |
| *Licorice* | Gancao | MOL004966 | Vascular endothelial growth factor receptor 2 | KDR |
| *Licorice* | Gancao | MOL005001 | Vascular endothelial growth factor receptor 2 | KDR |
| *Licorice* | Gancao | MOL005007 | Vascular endothelial growth factor receptor 2 | KDR |
| *Licorice* | Gancao | MOL005017 | Vascular endothelial growth factor receptor 2 | KDR |
| *Licorice* | Gancao | MOL001792 | Beta-lactamase | LACTB |
| *Licorice* | Gancao | MOL000392 | Beta-lactamase | LACTB |
| *Licorice* | Gancao | MOL004328 | Beta-lactamase | LACTB |
| *Licorice* | Gancao | MOL004941 | Beta-lactamase | LACTB |
| *Licorice* | Gancao | MOL004328 | Low-density lipoprotein receptor | LDLR |
| *Licorice* | Gancao | MOL003896 | Leukotriene A-4 hydrolase | LTA4H |
| *Licorice* | Gancao | MOL004835 | Leukotriene A-4 hydrolase | LTA4H |
| *Licorice* | Gancao | MOL001792 | Amine oxidase [flavin-containing] B | MAOB |
| *Licorice* | Gancao | MOL000354 | Amine oxidase [flavin-containing] B | MAOB |
| *Licorice* | Gancao | MOL003896 | Amine oxidase [flavin-containing] B | MAOB |
| *Licorice* | Gancao | MOL000392 | Amine oxidase [flavin-containing] B | MAOB |
| *Licorice* | Gancao | MOL004835 | Amine oxidase [flavin-containing] B | MAOB |
| *Licorice* | Gancao | MOL004941 | Amine oxidase [flavin-containing] B | MAOB |
| *Licorice* | Gancao | MOL004957 | Amine oxidase [flavin-containing] B | MAOB |
| *Licorice* | Gancao | MOL000098 | Amine oxidase [flavin-containing] B | MAOB |
| *Licorice* | Gancao | MOL004328 | Mitogen-activated protein kinase 1 | MAPK1 |
| *Licorice* | Gancao | MOL000497 | Mitogen-activated protein kinase 1 | MAPK1 |
| *Licorice* | Gancao | MOL000098 | Mitogen-activated protein kinase 1 | MAPK1 |
| *Licorice* | Gancao | MOL002565 | Mitogen-activated protein kinase 10 | MAPK10 |
| *Licorice* | Gancao | MOL002311 | Mitogen-activated protein kinase 14 | MAPK14 |
| *Licorice* | Gancao | MOL000354 | Mitogen-activated protein kinase 14 | MAPK14 |
| *Licorice* | Gancao | MOL003656 | Mitogen-activated protein kinase 14 | MAPK14 |
| *Licorice* | Gancao | MOL003896 | Mitogen-activated protein kinase 14 | MAPK14 |
| *Licorice* | Gancao | MOL000392 | Mitogen-activated protein kinase 14 | MAPK14 |
| *Licorice* | Gancao | MOL000417 | Mitogen-activated protein kinase 14 | MAPK14 |
| *Licorice* | Gancao | MOL004805 | Mitogen-activated protein kinase 14 | MAPK14 |
| *Licorice* | Gancao | MOL004810 | Mitogen-activated protein kinase 14 | MAPK14 |
| *Licorice* | Gancao | MOL004811 | Mitogen-activated protein kinase 14 | MAPK14 |
| *Licorice* | Gancao | MOL004814 | Mitogen-activated protein kinase 14 | MAPK14 |
| *Licorice* | Gancao | MOL004815 | Mitogen-activated protein kinase 14 | MAPK14 |
| *Licorice* | Gancao | MOL004820 | Mitogen-activated protein kinase 14 | MAPK14 |
| *Licorice* | Gancao | MOL004824 | Mitogen-activated protein kinase 14 | MAPK14 |
| *Licorice* | Gancao | MOL004828 | Mitogen-activated protein kinase 14 | MAPK14 |
| *Licorice* | Gancao | MOL004833 | Mitogen-activated protein kinase 14 | MAPK14 |
| *Licorice* | Gancao | MOL004835 | Mitogen-activated protein kinase 14 | MAPK14 |
| *Licorice* | Gancao | MOL004841 | Mitogen-activated protein kinase 14 | MAPK14 |
| *Licorice* | Gancao | MOL004848 | Mitogen-activated protein kinase 14 | MAPK14 |
| *Licorice* | Gancao | MOL004849 | Mitogen-activated protein kinase 14 | MAPK14 |
| *Licorice* | Gancao | MOL004863 | Mitogen-activated protein kinase 14 | MAPK14 |
| *Licorice* | Gancao | MOL004864 | Mitogen-activated protein kinase 14 | MAPK14 |
| *Licorice* | Gancao | MOL004883 | Mitogen-activated protein kinase 14 | MAPK14 |
| *Licorice* | Gancao | MOL004891 | Mitogen-activated protein kinase 14 | MAPK14 |
| *Licorice* | Gancao | MOL004898 | Mitogen-activated protein kinase 14 | MAPK14 |
| *Licorice* | Gancao | MOL004907 | Mitogen-activated protein kinase 14 | MAPK14 |
| *Licorice* | Gancao | MOL004908 | Mitogen-activated protein kinase 14 | MAPK14 |
| *Licorice* | Gancao | MOL004911 | Mitogen-activated protein kinase 14 | MAPK14 |
| *Licorice* | Gancao | MOL004912 | Mitogen-activated protein kinase 14 | MAPK14 |
| *Licorice* | Gancao | MOL004913 | Mitogen-activated protein kinase 14 | MAPK14 |
| *Licorice* | Gancao | MOL004914 | Mitogen-activated protein kinase 14 | MAPK14 |
| *Licorice* | Gancao | MOL004915 | Mitogen-activated protein kinase 14 | MAPK14 |
| *Licorice* | Gancao | MOL004957 | Mitogen-activated protein kinase 14 | MAPK14 |
| *Licorice* | Gancao | MOL004959 | Mitogen-activated protein kinase 14 | MAPK14 |
| *Licorice* | Gancao | MOL004961 | Mitogen-activated protein kinase 14 | MAPK14 |
| *Licorice* | Gancao | MOL004966 | Mitogen-activated protein kinase 14 | MAPK14 |
| *Licorice* | Gancao | MOL000497 | Mitogen-activated protein kinase 14 | MAPK14 |
| *Licorice* | Gancao | MOL004974 | Mitogen-activated protein kinase 14 | MAPK14 |
| *Licorice* | Gancao | MOL004978 | Mitogen-activated protein kinase 14 | MAPK14 |
| *Licorice* | Gancao | MOL004990 | Mitogen-activated protein kinase 14 | MAPK14 |
| *Licorice* | Gancao | MOL004991 | Mitogen-activated protein kinase 14 | MAPK14 |
| *Licorice* | Gancao | MOL000500 | Mitogen-activated protein kinase 14 | MAPK14 |
| *Licorice* | Gancao | MOL005000 | Mitogen-activated protein kinase 14 | MAPK14 |
| *Licorice* | Gancao | MOL005003 | Mitogen-activated protein kinase 14 | MAPK14 |
| *Licorice* | Gancao | MOL005012 | Mitogen-activated protein kinase 14 | MAPK14 |
| *Licorice* | Gancao | MOL005016 | Mitogen-activated protein kinase 14 | MAPK14 |
| *Licorice* | Gancao | MOL005017 | Mitogen-activated protein kinase 14 | MAPK14 |
| *Licorice* | Gancao | MOL005020 | Mitogen-activated protein kinase 14 | MAPK14 |
| *Licorice* | Gancao | MOL004328 | Mitogen-activated protein kinase 3 | MAPK3 |
| *Licorice* | Gancao | MOL000422 | Mitogen-activated protein kinase 8 | MAPK8 |
| *Licorice* | Gancao | MOL000098 | Maltase-glucoamylase, intestinal | MGAM |
| *Licorice* | Gancao | MOL000422 | Interstitial collagenase | MMP1 |
| *Licorice* | Gancao | MOL000098 | Interstitial collagenase | MMP1 |
| *Licorice* | Gancao | MOL000098 | 72 kDa type IV collagenase | MMP2 |
| *Licorice* | Gancao | MOL000098 | Stromelysin-1 | MMP3 |
| *Licorice* | Gancao | MOL000098 | Matrix metalloproteinase-9 | MMP9 |
| *Licorice* | Gancao | MOL000098 | Myeloperoxidase | MPO |
| *Licorice* | Gancao | MOL000392 | NADH-ubiquinone oxidoreductase chain 6 | MT-ND6 |
| *Licorice* | Gancao | MOL004328 | Microsomal triglyceride transfer protein large subunit | MTTP |
| *Licorice* | Gancao | MOL000098 | Myc proto-oncogene protein | MYC |
| *Licorice* | Gancao | MOL000354 | Neutrophil cytosol factor 1 | NCF1 |
| *Licorice* | Gancao | MOL000098 | Neutrophil cytosol factor 1 | NCF1 |
| *Licorice* | Gancao | MOL000354 | Nuclear receptor coactivator 1 | NCOA1 |
| *Licorice* | Gancao | MOL003896 | Nuclear receptor coactivator 1 | NCOA1 |
| *Licorice* | Gancao | MOL004820 | Nuclear receptor coactivator 1 | NCOA1 |
| *Licorice* | Gancao | MOL004829 | Nuclear receptor coactivator 1 | NCOA1 |
| *Licorice* | Gancao | MOL004833 | Nuclear receptor coactivator 1 | NCOA1 |
| *Licorice* | Gancao | MOL004835 | Nuclear receptor coactivator 1 | NCOA1 |
| *Licorice* | Gancao | MOL004849 | Nuclear receptor coactivator 1 | NCOA1 |
| *Licorice* | Gancao | MOL004885 | Nuclear receptor coactivator 1 | NCOA1 |
| *Licorice* | Gancao | MOL004891 | Nuclear receptor coactivator 1 | NCOA1 |
| *Licorice* | Gancao | MOL004908 | Nuclear receptor coactivator 1 | NCOA1 |
| *Licorice* | Gancao | MOL004959 | Nuclear receptor coactivator 1 | NCOA1 |
| *Licorice* | Gancao | MOL004966 | Nuclear receptor coactivator 1 | NCOA1 |
| *Licorice* | Gancao | MOL004974 | Nuclear receptor coactivator 1 | NCOA1 |
| *Licorice* | Gancao | MOL004978 | Nuclear receptor coactivator 1 | NCOA1 |
| *Licorice* | Gancao | MOL004993 | Nuclear receptor coactivator 1 | NCOA1 |
| *Licorice* | Gancao | MOL005007 | Nuclear receptor coactivator 1 | NCOA1 |
| *Licorice* | Gancao | MOL000239 | Nuclear receptor coactivator 2 | NCOA2 |
| *Licorice* | Gancao | MOL000354 | Nuclear receptor coactivator 2 | NCOA2 |
| *Licorice* | Gancao | MOL000359 | Nuclear receptor coactivator 2 | NCOA2 |
| *Licorice* | Gancao | MOL003656 | Nuclear receptor coactivator 2 | NCOA2 |
| *Licorice* | Gancao | MOL003896 | Nuclear receptor coactivator 2 | NCOA2 |
| *Licorice* | Gancao | MOL000417 | Nuclear receptor coactivator 2 | NCOA2 |
| *Licorice* | Gancao | MOL000422 | Nuclear receptor coactivator 2 | NCOA2 |
| *Licorice* | Gancao | MOL004808 | Nuclear receptor coactivator 2 | NCOA2 |
| *Licorice* | Gancao | MOL004811 | Nuclear receptor coactivator 2 | NCOA2 |
| *Licorice* | Gancao | MOL004815 | Nuclear receptor coactivator 2 | NCOA2 |
| *Licorice* | Gancao | MOL004820 | Nuclear receptor coactivator 2 | NCOA2 |
| *Licorice* | Gancao | MOL004848 | Nuclear receptor coactivator 2 | NCOA2 |
| *Licorice* | Gancao | MOL004849 | Nuclear receptor coactivator 2 | NCOA2 |
| *Licorice* | Gancao | MOL004855 | Nuclear receptor coactivator 2 | NCOA2 |
| *Licorice* | Gancao | MOL004856 | Nuclear receptor coactivator 2 | NCOA2 |
| *Licorice* | Gancao | MOL004857 | Nuclear receptor coactivator 2 | NCOA2 |
| *Licorice* | Gancao | MOL004863 | Nuclear receptor coactivator 2 | NCOA2 |
| *Licorice* | Gancao | MOL004864 | Nuclear receptor coactivator 2 | NCOA2 |
| *Licorice* | Gancao | MOL004879 | Nuclear receptor coactivator 2 | NCOA2 |
| *Licorice* | Gancao | MOL004883 | Nuclear receptor coactivator 2 | NCOA2 |
| *Licorice* | Gancao | MOL004898 | Nuclear receptor coactivator 2 | NCOA2 |
| *Licorice* | Gancao | MOL004908 | Nuclear receptor coactivator 2 | NCOA2 |
| *Licorice* | Gancao | MOL004911 | Nuclear receptor coactivator 2 | NCOA2 |
| *Licorice* | Gancao | MOL004949 | Nuclear receptor coactivator 2 | NCOA2 |
| *Licorice* | Gancao | MOL004959 | Nuclear receptor coactivator 2 | NCOA2 |
| *Licorice* | Gancao | MOL004961 | Nuclear receptor coactivator 2 | NCOA2 |
| *Licorice* | Gancao | MOL004966 | Nuclear receptor coactivator 2 | NCOA2 |
| *Licorice* | Gancao | MOL000497 | Nuclear receptor coactivator 2 | NCOA2 |
| *Licorice* | Gancao | MOL004974 | Nuclear receptor coactivator 2 | NCOA2 |
| *Licorice* | Gancao | MOL004978 | Nuclear receptor coactivator 2 | NCOA2 |
| *Licorice* | Gancao | MOL004980 | Nuclear receptor coactivator 2 | NCOA2 |
| *Licorice* | Gancao | MOL004985 | Nuclear receptor coactivator 2 | NCOA2 |
| *Licorice* | Gancao | MOL004988 | Nuclear receptor coactivator 2 | NCOA2 |
| *Licorice* | Gancao | MOL004991 | Nuclear receptor coactivator 2 | NCOA2 |
| *Licorice* | Gancao | MOL004996 | Nuclear receptor coactivator 2 | NCOA2 |
| *Licorice* | Gancao | MOL005000 | Nuclear receptor coactivator 2 | NCOA2 |
| *Licorice* | Gancao | MOL005001 | Nuclear receptor coactivator 2 | NCOA2 |
| *Licorice* | Gancao | MOL005003 | Nuclear receptor coactivator 2 | NCOA2 |
| *Licorice* | Gancao | MOL005007 | Nuclear receptor coactivator 2 | NCOA2 |
| *Licorice* | Gancao | MOL005016 | Nuclear receptor coactivator 2 | NCOA2 |
| *Licorice* | Gancao | MOL005018 | Nuclear receptor coactivator 2 | NCOA2 |
| *Licorice* | Gancao | MOL005020 | Nuclear receptor coactivator 2 | NCOA2 |
| *Licorice* | Gancao | MOL000098 | Nuclear receptor coactivator 2 | NCOA2 |
| *Licorice* | Gancao | MOL000098 | Nuclear factor erythroid 2-related factor 2 | NFE2L2 |
| *Licorice* | Gancao | MOL000098 | NF-kappa-B inhibitor alpha | NFKBIA |
| *Licorice* | Gancao | MOL000098 | Homeobox protein Nkx-3.1 | NKX3-1 |
| *Licorice* | Gancao | MOL002311 | Nitric oxide synthase, inducible | NOS2 |
| *Licorice* | Gancao | MOL000239 | Nitric oxide synthase, inducible | NOS2 |
| *Licorice* | Gancao | MOL002565 | Nitric oxide synthase, inducible | NOS2 |
| *Licorice* | Gancao | MOL000354 | Nitric oxide synthase, inducible | NOS2 |
| *Licorice* | Gancao | MOL003656 | Nitric oxide synthase, inducible | NOS2 |
| *Licorice* | Gancao | MOL003896 | Nitric oxide synthase, inducible | NOS2 |
| *Licorice* | Gancao | MOL000392 | Nitric oxide synthase, inducible | NOS2 |
| *Licorice* | Gancao | MOL000417 | Nitric oxide synthase, inducible | NOS2 |
| *Licorice* | Gancao | MOL000422 | Nitric oxide synthase, inducible | NOS2 |
| *Licorice* | Gancao | MOL004805 | Nitric oxide synthase, inducible | NOS2 |
| *Licorice* | Gancao | MOL004806 | Nitric oxide synthase, inducible | NOS2 |
| *Licorice* | Gancao | MOL004808 | Nitric oxide synthase, inducible | NOS2 |
| *Licorice* | Gancao | MOL004810 | Nitric oxide synthase, inducible | NOS2 |
| *Licorice* | Gancao | MOL004811 | Nitric oxide synthase, inducible | NOS2 |
| *Licorice* | Gancao | MOL004814 | Nitric oxide synthase, inducible | NOS2 |
| *Licorice* | Gancao | MOL004815 | Nitric oxide synthase, inducible | NOS2 |
| *Licorice* | Gancao | MOL004820 | Nitric oxide synthase, inducible | NOS2 |
| *Licorice* | Gancao | MOL004824 | Nitric oxide synthase, inducible | NOS2 |
| *Licorice* | Gancao | MOL004827 | Nitric oxide synthase, inducible | NOS2 |
| *Licorice* | Gancao | MOL004828 | Nitric oxide synthase, inducible | NOS2 |
| *Licorice* | Gancao | MOL004833 | Nitric oxide synthase, inducible | NOS2 |
| *Licorice* | Gancao | MOL004835 | Nitric oxide synthase, inducible | NOS2 |
| *Licorice* | Gancao | MOL004838 | Nitric oxide synthase, inducible | NOS2 |
| *Licorice* | Gancao | MOL004841 | Nitric oxide synthase, inducible | NOS2 |
| *Licorice* | Gancao | MOL004848 | Nitric oxide synthase, inducible | NOS2 |
| *Licorice* | Gancao | MOL004849 | Nitric oxide synthase, inducible | NOS2 |
| *Licorice* | Gancao | MOL004855 | Nitric oxide synthase, inducible | NOS2 |
| *Licorice* | Gancao | MOL004856 | Nitric oxide synthase, inducible | NOS2 |
| *Licorice* | Gancao | MOL004857 | Nitric oxide synthase, inducible | NOS2 |
| *Licorice* | Gancao | MOL004863 | Nitric oxide synthase, inducible | NOS2 |
| *Licorice* | Gancao | MOL004864 | Nitric oxide synthase, inducible | NOS2 |
| *Licorice* | Gancao | MOL004879 | Nitric oxide synthase, inducible | NOS2 |
| *Licorice* | Gancao | MOL004883 | Nitric oxide synthase, inducible | NOS2 |
| *Licorice* | Gancao | MOL004884 | Nitric oxide synthase, inducible | NOS2 |
| *Licorice* | Gancao | MOL004885 | Nitric oxide synthase, inducible | NOS2 |
| *Licorice* | Gancao | MOL004891 | Nitric oxide synthase, inducible | NOS2 |
| *Licorice* | Gancao | MOL004904 | Nitric oxide synthase, inducible | NOS2 |
| *Licorice* | Gancao | MOL004907 | Nitric oxide synthase, inducible | NOS2 |
| *Licorice* | Gancao | MOL004908 | Nitric oxide synthase, inducible | NOS2 |
| *Licorice* | Gancao | MOL004910 | Nitric oxide synthase, inducible | NOS2 |
| *Licorice* | Gancao | MOL004911 | Nitric oxide synthase, inducible | NOS2 |
| *Licorice* | Gancao | MOL004912 | Nitric oxide synthase, inducible | NOS2 |
| *Licorice* | Gancao | MOL004915 | Nitric oxide synthase, inducible | NOS2 |
| *Licorice* | Gancao | MOL004945 | Nitric oxide synthase, inducible | NOS2 |
| *Licorice* | Gancao | MOL004948 | Nitric oxide synthase, inducible | NOS2 |
| *Licorice* | Gancao | MOL004949 | Nitric oxide synthase, inducible | NOS2 |
| *Licorice* | Gancao | MOL004957 | Nitric oxide synthase, inducible | NOS2 |
| *Licorice* | Gancao | MOL004959 | Nitric oxide synthase, inducible | NOS2 |
| *Licorice* | Gancao | MOL004961 | Nitric oxide synthase, inducible | NOS2 |
| *Licorice* | Gancao | MOL004966 | Nitric oxide synthase, inducible | NOS2 |
| *Licorice* | Gancao | MOL000497 | Nitric oxide synthase, inducible | NOS2 |
| *Licorice* | Gancao | MOL004974 | Nitric oxide synthase, inducible | NOS2 |
| *Licorice* | Gancao | MOL004978 | Nitric oxide synthase, inducible | NOS2 |
| *Licorice* | Gancao | MOL004989 | Nitric oxide synthase, inducible | NOS2 |
| *Licorice* | Gancao | MOL004990 | Nitric oxide synthase, inducible | NOS2 |
| *Licorice* | Gancao | MOL004991 | Nitric oxide synthase, inducible | NOS2 |
| *Licorice* | Gancao | MOL000500 | Nitric oxide synthase, inducible | NOS2 |
| *Licorice* | Gancao | MOL005000 | Nitric oxide synthase, inducible | NOS2 |
| *Licorice* | Gancao | MOL005003 | Nitric oxide synthase, inducible | NOS2 |
| *Licorice* | Gancao | MOL005007 | Nitric oxide synthase, inducible | NOS2 |
| *Licorice* | Gancao | MOL005008 | Nitric oxide synthase, inducible | NOS2 |
| *Licorice* | Gancao | MOL005012 | Nitric oxide synthase, inducible | NOS2 |
| *Licorice* | Gancao | MOL005016 | Nitric oxide synthase, inducible | NOS2 |
| *Licorice* | Gancao | MOL005018 | Nitric oxide synthase, inducible | NOS2 |
| *Licorice* | Gancao | MOL005020 | Nitric oxide synthase, inducible | NOS2 |
| *Licorice* | Gancao | MOL000354 | Nitric-oxide synthase, endothelial | NOS3 |
| *Licorice* | Gancao | MOL003896 | Nitric-oxide synthase, endothelial | NOS3 |
| *Licorice* | Gancao | MOL000392 | Nitric-oxide synthase, endothelial | NOS3 |
| *Licorice* | Gancao | MOL000422 | Nitric-oxide synthase, endothelial | NOS3 |
| *Licorice* | Gancao | MOL004828 | Nitric-oxide synthase, endothelial | NOS3 |
| *Licorice* | Gancao | MOL004829 | Nitric-oxide synthase, endothelial | NOS3 |
| *Licorice* | Gancao | MOL004910 | Nitric-oxide synthase, endothelial | NOS3 |
| *Licorice* | Gancao | MOL004959 | Nitric-oxide synthase, endothelial | NOS3 |
| *Licorice* | Gancao | MOL004978 | Nitric-oxide synthase, endothelial | NOS3 |
| *Licorice* | Gancao | MOL004991 | Nitric-oxide synthase, endothelial | NOS3 |
| *Licorice* | Gancao | MOL005000 | Nitric-oxide synthase, endothelial | NOS3 |
| *Licorice* | Gancao | MOL005003 | Nitric-oxide synthase, endothelial | NOS3 |
| *Licorice* | Gancao | MOL000098 | Nitric-oxide synthase, endothelial | NOS3 |
| *Licorice* | Gancao | MOL000098 | Nitric oxide synthase, endothelial | NOS3 |
| *Licorice* | Gancao | MOL000098 | Puromycin-sensitive aminopeptidase | NPEPPS |
| *Licorice* | Gancao | MOL000098 | NAD(P)H dehydrogenase [quinone] 1 | NQO1 |
| *Licorice* | Gancao | MOL000422 | Nuclear receptor subfamily 1 group I member 2 | NR1I2 |
| *Licorice* | Gancao | MOL000098 | Nuclear receptor subfamily 1 group I member 2 | NR1I2 |
| *Licorice* | Gancao | MOL000422 | Nuclear receptor subfamily 1 group I member 3 | NR1I3 |
| *Licorice* | Gancao | MOL000098 | Nuclear receptor subfamily 1 group I member 3 | NR1I3 |
| *Licorice* | Gancao | MOL000359 | Mineralocorticoid receptor | NR3C2 |
| *Licorice* | Gancao | MOL000098 | Ornithine decarboxylase | ODC1 |
| *Licorice* | Gancao | MOL000354 | Oxidized low-density lipoprotein receptor 1 | OLR1 |
| *Licorice* | Gancao | MOL002565 | Delta-type opioid receptor | OPRD1 |
| *Licorice* | Gancao | MOL004891 | Delta-type opioid receptor | OPRD1 |
| *Licorice* | Gancao | MOL001484 | Mu-type opioid receptor | OPRM1 |
| *Licorice* | Gancao | MOL002565 | Mu-type opioid receptor | OPRM1 |
| *Licorice* | Gancao | MOL003896 | Mu-type opioid receptor | OPRM1 |
| *Licorice* | Gancao | MOL004891 | Mu-type opioid receptor | OPRM1 |
| *Licorice* | Gancao | MOL000098 | Poly [ADP-ribose] polymerase 1 | PARP1 |
| *Licorice* | Gancao | MOL000098 | Procollagen C-endopeptidase enhancer 1 | PCOLCE |
| *Licorice* | Gancao | MOL002565 | CGMP-inhibited 3',5'-cyclic phosphodiesterase A | PDE3A |
| *Licorice* | Gancao | MOL003896 | CGMP-inhibited 3',5'-cyclic phosphodiesterase A | PDE3A |
| *Licorice* | Gancao | MOL000392 | CGMP-inhibited 3',5'-cyclic phosphodiesterase A | PDE3A |
| *Licorice* | Gancao | MOL000417 | CGMP-inhibited 3',5'-cyclic phosphodiesterase A | PDE3A |
| *Licorice* | Gancao | MOL004828 | CGMP-inhibited 3',5'-cyclic phosphodiesterase A | PDE3A |
| *Licorice* | Gancao | MOL004829 | CGMP-inhibited 3',5'-cyclic phosphodiesterase A | PDE3A |
| *Licorice* | Gancao | MOL004835 | CGMP-inhibited 3',5'-cyclic phosphodiesterase | PDE3A |
| *Licorice* | Gancao | MOL004841 | CGMP-inhibited 3',5'-cyclic phosphodiesterase A | PDE3A |
| *Licorice* | Gancao | MOL004910 | CGMP-inhibited 3',5'-cyclic phosphodiesterase A | PDE3A |
| *Licorice* | Gancao | MOL004941 | CGMP-inhibited 3',5'-cyclic phosphodiesterase A | PDE3A |
| *Licorice* | Gancao | MOL004945 | CGMP-inhibited 3',5'-cyclic phosphodiesterase A | PDE3A |
| *Licorice* | Gancao | MOL004957 | CGMP-inhibited 3',5'-cyclic phosphodiesterase A | PDE3A |
| *Licorice* | Gancao | MOL004991 | CGMP-inhibited 3',5'-cyclic phosphodiesterase A | PDE3A |
| *Licorice* | Gancao | MOL000500 | CGMP-inhibited 3',5'-cyclic phosphodiesterase A | PDE3A |
| *Licorice* | Gancao | MOL000211 | Progesterone receptor | PGR |
| *Licorice* | Gancao | MOL000359 | Progesterone receptor | PGR |
| *Licorice* | Gancao | MOL000422 | Progesterone receptor | PGR |
| *Licorice* | Gancao | MOL001484 | Phosphatidylinositol-4,5-bisphosphate 3-kinase catalytic subunit, gamma isoform | PIK3CG |
| *Licorice* | Gancao | MOL001792 | Phosphatidylinositol-4,5-bisphosphate 3-kinase catalytic subunit, gamma isoform | PIK3CG |
| *Licorice* | Gancao | MOL002565 | Phosphatidylinositol-4,5-bisphosphate 3-kinase catalytic subunit, gamma isoform | PIK3CG |
| *Licorice* | Gancao | MOL000354 | Phosphatidylinositol-4,5-bisphosphate 3-kinase catalytic subunit, gamma isoform | PIK3CG |
| *Licorice* | Gancao | MOL000422 | Phosphatidylinositol-4,5-bisphosphate 3-kinase catalytic subunit, gamma isoform | PIK3CG |
| *Licorice* | Gancao | MOL004328 | Phosphatidylinositol-4,5-bisphosphate 3-kinase catalytic subunit, gamma isoform | PIK3CG |
| *Licorice* | Gancao | MOL004814 | Phosphatidylinositol-4,5-bisphosphate 3-kinase catalytic subunit, gamma isoform | PIK3CG |
| *Licorice* | Gancao | MOL004838 | Phosphatidylinositol-4,5-bisphosphate 3-kinase catalytic subunit, gamma isoform | PIK3CG |
| *Licorice* | Gancao | MOL004891 | Phosphatidylinositol-4,5-bisphosphate 3-kinase catalytic subunit, gamma isoform | PIK3CG |
| *Licorice* | Gancao | MOL004907 | Phosphatidylinositol-4,5-bisphosphate 3-kinase catalytic subunit, gamma isoform | PIK3CG |
| *Licorice* | Gancao | MOL004941 | Phosphatidylinositol-4,5-bisphosphate 3-kinase catalytic subunit, gamma isoform | PIK3CG |
| *Licorice* | Gancao | MOL004959 | Phosphatidylinositol-4,5-bisphosphate 3-kinase catalytic subunit, gamma isoform | PIK3CG |
| *Licorice* | Gancao | MOL000098 | Phosphatidylinositol-4,5-bisphosphate 3-kinase catalytic subunit, gamma isoform | PIK3CG |
| *Licorice* | Gancao | MOL002311 | Proto-oncogene serine/threonine-protein kinase Pim-1 | PIM1 |
| *Licorice* | Gancao | MOL002565 | Proto-oncogene serine/threonine-protein kinase Pim-1 | PIM1 |
| *Licorice* | Gancao | MOL000354 | Proto-oncogene serine/threonine-protein kinase Pim-1 | PIM1 |
| *Licorice* | Gancao | MOL003656 | Proto-oncogene serine/threonine-protein kinase Pim-1 | PIM1 |
| *Licorice* | Gancao | MOL003896 | Proto-oncogene serine/threonine-protein kinase Pim-1 | PIM1 |
| *Licorice* | Gancao | MOL000392 | Proto-oncogene serine/threonine-protein kinase Pim-1 | PIM1 |
| *Licorice* | Gancao | MOL000417 | Proto-oncogene serine/threonine-protein kinase Pim-1 | PIM1 |
| *Licorice* | Gancao | MOL004805 | Proto-oncogene serine/threonine-protein kinase Pim-1 | PIM1 |
| *Licorice* | Gancao | MOL004806 | Proto-oncogene serine/threonine-protein kinase Pim-1 | PIM1 |
| *Licorice* | Gancao | MOL004808 | Proto-oncogene serine/threonine-protein kinase Pim-1 | PIM1 |
| *Licorice* | Gancao | MOL004810 | Proto-oncogene serine/threonine-protein kinase Pim-1 | PIM1 |
| *Licorice* | Gancao | MOL004811 | Proto-oncogene serine/threonine-protein kinase Pim-1 | PIM1 |
| *Licorice* | Gancao | MOL004814 | Proto-oncogene serine/threonine-protein kinase Pim-1 | PIM1 |
| *Licorice* | Gancao | MOL004815 | Proto-oncogene serine/threonine-protein kinase Pim-1 | PIM1 |
| *Licorice* | Gancao | MOL004820 | Proto-oncogene serine/threonine-protein kinase Pim-1 | PIM1 |
| *Licorice* | Gancao | MOL004824 | Proto-oncogene serine/threonine-protein kinase Pim-1 | PIM1 |
| *Licorice* | Gancao | MOL004828 | Proto-oncogene serine/threonine-protein kinase Pim-1 | PIM1 |
| *Licorice* | Gancao | MOL004833 | Proto-oncogene serine/threonine-protein kinase Pim-1 | PIM1 |
| *Licorice* | Gancao | MOL004841 | Proto-oncogene serine/threonine-protein kinase Pim-1 | PIM1 |
| *Licorice* | Gancao | MOL004848 | Proto-oncogene serine/threonine-protein kinase Pim-1 | PIM1 |
| *Licorice* | Gancao | MOL004849 | Proto-oncogene serine/threonine-protein kinase Pim-1 | PIM1 |
| *Licorice* | Gancao | MOL004855 | Proto-oncogene serine/threonine-protein kinase Pim-1 | PIM1 |
| *Licorice* | Gancao | MOL004856 | Proto-oncogene serine/threonine-protein kinase Pim-1 | PIM1 |
| *Licorice* | Gancao | MOL004857 | Proto-oncogene serine/threonine-protein kinase Pim-1 | PIM1 |
| *Licorice* | Gancao | MOL004863 | Proto-oncogene serine/threonine-protein kinase Pim-1 | PIM1 |
| *Licorice* | Gancao | MOL004864 | Proto-oncogene serine/threonine-protein kinase Pim-1 | PIM1 |
| *Licorice* | Gancao | MOL004866 | Proto-oncogene serine/threonine-protein kinase Pim-1 | PIM1 |
| *Licorice* | Gancao | MOL004879 | Proto-oncogene serine/threonine-protein kinase Pim-1 | PIM1 |
| *Licorice* | Gancao | MOL004883 | Proto-oncogene serine/threonine-protein kinase Pim-1 | PIM1 |
| *Licorice* | Gancao | MOL004884 | Proto-oncogene serine/threonine-protein kinase Pim-1 | PIM1 |
| *Licorice* | Gancao | MOL004885 | Proto-oncogene serine/threonine-protein kinase Pim-1 | PIM1 |
| *Licorice* | Gancao | MOL004891 | Proto-oncogene serine/threonine-protein kinase Pim-1 | PIM1 |
| *Licorice* | Gancao | MOL004898 | Proto-oncogene serine/threonine-protein kinase Pim-1 | PIM1 |
| *Licorice* | Gancao | MOL004904 | Proto-oncogene serine/threonine-protein kinase Pim-1 | PIM1 |
| *Licorice* | Gancao | MOL004907 | Proto-oncogene serine/threonine-protein kinase Pim-1 | PIM1 |
| *Licorice* | Gancao | MOL004908 | Proto-oncogene serine/threonine-protein kinase Pim-1 | PIM1 |
| *Licorice* | Gancao | MOL004911 | Proto-oncogene serine/threonine-protein kinase Pim-1 | PIM1 |
| *Licorice* | Gancao | MOL004912 | Proto-oncogene serine/threonine-protein kinase Pim-1 | PIM1 |
| *Licorice* | Gancao | MOL004915 | Proto-oncogene serine/threonine-protein kinase Pim-1 | PIM1 |
| *Licorice* | Gancao | MOL004948 | Proto-oncogene serine/threonine-protein kinase Pim-1 | PIM1 |
| *Licorice* | Gancao | MOL004949 | Proto-oncogene serine/threonine-protein kinase Pim-1 | PIM1 |
| *Licorice* | Gancao | MOL004957 | Proto-oncogene serine/threonine-protein kinase Pim-1 | PIM1 |
| *Licorice* | Gancao | MOL004959 | Proto-oncogene serine/threonine-protein kinase Pim-1 | PIM1 |
| *Licorice* | Gancao | MOL004966 | Proto-oncogene serine/threonine-protein kinase Pim-1 | PIM1 |
| *Licorice* | Gancao | MOL000497 | Proto-oncogene serine/threonine-protein kinase Pim-1 | PIM1 |
| *Licorice* | Gancao | MOL004974 | Proto-oncogene serine/threonine-protein kinase Pim-1 | PIM1 |
| *Licorice* | Gancao | MOL004978 | Proto-oncogene serine/threonine-protein kinase Pim-1 | PIM1 |
| *Licorice* | Gancao | MOL004980 | Proto-oncogene serine/threonine-protein kinase Pim-1 | PIM1 |
| *Licorice* | Gancao | MOL004988 | Proto-oncogene serine/threonine-protein kinase Pim-1 | PIM1 |
| *Licorice* | Gancao | MOL004990 | Proto-oncogene serine/threonine-protein kinase Pim-1 | PIM1 |
| *Licorice* | Gancao | MOL000500 | Proto-oncogene serine/threonine-protein kinase Pim-1 | PIM1 |
| *Licorice* | Gancao | MOL005000 | Proto-oncogene serine/threonine-protein kinase Pim-1 | PIM1 |
| *Licorice* | Gancao | MOL005001 | Proto-oncogene serine/threonine-protein kinase Pim-1 | PIM1 |
| *Licorice* | Gancao | MOL005003 | Proto-oncogene serine/threonine-protein kinase Pim-1 | PIM1 |
| *Licorice* | Gancao | MOL005007 | Proto-oncogene serine/threonine-protein kinase Pim-1 | PIM1 |
| *Licorice* | Gancao | MOL005008 | Proto-oncogene serine/threonine-protein kinase Pim-1 | PIM1 |
| *Licorice* | Gancao | MOL005012 | Proto-oncogene serine/threonine-protein kinase Pim-1 | PIM1 |
| *Licorice* | Gancao | MOL005016 | Proto-oncogene serine/threonine-protein kinase Pim-1 | PIM1 |
| *Licorice* | Gancao | MOL005017 | Proto-oncogene serine/threonine-protein kinase Pim-1 | PIM1 |
| *Licorice* | Gancao | MOL005018 | Proto-oncogene serine/threonine-protein kinase Pim-1 | PIM1 |
| *Licorice* | Gancao | MOL005020 | Proto-oncogene serine/threonine-protein kinase Pim-1 | PIM1 |
| *Licorice* | Gancao | MOL001792 | cAMP-dependent protein kinase inhibitor alpha | PKIA |
| *Licorice* | Gancao | MOL003896 | cAMP-dependent protein kinase inhibitor alpha | PKIA |
| *Licorice* | Gancao | MOL000392 | cAMP-dependent protein kinase inhibitor alpha | PKIA |
| *Licorice* | Gancao | MOL004835 | cAMP-dependent protein kinase inhibitor alpha | PKIA |
| *Licorice* | Gancao | MOL004941 | cAMP-dependent protein kinase inhibitor alpha | PKIA |
| *Licorice* | Gancao | MOL004957 | cAMP-dependent protein kinase inhibitor alpha | PKIA |
| *Licorice* | Gancao | MOL000500 | cAMP-dependent protein kinase inhibitor alpha | PKIA |
| *Licorice* | Gancao | MOL000098 | Tissue-type plasminogen activator | PLAT |
| *Licorice* | Gancao | MOL000098 | Urokinase-type plasminogen activator | PLAU |
| *Licorice* | Gancao | MOL004328 | Phospholipase B1, membrane-associated | PLB1 |
| *Licorice* | Gancao | MOL000098 | Serum paraoxonase/arylesterase 1 | PON1 |
| *Licorice* | Gancao | MOL000098 | NADPH--cytochrome P450 reductase | POR |
| *Licorice* | Gancao | MOL004328 | Peroxisome proliferator-activated receptor alpha | PPARA |
| *Licorice* | Gancao | MOL000098 | Peroxisome proliferator-activated receptor alpha | PPARA |
| *Licorice* | Gancao | MOL000354 | Peroxisome proliferator activated receptor delta | PPARD |
| *Licorice* | Gancao | MOL005007 | Peroxisome proliferator activated receptor delta | PPARD |
| *Licorice* | Gancao | MOL000098 | Peroxisome proliferator-activated receptor delta | PPARD |
| *Licorice* | Gancao | MOL002311 | Peroxisome proliferator activated receptor gamma | PPARG |
| *Licorice* | Gancao | MOL000354 | Peroxisome proliferator activated receptor gamma | PPARG |
| *Licorice* | Gancao | MOL003656 | Peroxisome proliferator activated receptor gamma | PPARG |
| *Licorice* | Gancao | MOL003896 | Peroxisome proliferator activated receptor gamma | PPARG |
| *Licorice* | Gancao | MOL000392 | Peroxisome proliferator activated receptor gamma | PPARG |
| *Licorice* | Gancao | MOL000392 | Peroxisome proliferator-activated receptor gamma | PPARG |
| *Licorice* | Gancao | MOL000417 | Peroxisome proliferator activated receptor gamma | PPARG |
| *Licorice* | Gancao | MOL000422 | Peroxisome proliferator activated receptor gamma | PPARG |
| *Licorice* | Gancao | MOL000422 | Peroxisome proliferator-activated receptor gamma | PPARG |
| *Licorice* | Gancao | MOL004328 | Peroxisome proliferator-activated receptor gamma | PPARG |
| *Licorice* | Gancao | MOL004805 | Peroxisome proliferator activated receptor gamma | PPARG |
| *Licorice* | Gancao | MOL004808 | Peroxisome proliferator activated receptor gamma | PPARG |
| *Licorice* | Gancao | MOL004810 | Peroxisome proliferator activated receptor gamma | PPARG |
| *Licorice* | Gancao | MOL004811 | Peroxisome proliferator activated receptor gamma | PPARG |
| *Licorice* | Gancao | MOL004815 | Peroxisome proliferator activated receptor gamma | PPARG |
| *Licorice* | Gancao | MOL004820 | Peroxisome proliferator activated receptor gamma | PPARG |
| *Licorice* | Gancao | MOL004824 | Peroxisome proliferator activated receptor gamma | PPARG |
| *Licorice* | Gancao | MOL004827 | Peroxisome proliferator activated receptor gamma | PPARG |
| *Licorice* | Gancao | MOL004828 | Peroxisome proliferator activated receptor gamma | PPARG |
| *Licorice* | Gancao | MOL004833 | Peroxisome proliferator activated receptor gamma | PPARG |
| *Licorice* | Gancao | MOL004835 | Peroxisome proliferator activated receptor gamm | PPARG |
| *Licorice* | Gancao | MOL004841 | Peroxisome proliferator activated receptor gamma | PPARG |
| *Licorice* | Gancao | MOL004848 | Peroxisome proliferator activated receptor gamma | PPARG |
| *Licorice* | Gancao | MOL004849 | Peroxisome proliferator activated receptor gamma | PPARG |
| *Licorice* | Gancao | MOL004855 | Peroxisome proliferator activated receptor gamma | PPARG |
| *Licorice* | Gancao | MOL004856 | Peroxisome proliferator activated receptor gamma | PPARG |
| *Licorice* | Gancao | MOL004857 | Peroxisome proliferator activated receptor gamma | PPARG |
| *Licorice* | Gancao | MOL004863 | Peroxisome proliferator activated receptor gamma | PPARG |
| *Licorice* | Gancao | MOL004864 | Peroxisome proliferator activated receptor gamma | PPARG |
| *Licorice* | Gancao | MOL004866 | Peroxisome proliferator activated receptor gamma | PPARG |
| *Licorice* | Gancao | MOL004879 | Peroxisome proliferator activated receptor gamma | PPARG |
| *Licorice* | Gancao | MOL004883 | Peroxisome proliferator activated receptor gamma | PPARG |
| *Licorice* | Gancao | MOL004884 | Peroxisome proliferator activated receptor gamma | PPARG |
| *Licorice* | Gancao | MOL004885 | Peroxisome proliferator activated receptor gamma | PPARG |
| *Licorice* | Gancao | MOL004891 | Peroxisome proliferator activated receptor gamma | PPARG |
| *Licorice* | Gancao | MOL004898 | Peroxisome proliferator activated receptor gamma | PPARG |
| *Licorice* | Gancao | MOL004904 | Peroxisome proliferator activated receptor gamma | PPARG |
| *Licorice* | Gancao | MOL004907 | Peroxisome proliferator activated receptor gamma | PPARG |
| *Licorice* | Gancao | MOL004908 | Peroxisome proliferator activated receptor gamma | PPARG |
| *Licorice* | Gancao | MOL004911 | Peroxisome proliferator activated receptor gamma | PPARG |
| *Licorice* | Gancao | MOL004912 | Peroxisome proliferator activated receptor gamma | PPARG |
| *Licorice* | Gancao | MOL004913 | Peroxisome proliferator activated receptor gamma | PPARG |
| *Licorice* | Gancao | MOL004914 | Peroxisome proliferator activated receptor gamma | PPARG |
| *Licorice* | Gancao | MOL004915 | Peroxisome proliferator activated receptor gamma | PPARG |
| *Licorice* | Gancao | MOL004949 | Peroxisome proliferator activated receptor gamma | PPARG |
| *Licorice* | Gancao | MOL004957 | Peroxisome proliferator activated receptor gamma | PPARG |
| *Licorice* | Gancao | MOL004959 | Peroxisome proliferator activated receptor gamma | PPARG |
| *Licorice* | Gancao | MOL004961 | Peroxisome proliferator activated receptor gamma | PPARG |
| *Licorice* | Gancao | MOL004966 | Peroxisome proliferator activated receptor gamma | PPARG |
| *Licorice* | Gancao | MOL000497 | Peroxisome proliferator activated receptor gamma | PPARG |
| *Licorice* | Gancao | MOL004974 | Peroxisome proliferator activated receptor gamma | PPARG |
| *Licorice* | Gancao | MOL004978 | Peroxisome proliferator activated receptor gamma | PPARG |
| *Licorice* | Gancao | MOL004980 | Peroxisome proliferator activated receptor gamma | PPARG |
| *Licorice* | Gancao | MOL004990 | Peroxisome proliferator activated receptor gamma | PPARG |
| *Licorice* | Gancao | MOL004991 | Peroxisome proliferator activated receptor gamma | PPARG |
| *Licorice* | Gancao | MOL000500 | Peroxisome proliferator activated receptor gamma | PPARG |
| *Licorice* | Gancao | MOL005000 | Peroxisome proliferator activated receptor gamma | PPARG |
| *Licorice* | Gancao | MOL005003 | Peroxisome proliferator activated receptor gamma | PPARG |
| *Licorice* | Gancao | MOL005007 | Peroxisome proliferator activated receptor gamma | PPARG |
| *Licorice* | Gancao | MOL005012 | Peroxisome proliferator activated receptor gamma | PPARG |
| *Licorice* | Gancao | MOL005016 | Peroxisome proliferator activated receptor gamma | PPARG |
| *Licorice* | Gancao | MOL005017 | Peroxisome proliferator activated receptor gamma | PPARG |
| *Licorice* | Gancao | MOL005020 | Peroxisome proliferator activated receptor gamma | PPARG |
| *Licorice* | Gancao | MOL000098 | Peroxisome proliferator activated receptor gamma | PPARG |
| *Licorice* | Gancao | MOL000098 | Peroxisome proliferator-activated receptor gamma | PPARG |
| *Licorice* | Gancao | MOL000422 | Serine/threonine-protein phosphatase 2B catalytic subunit alpha isoform | PPP3CA |
| *Licorice* | Gancao | MOL000098 | Protein kinase C alpha type | PRKCA |
| *Licorice* | Gancao | MOL000098 | Protein kinase C beta type | PRKCB |
| *Licorice* | Gancao | MOL001484 | Trypsin-1 | PRSS1 |
| *Licorice* | Gancao | MOL000239 | Trypsin-1 | PRSS1 |
| *Licorice* | Gancao | MOL002565 | Trypsin-1 | PRSS1 |
| *Licorice* | Gancao | MOL000354 | Trypsin-1 | PRSS1 |
| *Licorice* | Gancao | MOL003656 | Trypsin-1 | PRSS1 |
| *Licorice* | Gancao | MOL003896 | Trypsin-1 | PRSS1 |
| *Licorice* | Gancao | MOL000392 | Trypsin-1 | PRSS1 |
| *Licorice* | Gancao | MOL000417 | Trypsin-1 | PRSS1 |
| *Licorice* | Gancao | MOL000422 | Trypsin-1 | PRSS1 |
| *Licorice* | Gancao | MOL004808 | Trypsin-1 | PRSS1 |
| *Licorice* | Gancao | MOL004810 | Trypsin-1 | PRSS1 |
| *Licorice* | Gancao | MOL004811 | Trypsin-1 | PRSS1 |
| *Licorice* | Gancao | MOL004820 | Trypsin-1 | PRSS1 |
| *Licorice* | Gancao | MOL004824 | Trypsin-1 | PRSS1 |
| *Licorice* | Gancao | MOL004827 | Trypsin-1 | PRSS1 |
| *Licorice* | Gancao | MOL004828 | Trypsin-1 | PRSS1 |
| *Licorice* | Gancao | MOL004833 | Trypsin-1 | PRSS1 |
| *Licorice* | Gancao | MOL004849 | Trypsin-1 | PRSS1 |
| *Licorice* | Gancao | MOL004855 | Trypsin-1 | PRSS1 |
| *Licorice* | Gancao | MOL004856 | Trypsin-1 | PRSS1 |
| *Licorice* | Gancao | MOL004857 | Trypsin-1 | PRSS1 |
| *Licorice* | Gancao | MOL004863 | Trypsin-1 | PRSS1 |
| *Licorice* | Gancao | MOL004864 | Trypsin-1 | PRSS1 |
| *Licorice* | Gancao | MOL004866 | Trypsin-1 | PRSS1 |
| *Licorice* | Gancao | MOL004879 | Trypsin-1 | PRSS1 |
| *Licorice* | Gancao | MOL004883 | Trypsin-1 | PRSS1 |
| *Licorice* | Gancao | MOL004884 | Trypsin-1 | PRSS1 |
| *Licorice* | Gancao | MOL004885 | Trypsin-1 | PRSS1 |
| *Licorice* | Gancao | MOL004891 | Trypsin-1 | PRSS1 |
| *Licorice* | Gancao | MOL004904 | Trypsin-1 | PRSS1 |
| *Licorice* | Gancao | MOL004907 | Trypsin-1 | PRSS1 |
| *Licorice* | Gancao | MOL004908 | Trypsin-1 | PRSS1 |
| *Licorice* | Gancao | MOL004911 | Trypsin-1 | PRSS1 |
| *Licorice* | Gancao | MOL004912 | Trypsin-1 | PRSS1 |
| *Licorice* | Gancao | MOL004915 | Trypsin-1 | PRSS1 |
| *Licorice* | Gancao | MOL004949 | Trypsin-1 | PRSS1 |
| *Licorice* | Gancao | MOL004957 | Trypsin-1 | PRSS1 |
| *Licorice* | Gancao | MOL004959 | Trypsin-1 | PRSS1 |
| *Licorice* | Gancao | MOL004961 | Trypsin-1 | PRSS1 |
| *Licorice* | Gancao | MOL004966 | Trypsin-1 | PRSS1 |
| *Licorice* | Gancao | MOL004974 | Trypsin-1 | PRSS1 |
| *Licorice* | Gancao | MOL004978 | Trypsin-1 | PRSS1 |
| *Licorice* | Gancao | MOL004980 | Trypsin-1 | PRSS1 |
| *Licorice* | Gancao | MOL004991 | Trypsin-1 | PRSS1 |
| *Licorice* | Gancao | MOL000500 | Trypsin-1 | PRSS1 |
| *Licorice* | Gancao | MOL005000 | Trypsin-1 | PRSS1 |
| *Licorice* | Gancao | MOL005001 | Trypsin-1 | PRSS1 |
| *Licorice* | Gancao | MOL005003 | Trypsin-1 | PRSS1 |
| *Licorice* | Gancao | MOL005007 | Trypsin-1 | PRSS1 |
| *Licorice* | Gancao | MOL005008 | Trypsin-1 | PRSS1 |
| *Licorice* | Gancao | MOL005012 | Trypsin-1 | PRSS1 |
| *Licorice* | Gancao | MOL005016 | Trypsin-1 | PRSS1 |
| *Licorice* | Gancao | MOL005020 | Trypsin-1 | PRSS1 |
| *Licorice* | Gancao | MOL000098 | Trypsin-1 | PRSS1 |
| *Licorice* | Gancao | MOL000422 | 26S proteasome non-ATPase regulatory subunit 3 | PSMD3 |
| *Licorice* | Gancao | MOL000098 | 26S proteasome non-ATPase regulatory subunit 3 | PSMD3 |
| *Licorice* | Gancao | MOL000098 | Phosphatidylinositol-3,4,5-trisphosphate 3-phosphatase and dual-specificity protein phosphatase PTEN | PTEN |
| *Licorice* | Gancao | MOL000098 | Prostaglandin E2 receptor EP3 subtype | PTGER3 |
| *Licorice* | Gancao | MOL001484 | Prostaglandin G/H synthase 1 | PTGS1 |
| *Licorice* | Gancao | MOL001792 | Prostaglandin G/H synthase 1 | PTGS1 |
| *Licorice* | Gancao | MOL000239 | Prostaglandin G/H synthase 1 | PTGS1 |
| *Licorice* | Gancao | MOL002565 | Prostaglandin G/H synthase 1 | PTGS1 |
| *Licorice* | Gancao | MOL000354 | Prostaglandin G/H synthase 1 | PTGS1 |
| *Licorice* | Gancao | MOL003896 | Prostaglandin G/H synthase 1 | PTGS1 |
| *Licorice* | Gancao | MOL000392 | Prostaglandin G/H synthase 1 | PTGS1 |
| *Licorice* | Gancao | MOL000417 | Prostaglandin G/H synthase 1 | PTGS1 |
| *Licorice* | Gancao | MOL000422 | Prostaglandin G/H synthase 1 | PTGS1 |
| *Licorice* | Gancao | MOL004328 | Prostaglandin G/H synthase 1 | PTGS1 |
| *Licorice* | Gancao | MOL004810 | Prostaglandin G/H synthase 1 | PTGS1 |
| *Licorice* | Gancao | MOL004815 | Prostaglandin G/H synthase 1 | PTGS1 |
| *Licorice* | Gancao | MOL004820 | Prostaglandin G/H synthase 1 | PTGS1 |
| *Licorice* | Gancao | MOL004828 | Prostaglandin G/H synthase 1 | PTGS1 |
| *Licorice* | Gancao | MOL004829 | Prostaglandin G/H synthase 1 | PTGS1 |
| *Licorice* | Gancao | MOL004835 | Prostaglandin G/H synthase 1 | PTGS1 |
| *Licorice* | Gancao | MOL004841 | Prostaglandin G/H synthase 1 | PTGS1 |
| *Licorice* | Gancao | MOL004885 | Prostaglandin G/H synthase 1 | PTGS1 |
| *Licorice* | Gancao | MOL004891 | Prostaglandin G/H synthase 1 | PTGS1 |
| *Licorice* | Gancao | MOL004907 | Prostaglandin G/H synthase 1 | PTGS1 |
| *Licorice* | Gancao | MOL004910 | Prostaglandin G/H synthase 1 | PTGS1 |
| *Licorice* | Gancao | MOL004911 | Prostaglandin G/H synthase 1 | PTGS1 |
| *Licorice* | Gancao | MOL004912 | Prostaglandin G/H synthase 1 | PTGS1 |
| *Licorice* | Gancao | MOL004941 | Prostaglandin G/H synthase 1 | PTGS1 |
| *Licorice* | Gancao | MOL004945 | Prostaglandin G/H synthase 1 | PTGS1 |
| *Licorice* | Gancao | MOL004957 | Prostaglandin G/H synthase 1 | PTGS1 |
| *Licorice* | Gancao | MOL004959 | Prostaglandin G/H synthase 1 | PTGS1 |
| *Licorice* | Gancao | MOL004961 | Prostaglandin G/H synthase 1 | PTGS1 |
| *Licorice* | Gancao | MOL004966 | Prostaglandin G/H synthase 1 | PTGS1 |
| *Licorice* | Gancao | MOL000497 | Prostaglandin G/H synthase 1 | PTGS1 |
| *Licorice* | Gancao | MOL004974 | Prostaglandin G/H synthase 1 | PTGS1 |
| *Licorice* | Gancao | MOL004978 | Prostaglandin G/H synthase 1 | PTGS1 |
| *Licorice* | Gancao | MOL004980 | Prostaglandin G/H synthase 1 | PTGS1 |
| *Licorice* | Gancao | MOL004990 | Prostaglandin G/H synthase 1 | PTGS1 |
| *Licorice* | Gancao | MOL004991 | Prostaglandin G/H synthase 1 | PTGS1 |
| *Licorice* | Gancao | MOL000500 | Prostaglandin G/H synthase 1 | PTGS1 |
| *Licorice* | Gancao | MOL005003 | Prostaglandin G/H synthase 1 | PTGS1 |
| *Licorice* | Gancao | MOL005007 | Prostaglandin G/H synthase 1 | PTGS1 |
| *Licorice* | Gancao | MOL005016 | Prostaglandin G/H synthase 1 | PTGS1 |
| *Licorice* | Gancao | MOL000098 | Prostaglandin G/H synthase 1 | PTGS1 |
| *Licorice* | Gancao | MOL001484 | Prostaglandin G/H synthase 2 | PTGS2 |
| *Licorice* | Gancao | MOL001792 | Prostaglandin G/H synthase 2 | PTGS2 |
| *Licorice* | Gancao | MOL002311 | Prostaglandin G/H synthase 2 | PTGS2 |
| *Licorice* | Gancao | MOL000239 | Prostaglandin G/H synthase 2 | PTGS2 |
| *Licorice* | Gancao | MOL002565 | Prostaglandin G/H synthase 2 | PTGS2 |
| *Licorice* | Gancao | MOL000354 | Prostaglandin G/H synthase 2 | PTGS2 |
| *Licorice* | Gancao | MOL003656 | Prostaglandin G/H synthase 2 | PTGS2 |
| *Licorice* | Gancao | MOL003896 | Prostaglandin G/H synthase 2 | PTGS2 |
| *Licorice* | Gancao | MOL000392 | Prostaglandin G/H synthase 2 | PTGS2 |
| *Licorice* | Gancao | MOL000417 | Prostaglandin G/H synthase 2 | PTGS2 |
| *Licorice* | Gancao | MOL000422 | Prostaglandin G/H synthase 2 | PTGS2 |
| *Licorice* | Gancao | MOL004328 | Prostaglandin G/H synthase 2 | PTGS2 |
| *Licorice* | Gancao | MOL004805 | Prostaglandin G/H synthase 2 | PTGS2 |
| *Licorice* | Gancao | MOL004806 | Prostaglandin G/H synthase 2 | PTGS2 |
| *Licorice* | Gancao | MOL004808 | Prostaglandin G/H synthase 2 | PTGS2 |
| *Licorice* | Gancao | MOL004810 | Prostaglandin G/H synthase 2 | PTGS2 |
| *Licorice* | Gancao | MOL004811 | Prostaglandin G/H synthase 2 | PTGS2 |
| *Licorice* | Gancao | MOL004814 | Prostaglandin G/H synthase 2 | PTGS2 |
| *Licorice* | Gancao | MOL004815 | Prostaglandin G/H synthase 2 | PTGS2 |
| *Licorice* | Gancao | MOL004820 | Prostaglandin G/H synthase 2 | PTGS2 |
| *Licorice* | Gancao | MOL004824 | Prostaglandin G/H synthase 2 | PTGS2 |
| *Licorice* | Gancao | MOL004827 | Prostaglandin G/H synthase 2 | PTGS2 |
| *Licorice* | Gancao | MOL004828 | Prostaglandin G/H synthase 2 | PTGS2 |
| *Licorice* | Gancao | MOL004829 | Prostaglandin G/H synthase 2 | PTGS2 |
| *Licorice* | Gancao | MOL004833 | Prostaglandin G/H synthase 2 | PTGS2 |
| *Licorice* | Gancao | MOL004835 | Prostaglandin G/H synthase 2 | PTGS2 |
| *Licorice* | Gancao | MOL004838 | Prostaglandin G/H synthase 2 | PTGS2 |
| *Licorice* | Gancao | MOL004841 | Prostaglandin G/H synthase 2 | PTGS2 |
| *Licorice* | Gancao | MOL004848 | Prostaglandin G/H synthase 2 | PTGS2 |
| *Licorice* | Gancao | MOL004849 | Prostaglandin G/H synthase 2 | PTGS2 |
| *Licorice* | Gancao | MOL004855 | Prostaglandin G/H synthase 2 | PTGS2 |
| *Licorice* | Gancao | MOL004856 | Prostaglandin G/H synthase 2 | PTGS2 |
| *Licorice* | Gancao | MOL004857 | Prostaglandin G/H synthase 2 | PTGS2 |
| *Licorice* | Gancao | MOL004863 | Prostaglandin G/H synthase 2 | PTGS2 |
| *Licorice* | Gancao | MOL004864 | Prostaglandin G/H synthase 2 | PTGS2 |
| *Licorice* | Gancao | MOL004866 | Prostaglandin G/H synthase 2 | PTGS2 |
| *Licorice* | Gancao | MOL004879 | Prostaglandin G/H synthase 2 | PTGS2 |
| *Licorice* | Gancao | MOL004883 | Prostaglandin G/H synthase 2 | PTGS2 |
| *Licorice* | Gancao | MOL004884 | Prostaglandin G/H synthase 2 | PTGS2 |
| *Licorice* | Gancao | MOL004885 | Prostaglandin G/H synthase 2 | PTGS2 |
| *Licorice* | Gancao | MOL004891 | Prostaglandin G/H synthase 2 | PTGS2 |
| *Licorice* | Gancao | MOL004898 | Prostaglandin G/H synthase 2 | PTGS2 |
| *Licorice* | Gancao | MOL004903 | Prostaglandin G/H synthase 2 | PTGS2 |
| *Licorice* | Gancao | MOL004904 | Prostaglandin G/H synthase 2 | PTGS2 |
| *Licorice* | Gancao | MOL004907 | Prostaglandin G/H synthase 2 | PTGS2 |
| *Licorice* | Gancao | MOL004908 | Prostaglandin G/H synthase 2 | PTGS2 |
| *Licorice* | Gancao | MOL004910 | Prostaglandin G/H synthase 2 | PTGS2 |
| *Licorice* | Gancao | MOL004911 | Prostaglandin G/H synthase 2 | PTGS2 |
| *Licorice* | Gancao | MOL004912 | Prostaglandin G/H synthase 2 | PTGS2 |
| *Licorice* | Gancao | MOL004915 | Prostaglandin G/H synthase 2 | PTGS2 |
| *Licorice* | Gancao | MOL004924 | Prostaglandin G/H synthase 2 | PTGS2 |
| *Licorice* | Gancao | MOL004935 | Prostaglandin G/H synthase 2 | PTGS2 |
| *Licorice* | Gancao | MOL004941 | Prostaglandin G/H synthase 2 | PTGS2 |
| *Licorice* | Gancao | MOL004945 | Prostaglandin G/H synthase 2 | PTGS2 |
| *Licorice* | Gancao | MOL004948 | Prostaglandin G/H synthase 2 | PTGS2 |
| *Licorice* | Gancao | MOL004949 | Prostaglandin G/H synthase 2 | PTGS2 |
| *Licorice* | Gancao | MOL004957 | Prostaglandin G/H synthase 2 | PTGS2 |
| *Licorice* | Gancao | MOL004959 | Prostaglandin G/H synthase 2 | PTGS2 |
| *Licorice* | Gancao | MOL004961 | Prostaglandin G/H synthase 2 | PTGS2 |
| *Licorice* | Gancao | MOL004966 | Prostaglandin G/H synthase 2 | PTGS2 |
| *Licorice* | Gancao | MOL000497 | Prostaglandin G/H synthase 2 | PTGS2 |
| *Licorice* | Gancao | MOL004974 | Prostaglandin G/H synthase 2 | PTGS2 |
| *Licorice* | Gancao | MOL004978 | Prostaglandin G/H synthase 2 | PTGS2 |
| *Licorice* | Gancao | MOL004980 | Prostaglandin G/H synthase 2 | PTGS2 |
| *Licorice* | Gancao | MOL004988 | Prostaglandin G/H synthase 2 | PTGS2 |
| *Licorice* | Gancao | MOL004989 | Prostaglandin G/H synthase 2 | PTGS2 |
| *Licorice* | Gancao | MOL004990 | Prostaglandin G/H synthase 2 | PTGS2 |
| *Licorice* | Gancao | MOL004991 | Prostaglandin G/H synthase 2 | PTGS2 |
| *Licorice* | Gancao | MOL004993 | Prostaglandin G/H synthase 2 | PTGS2 |
| *Licorice* | Gancao | MOL000500 | Prostaglandin G/H synthase 2 | PTGS2 |
| *Licorice* | Gancao | MOL005000 | Prostaglandin G/H synthase 2 | PTGS2 |
| *Licorice* | Gancao | MOL005001 | Prostaglandin G/H synthase 2 | PTGS2 |
| *Licorice* | Gancao | MOL005003 | Prostaglandin G/H synthase 2 | PTGS2 |
| *Licorice* | Gancao | MOL005007 | Prostaglandin G/H synthase 2 | PTGS2 |
| *Licorice* | Gancao | MOL005008 | Prostaglandin G/H synthase 2 | PTGS2 |
| *Licorice* | Gancao | MOL005012 | Prostaglandin G/H synthase 2 | PTGS2 |
| *Licorice* | Gancao | MOL005016 | Prostaglandin G/H synthase 2 | PTGS2 |
| *Licorice* | Gancao | MOL005018 | Prostaglandin G/H synthase 2 | PTGS2 |
| *Licorice* | Gancao | MOL005020 | Prostaglandin G/H synthase 2 | PTGS2 |
| *Licorice* | Gancao | MOL000098 | Prostaglandin G/H synthase 2 | PTGS2 |
| *Licorice* | Gancao | MOL000354 | mRNA of Protein-tyrosine phosphatase, non-receptor type 1 | PTPN1 |
| *Licorice* | Gancao | MOL004863 | mRNA of Protein-tyrosine phosphatase, non-receptor type 1 | PTPN1 |
| *Licorice* | Gancao | MOL004961 | mRNA of Protein-tyrosine phosphatase, non-receptor type 1 | PTPN1 |
| *Licorice* | Gancao | MOL000354 | Glycogen phosphorylase, muscle form | PYGM |
| *Licorice* | Gancao | MOL000098 | RAF proto-oncogene serine/threonine-protein kinase | RAF1 |
| *Licorice* | Gancao | MOL000098 | Ras GTPase-activating protein 1 | RASA1 |
| *Licorice* | Gancao | MOL000098 | Ras association domain-containing protein 1 | RASSF1 |
| *Licorice* | Gancao | MOL000497 | Retinoblastoma-associated protein | RB1 |
| *Licorice* | Gancao | MOL000098 | Retinoblastoma-associated protein | RB1 |
| *Licorice* | Gancao | MOL000354 | Transcription factor p65 | RELA |
| *Licorice* | Gancao | MOL000422 | Transcription factor p65 | RELA |
| *Licorice* | Gancao | MOL004328 | Transcription factor p65 | RELA |
| *Licorice* | Gancao | MOL000497 | Transcription factor p65 | RELA |
| *Licorice* | Gancao | MOL000098 | Transcription factor p65 | RELA |
| *Licorice* | Gancao | MOL000098 | Protein CBFA2T1 | RUNX1T1 |
| *Licorice* | Gancao | MOL000098 | Runt-related transcription factor 2 | RUNX2 |
| *Licorice* | Gancao | MOL001484 | Retinoic acid receptor RXR-alpha | RXRA |
| *Licorice* | Gancao | MOL001792 | Retinoic acid receptor RXR-alpha | RXRA |
| *Licorice* | Gancao | MOL002565 | Retinoic acid receptor RXR-alpha | RXRA |
| *Licorice* | Gancao | MOL003896 | Retinoic acid receptor RXR-alpha | RXRA |
| *Licorice* | Gancao | MOL000392 | Retinoic acid receptor RXR-alpha | RXRA |
| *Licorice* | Gancao | MOL000417 | Retinoic acid receptor RXR-alpha | RXRA |
| *Licorice* | Gancao | MOL004811 | Retinoic acid receptor RXR-alpha | RXRA |
| *Licorice* | Gancao | MOL004815 | Retinoic acid receptor RXR-alpha | RXRA |
| *Licorice* | Gancao | MOL004820 | Retinoic acid receptor RXR-alpha | RXRA |
| *Licorice* | Gancao | MOL004828 | Retinoic acid receptor RXR-alpha | RXRA |
| *Licorice* | Gancao | MOL004829 | Retinoic acid receptor RXR-alpha | RXRA |
| *Licorice* | Gancao | MOL004833 | Retinoic acid receptor RXR-alpha | RXRA |
| *Licorice* | Gancao | MOL004838 | Retinoic acid receptor RXR-alpha | RXRA |
| *Licorice* | Gancao | MOL004891 | Retinoic acid receptor RXR-alpha | RXRA |
| *Licorice* | Gancao | MOL004908 | Retinoic acid receptor RXR-alpha | RXRA |
| *Licorice* | Gancao | MOL004911 | Retinoic acid receptor RXR-alpha | RXRA |
| *Licorice* | Gancao | MOL004912 | Retinoic acid receptor RXR-alpha | RXRA |
| *Licorice* | Gancao | MOL004941 | Retinoic acid receptor RXR-alpha | RXRA |
| *Licorice* | Gancao | MOL004957 | Retinoic acid receptor RXR-alpha | RXRA |
| *Licorice* | Gancao | MOL004959 | Retinoic acid receptor RXR-alpha | RXRA |
| *Licorice* | Gancao | MOL004974 | Retinoic acid receptor RXR-alpha | RXRA |
| *Licorice* | Gancao | MOL004978 | Retinoic acid receptor RXR-alpha | RXRA |
| *Licorice* | Gancao | MOL004991 | Retinoic acid receptor RXR-alpha | RXRA |
| *Licorice* | Gancao | MOL000500 | Retinoic acid receptor RXR-alpha | RXRA |
| *Licorice* | Gancao | MOL005003 | Retinoic acid receptor RXR-alpha | RXRA |
| *Licorice* | Gancao | MOL005016 | Retinoic acid receptor RXR-alpha | RXRA |
| *Licorice* | Gancao | MOL000098 | Retinoic acid receptor RXR-alpha | RXRA |
| *Licorice* | Gancao | MOL004891 | Retinoic acid receptor RXR-beta | RXRB |
| *Licorice* | Gancao | MOL004908 | Retinoic acid receptor RXR-beta | RXRB |
| *Licorice* | Gancao | MOL004978 | Retinoic acid receptor RXR-beta | RXRB |
| *Licorice* | Gancao | MOL005003 | Retinoic acid receptor RXR-beta | RXRB |
| *Licorice* | Gancao | MOL001484 | Sodium channel protein type 5 subunit alpha | SCN5A |
| *Licorice* | Gancao | MOL000239 | Sodium channel protein type 5 subunit alpha | SCN5A |
| *Licorice* | Gancao | MOL002565 | Sodium channel protein type 5 subunit alpha | SCN5A |
| *Licorice* | Gancao | MOL003656 | Sodium channel protein type 5 subunit alpha | SCN5A |
| *Licorice* | Gancao | MOL003896 | Sodium channel protein type 5 subunit alpha | SCN5A |
| *Licorice* | Gancao | MOL004806 | Sodium channel protein type 5 subunit alpha | SCN5A |
| *Licorice* | Gancao | MOL004810 | Sodium channel protein type 5 subunit alpha | SCN5A |
| *Licorice* | Gancao | MOL004811 | Sodium channel protein type 5 subunit alpha | SCN5A |
| *Licorice* | Gancao | MOL004815 | Sodium channel protein type 5 subunit alpha | SCN5A |
| *Licorice* | Gancao | MOL004820 | Sodium channel protein type 5 subunit alpha | SCN5A |
| *Licorice* | Gancao | MOL004827 | Sodium channel protein type 5 subunit alpha | SCN5A |
| *Licorice* | Gancao | MOL004828 | Sodium channel protein type 5 subunit alpha | SCN5A |
| *Licorice* | Gancao | MOL004829 | Sodium channel protein type 5 subunit alpha | SCN5A |
| *Licorice* | Gancao | MOL004833 | Sodium channel protein type 5 subunit alpha | SCN5A |
| *Licorice* | Gancao | MOL004835 | Sodium channel protein type 5 subunit alpha | SCN5A |
| *Licorice* | Gancao | MOL004856 | Sodium channel protein type 5 subunit alpha | SCN5A |
| *Licorice* | Gancao | MOL004866 | Sodium channel protein type 5 subunit alpha | SCN5A |
| *Licorice* | Gancao | MOL004885 | Sodium channel protein type 5 subunit alpha | SCN5A |
| *Licorice* | Gancao | MOL004891 | Sodium channel protein type 5 subunit alpha | SCN5A |
| *Licorice* | Gancao | MOL004908 | Sodium channel protein type 5 subunit alpha | SCN5A |
| *Licorice* | Gancao | MOL004910 | Sodium channel protein type 5 subunit alpha | SCN5A |
| *Licorice* | Gancao | MOL004911 | Sodium channel protein type 5 subunit alpha | SCN5A |
| *Licorice* | Gancao | MOL004912 | Sodium channel protein type 5 subunit alpha | SCN5A |
| *Licorice* | Gancao | MOL004915 | Sodium channel protein type 5 subunit alpha | SCN5A |
| *Licorice* | Gancao | MOL004945 | Sodium channel protein type 5 subunit alpha | SCN5A |
| *Licorice* | Gancao | MOL004957 | Sodium channel protein type 5 subunit alpha | SCN5A |
| *Licorice* | Gancao | MOL004959 | Sodium channel protein type 5 subunit alpha | SCN5A |
| *Licorice* | Gancao | MOL004961 | Sodium channel protein type 5 subunit alpha | SCN5A |
| *Licorice* | Gancao | MOL004966 | Sodium channel protein type 5 subunit alpha | SCN5A |
| *Licorice* | Gancao | MOL000497 | Sodium channel protein type 5 subunit alpha | SCN5A |
| *Licorice* | Gancao | MOL004974 | Sodium channel protein type 5 subunit alpha | SCN5A |
| *Licorice* | Gancao | MOL004978 | Sodium channel protein type 5 subunit alpha | SCN5A |
| *Licorice* | Gancao | MOL004980 | Sodium channel protein type 5 subunit alpha | SCN5A |
| *Licorice* | Gancao | MOL004989 | Sodium channel protein type 5 subunit alpha | SCN5A |
| *Licorice* | Gancao | MOL004991 | Sodium channel protein type 5 subunit alpha | SCN5A |
| *Licorice* | Gancao | MOL004993 | Sodium channel protein type 5 subunit alpha | SCN5A |
| *Licorice* | Gancao | MOL000500 | Sodium channel protein type 5 subunit alpha | SCN5A |
| *Licorice* | Gancao | MOL005003 | Sodium channel protein type 5 subunit alpha | SCN5A |
| *Licorice* | Gancao | MOL005007 | Sodium channel protein type 5 subunit alpha | SCN5A |
| *Licorice* | Gancao | MOL005012 | Sodium channel protein type 5 subunit alpha | SCN5A |
| *Licorice* | Gancao | MOL005016 | Sodium channel protein type 5 subunit alpha | SCN5A |
| *Licorice* | Gancao | MOL005020 | Sodium channel protein type 5 subunit alpha | SCN5A |
| *Licorice* | Gancao | MOL000098 | Sodium channel protein type 5 subunit alpha | SCN5A |
| *Licorice* | Gancao | MOL000422 | E-selectin | SELE |
| *Licorice* | Gancao | MOL000098 | E-selectin | SELE |
| *Licorice* | Gancao | MOL000098 | Plasminogen activator inhibitor 1 | SERPINE1 |
| *Licorice* | Gancao | MOL000392 | NAD-dependent deacetylase sirtuin-1 | SIRT1 |
| *Licorice* | Gancao | MOL000422 | Solute carrier family 2, facilitated glucose transporter member 4 | SLC2A4 |
| *Licorice* | Gancao | MOL000098 | Solute carrier family 2, facilitated glucose transporter member 4 | SLC2A4 |
| *Licorice* | Gancao | MOL000422 | Sodium-dependent noradrenaline transporter | SLC6A2 |
| *Licorice* | Gancao | MOL002565 | Sodium-dependent dopamine transporter | SLC6A3 |
| *Licorice* | Gancao | MOL003896 | Sodium-dependent dopamine transporter | SLC6A3 |
| *Licorice* | Gancao | MOL000392 | Sodium-dependent dopamine transporter | SLC6A3 |
| *Licorice* | Gancao | MOL004835 | Sodium-dependent dopamine transporter | SLC6A3 |
| *Licorice* | Gancao | MOL004957 | Sodium-dependent dopamine transporter | SLC6A3 |
| *Licorice* | Gancao | MOL000497 | Sodium-dependent dopamine transporter | SLC6A3 |
| *Licorice* | Gancao | MOL004978 | Sodium-dependent dopamine transporter | SLC6A3 |
| *Licorice* | Gancao | MOL000500 | Sodium-dependent dopamine transporter | SLC6A3 |
| *Licorice* | Gancao | MOL001792 | Sodium-dependent serotonin transporter | SLC6A4 |
| *Licorice* | Gancao | MOL002565 | Sodium-dependent serotonin transporter | SLC6A4 |
| *Licorice* | Gancao | MOL003896 | Sodium-dependent serotonin transporter | SLC6A4 |
| *Licorice* | Gancao | MOL000392 | Sodium-dependent serotonin transporter | SLC6A4 |
| *Licorice* | Gancao | MOL004835 | Sodium-dependent serotonin transporter | SLC6A4 |
| *Licorice* | Gancao | MOL004941 | Sodium-dependent serotonin transporter | SLC6A4 |
| *Licorice* | Gancao | MOL004957 | Sodium-dependent serotonin transporter | SLC6A4 |
| *Licorice* | Gancao | MOL000500 | Sodium-dependent serotonin transporter | SLC6A4 |
| *Licorice* | Gancao | MOL000422 | Antileukoproteinase | SLPI |
| *Licorice* | Gancao | MOL004328 | Sterol O-acyltransferase 1 | SOAT1 |
| *Licorice* | Gancao | MOL004328 | Sterol O-acyltransferase 2 | SOAT2 |
| *Licorice* | Gancao | MOL004328 | Superoxide dismutase [Cu-Zn] | SOD1 |
| *Licorice* | Gancao | MOL004903 | Superoxide dismutase [Cu-Zn] | SOD1 |
| *Licorice* | Gancao | MOL000098 | Superoxide dismutase [Cu-Zn] | SOD1 |
| *Licorice* | Gancao | MOL000098 | Osteopontin | SPP1 |
| *Licorice* | Gancao | MOL004328 | Sterol regulatory element-binding protein 1 | SREBF1 |
| *Licorice* | Gancao | MOL000422 | Signal transducer and activator of transcription 1-alpha/beta | STAT1 |
| *Licorice* | Gancao | MOL000098 | Signal transducer and activator of transcription 1-alpha/beta | STAT1 |
| *Licorice* | Gancao | MOL000497 | Signal transducer and activator of transcription 3 | STAT3 |
| *Licorice* | Gancao | MOL000098 | Estrogen sulfotransferase | SULT1E1 |
| *Licorice* | Gancao | MOL000098 | Transforming growth factor beta-1 | TGFB1 |
| *Licorice* | Gancao | MOL000098 | Thrombomodulin | THBD |
| *Licorice* | Gancao | MOL000422 | Tumor necrosis factor | TNF |
| *Licorice* | Gancao | MOL000098 | Tumor necrosis factor | TNF |
| *Licorice* | Gancao | MOL000098 | DNA topoisomerase 1 | TOP1 |
| *Licorice* | Gancao | MOL000098 | DNA topoisomerase 2-alpha | TOP2A |
| *Licorice* | Gancao | MOL003656 | DNA topoisomerase II | TOP2B |
| *Licorice* | Gancao | MOL000422 | DNA topoisomerase II | TOP2B |
| *Licorice* | Gancao | MOL004808 | DNA topoisomerase II | TOP2B |
| *Licorice* | Gancao | MOL004810 | DNA topoisomerase II | TOP2B |
| *Licorice* | Gancao | MOL004811 | DNA topoisomerase II | TOP2B |
| *Licorice* | Gancao | MOL004820 | DNA topoisomerase II | TOP2B |
| *Licorice* | Gancao | MOL004824 | DNA topoisomerase II | TOP2B |
| *Licorice* | Gancao | MOL004827 | DNA topoisomerase II | TOP2B |
| *Licorice* | Gancao | MOL004828 | DNA topoisomerase II | TOP2B |
| *Licorice* | Gancao | MOL004829 | DNA topoisomerase II | TOP2B |
| *Licorice* | Gancao | MOL004849 | DNA topoisomerase II | TOP2B |
| *Licorice* | Gancao | MOL004855 | DNA topoisomerase II | TOP2B |
| *Licorice* | Gancao | MOL004856 | DNA topoisomerase II | TOP2B |
| *Licorice* | Gancao | MOL004857 | DNA topoisomerase II | TOP2B |
| *Licorice* | Gancao | MOL004864 | DNA topoisomerase II | TOP2B |
| *Licorice* | Gancao | MOL004879 | DNA topoisomerase II | TOP2B |
| *Licorice* | Gancao | MOL004883 | DNA topoisomerase II | TOP2B |
| *Licorice* | Gancao | MOL004884 | DNA topoisomerase II | TOP2B |
| *Licorice* | Gancao | MOL004885 | DNA topoisomerase II | TOP2B |
| *Licorice* | Gancao | MOL004904 | DNA topoisomerase II | TOP2B |
| *Licorice* | Gancao | MOL004959 | DNA topoisomerase II | TOP2B |
| *Licorice* | Gancao | MOL004966 | DNA topoisomerase II | TOP2B |
| *Licorice* | Gancao | MOL004974 | DNA topoisomerase II | TOP2B |
| *Licorice* | Gancao | MOL005000 | DNA topoisomerase II | TOP2B |
| *Licorice* | Gancao | MOL005001 | DNA topoisomerase II | TOP2B |
| *Licorice* | Gancao | MOL005007 | DNA topoisomerase II | TOP2B |
| *Licorice* | Gancao | MOL005008 | DNA topoisomerase II | TOP2B |
| *Licorice* | Gancao | MOL000098 | DNA topoisomerase II | TOP2B |
| *Licorice* | Gancao | MOL000098 | Cellular tumor antigen p53 | TP53 |
| *Licorice* | Gancao | MOL004328 | UDP-glucuronosyltransferase 1-1 | UGT1A1 |
| *Licorice* | Gancao | MOL000422 | Vascular cell adhesion protein 1 | VCAM1 |
| *Licorice* | Gancao | MOL000098 | Vascular cell adhesion protein 1 | VCAM1 |
| *Licorice* | Gancao | MOL000098 | Vascular endothelial growth factor A | VEGFA |
| *Licorice* | Gancao | MOL000354 | Xanthine dehydrogenase/oxidase | XDH |
| *Licorice* | Gancao | MOL000422 | Xanthine dehydrogenase/oxidase | XDH |
| *Licorice* | Gancao | MOL000098 | Xanthine dehydrogenase/oxidase | XDH |
| *Isatidis Radix* | Banlangen | MOL001735 | Acetylcholinesterase | ACHE |
| *Isatidis Radix* | Banlangen | MOL001779 | Acetylcholinesterase | ACHE |
| *Isatidis Radix* | Banlangen | MOL001803 | Acetylcholinesterase | ACHE |
| *Isatidis Radix* | Banlangen | MOL000449 | Alcohol dehydrogenase 1C | ADH1C |
| *Isatidis Radix* | Banlangen | MOL000358 | Alpha-1A adrenergic receptor | ADRA1A |
| *Isatidis Radix* | Banlangen | MOL000449 | Alpha-1A adrenergic receptor | ADRA1A |
| *Isatidis Radix* | Banlangen | MOL001721 | Alpha-1B adrenergic receptor | ADRA1B |
| *Isatidis Radix* | Banlangen | MOL001779 | Alpha-1B adrenergic receptor | ADRA1B |
| *Isatidis Radix* | Banlangen | MOL001803 | Alpha-1B adrenergic receptor | ADRA1B |
| *Isatidis Radix* | Banlangen | MOL000358 | Alpha-1B adrenergic receptor | ADRA1B |
| *Isatidis Radix* | Banlangen | MOL000449 | Alpha-1B adrenergic receptor | ADRA1B |
| *Isatidis Radix* | Banlangen | MOL000449 | Alpha-2A adrenergic receptor | ADRA2A |
| *Isatidis Radix* | Banlangen | MOL000449 | Beta-1 adrenergic receptor | ADRB1 |
| *Isatidis Radix* | Banlangen | MOL001689 | Beta-2 adrenergic receptor | ADRB2 |
| *Isatidis Radix* | Banlangen | MOL001749 | Beta-2 adrenergic receptor | ADRB2 |
| *Isatidis Radix* | Banlangen | MOL001792 | Beta-2 adrenergic receptor | ADRB2 |
| *Isatidis Radix* | Banlangen | MOL001803 | Beta-2 adrenergic receptor | ADRB2 |
| *Isatidis Radix* | Banlangen | MOL001820 | Beta-2 adrenergic receptor | ADRB2 |
| *Isatidis Radix* | Banlangen | MOL000358 | Beta-2 adrenergic receptor | ADRB2 |
| *Isatidis Radix* | Banlangen | MOL000449 | Beta-2 adrenergic receptor | ADRB2 |
| *Isatidis Radix* | Banlangen | MOL000449 | Aldose reductase | AKR1B1 |
| *Isatidis Radix* | Banlangen | MOL001689 | Androgen receptor | AR |
| *Isatidis Radix* | Banlangen | MOL002322 | Androgen receptor | AR |
| *Isatidis Radix* | Banlangen | MOL001767 | Androgen receptor | AR |
| *Isatidis Radix* | Banlangen | MOL001779 | Androgen receptor | AR |
| *Isatidis Radix* | Banlangen | MOL001782 | Androgen receptor | AR |
| *Isatidis Radix* | Banlangen | MOL001793 | Androgen receptor | AR |
| *Isatidis Radix* | Banlangen | MOL001803 | Androgen receptor | AR |
| *Isatidis Radix* | Banlangen | MOL001833 | Androgen receptor | AR |
| *Isatidis Radix* | Banlangen | MOL001833 | Beta-lactamase | BACE1 |
| *Isatidis Radix* | Banlangen | MOL001689 | Apoptosis regulator BAX | BAX |
| *Isatidis Radix* | Banlangen | MOL000358 | Apoptosis regulator BAX | BAX |
| *Isatidis Radix* | Banlangen | MOL001689 | Apoptosis regulator Bcl-2 | BCL2 |
| *Isatidis Radix* | Banlangen | MOL000358 | Apoptosis regulator Bcl-2 | BCL2 |
| *Isatidis Radix* | Banlangen | MOL001722 | Carbonic anhydrase II | CA2 |
| *Isatidis Radix* | Banlangen | MOL001750 | Carbonic anhydrase II | CA2 |
| *Isatidis Radix* | Banlangen | MOL001689 | Calmodulin | CALM1 |
| *Isatidis Radix* | Banlangen | MOL001733 | Calmodulin | CALM1 |
| *Isatidis Radix* | Banlangen | MOL001735 | Calmodulin | CALM1 |
| *Isatidis Radix* | Banlangen | MOL001779 | Calmodulin | CALM1 |
| *Isatidis Radix* | Banlangen | MOL001798 | Calmodulin | CALM1 |
| *Isatidis Radix* | Banlangen | MOL001803 | Calmodulin | CALM1 |
| *Isatidis Radix* | Banlangen | MOL001689 | Caspase-3 | CASP3 |
| *Isatidis Radix* | Banlangen | MOL000358 | Caspase-3 | CASP3 |
| *Isatidis Radix* | Banlangen | MOL001689 | Caspase-8 | CASP8 |
| *Isatidis Radix* | Banlangen | MOL000358 | Caspase-8 | CASP8 |
| *Isatidis Radix* | Banlangen | MOL000358 | Caspase-9 | CASP9 |
| *Isatidis Radix* | Banlangen | MOL001767 | Cyclin-A2 | CCNA2 |
| *Isatidis Radix* | Banlangen | MOL001781 | Cyclin-A2 | CCNA2 |
| *Isatidis Radix* | Banlangen | MOL001782 | Cyclin-A2 | CCNA2 |
| *Isatidis Radix* | Banlangen | MOL001793 | Cyclin-A2 | CCNA2 |
| *Isatidis Radix* | Banlangen | MOL001833 | Cyclin-A2 | CCNA2 |
| *Isatidis Radix* | Banlangen | MOL001689 | Cell division protein kinase 2 | CDK2 |
| *Isatidis Radix* | Banlangen | MOL001767 | Cell division protein kinase 2 | CDK2 |
| *Isatidis Radix* | Banlangen | MOL001781 | Cell division protein kinase 2 | CDK2 |
| *Isatidis Radix* | Banlangen | MOL001782 | Cell division protein kinase 2 | CDK2 |
| *Isatidis Radix* | Banlangen | MOL001793 | Cell division protein kinase 2 | CDK2 |
| *Isatidis Radix* | Banlangen | MOL001833 | Cell division protein kinase 2 | CDK2 |
| *Isatidis Radix* | Banlangen | MOL001689 | Cyclin-dependent kinase inhibitor 1 | CDKN1A |
| *Isatidis Radix* | Banlangen | MOL001689 | Serine/threonine-protein kinase Chk1 | CHEK1 |
| *Isatidis Radix* | Banlangen | MOL001782 | Serine/threonine-protein kinase Chk1 | CHEK1 |
| *Isatidis Radix* | Banlangen | MOL001793 | Serine/threonine-protein kinase Chk1 | CHEK1 |
| *Isatidis Radix* | Banlangen | MOL001803 | Serine/threonine-protein kinase Chk1 | CHEK1 |
| *Isatidis Radix* | Banlangen | MOL001749 | Muscarinic acetylcholine receptor M1 | CHRM1 |
| *Isatidis Radix* | Banlangen | MOL001779 | Muscarinic acetylcholine receptor M1 | CHRM1 |
| *Isatidis Radix* | Banlangen | MOL001820 | Muscarinic acetylcholine receptor M1 | CHRM1 |
| *Isatidis Radix* | Banlangen | MOL000358 | Muscarinic acetylcholine receptor M1 | CHRM1 |
| *Isatidis Radix* | Banlangen | MOL000449 | Muscarinic acetylcholine receptor M1 | CHRM1 |
| *Isatidis Radix* | Banlangen | MOL000358 | Muscarinic acetylcholine receptor M2 | CHRM2 |
| *Isatidis Radix* | Banlangen | MOL000449 | Muscarinic acetylcholine receptor M2 | CHRM2 |
| *Isatidis Radix* | Banlangen | MOL001749 | Muscarinic acetylcholine receptor M3 | CHRM3 |
| *Isatidis Radix* | Banlangen | MOL001779 | Muscarinic acetylcholine receptor M3 | CHRM3 |
| *Isatidis Radix* | Banlangen | MOL001779 | Muscarinic acetylcholine receptor M5 | CHRM3 |
| *Isatidis Radix* | Banlangen | MOL000358 | Muscarinic acetylcholine receptor M3 | CHRM3 |
| *Isatidis Radix* | Banlangen | MOL000449 | Muscarinic acetylcholine receptor M3 | CHRM3 |
| *Isatidis Radix* | Banlangen | MOL001721 | Muscarinic acetylcholine receptor M4 | CHRM4 |
| *Isatidis Radix* | Banlangen | MOL000358 | Muscarinic acetylcholine receptor M4 | CHRM4 |
| *Isatidis Radix* | Banlangen | MOL000358 | Neuronal acetylcholine receptor subunit alpha-2 | CHRNA2 |
| *Isatidis Radix* | Banlangen | MOL001820 | Neuronal acetylcholine receptor protein, alpha-7 chain | CHRNA7 |
| *Isatidis Radix* | Banlangen | MOL000358 | Neuronal acetylcholine receptor protein, alpha-7 chain | CHRNA7 |
| *Isatidis Radix* | Banlangen | MOL000449 | Neuronal acetylcholine receptor protein, alpha-7 chain | CHRNA7 |
| *Isatidis Radix* | Banlangen | MOL000449 | Chymotrypsinogen B | CTRB1 |
| *Isatidis Radix* | Banlangen | MOL001689 | Cytochrome P450 19A1 | CYP19A1 |
| *Isatidis Radix* | Banlangen | MOL001689 | Dipeptidyl peptidase IV | DPP4 |
| *Isatidis Radix* | Banlangen | MOL001733 | Dipeptidyl peptidase IV | DPP4 |
| *Isatidis Radix* | Banlangen | MOL001735 | Dipeptidyl peptidase IV | DPP4 |
| *Isatidis Radix* | Banlangen | MOL001779 | Dipeptidyl peptidase IV | DPP4 |
| *Isatidis Radix* | Banlangen | MOL001803 | Dipeptidyl peptidase IV | DPP4 |
| *Isatidis Radix* | Banlangen | MOL000358 | Dopamine D1 receptor | DRD1 |
| *Isatidis Radix* | Banlangen | MOL001767 | Estrogen receptor | ESR1 |
| *Isatidis Radix* | Banlangen | MOL001779 | Estrogen receptor | ESR1 |
| *Isatidis Radix* | Banlangen | MOL001782 | Estrogen receptor | ESR1 |
| *Isatidis Radix* | Banlangen | MOL001792 | Estrogen receptor | ESR1 |
| *Isatidis Radix* | Banlangen | MOL001793 | Estrogen receptor | ESR1 |
| *Isatidis Radix* | Banlangen | MOL001810 | Estrogen receptor | ESR1 |
| *Isatidis Radix* | Banlangen | MOL001833 | Estrogen receptor | ESR1 |
| *Isatidis Radix* | Banlangen | MOL001779 | Estrogen receptor beta | ESR2 |
| *Isatidis Radix* | Banlangen | MOL001803 | Estrogen receptor beta | ESR2 |
| *Isatidis Radix* | Banlangen | MOL001733 | Coagulation factor Xa | F10 |
| *Isatidis Radix* | Banlangen | MOL001734 | Coagulation factor Xa | F10 |
| *Isatidis Radix* | Banlangen | MOL001783 | Coagulation factor Xa | F10 |
| *Isatidis Radix* | Banlangen | MOL001803 | Coagulation factor Xa | F10 |
| *Isatidis Radix* | Banlangen | MOL001828 | Coagulation factor Xa | F10 |
| *Isatidis Radix* | Banlangen | MOL001733 | Thrombin | F2 |
| *Isatidis Radix* | Banlangen | MOL001735 | Thrombin | F2 |
| *Isatidis Radix* | Banlangen | MOL001750 | Thrombin | F2 |
| *Isatidis Radix* | Banlangen | MOL001779 | Thrombin | F2 |
| *Isatidis Radix* | Banlangen | MOL001803 | Thrombin | F2 |
| *Isatidis Radix* | Banlangen | MOL001733 | Coagulation factor VII | F7 |
| *Isatidis Radix* | Banlangen | MOL001803 | Coagulation factor VII | F7 |
| *Isatidis Radix* | Banlangen | MOL001828 | Coagulation factor VII | F7 |
| *Isatidis Radix* | Banlangen | MOL001689 | Tumor necrosis factor ligand superfamily member 6 | FASLG |
| *Isatidis Radix* | Banlangen | MOL001689 | Fatty acid synthase | FASN |
| *Isatidis Radix* | Banlangen | MOL001782 | Gamma-aminobutyric acid receptor subunit alpha-1 | GABRA1 |
| *Isatidis Radix* | Banlangen | MOL001814 | Gamma-aminobutyric acid receptor subunit alpha-1 | GABRA1 |
| *Isatidis Radix* | Banlangen | MOL001820 | Gamma-aminobutyric acid receptor subunit alpha-1 | GABRA1 |
| *Isatidis Radix* | Banlangen | MOL000358 | Gamma-aminobutyric acid receptor subunit alpha-1 | GABRA1 |
| *Isatidis Radix* | Banlangen | MOL000449 | Gamma-aminobutyric acid receptor subunit alpha-1 | GABRA1 |
| *Isatidis Radix* | Banlangen | MOL000358 | Gamma-aminobutyric-acid receptor alpha-2 subunit | GABRA2 |
| *Isatidis Radix* | Banlangen | MOL000358 | Gamma-aminobutyric-acid receptor alpha-3 subunit | GABRA3 |
| *Isatidis Radix* | Banlangen | MOL000449 | Gamma-aminobutyric-acid receptor alpha-3 subunit | GABRA3 |
| *Isatidis Radix* | Banlangen | MOL000358 | Gamma-aminobutyric-acid receptor alpha-5 subunit | GABRA5 |
| *Isatidis Radix* | Banlangen | MOL001767 | Glycogen synthase kinase-3 beta | GSK3B |
| *Isatidis Radix* | Banlangen | MOL001782 | Glycogen synthase kinase-3 beta | GSK3B |
| *Isatidis Radix* | Banlangen | MOL001793 | Glycogen synthase kinase-3 beta | GSK3B |
| *Isatidis Radix* | Banlangen | MOL001689 | Heat shock protein HSP 90 | HSP90AB1 |
| *Isatidis Radix* | Banlangen | MOL001721 | Heat shock protein HSP 90 | HSP90AB1 |
| *Isatidis Radix* | Banlangen | MOL001722 | Heat shock protein HSP 90 | HSP90AB1 |
| *Isatidis Radix* | Banlangen | MOL001733 | Heat shock protein HSP 90 | HSP90AB1 |
| *Isatidis Radix* | Banlangen | MOL001734 | Heat shock protein HSP 90 | HSP90AB1 |
| *Isatidis Radix* | Banlangen | MOL001735 | Heat shock protein HSP 90 | HSP90AB1 |
| *Isatidis Radix* | Banlangen | MOL001736 | Heat shock protein HSP 90 | HSP90AB1 |
| *Isatidis Radix* | Banlangen | MOL001767 | Heat shock protein HSP 90 | HSP90AB1 |
| *Isatidis Radix* | Banlangen | MOL001779 | Heat shock protein HSP 90 | HSP90AB1 |
| *Isatidis Radix* | Banlangen | MOL001782 | Heat shock protein HSP 90 | HSP90AB1 |
| *Isatidis Radix* | Banlangen | MOL001783 | Heat shock protein HSP 90 | HSP90AB1 |
| *Isatidis Radix* | Banlangen | MOL001792 | Heat shock protein HSP 90 | HSP90AB1 |
| *Isatidis Radix* | Banlangen | MOL001793 | Heat shock protein HSP 90 | HSP90AB1 |
| *Isatidis Radix* | Banlangen | MOL001798 | Heat shock protein HSP 90 | HSP90AB1 |
| *Isatidis Radix* | Banlangen | MOL001803 | Heat shock protein HSP 90 | HSP90AB1 |
| *Isatidis Radix* | Banlangen | MOL001814 | Heat shock protein HSP 90 | HSP90AB1 |
| *Isatidis Radix* | Banlangen | MOL001820 | Heat shock protein HSP 90 | HSP90AB1 |
| *Isatidis Radix* | Banlangen | MOL001828 | Heat shock protein HSP 90 | HSP90AB1 |
| *Isatidis Radix* | Banlangen | MOL000358 | Heat shock protein HSP 90 | HSP90AB1 |
| *Isatidis Radix* | Banlangen | MOL000358 | 5-hydroxytryptamine 2A receptor | HTR2A |
| *Isatidis Radix* | Banlangen | MOL000449 | 5-hydroxytryptamine 2A receptor | HTR2A |
| *Isatidis Radix* | Banlangen | MOL001735 | Ig gamma-1 chain C region | IGHG1 |
| *Isatidis Radix* | Banlangen | MOL000449 | Ig gamma-1 chain C region | IGHG1 |
| *Isatidis Radix* | Banlangen | MOL002322 | Inhibitor of nuclear factor kappa-B kinase subunit beta | IKBKB |
| *Isatidis Radix* | Banlangen | MOL000358 | Transcription factor AP-1 | JUN |
| *Isatidis Radix* | Banlangen | MOL001783 | Potassium voltage-gated channel subfamily H member 2 | KCNH2 |
| *Isatidis Radix* | Banlangen | MOL001803 | Potassium voltage-gated channel subfamily H member 2 | KCNH2 |
| *Isatidis Radix* | Banlangen | MOL001828 | Potassium voltage-gated channel subfamily H member 2 | KCNH2 |
| *Isatidis Radix* | Banlangen | MOL000358 | Potassium voltage-gated channel subfamily H member 2 | KCNH2 |
| *Isatidis Radix* | Banlangen | MOL001783 | Calcium-activated potassium channel subunit alpha 1 | KCNMA1 |
| *Isatidis Radix* | Banlangen | MOL001810 | Vascular endothelial growth factor receptor 2 | KDR |
| *Isatidis Radix* | Banlangen | MOL001792 | Beta-lactamase | LACTB |
| *Isatidis Radix* | Banlangen | MOL000449 | Leukotriene A-4 hydrolase | LTA4H |
| *Isatidis Radix* | Banlangen | MOL000449 | Amine oxidase [flavin-containing] A | MAOA |
| *Isatidis Radix* | Banlangen | MOL001756 | Amine oxidase [flavin-containing] B | MAOB |
| *Isatidis Radix* | Banlangen | MOL001792 | Amine oxidase [flavin-containing] B | MAOB |
| *Isatidis Radix* | Banlangen | MOL000449 | Amine oxidase [flavin-containing] B | MAOB |
| *Isatidis Radix* | Banlangen | MOL000358 | Microtubule-associated protein 2 | MAP2 |
| *Isatidis Radix* | Banlangen | MOL001767 | Mitogen-activated protein kinase 14 | MAPK14 |
| *Isatidis Radix* | Banlangen | MOL001782 | Mitogen-activated protein kinase 14 | MAPK14 |
| *Isatidis Radix* | Banlangen | MOL001793 | Mitogen-activated protein kinase 14 | MAPK14 |
| *Isatidis Radix* | Banlangen | MOL001689 | Nuclear receptor coactivator 1 | NCOA1 |
| *Isatidis Radix* | Banlangen | MOL001733 | Nuclear receptor coactivator 1 | NCOA1 |
| *Isatidis Radix* | Banlangen | MOL001735 | Nuclear receptor coactivator 1 | NCOA1 |
| *Isatidis Radix* | Banlangen | MOL001803 | Nuclear receptor coactivator 1 | NCOA1 |
| *Isatidis Radix* | Banlangen | MOL000449 | Nuclear receptor coactivator 1 | NCOA1 |
| *Isatidis Radix* | Banlangen | MOL001689 | Nuclear receptor coactivator 2 | NCOA2 |
| *Isatidis Radix* | Banlangen | MOL001733 | Nuclear receptor coactivator 2 | NCOA2 |
| *Isatidis Radix* | Banlangen | MOL001735 | Nuclear receptor coactivator 2 | NCOA2 |
| *Isatidis Radix* | Banlangen | MOL001756 | Nuclear receptor coactivator 2 | NCOA2 |
| *Isatidis Radix* | Banlangen | MOL001771 | Nuclear receptor coactivator 2 | NCOA2 |
| *Isatidis Radix* | Banlangen | MOL001783 | Nuclear receptor coactivator 2 | NCOA2 |
| *Isatidis Radix* | Banlangen | MOL001803 | Nuclear receptor coactivator 2 | NCOA2 |
| *Isatidis Radix* | Banlangen | MOL001804 | Nuclear receptor coactivator 2 | NCOA2 |
| *Isatidis Radix* | Banlangen | MOL000358 | Nuclear receptor coactivator 2 | NCOA2 |
| *Isatidis Radix* | Banlangen | MOL000359 | Nuclear receptor coactivator 2 | NCOA2 |
| *Isatidis Radix* | Banlangen | MOL000449 | Nuclear receptor coactivator 2 | NCOA2 |
| *Isatidis Radix* | Banlangen | MOL000953 | Nuclear receptor coactivator 2 | NCOA2 |
| *Isatidis Radix* | Banlangen | MOL001689 | Nitric oxide synthase, inducible | NOS2 |
| *Isatidis Radix* | Banlangen | MOL001733 | Nitric oxide synthase, inducible | NOS2 |
| *Isatidis Radix* | Banlangen | MOL001735 | Nitric oxide synthase, inducible | NOS2 |
| *Isatidis Radix* | Banlangen | MOL001767 | Nitric oxide synthase, inducible | NOS2 |
| *Isatidis Radix* | Banlangen | MOL001779 | Nitric oxide synthase, inducible | NOS2 |
| *Isatidis Radix* | Banlangen | MOL001782 | Nitric oxide synthase, inducible | NOS2 |
| *Isatidis Radix* | Banlangen | MOL001793 | Nitric oxide synthase, inducible | NOS2 |
| *Isatidis Radix* | Banlangen | MOL001803 | Nitric oxide synthase, inducible | NOS2 |
| *Isatidis Radix* | Banlangen | MOL001735 | Nitric-oxide synthase, endothelial | NOS3 |
| *Isatidis Radix* | Banlangen | MOL001779 | Nitric-oxide synthase, endothelial | NOS3 |
| *Isatidis Radix* | Banlangen | MOL001755 | Mineralocorticoid receptor | NR3C2 |
| *Isatidis Radix* | Banlangen | MOL001774 | Mineralocorticoid receptor | NR3C2 |
| *Isatidis Radix* | Banlangen | MOL000359 | Mineralocorticoid receptor | NR3C2 |
| *Isatidis Radix* | Banlangen | MOL000449 | Mineralocorticoid receptor | NR3C2 |
| *Isatidis Radix* | Banlangen | MOL000953 | Mineralocorticoid receptor | NR3C2 |
| *Isatidis Radix* | Banlangen | MOL001721 | Delta-type opioid receptor | OPRD1 |
| *Isatidis Radix* | Banlangen | MOL001779 | Delta-type opioid receptor | OPRD1 |
| *Isatidis Radix* | Banlangen | MOL001779 | Mu-type opioid receptor | OPRM1 |
| *Isatidis Radix* | Banlangen | MOL000358 | Mu-type opioid receptor | OPRM1 |
| *Isatidis Radix* | Banlangen | MOL001689 | CGMP-inhibited 3',5'-cyclic phosphodiesterase A | PDE3A |
| *Isatidis Radix* | Banlangen | MOL001814 | CGMP-inhibited 3',5'-cyclic phosphodiesterase A | PDE3A |
| *Isatidis Radix* | Banlangen | MOL001820 | CGMP-inhibited 3',5'-cyclic phosphodiesterase A | PDE3A |
| *Isatidis Radix* | Banlangen | MOL000358 | CGMP-inhibited 3',5'-cyclic phosphodiesterase A | PDE3A |
| *Isatidis Radix* | Banlangen | MOL001755 | Progesterone receptor | PGR |
| *Isatidis Radix* | Banlangen | MOL001771 | Progesterone receptor | PGR |
| *Isatidis Radix* | Banlangen | MOL001800 | Progesterone receptor | PGR |
| *Isatidis Radix* | Banlangen | MOL001804 | Progesterone receptor | PGR |
| *Isatidis Radix* | Banlangen | MOL000358 | Progesterone receptor | PGR |
| *Isatidis Radix* | Banlangen | MOL000359 | Progesterone receptor | PGR |
| *Isatidis Radix* | Banlangen | MOL000449 | Progesterone receptor | PGR |
| *Isatidis Radix* | Banlangen | MOL000953 | Progesterone receptor | PGR |
| *Isatidis Radix* | Banlangen | MOL001689 | Phosphatidylinositol-4,5-bisphosphate 3-kinase catalytic subunit, gamma isoform | PIK3CG |
| *Isatidis Radix* | Banlangen | MOL001735 | Phosphatidylinositol-4,5-bisphosphate 3-kinase catalytic subunit, gamma isoform | PIK3CG |
| *Isatidis Radix* | Banlangen | MOL001736 | Phosphatidylinositol-4,5-bisphosphate 3-kinase catalytic subunit, gamma isoform | PIK3CG |
| *Isatidis Radix* | Banlangen | MOL001767 | Phosphatidylinositol-4,5-bisphosphate 3-kinase catalytic subunit, gamma isoform | PIK3CG |
| *Isatidis Radix* | Banlangen | MOL001792 | Phosphatidylinositol-4,5-bisphosphate 3-kinase catalytic subunit, gamma isoform | PIK3CG |
| *Isatidis Radix* | Banlangen | MOL001793 | Phosphatidylinositol-4,5-bisphosphate 3-kinase catalytic subunit, gamma isoform | PIK3CG |
| *Isatidis Radix* | Banlangen | MOL001798 | Phosphatidylinositol-4,5-bisphosphate 3-kinase catalytic subunit, gamma isoform | PIK3CG |
| *Isatidis Radix* | Banlangen | MOL001814 | Phosphatidylinositol-4,5-bisphosphate 3-kinase catalytic subunit, gamma isoform | PIK3CG |
| *Isatidis Radix* | Banlangen | MOL001820 | Phosphatidylinositol-4,5-bisphosphate 3-kinase catalytic subunit, gamma isoform | PIK3CG |
| *Isatidis Radix* | Banlangen | MOL000358 | Phosphatidylinositol-4,5-bisphosphate 3-kinase catalytic subunit, gamma isoform | PIK3CG |
| *Isatidis Radix* | Banlangen | MOL001767 | Proto-oncogene serine/threonine-protein kinase Pim-1 | PIM1 |
| *Isatidis Radix* | Banlangen | MOL001781 | Proto-oncogene serine/threonine-protein kinase Pim-1 | PIM1 |
| *Isatidis Radix* | Banlangen | MOL001782 | Proto-oncogene serine/threonine-protein kinase Pim-1 | PIM1 |
| *Isatidis Radix* | Banlangen | MOL001793 | Proto-oncogene serine/threonine-protein kinase Pim-1 | PIM1 |
| *Isatidis Radix* | Banlangen | MOL001756 | cAMP-dependent protein kinase inhibitor alpha | PKIA |
| *Isatidis Radix* | Banlangen | MOL001792 | cAMP-dependent protein kinase inhibitor alpha | PKIA |
| *Isatidis Radix* | Banlangen | MOL000449 | Urokinase-type plasminogen activator | PLAU |
| *Isatidis Radix* | Banlangen | MOL000358 | Serum paraoxonase/arylesterase 1 | PON1 |
| *Isatidis Radix* | Banlangen | MOL001767 | Peroxisome proliferator activated receptor gamma | PPARG |
| *Isatidis Radix* | Banlangen | MOL000358 | Protein kinase C alpha type | PRKCA |
| *Isatidis Radix* | Banlangen | MOL001689 | Trypsin-1 | PRSS1 |
| *Isatidis Radix* | Banlangen | MOL001733 | Trypsin-1 | PRSS1 |
| *Isatidis Radix* | Banlangen | MOL001735 | Trypsin-1 | PRSS1 |
| *Isatidis Radix* | Banlangen | MOL001783 | Trypsin-1 | PRSS1 |
| *Isatidis Radix* | Banlangen | MOL001803 | Trypsin-1 | PRSS1 |
| *Isatidis Radix* | Banlangen | MOL001810 | Trypsin-1 | PRSS1 |
| *Isatidis Radix* | Banlangen | MOL001828 | Trypsin-1 | PRSS1 |
| *Isatidis Radix* | Banlangen | MOL001689 | Prostaglandin G/H synthase 1 | PTGS1 |
| *Isatidis Radix* | Banlangen | MOL001721 | Prostaglandin G/H synthase 1 | PTGS1 |
| *Isatidis Radix* | Banlangen | MOL001733 | Prostaglandin G/H synthase 1 | PTGS1 |
| *Isatidis Radix* | Banlangen | MOL001735 | Prostaglandin G/H synthase 1 | PTGS1 |
| *Isatidis Radix* | Banlangen | MOL001736 | Prostaglandin G/H synthase 1 | PTGS1 |
| *Isatidis Radix* | Banlangen | MOL001756 | Prostaglandin G/H synthase 1 | PTGS1 |
| *Isatidis Radix* | Banlangen | MOL001767 | Prostaglandin G/H synthase 1 | PTGS1 |
| *Isatidis Radix* | Banlangen | MOL001779 | Prostaglandin G/H synthase 1 | PTGS1 |
| *Isatidis Radix* | Banlangen | MOL001781 | Prostaglandin G/H synthase 1 | PTGS1 |
| *Isatidis Radix* | Banlangen | MOL001782 | Prostaglandin G/H synthase 1 | PTGS1 |
| *Isatidis Radix* | Banlangen | MOL001792 | Prostaglandin G/H synthase 1 | PTGS1 |
| *Isatidis Radix* | Banlangen | MOL001793 | Prostaglandin G/H synthase 1 | PTGS1 |
| *Isatidis Radix* | Banlangen | MOL001793 | Prostaglandin G/H synthase 2 | PTGS1 |
| *Isatidis Radix* | Banlangen | MOL001798 | Prostaglandin G/H synthase 1 | PTGS1 |
| *Isatidis Radix* | Banlangen | MOL001803 | Prostaglandin G/H synthase 1 | PTGS1 |
| *Isatidis Radix* | Banlangen | MOL001810 | Prostaglandin G/H synthase 1 | PTGS1 |
| *Isatidis Radix* | Banlangen | MOL001814 | Prostaglandin G/H synthase 1 | PTGS1 |
| *Isatidis Radix* | Banlangen | MOL001820 | Prostaglandin G/H synthase 1 | PTGS1 |
| *Isatidis Radix* | Banlangen | MOL001828 | Prostaglandin G/H synthase 1 | PTGS1 |
| *Isatidis Radix* | Banlangen | MOL000358 | Prostaglandin G/H synthase 1 | PTGS1 |
| *Isatidis Radix* | Banlangen | MOL000449 | Prostaglandin G/H synthase 1 | PTGS1 |
| *Isatidis Radix* | Banlangen | MOL001689 | Prostaglandin G/H synthase 2 | PTGS2 |
| *Isatidis Radix* | Banlangen | MOL002322 | Prostaglandin G/H synthase 2 | PTGS2 |
| *Isatidis Radix* | Banlangen | MOL001721 | Prostaglandin G/H synthase 2 | PTGS2 |
| *Isatidis Radix* | Banlangen | MOL001722 | Prostaglandin G/H synthase 2 | PTGS2 |
| *Isatidis Radix* | Banlangen | MOL001733 | Prostaglandin G/H synthase 2 | PTGS2 |
| *Isatidis Radix* | Banlangen | MOL001734 | Prostaglandin G/H synthase 2 | PTGS2 |
| *Isatidis Radix* | Banlangen | MOL001735 | Prostaglandin G/H synthase 2 | PTGS2 |
| *Isatidis Radix* | Banlangen | MOL001736 | Prostaglandin G/H synthase 2 | PTGS2 |
| *Isatidis Radix* | Banlangen | MOL001756 | Prostaglandin G/H synthase 2 | PTGS2 |
| *Isatidis Radix* | Banlangen | MOL001767 | Prostaglandin G/H synthase 2 | PTGS2 |
| *Isatidis Radix* | Banlangen | MOL001779 | Prostaglandin G/H synthase 2 | PTGS2 |
| *Isatidis Radix* | Banlangen | MOL001781 | Prostaglandin G/H synthase 2 | PTGS2 |
| *Isatidis Radix* | Banlangen | MOL001782 | Prostaglandin G/H synthase 2 | PTGS2 |
| *Isatidis Radix* | Banlangen | MOL001783 | Prostaglandin G/H synthase 2 | PTGS2 |
| *Isatidis Radix* | Banlangen | MOL001792 | Prostaglandin G/H synthase 2 | PTGS2 |
| *Isatidis Radix* | Banlangen | MOL001798 | Prostaglandin G/H synthase 2 | PTGS2 |
| *Isatidis Radix* | Banlangen | MOL001803 | Prostaglandin G/H synthase 2 | PTGS2 |
| *Isatidis Radix* | Banlangen | MOL001810 | Prostaglandin G/H synthase 2 | PTGS2 |
| *Isatidis Radix* | Banlangen | MOL001814 | Prostaglandin G/H synthase 2 | PTGS2 |
| *Isatidis Radix* | Banlangen | MOL001820 | Prostaglandin G/H synthase 2 | PTGS2 |
| *Isatidis Radix* | Banlangen | MOL001828 | Prostaglandin G/H synthase 2 | PTGS2 |
| *Isatidis Radix* | Banlangen | MOL001833 | Prostaglandin G/H synthase 2 | PTGS2 |
| *Isatidis Radix* | Banlangen | MOL000358 | Prostaglandin G/H synthase 2 | PTGS2 |
| *Isatidis Radix* | Banlangen | MOL000449 | Prostaglandin G/H synthase 2 | PTGS2 |
| *Isatidis Radix* | Banlangen | MOL001734 | mRNA of Protein-tyrosine phosphatase, non-receptor type 1 | PTPN1 |
| *Isatidis Radix* | Banlangen | MOL001820 | mRNA of Protein-tyrosine phosphatase, non-receptor type 1 | PTPN1 |
| *Isatidis Radix* | Banlangen | MOL001689 | Transcription factor p65 | RELA |
| *Isatidis Radix* | Banlangen | MOL002322 | Transcription factor p65 | RELA |
| *Isatidis Radix* | Banlangen | MOL001735 | Rhodopsin | RHO |
| *Isatidis Radix* | Banlangen | MOL001779 | Retinoic acid receptor RXR-alpha | RXRA |
| *Isatidis Radix* | Banlangen | MOL001781 | Retinoic acid receptor RXR-alpha | RXRA |
| *Isatidis Radix* | Banlangen | MOL001782 | Retinoic acid receptor RXR-alpha | RXRA |
| *Isatidis Radix* | Banlangen | MOL001792 | Retinoic acid receptor RXR-alpha | RXRA |
| *Isatidis Radix* | Banlangen | MOL001793 | Retinoic acid receptor RXR-alpha | RXRA |
| *Isatidis Radix* | Banlangen | MOL001814 | Retinoic acid receptor RXR-alpha | RXRA |
| *Isatidis Radix* | Banlangen | MOL001820 | Retinoic acid receptor RXR-alpha | RXRA |
| *Isatidis Radix* | Banlangen | MOL000449 | Retinoic acid receptor RXR-alpha | RXRA |
| *Isatidis Radix* | Banlangen | MOL001733 | Sodium channel protein type 5 subunit alpha | SCN5A |
| *Isatidis Radix* | Banlangen | MOL001749 | Sodium channel protein type 5 subunit alpha | SCN5A |
| *Isatidis Radix* | Banlangen | MOL001779 | Sodium channel protein type 5 subunit alpha | SCN5A |
| *Isatidis Radix* | Banlangen | MOL001798 | Sodium channel protein type 5 subunit alpha | SCN5A |
| *Isatidis Radix* | Banlangen | MOL001803 | Sodium channel protein type 5 subunit alpha | SCN5A |
| *Isatidis Radix* | Banlangen | MOL001814 | Sodium channel protein type 5 subunit alpha | SCN5A |
| *Isatidis Radix* | Banlangen | MOL001820 | Sodium channel protein type 5 subunit alpha | SCN5A |
| *Isatidis Radix* | Banlangen | MOL001828 | Sodium channel protein type 5 subunit alpha | SCN5A |
| *Isatidis Radix* | Banlangen | MOL000358 | Sodium channel protein type 5 subunit alpha | SCN5A |
| *Isatidis Radix* | Banlangen | MOL000449 | Sodium channel protein type 5 subunit alpha | SCN5A |
| *Isatidis Radix* | Banlangen | MOL000449 | Sodium-dependent noradrenaline transporter | SLC6A2 |
| *Isatidis Radix* | Banlangen | MOL000449 | Sodium-dependent dopamine transporter | SLC6A3 |
| *Isatidis Radix* | Banlangen | MOL001792 | Sodium-dependent serotonin transporter | SLC6A4 |
| *Isatidis Radix* | Banlangen | MOL000358 | Sodium-dependent serotonin transporter | SLC6A4 |
| *Isatidis Radix* | Banlangen | MOL000358 | Transforming growth factor beta-1 | TGFB1 |
| *Isatidis Radix* | Banlangen | MOL002322 | Tumor necrosis factor | TNF |
| *Isatidis Radix* | Banlangen | MOL002322 | DNA topoisomerase II | TOP2B |
| *Isatidis Radix* | Banlangen | MOL001733 | DNA topoisomerase II | TOP2B |
| *Isatidis Radix* | Banlangen | MOL001779 | DNA topoisomerase II | TOP2B |
| *Isatidis Radix* | Banlangen | MOL001783 | DNA topoisomerase II | TOP2B |
| *Isatidis Radix* | Banlangen | MOL001790 | DNA topoisomerase II | TOP2B |
| *Isatidis Radix* | Banlangen | MOL001803 | DNA topoisomerase II | TOP2B |
| *Isatidis Radix* | Banlangen | MOL001689 | Cellular tumor antigen p53 | TP53 |
| *Lonicerae Japonicae Flos* | Jinyinhua | MOL000098 | ATP-binding cassette sub-family G member 2 | ABCG2 |
| *Lonicerae Japonicae Flos* | Jinyinhua | MOL000098 | Acetyl-CoA carboxylase 1 | ACACA |
| *Lonicerae Japonicae Flos* | Jinyinhua | MOL000422 | Acetylcholinesterase | ACHE |
| *Lonicerae Japonicae Flos* | Jinyinhua | MOL000098 | Acetylcholinesterase | ACHE |
| *Lonicerae Japonicae Flos* | Jinyinhua | MOL000098 | Prostatic acid phosphatase | ACP3 |
| *Lonicerae Japonicae Flos* | Jinyinhua | MOL000006 | Adenylate cyclase type 2 | ADCY2 |
| *Lonicerae Japonicae Flos* | Jinyinhua | MOL000449 | Alcohol dehydrogenase 1C | ADH1C |
| *Lonicerae Japonicae Flos* | Jinyinhua | MOL000358 | Alpha-1A adrenergic receptor | ADRA1A |
| *Lonicerae Japonicae Flos* | Jinyinhua | MOL000449 | Alpha-1A adrenergic receptor | ADRA1A |
| *Lonicerae Japonicae Flos* | Jinyinhua | MOL000358 | Alpha-1B adrenergic receptor | ADRA1B |
| *Lonicerae Japonicae Flos* | Jinyinhua | MOL000422 | Alpha-1B adrenergic receptor | ADRA1B |
| *Lonicerae Japonicae Flos* | Jinyinhua | MOL000449 | Alpha-1B adrenergic receptor | ADRA1B |
| *Lonicerae Japonicae Flos* | Jinyinhua | MOL000449 | Alpha-2A adrenergic receptor | ADRA2A |
| *Lonicerae Japonicae Flos* | Jinyinhua | MOL000449 | Beta-1 adrenergic receptor | ADRB1 |
| *Lonicerae Japonicae Flos* | Jinyinhua | MOL003095 | Beta-2 adrenergic receptor | ADRB2 |
| *Lonicerae Japonicae Flos* | Jinyinhua | MOL000358 | Beta-2 adrenergic receptor | ADRB2 |
| *Lonicerae Japonicae Flos* | Jinyinhua | MOL000449 | Beta-2 adrenergic receptor | ADRB2 |
| *Lonicerae Japonicae Flos* | Jinyinhua | MOL000098 | Beta-2 adrenergic receptor | ADRB2 |
| *Lonicerae Japonicae Flos* | Jinyinhua | MOL000422 | Aryl hydrocarbon receptor | AHR |
| *Lonicerae Japonicae Flos* | Jinyinhua | MOL000098 | Aryl hydrocarbon receptor | AHR |
| *Lonicerae Japonicae Flos* | Jinyinhua | MOL000422 | Activator of 90 kDa heat shock protein ATPase homolog 1 | AHSA1 |
| *Lonicerae Japonicae Flos* | Jinyinhua | MOL000098 | Activator of 90 kDa heat shock protein ATPase homolog 1 | AHSA1 |
| *Lonicerae Japonicae Flos* | Jinyinhua | MOL000449 | Aldose reductase | AKR1B1 |
| *Lonicerae Japonicae Flos* | Jinyinhua | MOL000098 | Aldose reductase | AKR1B1 |
| *Lonicerae Japonicae Flos* | Jinyinhua | MOL000422 | Aldo-keto reductase family 1 member C3 | AKR1C3 |
| *Lonicerae Japonicae Flos* | Jinyinhua | MOL002773 | RAC-alpha serine/threonine-protein kinase | AKT1 |
| *Lonicerae Japonicae Flos* | Jinyinhua | MOL000422 | RAC-alpha serine/threonine-protein kinase | AKT1 |
| *Lonicerae Japonicae Flos* | Jinyinhua | MOL000006 | RAC-alpha serine/threonine-protein kinase | AKT1 |
| *Lonicerae Japonicae Flos* | Jinyinhua | MOL000098 | RAC-alpha serine/threonine-protein kinase | AKT1 |
| *Lonicerae Japonicae Flos* | Jinyinhua | MOL002773 | Serum albumin | ALB |
| *Lonicerae Japonicae Flos* | Jinyinhua | MOL000422 | Arachidonate 5-lipoxygenase | ALOX5 |
| *Lonicerae Japonicae Flos* | Jinyinhua | MOL000098 | Arachidonate 5-lipoxygenase | ALOX5 |
| *Lonicerae Japonicae Flos* | Jinyinhua | MOL000006 | Amyloid beta A4 protein | APP |
| *Lonicerae Japonicae Flos* | Jinyinhua | MOL003044 | Androgen receptor | AR |
| *Lonicerae Japonicae Flos* | Jinyinhua | MOL003095 | Androgen receptor | AR |
| *Lonicerae Japonicae Flos* | Jinyinhua | MOL003111 | Androgen receptor | AR |
| *Lonicerae Japonicae Flos* | Jinyinhua | MOL003128 | Androgen receptor | AR |
| *Lonicerae Japonicae Flos* | Jinyinhua | MOL000422 | Androgen receptor | AR |
| *Lonicerae Japonicae Flos* | Jinyinhua | MOL000006 | Androgen receptor | AR |
| *Lonicerae Japonicae Flos* | Jinyinhua | MOL000098 | Androgen receptor | AR |
| *Lonicerae Japonicae Flos* | Jinyinhua | MOL003095 | Beta-secretase | BACE1 |
| *Lonicerae Japonicae Flos* | Jinyinhua | MOL000358 | Apoptosis regulator BAX | BAX |
| *Lonicerae Japonicae Flos* | Jinyinhua | MOL000422 | Apoptosis regulator BAX | BAX |
| *Lonicerae Japonicae Flos* | Jinyinhua | MOL000098 | Apoptosis regulator BAX | BAX |
| *Lonicerae Japonicae Flos* | Jinyinhua | MOL002773 | Apoptosis regulator Bcl-2 | BCL2 |
| *Lonicerae Japonicae Flos* | Jinyinhua | MOL000358 | Apoptosis regulator Bcl-2 | BCL2 |
| *Lonicerae Japonicae Flos* | Jinyinhua | MOL000422 | Apoptosis regulator Bcl-2 | BCL2 |
| *Lonicerae Japonicae Flos* | Jinyinhua | MOL000098 | Apoptosis regulator Bcl-2 | BCL2 |
| *Lonicerae Japonicae Flos* | Jinyinhua | MOL000006 | Bcl-2-like protein 1 | BCL2L1 |
| *Lonicerae Japonicae Flos* | Jinyinhua | MOL000098 | Bcl-2-like protein 1 | BCL2L1 |
| *Lonicerae Japonicae Flos* | Jinyinhua | MOL000006 | Baculoviral IAP repeat-containing protein 5 | BIRC5 |
| *Lonicerae Japonicae Flos* | Jinyinhua | MOL000098 | Baculoviral IAP repeat-containing protein 5 | BIRC5 |
| *Lonicerae Japonicae Flos* | Jinyinhua | MOL003006 | Carbonic anhydrase II | CA2 |
| *Lonicerae Japonicae Flos* | Jinyinhua | MOL003111 | Carbonic anhydrase II | CA2 |
| *Lonicerae Japonicae Flos* | Jinyinhua | MOL003128 | Carbonic anhydrase II | CA2 |
| *Lonicerae Japonicae Flos* | Jinyinhua | MOL003095 | Voltage-dependent calcium channel subunit alpha-2/delta-1 | CACNA2D1 |
| *Lonicerae Japonicae Flos* | Jinyinhua | MOL002914 | Calmodulin | CALM1 |
| *Lonicerae Japonicae Flos* | Jinyinhua | MOL003044 | Calmodulin | CALM1 |
| *Lonicerae Japonicae Flos* | Jinyinhua | MOL003095 | Calmodulin | CALM1 |
| *Lonicerae Japonicae Flos* | Jinyinhua | MOL000422 | Calmodulin | CALM1 |
| *Lonicerae Japonicae Flos* | Jinyinhua | MOL002773 | Caspase-3 | CASP3 |
| *Lonicerae Japonicae Flos* | Jinyinhua | MOL000358 | Caspase-3 | CASP3 |
| *Lonicerae Japonicae Flos* | Jinyinhua | MOL000422 | Caspase-3 | CASP3 |
| *Lonicerae Japonicae Flos* | Jinyinhua | MOL000006 | Caspase-3 | CASP3 |
| *Lonicerae Japonicae Flos* | Jinyinhua | MOL000098 | Caspase-3 | CASP3 |
| *Lonicerae Japonicae Flos* | Jinyinhua | MOL002773 | Caspase-7 | CASP7 |
| *Lonicerae Japonicae Flos* | Jinyinhua | MOL000006 | Caspase-7 | CASP7 |
| *Lonicerae Japonicae Flos* | Jinyinhua | MOL002773 | Caspase-8 | CASP8 |
| *Lonicerae Japonicae Flos* | Jinyinhua | MOL000358 | Caspase-8 | CASP8 |
| *Lonicerae Japonicae Flos* | Jinyinhua | MOL000098 | Caspase-8 | CASP8 |
| *Lonicerae Japonicae Flos* | Jinyinhua | MOL002773 | Caspase-9 | CASP9 |
| *Lonicerae Japonicae Flos* | Jinyinhua | MOL000358 | Caspase-9 | CASP9 |
| *Lonicerae Japonicae Flos* | Jinyinhua | MOL000006 | Caspase-9 | CASP9 |
| *Lonicerae Japonicae Flos* | Jinyinhua | MOL000098 | Caspase-9 | CASP9 |
| *Lonicerae Japonicae Flos* | Jinyinhua | MOL002773 | Caveolin-1 | CAV1 |
| *Lonicerae Japonicae Flos* | Jinyinhua | MOL000098 | Caveolin-1 | CAV1 |
| *Lonicerae Japonicae Flos* | Jinyinhua | MOL000098 | C-C motif chemokine 2 | CCL2 |
| *Lonicerae Japonicae Flos* | Jinyinhua | MOL000006 | G2/mitotic-specific cyclin-B1 | CCNB1 |
| *Lonicerae Japonicae Flos* | Jinyinhua | MOL000098 | G2/mitotic-specific cyclin-B1 | CCNB1 |
| *Lonicerae Japonicae Flos* | Jinyinhua | MOL000006 | G1/S-specific cyclin-D1 | CCND1 |
| *Lonicerae Japonicae Flos* | Jinyinhua | MOL000098 | G1/S-specific cyclin-D1 | CCND1 |
| *Lonicerae Japonicae Flos* | Jinyinhua | MOL000006 | CD40 ligand | CD40LG |
| *Lonicerae Japonicae Flos* | Jinyinhua | MOL000098 | CD40 ligand | CD40LG |
| *Lonicerae Japonicae Flos* | Jinyinhua | MOL000422 | Cell division control protein 2 homolog | CDK1 |
| *Lonicerae Japonicae Flos* | Jinyinhua | MOL000098 | Cell division control protein 2 homolog | CDK1 |
| *Lonicerae Japonicae Flos* | Jinyinhua | MOL003044 | Cell division protein kinase 2 | CDK2 |
| *Lonicerae Japonicae Flos* | Jinyinhua | MOL000006 | Cell division protein kinase 4 | CDK4 |
| *Lonicerae Japonicae Flos* | Jinyinhua | MOL000098 | Cyclin-dependent kinase inhibitor 1 | CDKN1A |
| *Lonicerae Japonicae Flos* | Jinyinhua | MOL000006 | Cyclin-dependent kinase inhibitor 1 | CDKN1B |
| *Lonicerae Japonicae Flos* | Jinyinhua | MOL000098 | Cyclin-dependent kinase inhibitor 2A, isoforms 1/2/3 | CDKN2A |
| *Lonicerae Japonicae Flos* | Jinyinhua | MOL003044 | Serine/threonine-protein kinase Chk1 | CHEK1 |
| *Lonicerae Japonicae Flos* | Jinyinhua | MOL003095 | Serine/threonine-protein kinase Chk1 | CHEK1 |
| *Lonicerae Japonicae Flos* | Jinyinhua | MOL000098 | Serine/threonine-protein kinase Chk2 | CHEK2 |
| *Lonicerae Japonicae Flos* | Jinyinhua | MOL000358 | Muscarinic acetylcholine receptor M1 | CHRM1 |
| *Lonicerae Japonicae Flos* | Jinyinhua | MOL000422 | Muscarinic acetylcholine receptor M1 | CHRM1 |
| *Lonicerae Japonicae Flos* | Jinyinhua | MOL000449 | Muscarinic acetylcholine receptor M1 | CHRM1 |
| *Lonicerae Japonicae Flos* | Jinyinhua | MOL000358 | Muscarinic acetylcholine receptor M2 | CHRM2 |
| *Lonicerae Japonicae Flos* | Jinyinhua | MOL000422 | Muscarinic acetylcholine receptor M2 | CHRM2 |
| *Lonicerae Japonicae Flos* | Jinyinhua | MOL000449 | Muscarinic acetylcholine receptor M2 | CHRM2 |
| *Lonicerae Japonicae Flos* | Jinyinhua | MOL000358 | Muscarinic acetylcholine receptor M3 | CHRM3 |
| *Lonicerae Japonicae Flos* | Jinyinhua | MOL000449 | Muscarinic acetylcholine receptor M3 | CHRM3 |
| *Lonicerae Japonicae Flos* | Jinyinhua | MOL000358 | Muscarinic acetylcholine receptor M4 | CHRM4 |
| *Lonicerae Japonicae Flos* | Jinyinhua | MOL000358 | Neuronal acetylcholine receptor subunit alpha-2 | CHRNA2 |
| *Lonicerae Japonicae Flos* | Jinyinhua | MOL000358 | Neuronal acetylcholine receptor protein, alpha-7 chain | CHRNA7 |
| *Lonicerae Japonicae Flos* | Jinyinhua | MOL000449 | Neuronal acetylcholine receptor protein, alpha-7 chain | CHRNA7 |
| *Lonicerae Japonicae Flos* | Jinyinhua | MOL000098 | Inhibitor of nuclear factor kappa-B kinase subunit alpha | CHUK |
| *Lonicerae Japonicae Flos* | Jinyinhua | MOL000098 | Claudin-4 | CLDN4 |
| *Lonicerae Japonicae Flos* | Jinyinhua | MOL000098 | Collagen alpha-1(I) chain | COL1A1 |
| *Lonicerae Japonicae Flos* | Jinyinhua | MOL000098 | Collagen alpha-1(III) chain | COL3A1 |
| *Lonicerae Japonicae Flos* | Jinyinhua | MOL000098 | C-reactive protein | CRP |
| *Lonicerae Japonicae Flos* | Jinyinhua | MOL002773 | Catenin beta-1 | CTNNB1 |
| *Lonicerae Japonicae Flos* | Jinyinhua | MOL000449 | Chymotrypsinogen B | CTRB1 |
| *Lonicerae Japonicae Flos* | Jinyinhua | MOL000098 | Cathepsin D | CTSD |
| *Lonicerae Japonicae Flos* | Jinyinhua | MOL000098 | C-X-C motif chemokine 10 | CXCL10 |
| *Lonicerae Japonicae Flos* | Jinyinhua | MOL000098 | C-X-C motif chemokine 11 | CXCL11 |
| *Lonicerae Japonicae Flos* | Jinyinhua | MOL000098 | C-X-C motif chemokine 2 | CXCL2 |
| *Lonicerae Japonicae Flos* | Jinyinhua | MOL000098 | Interleukin-8 | CXCL8 |
| *Lonicerae Japonicae Flos* | Jinyinhua | MOL000422 | Cytochrome P450 1A1 | CYP1A1 |
| *Lonicerae Japonicae Flos* | Jinyinhua | MOL000098 | Cytochrome P450 1A1 | CYP1A1 |
| *Lonicerae Japonicae Flos* | Jinyinhua | MOL002773 | Cytochrome P450 1A2 | CYP1A2 |
| *Lonicerae Japonicae Flos* | Jinyinhua | MOL000422 | Cytochrome P450 1A2 | CYP1A2 |
| *Lonicerae Japonicae Flos* | Jinyinhua | MOL000098 | Cytochrome P450 1A2 | CYP1A2 |
| *Lonicerae Japonicae Flos* | Jinyinhua | MOL000422 | Cytochrome P450 1B1 | CYP1B1 |
| *Lonicerae Japonicae Flos* | Jinyinhua | MOL000098 | Cytochrome P450 1B1 | CYP1B1 |
| *Lonicerae Japonicae Flos* | Jinyinhua | MOL002773 | Cytochrome P450 3A4 | CYP3A4 |
| *Lonicerae Japonicae Flos* | Jinyinhua | MOL000422 | Cytochrome P450 3A4 | CYP3A4 |
| *Lonicerae Japonicae Flos* | Jinyinhua | MOL000098 | Cytochrome P450 3A4 | CYP3A4 |
| *Lonicerae Japonicae Flos* | Jinyinhua | MOL000098 | DDB1- and CUL4-associated factor 5 | DCAF5 |
| *Lonicerae Japonicae Flos* | Jinyinhua | MOL000422 | Type I iodothyronine deiodinase | DIO1 |
| *Lonicerae Japonicae Flos* | Jinyinhua | MOL000098 | Type I iodothyronine deiodinase | DIO1 |
| *Lonicerae Japonicae Flos* | Jinyinhua | MOL003006 | Dipeptidyl peptidase IV | DPP4 |
| *Lonicerae Japonicae Flos* | Jinyinhua | MOL003044 | Dipeptidyl peptidase IV | DPP4 |
| *Lonicerae Japonicae Flos* | Jinyinhua | MOL003095 | Dipeptidyl peptidase IV | DPP4 |
| *Lonicerae Japonicae Flos* | Jinyinhua | MOL003111 | Dipeptidyl peptidase IV | DPP4 |
| *Lonicerae Japonicae Flos* | Jinyinhua | MOL003117 | Dipeptidyl peptidase IV | DPP4 |
| *Lonicerae Japonicae Flos* | Jinyinhua | MOL000422 | Dipeptidyl peptidase IV | DPP4 |
| *Lonicerae Japonicae Flos* | Jinyinhua | MOL000006 | Dipeptidyl peptidase IV | DPP4 |
| *Lonicerae Japonicae Flos* | Jinyinhua | MOL000098 | Dipeptidyl peptidase IV | DPP4 |
| *Lonicerae Japonicae Flos* | Jinyinhua | MOL000358 | Dopamine D1 receptor | DRD1 |
| *Lonicerae Japonicae Flos* | Jinyinhua | MOL000098 | Dual oxidase 2 | DUOX2 |
| *Lonicerae Japonicae Flos* | Jinyinhua | MOL000098 | Transcription factor E2F1 | E2F1 |
| *Lonicerae Japonicae Flos* | Jinyinhua | MOL000098 | Transcription factor E2F2 | E2F2 |
| *Lonicerae Japonicae Flos* | Jinyinhua | MOL000098 | Pro-epidermal growth factor | EGF |
| *Lonicerae Japonicae Flos* | Jinyinhua | MOL000006 | Epidermal growth factor receptor | EGFR |
| *Lonicerae Japonicae Flos* | Jinyinhua | MOL000098 | Epidermal growth factor receptor | EGFR |
| *Lonicerae Japonicae Flos* | Jinyinhua | MOL000098 | Eukaryotic translation initiation factor 6 | EIF6 |
| *Lonicerae Japonicae Flos* | Jinyinhua | MOL000098 | ETS domain-containing protein Elk-1 | ELK1 |
| *Lonicerae Japonicae Flos* | Jinyinhua | MOL000006 | Receptor tyrosine-protein kinase erbB-2 | ERBB2 |
| *Lonicerae Japonicae Flos* | Jinyinhua | MOL000098 | Receptor tyrosine-protein kinase erbB-2 | ERBB2 |
| *Lonicerae Japonicae Flos* | Jinyinhua | MOL000098 | Receptor tyrosine-protein kinase erbB-3 | ERBB3 |
| *Lonicerae Japonicae Flos* | Jinyinhua | MOL003014 | Estrogen receptor | ESR1 |
| *Lonicerae Japonicae Flos* | Jinyinhua | MOL003044 | Estrogen receptor | ESR1 |
| *Lonicerae Japonicae Flos* | Jinyinhua | MOL003095 | Estrogen receptor | ESR1 |
| *Lonicerae Japonicae Flos* | Jinyinhua | MOL003111 | Estrogen receptor | ESR1 |
| *Lonicerae Japonicae Flos* | Jinyinhua | MOL003095 | Estrogen receptor beta | ESR2 |
| *Lonicerae Japonicae Flos* | Jinyinhua | MOL003095 | Coagulation factor Xa | F10 |
| *Lonicerae Japonicae Flos* | Jinyinhua | MOL000098 | Coagulation factor Xa | F10 |
| *Lonicerae Japonicae Flos* | Jinyinhua | MOL003014 | Thrombin | F2 |
| *Lonicerae Japonicae Flos* | Jinyinhua | MOL003095 | Thrombin | F2 |
| *Lonicerae Japonicae Flos* | Jinyinhua | MOL003111 | Thrombin | F2 |
| *Lonicerae Japonicae Flos* | Jinyinhua | MOL003117 | Thrombin | F2 |
| *Lonicerae Japonicae Flos* | Jinyinhua | MOL000422 | Thrombin | F2 |
| *Lonicerae Japonicae Flos* | Jinyinhua | MOL000098 | Thrombin | F2 |
| *Lonicerae Japonicae Flos* | Jinyinhua | MOL002773 | Tissue factor | F3 |
| *Lonicerae Japonicae Flos* | Jinyinhua | MOL000098 | Tissue factor | F3 |
| *Lonicerae Japonicae Flos* | Jinyinhua | MOL000422 | Coagulation factor VII | F7 |
| *Lonicerae Japonicae Flos* | Jinyinhua | MOL000098 | Coagulation factor VII | F7 |
| *Lonicerae Japonicae Flos* | Jinyinhua | MOL000098 | Proto-oncogene c-Fos | FOS |
| *Lonicerae Japonicae Flos* | Jinyinhua | MOL003006 | Gamma-aminobutyric acid receptor subunit alpha-1 | GABRA1 |
| *Lonicerae Japonicae Flos* | Jinyinhua | MOL003117 | Gamma-aminobutyric acid receptor subunit alpha-1 | GABRA1 |
| *Lonicerae Japonicae Flos* | Jinyinhua | MOL000358 | Gamma-aminobutyric acid receptor subunit alpha-1 | GABRA1 |
| *Lonicerae Japonicae Flos* | Jinyinhua | MOL000422 | Gamma-aminobutyric acid receptor subunit alpha-1 | GABRA1 |
| *Lonicerae Japonicae Flos* | Jinyinhua | MOL000449 | Gamma-aminobutyric acid receptor subunit alpha-1 | GABRA1 |
| *Lonicerae Japonicae Flos* | Jinyinhua | MOL000098 | Gamma-aminobutyric acid receptor subunit alpha-1 | GABRA1 |
| *Lonicerae Japonicae Flos* | Jinyinhua | MOL000358 | Gamma-aminobutyric-acid receptor alpha-2 subunit | GABRA2 |
| *Lonicerae Japonicae Flos* | Jinyinhua | MOL000422 | Gamma-aminobutyric-acid receptor alpha-2 subunit | GABRA2 |
| *Lonicerae Japonicae Flos* | Jinyinhua | MOL000358 | Gamma-aminobutyric-acid receptor alpha-3 subunit | GABRA3 |
| *Lonicerae Japonicae Flos* | Jinyinhua | MOL000449 | Gamma-aminobutyric-acid receptor alpha-3 subunit | GABRA3 |
| *Lonicerae Japonicae Flos* | Jinyinhua | MOL000358 | Gamma-aminobutyric-acid receptor alpha-5 subunit | GABRA5 |
| *Lonicerae Japonicae Flos* | Jinyinhua | MOL002773 | Gap junction alpha-1 protein | GJA1 |
| *Lonicerae Japonicae Flos* | Jinyinhua | MOL000098 | Gap junction alpha-1 protein | GJA1 |
| *Lonicerae Japonicae Flos* | Jinyinhua | MOL003044 | Glycogen synthase kinase-3 beta | GSK3B |
| *Lonicerae Japonicae Flos* | Jinyinhua | MOL003095 | Glycogen synthase kinase-3 beta | GSK3B |
| *Lonicerae Japonicae Flos* | Jinyinhua | MOL000422 | Glutathione S-transferase Mu 1 | GSTM1 |
| *Lonicerae Japonicae Flos* | Jinyinhua | MOL000098 | Glutathione S-transferase Mu 1 | GSTM1 |
| *Lonicerae Japonicae Flos* | Jinyinhua | MOL000422 | Glutathione S-transferase Mu 2 | GSTM2 |
| *Lonicerae Japonicae Flos* | Jinyinhua | MOL000098 | Glutathione S-transferase Mu 2 | GSTM2 |
| *Lonicerae Japonicae Flos* | Jinyinhua | MOL000422 | Glutathione S-transferase P | GSTP1 |
| *Lonicerae Japonicae Flos* | Jinyinhua | MOL000006 | Glutathione S-transferase P | GSTP1 |
| *Lonicerae Japonicae Flos* | Jinyinhua | MOL000098 | Glutathione S-transferase P | GSTP1 |
| *Lonicerae Japonicae Flos* | Jinyinhua | MOL000422 | Hyaluronan synthase 2 | HAS2 |
| *Lonicerae Japonicae Flos* | Jinyinhua | MOL000098 | Hyaluronan synthase 2 | HAS2 |
| *Lonicerae Japonicae Flos* | Jinyinhua | MOL000098 | Probable E3 ubiquitin-protein ligase HERC5 | HERC5 |
| *Lonicerae Japonicae Flos* | Jinyinhua | MOL000098 | Hypoxia-inducible factor 1-alpha | HIF1A |
| *Lonicerae Japonicae Flos* | Jinyinhua | MOL000098 | Hexokinase-2 | HK2 |
| *Lonicerae Japonicae Flos* | Jinyinhua | MOL002773 | Heme oxygenase 1 | HMOX1 |
| *Lonicerae Japonicae Flos* | Jinyinhua | MOL000422 | Heme oxygenase 1 | HMOX1 |
| *Lonicerae Japonicae Flos* | Jinyinhua | MOL000006 | Heme oxygenase 1 | HMOX1 |
| *Lonicerae Japonicae Flos* | Jinyinhua | MOL000098 | Heme oxygenase 1 | HMOX1 |
| *Lonicerae Japonicae Flos* | Jinyinhua | MOL000098 | Heat shock factor protein 1 | HSF1 |
| *Lonicerae Japonicae Flos* | Jinyinhua | MOL002914 | Heat shock protein HSP 90 | HSP90AB1 |
| *Lonicerae Japonicae Flos* | Jinyinhua | MOL003044 | Heat shock protein HSP 90 | HSP90AB1 |
| *Lonicerae Japonicae Flos* | Jinyinhua | MOL003095 | Heat shock protein HSP 90 | HSP90AB1 |
| *Lonicerae Japonicae Flos* | Jinyinhua | MOL003111 | Heat shock protein HSP 90 | HSP90AB1 |
| *Lonicerae Japonicae Flos* | Jinyinhua | MOL000358 | Heat shock protein HSP 90 | HSP90AB1 |
| *Lonicerae Japonicae Flos* | Jinyinhua | MOL000422 | Heat shock protein HSP 90 | HSP90AB1 |
| *Lonicerae Japonicae Flos* | Jinyinhua | MOL000006 | Heat shock protein HSP 90 | HSP90AB1 |
| *Lonicerae Japonicae Flos* | Jinyinhua | MOL000098 | Heat shock protein HSP 90 | HSP90AB1 |
| *Lonicerae Japonicae Flos* | Jinyinhua | MOL000098 | 78 kDa glucose-regulated protein | HSPA5 |
| *Lonicerae Japonicae Flos* | Jinyinhua | MOL000098 | Heat shock protein beta-1 | HSPB1 |
| *Lonicerae Japonicae Flos* | Jinyinhua | MOL000358 | 5-hydroxytryptamine 2A receptor | HTR2A |
| *Lonicerae Japonicae Flos* | Jinyinhua | MOL000449 | 5-hydroxytryptamine 2A receptor | HTR2A |
| *Lonicerae Japonicae Flos* | Jinyinhua | MOL000422 | Intercellular adhesion molecule 1 | ICAM1 |
| *Lonicerae Japonicae Flos* | Jinyinhua | MOL000006 | Intercellular adhesion molecule 1 | ICAM1 |
| *Lonicerae Japonicae Flos* | Jinyinhua | MOL000098 | Intercellular adhesion molecule 1 | ICAM1 |
| *Lonicerae Japonicae Flos* | Jinyinhua | MOL000006 | Interferon gamma | IFNG |
| *Lonicerae Japonicae Flos* | Jinyinhua | MOL000098 | Interferon gamma | IFNG |
| *Lonicerae Japonicae Flos* | Jinyinhua | MOL000098 | Insulin-like growth factor II | IGF2 |
| *Lonicerae Japonicae Flos* | Jinyinhua | MOL000098 | Insulin-like growth factor-binding protein 3 | IGFBP3 |
| *Lonicerae Japonicae Flos* | Jinyinhua | MOL000449 | Ig gamma-1 chain C region | IGHG1 |
| *Lonicerae Japonicae Flos* | Jinyinhua | MOL000422 | Inhibitor of nuclear factor kappa-B kinase subunit beta | IKBKB |
| *Lonicerae Japonicae Flos* | Jinyinhua | MOL000006 | Interleukin-10 | IL10 |
| *Lonicerae Japonicae Flos* | Jinyinhua | MOL000098 | Interleukin-10 | IL10 |
| *Lonicerae Japonicae Flos* | Jinyinhua | MOL000098 | Interleukin-1 alpha | IL1A |
| *Lonicerae Japonicae Flos* | Jinyinhua | MOL000098 | Interleukin-1 beta | IL1B |
| *Lonicerae Japonicae Flos* | Jinyinhua | MOL000006 | Interleukin-2 | IL2 |
| *Lonicerae Japonicae Flos* | Jinyinhua | MOL000098 | Interleukin-2 | IL2 |
| *Lonicerae Japonicae Flos* | Jinyinhua | MOL000006 | Interleukin-4 | IL4 |
| *Lonicerae Japonicae Flos* | Jinyinhua | MOL000006 | Interleukin-6 | IL6 |
| *Lonicerae Japonicae Flos* | Jinyinhua | MOL000098 | Interleukin-6 | IL6 |
| *Lonicerae Japonicae Flos* | Jinyinhua | MOL000422 | Insulin receptor | INSR |
| *Lonicerae Japonicae Flos* | Jinyinhua | MOL000006 | Insulin receptor | INSR |
| *Lonicerae Japonicae Flos* | Jinyinhua | MOL000098 | Insulin receptor | INSR |
| *Lonicerae Japonicae Flos* | Jinyinhua | MOL000098 | Interferon regulatory factor 1 | IRF1 |
| *Lonicerae Japonicae Flos* | Jinyinhua | MOL002773 | Transcription factor AP-1 | JUN |
| *Lonicerae Japonicae Flos* | Jinyinhua | MOL000358 | Transcription factor AP-1 | JUN |
| *Lonicerae Japonicae Flos* | Jinyinhua | MOL000422 | Transcription factor AP-1 | JUN |
| *Lonicerae Japonicae Flos* | Jinyinhua | MOL000006 | Transcription factor AP-1 | JUN |
| *Lonicerae Japonicae Flos* | Jinyinhua | MOL000098 | Transcription factor AP-1 | JUN |
| *Lonicerae Japonicae Flos* | Jinyinhua | MOL003095 | Potassium voltage-gated channel subfamily H member 2 | KCNH2 |
| *Lonicerae Japonicae Flos* | Jinyinhua | MOL000358 | Potassium voltage-gated channel subfamily H member 2 | KCNH2 |
| *Lonicerae Japonicae Flos* | Jinyinhua | MOL000098 | Potassium voltage-gated channel subfamily H member 2 | KCNH2 |
| *Lonicerae Japonicae Flos* | Jinyinhua | MOL003095 | Calcium-activated potassium channel subunit alpha 1 | KCNMA1 |
| *Lonicerae Japonicae Flos* | Jinyinhua | MOL003117 | Beta-lactamase | LACTB |
| *Lonicerae Japonicae Flos* | Jinyinhua | MOL000449 | Leukotriene A-4 hydrolase | LTA4H |
| *Lonicerae Japonicae Flos* | Jinyinhua | MOL000449 | Amine oxidase [flavin-containing] A | MAOA |
| *Lonicerae Japonicae Flos* | Jinyinhua | MOL000449 | Amine oxidase [flavin-containing] B | MAOB |
| *Lonicerae Japonicae Flos* | Jinyinhua | MOL000098 | Amine oxidase [flavin-containing] B | MAOB |
| *Lonicerae Japonicae Flos* | Jinyinhua | MOL000358 | Microtubule-associated protein 2 | MAP2 |
| *Lonicerae Japonicae Flos* | Jinyinhua | MOL000006 | Mitogen-activated protein kinase 1 | MAPK1 |
| *Lonicerae Japonicae Flos* | Jinyinhua | MOL000098 | Mitogen-activated protein kinase 1 | MAPK1 |
| *Lonicerae Japonicae Flos* | Jinyinhua | MOL003044 | Mitogen-activated protein kinase 14 | MAPK14 |
| *Lonicerae Japonicae Flos* | Jinyinhua | MOL003095 | Mitogen-activated protein kinase 14 | MAPK14 |
| *Lonicerae Japonicae Flos* | Jinyinhua | MOL000422 | Mitogen-activated protein kinase 8 | MAPK8 |
| *Lonicerae Japonicae Flos* | Jinyinhua | MOL000006 | Induced myeloid leukemia cell differentiation protein Mcl-1 | MCL1 |
| *Lonicerae Japonicae Flos* | Jinyinhua | MOL000006 | E3 ubiquitin-protein ligase Mdm2 | MDM2 |
| *Lonicerae Japonicae Flos* | Jinyinhua | MOL000006 | Hepatocyte growth factor receptor | MET |
| *Lonicerae Japonicae Flos* | Jinyinhua | MOL000098 | Maltase-glucoamylase, intestinal | MGAM |
| *Lonicerae Japonicae Flos* | Jinyinhua | MOL002773 | Interstitial collagenase | MMP1 |
| *Lonicerae Japonicae Flos* | Jinyinhua | MOL000422 | Interstitial collagenase | MMP1 |
| *Lonicerae Japonicae Flos* | Jinyinhua | MOL000006 | Interstitial collagenase | MMP1 |
| *Lonicerae Japonicae Flos* | Jinyinhua | MOL000098 | Interstitial collagenase | MMP1 |
| *Lonicerae Japonicae Flos* | Jinyinhua | MOL002773 | Stromelysin-2 | MMP10 |
| *Lonicerae Japonicae Flos* | Jinyinhua | MOL002773 | 72 kDa type IV collagenase | MMP2 |
| *Lonicerae Japonicae Flos* | Jinyinhua | MOL000006 | 72 kDa type IV collagenase | MMP2 |
| *Lonicerae Japonicae Flos* | Jinyinhua | MOL000098 | 72 kDa type IV collagenase | MMP2 |
| *Lonicerae Japonicae Flos* | Jinyinhua | MOL000098 | Stromelysin-1 | MMP3 |
| *Lonicerae Japonicae Flos* | Jinyinhua | MOL000006 | Matrix metalloproteinase-9 | MMP9 |
| *Lonicerae Japonicae Flos* | Jinyinhua | MOL000098 | Matrix metalloproteinase-9 | MMP9 |
| *Lonicerae Japonicae Flos* | Jinyinhua | MOL000098 | Myeloperoxidase | MPO |
| *Lonicerae Japonicae Flos* | Jinyinhua | MOL002773 | Myc proto-oncogene protein | MYC |
| *Lonicerae Japonicae Flos* | Jinyinhua | MOL000098 | Myc proto-oncogene protein | MYC |
| *Lonicerae Japonicae Flos* | Jinyinhua | MOL000098 | Neutrophil cytosol factor 1 | NCF1 |
| *Lonicerae Japonicae Flos* | Jinyinhua | MOL003044 | Nuclear receptor coactivator 1 | NCOA1 |
| *Lonicerae Japonicae Flos* | Jinyinhua | MOL003095 | Nuclear receptor coactivator 1 | NCOA1 |
| *Lonicerae Japonicae Flos* | Jinyinhua | MOL003111 | Nuclear receptor coactivator 1 | NCOA1 |
| *Lonicerae Japonicae Flos* | Jinyinhua | MOL000449 | Nuclear receptor coactivator 1 | NCOA1 |
| *Lonicerae Japonicae Flos* | Jinyinhua | MOL001494 | Nuclear receptor coactivator 2 | NCOA2 |
| *Lonicerae Japonicae Flos* | Jinyinhua | MOL001495 | Nuclear receptor coactivator 2 | NCOA2 |
| *Lonicerae Japonicae Flos* | Jinyinhua | MOL002914 | Nuclear receptor coactivator 2 | NCOA2 |
| *Lonicerae Japonicae Flos* | Jinyinhua | MOL003036 | Nuclear receptor coactivator 2 | NCOA2 |
| *Lonicerae Japonicae Flos* | Jinyinhua | MOL003044 | Nuclear receptor coactivator 2 | NCOA2 |
| *Lonicerae Japonicae Flos* | Jinyinhua | MOL003095 | Nuclear receptor coactivator 2 | NCOA2 |
| *Lonicerae Japonicae Flos* | Jinyinhua | MOL003111 | Nuclear receptor coactivator 2 | NCOA2 |
| *Lonicerae Japonicae Flos* | Jinyinhua | MOL000358 | Nuclear receptor coactivator 2 | NCOA2 |
| *Lonicerae Japonicae Flos* | Jinyinhua | MOL000422 | Nuclear receptor coactivator 2 | NCOA2 |
| *Lonicerae Japonicae Flos* | Jinyinhua | MOL000449 | Nuclear receptor coactivator 2 | NCOA2 |
| *Lonicerae Japonicae Flos* | Jinyinhua | MOL000006 | Nuclear receptor coactivator 2 | NCOA2 |
| *Lonicerae Japonicae Flos* | Jinyinhua | MOL000098 | Nuclear receptor coactivator 2 | NCOA2 |
| *Lonicerae Japonicae Flos* | Jinyinhua | MOL000098 | Nuclear factor erythroid 2-related factor 2 | NFE2L2 |
| *Lonicerae Japonicae Flos* | Jinyinhua | MOL000006 | NF-kappa-B inhibitor alpha | NFKBIA |
| *Lonicerae Japonicae Flos* | Jinyinhua | MOL000098 | NF-kappa-B inhibitor alpha | NFKBIA |
| *Lonicerae Japonicae Flos* | Jinyinhua | MOL000098 | Homeobox protein Nkx-3.1 | NKX3-1 |
| *Lonicerae Japonicae Flos* | Jinyinhua | MOL003044 | Nitric oxide synthase, inducible | NOS2 |
| *Lonicerae Japonicae Flos* | Jinyinhua | MOL003095 | Nitric oxide synthase, inducible | NOS2 |
| *Lonicerae Japonicae Flos* | Jinyinhua | MOL003117 | Nitric oxide synthase, inducible | NOS2 |
| *Lonicerae Japonicae Flos* | Jinyinhua | MOL000422 | Nitric oxide synthase, inducible | NOS2 |
| *Lonicerae Japonicae Flos* | Jinyinhua | MOL003095 | Nitric-oxide synthase, endothelial | NOS3 |
| *Lonicerae Japonicae Flos* | Jinyinhua | MOL000422 | Nitric-oxide synthase, endothelial | NOS3 |
| *Lonicerae Japonicae Flos* | Jinyinhua | MOL000098 | Nitric-oxide synthase, endothelial | NOS3 |
| *Lonicerae Japonicae Flos* | Jinyinhua | MOL000098 | Nitric oxide synthase, endothelial | NOS3 |
| *Lonicerae Japonicae Flos* | Jinyinhua | MOL000098 | Puromycin-sensitive aminopeptidase | NPEPPS |
| *Lonicerae Japonicae Flos* | Jinyinhua | MOL000098 | NAD(P)H dehydrogenase [quinone] 1 | NQO1 |
| *Lonicerae Japonicae Flos* | Jinyinhua | MOL000422 | Nuclear receptor subfamily 1 group I member 2 | NR1I2 |
| *Lonicerae Japonicae Flos* | Jinyinhua | MOL000098 | Nuclear receptor subfamily 1 group I member 2 | NR1I2 |
| *Lonicerae Japonicae Flos* | Jinyinhua | MOL000422 | Nuclear receptor subfamily 1 group I member 3 | NR1I3 |
| *Lonicerae Japonicae Flos* | Jinyinhua | MOL000098 | Nuclear receptor subfamily 1 group I member 3 | NR1I3 |
| *Lonicerae Japonicae Flos* | Jinyinhua | MOL003036 | Mineralocorticoid receptor | NR3C2 |
| *Lonicerae Japonicae Flos* | Jinyinhua | MOL000449 | Mineralocorticoid receptor | NR3C2 |
| *Lonicerae Japonicae Flos* | Jinyinhua | MOL000006 | Kinetochore protein Nuf2 | NUF2 |
| *Lonicerae Japonicae Flos* | Jinyinhua | MOL000098 | Ornithine decarboxylase | ODC1 |
| *Lonicerae Japonicae Flos* | Jinyinhua | MOL000358 | Mu-type opioid receptor | OPRM1 |
| *Lonicerae Japonicae Flos* | Jinyinhua | MOL000098 | Poly [ADP-ribose] polymerase 1 | PARP1 |
| *Lonicerae Japonicae Flos* | Jinyinhua | MOL000006 | Proliferating cell nuclear antigen | PCNA |
| *Lonicerae Japonicae Flos* | Jinyinhua | MOL000098 | Procollagen C-endopeptidase enhancer 1 | PCOLCE |
| *Lonicerae Japonicae Flos* | Jinyinhua | MOL000358 | CGMP-inhibited 3',5'-cyclic phosphodiesterase A | PDE3A |
| *Lonicerae Japonicae Flos* | Jinyinhua | MOL003036 | Progesterone receptor | PGR |
| *Lonicerae Japonicae Flos* | Jinyinhua | MOL000358 | Progesterone receptor | PGR |
| *Lonicerae Japonicae Flos* | Jinyinhua | MOL000422 | Progesterone receptor | PGR |
| *Lonicerae Japonicae Flos* | Jinyinhua | MOL000449 | Progesterone receptor | PGR |
| *Lonicerae Japonicae Flos* | Jinyinhua | MOL002914 | Phosphatidylinositol-4,5-bisphosphate 3-kinase catalytic subunit, gamma isoform | PIK3CG |
| *Lonicerae Japonicae Flos* | Jinyinhua | MOL003044 | Phosphatidylinositol-4,5-bisphosphate 3-kinase catalytic subunit, gamma isoform | PIK3CG |
| *Lonicerae Japonicae Flos* | Jinyinhua | MOL000358 | Phosphatidylinositol-4,5-bisphosphate 3-kinase catalytic subunit, gamma isoform | PIK3CG |
| *Lonicerae Japonicae Flos* | Jinyinhua | MOL000422 | Phosphatidylinositol-4,5-bisphosphate 3-kinase catalytic subunit, gamma isoform | PIK3CG |
| *Lonicerae Japonicae Flos* | Jinyinhua | MOL000006 | Phosphatidylinositol-4,5-bisphosphate 3-kinase catalytic subunit, gamma isoform | PIK3CG |
| *Lonicerae Japonicae Flos* | Jinyinhua | MOL000098 | Phosphatidylinositol-4,5-bisphosphate 3-kinase catalytic subunit, gamma isoform | PIK3CG |
| *Lonicerae Japonicae Flos* | Jinyinhua | MOL000098 | Tissue-type plasminogen activator | PLAT |
| *Lonicerae Japonicae Flos* | Jinyinhua | MOL000449 | Urokinase-type plasminogen activator | PLAU |
| *Lonicerae Japonicae Flos* | Jinyinhua | MOL000098 | Urokinase-type plasminogen activator | PLAU |
| *Lonicerae Japonicae Flos* | Jinyinhua | MOL000358 | Serum paraoxonase/arylesterase 1 | PON1 |
| *Lonicerae Japonicae Flos* | Jinyinhua | MOL000098 | Serum paraoxonase/arylesterase 1 | PON1 |
| *Lonicerae Japonicae Flos* | Jinyinhua | MOL000098 | NADPH--cytochrome P450 reductase | POR |
| *Lonicerae Japonicae Flos* | Jinyinhua | MOL000098 | Peroxisome proliferator-activated receptor alpha | PPARA |
| *Lonicerae Japonicae Flos* | Jinyinhua | MOL000098 | Peroxisome proliferator-activated receptor delta | PPARD |
| *Lonicerae Japonicae Flos* | Jinyinhua | MOL003044 | Peroxisome proliferator activated receptor gamma | PPARG |
| *Lonicerae Japonicae Flos* | Jinyinhua | MOL003095 | Peroxisome proliferator activated receptor gamma | PPARG |
| *Lonicerae Japonicae Flos* | Jinyinhua | MOL000422 | Peroxisome proliferator activated receptor gamma | PPARG |
| *Lonicerae Japonicae Flos* | Jinyinhua | MOL000422 | Peroxisome proliferator-activated receptor gamma | PPARG |
| *Lonicerae Japonicae Flos* | Jinyinhua | MOL000006 | Peroxisome proliferator-activated receptor gamma | PPARG |
| *Lonicerae Japonicae Flos* | Jinyinhua | MOL000098 | Peroxisome proliferator activated receptor gamma | PPARG |
| *Lonicerae Japonicae Flos* | Jinyinhua | MOL000098 | Peroxisome proliferator-activated receptor gamma | PPARG |
| *Lonicerae Japonicae Flos* | Jinyinhua | MOL000422 | Serine/threonine-protein phosphatase 2B catalytic subunit alpha isoform | PPP3CA |
| *Lonicerae Japonicae Flos* | Jinyinhua | MOL000358 | Protein kinase C alpha type | PRKCA |
| *Lonicerae Japonicae Flos* | Jinyinhua | MOL000098 | Protein kinase C alpha type | PRKCA |
| *Lonicerae Japonicae Flos* | Jinyinhua | MOL000098 | Protein kinase C beta type | PRKCB |
| *Lonicerae Japonicae Flos* | Jinyinhua | MOL003044 | Trypsin-1 | PRSS1 |
| *Lonicerae Japonicae Flos* | Jinyinhua | MOL003095 | Trypsin-1 | PRSS1 |
| *Lonicerae Japonicae Flos* | Jinyinhua | MOL003111 | Trypsin-1 | PRSS1 |
| *Lonicerae Japonicae Flos* | Jinyinhua | MOL000422 | Trypsin-1 | PRSS1 |
| *Lonicerae Japonicae Flos* | Jinyinhua | MOL000006 | Trypsin-1 | PRSS1 |
| *Lonicerae Japonicae Flos* | Jinyinhua | MOL000098 | Trypsin-1 | PRSS1 |
| *Lonicerae Japonicae Flos* | Jinyinhua | MOL000422 | 26S proteasome non-ATPase regulatory subunit 3 | PSMD3 |
| *Lonicerae Japonicae Flos* | Jinyinhua | MOL000098 | 26S proteasome non-ATPase regulatory subunit 3 | PSMD3 |
| *Lonicerae Japonicae Flos* | Jinyinhua | MOL000098 | Phosphatidylinositol-3,4,5-trisphosphate 3-phosphatase and dual-specificity protein phosphatase PTEN | PTEN |
| *Lonicerae Japonicae Flos* | Jinyinhua | MOL000098 | Prostaglandin E2 receptor EP3 subtype | PTGER3 |
| *Lonicerae Japonicae Flos* | Jinyinhua | MOL000006 | Prostaglandin E synthase | PTGES |
| *Lonicerae Japonicae Flos* | Jinyinhua | MOL001494 | Prostaglandin G/H synthase 1 | PTGS1 |
| *Lonicerae Japonicae Flos* | Jinyinhua | MOL001495 | Prostaglandin G/H synthase 1 | PTGS1 |
| *Lonicerae Japonicae Flos* | Jinyinhua | MOL002914 | Prostaglandin G/H synthase 1 | PTGS1 |
| *Lonicerae Japonicae Flos* | Jinyinhua | MOL003044 | Prostaglandin G/H synthase 1 | PTGS1 |
| *Lonicerae Japonicae Flos* | Jinyinhua | MOL003095 | Prostaglandin G/H synthase 1 | PTGS1 |
| *Lonicerae Japonicae Flos* | Jinyinhua | MOL000358 | Prostaglandin G/H synthase 1 | PTGS1 |
| *Lonicerae Japonicae Flos* | Jinyinhua | MOL000422 | Prostaglandin G/H synthase 1 | PTGS1 |
| *Lonicerae Japonicae Flos* | Jinyinhua | MOL000449 | Prostaglandin G/H synthase 1 | PTGS1 |
| *Lonicerae Japonicae Flos* | Jinyinhua | MOL000006 | Prostaglandin G/H synthase 1 | PTGS1 |
| *Lonicerae Japonicae Flos* | Jinyinhua | MOL000098 | Prostaglandin G/H synthase 1 | PTGS1 |
| *Lonicerae Japonicae Flos* | Jinyinhua | MOL001494 | Prostaglandin G/H synthase 2 | PTGS2 |
| *Lonicerae Japonicae Flos* | Jinyinhua | MOL002914 | Prostaglandin G/H synthase 2 | PTGS2 |
| *Lonicerae Japonicae Flos* | Jinyinhua | MOL003006 | Prostaglandin G/H synthase 2 | PTGS2 |
| *Lonicerae Japonicae Flos* | Jinyinhua | MOL003014 | Prostaglandin G/H synthase 2 | PTGS2 |
| *Lonicerae Japonicae Flos* | Jinyinhua | MOL002773 | Prostaglandin G/H synthase 2 | PTGS2 |
| *Lonicerae Japonicae Flos* | Jinyinhua | MOL003044 | Prostaglandin G/H synthase 2 | PTGS2 |
| *Lonicerae Japonicae Flos* | Jinyinhua | MOL003095 | Prostaglandin G/H synthase 2 | PTGS2 |
| *Lonicerae Japonicae Flos* | Jinyinhua | MOL003117 | Prostaglandin G/H synthase 2 | PTGS2 |
| *Lonicerae Japonicae Flos* | Jinyinhua | MOL000358 | Prostaglandin G/H synthase 2 | PTGS2 |
| *Lonicerae Japonicae Flos* | Jinyinhua | MOL000422 | Prostaglandin G/H synthase 2 | PTGS2 |
| *Lonicerae Japonicae Flos* | Jinyinhua | MOL000449 | Prostaglandin G/H synthase 2 | PTGS2 |
| *Lonicerae Japonicae Flos* | Jinyinhua | MOL000006 | Prostaglandin G/H synthase 2 | PTGS2 |
| *Lonicerae Japonicae Flos* | Jinyinhua | MOL000098 | Prostaglandin G/H synthase 2 | PTGS2 |
| *Lonicerae Japonicae Flos* | Jinyinhua | MOL002914 | Glycogen phosphorylase, muscle form | PYGM |
| *Lonicerae Japonicae Flos* | Jinyinhua | MOL000098 | RAF proto-oncogene serine/threonine-protein kinase | RAF1 |
| *Lonicerae Japonicae Flos* | Jinyinhua | MOL000098 | Ras GTPase-activating protein 1 | RASA1 |
| *Lonicerae Japonicae Flos* | Jinyinhua | MOL000098 | Ras association domain-containing protein 1 | RASSF1 |
| *Lonicerae Japonicae Flos* | Jinyinhua | MOL000006 | Retinoblastoma-associated protein | RB1 |
| *Lonicerae Japonicae Flos* | Jinyinhua | MOL000098 | Retinoblastoma-associated protein | RB1 |
| *Lonicerae Japonicae Flos* | Jinyinhua | MOL000422 | Transcription factor p65 | RELA |
| *Lonicerae Japonicae Flos* | Jinyinhua | MOL000006 | Transcription factor p65 | RELA |
| *Lonicerae Japonicae Flos* | Jinyinhua | MOL000098 | Transcription factor p65 | RELA |
| *Lonicerae Japonicae Flos* | Jinyinhua | MOL000098 | Protein CBFA2T1 | RUNX1T1 |
| *Lonicerae Japonicae Flos* | Jinyinhua | MOL000098 | Runt-related transcription factor 2 | RUNX2 |
| *Lonicerae Japonicae Flos* | Jinyinhua | MOL000449 | Retinoic acid receptor RXR-alpha | RXRA |
| *Lonicerae Japonicae Flos* | Jinyinhua | MOL000098 | Retinoic acid receptor RXR-alpha | RXRA |
| *Lonicerae Japonicae Flos* | Jinyinhua | MOL003095 | Sodium channel protein type 5 subunit alpha | SCN5A |
| *Lonicerae Japonicae Flos* | Jinyinhua | MOL000358 | Sodium channel protein type 5 subunit alpha | SCN5A |
| *Lonicerae Japonicae Flos* | Jinyinhua | MOL000449 | Sodium channel protein type 5 subunit alpha | SCN5A |
| *Lonicerae Japonicae Flos* | Jinyinhua | MOL000098 | Sodium channel protein type 5 subunit alpha | SCN5A |
| *Lonicerae Japonicae Flos* | Jinyinhua | MOL000422 | E-selectin | SELE |
| *Lonicerae Japonicae Flos* | Jinyinhua | MOL000098 | E-selectin | SELE |
| *Lonicerae Japonicae Flos* | Jinyinhua | MOL000098 | Plasminogen activator inhibitor 1 | SERPINE1 |
| *Lonicerae Japonicae Flos* | Jinyinhua | MOL000422 | Solute carrier family 2, facilitated glucose transporter member 4 | SLC2A4 |
| *Lonicerae Japonicae Flos* | Jinyinhua | MOL000006 | Solute carrier family 2, facilitated glucose transporter member 4 | SLC2A4 |
| *Lonicerae Japonicae Flos* | Jinyinhua | MOL000098 | Solute carrier family 2, facilitated glucose transporter member 4 | SLC2A4 |
| *Lonicerae Japonicae Flos* | Jinyinhua | MOL000422 | Sodium-dependent noradrenaline transporter | SLC6A2 |
| *Lonicerae Japonicae Flos* | Jinyinhua | MOL000449 | Sodium-dependent noradrenaline transporter | SLC6A2 |
| *Lonicerae Japonicae Flos* | Jinyinhua | MOL000449 | Sodium-dependent dopamine transporter | SLC6A3 |
| *Lonicerae Japonicae Flos* | Jinyinhua | MOL000358 | Sodium-dependent serotonin transporter | SLC6A4 |
| *Lonicerae Japonicae Flos* | Jinyinhua | MOL000422 | Antileukoproteinase | SLPI |
| *Lonicerae Japonicae Flos* | Jinyinhua | MOL000098 | Superoxide dismutase [Cu-Zn] | SOD1 |
| *Lonicerae Japonicae Flos* | Jinyinhua | MOL000098 | Osteopontin | SPP1 |
| *Lonicerae Japonicae Flos* | Jinyinhua | MOL000422 | Signal transducer and activator of transcription 1-alpha/beta | STAT1 |
| *Lonicerae Japonicae Flos* | Jinyinhua | MOL000098 | Signal transducer and activator of transcription 1-alpha/beta | STAT1 |
| *Lonicerae Japonicae Flos* | Jinyinhua | MOL000098 | Estrogen sulfotransferase | SULT1E1 |
| *Lonicerae Japonicae Flos* | Jinyinhua | MOL000358 | Transforming growth factor beta-1 | TGFB1 |
| *Lonicerae Japonicae Flos* | Jinyinhua | MOL000098 | Transforming growth factor beta-1 | TGFB1 |
| *Lonicerae Japonicae Flos* | Jinyinhua | MOL000098 | Thrombomodulin | THBD |
| *Lonicerae Japonicae Flos* | Jinyinhua | MOL000422 | Tumor necrosis factor | TNF |
| *Lonicerae Japonicae Flos* | Jinyinhua | MOL000006 | Tumor necrosis factor | TNF |
| *Lonicerae Japonicae Flos* | Jinyinhua | MOL000098 | Tumor necrosis factor | TNF |
| *Lonicerae Japonicae Flos* | Jinyinhua | MOL000006 | DNA topoisomerase 1 | TOP1 |
| *Lonicerae Japonicae Flos* | Jinyinhua | MOL000098 | DNA topoisomerase 1 | TOP1 |
| *Lonicerae Japonicae Flos* | Jinyinhua | MOL000006 | DNA topoisomerase 2-alpha | TOP2A |
| *Lonicerae Japonicae Flos* | Jinyinhua | MOL000098 | DNA topoisomerase 2-alpha | TOP2A |
| *Lonicerae Japonicae Flos* | Jinyinhua | MOL003095 | DNA topoisomerase II | TOP2B |
| *Lonicerae Japonicae Flos* | Jinyinhua | MOL000422 | DNA topoisomerase II | TOP2B |
| *Lonicerae Japonicae Flos* | Jinyinhua | MOL000098 | DNA topoisomerase II | TOP2B |
| *Lonicerae Japonicae Flos* | Jinyinhua | MOL000006 | Cellular tumor antigen p53 | TP53 |
| *Lonicerae Japonicae Flos* | Jinyinhua | MOL000098 | Cellular tumor antigen p53 | TP53 |
| *Lonicerae Japonicae Flos* | Jinyinhua | MOL000006 | Tyrosinase | TYR |
| *Lonicerae Japonicae Flos* | Jinyinhua | MOL000422 | Vascular cell adhesion protein 1 | VCAM1 |
| *Lonicerae Japonicae Flos* | Jinyinhua | MOL000098 | Vascular cell adhesion protein 1 | VCAM1 |
| *Lonicerae Japonicae Flos* | Jinyinhua | MOL002773 | Vascular endothelial growth factor A | VEGFA |
| *Lonicerae Japonicae Flos* | Jinyinhua | MOL000006 | Vascular endothelial growth factor A | VEGFA |
| *Lonicerae Japonicae Flos* | Jinyinhua | MOL000098 | Vascular endothelial growth factor A | VEGFA |
| *Lonicerae Japonicae Flos* | Jinyinhua | MOL000422 | Xanthine dehydrogenase/oxidase | XDH |
| *Lonicerae Japonicae Flos* | Jinyinhua | MOL000006 | Xanthine dehydrogenase/oxidase | XDH |
| *Lonicerae Japonicae Flos* | Jinyinhua | MOL000098 | Xanthine dehydrogenase/oxidase | XDH |
| *Lonicerae Japonicae Flos* | Jinyinhua | MOL000006 | Baculoviral IAP repeat-containing protein 4 | XIAP |
| *Hedysarum Multijugum Maxim* | Huangqi | MOL000098 | ATP-binding cassette sub-family G member 2 | ABCG2 |
| *Hedysarum Multijugum Maxim* | Huangqi | MOL000098 | Acetyl-CoA carboxylase 1 | ACACA |
| *Hedysarum Multijugum Maxim* | Huangqi | MOL000354 | Acetylcholinesterase | ACHE |
| *Hedysarum Multijugum Maxim* | Huangqi | MOL000371 | Acetylcholinesterase | ACHE |
| *Hedysarum Multijugum Maxim* | Huangqi | MOL000380 | Acetylcholinesterase | ACHE |
| *Hedysarum Multijugum Maxim* | Huangqi | MOL000392 | Acetylcholinesterase | ACHE |
| *Hedysarum Multijugum Maxim* | Huangqi | MOL000422 | Acetylcholinesterase | ACHE |
| *Hedysarum Multijugum Maxim* | Huangqi | MOL000098 | Acetylcholinesterase | ACHE |
| *Hedysarum Multijugum Maxim* | Huangqi | MOL000098 | Prostatic acid phosphatase | ACP3 |
| *Hedysarum Multijugum Maxim* | Huangqi | MOL000296 | Alcohol dehydrogenase 1B | ADH1B |
| *Hedysarum Multijugum Maxim* | Huangqi | MOL000296 | Alcohol dehydrogenase 1C | ADH1C |
| *Hedysarum Multijugum Maxim* | Huangqi | MOL000378 | Alpha-1A adrenergic receptor | ADRA1A |
| *Hedysarum Multijugum Maxim* | Huangqi | MOL000392 | Alpha-1A adrenergic receptor | ADRA1A |
| *Hedysarum Multijugum Maxim* | Huangqi | MOL000296 | Alpha-1B adrenergic receptor | ADRA1B |
| *Hedysarum Multijugum Maxim* | Huangqi | MOL000371 | Alpha-1B adrenergic receptor | ADRA1B |
| *Hedysarum Multijugum Maxim* | Huangqi | MOL000378 | Alpha-1B adrenergic receptor | ADRA1B |
| *Hedysarum Multijugum Maxim* | Huangqi | MOL000380 | Alpha-1B adrenergic receptor | ADRA1B |
| *Hedysarum Multijugum Maxim* | Huangqi | MOL000422 | Alpha-1B adrenergic receptor | ADRA1B |
| *Hedysarum Multijugum Maxim* | Huangqi | MOL000371 | Alpha-1D adrenergic receptor | ADRA1D |
| *Hedysarum Multijugum Maxim* | Huangqi | MOL000378 | Alpha-1D adrenergic receptor | ADRA1D |
| *Hedysarum Multijugum Maxim* | Huangqi | MOL000380 | Alpha-1D adrenergic receptor | ADRA1D |
| *Hedysarum Multijugum Maxim* | Huangqi | MOL000371 | Alpha-2C adrenergic receptor | ADRA2C |
| *Hedysarum Multijugum Maxim* | Huangqi | MOL000378 | Alpha-2C adrenergic receptor | ADRA2C |
| *Hedysarum Multijugum Maxim* | Huangqi | MOL000371 | Beta-1 adrenergic receptor | ADRB1 |
| *Hedysarum Multijugum Maxim* | Huangqi | MOL000378 | Beta-1 adrenergic receptor | ADRB1 |
| *Hedysarum Multijugum Maxim* | Huangqi | MOL000371 | Beta-2 adrenergic receptor | ADRB2 |
| *Hedysarum Multijugum Maxim* | Huangqi | MOL000378 | Beta-2 adrenergic receptor | ADRB2 |
| *Hedysarum Multijugum Maxim* | Huangqi | MOL000380 | Beta-2 adrenergic receptor | ADRB2 |
| *Hedysarum Multijugum Maxim* | Huangqi | MOL000392 | Beta-2 adrenergic receptor | ADRB2 |
| *Hedysarum Multijugum Maxim* | Huangqi | MOL000417 | Beta-2 adrenergic receptor | ADRB2 |
| *Hedysarum Multijugum Maxim* | Huangqi | MOL000098 | Beta-2 adrenergic receptor | ADRB2 |
| *Hedysarum Multijugum Maxim* | Huangqi | MOL000422 | Aryl hydrocarbon receptor | AHR |
| *Hedysarum Multijugum Maxim* | Huangqi | MOL000098 | Aryl hydrocarbon receptor | AHR |
| *Hedysarum Multijugum Maxim* | Huangqi | MOL000422 | Activator of 90 kDa heat shock protein ATPase homolog 1 | AHSA1 |
| *Hedysarum Multijugum Maxim* | Huangqi | MOL000098 | Activator of 90 kDa heat shock protein ATPase homolog 1 | AHSA1 |
| *Hedysarum Multijugum Maxim* | Huangqi | MOL000354 | Aldose reductase | AKR1B1 |
| *Hedysarum Multijugum Maxim* | Huangqi | MOL000098 | Aldose reductase | AKR1B1 |
| *Hedysarum Multijugum Maxim* | Huangqi | MOL000422 | Aldo-keto reductase family 1 member C3 | AKR1C3 |
| *Hedysarum Multijugum Maxim* | Huangqi | MOL000422 | RAC-alpha serine/threonine-protein kinase | AKT1 |
| *Hedysarum Multijugum Maxim* | Huangqi | MOL000098 | RAC-alpha serine/threonine-protein kinase | AKT1 |
| *Hedysarum Multijugum Maxim* | Huangqi | MOL000422 | Arachidonate 5-lipoxygenase | ALOX5 |
| *Hedysarum Multijugum Maxim* | Huangqi | MOL000098 | Arachidonate 5-lipoxygenase | ALOX5 |
| *Hedysarum Multijugum Maxim* | Huangqi | MOL000239 | Androgen receptor | AR |
| *Hedysarum Multijugum Maxim* | Huangqi | MOL000354 | Androgen receptor | AR |
| *Hedysarum Multijugum Maxim* | Huangqi | MOL000378 | Androgen receptor | AR |
| *Hedysarum Multijugum Maxim* | Huangqi | MOL000392 | Androgen receptor | AR |
| *Hedysarum Multijugum Maxim* | Huangqi | MOL000417 | Androgen receptor | AR |
| *Hedysarum Multijugum Maxim* | Huangqi | MOL000422 | Androgen receptor | AR |
| *Hedysarum Multijugum Maxim* | Huangqi | MOL000098 | Androgen receptor | AR |
| *Hedysarum Multijugum Maxim* | Huangqi | MOL000392 | ATP synthase subunit beta, mitochondrial | ATP5F1B |
| *Hedysarum Multijugum Maxim* | Huangqi | MOL000422 | Apoptosis regulator BAX | BAX |
| *Hedysarum Multijugum Maxim* | Huangqi | MOL000098 | Apoptosis regulator BAX | BAX |
| *Hedysarum Multijugum Maxim* | Huangqi | MOL000422 | Apoptosis regulator Bcl-2 | BCL2 |
| *Hedysarum Multijugum Maxim* | Huangqi | MOL000098 | Apoptosis regulator Bcl-2 | BCL2 |
| *Hedysarum Multijugum Maxim* | Huangqi | MOL000098 | Bcl-2-like protein 1 | BCL2L1 |
| *Hedysarum Multijugum Maxim* | Huangqi | MOL000098 | Baculoviral IAP repeat-containing protein 5 | BIRC5 |
| *Hedysarum Multijugum Maxim* | Huangqi | MOL000239 | Calmodulin | CALM1 |
| *Hedysarum Multijugum Maxim* | Huangqi | MOL000354 | Calmodulin | CALM1 |
| *Hedysarum Multijugum Maxim* | Huangqi | MOL000371 | Calmodulin | CALM1 |
| *Hedysarum Multijugum Maxim* | Huangqi | MOL000378 | Calmodulin | CALM1 |
| *Hedysarum Multijugum Maxim* | Huangqi | MOL000380 | Calmodulin | CALM1 |
| *Hedysarum Multijugum Maxim* | Huangqi | MOL000392 | Calmodulin | CALM1 |
| *Hedysarum Multijugum Maxim* | Huangqi | MOL000417 | Calmodulin | CALM1 |
| *Hedysarum Multijugum Maxim* | Huangqi | MOL000422 | Calmodulin | CALM1 |
| *Hedysarum Multijugum Maxim* | Huangqi | MOL000422 | Caspase-3 | CASP3 |
| *Hedysarum Multijugum Maxim* | Huangqi | MOL000098 | Caspase-3 | CASP3 |
| *Hedysarum Multijugum Maxim* | Huangqi | MOL000098 | Caspase-8 | CASP8 |
| *Hedysarum Multijugum Maxim* | Huangqi | MOL000098 | Caspase-9 | CASP9 |
| *Hedysarum Multijugum Maxim* | Huangqi | MOL000098 | Caveolin-1 | CAV1 |
| *Hedysarum Multijugum Maxim* | Huangqi | MOL000098 | C-C motif chemokine 2 | CCL2 |
| *Hedysarum Multijugum Maxim* | Huangqi | MOL000354 | Cyclin-A2 | CCNA2 |
| *Hedysarum Multijugum Maxim* | Huangqi | MOL000378 | Cyclin-A2 | CCNA2 |
| *Hedysarum Multijugum Maxim* | Huangqi | MOL000392 | Cyclin-A2 | CCNA2 |
| *Hedysarum Multijugum Maxim* | Huangqi | MOL000417 | Cyclin-A2 | CCNA2 |
| *Hedysarum Multijugum Maxim* | Huangqi | MOL000098 | G2/mitotic-specific cyclin-B1 | CCNB1 |
| *Hedysarum Multijugum Maxim* | Huangqi | MOL000098 | G1/S-specific cyclin-D1 | CCND1 |
| *Hedysarum Multijugum Maxim* | Huangqi | MOL000098 | CD40 ligand | CD40LG |
| *Hedysarum Multijugum Maxim* | Huangqi | MOL000422 | Cell division control protein 2 homolog | CDK1 |
| *Hedysarum Multijugum Maxim* | Huangqi | MOL000098 | Cell division control protein 2 homolog | CDK1 |
| *Hedysarum Multijugum Maxim* | Huangqi | MOL000239 | Cell division protein kinase 2 | CDK2 |
| *Hedysarum Multijugum Maxim* | Huangqi | MOL000354 | Cell division protein kinase 2 | CDK2 |
| *Hedysarum Multijugum Maxim* | Huangqi | MOL000378 | Cell division protein kinase 2 | CDK2 |
| *Hedysarum Multijugum Maxim* | Huangqi | MOL000392 | Cell division protein kinase 2 | CDK2 |
| *Hedysarum Multijugum Maxim* | Huangqi | MOL000417 | Cell division protein kinase 2 | CDK2 |
| *Hedysarum Multijugum Maxim* | Huangqi | MOL000433 | Cell division protein kinase 2 | CDK2 |
| *Hedysarum Multijugum Maxim* | Huangqi | MOL000098 | Cyclin-dependent kinase inhibitor 1 | CDKN1A |
| *Hedysarum Multijugum Maxim* | Huangqi | MOL000098 | Cyclin-dependent kinase inhibitor 2A, isoforms 1/2/3 | CDKN2A |
| *Hedysarum Multijugum Maxim* | Huangqi | MOL000239 | Serine/threonine-protein kinase Chk1 | CHEK1 |
| *Hedysarum Multijugum Maxim* | Huangqi | MOL000354 | Serine/threonine-protein kinase Chk1 | CHEK1 |
| *Hedysarum Multijugum Maxim* | Huangqi | MOL000378 | Serine/threonine-protein kinase Chk1 | CHEK1 |
| *Hedysarum Multijugum Maxim* | Huangqi | MOL000392 | Serine/threonine-protein kinase Chk1 | CHEK1 |
| *Hedysarum Multijugum Maxim* | Huangqi | MOL000417 | Serine/threonine-protein kinase Chk1 | CHEK1 |
| *Hedysarum Multijugum Maxim* | Huangqi | MOL000098 | Serine/threonine-protein kinase Chk2 | CHEK2 |
| *Hedysarum Multijugum Maxim* | Huangqi | MOL000296 | Muscarinic acetylcholine receptor M1 | CHRM1 |
| *Hedysarum Multijugum Maxim* | Huangqi | MOL000371 | Muscarinic acetylcholine receptor M1 | CHRM1 |
| *Hedysarum Multijugum Maxim* | Huangqi | MOL000378 | Muscarinic acetylcholine receptor M1 | CHRM1 |
| *Hedysarum Multijugum Maxim* | Huangqi | MOL000380 | Muscarinic acetylcholine receptor M1 | CHRM1 |
| *Hedysarum Multijugum Maxim* | Huangqi | MOL000392 | Muscarinic acetylcholine receptor M1 | CHRM1 |
| *Hedysarum Multijugum Maxim* | Huangqi | MOL000422 | Muscarinic acetylcholine receptor M1 | CHRM1 |
| *Hedysarum Multijugum Maxim* | Huangqi | MOL000296 | Muscarinic acetylcholine receptor M2 | CHRM2 |
| *Hedysarum Multijugum Maxim* | Huangqi | MOL000378 | Muscarinic acetylcholine receptor M2 | CHRM2 |
| *Hedysarum Multijugum Maxim* | Huangqi | MOL000422 | Muscarinic acetylcholine receptor M2 | CHRM2 |
| *Hedysarum Multijugum Maxim* | Huangqi | MOL000296 | Muscarinic acetylcholine receptor M3 | CHRM3 |
| *Hedysarum Multijugum Maxim* | Huangqi | MOL000371 | Muscarinic acetylcholine receptor M3 | CHRM3 |
| *Hedysarum Multijugum Maxim* | Huangqi | MOL000378 | Muscarinic acetylcholine receptor M3 | CHRM3 |
| *Hedysarum Multijugum Maxim* | Huangqi | MOL000380 | Muscarinic acetylcholine receptor M3 | CHRM3 |
| *Hedysarum Multijugum Maxim* | Huangqi | MOL000378 | Muscarinic acetylcholine receptor M4 | CHRM4 |
| *Hedysarum Multijugum Maxim* | Huangqi | MOL000380 | Muscarinic acetylcholine receptor M4 | CHRM4 |
| *Hedysarum Multijugum Maxim* | Huangqi | MOL000378 | Muscarinic acetylcholine receptor M5 | CHRM5 |
| *Hedysarum Multijugum Maxim* | Huangqi | MOL000380 | Neuronal acetylcholine receptor protein, alpha-7 chain | CHRNA7 |
| *Hedysarum Multijugum Maxim* | Huangqi | MOL000098 | Inhibitor of nuclear factor kappa-B kinase subunit alpha | CHUK |
| *Hedysarum Multijugum Maxim* | Huangqi | MOL000098 | Claudin-4 | CLDN4 |
| *Hedysarum Multijugum Maxim* | Huangqi | MOL000098 | Collagen alpha-1(I) chain | COL1A1 |
| *Hedysarum Multijugum Maxim* | Huangqi | MOL000098 | Collagen alpha-1(III) chain | COL3A1 |
| *Hedysarum Multijugum Maxim* | Huangqi | MOL000098 | C-reactive protein | CRP |
| *Hedysarum Multijugum Maxim* | Huangqi | MOL000098 | Cathepsin D | CTSD |
| *Hedysarum Multijugum Maxim* | Huangqi | MOL000098 | C-X-C motif chemokine 10 | CXCL10 |
| *Hedysarum Multijugum Maxim* | Huangqi | MOL000098 | C-X-C motif chemokine 11 | CXCL11 |
| *Hedysarum Multijugum Maxim* | Huangqi | MOL000098 | C-X-C motif chemokine 2 | CXCL2 |
| *Hedysarum Multijugum Maxim* | Huangqi | MOL000098 | Interleukin-8 | CXCL8 |
| *Hedysarum Multijugum Maxim* | Huangqi | MOL000422 | Cytochrome P450 1A1 | CYP1A1 |
| *Hedysarum Multijugum Maxim* | Huangqi | MOL000098 | Cytochrome P450 1A1 | CYP1A1 |
| *Hedysarum Multijugum Maxim* | Huangqi | MOL000422 | Cytochrome P450 1A2 | CYP1A2 |
| *Hedysarum Multijugum Maxim* | Huangqi | MOL000098 | Cytochrome P450 1A2 | CYP1A2 |
| *Hedysarum Multijugum Maxim* | Huangqi | MOL000422 | Cytochrome P450 1B1 | CYP1B1 |
| *Hedysarum Multijugum Maxim* | Huangqi | MOL000098 | Cytochrome P450 1B1 | CYP1B1 |
| *Hedysarum Multijugum Maxim* | Huangqi | MOL000422 | Cytochrome P450 3A4 | CYP3A4 |
| *Hedysarum Multijugum Maxim* | Huangqi | MOL000098 | Cytochrome P450 3A4 | CYP3A4 |
| *Hedysarum Multijugum Maxim* | Huangqi | MOL000098 | DDB1- and CUL4-associated factor 5 | DCAF5 |
| *Hedysarum Multijugum Maxim* | Huangqi | MOL000422 | Type I iodothyronine deiodinase | DIO1 |
| *Hedysarum Multijugum Maxim* | Huangqi | MOL000098 | Type I iodothyronine deiodinase | DIO1 |
| *Hedysarum Multijugum Maxim* | Huangqi | MOL000239 | Dipeptidyl peptidase IV | DPP4 |
| *Hedysarum Multijugum Maxim* | Huangqi | MOL000354 | Dipeptidyl peptidase IV | DPP4 |
| *Hedysarum Multijugum Maxim* | Huangqi | MOL000378 | Dipeptidyl peptidase IV | DPP4 |
| *Hedysarum Multijugum Maxim* | Huangqi | MOL000392 | Dipeptidyl peptidase IV | DPP4 |
| *Hedysarum Multijugum Maxim* | Huangqi | MOL000417 | Dipeptidyl peptidase IV | DPP4 |
| *Hedysarum Multijugum Maxim* | Huangqi | MOL000422 | Dipeptidyl peptidase IV | DPP4 |
| *Hedysarum Multijugum Maxim* | Huangqi | MOL000098 | Dipeptidyl peptidase IV | DPP4 |
| *Hedysarum Multijugum Maxim* | Huangqi | MOL000378 | Dopamine D1 receptor | DRD1 |
| *Hedysarum Multijugum Maxim* | Huangqi | MOL000098 | Dual oxidase 2 | DUOX2 |
| *Hedysarum Multijugum Maxim* | Huangqi | MOL000098 | Transcription factor E2F1 | E2F1 |
| *Hedysarum Multijugum Maxim* | Huangqi | MOL000098 | Transcription factor E2F2 | E2F2 |
| *Hedysarum Multijugum Maxim* | Huangqi | MOL000098 | Pro-epidermal growth factor | EGF |
| *Hedysarum Multijugum Maxim* | Huangqi | MOL000098 | Epidermal growth factor receptor | EGFR |
| *Hedysarum Multijugum Maxim* | Huangqi | MOL000098 | Eukaryotic translation initiation factor 6 | EIF6 |
| *Hedysarum Multijugum Maxim* | Huangqi | MOL000098 | ETS domain-containing protein Elk-1 | ELK1 |
| *Hedysarum Multijugum Maxim* | Huangqi | MOL000098 | Receptor tyrosine-protein kinase erbB-2 | ERBB2 |
| *Hedysarum Multijugum Maxim* | Huangqi | MOL000098 | Receptor tyrosine-protein kinase erbB-3 | ERBB3 |
| *Hedysarum Multijugum Maxim* | Huangqi | MOL000354 | Estrogen receptor | ESR1 |
| *Hedysarum Multijugum Maxim* | Huangqi | MOL000371 | Estrogen receptor | ESR1 |
| *Hedysarum Multijugum Maxim* | Huangqi | MOL000378 | Estrogen receptor | ESR1 |
| *Hedysarum Multijugum Maxim* | Huangqi | MOL000380 | Estrogen receptor | ESR1 |
| *Hedysarum Multijugum Maxim* | Huangqi | MOL000392 | Estrogen receptor | ESR1 |
| *Hedysarum Multijugum Maxim* | Huangqi | MOL000417 | Estrogen receptor | ESR1 |
| *Hedysarum Multijugum Maxim* | Huangqi | MOL000239 | Estrogen receptor beta | ESR2 |
| *Hedysarum Multijugum Maxim* | Huangqi | MOL000354 | Estrogen receptor beta | ESR2 |
| *Hedysarum Multijugum Maxim* | Huangqi | MOL000378 | Estrogen receptor beta | ESR2 |
| *Hedysarum Multijugum Maxim* | Huangqi | MOL000392 | Estrogen receptor beta | ESR2 |
| *Hedysarum Multijugum Maxim* | Huangqi | MOL000417 | Estrogen receptor beta | ESR2 |
| *Hedysarum Multijugum Maxim* | Huangqi | MOL000378 | Coagulation factor Xa | F10 |
| *Hedysarum Multijugum Maxim* | Huangqi | MOL000098 | Coagulation factor Xa | F10 |
| *Hedysarum Multijugum Maxim* | Huangqi | MOL000354 | Thrombin | F2 |
| *Hedysarum Multijugum Maxim* | Huangqi | MOL000371 | Thrombin | F2 |
| *Hedysarum Multijugum Maxim* | Huangqi | MOL000378 | Thrombin | F2 |
| *Hedysarum Multijugum Maxim* | Huangqi | MOL000380 | Thrombin | F2 |
| *Hedysarum Multijugum Maxim* | Huangqi | MOL000392 | Thrombin | F2 |
| *Hedysarum Multijugum Maxim* | Huangqi | MOL000422 | Thrombin | F2 |
| *Hedysarum Multijugum Maxim* | Huangqi | MOL000433 | Thrombin | F2 |
| *Hedysarum Multijugum Maxim* | Huangqi | MOL000098 | Thrombin | F2 |
| *Hedysarum Multijugum Maxim* | Huangqi | MOL000098 | Tissue factor | F3 |
| *Hedysarum Multijugum Maxim* | Huangqi | MOL000354 | Coagulation factor VII | F7 |
| *Hedysarum Multijugum Maxim* | Huangqi | MOL000422 | Coagulation factor VII | F7 |
| *Hedysarum Multijugum Maxim* | Huangqi | MOL000098 | Coagulation factor VII | F7 |
| *Hedysarum Multijugum Maxim* | Huangqi | MOL000098 | Proto-oncogene c-Fos | FOS |
| *Hedysarum Multijugum Maxim* | Huangqi | MOL000296 | Gamma-aminobutyric acid receptor subunit alpha-1 | GABRA1 |
| *Hedysarum Multijugum Maxim* | Huangqi | MOL000354 | Gamma-aminobutyric acid receptor subunit alpha-1 | GABRA1 |
| *Hedysarum Multijugum Maxim* | Huangqi | MOL000371 | Gamma-aminobutyric acid receptor subunit alpha-1 | GABRA1 |
| *Hedysarum Multijugum Maxim* | Huangqi | MOL000378 | Gamma-aminobutyric acid receptor subunit alpha-1 | GABRA1 |
| *Hedysarum Multijugum Maxim* | Huangqi | MOL000380 | Gamma-aminobutyric acid receptor subunit alpha-1 | GABRA1 |
| *Hedysarum Multijugum Maxim* | Huangqi | MOL000422 | Gamma-aminobutyric acid receptor subunit alpha-1 | GABRA1 |
| *Hedysarum Multijugum Maxim* | Huangqi | MOL000098 | Gamma-aminobutyric acid receptor subunit alpha-1 | GABRA1 |
| *Hedysarum Multijugum Maxim* | Huangqi | MOL000296 | Gamma-aminobutyric-acid receptor alpha-2 subunit | GABRA2 |
| *Hedysarum Multijugum Maxim* | Huangqi | MOL000422 | Gamma-aminobutyric-acid receptor alpha-2 subunit | GABRA2 |
| *Hedysarum Multijugum Maxim* | Huangqi | MOL000296 | Gamma-aminobutyric-acid receptor alpha-3 subunit | GABRA3 |
| *Hedysarum Multijugum Maxim* | Huangqi | MOL000296 | Gamma-aminobutyric-acid receptor alpha-5 subunit | GABRA5 |
| *Hedysarum Multijugum Maxim* | Huangqi | MOL000296 | Gamma-aminobutyric-acid receptor subunit alpha-6 | GABRA6 |
| *Hedysarum Multijugum Maxim* | Huangqi | MOL000098 | Gap junction alpha-1 protein | GJA1 |
| *Hedysarum Multijugum Maxim* | Huangqi | MOL000296 | Glutamate receptor 2 | GRIA2 |
| *Hedysarum Multijugum Maxim* | Huangqi | MOL000354 | Glutamate receptor 2 | GRIA2 |
| *Hedysarum Multijugum Maxim* | Huangqi | MOL000354 | Glycogen synthase kinase-3 beta | GSK3B |
| *Hedysarum Multijugum Maxim* | Huangqi | MOL000378 | Glycogen synthase kinase-3 beta | GSK3B |
| *Hedysarum Multijugum Maxim* | Huangqi | MOL000392 | Glycogen synthase kinase-3 beta | GSK3B |
| *Hedysarum Multijugum Maxim* | Huangqi | MOL000417 | Glycogen synthase kinase-3 beta | GSK3B |
| *Hedysarum Multijugum Maxim* | Huangqi | MOL000433 | Glycogen synthase kinase-3 beta | GSK3B |
| *Hedysarum Multijugum Maxim* | Huangqi | MOL000422 | Glutathione S-transferase Mu 1 | GSTM1 |
| *Hedysarum Multijugum Maxim* | Huangqi | MOL000098 | Glutathione S-transferase Mu 1 | GSTM1 |
| *Hedysarum Multijugum Maxim* | Huangqi | MOL000422 | Glutathione S-transferase Mu 2 | GSTM2 |
| *Hedysarum Multijugum Maxim* | Huangqi | MOL000098 | Glutathione S-transferase Mu 2 | GSTM2 |
| *Hedysarum Multijugum Maxim* | Huangqi | MOL000422 | Glutathione S-transferase P | GSTP1 |
| *Hedysarum Multijugum Maxim* | Huangqi | MOL000098 | Glutathione S-transferase P | GSTP1 |
| *Hedysarum Multijugum Maxim* | Huangqi | MOL000422 | Hyaluronan synthase 2 | HAS2 |
| *Hedysarum Multijugum Maxim* | Huangqi | MOL000098 | Hyaluronan synthase 2 | HAS2 |
| *Hedysarum Multijugum Maxim* | Huangqi | MOL000098 | Probable E3 ubiquitin-protein ligase HERC5 | HERC5 |
| *Hedysarum Multijugum Maxim* | Huangqi | MOL000098 | Hypoxia-inducible factor 1-alpha | HIF1A |
| *Hedysarum Multijugum Maxim* | Huangqi | MOL000098 | Hexokinase-2 | HK2 |
| *Hedysarum Multijugum Maxim* | Huangqi | MOL000422 | Heme oxygenase 1 | HMOX1 |
| *Hedysarum Multijugum Maxim* | Huangqi | MOL000098 | Heme oxygenase 1 | HMOX1 |
| *Hedysarum Multijugum Maxim* | Huangqi | MOL000392 | 3 beta-hydroxysteroid dehydrogenase/Delta 5-->4-isomerase type 1 | HSD3B1 |
| *Hedysarum Multijugum Maxim* | Huangqi | MOL000392 | 3 beta-hydroxysteroid dehydrogenase/Delta 5-->4-isomerase type 2 | HSD3B2 |
| *Hedysarum Multijugum Maxim* | Huangqi | MOL000098 | Heat shock factor protein 1 | HSF1 |
| *Hedysarum Multijugum Maxim* | Huangqi | MOL000239 | Heat shock protein HSP 90 | HSP90AB1 |
| *Hedysarum Multijugum Maxim* | Huangqi | MOL000354 | Heat shock protein HSP 90 | HSP90AB1 |
| *Hedysarum Multijugum Maxim* | Huangqi | MOL000378 | Heat shock protein HSP 90 | HSP90AB1 |
| *Hedysarum Multijugum Maxim* | Huangqi | MOL000380 | Heat shock protein HSP 90 | HSP90AB1 |
| *Hedysarum Multijugum Maxim* | Huangqi | MOL000387 | Heat shock protein HSP 90 | HSP90AB1 |
| *Hedysarum Multijugum Maxim* | Huangqi | MOL000392 | Heat shock protein HSP 90 | HSP90AB1 |
| *Hedysarum Multijugum Maxim* | Huangqi | MOL000417 | Heat shock protein HSP 90 | HSP90AB1 |
| *Hedysarum Multijugum Maxim* | Huangqi | MOL000422 | Heat shock protein HSP 90 | HSP90AB1 |
| *Hedysarum Multijugum Maxim* | Huangqi | MOL000442 | Heat shock protein HSP 90 | HSP90AB1 |
| *Hedysarum Multijugum Maxim* | Huangqi | MOL000098 | Heat shock protein HSP 90 | HSP90AB1 |
| *Hedysarum Multijugum Maxim* | Huangqi | MOL000098 | 78 kDa glucose-regulated protein | HSPA5 |
| *Hedysarum Multijugum Maxim* | Huangqi | MOL000098 | Heat shock protein beta-1 | HSPB1 |
| *Hedysarum Multijugum Maxim* | Huangqi | MOL000378 | 5-hydroxytryptamine 2A receptor | HTR2A |
| *Hedysarum Multijugum Maxim* | Huangqi | MOL000371 | 5-hydroxytryptamine receptor 3A | HTR3A |
| *Hedysarum Multijugum Maxim* | Huangqi | MOL000380 | 5-hydroxytryptamine receptor 3A | HTR3A |
| *Hedysarum Multijugum Maxim* | Huangqi | MOL000422 | Intercellular adhesion molecule 1 | ICAM1 |
| *Hedysarum Multijugum Maxim* | Huangqi | MOL000098 | Intercellular adhesion molecule 1 | ICAM1 |
| *Hedysarum Multijugum Maxim* | Huangqi | MOL000098 | Interferon gamma | IFNG |
| *Hedysarum Multijugum Maxim* | Huangqi | MOL000098 | Insulin-like growth factor II | IGF2 |
| *Hedysarum Multijugum Maxim* | Huangqi | MOL000098 | Insulin-like growth factor-binding protein 3 | IGFBP3 |
| *Hedysarum Multijugum Maxim* | Huangqi | MOL000296 | Ig gamma-1 chain C region | IGHG1 |
| *Hedysarum Multijugum Maxim* | Huangqi | MOL000422 | Inhibitor of nuclear factor kappa-B kinase subunit beta | IKBKB |
| *Hedysarum Multijugum Maxim* | Huangqi | MOL000098 | Interleukin-10 | IL10 |
| *Hedysarum Multijugum Maxim* | Huangqi | MOL000098 | Interleukin-1 alpha | IL1A |
| *Hedysarum Multijugum Maxim* | Huangqi | MOL000098 | Interleukin-1 beta | IL1B |
| *Hedysarum Multijugum Maxim* | Huangqi | MOL000098 | Interleukin-2 | IL2 |
| *Hedysarum Multijugum Maxim* | Huangqi | MOL000392 | Interleukin-4 | IL4 |
| *Hedysarum Multijugum Maxim* | Huangqi | MOL000098 | Interleukin-6 | IL6 |
| *Hedysarum Multijugum Maxim* | Huangqi | MOL000422 | Insulin receptor | INSR |
| *Hedysarum Multijugum Maxim* | Huangqi | MOL000098 | Insulin receptor | INSR |
| *Hedysarum Multijugum Maxim* | Huangqi | MOL000098 | Interferon regulatory factor 1 | IRF1 |
| *Hedysarum Multijugum Maxim* | Huangqi | MOL000392 | Transcription factor AP-1 | JUN |
| *Hedysarum Multijugum Maxim* | Huangqi | MOL000422 | Transcription factor AP-1 | JUN |
| *Hedysarum Multijugum Maxim* | Huangqi | MOL000098 | Transcription factor AP-1 | JUN |
| *Hedysarum Multijugum Maxim* | Huangqi | MOL000378 | Potassium voltage-gated channel subfamily H member 2 | KCNH2 |
| *Hedysarum Multijugum Maxim* | Huangqi | MOL000098 | Potassium voltage-gated channel subfamily H member 2 | KCNH2 |
| *Hedysarum Multijugum Maxim* | Huangqi | MOL000378 | Calcium-activated potassium channel subunit alpha 1 | KCNMA1 |
| *Hedysarum Multijugum Maxim* | Huangqi | MOL000387 | Calcium-activated potassium channel subunit alpha 1 | KCNMA1 |
| *Hedysarum Multijugum Maxim* | Huangqi | MOL000387 | Vascular endothelial growth factor receptor 2 | KDR |
| *Hedysarum Multijugum Maxim* | Huangqi | MOL000392 | Beta-lactamase | LACTB |
| *Hedysarum Multijugum Maxim* | Huangqi | MOL000296 | Lysozyme | LYZ |
| *Hedysarum Multijugum Maxim* | Huangqi | MOL000354 | Amine oxidase [flavin-containing] B | MAOB |
| *Hedysarum Multijugum Maxim* | Huangqi | MOL000392 | Amine oxidase [flavin-containing] B | MAOB |
| *Hedysarum Multijugum Maxim* | Huangqi | MOL000098 | Amine oxidase [flavin-containing] B | MAOB |
| *Hedysarum Multijugum Maxim* | Huangqi | MOL000098 | Mitogen-activated protein kinase 1 | MAPK1 |
| *Hedysarum Multijugum Maxim* | Huangqi | MOL000354 | Mitogen-activated protein kinase 14 | MAPK14 |
| *Hedysarum Multijugum Maxim* | Huangqi | MOL000378 | Mitogen-activated protein kinase 14 | MAPK14 |
| *Hedysarum Multijugum Maxim* | Huangqi | MOL000392 | Mitogen-activated protein kinase 14 | MAPK14 |
| *Hedysarum Multijugum Maxim* | Huangqi | MOL000417 | Mitogen-activated protein kinase 14 | MAPK14 |
| *Hedysarum Multijugum Maxim* | Huangqi | MOL000422 | Mitogen-activated protein kinase 8 | MAPK8 |
| *Hedysarum Multijugum Maxim* | Huangqi | MOL000387 | Hepatocyte growth factor receptor | MET |
| *Hedysarum Multijugum Maxim* | Huangqi | MOL000098 | Maltase-glucoamylase, intestinal | MGAM |
| *Hedysarum Multijugum Maxim* | Huangqi | MOL000422 | Interstitial collagenase | MMP1 |
| *Hedysarum Multijugum Maxim* | Huangqi | MOL000098 | Interstitial collagenase | MMP1 |
| *Hedysarum Multijugum Maxim* | Huangqi | MOL000098 | 72 kDa type IV collagenase | MMP2 |
| *Hedysarum Multijugum Maxim* | Huangqi | MOL000098 | Stromelysin-1 | MMP3 |
| *Hedysarum Multijugum Maxim* | Huangqi | MOL000098 | Matrix metalloproteinase-9 | MMP9 |
| *Hedysarum Multijugum Maxim* | Huangqi | MOL000098 | Myeloperoxidase | MPO |
| *Hedysarum Multijugum Maxim* | Huangqi | MOL000392 | NADH-ubiquinone oxidoreductase chain 6 | MT-ND6 |
| *Hedysarum Multijugum Maxim* | Huangqi | MOL000098 | Myc proto-oncogene protein | MYC |
| *Hedysarum Multijugum Maxim* | Huangqi | MOL000354 | Neutrophil cytosol factor 1 | NCF1 |
| *Hedysarum Multijugum Maxim* | Huangqi | MOL000098 | Neutrophil cytosol factor 1 | NCF1 |
| *Hedysarum Multijugum Maxim* | Huangqi | MOL000354 | Nuclear receptor coactivator 1 | NCOA1 |
| *Hedysarum Multijugum Maxim* | Huangqi | MOL000380 | Nuclear receptor coactivator 1 | NCOA1 |
| *Hedysarum Multijugum Maxim* | Huangqi | MOL000239 | Nuclear receptor coactivator 2 | NCOA2 |
| *Hedysarum Multijugum Maxim* | Huangqi | MOL000296 | Nuclear receptor coactivator 2 | NCOA2 |
| *Hedysarum Multijugum Maxim* | Huangqi | MOL000354 | Nuclear receptor coactivator 2 | NCOA2 |
| *Hedysarum Multijugum Maxim* | Huangqi | MOL000371 | Nuclear receptor coactivator 2 | NCOA2 |
| *Hedysarum Multijugum Maxim* | Huangqi | MOL000378 | Nuclear receptor coactivator 2 | NCOA2 |
| *Hedysarum Multijugum Maxim* | Huangqi | MOL000379 | Nuclear receptor coactivator 2 | NCOA2 |
| *Hedysarum Multijugum Maxim* | Huangqi | MOL000380 | Nuclear receptor coactivator 2 | NCOA2 |
| *Hedysarum Multijugum Maxim* | Huangqi | MOL000417 | Nuclear receptor coactivator 2 | NCOA2 |
| *Hedysarum Multijugum Maxim* | Huangqi | MOL000422 | Nuclear receptor coactivator 2 | NCOA2 |
| *Hedysarum Multijugum Maxim* | Huangqi | MOL000098 | Nuclear receptor coactivator 2 | NCOA2 |
| *Hedysarum Multijugum Maxim* | Huangqi | MOL000098 | Nuclear factor erythroid 2-related factor 2 | NFE2L2 |
| *Hedysarum Multijugum Maxim* | Huangqi | MOL000098 | NF-kappa-B inhibitor alpha | NFKBIA |
| *Hedysarum Multijugum Maxim* | Huangqi | MOL000098 | Homeobox protein Nkx-3.1 | NKX3-1 |
| *Hedysarum Multijugum Maxim* | Huangqi | MOL000239 | Nitric oxide synthase, inducible | NOS2 |
| *Hedysarum Multijugum Maxim* | Huangqi | MOL000354 | Nitric oxide synthase, inducible | NOS2 |
| *Hedysarum Multijugum Maxim* | Huangqi | MOL000371 | Nitric oxide synthase, inducible | NOS2 |
| *Hedysarum Multijugum Maxim* | Huangqi | MOL000378 | Nitric oxide synthase, inducible | NOS2 |
| *Hedysarum Multijugum Maxim* | Huangqi | MOL000380 | Nitric oxide synthase, inducible | NOS2 |
| *Hedysarum Multijugum Maxim* | Huangqi | MOL000392 | Nitric oxide synthase, inducible | NOS2 |
| *Hedysarum Multijugum Maxim* | Huangqi | MOL000417 | Nitric oxide synthase, inducible | NOS2 |
| *Hedysarum Multijugum Maxim* | Huangqi | MOL000422 | Nitric oxide synthase, inducible | NOS2 |
| *Hedysarum Multijugum Maxim* | Huangqi | MOL000354 | Nitric-oxide synthase, endothelial | NOS3 |
| *Hedysarum Multijugum Maxim* | Huangqi | MOL000371 | Nitric-oxide synthase, endothelial | NOS3 |
| *Hedysarum Multijugum Maxim* | Huangqi | MOL000378 | Nitric-oxide synthase, endothelial | NOS3 |
| *Hedysarum Multijugum Maxim* | Huangqi | MOL000392 | Nitric-oxide synthase, endothelial | NOS3 |
| *Hedysarum Multijugum Maxim* | Huangqi | MOL000422 | Nitric-oxide synthase, endothelial | NOS3 |
| *Hedysarum Multijugum Maxim* | Huangqi | MOL000098 | Nitric-oxide synthase, endothelial | NOS3 |
| *Hedysarum Multijugum Maxim* | Huangqi | MOL000098 | Nitric oxide synthase, endothelial | NOS3 |
| *Hedysarum Multijugum Maxim* | Huangqi | MOL000098 | Puromycin-sensitive aminopeptidase | NPEPPS |
| *Hedysarum Multijugum Maxim* | Huangqi | MOL000098 | NAD(P)H dehydrogenase [quinone] 1 | NQO1 |
| *Hedysarum Multijugum Maxim* | Huangqi | MOL000422 | Nuclear receptor subfamily 1 group I member 2 | NR1I2 |
| *Hedysarum Multijugum Maxim* | Huangqi | MOL000098 | Nuclear receptor subfamily 1 group I member 2 | NR1I2 |
| *Hedysarum Multijugum Maxim* | Huangqi | MOL000422 | Nuclear receptor subfamily 1 group I member 3 | NR1I3 |
| *Hedysarum Multijugum Maxim* | Huangqi | MOL000098 | Nuclear receptor subfamily 1 group I member 3 | NR1I3 |
| *Hedysarum Multijugum Maxim* | Huangqi | MOL000098 | Ornithine decarboxylase | ODC1 |
| *Hedysarum Multijugum Maxim* | Huangqi | MOL000354 | Oxidized low-density lipoprotein receptor 1 | OLR1 |
| *Hedysarum Multijugum Maxim* | Huangqi | MOL000378 | Delta-type opioid receptor | OPRD1 |
| *Hedysarum Multijugum Maxim* | Huangqi | MOL000371 | Mu-type opioid receptor | OPRM1 |
| *Hedysarum Multijugum Maxim* | Huangqi | MOL000098 | Poly [ADP-ribose] polymerase 1 | PARP1 |
| *Hedysarum Multijugum Maxim* | Huangqi | MOL000098 | Procollagen C-endopeptidase enhancer 1 | PCOLCE |
| *Hedysarum Multijugum Maxim* | Huangqi | MOL000296 | CGMP-inhibited 3',5'-cyclic phosphodiesterase A | PDE3A |
| *Hedysarum Multijugum Maxim* | Huangqi | MOL000371 | CGMP-inhibited 3',5'-cyclic phosphodiesterase A | PDE3A |
| *Hedysarum Multijugum Maxim* | Huangqi | MOL000378 | CGMP-inhibited 3',5'-cyclic phosphodiesterase A | PDE3A |
| *Hedysarum Multijugum Maxim* | Huangqi | MOL000392 | CGMP-inhibited 3',5'-cyclic phosphodiesterase A | PDE3A |
| *Hedysarum Multijugum Maxim* | Huangqi | MOL000417 | CGMP-inhibited 3',5'-cyclic phosphodiesterase A | PDE3A |
| *Hedysarum Multijugum Maxim* | Huangqi | MOL000211 | Progesterone receptor | PGR |
| *Hedysarum Multijugum Maxim* | Huangqi | MOL000296 | Progesterone receptor | PGR |
| *Hedysarum Multijugum Maxim* | Huangqi | MOL000033 | Progesterone receptor | PGR |
| *Hedysarum Multijugum Maxim* | Huangqi | MOL000422 | Progesterone receptor | PGR |
| *Hedysarum Multijugum Maxim* | Huangqi | MOL000354 | Phosphatidylinositol-4,5-bisphosphate 3-kinase catalytic subunit, gamma isoform | PIK3CG |
| *Hedysarum Multijugum Maxim* | Huangqi | MOL000422 | Phosphatidylinositol-4,5-bisphosphate 3-kinase catalytic subunit, gamma isoform | PIK3CG |
| *Hedysarum Multijugum Maxim* | Huangqi | MOL000098 | Phosphatidylinositol-4,5-bisphosphate 3-kinase catalytic subunit, gamma isoform | PIK3CG |
| *Hedysarum Multijugum Maxim* | Huangqi | MOL000354 | Proto-oncogene serine/threonine-protein kinase Pim-1 | PIM1 |
| *Hedysarum Multijugum Maxim* | Huangqi | MOL000378 | Proto-oncogene serine/threonine-protein kinase Pim-1 | PIM1 |
| *Hedysarum Multijugum Maxim* | Huangqi | MOL000392 | Proto-oncogene serine/threonine-protein kinase Pim-1 | PIM1 |
| *Hedysarum Multijugum Maxim* | Huangqi | MOL000417 | Proto-oncogene serine/threonine-protein kinase Pim-1 | PIM1 |
| *Hedysarum Multijugum Maxim* | Huangqi | MOL000392 | cAMP-dependent protein kinase inhibitor alpha | PKIA |
| *Hedysarum Multijugum Maxim* | Huangqi | MOL000098 | Tissue-type plasminogen activator | PLAT |
| *Hedysarum Multijugum Maxim* | Huangqi | MOL000098 | Urokinase-type plasminogen activator | PLAU |
| *Hedysarum Multijugum Maxim* | Huangqi | MOL000098 | Serum paraoxonase/arylesterase 1 | PON1 |
| *Hedysarum Multijugum Maxim* | Huangqi | MOL000098 | NADPH--cytochrome P450 reductase | POR |
| *Hedysarum Multijugum Maxim* | Huangqi | MOL000098 | Peroxisome proliferator-activated receptor alpha | PPARA |
| *Hedysarum Multijugum Maxim* | Huangqi | MOL000354 | Peroxisome proliferator activated receptor delta | PPARD |
| *Hedysarum Multijugum Maxim* | Huangqi | MOL000098 | Peroxisome proliferator-activated receptor delta | PPARD |
| *Hedysarum Multijugum Maxim* | Huangqi | MOL000354 | Peroxisome proliferator activated receptor gamma | PPARG |
| *Hedysarum Multijugum Maxim* | Huangqi | MOL000378 | Peroxisome proliferator activated receptor gamma | PPARG |
| *Hedysarum Multijugum Maxim* | Huangqi | MOL000392 | Peroxisome proliferator activated receptor gamma | PPARG |
| *Hedysarum Multijugum Maxim* | Huangqi | MOL000392 | Peroxisome proliferator-activated receptor gamma | PPARG |
| *Hedysarum Multijugum Maxim* | Huangqi | MOL000417 | Peroxisome proliferator activated receptor gamma | PPARG |
| *Hedysarum Multijugum Maxim* | Huangqi | MOL000422 | Peroxisome proliferator activated receptor gamma | PPARG |
| *Hedysarum Multijugum Maxim* | Huangqi | MOL000422 | Peroxisome proliferator-activated receptor gamma | PPARG |
| *Hedysarum Multijugum Maxim* | Huangqi | MOL000098 | Peroxisome proliferator activated receptor gamma | PPARG |
| *Hedysarum Multijugum Maxim* | Huangqi | MOL000098 | Peroxisome proliferator-activated receptor gamma | PPARG |
| *Hedysarum Multijugum Maxim* | Huangqi | MOL000422 | Serine/threonine-protein phosphatase 2B catalytic subunit alpha isoform | PPP3CA |
| *Hedysarum Multijugum Maxim* | Huangqi | MOL000098 | Protein kinase C alpha type | PRKCA |
| *Hedysarum Multijugum Maxim* | Huangqi | MOL000098 | Protein kinase C beta type | PRKCB |
| *Hedysarum Multijugum Maxim* | Huangqi | MOL000239 | Trypsin-1 | PRSS1 |
| *Hedysarum Multijugum Maxim* | Huangqi | MOL000354 | Trypsin-1 | PRSS1 |
| *Hedysarum Multijugum Maxim* | Huangqi | MOL000371 | Trypsin-1 | PRSS1 |
| *Hedysarum Multijugum Maxim* | Huangqi | MOL000378 | Trypsin-1 | PRSS1 |
| *Hedysarum Multijugum Maxim* | Huangqi | MOL000380 | Trypsin-1 | PRSS1 |
| *Hedysarum Multijugum Maxim* | Huangqi | MOL000392 | Trypsin-1 | PRSS1 |
| *Hedysarum Multijugum Maxim* | Huangqi | MOL000417 | Trypsin-1 | PRSS1 |
| *Hedysarum Multijugum Maxim* | Huangqi | MOL000422 | Trypsin-1 | PRSS1 |
| *Hedysarum Multijugum Maxim* | Huangqi | MOL000442 | Trypsin-1 | PRSS1 |
| *Hedysarum Multijugum Maxim* | Huangqi | MOL000098 | Trypsin-1 | PRSS1 |
| *Hedysarum Multijugum Maxim* | Huangqi | MOL000422 | 26S proteasome non-ATPase regulatory subunit 3 | PSMD3 |
| *Hedysarum Multijugum Maxim* | Huangqi | MOL000098 | 26S proteasome non-ATPase regulatory subunit 3 | PSMD3 |
| *Hedysarum Multijugum Maxim* | Huangqi | MOL000098 | Phosphatidylinositol-3,4,5-trisphosphate 3-phosphatase and dual-specificity protein phosphatase PTEN | PTEN |
| *Hedysarum Multijugum Maxim* | Huangqi | MOL000098 | Prostaglandin E2 receptor EP3 subtype | PTGER3 |
| *Hedysarum Multijugum Maxim* | Huangqi | MOL000239 | Prostaglandin G/H synthase 1 | PTGS1 |
| *Hedysarum Multijugum Maxim* | Huangqi | MOL000296 | Prostaglandin G/H synthase 1 | PTGS1 |
| *Hedysarum Multijugum Maxim* | Huangqi | MOL000354 | Prostaglandin G/H synthase 1 | PTGS1 |
| *Hedysarum Multijugum Maxim* | Huangqi | MOL000371 | Prostaglandin G/H synthase 1 | PTGS1 |
| *Hedysarum Multijugum Maxim* | Huangqi | MOL000378 | Prostaglandin G/H synthase 1 | PTGS1 |
| *Hedysarum Multijugum Maxim* | Huangqi | MOL000380 | Prostaglandin G/H synthase 1 | PTGS1 |
| *Hedysarum Multijugum Maxim* | Huangqi | MOL000387 | Prostaglandin G/H synthase 1 | PTGS1 |
| *Hedysarum Multijugum Maxim* | Huangqi | MOL000392 | Prostaglandin G/H synthase 1 | PTGS1 |
| *Hedysarum Multijugum Maxim* | Huangqi | MOL000417 | Prostaglandin G/H synthase 1 | PTGS1 |
| *Hedysarum Multijugum Maxim* | Huangqi | MOL000422 | Prostaglandin G/H synthase | PTGS1 |
| *Hedysarum Multijugum Maxim* | Huangqi | MOL000098 | Prostaglandin G/H synthase 1 | PTGS1 |
| *Hedysarum Multijugum Maxim* | Huangqi | MOL000239 | Prostaglandin G/H synthase 2 | PTGS2 |
| *Hedysarum Multijugum Maxim* | Huangqi | MOL000296 | Prostaglandin G/H synthase 2 | PTGS2 |
| *Hedysarum Multijugum Maxim* | Huangqi | MOL000354 | Prostaglandin G/H synthase 2 | PTGS2 |
| *Hedysarum Multijugum Maxim* | Huangqi | MOL000371 | Prostaglandin G/H synthase 2 | PTGS2 |
| *Hedysarum Multijugum Maxim* | Huangqi | MOL000378 | Prostaglandin G/H synthase 2 | PTGS2 |
| *Hedysarum Multijugum Maxim* | Huangqi | MOL000379 | Prostaglandin G/H synthase 2 | PTGS2 |
| *Hedysarum Multijugum Maxim* | Huangqi | MOL000380 | Prostaglandin G/H synthase 2 | PTGS2 |
| *Hedysarum Multijugum Maxim* | Huangqi | MOL000387 | Prostaglandin G/H synthase 2 | PTGS2 |
| *Hedysarum Multijugum Maxim* | Huangqi | MOL000392 | Prostaglandin G/H synthase 2 | PTGS2 |
| *Hedysarum Multijugum Maxim* | Huangqi | MOL000417 | Prostaglandin G/H synthase 2 | PTGS2 |
| *Hedysarum Multijugum Maxim* | Huangqi | MOL000422 | Prostaglandin G/H synthase 2 | PTGS2 |
| *Hedysarum Multijugum Maxim* | Huangqi | MOL000442 | Prostaglandin G/H synthase 2 | PTGS2 |
| *Hedysarum Multijugum Maxim* | Huangqi | MOL000098 | Prostaglandin G/H synthase 2 | PTGS2 |
| *Hedysarum Multijugum Maxim* | Huangqi | MOL000354 | mRNA of Protein-tyrosine phosphatase, non-receptor type 1 | PTPN1 |
| *Hedysarum Multijugum Maxim* | Huangqi | MOL000354 | Glycogen phosphorylase, muscle form | PYGM |
| *Hedysarum Multijugum Maxim* | Huangqi | MOL000098 | RAF proto-oncogene serine/threonine-protein kinase | RAF1 |
| *Hedysarum Multijugum Maxim* | Huangqi | MOL000098 | Ras GTPase-activating protein 1 | RASA1 |
| *Hedysarum Multijugum Maxim* | Huangqi | MOL000098 | Ras association domain-containing protein 1 | RASSF1 |
| *Hedysarum Multijugum Maxim* | Huangqi | MOL000098 | Retinoblastoma-associated protein | RB1 |
| *Hedysarum Multijugum Maxim* | Huangqi | MOL000354 | Transcription factor p65 | RELA |
| *Hedysarum Multijugum Maxim* | Huangqi | MOL000422 | Transcription factor p65 | RELA |
| *Hedysarum Multijugum Maxim* | Huangqi | MOL000098 | Transcription factor p65 | RELA |
| *Hedysarum Multijugum Maxim* | Huangqi | MOL000098 | Protein CBFA2T1 | RUNX1T1 |
| *Hedysarum Multijugum Maxim* | Huangqi | MOL000098 | Runt-related transcription factor 2 | RUNX2 |
| *Hedysarum Multijugum Maxim* | Huangqi | MOL000296 | Retinoic acid receptor RXR-alpha | RXRA |
| *Hedysarum Multijugum Maxim* | Huangqi | MOL000371 | Retinoic acid receptor RXR-alpha | RXRA |
| *Hedysarum Multijugum Maxim* | Huangqi | MOL000378 | Retinoic acid receptor RXR-alpha | RXRA |
| *Hedysarum Multijugum Maxim* | Huangqi | MOL000378 | Retinoic acid receptor RXR-beta | RXRA |
| *Hedysarum Multijugum Maxim* | Huangqi | MOL000380 | Retinoic acid receptor RXR-alpha | RXRA |
| *Hedysarum Multijugum Maxim* | Huangqi | MOL000392 | Retinoic acid receptor RXR-alpha | RXRA |
| *Hedysarum Multijugum Maxim* | Huangqi | MOL000417 | Retinoic acid receptor RXR-alpha | RXRA |
| *Hedysarum Multijugum Maxim* | Huangqi | MOL000442 | Retinoic acid receptor RXR-alpha | RXRA |
| *Hedysarum Multijugum Maxim* | Huangqi | MOL000098 | Retinoic acid receptor RXR-alpha | RXRA |
| *Hedysarum Multijugum Maxim* | Huangqi | MOL000239 | Sodium channel protein type 5 subunit alpha | SCN5A |
| *Hedysarum Multijugum Maxim* | Huangqi | MOL000296 | Sodium channel protein type 5 subunit alpha | SCN5A |
| *Hedysarum Multijugum Maxim* | Huangqi | MOL000371 | Sodium channel protein type 5 subunit alpha | SCN5A |
| *Hedysarum Multijugum Maxim* | Huangqi | MOL000378 | Sodium channel protein type 5 subunit alpha | SCN5A |
| *Hedysarum Multijugum Maxim* | Huangqi | MOL000380 | Sodium channel protein type 5 subunit alpha | SCN5A |
| *Hedysarum Multijugum Maxim* | Huangqi | MOL000098 | Sodium channel protein type 5 subunit alpha | SCN5A |
| *Hedysarum Multijugum Maxim* | Huangqi | MOL000422 | E-selectin | SELE |
| *Hedysarum Multijugum Maxim* | Huangqi | MOL000098 | E-selectin | SELE |
| *Hedysarum Multijugum Maxim* | Huangqi | MOL000098 | Plasminogen activator inhibitor 1 | SERPINE1 |
| *Hedysarum Multijugum Maxim* | Huangqi | MOL000392 | NAD-dependent deacetylase sirtuin-1 | SIRT1 |
| *Hedysarum Multijugum Maxim* | Huangqi | MOL000422 | Solute carrier family 2, facilitated glucose transporter member 4 | SLC2A4 |
| *Hedysarum Multijugum Maxim* | Huangqi | MOL000098 | Solute carrier family 2, facilitated glucose transporter member 4 | SLC2A4 |
| *Hedysarum Multijugum Maxim* | Huangqi | MOL000296 | Sodium-dependent noradrenaline transporter | SLC6A2 |
| *Hedysarum Multijugum Maxim* | Huangqi | MOL000422 | Sodium-dependent noradrenaline transporter | SLC6A2 |
| *Hedysarum Multijugum Maxim* | Huangqi | MOL000378 | Sodium-dependent dopamine transporter | SLC6A3 |
| *Hedysarum Multijugum Maxim* | Huangqi | MOL000392 | Sodium-dependent dopamine transporter | SLC6A3 |
| *Hedysarum Multijugum Maxim* | Huangqi | MOL000378 | Sodium-dependent serotonin transporter | SLC6A4 |
| *Hedysarum Multijugum Maxim* | Huangqi | MOL000392 | Sodium-dependent serotonin transporter | SLC6A4 |
| *Hedysarum Multijugum Maxim* | Huangqi | MOL000422 | Antileukoproteinase | SLPI |
| *Hedysarum Multijugum Maxim* | Huangqi | MOL000098 | Superoxide dismutase [Cu-Zn] | SOD1 |
| *Hedysarum Multijugum Maxim* | Huangqi | MOL000098 | Osteopontin | SPP1 |
| *Hedysarum Multijugum Maxim* | Huangqi | MOL000422 | Signal transducer and activator of transcription 1-alpha/beta | STAT1 |
| *Hedysarum Multijugum Maxim* | Huangqi | MOL000098 | Signal transducer and activator of transcription 1-alpha/beta | STAT1 |
| *Hedysarum Multijugum Maxim* | Huangqi | MOL000098 | Estrogen sulfotransferase | SULT1E1 |
| *Hedysarum Multijugum Maxim* | Huangqi | MOL000098 | Transforming growth factor beta-1 | TGFB1 |
| *Hedysarum Multijugum Maxim* | Huangqi | MOL000098 | Thrombomodulin | THBD |
| *Hedysarum Multijugum Maxim* | Huangqi | MOL000422 | Tumor necrosis factor | TNF |
| *Hedysarum Multijugum Maxim* | Huangqi | MOL000098 | Tumor necrosis factor | TNF |
| *Hedysarum Multijugum Maxim* | Huangqi | MOL000098 | DNA topoisomerase 1 | TOP1 |
| *Hedysarum Multijugum Maxim* | Huangqi | MOL000098 | DNA topoisomerase 2-alpha | TOP2A |
| *Hedysarum Multijugum Maxim* | Huangqi | MOL000379 | DNA topoisomerase II | TOP2B |
| *Hedysarum Multijugum Maxim* | Huangqi | MOL000387 | DNA topoisomerase II | TOP2B |
| *Hedysarum Multijugum Maxim* | Huangqi | MOL000422 | DNA topoisomerase II | TOP2B |
| *Hedysarum Multijugum Maxim* | Huangqi | MOL000439 | DNA topoisomerase II | TOP2B |
| *Hedysarum Multijugum Maxim* | Huangqi | MOL000098 | DNA topoisomerase II | TOP2B |
| *Hedysarum Multijugum Maxim* | Huangqi | MOL000098 | Cellular tumor antigen p53 | TP53 |
| *Hedysarum Multijugum Maxim* | Huangqi | MOL000422 | Vascular cell adhesion protein 1 | VCAM1 |
| *Hedysarum Multijugum Maxim* | Huangqi | MOL000098 | Vascular cell adhesion protein 1 | VCAM1 |
| *Hedysarum Multijugum Maxim* | Huangqi | MOL000098 | Vascular endothelial growth factor A | VEGFA |
| *Hedysarum Multijugum Maxim* | Huangqi | MOL000354 | Xanthine dehydrogenase/oxidase | XDH |
| *Hedysarum Multijugum Maxim* | Huangqi | MOL000422 | Xanthine dehydrogenase/oxidase | XDH |
| *Hedysarum Multijugum Maxim* | Huangqi | MOL000098 | Xanthine dehydrogenase/oxidase | XDH |

| Herbal Name | Compounds Number | Targets Number |
| --- | --- | --- |
| Jinyinhua (JYH) | 17 | 437 |
| Gancao (GC) | 88 | 1738 |
| Banlangen (BLG) | 35 | 322 |
| Guanzhong (GZ) | 5 | 101 |
| Huangqi (HQ) | 17 | 450 |


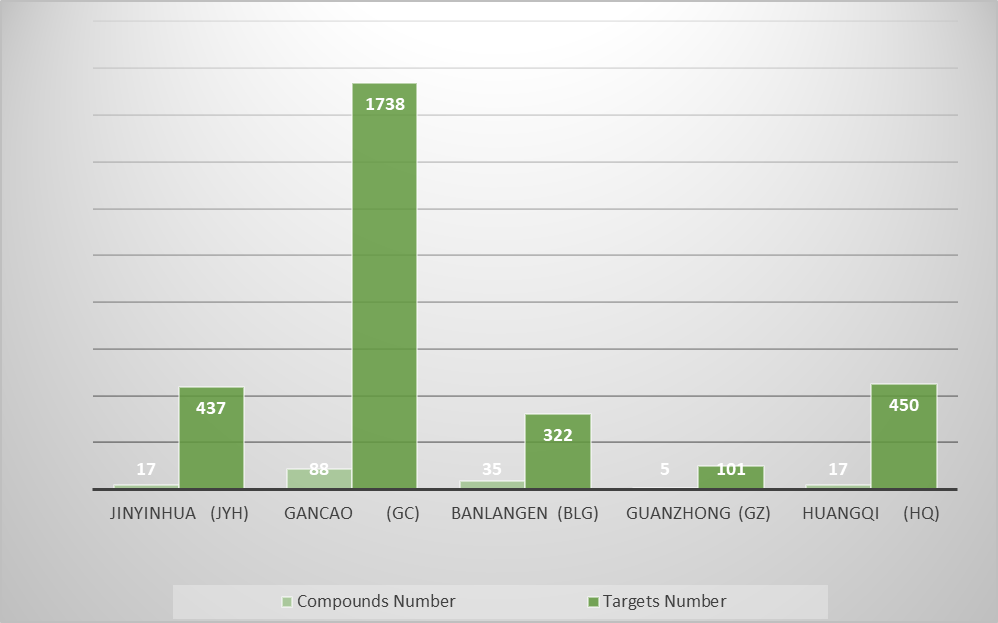


| Herbal Name | Targets Number |
| --- | --- |
| Jinyinhua (JYH) | 437 |
| Gancao (GC) | 1738 |
| Banlangen (BLG) | 322 |
| Guanzhong (GZ) | 101 |
| Huangqi (HQ) | 450 |


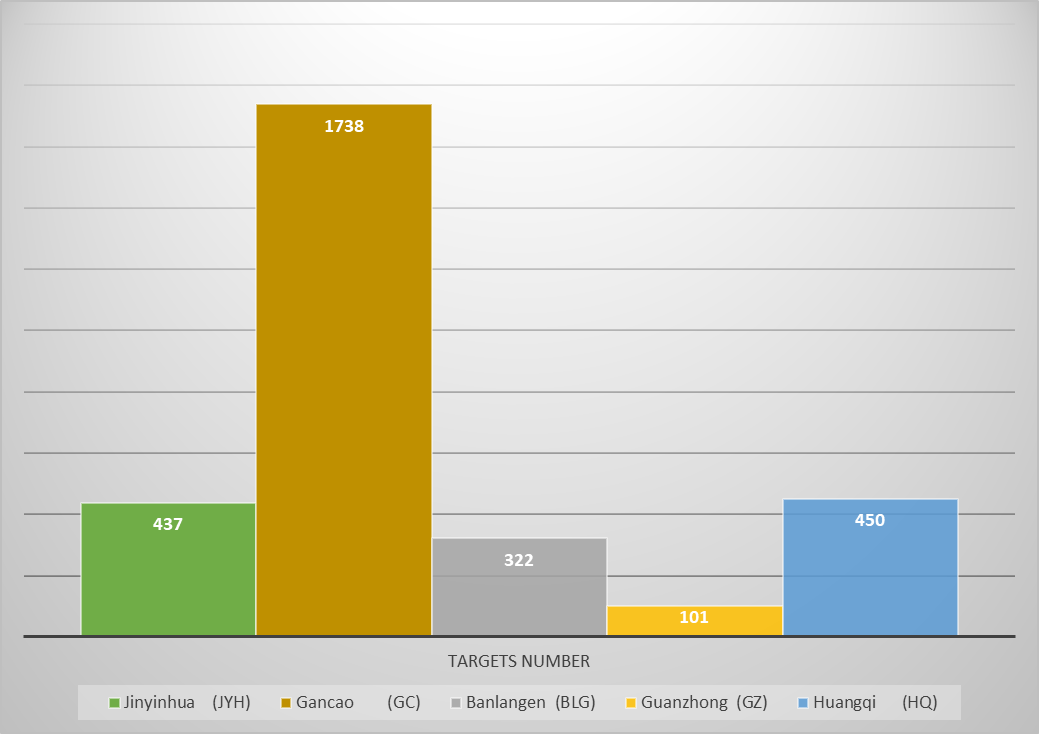

Supplement: Supplementary Materials — Supplementary file 1, Tables S1 and S2: the basic information of all active compounds and related targets. Supplementary file 2, Table S3; and Supplementary file 3, Figure S1: the detailed information of the potential target genes of acne vulgaris. Supplementary file 4, Tables 4, S5, and S6: the detailed information of GO enrichment analysis for BP, CC, and MF. Supplementary file 5, Table S7: the detailed information of screened KEGG pathways. [file 6944792.f1.zip › 6944792.f1/Supplementary file1, Tables S1, S2 The basic information of all active compounds and related targets. (1).docx]
