# Supplementary material for: Network Pharmacology-Based Investigation on the Mechanism of the JinGuanLan Formula in Treating Acne Vulgaris
Source: Evid Based Complement Alternat Med. 2022 Jul 13;2022:6944792. doi: 10.1155/2022/6944792 (PMC9300327; doi:10.1155/2022/6944792)

| **Supplementary file2: Table S3 : The detailed information of the potential target genes of acne vulgaris** | | | | | | | | | | | | | | | | | | |
| --- | --- | --- | --- | --- | --- | --- | --- | --- | --- | --- | --- | --- | --- | --- | --- | --- | --- | --- |
| **Target** | **Source** |  | **Target** | **Source** |  | **Target** | **Source** |  | **Target** | **Source** |  | **Target** | **Source** |  | **Database** | **Number** |  | **Final Target** |
| ABCA12 | GeneCards |  | FLG | OMIM |  | ALAD | TTD |  | ABCD2 | DisGeNET |  | ABCB1 | CTD |  | **GeneCards** | 524 |  | ABCA12 |
| ABCD2 | GeneCards |  | NCSTN | OMIM |  | AR | TTD |  | ACE | DisGeNET |  | ABCB11 | CTD |  | **OMIM** | 7 |  | ABCB1 |
| ACE | GeneCards |  | RBP4 | OMIM |  | BPI | TTD |  | ADIPOQ | DisGeNET |  | ACTA2 | CTD |  | **TTD** | 19 |  | ABCB11 |
| ACHE | GeneCards |  | PSEN1 | OMIM |  | ESR1 | TTD |  | ADORA2A-AS1 | DisGeNET |  | AGT | CTD |  | **DisGeNET** | 87 |  | ABCD2 |
| ACP5 | GeneCards |  | PSTPIP1 | OMIM |  | ESRRA | TTD |  | ANGPTL3 | DisGeNET |  | ALB | CTD |  | **CTD** | 95 |  | ACE |
| ADD2 | GeneCards |  | DSG3 | OMIM |  | FECH | TTD |  | ANGPTL4 | DisGeNET |  | AR | CTD |  |  |  |  | ACHE |
| ADIPOQ | GeneCards |  | PSENEN | OMIM |  | FGF2 | TTD |  | APH1A | DisGeNET |  | BCL2 | CTD |  |  |  |  | ACP5 |
| ADM | GeneCards |  |  |  |  | IKBKB | TTD |  | APOC3 | DisGeNET |  | CASP3 | CTD |  |  |  |  | ACTA2 |
| ADORA2A-AS1 | GeneCards |  |  |  |  | IL17A | TTD |  | AR | DisGeNET |  | CAT | CTD |  |  |  |  | ADD2 |
| AHR | GeneCards |  |  |  |  | IL1A | TTD |  | BDNF | DisGeNET |  | CAV1 | CTD |  |  |  |  | ADIPOQ |
| AKT1 | GeneCards |  |  |  |  | MC5R | TTD |  | C11orf49 | DisGeNET |  | CCL2 | CTD |  |  |  |  | ADM |
| ALB | GeneCards |  |  |  |  | MMP2 | TTD |  | C1orf112 | DisGeNET |  | CCL3 | CTD |  |  |  |  | ADORA2A-AS1 |
| ALG12 | GeneCards |  |  |  |  | RARA | TTD |  | CALCA | DisGeNET |  | CCL5 | CTD |  |  |  |  | AGT |
| ALOX5 | GeneCards |  |  |  |  | RARG | TTD |  | CAMP | DisGeNET |  | CRABP2 | CTD |  |  |  |  | AHR |
| ALPP | GeneCards |  |  |  |  | S1PR1 | TTD |  | CCL5 | DisGeNET |  | CRP | CTD |  |  |  |  | AKT1 |
| ANPEP | GeneCards |  |  |  |  | SRD5A1 | TTD |  | CD79A | DisGeNET |  | CXCL8 | CTD |  |  |  |  | ALAD |
| APOA1 | GeneCards |  |  |  |  | SRD5A2 | TTD |  | CD83 | DisGeNET |  | CYCS | CTD |  |  |  |  | ALB |
| APOB | GeneCards |  |  |  |  | VDR | TTD |  | CHI3L1 | DisGeNET |  | CYP26B1 | CTD |  |  |  |  | ALG12 |
| APOH | GeneCards |  |  |  |  | ZNF224 | TTD |  | CRH | DisGeNET |  | CYP26C1 | CTD |  |  |  |  | ALOX5 |
| AR | GeneCards |  |  |  |  |  |  |  | CXCL8 | DisGeNET |  | CYP3A4 | CTD |  |  |  |  | ALPP |
| ARSA | GeneCards |  |  |  |  |  |  |  | CYP17A1 | DisGeNET |  | DCN | CTD |  |  |  |  | ANGPTL3 |
| ASCL4 | GeneCards |  |  |  |  |  |  |  | CYP19A1 | DisGeNET |  | EGFR | CTD |  |  |  |  | ANGPTL4 |
| ASPRV1 | GeneCards |  |  |  |  |  |  |  | DCD | DisGeNET |  | ETFDH | CTD |  |  |  |  | ANPEP |
| ATP2C1 | GeneCards |  |  |  |  |  |  |  | DEFB1 | DisGeNET |  | F10 | CTD |  |  |  |  | APH1A |
| ATP6V1B1 | GeneCards |  |  |  |  |  |  |  | DEFB4A | DisGeNET |  | F2 | CTD |  |  |  |  | APOA1 |
| B3GNT2 | GeneCards |  |  |  |  |  |  |  | DEFB4B | DisGeNET |  | F7 | CTD |  |  |  |  | APOB |
| BANF1 | GeneCards |  |  |  |  |  |  |  | DNMT1 | DisGeNET |  | FGA | CTD |  |  |  |  | APOC3 |
| BCL11A | GeneCards |  |  |  |  |  |  |  | EEF1D | DisGeNET |  | FGB | CTD |  |  |  |  | APOH |
| BCL2 | GeneCards |  |  |  |  |  |  |  | FOXO1 | DisGeNET |  | FGG | CTD |  |  |  |  | AR |
| BCL2L1 | GeneCards |  |  |  |  |  |  |  | FOXO3 | DisGeNET |  | FOS | CTD |  |  |  |  | ARSA |
| BDNF | GeneCards |  |  |  |  |  |  |  | GALNS | DisGeNET |  | FSHB | CTD |  |  |  |  | ASCL4 |
| BGLAP | GeneCards |  |  |  |  |  |  |  | GAST | DisGeNET |  | G6PD | CTD |  |  |  |  | ASPRV1 |
| BIRC5 | GeneCards |  |  |  |  |  |  |  | GPT | DisGeNET |  | GCLC | CTD |  |  |  |  | ATP2C1 |
| BRD1 | GeneCards |  |  |  |  |  |  |  | HDAC8 | DisGeNET |  | GREB1 | CTD |  |  |  |  | ATP6V1B1 |
| BTBD11 | GeneCards |  |  |  |  |  |  |  | HDAC9 | DisGeNET |  | GSR | CTD |  |  |  |  | B3GNT2 |
| BTD | GeneCards |  |  |  |  |  |  |  | HSD17B3 | DisGeNET |  | GSTP1 | CTD |  |  |  |  | BANF1 |
| C11orf49 | GeneCards |  |  |  |  |  |  |  | HSD3B1 | DisGeNET |  | HMGCS1 | CTD |  |  |  |  | BCL11A |
| C11orf68 | GeneCards |  |  |  |  |  |  |  | IGF1 | DisGeNET |  | HPGD | CTD |  |  |  |  | BCL2 |
| C1orf141 | GeneCards |  |  |  |  |  |  |  | IGFBP7 | DisGeNET |  | HSD11B2 | CTD |  |  |  |  | BCL2L1 |
| C22orf34 | GeneCards |  |  |  |  |  |  |  | IGHA1 | DisGeNET |  | IFITM1 | CTD |  |  |  |  | BDNF |
| C2orf42 | GeneCards |  |  |  |  |  |  |  | IL10 | DisGeNET |  | IGF1 | CTD |  |  |  |  | BGLAP |
| C4A | GeneCards |  |  |  |  |  |  |  | IL17A | DisGeNET |  | IGFBP3 | CTD |  |  |  |  | BIRC5 |
| C5AR1 | GeneCards |  |  |  |  |  |  |  | IL1A | DisGeNET |  | IL10 | CTD |  |  |  |  | BPI |
| C5orf58 | GeneCards |  |  |  |  |  |  |  | IL1B | DisGeNET |  | IL1B | CTD |  |  |  |  | BRD1 |
| C6orf89 | GeneCards |  |  |  |  |  |  |  | IL1RN | DisGeNET |  | IL2 | CTD |  |  |  |  | BTBD11 |
| CA3 | GeneCards |  |  |  |  |  |  |  | IL6 | DisGeNET |  | IL4 | CTD |  |  |  |  | BTD |
| CABIN1 | GeneCards |  |  |  |  |  |  |  | LCN2 | DisGeNET |  | IL6 | CTD |  |  |  |  | C11orf49 |
| CADM1 | GeneCards |  |  |  |  |  |  |  | LEP | DisGeNET |  | KPNA1 | CTD |  |  |  |  | C11orf68 |
| CALCA | GeneCards |  |  |  |  |  |  |  | LGALS7 | DisGeNET |  | LCN2 | CTD |  |  |  |  | C1orf112 |
| CALCRL | GeneCards |  |  |  |  |  |  |  | LPL | DisGeNET |  | LHB | CTD |  |  |  |  | C1orf141 |
| CAMP | GeneCards |  |  |  |  |  |  |  | LYZ | DisGeNET |  | MAPK1 | CTD |  |  |  |  | C22orf34 |
| CARD14 | GeneCards |  |  |  |  |  |  |  | MEFV | DisGeNET |  | MAPK3 | CTD |  |  |  |  | C2orf42 |
| CASP1 | GeneCards |  |  |  |  |  |  |  | MMP2 | DisGeNET |  | MBNL1 | CTD |  |  |  |  | C4A |
| CAT | GeneCards |  |  |  |  |  |  |  | NAP1L2 | DisGeNET |  | MDK | CTD |  |  |  |  | C5AR1 |
| CATSPER1 | GeneCards |  |  |  |  |  |  |  | NLRP3 | DisGeNET |  | MKI67 | CTD |  |  |  |  | C5orf58 |
| CBS | GeneCards |  |  |  |  |  |  |  | NOD2 | DisGeNET |  | MMP13 | CTD |  |  |  |  | C6orf89 |
| CCDC167 | GeneCards |  |  |  |  |  |  |  | PAGR1 | DisGeNET |  | MMP2 | CTD |  |  |  |  | CA3 |
| CCDC85B | GeneCards |  |  |  |  |  |  |  | PCNX3 | DisGeNET |  | MMP3 | CTD |  |  |  |  | CABIN1 |
| CCL17 | GeneCards |  |  |  |  |  |  |  | PINX1 | DisGeNET |  | MMP9 | CTD |  |  |  |  | CADM1 |
| CCL2 | GeneCards |  |  |  |  |  |  |  | PLXNA2 | DisGeNET |  | MUC1 | CTD |  |  |  |  | CALCA |
| CCL20 | GeneCards |  |  |  |  |  |  |  | PPARD | DisGeNET |  | MYC | CTD |  |  |  |  | CALCRL |
| CCL27 | GeneCards |  |  |  |  |  |  |  | PPARG | DisGeNET |  | NKX2-1 | CTD |  |  |  |  | CAMP |
| CCL3 | GeneCards |  |  |  |  |  |  |  | PSTPIP1 | DisGeNET |  | NOS3 | CTD |  |  |  |  | CARD14 |
| CCR1 | GeneCards |  |  |  |  |  |  |  | PTGS2 | DisGeNET |  | NR3C1 | CTD |  |  |  |  | CASP1 |
| CCR3 | GeneCards |  |  |  |  |  |  |  | RETN | DisGeNET |  | ODC1 | CTD |  |  |  |  | CASP3 |
| CCR4 | GeneCards |  |  |  |  |  |  |  | RNASE7 | DisGeNET |  | PCK1 | CTD |  |  |  |  | CAT |
| CCR5 | GeneCards |  |  |  |  |  |  |  | S100A7 | DisGeNET |  | PER1 | CTD |  |  |  |  | CATSPER1 |
| CCR6 | GeneCards |  |  |  |  |  |  |  | S100A7A | DisGeNET |  | PGR | CTD |  |  |  |  | CAV1 |
| CD14 | GeneCards |  |  |  |  |  |  |  | SEMA4B | DisGeNET |  | PLAT | CTD |  |  |  |  | CBS |
| CD2 | GeneCards |  |  |  |  |  |  |  | SERPINA1 | DisGeNET |  | PLG | CTD |  |  |  |  | CCDC167 |
| CD207 | GeneCards |  |  |  |  |  |  |  | SETBP1 | DisGeNET |  | PRL | CTD |  |  |  |  | CCDC85B |
| CD209 | GeneCards |  |  |  |  |  |  |  | SFPQ | DisGeNET |  | PROC | CTD |  |  |  |  | CCL17 |
| CD27 | GeneCards |  |  |  |  |  |  |  | SMIM10L2A | DisGeNET |  | PROS1 | CTD |  |  |  |  | CCL2 |
| CD276 | GeneCards |  |  |  |  |  |  |  | SMIM10L2B | DisGeNET |  | PTGDS | CTD |  |  |  |  | CCL20 |
| CD28 | GeneCards |  |  |  |  |  |  |  | SOX9 | DisGeNET |  | PTGS2 | CTD |  |  |  |  | CCL27 |
| CD36 | GeneCards |  |  |  |  |  |  |  | SREBF1 | DisGeNET |  | RARA | CTD |  |  |  |  | CCL3 |
| CD4 | GeneCards |  |  |  |  |  |  |  | SUGCT | DisGeNET |  | RARG | CTD |  |  |  |  | CCL5 |
| CD40 | GeneCards |  |  |  |  |  |  |  | TIMP2 | DisGeNET |  | RBP1 | CTD |  |  |  |  | CCR1 |
| CD40LG | GeneCards |  |  |  |  |  |  |  | TLR2 | DisGeNET |  | RXRG | CTD |  |  |  |  | CCR3 |
| CD44 | GeneCards |  |  |  |  |  |  |  | TLR4 | DisGeNET |  | S100A8 | CTD |  |  |  |  | CCR4 |
| CD69 | GeneCards |  |  |  |  |  |  |  | TNF | DisGeNET |  | S100A9 | CTD |  |  |  |  | CCR5 |
| CD80 | GeneCards |  |  |  |  |  |  |  | TNFRSF1B | DisGeNET |  | SERPINC1 | CTD |  |  |  |  | CCR6 |
| CD8A | GeneCards |  |  |  |  |  |  |  | TNFSF12 | DisGeNET |  | SERPINE1 | CTD |  |  |  |  | CD14 |
| CDC42EP2 | GeneCards |  |  |  |  |  |  |  | UBE2E1 | DisGeNET |  | SERPING1 | CTD |  |  |  |  | CD2 |
| CDCP1 | GeneCards |  |  |  |  |  |  |  | UROD | DisGeNET |  | SOX9 | CTD |  |  |  |  | CD207 |
| CDH1 | GeneCards |  |  |  |  |  |  |  | VDR | DisGeNET |  | SRGN | CTD |  |  |  |  | CD209 |
| CDH3 | GeneCards |  |  |  |  |  |  |  | WNT10A | DisGeNET |  | TAGLN | CTD |  |  |  |  | CD27 |
| CDKAL1 | GeneCards |  |  |  |  |  |  |  |  |  |  | TF | CTD |  |  |  |  | CD276 |
| CDKN1A | GeneCards |  |  |  |  |  |  |  |  |  |  | TFRC | CTD |  |  |  |  | CD28 |
| CETP | GeneCards |  |  |  |  |  |  |  |  |  |  | TGFB1 | CTD |  |  |  |  | CD36 |
| CFH | GeneCards |  |  |  |  |  |  |  |  |  |  | TGFBR2 | CTD |  |  |  |  | CD4 |
| CFL1 | GeneCards |  |  |  |  |  |  |  |  |  |  | TNF | CTD |  |  |  |  | CD40 |
| CIITA | GeneCards |  |  |  |  |  |  |  |  |  |  | TP53 | CTD |  |  |  |  | CD40LG |
| CLEC3B | GeneCards |  |  |  |  |  |  |  |  |  |  | VCAM1 | CTD |  |  |  |  | CD44 |
| CLEC4F | GeneCards |  |  |  |  |  |  |  |  |  |  | VEGFA | CTD |  |  |  |  | CD69 |
| CMKLR1 | GeneCards |  |  |  |  |  |  |  |  |  |  |  |  |  |  |  |  | CD79A |
| CNR2 | GeneCards |  |  |  |  |  |  |  |  |  |  |  |  |  |  |  |  | CD80 |
| COMT | GeneCards |  |  |  |  |  |  |  |  |  |  |  |  |  |  |  |  | CD83 |
| CORO1C | GeneCards |  |  |  |  |  |  |  |  |  |  |  |  |  |  |  |  | CD8A |
| CP | GeneCards |  |  |  |  |  |  |  |  |  |  |  |  |  |  |  |  | CDC42EP2 |
| CPNE5 | GeneCards |  |  |  |  |  |  |  |  |  |  |  |  |  |  |  |  | CDCP1 |
| CRABP2 | GeneCards |  |  |  |  |  |  |  |  |  |  |  |  |  |  |  |  | CDH1 |
| CRELD2 | GeneCards |  |  |  |  |  |  |  |  |  |  |  |  |  |  |  |  | CDH3 |
| CRH | GeneCards |  |  |  |  |  |  |  |  |  |  |  |  |  |  |  |  | CDKAL1 |
| CRHR1 | GeneCards |  |  |  |  |  |  |  |  |  |  |  |  |  |  |  |  | CDKN1A |
| CRP | GeneCards |  |  |  |  |  |  |  |  |  |  |  |  |  |  |  |  | CETP |
| CSF1 | GeneCards |  |  |  |  |  |  |  |  |  |  |  |  |  |  |  |  | CFH |
| CSF2 | GeneCards |  |  |  |  |  |  |  |  |  |  |  |  |  |  |  |  | CFL1 |
| CSF3 | GeneCards |  |  |  |  |  |  |  |  |  |  |  |  |  |  |  |  | CHI3L1 |
| CSK | GeneCards |  |  |  |  |  |  |  |  |  |  |  |  |  |  |  |  | CIITA |
| CST6 | GeneCards |  |  |  |  |  |  |  |  |  |  |  |  |  |  |  |  | CLEC3B |
| CTLA4 | GeneCards |  |  |  |  |  |  |  |  |  |  |  |  |  |  |  |  | CLEC4F |
| CTNNB1 | GeneCards |  |  |  |  |  |  |  |  |  |  |  |  |  |  |  |  | CMKLR1 |
| CTSG | GeneCards |  |  |  |  |  |  |  |  |  |  |  |  |  |  |  |  | CNR2 |
| CTSW | GeneCards |  |  |  |  |  |  |  |  |  |  |  |  |  |  |  |  | COMT |
| CXCL8 | GeneCards |  |  |  |  |  |  |  |  |  |  |  |  |  |  |  |  | CORO1C |
| CXCR3 | GeneCards |  |  |  |  |  |  |  |  |  |  |  |  |  |  |  |  | CP |
| CXCR5 | GeneCards |  |  |  |  |  |  |  |  |  |  |  |  |  |  |  |  | CPNE5 |
| CYB5A | GeneCards |  |  |  |  |  |  |  |  |  |  |  |  |  |  |  |  | CRABP2 |
| CYP11B1 | GeneCards |  |  |  |  |  |  |  |  |  |  |  |  |  |  |  |  | CRELD2 |
| CYP17A1 | GeneCards |  |  |  |  |  |  |  |  |  |  |  |  |  |  |  |  | CRH |
| CYP19A1 | GeneCards |  |  |  |  |  |  |  |  |  |  |  |  |  |  |  |  | CRHR1 |
| CYP1A1 | GeneCards |  |  |  |  |  |  |  |  |  |  |  |  |  |  |  |  | CRP |
| CYP1A2 | GeneCards |  |  |  |  |  |  |  |  |  |  |  |  |  |  |  |  | CSF1 |
| CYP21A2 | GeneCards |  |  |  |  |  |  |  |  |  |  |  |  |  |  |  |  | CSF2 |
| CYP26A1 | GeneCards |  |  |  |  |  |  |  |  |  |  |  |  |  |  |  |  | CSF3 |
| CYP2C19 | GeneCards |  |  |  |  |  |  |  |  |  |  |  |  |  |  |  |  | CSK |
| CYP3A4 | GeneCards |  |  |  |  |  |  |  |  |  |  |  |  |  |  |  |  | CST6 |
| CYSLTR2 | GeneCards |  |  |  |  |  |  |  |  |  |  |  |  |  |  |  |  | CTLA4 |
| DCD | GeneCards |  |  |  |  |  |  |  |  |  |  |  |  |  |  |  |  | CTNNB1 |
| DDB2 | GeneCards |  |  |  |  |  |  |  |  |  |  |  |  |  |  |  |  | CTSG |
| DEFB1 | GeneCards |  |  |  |  |  |  |  |  |  |  |  |  |  |  |  |  | CTSW |
| DEFB103B | GeneCards |  |  |  |  |  |  |  |  |  |  |  |  |  |  |  |  | CXCL8 |
| DEFB4A | GeneCards |  |  |  |  |  |  |  |  |  |  |  |  |  |  |  |  | CXCR3 |
| DENND6B | GeneCards |  |  |  |  |  |  |  |  |  |  |  |  |  |  |  |  | CXCR5 |
| DOCK2 | GeneCards |  |  |  |  |  |  |  |  |  |  |  |  |  |  |  |  | CYB5A |
| DPF2 | GeneCards |  |  |  |  |  |  |  |  |  |  |  |  |  |  |  |  | CYCS |
| DPP4 | GeneCards |  |  |  |  |  |  |  |  |  |  |  |  |  |  |  |  | CYP11B1 |
| DRAP1 | GeneCards |  |  |  |  |  |  |  |  |  |  |  |  |  |  |  |  | CYP17A1 |
| DSC1 | GeneCards |  |  |  |  |  |  |  |  |  |  |  |  |  |  |  |  | CYP19A1 |
| DSC2 | GeneCards |  |  |  |  |  |  |  |  |  |  |  |  |  |  |  |  | CYP1A1 |
| DSC3 | GeneCards |  |  |  |  |  |  |  |  |  |  |  |  |  |  |  |  | CYP1A2 |
| DSG1 | GeneCards |  |  |  |  |  |  |  |  |  |  |  |  |  |  |  |  | CYP21A2 |
| DSG2 | GeneCards |  |  |  |  |  |  |  |  |  |  |  |  |  |  |  |  | CYP26A1 |
| DSG3 | GeneCards |  |  |  |  |  |  |  |  |  |  |  |  |  |  |  |  | CYP26B1 |
| DSG4 | GeneCards |  |  |  |  |  |  |  |  |  |  |  |  |  |  |  |  | CYP26C1 |
| DSP | GeneCards |  |  |  |  |  |  |  |  |  |  |  |  |  |  |  |  | CYP2C19 |
| DST | GeneCards |  |  |  |  |  |  |  |  |  |  |  |  |  |  |  |  | CYP3A4 |
| EFEMP2 | GeneCards |  |  |  |  |  |  |  |  |  |  |  |  |  |  |  |  | CYSLTR2 |
| EGF | GeneCards |  |  |  |  |  |  |  |  |  |  |  |  |  |  |  |  | DCD |
| EGFR | GeneCards |  |  |  |  |  |  |  |  |  |  |  |  |  |  |  |  | DCN |
| EHBP1L1 | GeneCards |  |  |  |  |  |  |  |  |  |  |  |  |  |  |  |  | DDB2 |
| EIF1AD | GeneCards |  |  |  |  |  |  |  |  |  |  |  |  |  |  |  |  | DEFB1 |
| ELANE | GeneCards |  |  |  |  |  |  |  |  |  |  |  |  |  |  |  |  | DEFB103B |
| ELN | GeneCards |  |  |  |  |  |  |  |  |  |  |  |  |  |  |  |  | DEFB4A |
| ENO1 | GeneCards |  |  |  |  |  |  |  |  |  |  |  |  |  |  |  |  | DEFB4B |
| EPB41L3 | GeneCards |  |  |  |  |  |  |  |  |  |  |  |  |  |  |  |  | DENND6B |
| ERAP1 | GeneCards |  |  |  |  |  |  |  |  |  |  |  |  |  |  |  |  | DNMT1 |
| ERAP2 | GeneCards |  |  |  |  |  |  |  |  |  |  |  |  |  |  |  |  | DOCK2 |
| ERBB2 | GeneCards |  |  |  |  |  |  |  |  |  |  |  |  |  |  |  |  | DPF2 |
| ERBB3 | GeneCards |  |  |  |  |  |  |  |  |  |  |  |  |  |  |  |  | DPP4 |
| ERBB4 | GeneCards |  |  |  |  |  |  |  |  |  |  |  |  |  |  |  |  | DRAP1 |
| ERI1 | GeneCards |  |  |  |  |  |  |  |  |  |  |  |  |  |  |  |  | DSC1 |
| ESR1 | GeneCards |  |  |  |  |  |  |  |  |  |  |  |  |  |  |  |  | DSC2 |
| ETV5 | GeneCards |  |  |  |  |  |  |  |  |  |  |  |  |  |  |  |  | DSC3 |
| EXOSC7 | GeneCards |  |  |  |  |  |  |  |  |  |  |  |  |  |  |  |  | DSG1 |
| EZR | GeneCards |  |  |  |  |  |  |  |  |  |  |  |  |  |  |  |  | DSG2 |
| F2 | GeneCards |  |  |  |  |  |  |  |  |  |  |  |  |  |  |  |  | DSG3 |
| F2R | GeneCards |  |  |  |  |  |  |  |  |  |  |  |  |  |  |  |  | DSG4 |
| F2RL1 | GeneCards |  |  |  |  |  |  |  |  |  |  |  |  |  |  |  |  | DSP |
| F3 | GeneCards |  |  |  |  |  |  |  |  |  |  |  |  |  |  |  |  | DST |
| FAM136A | GeneCards |  |  |  |  |  |  |  |  |  |  |  |  |  |  |  |  | EEF1D |
| FAM89B | GeneCards |  |  |  |  |  |  |  |  |  |  |  |  |  |  |  |  | EFEMP2 |
| FAS | GeneCards |  |  |  |  |  |  |  |  |  |  |  |  |  |  |  |  | EGF |
| FCER2 | GeneCards |  |  |  |  |  |  |  |  |  |  |  |  |  |  |  |  | EGFR |
| FCGR3A | GeneCards |  |  |  |  |  |  |  |  |  |  |  |  |  |  |  |  | EHBP1L1 |
| FCGR3B | GeneCards |  |  |  |  |  |  |  |  |  |  |  |  |  |  |  |  | EIF1AD |
| FDX1 | GeneCards |  |  |  |  |  |  |  |  |  |  |  |  |  |  |  |  | ELANE |
| FGD2 | GeneCards |  |  |  |  |  |  |  |  |  |  |  |  |  |  |  |  | ELN |
| FGF2 | GeneCards |  |  |  |  |  |  |  |  |  |  |  |  |  |  |  |  | ENO1 |
| FGF7 | GeneCards |  |  |  |  |  |  |  |  |  |  |  |  |  |  |  |  | EPB41L3 |
| FGFR2 | GeneCards |  |  |  |  |  |  |  |  |  |  |  |  |  |  |  |  | ERAP1 |
| FIBP | GeneCards |  |  |  |  |  |  |  |  |  |  |  |  |  |  |  |  | ERAP2 |
| FICD | GeneCards |  |  |  |  |  |  |  |  |  |  |  |  |  |  |  |  | ERBB2 |
| FIGLA | GeneCards |  |  |  |  |  |  |  |  |  |  |  |  |  |  |  |  | ERBB3 |
| FLG | GeneCards |  |  |  |  |  |  |  |  |  |  |  |  |  |  |  |  | ERBB4 |
| FOSL1 | GeneCards |  |  |  |  |  |  |  |  |  |  |  |  |  |  |  |  | ERI1 |
| FOXI1 | GeneCards |  |  |  |  |  |  |  |  |  |  |  |  |  |  |  |  | ESR1 |
| FOXO1 | GeneCards |  |  |  |  |  |  |  |  |  |  |  |  |  |  |  |  | ESRRA |
| FOXP3 | GeneCards |  |  |  |  |  |  |  |  |  |  |  |  |  |  |  |  | ETFDH |
| FRMD8 | GeneCards |  |  |  |  |  |  |  |  |  |  |  |  |  |  |  |  | ETV5 |
| FSHB | GeneCards |  |  |  |  |  |  |  |  |  |  |  |  |  |  |  |  | EXOSC7 |
| FST | GeneCards |  |  |  |  |  |  |  |  |  |  |  |  |  |  |  |  | EZR |
| G6PD | GeneCards |  |  |  |  |  |  |  |  |  |  |  |  |  |  |  |  | F10 |
| GAL3ST3 | GeneCards |  |  |  |  |  |  |  |  |  |  |  |  |  |  |  |  | F2 |
| GAPDH | GeneCards |  |  |  |  |  |  |  |  |  |  |  |  |  |  |  |  | F2R |
| GGT1 | GeneCards |  |  |  |  |  |  |  |  |  |  |  |  |  |  |  |  | F2RL1 |
| GGT5 | GeneCards |  |  |  |  |  |  |  |  |  |  |  |  |  |  |  |  | F3 |
| GH1 | GeneCards |  |  |  |  |  |  |  |  |  |  |  |  |  |  |  |  | F7 |
| GJB2 | GeneCards |  |  |  |  |  |  |  |  |  |  |  |  |  |  |  |  | FAM136A |
| GSTM1 | GeneCards |  |  |  |  |  |  |  |  |  |  |  |  |  |  |  |  | FAM89B |
| GSTT1 | GeneCards |  |  |  |  |  |  |  |  |  |  |  |  |  |  |  |  | FAS |
| GUCD1 | GeneCards |  |  |  |  |  |  |  |  |  |  |  |  |  |  |  |  | FCER2 |
| GXYLT1 | GeneCards |  |  |  |  |  |  |  |  |  |  |  |  |  |  |  |  | FCGR3A |
| H4-16 | GeneCards |  |  |  |  |  |  |  |  |  |  |  |  |  |  |  |  | FCGR3B |
| H6PD | GeneCards |  |  |  |  |  |  |  |  |  |  |  |  |  |  |  |  | FDX1 |
| HAMP | GeneCards |  |  |  |  |  |  |  |  |  |  |  |  |  |  |  |  | FECH |
| HBEGF | GeneCards |  |  |  |  |  |  |  |  |  |  |  |  |  |  |  |  | FGA |
| HDAC10 | GeneCards |  |  |  |  |  |  |  |  |  |  |  |  |  |  |  |  | FGB |
| HGD | GeneCards |  |  |  |  |  |  |  |  |  |  |  |  |  |  |  |  | FGD2 |
| HGS | GeneCards |  |  |  |  |  |  |  |  |  |  |  |  |  |  |  |  | FGF2 |
| HLA-A | GeneCards |  |  |  |  |  |  |  |  |  |  |  |  |  |  |  |  | FGF7 |
| HLA-B | GeneCards |  |  |  |  |  |  |  |  |  |  |  |  |  |  |  |  | FGFR2 |
| HLA-C | GeneCards |  |  |  |  |  |  |  |  |  |  |  |  |  |  |  |  | FGG |
| HLA-DPB1 | GeneCards |  |  |  |  |  |  |  |  |  |  |  |  |  |  |  |  | FIBP |
| HLA-DRB1 | GeneCards |  |  |  |  |  |  |  |  |  |  |  |  |  |  |  |  | FICD |
| HLA-DRB5 | GeneCards |  |  |  |  |  |  |  |  |  |  |  |  |  |  |  |  | FIGLA |
| HLA-G | GeneCards |  |  |  |  |  |  |  |  |  |  |  |  |  |  |  |  | FLG |
| HMOX1 | GeneCards |  |  |  |  |  |  |  |  |  |  |  |  |  |  |  |  | FOS |
| HP | GeneCards |  |  |  |  |  |  |  |  |  |  |  |  |  |  |  |  | FOSL1 |
| HRAS | GeneCards |  |  |  |  |  |  |  |  |  |  |  |  |  |  |  |  | FOXI1 |
| HRH1 | GeneCards |  |  |  |  |  |  |  |  |  |  |  |  |  |  |  |  | FOXO1 |
| HSD17B13 | GeneCards |  |  |  |  |  |  |  |  |  |  |  |  |  |  |  |  | FOXO3 |
| HSD17B3 | GeneCards |  |  |  |  |  |  |  |  |  |  |  |  |  |  |  |  | FOXP3 |
| HSD3B1 | GeneCards |  |  |  |  |  |  |  |  |  |  |  |  |  |  |  |  | FRMD8 |
| HSPA1A | GeneCards |  |  |  |  |  |  |  |  |  |  |  |  |  |  |  |  | FSHB |
| HSPA8 | GeneCards |  |  |  |  |  |  |  |  |  |  |  |  |  |  |  |  | FST |
| HSPB1 | GeneCards |  |  |  |  |  |  |  |  |  |  |  |  |  |  |  |  | G6PD |
| HSPD1 | GeneCards |  |  |  |  |  |  |  |  |  |  |  |  |  |  |  |  | GAL3ST3 |
| HSPG2 | GeneCards |  |  |  |  |  |  |  |  |  |  |  |  |  |  |  |  | GALNS |
| ICAM1 | GeneCards |  |  |  |  |  |  |  |  |  |  |  |  |  |  |  |  | GAPDH |
| ICOSLG | GeneCards |  |  |  |  |  |  |  |  |  |  |  |  |  |  |  |  | GAST |
| IDUA | GeneCards |  |  |  |  |  |  |  |  |  |  |  |  |  |  |  |  | GCLC |
| IFNA1 | GeneCards |  |  |  |  |  |  |  |  |  |  |  |  |  |  |  |  | GGT1 |
| IFNA2 | GeneCards |  |  |  |  |  |  |  |  |  |  |  |  |  |  |  |  | GGT5 |
| IFNB1 | GeneCards |  |  |  |  |  |  |  |  |  |  |  |  |  |  |  |  | GH1 |
| IFNG | GeneCards |  |  |  |  |  |  |  |  |  |  |  |  |  |  |  |  | GJB2 |
| IGF1 | GeneCards |  |  |  |  |  |  |  |  |  |  |  |  |  |  |  |  | GPT |
| IGF1R | GeneCards |  |  |  |  |  |  |  |  |  |  |  |  |  |  |  |  | GREB1 |
| IGFBP3 | GeneCards |  |  |  |  |  |  |  |  |  |  |  |  |  |  |  |  | GSR |
| IL10 | GeneCards |  |  |  |  |  |  |  |  |  |  |  |  |  |  |  |  | GSTM1 |
| IL10RA | GeneCards |  |  |  |  |  |  |  |  |  |  |  |  |  |  |  |  | GSTP1 |
| IL12A | GeneCards |  |  |  |  |  |  |  |  |  |  |  |  |  |  |  |  | GSTT1 |
| IL12RB1 | GeneCards |  |  |  |  |  |  |  |  |  |  |  |  |  |  |  |  | GUCD1 |
| IL12RB2 | GeneCards |  |  |  |  |  |  |  |  |  |  |  |  |  |  |  |  | GXYLT1 |
| IL13 | GeneCards |  |  |  |  |  |  |  |  |  |  |  |  |  |  |  |  | H4-16 |
| IL17A | GeneCards |  |  |  |  |  |  |  |  |  |  |  |  |  |  |  |  | H6PD |
| IL17F | GeneCards |  |  |  |  |  |  |  |  |  |  |  |  |  |  |  |  | HAMP |
| IL17REL | GeneCards |  |  |  |  |  |  |  |  |  |  |  |  |  |  |  |  | HBEGF |
| IL18 | GeneCards |  |  |  |  |  |  |  |  |  |  |  |  |  |  |  |  | HDAC10 |
| IL1A | GeneCards |  |  |  |  |  |  |  |  |  |  |  |  |  |  |  |  | HDAC8 |
| IL1B | GeneCards |  |  |  |  |  |  |  |  |  |  |  |  |  |  |  |  | HDAC9 |
| IL1R1 | GeneCards |  |  |  |  |  |  |  |  |  |  |  |  |  |  |  |  | HGD |
| IL1RAPL2 | GeneCards |  |  |  |  |  |  |  |  |  |  |  |  |  |  |  |  | HGS |
| IL1RN | GeneCards |  |  |  |  |  |  |  |  |  |  |  |  |  |  |  |  | HLA-A |
| IL2 | GeneCards |  |  |  |  |  |  |  |  |  |  |  |  |  |  |  |  | HLA-B |
| IL22 | GeneCards |  |  |  |  |  |  |  |  |  |  |  |  |  |  |  |  | HLA-C |
| IL23R | GeneCards |  |  |  |  |  |  |  |  |  |  |  |  |  |  |  |  | HLA-DPB1 |
| IL2RA | GeneCards |  |  |  |  |  |  |  |  |  |  |  |  |  |  |  |  | HLA-DRB1 |
| IL2RB | GeneCards |  |  |  |  |  |  |  |  |  |  |  |  |  |  |  |  | HLA-DRB5 |
| IL33 | GeneCards |  |  |  |  |  |  |  |  |  |  |  |  |  |  |  |  | HLA-G |
| IL37 | GeneCards |  |  |  |  |  |  |  |  |  |  |  |  |  |  |  |  | HMGCS1 |
| IL4 | GeneCards |  |  |  |  |  |  |  |  |  |  |  |  |  |  |  |  | HMOX1 |
| IL4R | GeneCards |  |  |  |  |  |  |  |  |  |  |  |  |  |  |  |  | HP |
| IL5 | GeneCards |  |  |  |  |  |  |  |  |  |  |  |  |  |  |  |  | HPGD |
| IL6 | GeneCards |  |  |  |  |  |  |  |  |  |  |  |  |  |  |  |  | HRAS |
| IL7 | GeneCards |  |  |  |  |  |  |  |  |  |  |  |  |  |  |  |  | HRH1 |
| INS | GeneCards |  |  |  |  |  |  |  |  |  |  |  |  |  |  |  |  | HSD11B2 |
| INSYN2B | GeneCards |  |  |  |  |  |  |  |  |  |  |  |  |  |  |  |  | HSD17B13 |
| IRF4 | GeneCards |  |  |  |  |  |  |  |  |  |  |  |  |  |  |  |  | HSD17B3 |
| IRF8 | GeneCards |  |  |  |  |  |  |  |  |  |  |  |  |  |  |  |  | HSD3B1 |
| ISCU | GeneCards |  |  |  |  |  |  |  |  |  |  |  |  |  |  |  |  | HSPA1A |
| ITGA1 | GeneCards |  |  |  |  |  |  |  |  |  |  |  |  |  |  |  |  | HSPA8 |
| ITGA2 | GeneCards |  |  |  |  |  |  |  |  |  |  |  |  |  |  |  |  | HSPB1 |
| ITGB2 | GeneCards |  |  |  |  |  |  |  |  |  |  |  |  |  |  |  |  | HSPD1 |
| ITLN1 | GeneCards |  |  |  |  |  |  |  |  |  |  |  |  |  |  |  |  | HSPG2 |
| IVL | GeneCards |  |  |  |  |  |  |  |  |  |  |  |  |  |  |  |  | ICAM1 |
| JAK2 | GeneCards |  |  |  |  |  |  |  |  |  |  |  |  |  |  |  |  | ICOSLG |
| JUN | GeneCards |  |  |  |  |  |  |  |  |  |  |  |  |  |  |  |  | IDUA |
| JUP | GeneCards |  |  |  |  |  |  |  |  |  |  |  |  |  |  |  |  | IFITM1 |
| KAT5 | GeneCards |  |  |  |  |  |  |  |  |  |  |  |  |  |  |  |  | IFNA1 |
| KCNIP1 | GeneCards |  |  |  |  |  |  |  |  |  |  |  |  |  |  |  |  | IFNA2 |
| KCNK7 | GeneCards |  |  |  |  |  |  |  |  |  |  |  |  |  |  |  |  | IFNB1 |
| KCNMB1 | GeneCards |  |  |  |  |  |  |  |  |  |  |  |  |  |  |  |  | IFNG |
| KIF15 | GeneCards |  |  |  |  |  |  |  |  |  |  |  |  |  |  |  |  | IGF1 |
| KIR2DL1 | GeneCards |  |  |  |  |  |  |  |  |  |  |  |  |  |  |  |  | IGF1R |
| KIR3DL1 | GeneCards |  |  |  |  |  |  |  |  |  |  |  |  |  |  |  |  | IGFBP3 |
| KLK11 | GeneCards |  |  |  |  |  |  |  |  |  |  |  |  |  |  |  |  | IGFBP7 |
| KLK3 | GeneCards |  |  |  |  |  |  |  |  |  |  |  |  |  |  |  |  | IGHA1 |
| KLK7 | GeneCards |  |  |  |  |  |  |  |  |  |  |  |  |  |  |  |  | IKBKB |
| KRT1 | GeneCards |  |  |  |  |  |  |  |  |  |  |  |  |  |  |  |  | IL10 |
| KRT10 | GeneCards |  |  |  |  |  |  |  |  |  |  |  |  |  |  |  |  | IL10RA |
| KRT13 | GeneCards |  |  |  |  |  |  |  |  |  |  |  |  |  |  |  |  | IL12A |
| KRT14 | GeneCards |  |  |  |  |  |  |  |  |  |  |  |  |  |  |  |  | IL12RB1 |
| KRT16 | GeneCards |  |  |  |  |  |  |  |  |  |  |  |  |  |  |  |  | IL12RB2 |
| KRT17 | GeneCards |  |  |  |  |  |  |  |  |  |  |  |  |  |  |  |  | IL13 |
| KRT19 | GeneCards |  |  |  |  |  |  |  |  |  |  |  |  |  |  |  |  | IL17A |
| KRT4 | GeneCards |  |  |  |  |  |  |  |  |  |  |  |  |  |  |  |  | IL17F |
| KRT5 | GeneCards |  |  |  |  |  |  |  |  |  |  |  |  |  |  |  |  | IL17REL |
| KRT7 | GeneCards |  |  |  |  |  |  |  |  |  |  |  |  |  |  |  |  | IL18 |
| KRT79 | GeneCards |  |  |  |  |  |  |  |  |  |  |  |  |  |  |  |  | IL1A |
| LACTB | GeneCards |  |  |  |  |  |  |  |  |  |  |  |  |  |  |  |  | IL1B |
| LAMC1 | GeneCards |  |  |  |  |  |  |  |  |  |  |  |  |  |  |  |  | IL1R1 |
| LAMC2 | GeneCards |  |  |  |  |  |  |  |  |  |  |  |  |  |  |  |  | IL1RAPL2 |
| LARS2 | GeneCards |  |  |  |  |  |  |  |  |  |  |  |  |  |  |  |  | IL1RN |
| LBR | GeneCards |  |  |  |  |  |  |  |  |  |  |  |  |  |  |  |  | IL2 |
| LCN2 | GeneCards |  |  |  |  |  |  |  |  |  |  |  |  |  |  |  |  | IL22 |
| LCP2 | GeneCards |  |  |  |  |  |  |  |  |  |  |  |  |  |  |  |  | IL23R |
| LCT | GeneCards |  |  |  |  |  |  |  |  |  |  |  |  |  |  |  |  | IL2RA |
| LEP | GeneCards |  |  |  |  |  |  |  |  |  |  |  |  |  |  |  |  | IL2RB |
| LIMD1 | GeneCards |  |  |  |  |  |  |  |  |  |  |  |  |  |  |  |  | IL33 |
| LPL | GeneCards |  |  |  |  |  |  |  |  |  |  |  |  |  |  |  |  | IL37 |
| LTA | GeneCards |  |  |  |  |  |  |  |  |  |  |  |  |  |  |  |  | IL4 |
| LTBP3 | GeneCards |  |  |  |  |  |  |  |  |  |  |  |  |  |  |  |  | IL4R |
| LTF | GeneCards |  |  |  |  |  |  |  |  |  |  |  |  |  |  |  |  | IL5 |
| LZTFL1 | GeneCards |  |  |  |  |  |  |  |  |  |  |  |  |  |  |  |  | IL6 |
| MAP3K11 | GeneCards |  |  |  |  |  |  |  |  |  |  |  |  |  |  |  |  | IL7 |
| MAPK1 | GeneCards |  |  |  |  |  |  |  |  |  |  |  |  |  |  |  |  | INS |
| MAPK11 | GeneCards |  |  |  |  |  |  |  |  |  |  |  |  |  |  |  |  | INSYN2B |
| MAPK12 | GeneCards |  |  |  |  |  |  |  |  |  |  |  |  |  |  |  |  | IRF4 |
| MAPK8 | GeneCards |  |  |  |  |  |  |  |  |  |  |  |  |  |  |  |  | IRF8 |
| MB | GeneCards |  |  |  |  |  |  |  |  |  |  |  |  |  |  |  |  | ISCU |
| MBL2 | GeneCards |  |  |  |  |  |  |  |  |  |  |  |  |  |  |  |  | ITGA1 |
| MC1R | GeneCards |  |  |  |  |  |  |  |  |  |  |  |  |  |  |  |  | ITGA2 |
| MC5R | GeneCards |  |  |  |  |  |  |  |  |  |  |  |  |  |  |  |  | ITGB2 |
| MDGA1 | GeneCards |  |  |  |  |  |  |  |  |  |  |  |  |  |  |  |  | ITLN1 |
| MFHAS1 | GeneCards |  |  |  |  |  |  |  |  |  |  |  |  |  |  |  |  | IVL |
| MICA | GeneCards |  |  |  |  |  |  |  |  |  |  |  |  |  |  |  |  | JAK2 |
| MICB | GeneCards |  |  |  |  |  |  |  |  |  |  |  |  |  |  |  |  | JUN |
| MIF | GeneCards |  |  |  |  |  |  |  |  |  |  |  |  |  |  |  |  | JUP |
| MLC1 | GeneCards |  |  |  |  |  |  |  |  |  |  |  |  |  |  |  |  | KAT5 |
| MME | GeneCards |  |  |  |  |  |  |  |  |  |  |  |  |  |  |  |  | KCNIP1 |
| MMP1 | GeneCards |  |  |  |  |  |  |  |  |  |  |  |  |  |  |  |  | KCNK7 |
| MMP13 | GeneCards |  |  |  |  |  |  |  |  |  |  |  |  |  |  |  |  | KCNMB1 |
| MMP2 | GeneCards |  |  |  |  |  |  |  |  |  |  |  |  |  |  |  |  | KIF15 |
| MMP3 | GeneCards |  |  |  |  |  |  |  |  |  |  |  |  |  |  |  |  | KIR2DL1 |
| MMP9 | GeneCards |  |  |  |  |  |  |  |  |  |  |  |  |  |  |  |  | KIR3DL1 |
| MOCS2 | GeneCards |  |  |  |  |  |  |  |  |  |  |  |  |  |  |  |  | KLK11 |
| MOV10L1 | GeneCards |  |  |  |  |  |  |  |  |  |  |  |  |  |  |  |  | KLK3 |
| MPO | GeneCards |  |  |  |  |  |  |  |  |  |  |  |  |  |  |  |  | KLK7 |
| MSN | GeneCards |  |  |  |  |  |  |  |  |  |  |  |  |  |  |  |  | KPNA1 |
| MTCH1 | GeneCards |  |  |  |  |  |  |  |  |  |  |  |  |  |  |  |  | KRT1 |
| MTHFR | GeneCards |  |  |  |  |  |  |  |  |  |  |  |  |  |  |  |  | KRT10 |
| MTOR | GeneCards |  |  |  |  |  |  |  |  |  |  |  |  |  |  |  |  | KRT13 |
| MUC1 | GeneCards |  |  |  |  |  |  |  |  |  |  |  |  |  |  |  |  | KRT14 |
| MUS81 | GeneCards |  |  |  |  |  |  |  |  |  |  |  |  |  |  |  |  | KRT16 |
| MYC | GeneCards |  |  |  |  |  |  |  |  |  |  |  |  |  |  |  |  | KRT17 |
| MYD88 | GeneCards |  |  |  |  |  |  |  |  |  |  |  |  |  |  |  |  | KRT19 |
| MYH11 | GeneCards |  |  |  |  |  |  |  |  |  |  |  |  |  |  |  |  | KRT4 |
| NAT2 | GeneCards |  |  |  |  |  |  |  |  |  |  |  |  |  |  |  |  | KRT5 |
| NDUFS4 | GeneCards |  |  |  |  |  |  |  |  |  |  |  |  |  |  |  |  | KRT7 |
| NEK9 | GeneCards |  |  |  |  |  |  |  |  |  |  |  |  |  |  |  |  | KRT79 |
| NF1 | GeneCards |  |  |  |  |  |  |  |  |  |  |  |  |  |  |  |  | LACTB |
| NF2 | GeneCards |  |  |  |  |  |  |  |  |  |  |  |  |  |  |  |  | LAMC1 |
| NFKB1 | GeneCards |  |  |  |  |  |  |  |  |  |  |  |  |  |  |  |  | LAMC2 |
| NFKBIA | GeneCards |  |  |  |  |  |  |  |  |  |  |  |  |  |  |  |  | LARS2 |
| NKIRAS1 | GeneCards |  |  |  |  |  |  |  |  |  |  |  |  |  |  |  |  | LBR |
| NLRP3 | GeneCards |  |  |  |  |  |  |  |  |  |  |  |  |  |  |  |  | LCN2 |
| NOD2 | GeneCards |  |  |  |  |  |  |  |  |  |  |  |  |  |  |  |  | LCP2 |
| NOS2 | GeneCards |  |  |  |  |  |  |  |  |  |  |  |  |  |  |  |  | LCT |
| NOTCH1 | GeneCards |  |  |  |  |  |  |  |  |  |  |  |  |  |  |  |  | LEP |
| NOTCH2 | GeneCards |  |  |  |  |  |  |  |  |  |  |  |  |  |  |  |  | LGALS7 |
| NPPB | GeneCards |  |  |  |  |  |  |  |  |  |  |  |  |  |  |  |  | LHB |
| NR0B1 | GeneCards |  |  |  |  |  |  |  |  |  |  |  |  |  |  |  |  | LIMD1 |
| NR1D2 | GeneCards |  |  |  |  |  |  |  |  |  |  |  |  |  |  |  |  | LPL |
| NR1H2 | GeneCards |  |  |  |  |  |  |  |  |  |  |  |  |  |  |  |  | LTA |
| NR3C1 | GeneCards |  |  |  |  |  |  |  |  |  |  |  |  |  |  |  |  | LTBP3 |
| NR3C2 | GeneCards |  |  |  |  |  |  |  |  |  |  |  |  |  |  |  |  | LTF |
| NR5A1 | GeneCards |  |  |  |  |  |  |  |  |  |  |  |  |  |  |  |  | LYZ |
| NRP1 | GeneCards |  |  |  |  |  |  |  |  |  |  |  |  |  |  |  |  | LZTFL1 |
| OPN4 | GeneCards |  |  |  |  |  |  |  |  |  |  |  |  |  |  |  |  | MAP3K11 |
| PANX2 | GeneCards |  |  |  |  |  |  |  |  |  |  |  |  |  |  |  |  | MAPK1 |
| PAPOLG | GeneCards |  |  |  |  |  |  |  |  |  |  |  |  |  |  |  |  | MAPK11 |
| PCBP1 | GeneCards |  |  |  |  |  |  |  |  |  |  |  |  |  |  |  |  | MAPK12 |
| PCYOX1 | GeneCards |  |  |  |  |  |  |  |  |  |  |  |  |  |  |  |  | MAPK3 |
| PELO | GeneCards |  |  |  |  |  |  |  |  |  |  |  |  |  |  |  |  | MAPK8 |
| PGR | GeneCards |  |  |  |  |  |  |  |  |  |  |  |  |  |  |  |  | MB |
| PI16 | GeneCards |  |  |  |  |  |  |  |  |  |  |  |  |  |  |  |  | MBL2 |
| PIK3C2A | GeneCards |  |  |  |  |  |  |  |  |  |  |  |  |  |  |  |  | MBNL1 |
| PIK3CG | GeneCards |  |  |  |  |  |  |  |  |  |  |  |  |  |  |  |  | MC1R |
| PIM1 | GeneCards |  |  |  |  |  |  |  |  |  |  |  |  |  |  |  |  | MC5R |
| PIM3 | GeneCards |  |  |  |  |  |  |  |  |  |  |  |  |  |  |  |  | MDGA1 |
| PIWIL3 | GeneCards |  |  |  |  |  |  |  |  |  |  |  |  |  |  |  |  | MDK |
| PLA2G2A | GeneCards |  |  |  |  |  |  |  |  |  |  |  |  |  |  |  |  | MEFV |
| PLXNB2 | GeneCards |  |  |  |  |  |  |  |  |  |  |  |  |  |  |  |  | MFHAS1 |
| POMC | GeneCards |  |  |  |  |  |  |  |  |  |  |  |  |  |  |  |  | MICA |
| PON1 | GeneCards |  |  |  |  |  |  |  |  |  |  |  |  |  |  |  |  | MICB |
| PPARA | GeneCards |  |  |  |  |  |  |  |  |  |  |  |  |  |  |  |  | MIF |
| PPARG | GeneCards |  |  |  |  |  |  |  |  |  |  |  |  |  |  |  |  | MKI67 |
| PPIL1 | GeneCards |  |  |  |  |  |  |  |  |  |  |  |  |  |  |  |  | MLC1 |
| PPP1R3B | GeneCards |  |  |  |  |  |  |  |  |  |  |  |  |  |  |  |  | MME |
| PPP6R2 | GeneCards |  |  |  |  |  |  |  |  |  |  |  |  |  |  |  |  | MMP1 |
| PRDM10 | GeneCards |  |  |  |  |  |  |  |  |  |  |  |  |  |  |  |  | MMP13 |
| PRDM4 | GeneCards |  |  |  |  |  |  |  |  |  |  |  |  |  |  |  |  | MMP2 |
| PRL | GeneCards |  |  |  |  |  |  |  |  |  |  |  |  |  |  |  |  | MMP3 |
| PRTN3 | GeneCards |  |  |  |  |  |  |  |  |  |  |  |  |  |  |  |  | MMP9 |
| PSAP | GeneCards |  |  |  |  |  |  |  |  |  |  |  |  |  |  |  |  | MOCS2 |
| PSTPIP1 | GeneCards |  |  |  |  |  |  |  |  |  |  |  |  |  |  |  |  | MOV10L1 |
| PTEN | GeneCards |  |  |  |  |  |  |  |  |  |  |  |  |  |  |  |  | MPO |
| PTGER1 | GeneCards |  |  |  |  |  |  |  |  |  |  |  |  |  |  |  |  | MSN |
| PTGER3 | GeneCards |  |  |  |  |  |  |  |  |  |  |  |  |  |  |  |  | MTCH1 |
| PTGER4 | GeneCards |  |  |  |  |  |  |  |  |  |  |  |  |  |  |  |  | MTHFR |
| PTGS2 | GeneCards |  |  |  |  |  |  |  |  |  |  |  |  |  |  |  |  | MTOR |
| PTPN12 | GeneCards |  |  |  |  |  |  |  |  |  |  |  |  |  |  |  |  | MUC1 |
| PTPN18 | GeneCards |  |  |  |  |  |  |  |  |  |  |  |  |  |  |  |  | MUS81 |
| PTPN22 | GeneCards |  |  |  |  |  |  |  |  |  |  |  |  |  |  |  |  | MYC |
| PWP1 | GeneCards |  |  |  |  |  |  |  |  |  |  |  |  |  |  |  |  | MYD88 |
| PXN | GeneCards |  |  |  |  |  |  |  |  |  |  |  |  |  |  |  |  | MYH11 |
| RAB4A | GeneCards |  |  |  |  |  |  |  |  |  |  |  |  |  |  |  |  | NAP1L2 |
| RARA | GeneCards |  |  |  |  |  |  |  |  |  |  |  |  |  |  |  |  | NAT2 |
| RARB | GeneCards |  |  |  |  |  |  |  |  |  |  |  |  |  |  |  |  | NCSTN |
| RBP4 | GeneCards |  |  |  |  |  |  |  |  |  |  |  |  |  |  |  |  | NDUFS4 |
| RDX | GeneCards |  |  |  |  |  |  |  |  |  |  |  |  |  |  |  |  | NEK9 |
| RETN | GeneCards |  |  |  |  |  |  |  |  |  |  |  |  |  |  |  |  | NF1 |
| RHO | GeneCards |  |  |  |  |  |  |  |  |  |  |  |  |  |  |  |  | NF2 |
| RNF8 | GeneCards |  |  |  |  |  |  |  |  |  |  |  |  |  |  |  |  | NFKB1 |
| RPL15 | GeneCards |  |  |  |  |  |  |  |  |  |  |  |  |  |  |  |  | NFKBIA |
| RRP15 | GeneCards |  |  |  |  |  |  |  |  |  |  |  |  |  |  |  |  | NKIRAS1 |
| RXRA | GeneCards |  |  |  |  |  |  |  |  |  |  |  |  |  |  |  |  | NKX2-1 |
| S100A7 | GeneCards |  |  |  |  |  |  |  |  |  |  |  |  |  |  |  |  | NLRP3 |
| S100A8 | GeneCards |  |  |  |  |  |  |  |  |  |  |  |  |  |  |  |  | NOD2 |
| S100A9 | GeneCards |  |  |  |  |  |  |  |  |  |  |  |  |  |  |  |  | NOS2 |
| SACM1L | GeneCards |  |  |  |  |  |  |  |  |  |  |  |  |  |  |  |  | NOS3 |
| SART3 | GeneCards |  |  |  |  |  |  |  |  |  |  |  |  |  |  |  |  | NOTCH1 |
| SDCBP | GeneCards |  |  |  |  |  |  |  |  |  |  |  |  |  |  |  |  | NOTCH2 |
| SDHD | GeneCards |  |  |  |  |  |  |  |  |  |  |  |  |  |  |  |  | NPPB |
| SEC24C | GeneCards |  |  |  |  |  |  |  |  |  |  |  |  |  |  |  |  | NR0B1 |
| SELE | GeneCards |  |  |  |  |  |  |  |  |  |  |  |  |  |  |  |  | NR1D2 |
| SELENOO | GeneCards |  |  |  |  |  |  |  |  |  |  |  |  |  |  |  |  | NR1H2 |
| SELL | GeneCards |  |  |  |  |  |  |  |  |  |  |  |  |  |  |  |  | NR3C1 |
| SELP | GeneCards |  |  |  |  |  |  |  |  |  |  |  |  |  |  |  |  | NR3C2 |
| SELPLG | GeneCards |  |  |  |  |  |  |  |  |  |  |  |  |  |  |  |  | NR5A1 |
| SERPINA6 | GeneCards |  |  |  |  |  |  |  |  |  |  |  |  |  |  |  |  | NRP1 |
| SGO1 | GeneCards |  |  |  |  |  |  |  |  |  |  |  |  |  |  |  |  | ODC1 |
| SGSM1 | GeneCards |  |  |  |  |  |  |  |  |  |  |  |  |  |  |  |  | OPN4 |
| SHBG | GeneCards |  |  |  |  |  |  |  |  |  |  |  |  |  |  |  |  | PAGR1 |
| SLAMF1 | GeneCards |  |  |  |  |  |  |  |  |  |  |  |  |  |  |  |  | PANX2 |
| SLC6A20 | GeneCards |  |  |  |  |  |  |  |  |  |  |  |  |  |  |  |  | PAPOLG |
| SLC9A3R1 | GeneCards |  |  |  |  |  |  |  |  |  |  |  |  |  |  |  |  | PCBP1 |
| SLCO1B1 | GeneCards |  |  |  |  |  |  |  |  |  |  |  |  |  |  |  |  | PCK1 |
| SLPI | GeneCards |  |  |  |  |  |  |  |  |  |  |  |  |  |  |  |  | PCNX3 |
| SNRPD3 | GeneCards |  |  |  |  |  |  |  |  |  |  |  |  |  |  |  |  | PCYOX1 |
| SNRPG | GeneCards |  |  |  |  |  |  |  |  |  |  |  |  |  |  |  |  | PELO |
| SOD1 | GeneCards |  |  |  |  |  |  |  |  |  |  |  |  |  |  |  |  | PER1 |
| SOD2 | GeneCards |  |  |  |  |  |  |  |  |  |  |  |  |  |  |  |  | PGR |
| SOX9 | GeneCards |  |  |  |  |  |  |  |  |  |  |  |  |  |  |  |  | PI16 |
| SPECC1L | GeneCards |  |  |  |  |  |  |  |  |  |  |  |  |  |  |  |  | PIK3C2A |
| SPP1 | GeneCards |  |  |  |  |  |  |  |  |  |  |  |  |  |  |  |  | PIK3CG |
| SPTBN1 | GeneCards |  |  |  |  |  |  |  |  |  |  |  |  |  |  |  |  | PIM1 |
| SRC | GeneCards |  |  |  |  |  |  |  |  |  |  |  |  |  |  |  |  | PIM3 |
| SRD5A1 | GeneCards |  |  |  |  |  |  |  |  |  |  |  |  |  |  |  |  | PINX1 |
| SREBF1 | GeneCards |  |  |  |  |  |  |  |  |  |  |  |  |  |  |  |  | PIWIL3 |
| SRY | GeneCards |  |  |  |  |  |  |  |  |  |  |  |  |  |  |  |  | PLA2G2A |
| SST | GeneCards |  |  |  |  |  |  |  |  |  |  |  |  |  |  |  |  | PLAT |
| STAT3 | GeneCards |  |  |  |  |  |  |  |  |  |  |  |  |  |  |  |  | PLG |
| STAT4 | GeneCards |  |  |  |  |  |  |  |  |  |  |  |  |  |  |  |  | PLXNA2 |
| STS | GeneCards |  |  |  |  |  |  |  |  |  |  |  |  |  |  |  |  | PLXNB2 |
| SULT1A3 | GeneCards |  |  |  |  |  |  |  |  |  |  |  |  |  |  |  |  | POMC |
| SUMO4 | GeneCards |  |  |  |  |  |  |  |  |  |  |  |  |  |  |  |  | PON1 |
| SUSD2 | GeneCards |  |  |  |  |  |  |  |  |  |  |  |  |  |  |  |  | PPARA |
| SYK | GeneCards |  |  |  |  |  |  |  |  |  |  |  |  |  |  |  |  | PPARD |
| SYP | GeneCards |  |  |  |  |  |  |  |  |  |  |  |  |  |  |  |  | PPARG |
| TAC1 | GeneCards |  |  |  |  |  |  |  |  |  |  |  |  |  |  |  |  | PPIL1 |
| TBC1D22B | GeneCards |  |  |  |  |  |  |  |  |  |  |  |  |  |  |  |  | PPP1R3B |
| TGFA | GeneCards |  |  |  |  |  |  |  |  |  |  |  |  |  |  |  |  | PPP6R2 |
| TGFB1 | GeneCards |  |  |  |  |  |  |  |  |  |  |  |  |  |  |  |  | PRDM10 |
| TGFB1I1 | GeneCards |  |  |  |  |  |  |  |  |  |  |  |  |  |  |  |  | PRDM4 |
| TGFB2 | GeneCards |  |  |  |  |  |  |  |  |  |  |  |  |  |  |  |  | PRL |
| TGFB3 | GeneCards |  |  |  |  |  |  |  |  |  |  |  |  |  |  |  |  | PROC |
| TGM4 | GeneCards |  |  |  |  |  |  |  |  |  |  |  |  |  |  |  |  | PROS1 |
| TH | GeneCards |  |  |  |  |  |  |  |  |  |  |  |  |  |  |  |  | PRTN3 |
| THBD | GeneCards |  |  |  |  |  |  |  |  |  |  |  |  |  |  |  |  | PSAP |
| THRB | GeneCards |  |  |  |  |  |  |  |  |  |  |  |  |  |  |  |  | PSEN1 |
| TIA1 | GeneCards |  |  |  |  |  |  |  |  |  |  |  |  |  |  |  |  | PSENEN |
| TIMP1 | GeneCards |  |  |  |  |  |  |  |  |  |  |  |  |  |  |  |  | PSTPIP1 |
| TIMP2 | GeneCards |  |  |  |  |  |  |  |  |  |  |  |  |  |  |  |  | PTEN |
| TKT | GeneCards |  |  |  |  |  |  |  |  |  |  |  |  |  |  |  |  | PTGDS |
| TLR1 | GeneCards |  |  |  |  |  |  |  |  |  |  |  |  |  |  |  |  | PTGER1 |
| TLR2 | GeneCards |  |  |  |  |  |  |  |  |  |  |  |  |  |  |  |  | PTGER3 |
| TLR3 | GeneCards |  |  |  |  |  |  |  |  |  |  |  |  |  |  |  |  | PTGER4 |
| TLR4 | GeneCards |  |  |  |  |  |  |  |  |  |  |  |  |  |  |  |  | PTGS2 |
| TLR5 | GeneCards |  |  |  |  |  |  |  |  |  |  |  |  |  |  |  |  | PTPN12 |
| TLR6 | GeneCards |  |  |  |  |  |  |  |  |  |  |  |  |  |  |  |  | PTPN18 |
| TLR7 | GeneCards |  |  |  |  |  |  |  |  |  |  |  |  |  |  |  |  | PTPN22 |
| TLR8 | GeneCards |  |  |  |  |  |  |  |  |  |  |  |  |  |  |  |  | PWP1 |
| TLR9 | GeneCards |  |  |  |  |  |  |  |  |  |  |  |  |  |  |  |  | PXN |
| TMEM119 | GeneCards |  |  |  |  |  |  |  |  |  |  |  |  |  |  |  |  | RAB4A |
| TMEM158 | GeneCards |  |  |  |  |  |  |  |  |  |  |  |  |  |  |  |  | RARA |
| TMEM211 | GeneCards |  |  |  |  |  |  |  |  |  |  |  |  |  |  |  |  | RARB |
| TMEM217 | GeneCards |  |  |  |  |  |  |  |  |  |  |  |  |  |  |  |  | RARG |
| TMEM42 | GeneCards |  |  |  |  |  |  |  |  |  |  |  |  |  |  |  |  | RBP1 |
| TNF | GeneCards |  |  |  |  |  |  |  |  |  |  |  |  |  |  |  |  | RBP4 |
| TNFAIP3 | GeneCards |  |  |  |  |  |  |  |  |  |  |  |  |  |  |  |  | RDX |
| TNFRSF10A | GeneCards |  |  |  |  |  |  |  |  |  |  |  |  |  |  |  |  | RETN |
| TNFRSF1A | GeneCards |  |  |  |  |  |  |  |  |  |  |  |  |  |  |  |  | RHO |
| TNFRSF1B | GeneCards |  |  |  |  |  |  |  |  |  |  |  |  |  |  |  |  | RNASE7 |
| TNFSF13B | GeneCards |  |  |  |  |  |  |  |  |  |  |  |  |  |  |  |  | RNF8 |
| TNIP1 | GeneCards |  |  |  |  |  |  |  |  |  |  |  |  |  |  |  |  | RPL15 |
| TNKS | GeneCards |  |  |  |  |  |  |  |  |  |  |  |  |  |  |  |  | RRP15 |
| TNXB | GeneCards |  |  |  |  |  |  |  |  |  |  |  |  |  |  |  |  | RXRA |
| TP53 | GeneCards |  |  |  |  |  |  |  |  |  |  |  |  |  |  |  |  | RXRG |
| TPMT | GeneCards |  |  |  |  |  |  |  |  |  |  |  |  |  |  |  |  | S100A7 |
| TRABD | GeneCards |  |  |  |  |  |  |  |  |  |  |  |  |  |  |  |  | S100A7A |
| TRAF3IP2 | GeneCards |  |  |  |  |  |  |  |  |  |  |  |  |  |  |  |  | S100A8 |
| TRPV1 | GeneCards |  |  |  |  |  |  |  |  |  |  |  |  |  |  |  |  | S100A9 |
| TUBGCP6 | GeneCards |  |  |  |  |  |  |  |  |  |  |  |  |  |  |  |  | S1PR1 |
| TYR | GeneCards |  |  |  |  |  |  |  |  |  |  |  |  |  |  |  |  | SACM1L |
| UBE2E1 | GeneCards |  |  |  |  |  |  |  |  |  |  |  |  |  |  |  |  | SART3 |
| UBE2E2 | GeneCards |  |  |  |  |  |  |  |  |  |  |  |  |  |  |  |  | SDCBP |
| UPB1 | GeneCards |  |  |  |  |  |  |  |  |  |  |  |  |  |  |  |  | SDHD |
| VAX2 | GeneCards |  |  |  |  |  |  |  |  |  |  |  |  |  |  |  |  | SEC24C |
| VCAM1 | GeneCards |  |  |  |  |  |  |  |  |  |  |  |  |  |  |  |  | SELE |
| VCL | GeneCards |  |  |  |  |  |  |  |  |  |  |  |  |  |  |  |  | SELENOO |
| VDR | GeneCards |  |  |  |  |  |  |  |  |  |  |  |  |  |  |  |  | SELL |
| VEGFA | GeneCards |  |  |  |  |  |  |  |  |  |  |  |  |  |  |  |  | SELP |
| VIM | GeneCards |  |  |  |  |  |  |  |  |  |  |  |  |  |  |  |  | SELPLG |
| WNT5A | GeneCards |  |  |  |  |  |  |  |  |  |  |  |  |  |  |  |  | SEMA4B |
| WSCD2 | GeneCards |  |  |  |  |  |  |  |  |  |  |  |  |  |  |  |  | SERPINA1 |
| WT1 | GeneCards |  |  |  |  |  |  |  |  |  |  |  |  |  |  |  |  | SERPINA6 |
| XDH | GeneCards |  |  |  |  |  |  |  |  |  |  |  |  |  |  |  |  | SERPINC1 |
| YAP1 | GeneCards |  |  |  |  |  |  |  |  |  |  |  |  |  |  |  |  | SERPINE1 |
| ZBED4 | GeneCards |  |  |  |  |  |  |  |  |  |  |  |  |  |  |  |  | SERPING1 |
| ZDHHC3 | GeneCards |  |  |  |  |  |  |  |  |  |  |  |  |  |  |  |  | SETBP1 |
| ZFAND3 | GeneCards |  |  |  |  |  |  |  |  |  |  |  |  |  |  |  |  | SFPQ |
|  |  |  |  |  |  |  |  |  |  |  |  |  |  |  |  |  |  | SGO1 |
|  |  |  |  |  |  |  |  |  |  |  |  |  |  |  |  |  |  | SGSM1 |
|  |  |  |  |  |  |  |  |  |  |  |  |  |  |  |  |  |  | SHBG |
|  |  |  |  |  |  |  |  |  |  |  |  |  |  |  |  |  |  | SLAMF1 |
|  |  |  |  |  |  |  |  |  |  |  |  |  |  |  |  |  |  | SLC6A20 |
|  |  |  |  |  |  |  |  |  |  |  |  |  |  |  |  |  |  | SLC9A3R1 |
|  |  |  |  |  |  |  |  |  |  |  |  |  |  |  |  |  |  | SLCO1B1 |
|  |  |  |  |  |  |  |  |  |  |  |  |  |  |  |  |  |  | SLPI |
|  |  |  |  |  |  |  |  |  |  |  |  |  |  |  |  |  |  | SMIM10L2A |
|  |  |  |  |  |  |  |  |  |  |  |  |  |  |  |  |  |  | SMIM10L2B |
|  |  |  |  |  |  |  |  |  |  |  |  |  |  |  |  |  |  | SNRPD3 |
|  |  |  |  |  |  |  |  |  |  |  |  |  |  |  |  |  |  | SNRPG |
|  |  |  |  |  |  |  |  |  |  |  |  |  |  |  |  |  |  | SOD1 |
|  |  |  |  |  |  |  |  |  |  |  |  |  |  |  |  |  |  | SOD2 |
|  |  |  |  |  |  |  |  |  |  |  |  |  |  |  |  |  |  | SOX9 |
|  |  |  |  |  |  |  |  |  |  |  |  |  |  |  |  |  |  | SPECC1L |
|  |  |  |  |  |  |  |  |  |  |  |  |  |  |  |  |  |  | SPP1 |
|  |  |  |  |  |  |  |  |  |  |  |  |  |  |  |  |  |  | SPTBN1 |
|  |  |  |  |  |  |  |  |  |  |  |  |  |  |  |  |  |  | SRC |
|  |  |  |  |  |  |  |  |  |  |  |  |  |  |  |  |  |  | SRD5A1 |
|  |  |  |  |  |  |  |  |  |  |  |  |  |  |  |  |  |  | SRD5A2 |
|  |  |  |  |  |  |  |  |  |  |  |  |  |  |  |  |  |  | SREBF1 |
|  |  |  |  |  |  |  |  |  |  |  |  |  |  |  |  |  |  | SRGN |
|  |  |  |  |  |  |  |  |  |  |  |  |  |  |  |  |  |  | SRY |
|  |  |  |  |  |  |  |  |  |  |  |  |  |  |  |  |  |  | SST |
|  |  |  |  |  |  |  |  |  |  |  |  |  |  |  |  |  |  | STAT3 |
|  |  |  |  |  |  |  |  |  |  |  |  |  |  |  |  |  |  | STAT4 |
|  |  |  |  |  |  |  |  |  |  |  |  |  |  |  |  |  |  | STS |
|  |  |  |  |  |  |  |  |  |  |  |  |  |  |  |  |  |  | SUGCT |
|  |  |  |  |  |  |  |  |  |  |  |  |  |  |  |  |  |  | SULT1A3 |
|  |  |  |  |  |  |  |  |  |  |  |  |  |  |  |  |  |  | SUMO4 |
|  |  |  |  |  |  |  |  |  |  |  |  |  |  |  |  |  |  | SUSD2 |
|  |  |  |  |  |  |  |  |  |  |  |  |  |  |  |  |  |  | SYK |
|  |  |  |  |  |  |  |  |  |  |  |  |  |  |  |  |  |  | SYP |
|  |  |  |  |  |  |  |  |  |  |  |  |  |  |  |  |  |  | TAC1 |
|  |  |  |  |  |  |  |  |  |  |  |  |  |  |  |  |  |  | TAGLN |
|  |  |  |  |  |  |  |  |  |  |  |  |  |  |  |  |  |  | TBC1D22B |
|  |  |  |  |  |  |  |  |  |  |  |  |  |  |  |  |  |  | TF |
|  |  |  |  |  |  |  |  |  |  |  |  |  |  |  |  |  |  | TFRC |
|  |  |  |  |  |  |  |  |  |  |  |  |  |  |  |  |  |  | TGFA |
|  |  |  |  |  |  |  |  |  |  |  |  |  |  |  |  |  |  | TGFB1 |
|  |  |  |  |  |  |  |  |  |  |  |  |  |  |  |  |  |  | TGFB1I1 |
|  |  |  |  |  |  |  |  |  |  |  |  |  |  |  |  |  |  | TGFB2 |
|  |  |  |  |  |  |  |  |  |  |  |  |  |  |  |  |  |  | TGFB3 |
|  |  |  |  |  |  |  |  |  |  |  |  |  |  |  |  |  |  | TGFBR2 |
|  |  |  |  |  |  |  |  |  |  |  |  |  |  |  |  |  |  | TGM4 |
|  |  |  |  |  |  |  |  |  |  |  |  |  |  |  |  |  |  | TH |
|  |  |  |  |  |  |  |  |  |  |  |  |  |  |  |  |  |  | THBD |
|  |  |  |  |  |  |  |  |  |  |  |  |  |  |  |  |  |  | THRB |
|  |  |  |  |  |  |  |  |  |  |  |  |  |  |  |  |  |  | TIA1 |
|  |  |  |  |  |  |  |  |  |  |  |  |  |  |  |  |  |  | TIMP1 |
|  |  |  |  |  |  |  |  |  |  |  |  |  |  |  |  |  |  | TIMP2 |
|  |  |  |  |  |  |  |  |  |  |  |  |  |  |  |  |  |  | TKT |
|  |  |  |  |  |  |  |  |  |  |  |  |  |  |  |  |  |  | TLR1 |
|  |  |  |  |  |  |  |  |  |  |  |  |  |  |  |  |  |  | TLR2 |
|  |  |  |  |  |  |  |  |  |  |  |  |  |  |  |  |  |  | TLR3 |
|  |  |  |  |  |  |  |  |  |  |  |  |  |  |  |  |  |  | TLR4 |
|  |  |  |  |  |  |  |  |  |  |  |  |  |  |  |  |  |  | TLR5 |
|  |  |  |  |  |  |  |  |  |  |  |  |  |  |  |  |  |  | TLR6 |
|  |  |  |  |  |  |  |  |  |  |  |  |  |  |  |  |  |  | TLR7 |
|  |  |  |  |  |  |  |  |  |  |  |  |  |  |  |  |  |  | TLR8 |
|  |  |  |  |  |  |  |  |  |  |  |  |  |  |  |  |  |  | TLR9 |
|  |  |  |  |  |  |  |  |  |  |  |  |  |  |  |  |  |  | TMEM119 |
|  |  |  |  |  |  |  |  |  |  |  |  |  |  |  |  |  |  | TMEM158 |
|  |  |  |  |  |  |  |  |  |  |  |  |  |  |  |  |  |  | TMEM211 |
|  |  |  |  |  |  |  |  |  |  |  |  |  |  |  |  |  |  | TMEM217 |
|  |  |  |  |  |  |  |  |  |  |  |  |  |  |  |  |  |  | TMEM42 |
|  |  |  |  |  |  |  |  |  |  |  |  |  |  |  |  |  |  | TNF |
|  |  |  |  |  |  |  |  |  |  |  |  |  |  |  |  |  |  | TNFAIP3 |
|  |  |  |  |  |  |  |  |  |  |  |  |  |  |  |  |  |  | TNFRSF10A |
|  |  |  |  |  |  |  |  |  |  |  |  |  |  |  |  |  |  | TNFRSF1A |
|  |  |  |  |  |  |  |  |  |  |  |  |  |  |  |  |  |  | TNFRSF1B |
|  |  |  |  |  |  |  |  |  |  |  |  |  |  |  |  |  |  | TNFSF12 |
|  |  |  |  |  |  |  |  |  |  |  |  |  |  |  |  |  |  | TNFSF13B |
|  |  |  |  |  |  |  |  |  |  |  |  |  |  |  |  |  |  | TNIP1 |
|  |  |  |  |  |  |  |  |  |  |  |  |  |  |  |  |  |  | TNKS |
|  |  |  |  |  |  |  |  |  |  |  |  |  |  |  |  |  |  | TNXB |
|  |  |  |  |  |  |  |  |  |  |  |  |  |  |  |  |  |  | TP53 |
|  |  |  |  |  |  |  |  |  |  |  |  |  |  |  |  |  |  | TPMT |
|  |  |  |  |  |  |  |  |  |  |  |  |  |  |  |  |  |  | TRABD |
|  |  |  |  |  |  |  |  |  |  |  |  |  |  |  |  |  |  | TRAF3IP2 |
|  |  |  |  |  |  |  |  |  |  |  |  |  |  |  |  |  |  | TRPV1 |
|  |  |  |  |  |  |  |  |  |  |  |  |  |  |  |  |  |  | TUBGCP6 |
|  |  |  |  |  |  |  |  |  |  |  |  |  |  |  |  |  |  | TYR |
|  |  |  |  |  |  |  |  |  |  |  |  |  |  |  |  |  |  | UBE2E1 |
|  |  |  |  |  |  |  |  |  |  |  |  |  |  |  |  |  |  | UBE2E2 |
|  |  |  |  |  |  |  |  |  |  |  |  |  |  |  |  |  |  | UPB1 |
|  |  |  |  |  |  |  |  |  |  |  |  |  |  |  |  |  |  | UROD |
|  |  |  |  |  |  |  |  |  |  |  |  |  |  |  |  |  |  | VAX2 |
|  |  |  |  |  |  |  |  |  |  |  |  |  |  |  |  |  |  | VCAM1 |
|  |  |  |  |  |  |  |  |  |  |  |  |  |  |  |  |  |  | VCL |
|  |  |  |  |  |  |  |  |  |  |  |  |  |  |  |  |  |  | VDR |
|  |  |  |  |  |  |  |  |  |  |  |  |  |  |  |  |  |  | VEGFA |
|  |  |  |  |  |  |  |  |  |  |  |  |  |  |  |  |  |  | VIM |
|  |  |  |  |  |  |  |  |  |  |  |  |  |  |  |  |  |  | WNT10A |
|  |  |  |  |  |  |  |  |  |  |  |  |  |  |  |  |  |  | WNT5A |
|  |  |  |  |  |  |  |  |  |  |  |  |  |  |  |  |  |  | WSCD2 |
|  |  |  |  |  |  |  |  |  |  |  |  |  |  |  |  |  |  | WT1 |
|  |  |  |  |  |  |  |  |  |  |  |  |  |  |  |  |  |  | XDH |
|  |  |  |  |  |  |  |  |  |  |  |  |  |  |  |  |  |  | YAP1 |
|  |  |  |  |  |  |  |  |  |  |  |  |  |  |  |  |  |  | ZBED4 |
|  |  |  |  |  |  |  |  |  |  |  |  |  |  |  |  |  |  | ZDHHC3 |
|  |  |  |  |  |  |  |  |  |  |  |  |  |  |  |  |  |  | ZFAND3 |
|  |  |  |  |  |  |  |  |  |  |  |  |  |  |  |  |  |  | ZNF224 |


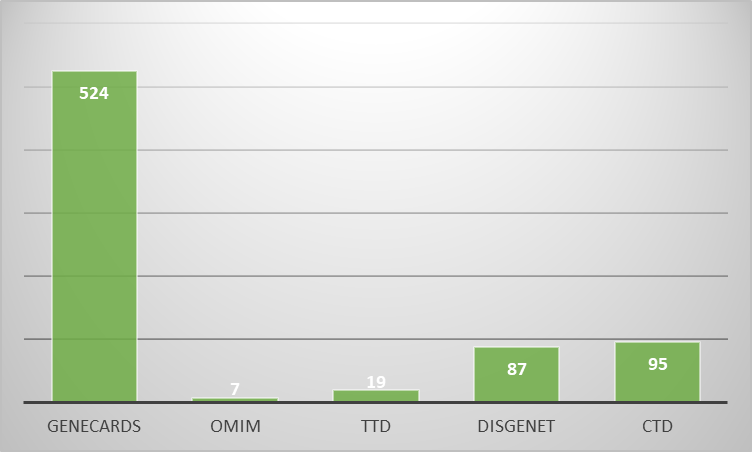

Supplement: Supplementary Materials — Supplementary file 1, Tables S1 and S2: the basic information of all active compounds and related targets. Supplementary file 2, Table S3; and Supplementary file 3, Figure S1: the detailed information of the potential target genes of acne vulgaris. Supplementary file 4, Tables 4, S5, and S6: the detailed information of GO enrichment analysis for BP, CC, and MF. Supplementary file 5, Table S7: the detailed information of screened KEGG pathways. [file 6944792.f1.zip › 6944792.f1/Supplementary file2, Table S3, The detailed information of the potential target genes of acne vulgaris.docx]
