# Supplementary material for: Network Pharmacology-Based Investigation on the Mechanism of the JinGuanLan Formula in Treating Acne Vulgaris
Source: Evid Based Complement Alternat Med. 2022 Jul 13;2022:6944792. doi: 10.1155/2022/6944792 (PMC9300327; doi:10.1155/2022/6944792)

**Supplementary file3:figure S1:The detailed information of the potential target genes of acne vulgaris**


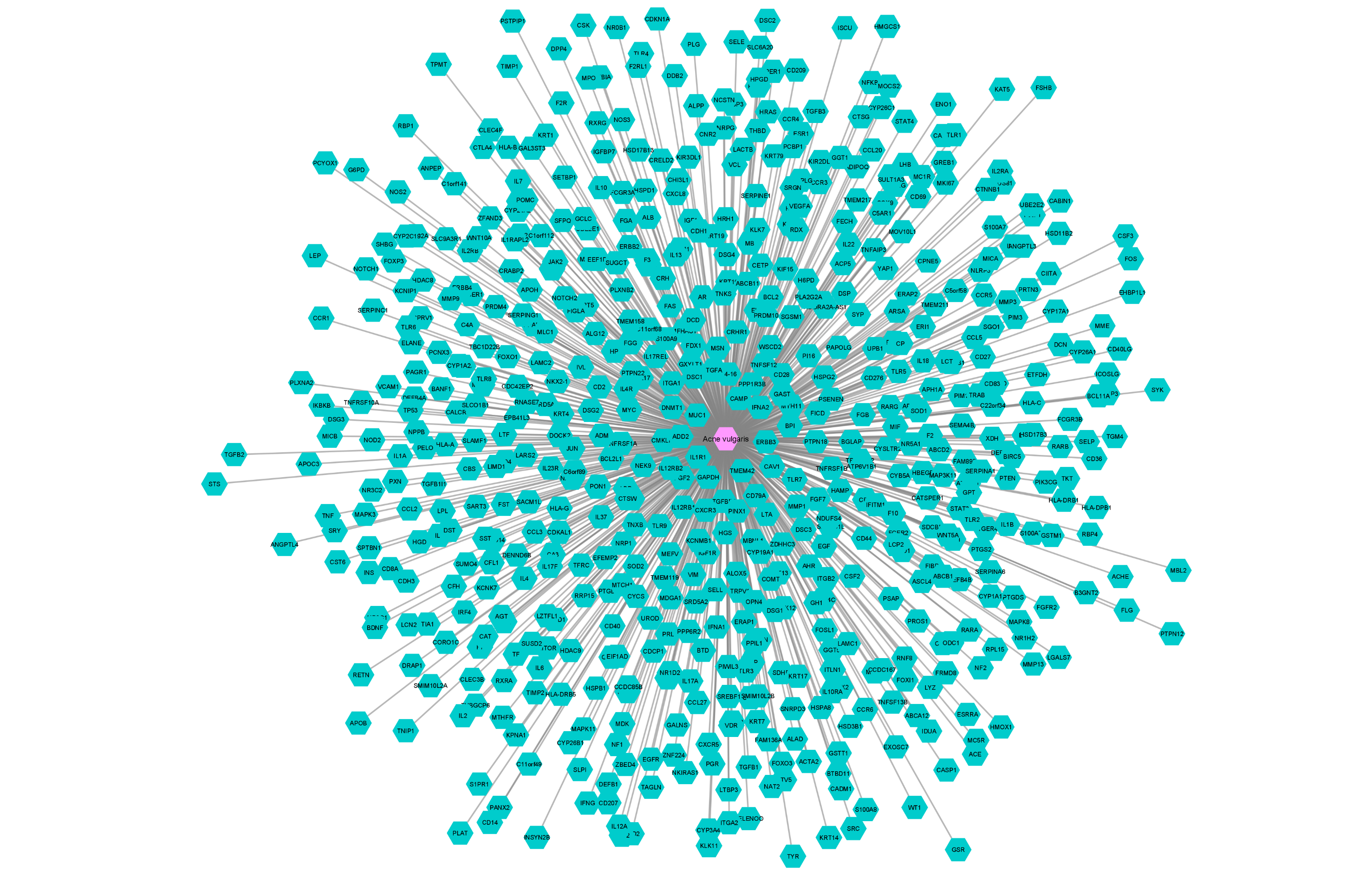

Supplement: Supplementary Materials — Supplementary file 1, Tables S1 and S2: the basic information of all active compounds and related targets. Supplementary file 2, Table S3; and Supplementary file 3, Figure S1: the detailed information of the potential target genes of acne vulgaris. Supplementary file 4, Tables 4, S5, and S6: the detailed information of GO enrichment analysis for BP, CC, and MF. Supplementary file 5, Table S7: the detailed information of screened KEGG pathways. [file 6944792.f1.zip › 6944792.f1/Supplementary file3, Figure S1 The detailed information of the potential target genes of acne vulgaris.docx]
