# Supplementary material for: Network Pharmacology-Based Investigation on the Mechanism of the JinGuanLan Formula in Treating Acne Vulgaris
Source: Evid Based Complement Alternat Med. 2022 Jul 13;2022:6944792. doi: 10.1155/2022/6944792 (PMC9300327; doi:10.1155/2022/6944792)
Supplement: Supplementary Materials — Supplementary file 1, Tables S1 and S2: the basic information of all active compounds and related targets. Supplementary file 2, Table S3; and Supplementary file 3, Figure S1: the detailed information of the potential target genes of acne vulgaris. Supplementary file 4, Tables 4, S5, and S6: the detailed information of GO enrichment analysis for BP, CC, and MF. Supplementary file 5, Table S7: the detailed information of screened KEGG pathways. [file 6944792.f1.zip › 6944792.f1/Supplementary file4, Tables S4, S5 and, S6 The detailed information of GO enrichment analysis for BP, CC, and MF. (1).docx]

| **Supplementary file4: Table S4. The detailed information of GO enrichment analysis of biological process (BP)** | | | | | | | |  |
| --- | --- | --- | --- | --- | --- | --- | --- | --- |
| **ID** | **Description** | **GeneRatio** | **Bg Ratio** | **p-value** | **P .adjust** | **q value** | **geneID** | **Count** |
| GO:0032496 | response to lipopolysaccharide | 29/97 | 334/18866 | 6.85E-28 | 2.55E-24 | 1.09E-24 | AKT1/APOB/CASP3/CCL2/CXCL8/CYP1A1/CYP1A2/FOS/GSTP1/ICAM1/IL10/IL1A/IL1B/IL6/MAPK1/MAPK3/MAPK8/MPO/NFKBIA/NOS2/NOS3/PPARD/PTGS2/SELE/SERPINE1/SLPI/TGFB1/THBD/TNF | 29 |
| GO:0002237 | response to molecule of bacterial origin | 29/97 | 356/18866 | 4.36E-27 | 7.07E-24 | 3.01E-24 | AKT1/APOB/CASP3/CCL2/CXCL8/CYP1A1/CYP1A2/FOS/GSTP1/ICAM1/IL10/IL1A/IL1B/IL6/MAPK1/MAPK3/MAPK8/MPO/NFKBIA/NOS2/NOS3/PPARD/PTGS2/SELE/SERPINE1/SLPI/TGFB1/THBD/TNF | 29 |
| GO:0062197 | cellular response to chemical stress | 29/97 | 360/18866 | 6.02E-27 | 7.07E-24 | 3.01E-24 | AKT1/ALOX5/BCL2/CASP3/CAT/CAV1/CTNNB1/EGFR/FOS/GSR/GSTP1/HMOX1/HSPB1/IL10/IL6/JUN/MAPK1/MAPK3/MAPK8/MMP2/MMP3/MMP9/MPO/NOS3/PPARG/PTGS2/SOD1/TNF/TP53 | 29 |
| GO:0072593 | reactive oxygen species metabolic process | 27/97 | 288/18866 | 7.59E-27 | 7.07E-24 | 3.01E-24 | AKT1/ALOX5/BCL2/CAT/CAV1/CDKN1A/CRP/CYP1A1/CYP1A2/EGFR/F2/GSTP1/ICAM1/IFNG/IL10/IL1B/MMP3/MPO/NOS2/NOS3/PTGS2/SOD1/STAT3/TGFB1/TNF/TP53/XDH | 27 |
| GO:0033002 | muscle cell proliferation | 24/97 | 244/18866 | 2.30E-24 | 1.71E-21 | 7.30E-22 | ADIPOQ/AKT1/CDKN1A/CTNNB1/EGFR/GSTP1/HMOX1/IFNG/IGFBP3/IL10/IL6/JUN/MAPK1/MMP2/MMP9/NOS3/PIM1/PPARD/PPARG/PTEN/PTGS2/RXRA/STAT3/TNF | 24 |
| GO:0050727 | regulation of inflammatory response | 28/97 | 425/18866 | 1.38E-23 | 7.80E-21 | 3.32E-21 | ADIPOQ/ALOX5/CYP19A1/EGFR/ESR1/F2/GSTP1/IFNG/IL10/IL1B/IL2/IL4/IL6/MMP3/MMP9/NFKBIA/NOS2/PIK3CG/PPARA/PPARD/PPARG/PTGER3/PTGS2/SELE/SERPINE1/SOD1/STAT3/TNF | 28 |
| GO:0031667 | response to nutrient levels | 29/97 | 473/18866 | 1.47E-23 | 7.80E-21 | 3.32E-21 | ADIPOQ/AKT1/ALB/BCL2/CAT/CDKN1A/CYP1A1/EGFR/F7/GSTP1/HMOX1/ICAM1/JUN/MAPK1/MAPK3/MAPK8/MPO/PIM1/PON1/PPARA/PPARD/PPARG/PTEN/PTGS2/SOD1/SPP1/SREBF1/TP53/TYR | 29 |
| GO:0034599 | cellular response to oxidative stress | 25/97 | 310/18866 | 3.27E-23 | 1.52E-20 | 6.48E-21 | AKT1/ALOX5/BCL2/CAT/CTNNB1/EGFR/FOS/GSR/GSTP1/HMOX1/HSPB1/IL10/IL6/JUN/MAPK1/MAPK3/MAPK8/MMP2/MMP3/MMP9/MPO/NOS3/SOD1/TNF/TP53 | 25 |
| GO:0006979 | response to oxidative stress | 28/97 | 458/18866 | 1.06E-22 | 4.39E-20 | 1.87E-20 | ADIPOQ/AKT1/ALOX5/BCL2/CASP3/CAT/CTNNB1/EGFR/FOS/GSR/GSTP1/HMOX1/HSPB1/IL10/IL6/JUN/MAPK1/MAPK3/MAPK8/MMP2/MMP3/MMP9/MPO/NOS3/PTGS2/SOD1/TNF/TP53 | 28 |
| GO:0048545 | response to steroid hormone | 25/97 | 346/18866 | 4.95E-22 | 1.84E-19 | 7.86E-20 | ADIPOQ/AR/BCL2/CASP3/CAV1/CDKN1A/EGFR/ESR1/FOS/GSTP1/HSD3B1/ICAM1/IL10/IL6/NR3C1/NR3C2/PGR/PPARA/PPARD/PTGS2/RXRA/SPP1/SREBF1/TGFB1/TNF | 25 |
| GO:2000377 | regulation of reactive oxygen species metabolic process | 21/97 | 200/18866 | 5.80E-22 | 1.96E-19 | 8.36E-20 | AKT1/ALOX5/BCL2/CAV1/CDKN1A/CRP/EGFR/F2/GSTP1/ICAM1/IFNG/IL10/IL1B/MMP3/PTGS2/SOD1/STAT3/TGFB1/TNF/TP53/XDH | 21 |
| GO:0048660 | regulation of smooth muscle cell proliferation | 20/97 | 173/18866 | 8.53E-22 | 2.65E-19 | 1.13E-19 | ADIPOQ/AKT1/CDKN1A/CTNNB1/EGFR/GSTP1/HMOX1/IFNG/IGFBP3/IL10/IL6/JUN/MMP2/MMP9/NOS3/PPARD/PPARG/PTEN/PTGS2/TNF | 20 |
| GO:0048659 | smooth muscle cell proliferation | 20/97 | 175/18866 | 1.08E-21 | 3.09E-19 | 1.32E-19 | ADIPOQ/AKT1/CDKN1A/CTNNB1/EGFR/GSTP1/HMOX1/IFNG/IGFBP3/IL10/IL6/JUN/MMP2/MMP9/NOS3/PPARD/PPARG/PTEN/PTGS2/TNF | 20 |
| GO:0000302 | response to reactive oxygen species | 21/97 | 235/18866 | 1.77E-20 | 4.70E-18 | 2.00E-18 | AKT1/BCL2/CASP3/CAT/EGFR/FOS/GSTP1/HMOX1/IL10/IL6/JUN/MAPK1/MAPK3/MAPK8/MMP2/MMP3/MMP9/MPO/NOS3/SOD1/TNF | 21 |
| GO:0050673 | epithelial cell proliferation | 26/97 | 453/18866 | 2.23E-20 | 5.53E-18 | 2.36E-18 | AKT1/ALOX5/AR/CAV1/CCL2/CTNNB1/EGFR/ERBB2/ESR1/F3/HMOX1/IGFBP3/IL10/IL6/JUN/MAPK1/MYC/PGR/PPARD/PPARG/PTEN/STAT3/TGFB1/TNF/VEGFA/XDH | 26 |
| GO:0048732 | gland development | 25/97 | 443/18866 | 2.02E-19 | 4.71E-17 | 2.01E-17 | AKT1/AR/BCL2/CAV1/CTNNB1/CYP19A1/CYP1A1/EGF/EGFR/ESR1/HMOX1/IL10/IL6/JUN/MAPK1/MAPK3/PGR/PTEN/RXRA/SOD1/TGFB1/TNF/TYR/VEGFA/XDH | 25 |
| GO:0042493 | response to drug | 24/97 | 397/18866 | 2.42E-19 | 5.31E-17 | 2.26E-17 | ADIPOQ/BCL2/CASP3/CAT/CDKN1A/CTNNB1/CYP1A1/CYP1A2/CYP3A4/EGFR/F7/FOS/ICAM1/IL10/IL1B/MYC/NOS2/PPARG/PTEN/PTGS2/SOD1/SREBF1/STAT3/TP53 | 24 |
| GO:0097191 | extrinsic apoptotic signaling pathway | 20/97 | 230/18866 | 2.72E-19 | 5.62E-17 | 2.40E-17 | AKT1/AR/BCL2/BCL2L1/CASP3/CAV1/ERBB3/GSTP1/HMOX1/ICAM1/IFNG/IL1A/IL1B/IL2/IL4/NOS3/PTEN/SERPINE1/TGFB1/TNF | 20 |
| GO:0051090 | regulation of DNA-binding transcription factor activity | 25/97 | 455/18866 | 3.84E-19 | 7.53E-17 | 3.21E-17 | AKT1/AR/CAT/CAV1/CD40LG/CTNNB1/ESR1/FOS/HMOX1/ICAM1/IKBKB/IL10/IL1B/IL6/JUN/MAPK1/MAPK3/MAPK8/NFKBIA/PIM1/PPARG/PTEN/STAT3/TNF/VEGFA | 25 |
| GO:0071222 | cellular response to lipopolysaccharide | 19/97 | 208/18866 | 9.13E-19 | 1.70E-16 | 7.25E-17 | AKT1/CCL2/CXCL8/GSTP1/ICAM1/IL10/IL1A/IL1B/IL6/MAPK1/MAPK3/MAPK8/NFKBIA/NOS2/NOS3/PPARD/SERPINE1/TGFB1/TNF | 19 |
| GO:0071216 | cellular response to biotic stimulus | 20/97 | 246/18866 | 1.04E-18 | 1.84E-16 | 7.83E-17 | AKT1/CCL2/CXCL8/GSTP1/ICAM1/IL10/IL1A/IL1B/IL6/MAPK1/MAPK3/MAPK8/NFKBIA/NOS2/NOS3/PPARD/SERPINE1/TGFB1/TNF/TP53 | 20 |
| GO:0071219 | cellular response to molecule of bacterial origin | 19/97 | 222/18866 | 3.15E-18 | 5.32E-16 | 2.27E-16 | AKT1/CCL2/CXCL8/GSTP1/ICAM1/IL10/IL1A/IL1B/IL6/MAPK1/MAPK3/MAPK8/NFKBIA/NOS2/NOS3/PPARD/SERPINE1/TGFB1/TNF | 19 |
| GO:0070482 | response to oxygen levels | 23/97 | 396/18866 | 3.67E-18 | 5.94E-16 | 2.53E-16 | ADIPOQ/AKT1/BCL2/CASP3/CAT/CAV1/CDKN1A/CYP1A1/DPP4/F7/HMOX1/ICAM1/MMP2/MYC/NOS2/PLAT/PPARA/PPARD/PPARG/PTEN/PTGS2/TP53/VEGFA | 23 |
| GO:1903409 | reactive oxygen species biosynthetic process | 16/97 | 128/18866 | 3.84E-18 | 5.96E-16 | 2.54E-16 | AKT1/ALOX5/CAV1/CYP1A1/CYP1A2/ICAM1/IFNG/IL10/IL1B/MPO/NOS2/NOS3/PTGS2/SOD1/STAT3/TNF | 16 |
| GO:0044706 | multi-multicellular organism process | 19/97 | 226/18866 | 4.41E-18 | 6.57E-16 | 2.80E-16 | AKT1/AR/BCL2/CYP1A1/ESR1/FOS/IL1B/MAPK1/MAPK3/MMP2/MMP9/PGR/PPARD/PTGS2/RXRA/SOD1/SPP1/THBD/VEGFA | 19 |
| GO:2000379 | positive regulation of reactive oxygen species metabolic process | 15/97 | 106/18866 | 6.66E-18 | 9.54E-16 | 4.07E-16 | AKT1/CDKN1A/CRP/EGFR/F2/GSTP1/ICAM1/IFNG/IL1B/PTGS2/SOD1/TGFB1/TNF/TP53/XDH | 15 |
| GO:0007565 | female pregnancy | 18/97 | 196/18866 | 7.39E-18 | 1.02E-15 | 4.35E-16 | AKT1/AR/BCL2/ESR1/FOS/IL1B/MAPK1/MAPK3/MMP2/MMP9/PGR/PPARD/PTGS2/RXRA/SOD1/SPP1/THBD/VEGFA | 18 |
| GO:0002526 | acute inflammatory response | 15/97 | 111/18866 | 1.37E-17 | 1.82E-15 | 7.76E-16 | CRP/F2/F3/GSTP1/ICAM1/IL1A/IL1B/IL4/IL6/PIK3CG/PPARG/PTGER3/PTGS2/STAT3/TNF | 15 |
| GO:0071496 | cellular response to external stimulus | 21/97 | 326/18866 | 1.56E-17 | 2.00E-15 | 8.53E-16 | AKT1/ALB/BCL2/CDKN1A/EGFR/FOS/GSTP1/HMOX1/ICAM1/IL1B/JUN/MAPK1/MAPK3/MAPK8/PIM1/PPARA/PPARG/PTGS2/SREBF1/TP53/VCAM1 | 21 |
| GO:1902893 | regulation of pri-miRNA transcription by RNA polymerase II | Dec-97 | 51/18866 | 2.27E-17 | 2.82E-15 | 1.20E-15 | FOS/IL10/JUN/NR3C1/PPARA/PPARD/PPARG/SREBF1/STAT3/TGFB1/TNF/TP53 | 12 |
| GO:0009636 | response to toxic substance | 19/97 | 250/18866 | 2.95E-17 | 3.54E-15 | 1.51E-15 | AHR/ALB/BCL2/CAT/CDKN1A/CYP1A1/FOS/GSR/GSTM1/GSTP1/MAPK1/MAPK3/MPO/NOS3/PIM1/PON1/PTGS2/SOD1/TNF | 19 |
| GO:0022407 | regulation of cell-cell adhesion | 23/97 | 439/18866 | 3.55E-17 | 4.14E-15 | 1.76E-15 | ADIPOQ/AKT1/ALOX5/CASP3/CAV1/CCL2/CD40LG/DPP4/ERBB2/ICAM1/IFNG/IL10/IL1A/IL1B/IL2/IL4/IL6/PPARA/SELE/TGFB1/TNF/VCAM1/VEGFA | 23 |
| GO:0061614 | pri-miRNA transcription by RNA polymerase II | Dec-97 | 53/18866 | 3.79E-17 | 4.28E-15 | 1.82E-15 | FOS/IL10/JUN/NR3C1/PPARA/PPARD/PPARG/SREBF1/STAT3/TGFB1/TNF/TP53 | 12 |
| GO:0071276 | cellular response to cadmium ion | Nov-97 | 38/18866 | 3.97E-17 | 4.35E-15 | 1.85E-15 | AKT1/CYP1A2/EGFR/FOS/HMOX1/JUN/MAPK1/MAPK3/MAPK8/MMP9/SOD1 | 11 |
| GO:0048608 | reproductive structure development | 23/97 | 443/18866 | 4.34E-17 | 4.61E-15 | 1.97E-15 | AKT1/AR/BCL2/BCL2L1/CASP3/CTNNB1/CYP19A1/EGFR/ESR1/ICAM1/IL10/MAPK1/MAPK3/NOS3/PGR/PPARD/PPARG/PTEN/PTGS2/RXRA/SOD1/SPP1/VEGFA | 23 |
| GO:0050678 | regulation of epithelial cell proliferation | 22/97 | 395/18866 | 5.27E-17 | 5.31E-15 | 2.26E-15 | AKT1/ALOX5/AR/CAV1/CCL2/CTNNB1/EGFR/ERBB2/F3/HMOX1/IL10/JUN/MYC/PGR/PPARD/PPARG/PTEN/STAT3/TGFB1/TNF/VEGFA/XDH | 22 |
| GO:0061458 | reproductive system development | 23/97 | 447/18866 | 5.28E-17 | 5.31E-15 | 2.26E-15 | AKT1/AR/BCL2/BCL2L1/CASP3/CTNNB1/CYP19A1/EGFR/ESR1/ICAM1/IL10/MAPK1/MAPK3/NOS3/PGR/PPARD/PPARG/PTEN/PTGS2/RXRA/SOD1/SPP1/VEGFA | 23 |
| GO:0001666 | response to hypoxia | 21/97 | 359/18866 | 1.10E-16 | 1.08E-14 | 4.61E-15 | ADIPOQ/AKT1/BCL2/CASP3/CAT/CAV1/CYP1A1/DPP4/F7/HMOX1/ICAM1/MMP2/MYC/NOS2/PLAT/PPARA/PPARD/PTEN/PTGS2/TP53/VEGFA | 21 |
| GO:0070661 | leukocyte proliferation | 20/97 | 313/18866 | 1.17E-16 | 1.12E-14 | 4.76E-15 | AHR/BCL2/CASP3/CD40LG/CDKN1A/CRP/CTNNB1/ERBB2/GSTP1/IL10/IL1A/IL1B/IL2/IL4/IL6/MAPK1/MAPK3/PIK3CG/TP53/VCAM1 | 20 |
| GO:0010001 | glial cell differentiation | 18/97 | 230/18866 | 1.30E-16 | 1.21E-14 | 5.15E-15 | AKT1/CTNNB1/EGFR/ERBB2/ERBB3/F2/GSTP1/IFNG/IL1B/IL6/MAPK1/MAPK3/PPARG/PTEN/SOD1/STAT3/TGFB1/TNF | 18 |
| GO:2001233 | regulation of apoptotic signaling pathway | 22/97 | 413/18866 | 1.34E-16 | 1.22E-14 | 5.21E-15 | AKT1/AR/BCL2/BCL2L1/CAV1/CTNNB1/GSTP1/HMOX1/HSPB1/ICAM1/IL1A/IL1B/IL4/MAPK8/MMP9/NOS3/PTEN/PTGS2/SERPINE1/SOD1/TNF/TP53 | 22 |
| GO:0097305 | response to alcohol | 18/97 | 234/18866 | 1.76E-16 | 1.56E-14 | 6.66E-15 | ADIPOQ/AHR/AKT1/BCL2L1/CAT/CDKN1A/CTNNB1/F7/FOS/GSTP1/HSD3B1/ICAM1/IL2/PPARG/PTEN/SOD1/STAT3/TGFB1 | 18 |
| GO:0036293 | response to decreased oxygen levels | 21/97 | 371/18866 | 2.14E-16 | 1.85E-14 | 7.90E-15 | ADIPOQ/AKT1/BCL2/CASP3/CAT/CAV1/CYP1A1/DPP4/F7/HMOX1/ICAM1/MMP2/MYC/NOS2/PLAT/PPARA/PPARD/PTEN/PTGS2/TP53/VEGFA | 21 |
| GO:0070663 | regulation of leukocyte proliferation | 18/97 | 240/18866 | 2.76E-16 | 2.34E-14 | 9.96E-15 | AHR/BCL2/CASP3/CD40LG/CDKN1A/CRP/CTNNB1/ERBB2/GSTP1/IL10/IL1A/IL1B/IL2/IL4/IL6/MAPK1/MAPK3/VCAM1 | 18 |
| GO:1903037 | regulation of leukocyte cell-cell adhesion | 20/97 | 329/18866 | 3.07E-16 | 2.54E-14 | 1.08E-14 | AKT1/ALOX5/CASP3/CAV1/CCL2/CD40LG/DPP4/ERBB2/ICAM1/IFNG/IL10/IL1A/IL1B/IL2/IL4/IL6/PPARA/SELE/TNF/VCAM1 | 20 |
| GO:0034614 | cellular response to reactive oxygen species | 16/97 | 170/18866 | 3.89E-16 | 3.15E-14 | 1.34E-14 | AKT1/EGFR/FOS/IL10/IL6/JUN/MAPK1/MAPK3/MAPK8/MMP2/MMP3/MMP9/MPO/NOS3/SOD1/TNF | 16 |
| GO:1905952 | regulation of lipid localization | 16/97 | 171/18866 | 4.28E-16 | 3.39E-14 | 1.44E-14 | ADIPOQ/AKT1/APOB/CAV1/CRP/CYP19A1/EGF/IL1B/IL6/NFKBIA/PON1/PPARA/PPARD/PPARG/SPP1/TNF | 16 |
| GO:0046686 | response to cadmium ion | Dec-97 | 64/18866 | 4.46E-16 | 3.46E-14 | 1.47E-14 | AKT1/CAT/CYP1A2/EGFR/FOS/HMOX1/JUN/MAPK1/MAPK3/MAPK8/MMP9/SOD1 | 12 |
| GO:0032355 | response to estradiol | 15/97 | 141/18866 | 5.44E-16 | 4.14E-14 | 1.76E-14 | APOB/CASP3/CAT/CTNNB1/CYP19A1/CYP1A2/EGFR/ESR1/F7/GSTP1/IL10/PTEN/PTGS2/STAT3/TGFB1 | 15 |
| GO:0062012 | regulation of small molecule metabolic process | 22/97 | 456/18866 | 1.06E-15 | 7.91E-14 | 3.37E-14 | ADIPOQ/AKT1/APOB/CAV1/EGF/IFNG/IGFBP3/IL1B/IL4/NOS2/NOS3/ODC1/PPARA/PPARD/PPARG/PTGS2/SOD1/SREBF1/STAT3/TGFB1/TNF/TP53 | 22 |
| GO:0042063 | gliogenesis | 19/97 | 307/18866 | 1.33E-15 | 9.69E-14 | 4.13E-14 | AKT1/CCL2/CTNNB1/EGFR/ERBB2/ERBB3/F2/GSTP1/IFNG/IL1B/IL6/MAPK1/MAPK3/PPARG/PTEN/SOD1/STAT3/TGFB1/TNF | 19 |
| GO:0062013 | positive regulation of small molecule metabolic process | 15/97 | 154/18866 | 2.07E-15 | 1.48E-13 | 6.33E-14 | ADIPOQ/AKT1/EGF/IFNG/IL1B/IL4/NOS2/NOS3/PPARA/PPARD/PPARG/PTGS2/SREBF1/STAT3/TNF | 15 |
| GO:0007159 | leukocyte cell-cell adhesion | 20/97 | 364/18866 | 2.14E-15 | 1.50E-13 | 6.41E-14 | AKT1/ALOX5/CASP3/CAV1/CCL2/CD40LG/DPP4/ERBB2/ICAM1/IFNG/IL10/IL1A/IL1B/IL2/IL4/IL6/PPARA/SELE/TNF/VCAM1 | 20 |
| GO:0010038 | response to metal ion | 20/97 | 366/18866 | 2.38E-15 | 1.64E-13 | 6.98E-14 | AKT1/BCL2/CASP3/CAT/CAV1/CYP1A1/CYP1A2/EGFR/FOS/HMOX1/ICAM1/IL1A/JUN/MAPK1/MAPK3/MAPK8/MMP9/PTEN/PTGS2/SOD1 | 20 |
| GO:0007568 | aging | 19/97 | 319/18866 | 2.68E-15 | 1.81E-13 | 7.74E-14 | AKT1/BCL2/CAT/CDKN1A/CYP1A1/FOS/ICAM1/IL10/JUN/MAPK1/MAPK3/MPO/PTEN/PTGS2/SERPINE1/SOD1/SREBF1/STAT3/TP53 | 19 |
| GO:2001234 | negative regulation of apoptotic signaling pathway | 17/97 | 233/18866 | 3.17E-15 | 2.11E-13 | 8.98E-14 | AKT1/AR/BCL2/BCL2L1/CTNNB1/GSTP1/HMOX1/HSPB1/ICAM1/IL1A/IL1B/IL4/MMP9/NOS3/PTGS2/SERPINE1/TNF | 17 |
| GO:0042110 | T cell activation | 22/97 | 483/18866 | 3.49E-15 | 2.28E-13 | 9.72E-14 | AKT1/BCL2/CASP3/CAV1/CCL2/CD40LG/CTNNB1/DPP4/ERBB2/ICAM1/IFNG/IL10/IL1A/IL1B/IL2/IL4/IL6/PIK3CG/SOD1/STAT3/TP53/VCAM1 | 22 |
| GO:2001236 | regulation of extrinsic apoptotic signaling pathway | 15/97 | 162/18866 | 4.44E-15 | 2.85E-13 | 1.22E-13 | AKT1/AR/BCL2/BCL2L1/CAV1/GSTP1/HMOX1/ICAM1/IL1A/IL1B/IL4/NOS3/PTEN/SERPINE1/TNF | 15 |
| GO:0032102 | negative regulation of response to external stimulus | 21/97 | 433/18866 | 4.69E-15 | 2.96E-13 | 1.26E-13 | ADIPOQ/ALOX5/CCL2/CYP19A1/DPP4/F2/GSTP1/IL10/IL2/IL4/NOS3/PLAT/PPARA/PPARD/PPARG/PTEN/SERPINE1/SOD1/SPP1/THBD/TNF | 21 |
| GO:1901654 | response to ketone | 16/97 | 200/18866 | 5.19E-15 | 3.22E-13 | 1.37E-13 | AHR/AKT1/AR/BCL2L1/CAV1/CDKN1A/EGFR/F7/FOS/HSD3B1/ICAM1/NR3C1/PPARG/SPP1/SREBF1/TGFB1 | 16 |
| GO:1902895 | positive regulation of pri-miRNA transcription by RNA polymerase II | Oct-97 | 40/18866 | 5.98E-15 | 3.65E-13 | 1.56E-13 | FOS/IL10/JUN/NR3C1/PPARG/SREBF1/STAT3/TGFB1/TNF/TP53 | 10 |
| GO:2001237 | negative regulation of extrinsic apoptotic signaling pathway | 13/97 | 107/18866 | 9.32E-15 | 5.59E-13 | 2.39E-13 | AKT1/AR/BCL2/BCL2L1/GSTP1/HMOX1/ICAM1/IL1A/IL1B/IL4/NOS3/SERPINE1/TNF | 13 |
| GO:0031668 | cellular response to extracellular stimulus | 17/97 | 253/18866 | 1.24E-14 | 7.35E-13 | 3.13E-13 | ALB/BCL2/CDKN1A/FOS/GSTP1/HMOX1/ICAM1/JUN/MAPK1/MAPK3/MAPK8/PIM1/PPARA/PPARG/SREBF1/TP53/VCAM1 | 17 |
| GO:0002685 | regulation of leukocyte migration | 16/97 | 212/18866 | 1.30E-14 | 7.57E-13 | 3.23E-13 | AKT1/CCL2/CXCL8/CYP19A1/DPP4/F7/HMOX1/ICAM1/IL4/IL6/MAPK1/MAPK3/SELE/SERPINE1/TNF/VEGFA | 16 |
| GO:0051384 | response to glucocorticoid | 14/97 | 147/18866 | 2.61E-14 | 1.49E-12 | 6.36E-13 | ADIPOQ/BCL2/CASP3/CDKN1A/EGFR/FOS/GSTP1/HSD3B1/ICAM1/IL10/IL6/NR3C1/PTGS2/TNF | 14 |
| GO:0051098 | regulation of binding | 19/97 | 367/18866 | 3.41E-14 | 1.93E-12 | 8.21E-13 | ADIPOQ/AKT1/CAV1/CDKN1A/CTNNB1/EGF/HMOX1/IFNG/IL10/JUN/MAPK3/MAPK8/MMP9/NFKBIA/PON1/PPARA/PPARG/SLPI/TGFB1 | 19 |
| GO:0051091 | positive regulation of DNA-binding transcription factor activity | 17/97 | 270/18866 | 3.63E-14 | 2.02E-12 | 8.61E-13 | AKT1/AR/CAT/CAV1/CD40LG/CTNNB1/ESR1/ICAM1/IKBKB/IL10/IL1B/IL6/PPARG/PTEN/STAT3/TNF/VEGFA | 17 |
| GO:0048662 | negative regulation of smooth muscle cell proliferation | Nov-97 | 69/18866 | 5.28E-14 | 2.89E-12 | 1.23E-12 | ADIPOQ/CDKN1A/GSTP1/HMOX1/IFNG/IGFBP3/IL10/NOS3/PPARD/PPARG/PTEN | 11 |
| GO:0022409 | positive regulation of cell-cell adhesion | 17/97 | 279/18866 | 6.22E-14 | 3.36E-12 | 1.43E-12 | AKT1/ALOX5/CAV1/CCL2/CD40LG/DPP4/ICAM1/IFNG/IL10/IL1A/IL1B/IL2/IL4/IL6/SELE/TNF/VCAM1 | 17 |
| GO:1903039 | positive regulation of leukocyte cell-cell adhesion | 16/97 | 235/18866 | 6.52E-14 | 3.47E-12 | 1.48E-12 | AKT1/ALOX5/CAV1/CCL2/CD40LG/DPP4/ICAM1/IFNG/IL1A/IL1B/IL2/IL4/IL6/SELE/TNF/VCAM1 | 16 |
| GO:0008202 | steroid metabolic process | 18/97 | 332/18866 | 7.89E-14 | 4.14E-12 | 1.76E-12 | APOB/CAT/CYP19A1/CYP1A1/CYP1A2/CYP3A4/ESR1/HSD3B1/IFNG/IL1B/IL4/PON1/PPARD/RXRA/SOD1/SPP1/SREBF1/TNF | 18 |
| GO:0032943 | mononuclear cell proliferation | 17/97 | 286/18866 | 9.33E-14 | 4.83E-12 | 2.06E-12 | AHR/BCL2/CASP3/CD40LG/CDKN1A/CRP/CTNNB1/ERBB2/IL10/IL1A/IL1B/IL2/IL4/IL6/PIK3CG/TP53/VCAM1 | 17 |
| GO:0031960 | response to corticosteroid | 14/97 | 164/18866 | 1.20E-13 | 6.14E-12 | 2.62E-12 | ADIPOQ/BCL2/CASP3/CDKN1A/EGFR/FOS/GSTP1/HSD3B1/ICAM1/IL10/IL6/NR3C1/PTGS2/TNF | 14 |
| GO:0007584 | response to nutrient | 14/97 | 171/18866 | 2.15E-13 | 1.08E-11 | 4.61E-12 | ADIPOQ/CAT/CYP1A1/EGFR/F7/GSTP1/HMOX1/PIM1/PPARD/PPARG/PTEN/PTGS2/SPP1/TYR | 14 |
| GO:0048511 | rhythmic process | 17/97 | 305/18866 | 2.66E-13 | 1.32E-11 | 5.63E-12 | ADIPOQ/AHR/CASP3/EGFR/ESR1/F7/JUN/MAPK8/NOS2/NOS3/PGR/PPARA/PPARG/PTEN/SERPINE1/SREBF1/TP53 | 17 |
| GO:0060326 | cell chemotaxis | 17/97 | 311/18866 | 3.65E-13 | 1.79E-11 | 7.62E-12 | ALOX5/CCL2/CXCL8/CYP19A1/DPP4/F7/GSTP1/HSPB1/IL10/IL1B/IL4/IL6/MAPK1/MAPK3/PIK3CG/SERPINE1/VEGFA | 17 |
| GO:0032944 | regulation of mononuclear cell proliferation | 15/97 | 221/18866 | 4.41E-13 | 2.13E-11 | 9.10E-12 | AHR/BCL2/CASP3/CD40LG/CDKN1A/CRP/CTNNB1/ERBB2/IL10/IL1A/IL1B/IL2/IL4/IL6/VCAM1 | 15 |
| GO:0045785 | positive regulation of cell adhesion | 19/97 | 428/18866 | 5.32E-13 | 2.54E-11 | 1.08E-11 | AKT1/ALOX5/CAV1/CCL2/CD40LG/DPP4/ERBB2/ICAM1/IFNG/IL10/IL1A/IL1B/IL2/IL4/IL6/SELE/TNF/VCAM1/VEGFA | 19 |
| GO:0034612 | response to tumor necrosis factor | 17/97 | 320/18866 | 5.78E-13 | 2.73E-11 | 1.16E-11 | ADIPOQ/AKT1/APOB/CASP3/CCL2/CD40LG/CXCL8/GSTP1/ICAM1/IKBKB/MAPK1/MAPK3/NFKBIA/PTGS2/SELE/TNF/TP53 | 17 |
| GO:0019216 | regulation of lipid metabolic process | 19/97 | 431/18866 | 6.02E-13 | 2.80E-11 | 1.19E-11 | ADIPOQ/AKT1/APOB/CAV1/CYP1A1/F2/IFNG/IL1B/LACTB/PIK3CG/PPARA/PPARD/PPARG/PTGS2/RXRA/SOD1/SREBF1/TGFB1/TNF | 19 |
| GO:1901214 | regulation of neuron death | 17/97 | 321/18866 | 6.08E-13 | 2.80E-11 | 1.19E-11 | AKT1/BCL2/BCL2L1/CASP3/CCL2/CTNNB1/ERBB3/FOS/HMOX1/IFNG/IL10/JUN/PPARA/SOD1/STAT3/TNF/TP53 | 17 |
| GO:0050920 | regulation of chemotaxis | 15/97 | 229/18866 | 7.41E-13 | 3.33E-11 | 1.42E-11 | CCL2/CXCL8/CYP19A1/DPP4/F3/F7/GSTP1/HSPB1/IL4/IL6/MAPK1/MAPK3/SERPINE1/TGFB1/VEGFA | 15 |
| GO:1904705 | regulation of vascular associated smooth muscle cell proliferation | Nov-97 | 87/18866 | 7.52E-13 | 3.33E-11 | 1.42E-11 | ADIPOQ/CDKN1A/GSTP1/HMOX1/IL10/JUN/MMP2/MMP9/PPARG/PTEN/TNF | 11 |
| GO:1990874 | vascular associated smooth muscle cell proliferation | Nov-97 | 87/18866 | 7.52E-13 | 3.33E-11 | 1.42E-11 | ADIPOQ/CDKN1A/GSTP1/HMOX1/IL10/JUN/MMP2/MMP9/PPARG/PTEN/TNF | 11 |
| GO:0043491 | protein kinase B signaling | 16/97 | 278/18866 | 8.74E-13 | 3.83E-11 | 1.63E-11 | AKT1/CCL2/EGF/EGFR/ERBB2/ERBB3/ESR1/F10/F3/F7/IL1B/PIK3CG/PTEN/TGFB1/TNF/XDH | 16 |
| GO:0030595 | leukocyte chemotaxis | 15/97 | 232/18866 | 8.95E-13 | 3.87E-11 | 1.65E-11 | ALOX5/CCL2/CXCL8/CYP19A1/DPP4/F7/IL10/IL1B/IL4/IL6/MAPK1/MAPK3/PIK3CG/SERPINE1/VEGFA | 15 |
| GO:0060135 | maternal process involved in female pregnancy | Oct-97 | 64/18866 | 9.67E-13 | 4.14E-11 | 1.76E-11 | AKT1/AR/ESR1/MAPK1/MAPK3/PGR/PPARD/PTGS2/RXRA/SPP1 | 10 |
| GO:1901342 | regulation of vasculature development | 19/97 | 444/18866 | 1.02E-12 | 4.30E-11 | 1.83E-11 | ALOX5/CTNNB1/CXCL8/ERBB2/F3/HMOX1/HSPB1/IL10/IL1A/IL1B/IL6/NOS3/PPARG/PTGS2/SERPINE1/STAT3/TNF/VEGFA/XDH | 19 |
| GO:0050863 | regulation of T cell activation | 17/97 | 332/18866 | 1.05E-12 | 4.38E-11 | 1.87E-11 | AKT1/CASP3/CAV1/CCL2/CD40LG/CTNNB1/DPP4/ERBB2/IFNG/IL10/IL1A/IL1B/IL2/IL4/IL6/SOD1/VCAM1 | 17 |
| GO:0046651 | lymphocyte proliferation | 16/97 | 283/18866 | 1.15E-12 | 4.75E-11 | 2.03E-11 | AHR/BCL2/CASP3/CD40LG/CDKN1A/CTNNB1/ERBB2/IL10/IL1A/IL1B/IL2/IL4/IL6/PIK3CG/TP53/VCAM1 | 16 |
| GO:0033273 | response to vitamin | Nov-97 | 91/18866 | 1.25E-12 | 5.12E-11 | 2.18E-11 | CAT/CYP1A1/EGFR/F7/GSTP1/PIM1/PPARD/PPARG/PTGS2/SPP1/TYR | 11 |
| GO:0006953 | acute-phase response | Sep-97 | 47/18866 | 1.99E-12 | 8.06E-11 | 3.44E-11 | CRP/F2/IL1A/IL1B/IL6/PTGER3/PTGS2/STAT3/TNF | 9 |
| GO:0045765 | regulation of angiogenesis | 18/97 | 403/18866 | 2.11E-12 | 8.45E-11 | 3.60E-11 | ALOX5/CTNNB1/CXCL8/ERBB2/F3/HMOX1/HSPB1/IL10/IL1A/IL1B/IL6/NOS3/PPARG/PTGS2/SERPINE1/STAT3/TNF/VEGFA | 18 |
| GO:0030336 | negative regulation of cell migration | 17/97 | 350/18866 | 2.44E-12 | 9.66E-11 | 4.12E-11 | ADIPOQ/AKT1/BCL2/CCL2/CYP19A1/DPP4/GSTP1/HMOX1/IGFBP3/IL4/PPARD/PPARG/PTEN/SERPINE1/STAT3/TGFB1/TNF | 17 |
| GO:0006352 | DNA-templated transcription, initiation | 15/97 | 249/18866 | 2.49E-12 | 9.76E-11 | 4.16E-11 | AR/CDKN1A/CTNNB1/ESR1/JUN/MAPK3/NR3C1/NR3C2/PGR/PPARA/PPARD/PPARG/PTEN/RXRA/TP53 | 15 |
| GO:0051222 | positive regulation of protein transport | 17/97 | 354/18866 | 2.92E-12 | 1.13E-10 | 4.83E-11 | ACHE/BCL2/EGFR/ERBB2/IFNG/IL10/IL1A/IL1B/IL2/IL6/MAPK1/MAPK8/PPARD/PTGS2/TGFB1/TNF/TP53 | 17 |
| GO:0002673 | regulation of acute inflammatory response | Sep-97 | 49/18866 | 2.98E-12 | 1.14E-10 | 4.88E-11 | GSTP1/IL1B/IL4/IL6/PIK3CG/PPARG/PTGER3/PTGS2/TNF | 9 |
| GO:0070997 | neuron death | 17/97 | 360/18866 | 3.82E-12 | 1.45E-10 | 6.19E-11 | AKT1/BCL2/BCL2L1/CASP3/CCL2/CTNNB1/ERBB3/FOS/HMOX1/IFNG/IL10/JUN/PPARA/SOD1/STAT3/TNF/TP53 | 17 |
| GO:2000146 | negative regulation of cell motility | 17/97 | 365/18866 | 4.76E-12 | 1.79E-10 | 7.63E-11 | ADIPOQ/AKT1/BCL2/CCL2/CYP19A1/DPP4/GSTP1/HMOX1/IGFBP3/IL4/PPARD/PPARG/PTEN/SERPINE1/STAT3/TGFB1/TNF | 17 |
| GO:0038034 | signal transduction in absence of ligand | Oct-97 | 75/18866 | 5.05E-12 | 1.86E-10 | 7.94E-11 | AKT1/BCL2/BCL2L1/CASP3/ERBB3/IL1A/IL1B/IL2/IL4/TNF | 10 |
| GO:0097192 | extrinsic apoptotic signaling pathway in absence of ligand | Oct-97 | 75/18866 | 5.05E-12 | 1.86E-10 | 7.94E-11 | AKT1/BCL2/BCL2L1/CASP3/ERBB3/IL1A/IL1B/IL2/IL4/TNF | 10 |
| GO:0050730 | regulation of peptidyl-tyrosine phosphorylation | 15/97 | 263/18866 | 5.46E-12 | 1.99E-10 | 8.50E-11 | ADIPOQ/CAV1/EGF/EGFR/ERBB3/ICAM1/IFNG/IL2/IL4/IL6/STAT3/TGFB1/TNF/TP53/VEGFA | 15 |
| GO:0060965 | negative regulation of gene silencing by miRNA | Jul-97 | 20/18866 | 5.59E-12 | 2.02E-10 | 8.62E-11 | ESR1/IL6/PPARG/STAT3/TGFB1/TNF/TP53 | 7 |
| GO:1904951 | positive regulation of establishment of protein localization | 17/97 | 370/18866 | 5.91E-12 | 2.12E-10 | 9.02E-11 | ACHE/BCL2/EGFR/ERBB2/IFNG/IL10/IL1A/IL1B/IL2/IL6/MAPK1/MAPK8/PPARD/PTGS2/TGFB1/TNF/TP53 | 17 |
| GO:0050670 | regulation of lymphocyte proliferation | 14/97 | 219/18866 | 6.36E-12 | 2.25E-10 | 9.61E-11 | AHR/BCL2/CASP3/CD40LG/CDKN1A/CTNNB1/ERBB2/IL10/IL1A/IL1B/IL2/IL4/IL6/VCAM1 | 14 |
| GO:0150076 | neuroinflammatory response | Oct-97 | 77/18866 | 6.63E-12 | 2.33E-10 | 9.93E-11 | EGFR/IFNG/IL1B/IL4/IL6/JUN/MMP3/MMP9/PTGS2/TNF | 10 |
| GO:0018108 | peptidyl-tyrosine phosphorylation | 17/97 | 374/18866 | 7.01E-12 | 2.44E-10 | 1.04E-10 | ADIPOQ/CAV1/EGF/EGFR/ERBB2/ERBB3/ICAM1/IFNG/IL2/IL4/IL6/MAPK3/STAT3/TGFB1/TNF/TP53/VEGFA | 17 |
| GO:0031669 | cellular response to nutrient levels | 14/97 | 221/18866 | 7.19E-12 | 2.46E-10 | 1.05E-10 | ALB/BCL2/CDKN1A/HMOX1/ICAM1/JUN/MAPK1/MAPK3/MAPK8/PIM1/PPARA/PPARG/SREBF1/TP53 | 14 |
| GO:0071241 | cellular response to inorganic substance | 14/97 | 221/18866 | 7.19E-12 | 2.46E-10 | 1.05E-10 | AKT1/CYP1A1/CYP1A2/EGFR/FOS/HMOX1/JUN/MAPK1/MAPK3/MAPK8/MMP3/MMP9/PTGS2/SOD1 | 14 |
| GO:0018212 | peptidyl-tyrosine modification | 17/97 | 377/18866 | 7.95E-12 | 2.69E-10 | 1.15E-10 | ADIPOQ/CAV1/EGF/EGFR/ERBB2/ERBB3/ICAM1/IFNG/IL2/IL4/IL6/MAPK3/STAT3/TGFB1/TNF/TP53/VEGFA | 17 |
| GO:0006809 | nitric oxide biosynthetic process | Oct-97 | 79/18866 | 8.63E-12 | 2.90E-10 | 1.23E-10 | AKT1/CAV1/ICAM1/IFNG/IL10/IL1B/NOS2/NOS3/PTGS2/TNF | 10 |
| GO:0010876 | lipid localization | 18/97 | 440/18866 | 9.13E-12 | 3.03E-10 | 1.29E-10 | ADIPOQ/AKT1/APOB/CAV1/CRP/CYP19A1/EGF/IL1B/IL6/NFKBIA/NOS2/PON1/PPARA/PPARD/PPARG/RXRA/SPP1/TNF | 18 |
| GO:1903034 | regulation of response to wounding | 13/97 | 183/18866 | 1.00E-11 | 3.31E-10 | 1.41E-10 | ALOX5/CAV1/F2/F3/F7/IL10/NOS3/PLAT/PTEN/SERPINE1/SPP1/THBD/TNF | 13 |
| GO:0001936 | regulation of endothelial cell proliferation | 13/97 | 184/18866 | 1.08E-11 | 3.52E-10 | 1.50E-10 | AKT1/ALOX5/CAV1/CCL2/F3/HMOX1/IL10/JUN/PPARG/STAT3/TNF/VEGFA/XDH | 13 |
| GO:0019915 | lipid storage | Oct-97 | 81/18866 | 1.12E-11 | 3.61E-10 | 1.54E-10 | APOB/CAV1/CRP/IL1B/IL6/NFKBIA/PPARA/PPARD/PPARG/TNF | 10 |
| GO:0001819 | positive regulation of cytokine production | 18/97 | 447/18866 | 1.19E-11 | 3.80E-10 | 1.62E-10 | ADIPOQ/CD40LG/CTNNB1/F3/HSPB1/IFNG/IL10/IL1A/IL1B/IL2/IL4/IL6/PTGS2/SERPINE1/SOD1/STAT3/TGFB1/TNF | 18 |
| GO:0010888 | negative regulation of lipid storage | Jul-97 | 22/18866 | 1.22E-11 | 3.88E-10 | 1.66E-10 | CRP/IL6/NFKBIA/PPARA/PPARD/PPARG/TNF | 7 |
| GO:0007566 | embryo implantation | Sep-97 | 58/18866 | 1.49E-11 | 4.69E-10 | 2.00E-10 | IL1B/MMP2/MMP9/PPARD/PTGS2/RXRA/SOD1/SPP1/VEGFA | 9 |
| GO:0014074 | response to purine-containing compound | Dec-97 | 149/18866 | 1.51E-11 | 4.72E-10 | 2.01E-10 | ADIPOQ/AHR/FOS/IL1B/PIK3CG/PPARG/PTEN/PTGS2/SOD1/SREBF1/THBD/TYR | 12 |
| GO:1990748 | cellular detoxification | Nov-97 | 114/18866 | 1.54E-11 | 4.79E-10 | 2.04E-10 | ALB/CAT/GSR/GSTM1/GSTP1/MPO/NOS3/PIM1/PTGS2/SOD1/TNF | 11 |
| GO:0046209 | nitric oxide metabolic process | Oct-97 | 84/18866 | 1.62E-11 | 4.98E-10 | 2.13E-10 | AKT1/CAV1/ICAM1/IFNG/IL10/IL1B/NOS2/NOS3/PTGS2/TNF | 10 |
| GO:0006631 | fatty acid metabolic process | 17/97 | 396/18866 | 1.73E-11 | 5.16E-10 | 2.20E-10 | ADIPOQ/AKT1/ALOX5/CAV1/CYP1A1/CYP1A2/CYP3A4/GSTM1/GSTP1/IL1B/MAPK3/PON1/PPARA/PPARD/PPARG/PTGS2/SREBF1 | 17 |
| GO:0060149 | negative regulation of posttranscriptional gene silencing | Jul-97 | 23/18866 | 1.75E-11 | 5.16E-10 | 2.20E-10 | ESR1/IL6/PPARG/STAT3/TGFB1/TNF/TP53 | 7 |
| GO:0060967 | negative regulation of gene silencing by RNA | Jul-97 | 23/18866 | 1.75E-11 | 5.16E-10 | 2.20E-10 | ESR1/IL6/PPARG/STAT3/TGFB1/TNF/TP53 | 7 |
| GO:1903798 | regulation of production of miRNAs involved in gene silencing by miRNA | Jul-97 | 23/18866 | 1.75E-11 | 5.16E-10 | 2.20E-10 | EGFR/ESR1/IL6/STAT3/TGFB1/TNF/TP53 | 7 |
| GO:0031663 | lipopolysaccharide-mediated signaling pathway | Sep-97 | 59/18866 | 1.75E-11 | 5.16E-10 | 2.20E-10 | AKT1/CCL2/IL1B/MAPK1/MAPK3/NFKBIA/NOS3/TGFB1/TNF | 9 |
| GO:0040013 | negative regulation of locomotion | 17/97 | 397/18866 | 1.80E-11 | 5.27E-10 | 2.25E-10 | ADIPOQ/AKT1/BCL2/CCL2/CYP19A1/DPP4/GSTP1/HMOX1/IGFBP3/IL4/PPARD/PPARG/PTEN/SERPINE1/STAT3/TGFB1/TNF | 17 |
| GO:0071248 | cellular response to metal ion | 13/97 | 193/18866 | 1.97E-11 | 5.73E-10 | 2.44E-10 | AKT1/CYP1A1/CYP1A2/EGFR/FOS/HMOX1/JUN/MAPK1/MAPK3/MAPK8/MMP9/PTGS2/SOD1 | 13 |
| GO:0051271 | negative regulation of cellular component movement | 17/97 | 400/18866 | 2.02E-11 | 5.84E-10 | 2.49E-10 | ADIPOQ/AKT1/BCL2/CCL2/CYP19A1/DPP4/GSTP1/HMOX1/IGFBP3/IL4/PPARD/PPARG/PTEN/SERPINE1/STAT3/TGFB1/TNF | 17 |
| GO:1904019 | epithelial cell apoptotic process | Nov-97 | 117/18866 | 2.06E-11 | 5.87E-10 | 2.50E-10 | BCL2L1/CCL2/CD40LG/HMOX1/ICAM1/IL10/IL4/IL6/PIK3CG/SERPINE1/TNF | 11 |
| GO:0045834 | positive regulation of lipid metabolic process | Dec-97 | 153/18866 | 2.06E-11 | 5.87E-10 | 2.50E-10 | ADIPOQ/AKT1/F2/IFNG/IL1B/PPARA/PPARD/PPARG/PTGS2/SREBF1/TGFB1/TNF | 12 |
| GO:0014065 | phosphatidylinositol 3-kinase signaling | Dec-97 | 154/18866 | 2.23E-11 | 6.23E-10 | 2.66E-10 | AKT1/CAT/EGFR/ERBB2/ERBB3/F2/MAPK1/MAPK3/PIK3CG/PPARD/PTEN/TNF | 12 |
| GO:0042098 | T cell proliferation | 13/97 | 195/18866 | 2.24E-11 | 6.23E-10 | 2.66E-10 | CASP3/CD40LG/CTNNB1/ERBB2/IL10/IL1A/IL1B/IL2/IL4/IL6/PIK3CG/TP53/VCAM1 | 13 |
| GO:0050731 | positive regulation of peptidyl-tyrosine phosphorylation | 13/97 | 195/18866 | 2.24E-11 | 6.23E-10 | 2.66E-10 | ADIPOQ/EGF/ERBB3/ICAM1/IFNG/IL2/IL4/IL6/STAT3/TGFB1/TNF/TP53/VEGFA | 13 |
| GO:2001057 | reactive nitrogen species metabolic process | Oct-97 | 87/18866 | 2.32E-11 | 6.39E-10 | 2.73E-10 | AKT1/CAV1/ICAM1/IFNG/IL10/IL1B/NOS2/NOS3/PTGS2/TNF | 10 |
| GO:0043112 | receptor metabolic process | 13/97 | 198/18866 | 2.72E-11 | 7.44E-10 | 3.17E-10 | ACHE/ADIPOQ/CAV1/CXCL8/EGF/IFNG/IL10/PPARA/PPARG/SELE/TGFB1/TNF/VEGFA | 13 |
| GO:0001935 | endothelial cell proliferation | 13/97 | 199/18866 | 2.90E-11 | 7.87E-10 | 3.36E-10 | AKT1/ALOX5/CAV1/CCL2/F3/HMOX1/IL10/JUN/PPARG/STAT3/TNF/VEGFA/XDH | 13 |
| GO:0050729 | positive regulation of inflammatory response | Dec-97 | 158/18866 | 3.02E-11 | 8.14E-10 | 3.47E-10 | EGFR/IFNG/IL1B/IL2/IL6/NFKBIA/PIK3CG/PTGER3/PTGS2/SERPINE1/STAT3/TNF | 12 |
| GO:0050708 | regulation of protein secretion | 16/97 | 352/18866 | 3.10E-11 | 8.29E-10 | 3.54E-10 | ACHE/ALOX5/CD40LG/DPP4/EGFR/IFNG/IL10/IL1A/IL1B/IL2/IL6/NOS2/PPARD/SREBF1/TGFB1/TNF | 16 |
| GO:0097237 | cellular response to toxic substance | Nov-97 | 122/18866 | 3.26E-11 | 8.66E-10 | 3.69E-10 | ALB/CAT/GSR/GSTM1/GSTP1/MPO/NOS3/PIM1/PTGS2/SOD1/TNF | 11 |
| GO:0031099 | regeneration | 13/97 | 201/18866 | 3.28E-11 | 8.67E-10 | 3.70E-10 | BCL2/CDKN1A/EGFR/F7/GSTP1/HMOX1/IL10/IL6/JUN/PPARD/PPARG/PTEN/SPP1 | 13 |
| GO:0070920 | regulation of production of small RNA involved in gene silencing by RNA | Jul-97 | 25/18866 | 3.40E-11 | 8.91E-10 | 3.80E-10 | EGFR/ESR1/IL6/STAT3/TGFB1/TNF/TP53 | 7 |
| GO:0002688 | regulation of leukocyte chemotaxis | Nov-97 | 124/18866 | 3.89E-11 | 1.01E-09 | 4.32E-10 | CCL2/CXCL8/CYP19A1/DPP4/F7/IL4/IL6/MAPK1/MAPK3/SERPINE1/VEGFA | 11 |
| GO:1905953 | negative regulation of lipid localization | Sep-97 | 65/18866 | 4.33E-11 | 1.12E-09 | 4.78E-10 | AKT1/CRP/EGF/IL6/NFKBIA/PPARA/PPARD/PPARG/TNF | 9 |
| GO:0042326 | negative regulation of phosphorylation | 18/97 | 484/18866 | 4.39E-11 | 1.13E-09 | 4.80E-10 | ADIPOQ/AKT1/CASP3/CAV1/CDKN1A/GSTP1/HSPB1/IFNG/IGFBP3/IL1B/IL2/JUN/MYC/PPARA/PTEN/STAT3/TGFB1/XDH | 18 |
| GO:0051896 | regulation of protein kinase B signaling | 14/97 | 253/18866 | 4.41E-11 | 1.13E-09 | 4.80E-10 | AKT1/EGF/EGFR/ERBB2/ERBB3/ESR1/F10/F3/F7/PIK3CG/PTEN/TGFB1/TNF/XDH | 14 |
| GO:1903799 | negative regulation of production of miRNAs involved in gene silencing by miRNA | Jun-97 | 14/18866 | 4.59E-11 | 1.16E-09 | 4.95E-10 | ESR1/IL6/STAT3/TGFB1/TNF/TP53 | 6 |
| GO:0010631 | epithelial cell migration | 16/97 | 365/18866 | 5.31E-11 | 1.34E-09 | 5.69E-10 | AKT1/DPP4/HMOX1/HSPB1/IFNG/IL4/JUN/MMP9/NOS3/PPARD/PPARG/PTEN/PTGS2/TGFB1/TNF/VEGFA | 16 |
| GO:0018105 | peptidyl-serine phosphorylation | 15/97 | 310/18866 | 5.65E-11 | 1.41E-09 | 6.01E-10 | AKT1/BCL2/CAV1/EGFR/IFNG/IKBKB/IL6/MAPK1/MAPK3/MAPK8/PTEN/PTGS2/TGFB1/TNF/VEGFA | 15 |
| GO:0090132 | epithelium migration | 16/97 | 368/18866 | 5.99E-11 | 1.49E-09 | 6.34E-10 | AKT1/DPP4/HMOX1/HSPB1/IFNG/IL4/JUN/MMP9/NOS3/PPARD/PPARG/PTEN/PTGS2/TGFB1/TNF/VEGFA | 16 |
| GO:0051100 | negative regulation of binding | Dec-97 | 169/18866 | 6.65E-11 | 1.64E-09 | 6.99E-10 | ADIPOQ/AKT1/CAV1/CDKN1A/HMOX1/IL10/JUN/MAPK3/MAPK8/NFKBIA/PPARA/SLPI | 12 |
| GO:0090130 | tissue migration | 16/97 | 374/18866 | 7.62E-11 | 1.87E-09 | 7.95E-10 | AKT1/DPP4/HMOX1/HSPB1/IFNG/IL4/JUN/MMP9/NOS3/PPARD/PPARG/PTEN/PTGS2/TGFB1/TNF/VEGFA | 16 |
| GO:0030522 | intracellular receptor signaling pathway | 14/97 | 265/18866 | 8.17E-11 | 1.99E-09 | 8.48E-10 | AHR/AR/ESR1/NFKBIA/NR3C1/NR3C2/PGR/PIM1/PPARA/PPARD/PPARG/RXRA/SREBF1/STAT3 | 14 |
| GO:0032800 | receptor biosynthetic process | Jul-97 | 28/18866 | 8.26E-11 | 2.00E-09 | 8.52E-10 | ACHE/ADIPOQ/IFNG/IL10/PPARA/PPARG/TNF | 7 |
| GO:0046683 | response to organophosphorus | Nov-97 | 134/18866 | 9.07E-11 | 2.17E-09 | 9.23E-10 | ADIPOQ/AHR/FOS/IL1B/PIK3CG/PTEN/PTGS2/SOD1/SREBF1/THBD/TYR | 11 |
| GO:0007623 | circadian rhythm | 13/97 | 218/18866 | 9.07E-11 | 2.17E-09 | 9.23E-10 | ADIPOQ/AHR/EGFR/F7/JUN/MAPK8/NOS2/PPARA/PPARG/PTEN/SERPINE1/SREBF1/TP53 | 13 |
| GO:0008585 | female gonad development | Oct-97 | 100/18866 | 9.49E-11 | 2.25E-09 | 9.60E-10 | BCL2/BCL2L1/CASP3/CYP19A1/ESR1/ICAM1/NOS3/PGR/SOD1/VEGFA | 10 |
| GO:0002791 | regulation of peptide secretion | 16/97 | 381/18866 | 1.00E-10 | 2.36E-09 | 1.01E-09 | ACHE/ALOX5/CD40LG/DPP4/EGFR/IFNG/IL10/IL1A/IL1B/IL2/IL6/NOS2/PPARD/SREBF1/TGFB1/TNF | 16 |
| GO:0032637 | interleukin-8 production | Oct-97 | 101/18866 | 1.05E-10 | 2.46E-09 | 1.05E-09 | ADIPOQ/CRP/F3/IL10/IL1B/IL6/NOS2/SERPINE1/STAT3/TNF | 10 |
| GO:0070371 | ERK1 and ERK2 cascade | 15/97 | 325/18866 | 1.10E-10 | 2.55E-09 | 1.09E-09 | ADIPOQ/CCL2/EGF/EGFR/ERBB2/GSTP1/ICAM1/IL1B/JUN/MAPK1/MAPK3/MYC/PTEN/TGFB1/TNF | 15 |
| GO:0098754 | detoxification | Nov-97 | 138/18866 | 1.25E-10 | 2.89E-09 | 1.23E-09 | ALB/CAT/GSR/GSTM1/GSTP1/MPO/NOS3/PIM1/PTGS2/SOD1/TNF | 11 |
| GO:0048661 | positive regulation of smooth muscle cell proliferation | Oct-97 | 103/18866 | 1.28E-10 | 2.94E-09 | 1.25E-09 | AKT1/EGFR/HMOX1/IL10/IL6/JUN/MMP2/MMP9/PTGS2/TNF | 10 |
| GO:0006801 | superoxide metabolic process | Sep-97 | 74/18866 | 1.44E-10 | 3.30E-09 | 1.40E-09 | CRP/EGFR/GSTP1/MPO/NOS2/NOS3/SOD1/TGFB1/TNF | 9 |
| GO:0009411 | response to UV | Nov-97 | 140/18866 | 1.46E-10 | 3.31E-09 | 1.41E-09 | AKT1/BCL2/CASP3/CAT/CDKN1A/EGFR/MAPK8/MYC/PTGS2/TP53/TYR | 11 |
| GO:0018209 | peptidyl-serine modification | 15/97 | 333/18866 | 1.54E-10 | 3.47E-09 | 1.48E-09 | AKT1/BCL2/CAV1/EGFR/IFNG/IKBKB/IL6/MAPK1/MAPK3/MAPK8/PTEN/PTGS2/TGFB1/TNF/VEGFA | 15 |
| GO:0046545 | development of primary female sexual characteristics | Oct-97 | 105/18866 | 1.55E-10 | 3.47E-09 | 1.48E-09 | BCL2/BCL2L1/CASP3/CYP19A1/ESR1/ICAM1/NOS3/PGR/SOD1/VEGFA | 10 |
| GO:0002687 | positive regulation of leukocyte migration | Nov-97 | 142/18866 | 1.70E-10 | 3.79E-09 | 1.62E-09 | CXCL8/F7/ICAM1/IL4/IL6/MAPK1/MAPK3/SELE/SERPINE1/TNF/VEGFA | 11 |
| GO:1903426 | regulation of reactive oxygen species biosynthetic process | Oct-97 | 107/18866 | 1.87E-10 | 4.15E-09 | 1.77E-09 | AKT1/ALOX5/CAV1/ICAM1/IFNG/IL10/IL1B/PTGS2/STAT3/TNF | 10 |
| GO:0002683 | negative regulation of immune system process | 17/97 | 463/18866 | 1.97E-10 | 4.33E-09 | 1.85E-09 | ADIPOQ/AKT1/CASP3/CCL2/CTNNB1/CYP19A1/DPP4/ERBB2/HMOX1/IL10/IL2/IL4/MYC/NFKBIA/PPARG/TGFB1/TNF | 17 |
| GO:0050921 | positive regulation of chemotaxis | Nov-97 | 144/18866 | 1.98E-10 | 4.33E-09 | 1.85E-09 | CXCL8/F3/F7/HSPB1/IL4/IL6/MAPK1/MAPK3/SERPINE1/TGFB1/VEGFA | 11 |
| GO:0033135 | regulation of peptidyl-serine phosphorylation | Nov-97 | 145/18866 | 2.13E-10 | 4.64E-09 | 1.98E-09 | AKT1/BCL2/CAV1/EGFR/IFNG/IL6/PTEN/PTGS2/TGFB1/TNF/VEGFA | 11 |
| GO:0043405 | regulation of MAP kinase activity | 15/97 | 342/18866 | 2.23E-10 | 4.83E-09 | 2.06E-09 | ADIPOQ/CAV1/CD40LG/EGF/EGFR/ERBB2/GSTP1/IL1B/MAPK1/MAPK3/PIK3CG/SOD1/TGFB1/TNF/VEGFA | 15 |
| GO:0033138 | positive regulation of peptidyl-serine phosphorylation | Oct-97 | 109/18866 | 2.25E-10 | 4.85E-09 | 2.07E-09 | AKT1/BCL2/CAV1/EGFR/IFNG/IL6/PTGS2/TGFB1/TNF/VEGFA | 10 |
| GO:0010883 | regulation of lipid storage | Aug-97 | 52/18866 | 2.27E-10 | 4.87E-09 | 2.07E-09 | APOB/CRP/IL6/NFKBIA/PPARA/PPARD/PPARG/TNF | 8 |
| GO:0007596 | blood coagulation | 15/97 | 343/18866 | 2.32E-10 | 4.94E-09 | 2.11E-09 | CAV1/CD40LG/F10/F2/F3/F7/HSPB1/IL6/MAPK1/MAPK3/NOS3/PIK3CG/PLAT/SERPINE1/THBD | 15 |
| GO:0006367 | transcription initiation from RNA polymerase II promoter | Dec-97 | 189/18866 | 2.44E-10 | 5.17E-09 | 2.20E-09 | AR/CDKN1A/ESR1/NR3C1/NR3C2/PGR/PPARA/PPARD/PPARG/PTEN/RXRA/TP53 | 12 |
| GO:0071902 | positive regulation of protein serine/threonine kinase activity | 15/97 | 345/18866 | 2.52E-10 | 5.30E-09 | 2.26E-09 | ADIPOQ/AKT1/CD40LG/EGF/EGFR/ERBB2/IFNG/IL1B/MAPK1/MAPK3/PIK3CG/SOD1/TGFB1/TNF/VEGFA | 15 |
| GO:0002696 | positive regulation of leukocyte activation | 16/97 | 406/18866 | 2.55E-10 | 5.33E-09 | 2.27E-09 | AKT1/BCL2/CAV1/CCL2/CD40LG/CDKN1A/DPP4/IFNG/IL10/IL1A/IL1B/IL2/IL4/IL6/TGFB1/VCAM1 | 16 |
| GO:0003018 | vascular process in circulatory system | Dec-97 | 190/18866 | 2.60E-10 | 5.40E-09 | 2.30E-09 | AKT1/CAV1/CRP/EGFR/HMOX1/ICAM1/NOS3/PPARD/PTGS2/SOD1/TGFB1/VEGFA | 12 |
| GO:1902105 | regulation of leukocyte differentiation | 14/97 | 290/18866 | 2.68E-10 | 5.55E-09 | 2.37E-09 | ADIPOQ/CTNNB1/ERBB2/FOS/IFNG/IL1A/IL1B/IL2/IL4/JUN/MYC/SOD1/TGFB1/TNF | 14 |
| GO:0007599 | hemostasis | 15/97 | 348/18866 | 2.84E-10 | 5.84E-09 | 2.49E-09 | CAV1/CD40LG/F10/F2/F3/F7/HSPB1/IL6/MAPK1/MAPK3/NOS3/PIK3CG/PLAT/SERPINE1/THBD | 15 |
| GO:0048015 | phosphatidylinositol-mediated signaling | Dec-97 | 192/18866 | 2.93E-10 | 5.99E-09 | 2.56E-09 | AKT1/CAT/EGFR/ERBB2/ERBB3/F2/MAPK1/MAPK3/PIK3CG/PPARD/PTEN/TNF | 12 |
| GO:0050817 | coagulation | 15/97 | 349/18866 | 2.96E-10 | 6.01E-09 | 2.56E-09 | CAV1/CD40LG/F10/F2/F3/F7/HSPB1/IL6/MAPK1/MAPK3/NOS3/PIK3CG/PLAT/SERPINE1/THBD | 15 |
| GO:0070665 | positive regulation of leukocyte proliferation | Nov-97 | 150/18866 | 3.07E-10 | 6.21E-09 | 2.65E-09 | BCL2/CD40LG/CDKN1A/IL1A/IL1B/IL2/IL4/IL6/MAPK1/MAPK3/VCAM1 | 11 |
| GO:0001503 | ossification | 16/97 | 412/18866 | 3.16E-10 | 6.36E-09 | 2.71E-09 | ACHE/AKT1/ALOX5/BCL2/CAT/CTNNB1/EGFR/IGFBP3/IL6/MAPK1/MAPK3/MMP2/PTGS2/SPP1/TGFB1/TNF | 16 |
| GO:0061041 | regulation of wound healing | Nov-97 | 151/18866 | 3.30E-10 | 6.60E-09 | 2.81E-09 | ALOX5/CAV1/F2/F3/F7/NOS3/PLAT/PTEN/SERPINE1/THBD/TNF | 11 |
| GO:0042759 | long-chain fatty acid biosynthetic process | Jul-97 | 34/18866 | 3.66E-10 | 7.26E-09 | 3.10E-09 | ALOX5/CYP1A1/CYP1A2/CYP3A4/GSTM1/GSTP1/PTGS2 | 7 |
| GO:0071356 | cellular response to tumor necrosis factor | 14/97 | 297/18866 | 3.67E-10 | 7.26E-09 | 3.10E-09 | ADIPOQ/AKT1/APOB/CCL2/CD40LG/CXCL8/GSTP1/ICAM1/IKBKB/MAPK1/MAPK3/NFKBIA/TNF/TP53 | 14 |
| GO:0048017 | inositol lipid-mediated signaling | Dec-97 | 196/18866 | 3.72E-10 | 7.28E-09 | 3.11E-09 | AKT1/CAT/EGFR/ERBB2/ERBB3/F2/MAPK1/MAPK3/PIK3CG/PPARD/PTEN/TNF | 12 |
| GO:0050728 | negative regulation of inflammatory response | Dec-97 | 196/18866 | 3.72E-10 | 7.28E-09 | 3.11E-09 | ADIPOQ/ALOX5/CYP19A1/F2/GSTP1/IL10/IL2/IL4/PPARA/PPARD/PPARG/SOD1 | 12 |
| GO:0051251 | positive regulation of lymphocyte activation | 15/97 | 357/18866 | 4.05E-10 | 7.89E-09 | 3.36E-09 | AKT1/BCL2/CAV1/CCL2/CD40LG/CDKN1A/DPP4/IFNG/IL1A/IL1B/IL2/IL4/IL6/TGFB1/VCAM1 | 15 |
| GO:0048145 | regulation of fibroblast proliferation | Sep-97 | 83/18866 | 4.13E-10 | 7.97E-09 | 3.40E-09 | CDKN1A/CTNNB1/EGFR/ESR1/GSTP1/JUN/MYC/PPARG/TP53 | 9 |
| GO:0048708 | astrocyte differentiation | Sep-97 | 83/18866 | 4.13E-10 | 7.97E-09 | 3.40E-09 | EGFR/F2/IFNG/IL1B/IL6/MAPK1/MAPK3/STAT3/TNF | 9 |
| GO:0050867 | positive regulation of cell activation | 16/97 | 421/18866 | 4.33E-10 | 8.31E-09 | 3.54E-09 | AKT1/BCL2/CAV1/CCL2/CD40LG/CDKN1A/DPP4/IFNG/IL10/IL1A/IL1B/IL2/IL4/IL6/TGFB1/VCAM1 | 16 |
| GO:0010632 | regulation of epithelial cell migration | 14/97 | 301/18866 | 4.37E-10 | 8.34E-09 | 3.56E-09 | AKT1/HMOX1/HSPB1/IFNG/IL4/JUN/MMP9/NOS3/PPARG/PTEN/PTGS2/TGFB1/TNF/VEGFA | 14 |
| GO:0048144 | fibroblast proliferation | Sep-97 | 84/18866 | 4.61E-10 | 8.75E-09 | 3.73E-09 | CDKN1A/CTNNB1/EGFR/ESR1/GSTP1/JUN/MYC/PPARG/TP53 | 9 |
| GO:0001890 | placenta development | Nov-97 | 156/18866 | 4.68E-10 | 8.84E-09 | 3.77E-09 | AKT1/EGFR/IL10/MAPK1/MAPK3/PPARD/PPARG/PTGS2/RXRA/SOD1/SPP1 | 11 |
| GO:0042743 | hydrogen peroxide metabolic process | Aug-97 | 57/18866 | 4.89E-10 | 9.20E-09 | 3.92E-09 | CAT/CYP1A1/CYP1A2/EGFR/MMP3/MPO/SOD1/STAT3 | 8 |
| GO:0046660 | female sex differentiation | Oct-97 | 119/18866 | 5.39E-10 | 1.01E-08 | 4.30E-09 | BCL2/BCL2L1/CASP3/CYP19A1/ESR1/ICAM1/NOS3/PGR/SOD1/VEGFA | 10 |
| GO:0051052 | regulation of DNA metabolic process | 15/97 | 365/18866 | 5.49E-10 | 1.02E-08 | 4.36E-09 | ADIPOQ/AKT1/CDKN1A/CTNNB1/EGFR/IL10/IL2/IL4/IL6/MAPK1/MAPK3/MYC/PPARG/TGFB1/TP53 | 15 |
| GO:0001893 | maternal placenta development | Jul-97 | 36/18866 | 5.63E-10 | 1.04E-08 | 4.45E-09 | AKT1/MAPK1/MAPK3/PPARD/PTGS2/RXRA/SPP1 | 7 |
| GO:1903706 | regulation of hemopoiesis | 17/97 | 498/18866 | 6.03E-10 | 1.11E-08 | 4.74E-09 | ADIPOQ/CTNNB1/ERBB2/FOS/IFNG/IL1A/IL1B/IL2/IL4/JUN/MYC/NFKBIA/PIM1/SOD1/STAT3/TGFB1/TNF | 17 |
| GO:0030098 | lymphocyte differentiation | 15/97 | 368/18866 | 6.15E-10 | 1.13E-08 | 4.81E-09 | BCL2/CD40LG/CTNNB1/ERBB2/IFNG/IL10/IL1A/IL1B/IL2/IL4/IL6/SOD1/STAT3/TP53/VCAM1 | 15 |
| GO:0060969 | negative regulation of gene silencing | Jul-97 | 37/18866 | 6.92E-10 | 1.26E-08 | 5.38E-09 | ESR1/IL6/PPARG/STAT3/TGFB1/TNF/TP53 | 7 |
| GO:1903532 | positive regulation of secretion by cell | 14/97 | 313/18866 | 7.27E-10 | 1.32E-08 | 5.63E-09 | ACHE/CYP19A1/EGFR/IFNG/IL10/IL1A/IL1B/IL2/IL4/IL6/PPARD/SPP1/TGFB1/TNF | 14 |
| GO:0045766 | positive regulation of angiogenesis | Dec-97 | 208/18866 | 7.36E-10 | 1.33E-08 | 5.67E-09 | CXCL8/F3/HMOX1/HSPB1/IL10/IL1A/IL1B/NOS3/PTGS2/SERPINE1/STAT3/VEGFA | 12 |
| GO:0007259 | receptor signaling pathway via JAK-STAT | Nov-97 | 164/18866 | 7.98E-10 | 1.43E-08 | 6.10E-09 | CAV1/CCL2/EGF/F2/IFNG/IL10/IL2/IL4/IL6/STAT3/TNF | 11 |
| GO:0001660 | fever generation | May-97 | 10/18866 | 7.99E-10 | 1.43E-08 | 6.10E-09 | IL1A/IL1B/PTGER3/PTGS2/TNF | 5 |
| GO:0022612 | gland morphogenesis | Oct-97 | 124/18866 | 8.09E-10 | 1.43E-08 | 6.12E-09 | AR/BCL2/CAV1/EGFR/ESR1/IL6/PGR/RXRA/TGFB1/TNF | 10 |
| GO:0051101 | regulation of DNA binding | Oct-97 | 124/18866 | 8.09E-10 | 1.43E-08 | 6.12E-09 | CTNNB1/EGF/HMOX1/IFNG/JUN/MAPK8/MMP9/NFKBIA/PPARG/TGFB1 | 10 |
| GO:0002573 | myeloid leukocyte differentiation | Dec-97 | 210/18866 | 8.21E-10 | 1.45E-08 | 6.18E-09 | ADIPOQ/CTNNB1/FOS/IFNG/IL4/JUN/MMP9/MYC/PPARG/TGFB1/TNF/VEGFA | 12 |
| GO:0001894 | tissue homeostasis | 13/97 | 261/18866 | 8.37E-10 | 1.47E-08 | 6.27E-09 | ALB/BCL2/CTNNB1/EGFR/HSPB1/IL6/LYZ/NOS3/PTGS2/RHO/SOD1/SPP1/VEGFA | 13 |
| GO:0010742 | macrophage derived foam cell differentiation | Jul-97 | 38/18866 | 8.45E-10 | 1.47E-08 | 6.27E-09 | ADIPOQ/APOB/CRP/NFKBIA/PPARA/PPARG/TGFB1 | 7 |
| GO:0090077 | foam cell differentiation | Jul-97 | 38/18866 | 8.45E-10 | 1.47E-08 | 6.27E-09 | ADIPOQ/APOB/CRP/NFKBIA/PPARA/PPARG/TGFB1 | 7 |
| GO:0050679 | positive regulation of epithelial cell proliferation | Dec-97 | 211/18866 | 8.67E-10 | 1.50E-08 | 6.40E-09 | AKT1/AR/CTNNB1/EGFR/ERBB2/F3/HMOX1/IL10/JUN/MYC/STAT3/VEGFA | 12 |
| GO:1901215 | negative regulation of neuron death | Dec-97 | 212/18866 | 9.15E-10 | 1.58E-08 | 6.73E-09 | AKT1/BCL2/BCL2L1/CCL2/CTNNB1/ERBB3/HMOX1/IL10/JUN/PPARA/SOD1/STAT3 | 12 |
| GO:0001933 | negative regulation of protein phosphorylation | 16/97 | 444/18866 | 9.37E-10 | 1.61E-08 | 6.85E-09 | ADIPOQ/AKT1/CASP3/CAV1/CDKN1A/GSTP1/HSPB1/IFNG/IGFBP3/IL1B/IL2/JUN/MYC/PTEN/TGFB1/XDH | 16 |
| GO:0042129 | regulation of T cell proliferation | Nov-97 | 167/18866 | 9.68E-10 | 1.65E-08 | 7.05E-09 | CASP3/CD40LG/CTNNB1/ERBB2/IL10/IL1A/IL1B/IL2/IL4/IL6/VCAM1 | 11 |
| GO:0043523 | regulation of neuron apoptotic process | Dec-97 | 214/18866 | 1.02E-09 | 1.72E-08 | 7.33E-09 | BCL2/BCL2L1/CASP3/CCL2/CTNNB1/ERBB3/HMOX1/IL10/JUN/SOD1/TNF/TP53 | 12 |
| GO:0050870 | positive regulation of T cell activation | Dec-97 | 214/18866 | 1.02E-09 | 1.72E-08 | 7.33E-09 | AKT1/CAV1/CCL2/CD40LG/DPP4/IFNG/IL1A/IL1B/IL2/IL4/IL6/VCAM1 | 12 |
| GO:0014066 | regulation of phosphatidylinositol 3-kinase signaling | Oct-97 | 127/18866 | 1.02E-09 | 1.72E-08 | 7.33E-09 | CAT/EGFR/ERBB3/F2/MAPK1/MAPK3/PIK3CG/PPARD/PTEN/TNF | 10 |
| GO:1904706 | negative regulation of vascular associated smooth muscle cell proliferation | Jul-97 | 39/18866 | 1.03E-09 | 1.72E-08 | 7.33E-09 | ADIPOQ/CDKN1A/GSTP1/HMOX1/IL10/PPARG/PTEN | 7 |
| GO:0009314 | response to radiation | 16/97 | 447/18866 | 1.03E-09 | 1.72E-08 | 7.35E-09 | AKT1/BCL2/BCL2L1/CASP3/CAT/CDKN1A/EGFR/FOS/ICAM1/MAPK8/MYC/PTGS2/RHO/THBD/TP53/TYR | 16 |
| GO:0016125 | sterol metabolic process | Nov-97 | 169/18866 | 1.10E-09 | 1.83E-08 | 7.79E-09 | APOB/CAT/CYP19A1/CYP1A2/CYP3A4/IL4/PON1/PPARD/RXRA/SOD1/SREBF1 | 11 |
| GO:0046883 | regulation of hormone secretion | 13/97 | 267/18866 | 1.10E-09 | 1.83E-08 | 7.79E-09 | ADIPOQ/ALOX5/CYP19A1/DPP4/EGFR/IFNG/IL1B/IL6/NOS2/PPARD/SPP1/SREBF1/TNF | 13 |
| GO:0051235 | maintenance of location | 14/97 | 324/18866 | 1.14E-09 | 1.87E-08 | 7.99E-09 | AKT1/ALB/APOB/CAV1/CRP/F2/IL10/IL1B/IL6/NFKBIA/PPARA/PPARD/PPARG/TNF | 14 |
| GO:0006066 | alcohol metabolic process | 15/97 | 385/18866 | 1.14E-09 | 1.88E-08 | 7.99E-09 | APOB/CAT/CYP1A1/CYP1A2/CYP3A4/IFNG/IL1B/IL4/PON1/PPARD/PTEN/RXRA/SOD1/SREBF1/TNF | 15 |
| GO:0032677 | regulation of interleukin-8 production | Sep-97 | 93/18866 | 1.16E-09 | 1.88E-08 | 8.03E-09 | ADIPOQ/CRP/F3/IL10/IL1B/IL6/SERPINE1/STAT3/TNF | 9 |
| GO:1903035 | negative regulation of response to wounding | Sep-97 | 93/18866 | 1.16E-09 | 1.88E-08 | 8.03E-09 | ALOX5/F2/NOS3/PLAT/PTEN/SERPINE1/SPP1/THBD/TNF | 9 |
| GO:0030856 | regulation of epithelial cell differentiation | Nov-97 | 171/18866 | 1.25E-09 | 2.02E-08 | 8.59E-09 | ADIPOQ/CAV1/CTNNB1/IFNG/IKBKB/IL1B/MMP9/SERPINE1/TNF/VEGFA/XDH | 11 |
| GO:0002064 | epithelial cell development | Dec-97 | 221/18866 | 1.47E-09 | 2.37E-08 | 1.01E-08 | ADIPOQ/AR/CDKN1A/CTNNB1/ESR1/ICAM1/IKBKB/IL1B/PGR/SOD1/TNF/VEGFA | 12 |
| GO:0001659 | temperature homeostasis | Nov-97 | 174/18866 | 1.50E-09 | 2.39E-08 | 1.02E-08 | ACHE/ADIPOQ/CAV1/IL1A/IL1B/IL4/PTGER3/PTGS2/STAT3/TNF/VEGFA | 11 |
| GO:0097696 | receptor signaling pathway via STAT | Nov-97 | 174/18866 | 1.50E-09 | 2.39E-08 | 1.02E-08 | CAV1/CCL2/EGF/F2/IFNG/IL10/IL2/IL4/IL6/STAT3/TNF | 11 |
| GO:0006869 | lipid transport | 15/97 | 393/18866 | 1.51E-09 | 2.41E-08 | 1.03E-08 | ADIPOQ/AKT1/APOB/CAV1/CYP19A1/EGF/IL1B/NFKBIA/NOS2/PON1/PPARA/PPARD/PPARG/RXRA/SPP1 | 15 |
| GO:0097529 | myeloid leukocyte migration | Dec-97 | 222/18866 | 1.55E-09 | 2.45E-08 | 1.05E-08 | CCL2/CXCL8/CYP19A1/DPP4/IL1B/IL4/IL6/MAPK1/MAPK3/PIK3CG/SERPINE1/VEGFA | 12 |
| GO:0032768 | regulation of monooxygenase activity | Aug-97 | 66/18866 | 1.64E-09 | 2.58E-08 | 1.10E-08 | AKT1/CAV1/EGFR/IFNG/IL1A/IL1B/NOS3/TNF | 8 |
| GO:0009306 | protein secretion | 16/97 | 462/18866 | 1.66E-09 | 2.61E-08 | 1.11E-08 | ACHE/ALOX5/CD40LG/DPP4/EGFR/IFNG/IL10/IL1A/IL1B/IL2/IL6/NOS2/PPARD/SREBF1/TGFB1/TNF | 16 |
| GO:0035592 | establishment of protein localization to extracellular region | 16/97 | 463/18866 | 1.71E-09 | 2.68E-08 | 1.14E-08 | ACHE/ALOX5/CD40LG/DPP4/EGFR/IFNG/IL10/IL1A/IL1B/IL2/IL6/NOS2/PPARD/SREBF1/TGFB1/TNF | 16 |
| GO:0032368 | regulation of lipid transport | Oct-97 | 135/18866 | 1.86E-09 | 2.90E-08 | 1.24E-08 | ADIPOQ/AKT1/CAV1/CYP19A1/EGF/IL1B/NFKBIA/PON1/PPARG/SPP1 | 10 |
| GO:0048771 | tissue remodeling | Nov-97 | 178/18866 | 1.90E-09 | 2.94E-08 | 1.25E-08 | CAV1/CTNNB1/EGFR/IL1A/IL2/IL6/MMP2/NOS3/SPP1/TGFB1/TP53 | 11 |
| GO:0051897 | positive regulation of protein kinase B signaling | Nov-97 | 178/18866 | 1.90E-09 | 2.94E-08 | 1.25E-08 | EGF/EGFR/ERBB2/ERBB3/ESR1/F10/F3/F7/PIK3CG/TGFB1/TNF | 11 |
| GO:1903829 | positive regulation of cellular protein localization | 14/97 | 338/18866 | 1.96E-09 | 3.02E-08 | 1.29E-08 | AKT1/BCL2/EGF/EGFR/ERBB2/F2/IFNG/IL1B/MAPK1/MAPK8/PTGS2/TGFB1/TNF/TP53 | 14 |
| GO:0060249 | anatomical structure homeostasis | 16/97 | 469/18866 | 2.06E-09 | 3.16E-08 | 1.35E-08 | ALB/BCL2/CTNNB1/EGFR/HSPB1/IL6/LYZ/MAPK1/MAPK3/MYC/NOS3/PTGS2/RHO/SOD1/SPP1/VEGFA | 16 |
| GO:0045428 | regulation of nitric oxide biosynthetic process | Aug-97 | 68/18866 | 2.09E-09 | 3.19E-08 | 1.36E-08 | AKT1/CAV1/ICAM1/IFNG/IL10/IL1B/PTGS2/TNF | 8 |
| GO:0150077 | regulation of neuroinflammatory response | Jul-97 | 43/18866 | 2.11E-09 | 3.20E-08 | 1.37E-08 | IL1B/IL4/IL6/MMP3/MMP9/PTGS2/TNF | 7 |
| GO:0051047 | positive regulation of secretion | 14/97 | 340/18866 | 2.12E-09 | 3.20E-08 | 1.37E-08 | ACHE/CYP19A1/EGFR/IFNG/IL10/IL1A/IL1B/IL2/IL4/IL6/PPARD/SPP1/TGFB1/TNF | 14 |
| GO:0071692 | protein localization to extracellular region | 16/97 | 470/18866 | 2.12E-09 | 3.20E-08 | 1.37E-08 | ACHE/ALOX5/CD40LG/DPP4/EGFR/IFNG/IL10/IL1A/IL1B/IL2/IL6/NOS2/PPARD/SREBF1/TGFB1/TNF | 16 |
| GO:0019217 | regulation of fatty acid metabolic process | Sep-97 | 101/18866 | 2.43E-09 | 3.65E-08 | 1.56E-08 | ADIPOQ/AKT1/CAV1/IL1B/PPARA/PPARD/PPARG/PTGS2/SREBF1 | 9 |
| GO:0051403 | stress-activated MAPK cascade | 13/97 | 286/18866 | 2.54E-09 | 3.80E-08 | 1.62E-08 | AKT1/CD40LG/EGFR/GSTP1/IKBKB/IL1B/MAPK1/MAPK3/MAPK8/MYC/TNF/VEGFA/XDH | 13 |
| GO:0009266 | response to temperature stimulus | Dec-97 | 233/18866 | 2.68E-09 | 3.99E-08 | 1.70E-08 | AKT1/CDKN1A/FOS/HMOX1/IL1A/MAPK1/MAPK3/NFKBIA/NOS3/PPARG/PTGS2/SOD1 | 12 |
| GO:0098869 | cellular oxidant detoxification | Sep-97 | 103/18866 | 2.90E-09 | 4.30E-08 | 1.83E-08 | ALB/CAT/GSR/GSTP1/MPO/NOS3/PTGS2/SOD1/TNF | 9 |
| GO:0071453 | cellular response to oxygen levels | Dec-97 | 235/18866 | 2.95E-09 | 4.33E-08 | 1.85E-08 | AKT1/BCL2/CAV1/HMOX1/ICAM1/MYC/PPARD/PPARG/PTEN/PTGS2/TP53/VEGFA | 12 |
| GO:1904018 | positive regulation of vasculature development | Dec-97 | 235/18866 | 2.95E-09 | 4.33E-08 | 1.85E-08 | CXCL8/F3/HMOX1/HSPB1/IL10/IL1A/IL1B/NOS3/PTGS2/SERPINE1/STAT3/VEGFA | 12 |
| GO:0001667 | ameboidal-type cell migration | 16/97 | 481/18866 | 2.96E-09 | 4.33E-08 | 1.85E-08 | AKT1/DPP4/HMOX1/HSPB1/IFNG/IL4/JUN/MMP9/NOS3/PPARD/PPARG/PTEN/PTGS2/TGFB1/TNF/VEGFA | 16 |
| GO:0097193 | intrinsic apoptotic signaling pathway | 13/97 | 290/18866 | 3.01E-09 | 4.39E-08 | 1.87E-08 | AKT1/BCL2/BCL2L1/CASP3/CAV1/CDKN1A/HMOX1/HSPB1/MMP9/PTGS2/SOD1/TNF/TP53 | 13 |
| GO:0030879 | mammary gland development | Oct-97 | 142/18866 | 3.05E-09 | 4.42E-08 | 1.89E-08 | AKT1/AR/CAV1/CYP19A1/EGF/ESR1/MAPK1/PGR/VEGFA/XDH | 10 |
| GO:0046425 | regulation of receptor signaling pathway via JAK-STAT | Oct-97 | 142/18866 | 3.05E-09 | 4.42E-08 | 1.89E-08 | CAV1/EGF/F2/IFNG/IL10/IL2/IL4/IL6/STAT3/TNF | 10 |
| GO:0051051 | negative regulation of transport | 16/97 | 483/18866 | 3.14E-09 | 4.53E-08 | 1.93E-08 | ADIPOQ/AKT1/BCL2/CAV1/EGF/ERBB3/HMOX1/ICAM1/IL1B/MMP9/NOS3/PTEN/PTGER3/PTGS2/SREBF1/TNF | 16 |
| GO:0061138 | morphogenesis of a branching epithelium | Nov-97 | 187/18866 | 3.20E-09 | 4.61E-08 | 1.96E-08 | AR/BCL2/CTNNB1/EGF/ESR1/IL10/MYC/PGR/RXRA/TNF/VEGFA | 11 |
| GO:0035296 | regulation of tube diameter | Oct-97 | 143/18866 | 3.27E-09 | 4.64E-08 | 1.98E-08 | AKT1/CAV1/CRP/EGFR/HMOX1/ICAM1/NOS3/PPARD/PTGS2/SOD1 | 10 |
| GO:0045598 | regulation of fat cell differentiation | Oct-97 | 143/18866 | 3.27E-09 | 4.64E-08 | 1.98E-08 | ADIPOQ/AKT1/ALOX5/IL6/PIM1/PPARD/PPARG/PTGS2/TGFB1/TNF | 10 |
| GO:0097746 | regulation of blood vessel diameter | Oct-97 | 143/18866 | 3.27E-09 | 4.64E-08 | 1.98E-08 | AKT1/CAV1/CRP/EGFR/HMOX1/ICAM1/NOS3/PPARD/PTGS2/SOD1 | 10 |
| GO:0035150 | regulation of tube size | Oct-97 | 144/18866 | 3.50E-09 | 4.95E-08 | 2.11E-08 | AKT1/CAV1/CRP/EGFR/HMOX1/ICAM1/NOS3/PPARD/PTGS2/SOD1 | 10 |
| GO:0001558 | regulation of cell growth | 15/97 | 420/18866 | 3.73E-09 | 5.26E-08 | 2.24E-08 | AKT1/BCL2/CDKN1A/EGFR/ERBB2/F2/IGFBP3/IL2/PPARA/PPARD/PPARG/SPP1/TGFB1/TP53/VEGFA | 15 |
| GO:0032091 | negative regulation of protein binding | Sep-97 | 106/18866 | 3.75E-09 | 5.26E-08 | 2.24E-08 | ADIPOQ/AKT1/CAV1/CDKN1A/IL10/MAPK3/MAPK8/PPARA/SLPI | 9 |
| GO:0016049 | cell growth | 16/97 | 490/18866 | 3.85E-09 | 5.39E-08 | 2.30E-08 | AKT1/BCL2/CDKN1A/CTNNB1/EGFR/ERBB2/F2/IGFBP3/IL2/PPARA/PPARD/PPARG/SPP1/TGFB1/TP53/VEGFA | 16 |
| GO:0035265 | organ growth | Nov-97 | 191/18866 | 4.00E-09 | 5.58E-08 | 2.38E-08 | AKT1/AR/BCL2/CYP19A1/ESR1/MAPK1/PIM1/PPARA/PTEN/RXRA/SOD1 | 11 |
| GO:0010745 | negative regulation of macrophage derived foam cell differentiation | May-97 | 13/18866 | 4.03E-09 | 5.60E-08 | 2.39E-08 | ADIPOQ/CRP/NFKBIA/PPARA/PPARG | 5 |
| GO:0031098 | stress-activated protein kinase signaling cascade | 13/97 | 300/18866 | 4.53E-09 | 6.26E-08 | 2.67E-08 | AKT1/CD40LG/EGFR/GSTP1/IKBKB/IL1B/MAPK1/MAPK3/MAPK8/MYC/TNF/VEGFA/XDH | 13 |
| GO:0031100 | animal organ regeneration | Aug-97 | 75/18866 | 4.63E-09 | 6.38E-08 | 2.72E-08 | CDKN1A/EGFR/F7/GSTP1/HMOX1/IL10/IL6/PPARG | 8 |
| GO:0007162 | negative regulation of cell adhesion | 13/97 | 301/18866 | 4.71E-09 | 6.47E-08 | 2.76E-08 | ADIPOQ/AKT1/CASP3/ERBB2/ERBB3/IL10/IL2/IL4/PPARA/PTEN/SERPINE1/TGFB1/VEGFA | 13 |
| GO:0051402 | neuron apoptotic process | Dec-97 | 245/18866 | 4.72E-09 | 6.47E-08 | 2.76E-08 | BCL2/BCL2L1/CASP3/CCL2/CTNNB1/ERBB3/HMOX1/IL10/JUN/SOD1/TNF/TP53 | 12 |
| GO:1905475 | regulation of protein localization to membrane | Nov-97 | 195/18866 | 4.97E-09 | 6.78E-08 | 2.89E-08 | AKT1/AR/BCL2/BCL2L1/EGFR/ERBB2/IFNG/MAPK8/TGFB1/TNF/TP53 | 11 |
| GO:0030099 | myeloid cell differentiation | 15/97 | 431/18866 | 5.29E-09 | 7.19E-08 | 3.07E-08 | ADIPOQ/CASP3/CTNNB1/FOS/IFNG/IL4/JUN/MMP9/MYC/NFKBIA/PPARG/STAT3/TGFB1/TNF/VEGFA | 15 |
| GO:0035196 | production of miRNAs involved in gene silencing by miRNA | Jul-97 | 49/18866 | 5.49E-09 | 7.43E-08 | 3.17E-08 | EGFR/ESR1/IL6/STAT3/TGFB1/TNF/TP53 | 7 |
| GO:1904892 | regulation of receptor signaling pathway via STAT | Oct-97 | 151/18866 | 5.54E-09 | 7.48E-08 | 3.19E-08 | CAV1/EGF/F2/IFNG/IL10/IL2/IL4/IL6/STAT3/TNF | 10 |
| GO:0008203 | cholesterol metabolic process | Oct-97 | 153/18866 | 6.29E-09 | 8.46E-08 | 3.61E-08 | APOB/CAT/CYP1A2/CYP3A4/IL4/PON1/PPARD/RXRA/SOD1/SREBF1 | 10 |
| GO:0061045 | negative regulation of wound healing | Aug-97 | 78/18866 | 6.35E-09 | 8.51E-08 | 3.63E-08 | ALOX5/F2/NOS3/PLAT/PTEN/SERPINE1/THBD/TNF | 8 |
| GO:0032963 | collagen metabolic process | Sep-97 | 113/18866 | 6.61E-09 | 8.82E-08 | 3.76E-08 | F2/IL6/MMP1/MMP2/MMP3/MMP9/PPARD/PPARG/TGFB1 | 9 |
| GO:0030217 | T cell differentiation | Dec-97 | 253/18866 | 6.78E-09 | 9.01E-08 | 3.84E-08 | BCL2/CTNNB1/ERBB2/IFNG/IL1A/IL1B/IL2/IL4/IL6/SOD1/STAT3/TP53 | 12 |
| GO:0001763 | morphogenesis of a branching structure | Nov-97 | 201/18866 | 6.83E-09 | 9.05E-08 | 3.86E-08 | AR/BCL2/CTNNB1/EGF/ESR1/IL10/MYC/PGR/RXRA/TNF/VEGFA | 11 |
| GO:0046879 | hormone secretion | 13/97 | 314/18866 | 7.82E-09 | 1.03E-07 | 4.40E-08 | ADIPOQ/ALOX5/CYP19A1/DPP4/EGFR/IFNG/IL1B/IL6/NOS2/PPARD/SPP1/SREBF1/TNF | 13 |
| GO:0050999 | regulation of nitric-oxide synthase activity | Jul-97 | 52/18866 | 8.44E-09 | 1.11E-07 | 4.74E-08 | AKT1/CAV1/EGFR/IL1A/IL1B/NOS3/TNF | 7 |
| GO:0043434 | response to peptide hormone | 15/97 | 447/18866 | 8.63E-09 | 1.13E-07 | 4.83E-08 | ADIPOQ/AKT1/CAT/CAV1/F7/GSTP1/ICAM1/IL10/IL1B/PPARA/PPARG/PTEN/PTGS2/SREBF1/STAT3 | 15 |
| GO:0042594 | response to starvation | Nov-97 | 206/18866 | 8.82E-09 | 1.15E-07 | 4.90E-08 | ALB/BCL2/CDKN1A/JUN/MAPK1/MAPK3/MAPK8/PPARA/PPARG/SREBF1/TP53 | 11 |
| GO:0071383 | cellular response to steroid hormone stimulus | Nov-97 | 206/18866 | 8.82E-09 | 1.15E-07 | 4.90E-08 | AR/EGFR/ESR1/GSTP1/ICAM1/NR3C1/NR3C2/PGR/PPARA/PPARD/RXRA | 11 |
| GO:0009416 | response to light stimulus | 13/97 | 319/18866 | 9.44E-09 | 1.22E-07 | 5.22E-08 | AKT1/BCL2/CASP3/CAT/CDKN1A/EGFR/FOS/MAPK8/MYC/PTGS2/RHO/TP53/TYR | 13 |
| GO:0030193 | regulation of blood coagulation | Aug-97 | 82/18866 | 9.50E-09 | 1.23E-07 | 5.23E-08 | CAV1/F2/F3/F7/NOS3/PLAT/SERPINE1/THBD | 8 |
| GO:0009612 | response to mechanical stimulus | Nov-97 | 209/18866 | 1.03E-08 | 1.32E-07 | 5.63E-08 | AKT1/EGFR/FOS/IL1B/JUN/MAPK3/MAPK8/MPO/NFKBIA/PPARG/PTGS2 | 11 |
| GO:0031349 | positive regulation of defense response | 14/97 | 385/18866 | 1.03E-08 | 1.33E-07 | 5.66E-08 | EGFR/IFNG/IKBKB/IL1B/IL2/IL6/MAPK3/NFKBIA/PIK3CG/PTGER3/PTGS2/SERPINE1/STAT3/TNF | 14 |
| GO:1900046 | regulation of hemostasis | Aug-97 | 83/18866 | 1.05E-08 | 1.34E-07 | 5.70E-08 | CAV1/F2/F3/F7/NOS3/PLAT/SERPINE1/THBD | 8 |
| GO:0002675 | positive regulation of acute inflammatory response | Jun-97 | 31/18866 | 1.05E-08 | 1.34E-07 | 5.70E-08 | IL1B/IL6/PIK3CG/PTGER3/PTGS2/TNF | 6 |
| GO:0043406 | positive regulation of MAP kinase activity | Dec-97 | 264/18866 | 1.09E-08 | 1.38E-07 | 5.89E-08 | CD40LG/EGF/EGFR/ERBB2/IL1B/MAPK1/MAPK3/PIK3CG/SOD1/TGFB1/TNF/VEGFA | 12 |
| GO:1902652 | secondary alcohol metabolic process | Oct-97 | 162/18866 | 1.09E-08 | 1.38E-07 | 5.89E-08 | APOB/CAT/CYP1A2/CYP3A4/IL4/PON1/PPARD/RXRA/SOD1/SREBF1 | 10 |
| GO:0009914 | hormone transport | 13/97 | 323/18866 | 1.09E-08 | 1.38E-07 | 5.89E-08 | ADIPOQ/ALOX5/CYP19A1/DPP4/EGFR/IFNG/IL1B/IL6/NOS2/PPARD/SPP1/SREBF1/TNF | 13 |
| GO:0031050 | dsRNA processing | Jul-97 | 54/18866 | 1.11E-08 | 1.39E-07 | 5.91E-08 | EGFR/ESR1/IL6/STAT3/TGFB1/TNF/TP53 | 7 |
| GO:0032757 | positive regulation of interleukin-8 production | Jul-97 | 54/18866 | 1.11E-08 | 1.39E-07 | 5.91E-08 | ADIPOQ/F3/IL1B/IL6/SERPINE1/STAT3/TNF | 7 |
| GO:0070918 | production of small RNA involved in gene silencing by RNA | Jul-97 | 54/18866 | 1.11E-08 | 1.39E-07 | 5.91E-08 | EGFR/ESR1/IL6/STAT3/TGFB1/TNF/TP53 | 7 |
| GO:0009267 | cellular response to starvation | Oct-97 | 163/18866 | 1.16E-08 | 1.44E-07 | 6.15E-08 | ALB/BCL2/CDKN1A/JUN/MAPK1/MAPK3/MAPK8/PPARA/SREBF1/TP53 | 10 |
| GO:0010743 | regulation of macrophage derived foam cell differentiation | Jun-97 | 32/18866 | 1.28E-08 | 1.59E-07 | 6.80E-08 | ADIPOQ/APOB/CRP/NFKBIA/PPARA/PPARG | 6 |
| GO:0006690 | icosanoid metabolic process | Sep-97 | 122/18866 | 1.30E-08 | 1.60E-07 | 6.84E-08 | ALOX5/CYP1A1/CYP1A2/GSTM1/GSTP1/IL1B/MAPK3/PON1/PTGS2 | 9 |
| GO:0021782 | glial cell development | Sep-97 | 122/18866 | 1.30E-08 | 1.60E-07 | 6.84E-08 | AKT1/EGFR/GSTP1/IFNG/IL1B/IL6/PTEN/SOD1/TNF | 9 |
| GO:0031348 | negative regulation of defense response | Dec-97 | 269/18866 | 1.34E-08 | 1.65E-07 | 7.04E-08 | ADIPOQ/ALOX5/CYP19A1/F2/GSTP1/IL10/IL2/IL4/PPARA/PPARD/PPARG/SOD1 | 12 |
| GO:0002761 | regulation of myeloid leukocyte differentiation | Sep-97 | 123/18866 | 1.40E-08 | 1.71E-07 | 7.30E-08 | ADIPOQ/CTNNB1/FOS/IFNG/IL4/JUN/MYC/TGFB1/TNF | 9 |
| GO:0001655 | urogenital system development | 13/97 | 330/18866 | 1.41E-08 | 1.72E-07 | 7.35E-08 | ADIPOQ/AR/BCL2/CAT/CTNNB1/CYP19A1/ESR1/MMP9/MYC/ODC1/PTEN/RXRA/VEGFA | 13 |
| GO:0071214 | cellular response to abiotic stimulus | 13/97 | 331/18866 | 1.46E-08 | 1.78E-07 | 7.57E-08 | AKT1/BCL2L1/CASP3/CDKN1A/EGFR/IL1B/MAPK3/MAPK8/MYC/PTEN/PTGS2/RHO/TP53 | 13 |
| GO:0104004 | cellular response to environmental stimulus | 13/97 | 331/18866 | 1.46E-08 | 1.78E-07 | 7.57E-08 | AKT1/BCL2L1/CASP3/CDKN1A/EGFR/IL1B/MAPK3/MAPK8/MYC/PTEN/PTGS2/RHO/TP53 | 13 |
| GO:0052547 | regulation of peptidase activity | 15/97 | 466/18866 | 1.50E-08 | 1.82E-07 | 7.75E-08 | AKT1/BIRC5/CAV1/F3/MAPK3/MMP9/MYC/PPARG/PTGS2/SERPINE1/SLPI/STAT3/TNF/VEGFA/XDH | 15 |
| GO:0050818 | regulation of coagulation | Aug-97 | 88/18866 | 1.67E-08 | 2.01E-07 | 8.58E-08 | CAV1/F2/F3/F7/NOS3/PLAT/SERPINE1/THBD | 8 |
| GO:0008625 | extrinsic apoptotic signaling pathway via death domain receptors | Aug-97 | 89/18866 | 1.83E-08 | 2.19E-07 | 9.36E-08 | BCL2/BCL2L1/HMOX1/ICAM1/NOS3/PTEN/SERPINE1/TNF | 8 |
| GO:0031649 | heat generation | May-97 | 17/18866 | 1.91E-08 | 2.28E-07 | 9.74E-08 | IL1A/IL1B/PTGER3/PTGS2/TNF | 5 |
| GO:0050714 | positive regulation of protein secretion | Oct-97 | 172/18866 | 1.94E-08 | 2.31E-07 | 9.86E-08 | ACHE/EGFR/IFNG/IL10/IL1A/IL2/IL6/PPARD/TGFB1/TNF | 10 |
| GO:0046427 | positive regulation of receptor signaling pathway via JAK-STAT | Aug-97 | 90/18866 | 2.00E-08 | 2.37E-07 | 1.01E-07 | F2/IFNG/IL10/IL2/IL4/IL6/STAT3/TNF | 8 |
| GO:0070542 | response to fatty acid | Aug-97 | 90/18866 | 2.00E-08 | 2.37E-07 | 1.01E-07 | ADIPOQ/AKT1/APOB/CAT/PON1/PPARG/PTGS2/SREBF1 | 8 |
| GO:0008406 | gonad development | Nov-97 | 223/18866 | 2.01E-08 | 2.37E-07 | 1.01E-07 | AR/BCL2/BCL2L1/CASP3/CYP19A1/ESR1/ICAM1/NOS3/PGR/SOD1/VEGFA | 11 |
| GO:0007548 | sex differentiation | Dec-97 | 280/18866 | 2.10E-08 | 2.47E-07 | 1.05E-07 | AR/BCL2/BCL2L1/CASP3/CTNNB1/CYP19A1/ESR1/ICAM1/NOS3/PGR/SOD1/VEGFA | 12 |
| GO:0050680 | negative regulation of epithelial cell proliferation | Oct-97 | 175/18866 | 2.29E-08 | 2.68E-07 | 1.14E-07 | ALOX5/AR/CAV1/CCL2/PPARD/PPARG/PTEN/TGFB1/TNF/XDH | 10 |
| GO:0061900 | glial cell activation | Jul-97 | 60/18866 | 2.36E-08 | 2.76E-07 | 1.18E-07 | EGFR/IFNG/IL1B/IL4/IL6/JUN/TNF | 7 |
| GO:1900407 | regulation of cellular response to oxidative stress | Aug-97 | 92/18866 | 2.38E-08 | 2.78E-07 | 1.18E-07 | AKT1/ALOX5/CTNNB1/HSPB1/IL10/MMP3/SOD1/TNF | 8 |
| GO:0010634 | positive regulation of epithelial cell migration | Oct-97 | 176/18866 | 2.41E-08 | 2.81E-07 | 1.20E-07 | AKT1/HMOX1/HSPB1/IFNG/JUN/MMP9/NOS3/PTGS2/TGFB1/VEGFA | 10 |
| GO:0030324 | lung development | Oct-97 | 177/18866 | 2.55E-08 | 2.95E-07 | 1.26E-07 | CTNNB1/CYP1A2/EGFR/MAPK1/MAPK3/NOS3/PGR/SREBF1/TNF/VEGFA | 10 |
| GO:0010506 | regulation of autophagy | 13/97 | 347/18866 | 2.56E-08 | 2.96E-07 | 1.26E-07 | AKT1/BCL2/CASP3/HMOX1/HSPB1/IFNG/IL10/IL4/MAPK3/MAPK8/SREBF1/STAT3/TP53 | 13 |
| GO:1904894 | positive regulation of receptor signaling pathway via STAT | Aug-97 | 93/18866 | 2.59E-08 | 2.98E-07 | 1.27E-07 | F2/IFNG/IL10/IL2/IL4/IL6/STAT3/TNF | 8 |
| GO:0001101 | response to acid chemical | Sep-97 | 132/18866 | 2.59E-08 | 2.98E-07 | 1.27E-07 | BCL2L1/CASP3/EGFR/F7/GSTP1/ICAM1/MMP2/TNF/VEGFA | 9 |
| GO:0045137 | development of primary sexual characteristics | Nov-97 | 229/18866 | 2.64E-08 | 3.02E-07 | 1.29E-07 | AR/BCL2/BCL2L1/CASP3/CYP19A1/ESR1/ICAM1/NOS3/PGR/SOD1/VEGFA | 11 |
| GO:1901099 | negative regulation of signal transduction in absence of ligand | Jun-97 | 36/18866 | 2.72E-08 | 3.09E-07 | 1.32E-07 | AKT1/BCL2/BCL2L1/IL1A/IL1B/TNF | 6 |
| GO:2001240 | negative regulation of extrinsic apoptotic signaling pathway in absence of ligand | Jun-97 | 36/18866 | 2.72E-08 | 3.09E-07 | 1.32E-07 | AKT1/BCL2/BCL2L1/IL1A/IL1B/TNF | 6 |
| GO:1904035 | regulation of epithelial cell apoptotic process | Aug-97 | 94/18866 | 2.82E-08 | 3.19E-07 | 1.36E-07 | CCL2/CD40LG/HMOX1/ICAM1/IL4/IL6/SERPINE1/TNF | 8 |
| GO:1905954 | positive regulation of lipid localization | Aug-97 | 94/18866 | 2.82E-08 | 3.19E-07 | 1.36E-07 | ADIPOQ/APOB/CAV1/CYP19A1/IL1B/NFKBIA/PON1/SPP1 | 8 |
| GO:0030888 | regulation of B cell proliferation | Jul-97 | 62/18866 | 2.98E-08 | 3.36E-07 | 1.43E-07 | AHR/BCL2/CASP3/CDKN1A/IL10/IL2/IL4 | 7 |
| GO:0048638 | regulation of developmental growth | 13/97 | 353/18866 | 3.13E-08 | 3.52E-07 | 1.50E-07 | AKT1/AR/BCL2/CDKN1A/MAPK1/PIM1/PPARA/PPARD/PTEN/SOD1/SPP1/STAT3/VEGFA | 13 |
| GO:0030323 | respiratory tube development | Oct-97 | 181/18866 | 3.15E-08 | 3.52E-07 | 1.50E-07 | CTNNB1/CYP1A2/EGFR/MAPK1/MAPK3/NOS3/PGR/SREBF1/TNF/VEGFA | 10 |
| GO:0009743 | response to carbohydrate | Nov-97 | 233/18866 | 3.15E-08 | 3.52E-07 | 1.50E-07 | ADIPOQ/APOB/CASP3/CAT/GSTP1/ICAM1/IL1B/PPARD/PTEN/PTGS2/SREBF1 | 11 |
| GO:0090322 | regulation of superoxide metabolic process | Jun-97 | 37/18866 | 3.23E-08 | 3.60E-07 | 1.53E-07 | CRP/EGFR/GSTP1/SOD1/TGFB1/TNF | 6 |
| GO:0002690 | positive regulation of leukocyte chemotaxis | Aug-97 | 96/18866 | 3.33E-08 | 3.70E-07 | 1.58E-07 | CXCL8/F7/IL4/IL6/MAPK1/MAPK3/SERPINE1/VEGFA | 8 |
| GO:0050671 | positive regulation of lymphocyte proliferation | Sep-97 | 136/18866 | 3.37E-08 | 3.73E-07 | 1.59E-07 | BCL2/CD40LG/CDKN1A/IL1A/IL1B/IL2/IL4/IL6/VCAM1 | 9 |
| GO:0045444 | fat cell differentiation | Nov-97 | 235/18866 | 3.44E-08 | 3.80E-07 | 1.62E-07 | ADIPOQ/AKT1/ALOX5/IL6/PIM1/PPARD/PPARG/PTGS2/SREBF1/TGFB1/TNF | 11 |
| GO:0032930 | positive regulation of superoxide anion generation | May-97 | 19/18866 | 3.56E-08 | 3.92E-07 | 1.67E-07 | CRP/EGFR/GSTP1/SOD1/TGFB1 | 5 |
| GO:0032946 | positive regulation of mononuclear cell proliferation | Sep-97 | 137/18866 | 3.59E-08 | 3.94E-07 | 1.68E-07 | BCL2/CD40LG/CDKN1A/IL1A/IL1B/IL2/IL4/IL6/VCAM1 | 9 |
| GO:0042100 | B cell proliferation | Aug-97 | 97/18866 | 3.62E-08 | 3.94E-07 | 1.68E-07 | AHR/BCL2/CASP3/CD40LG/CDKN1A/IL10/IL2/IL4 | 8 |
| GO:0050764 | regulation of phagocytosis | Aug-97 | 97/18866 | 3.62E-08 | 3.94E-07 | 1.68E-07 | ADIPOQ/CCL2/IFNG/IL1B/PPARG/PTEN/SOD1/TNF | 8 |
| GO:1901655 | cellular response to ketone | Aug-97 | 97/18866 | 3.62E-08 | 3.94E-07 | 1.68E-07 | AHR/AKT1/AR/EGFR/ICAM1/NR3C1/PPARG/SPP1 | 8 |
| GO:0032872 | regulation of stress-activated MAPK cascade | Nov-97 | 237/18866 | 3.75E-08 | 4.07E-07 | 1.74E-07 | AKT1/CD40LG/EGFR/GSTP1/IL1B/MAPK1/MAPK3/MYC/TNF/VEGFA/XDH | 11 |
| GO:0045923 | positive regulation of fatty acid metabolic process | Jun-97 | 38/18866 | 3.82E-08 | 4.13E-07 | 1.76E-07 | ADIPOQ/IL1B/PPARA/PPARD/PPARG/PTGS2 | 6 |
| GO:0051348 | negative regulation of transferase activity | Dec-97 | 296/18866 | 3.87E-08 | 4.18E-07 | 1.78E-07 | ADIPOQ/AKT1/CASP3/CAV1/CDKN1A/GSTP1/HSPB1/IFNG/IL1B/PPARG/PTEN/TP53 | 12 |
| GO:0070302 | regulation of stress-activated protein kinase signaling cascade | Nov-97 | 240/18866 | 4.27E-08 | 4.59E-07 | 1.96E-07 | AKT1/CD40LG/EGFR/GSTP1/IL1B/MAPK1/MAPK3/MYC/TNF/VEGFA/XDH | 11 |
| GO:0002285 | lymphocyte activation involved in immune response | Oct-97 | 187/18866 | 4.29E-08 | 4.60E-07 | 1.96E-07 | CD40LG/ICAM1/IFNG/IL10/IL2/IL4/IL6/STAT3/TGFB1/TP53 | 10 |
| GO:0010565 | regulation of cellular ketone metabolic process | Oct-97 | 189/18866 | 4.74E-08 | 5.08E-07 | 2.16E-07 | ADIPOQ/AKT1/CAV1/IL1B/ODC1/PPARA/PPARD/PPARG/PTGS2/SREBF1 | 10 |
| GO:1902882 | regulation of response to oxidative stress | Aug-97 | 101/18866 | 4.97E-08 | 5.30E-07 | 2.26E-07 | AKT1/ALOX5/CTNNB1/HSPB1/IL10/MMP3/SOD1/TNF | 8 |
| GO:0022408 | negative regulation of cell-cell adhesion | Oct-97 | 191/18866 | 5.24E-08 | 5.57E-07 | 2.38E-07 | ADIPOQ/AKT1/CASP3/ERBB2/IL10/IL2/IL4/PPARA/TGFB1/VEGFA | 10 |
| GO:0045862 | positive regulation of proteolysis | 13/97 | 370/18866 | 5.43E-08 | 5.76E-07 | 2.45E-07 | AKT1/CAV1/EGF/F3/IFNG/IL1B/MAPK3/MYC/PPARG/PTEN/STAT3/TNF/XDH | 13 |
| GO:0002700 | regulation of production of molecular mediator of immune response | Sep-97 | 144/18866 | 5.53E-08 | 5.85E-07 | 2.49E-07 | CD40LG/HMOX1/IL10/IL1B/IL2/IL4/IL6/TGFB1/TNF | 9 |
| GO:0070372 | regulation of ERK1 and ERK2 cascade | Dec-97 | 306/18866 | 5.57E-08 | 5.88E-07 | 2.51E-07 | ADIPOQ/CCL2/EGFR/ERBB2/GSTP1/ICAM1/IL1B/JUN/MAPK3/PTEN/TGFB1/TNF | 12 |
| GO:0002637 | regulation of immunoglobulin production | Jul-97 | 68/18866 | 5.72E-08 | 6.00E-07 | 2.56E-07 | CD40LG/IL10/IL2/IL4/IL6/TGFB1/TNF | 7 |
| GO:0072577 | endothelial cell apoptotic process | Jul-97 | 68/18866 | 5.72E-08 | 6.00E-07 | 2.56E-07 | CCL2/CD40LG/ICAM1/IL10/IL4/SERPINE1/TNF | 7 |
| GO:0002793 | positive regulation of peptide secretion | Oct-97 | 193/18866 | 5.78E-08 | 6.05E-07 | 2.58E-07 | ACHE/EGFR/IFNG/IL10/IL1A/IL2/IL6/PPARD/TGFB1/TNF | 10 |
| GO:0071901 | negative regulation of protein serine/threonine kinase activity | Sep-97 | 148/18866 | 7.00E-08 | 7.28E-07 | 3.10E-07 | ADIPOQ/AKT1/CASP3/CAV1/CDKN1A/GSTP1/HSPB1/IL1B/PTEN | 9 |
| GO:0006694 | steroid biosynthetic process | Oct-97 | 197/18866 | 7.01E-08 | 7.28E-07 | 3.10E-07 | APOB/CYP19A1/CYP1A1/CYP3A4/HSD3B1/IFNG/IL1B/SOD1/SREBF1/TNF | 10 |
| GO:0032370 | positive regulation of lipid transport | Jul-97 | 70/18866 | 7.02E-08 | 7.28E-07 | 3.10E-07 | ADIPOQ/CAV1/CYP19A1/IL1B/NFKBIA/PON1/SPP1 | 7 |
| GO:0006606 | protein import into nucleus | Sep-97 | 150/18866 | 7.86E-08 | 8.13E-07 | 3.47E-07 | AKT1/CDKN1A/IFNG/MAPK1/NFKBIA/PTGS2/STAT3/TGFB1/TP53 | 9 |
| GO:0032928 | regulation of superoxide anion generation | May-97 | 22/18866 | 7.96E-08 | 8.16E-07 | 3.48E-07 | CRP/EGFR/GSTP1/SOD1/TGFB1 | 5 |
| GO:0042359 | vitamin D metabolic process | May-97 | 22/18866 | 7.96E-08 | 8.16E-07 | 3.48E-07 | CYP1A1/CYP3A4/IFNG/IL1B/TNF | 5 |
| GO:0055093 | response to hyperoxia | May-97 | 22/18866 | 7.96E-08 | 8.16E-07 | 3.48E-07 | CAT/CAV1/CDKN1A/CYP1A1/PPARG | 5 |
| GO:0051054 | positive regulation of DNA metabolic process | Oct-97 | 200/18866 | 8.08E-08 | 8.27E-07 | 3.52E-07 | AKT1/CTNNB1/EGFR/IL2/IL4/IL6/MAPK1/MAPK3/MYC/TGFB1 | 10 |
| GO:2000278 | regulation of DNA biosynthetic process | Aug-97 | 109/18866 | 9.04E-08 | 9.22E-07 | 3.93E-07 | ADIPOQ/CDKN1A/CTNNB1/MAPK1/MAPK3/MYC/PPARG/TP53 | 8 |
| GO:0042180 | cellular ketone metabolic process | Nov-97 | 260/18866 | 9.64E-08 | 9.80E-07 | 4.18E-07 | ADIPOQ/AKT1/CAV1/CYP19A1/IL1B/ODC1/PPARA/PPARD/PPARG/PTGS2/SREBF1 | 11 |
| GO:0060541 | respiratory system development | Oct-97 | 204/18866 | 9.73E-08 | 9.85E-07 | 4.20E-07 | CTNNB1/CYP1A2/EGFR/MAPK1/MAPK3/NOS3/PGR/SREBF1/TNF/VEGFA | 10 |
| GO:1903708 | positive regulation of hemopoiesis | Oct-97 | 204/18866 | 9.73E-08 | 9.85E-07 | 4.20E-07 | FOS/IFNG/IL1A/IL1B/IL2/IL4/JUN/STAT3/TGFB1/TNF | 10 |
| GO:0009895 | negative regulation of catabolic process | Dec-97 | 323/18866 | 1.00E-07 | 1.01E-06 | 4.32E-07 | AKT1/BCL2/EGFR/HMOX1/IL10/IL1B/NOS2/PIK3CG/PPARA/STAT3/TNF/TP53 | 12 |
| GO:1904996 | positive regulation of leukocyte adhesion to vascular endothelial cell | May-97 | 23/18866 | 1.01E-07 | 1.02E-06 | 4.34E-07 | ALOX5/ICAM1/IL6/SELE/TNF | 5 |
| GO:0048754 | branching morphogenesis of an epithelial tube | Sep-97 | 155/18866 | 1.04E-07 | 1.05E-06 | 4.46E-07 | AR/BCL2/CTNNB1/EGF/ESR1/MYC/PGR/TNF/VEGFA | 9 |
| GO:0045637 | regulation of myeloid cell differentiation | Nov-97 | 263/18866 | 1.08E-07 | 1.08E-06 | 4.62E-07 | ADIPOQ/CTNNB1/FOS/IFNG/IL4/JUN/MYC/NFKBIA/STAT3/TGFB1/TNF | 11 |
| GO:0006775 | fat-soluble vitamin metabolic process | Jun-97 | 45/18866 | 1.09E-07 | 1.09E-06 | 4.63E-07 | CYP1A1/CYP3A4/IFNG/IL1B/PPARD/TNF | 6 |
| GO:0035094 | response to nicotine | Jun-97 | 45/18866 | 1.09E-07 | 1.09E-06 | 4.63E-07 | BCL2/CASP3/HMOX1/MAPK1/PPARA/TNF | 6 |
| GO:0045429 | positive regulation of nitric oxide biosynthetic process | Jun-97 | 45/18866 | 1.09E-07 | 1.09E-06 | 4.63E-07 | AKT1/ICAM1/IFNG/IL1B/PTGS2/TNF | 6 |
| GO:0033157 | regulation of intracellular protein transport | Nov-97 | 264/18866 | 1.12E-07 | 1.11E-06 | 4.75E-07 | ADIPOQ/BCL2/ERBB2/IFNG/IL1B/MAPK1/MAPK8/PTGS2/SREBF1/TGFB1/TP53 | 11 |
| GO:0043627 | response to estrogen | Jul-97 | 75/18866 | 1.14E-07 | 1.12E-06 | 4.79E-07 | AR/CAV1/ESR1/F7/HMOX1/MAPK1/PPARG | 7 |
| GO:0030198 | extracellular matrix organization | 13/97 | 395/18866 | 1.16E-07 | 1.14E-06 | 4.87E-07 | CAV1/DPP4/ICAM1/IL6/MMP1/MMP2/MMP3/MMP9/SERPINE1/SPP1/TGFB1/TNF/VCAM1 | 13 |
| GO:0071456 | cellular response to hypoxia | Oct-97 | 208/18866 | 1.17E-07 | 1.15E-06 | 4.89E-07 | AKT1/BCL2/HMOX1/ICAM1/MYC/PPARD/PTEN/PTGS2/TP53/VEGFA | 10 |
| GO:0042113 | B cell activation | Dec-97 | 328/18866 | 1.19E-07 | 1.16E-06 | 4.95E-07 | AHR/BCL2/CASP3/CD40LG/CDKN1A/IL10/IL2/IL4/IL6/TGFB1/TP53/VCAM1 | 12 |
| GO:0010959 | regulation of metal ion transport | 13/97 | 396/18866 | 1.19E-07 | 1.16E-06 | 4.96E-07 | AKT1/BCL2/CAV1/CCL2/CTNNB1/EGF/F2/ICAM1/IFNG/NOS3/PIK3CG/PTEN/PTGS2 | 13 |
| GO:0043062 | extracellular structure organization | 13/97 | 396/18866 | 1.19E-07 | 1.16E-06 | 4.96E-07 | CAV1/DPP4/ICAM1/IL6/MMP1/MMP2/MMP3/MMP9/SERPINE1/SPP1/TGFB1/TNF/VCAM1 | 13 |
| GO:0001676 | long-chain fatty acid metabolic process | Aug-97 | 113/18866 | 1.20E-07 | 1.16E-06 | 4.96E-07 | ALOX5/CYP1A1/CYP1A2/CYP3A4/GSTM1/GSTP1/MAPK3/PTGS2 | 8 |
| GO:0030168 | platelet activation | Sep-97 | 158/18866 | 1.23E-07 | 1.19E-06 | 5.08E-07 | CD40LG/F2/HSPB1/IL6/MAPK1/MAPK3/NOS3/PIK3CG/THBD | 9 |
| GO:0033077 | T cell differentiation in thymus | Jul-97 | 76/18866 | 1.25E-07 | 1.20E-06 | 5.13E-07 | BCL2/CTNNB1/ERBB2/IL1A/IL1B/SOD1/TP53 | 7 |
| GO:0048538 | thymus development | Jun-97 | 46/18866 | 1.25E-07 | 1.20E-06 | 5.13E-07 | BCL2/CTNNB1/MAPK1/MAPK3/SOD1/TYR | 6 |
| GO:0061028 | establishment of endothelial barrier | Jun-97 | 46/18866 | 1.25E-07 | 1.20E-06 | 5.13E-07 | CTNNB1/ICAM1/IKBKB/IL1B/TNF/VEGFA | 6 |
| GO:1904407 | positive regulation of nitric oxide metabolic process | Jun-97 | 46/18866 | 1.25E-07 | 1.20E-06 | 5.13E-07 | AKT1/ICAM1/IFNG/IL1B/PTGS2/TNF | 6 |
| GO:2000637 | positive regulation of gene silencing by miRNA | May-97 | 24/18866 | 1.27E-07 | 1.22E-06 | 5.20E-07 | EGFR/IL6/STAT3/TGFB1/TP53 | 5 |
| GO:0043200 | response to amino acid | Aug-97 | 114/18866 | 1.28E-07 | 1.22E-06 | 5.22E-07 | BCL2L1/CASP3/EGFR/F7/GSTP1/ICAM1/MMP2/TNF | 8 |
| GO:0051092 | positive regulation of NF-kappaB transcription factor activity | Sep-97 | 159/18866 | 1.30E-07 | 1.23E-06 | 5.25E-07 | AR/CAT/CAV1/CD40LG/ICAM1/IKBKB/IL1B/STAT3/TNF | 9 |
| GO:1902107 | positive regulation of leukocyte differentiation | Sep-97 | 159/18866 | 1.30E-07 | 1.23E-06 | 5.25E-07 | FOS/IFNG/IL1A/IL1B/IL2/IL4/JUN/TGFB1/TNF | 9 |
| GO:0002703 | regulation of leukocyte mediated immunity | Oct-97 | 211/18866 | 1.33E-07 | 1.26E-06 | 5.36E-07 | HMOX1/ICAM1/IL10/IL1B/IL2/IL4/IL6/NOS2/TGFB1/TNF | 10 |
| GO:0043393 | regulation of protein binding | Oct-97 | 211/18866 | 1.33E-07 | 1.26E-06 | 5.36E-07 | ADIPOQ/AKT1/CAV1/CDKN1A/IL10/MAPK3/MAPK8/MMP9/PPARA/SLPI | 10 |
| GO:0070555 | response to interleukin-1 | Oct-97 | 211/18866 | 1.33E-07 | 1.26E-06 | 5.36E-07 | CCL2/CXCL8/ICAM1/IKBKB/IL1A/IL1B/IL6/MAPK3/NFKBIA/SELE | 10 |
| GO:0002676 | regulation of chronic inflammatory response | Apr-97 | 10/18866 | 1.35E-07 | 1.27E-06 | 5.40E-07 | CYP19A1/IL10/IL4/TNF | 4 |
| GO:0070849 | response to epidermal growth factor | Jun-97 | 47/18866 | 1.43E-07 | 1.34E-06 | 5.72E-07 | AKT1/EGFR/ERBB2/GSTP1/MAPK1/MAPK3 | 6 |
| GO:0090276 | regulation of peptide hormone secretion | Oct-97 | 213/18866 | 1.46E-07 | 1.36E-06 | 5.81E-07 | ALOX5/DPP4/EGFR/IFNG/IL1B/IL6/NOS2/PPARD/SREBF1/TNF | 10 |
| GO:0060964 | regulation of gene silencing by miRNA | Aug-97 | 116/18866 | 1.47E-07 | 1.37E-06 | 5.84E-07 | EGFR/ESR1/IL6/PPARG/STAT3/TGFB1/TNF/TP53 | 8 |
| GO:0051341 | regulation of oxidoreductase activity | Aug-97 | 117/18866 | 1.57E-07 | 1.46E-06 | 6.22E-07 | AKT1/CAV1/EGFR/IFNG/IL1A/IL1B/NOS3/TNF | 8 |
| GO:0046697 | decidualization | May-97 | 25/18866 | 1.59E-07 | 1.47E-06 | 6.26E-07 | MAPK1/MAPK3/PPARD/PTGS2/SPP1 | 5 |
| GO:0060148 | positive regulation of posttranscriptional gene silencing | May-97 | 25/18866 | 1.59E-07 | 1.47E-06 | 6.26E-07 | EGFR/IL6/STAT3/TGFB1/TP53 | 5 |
| GO:0043281 | regulation of cysteine-type endopeptidase activity involved in apoptotic process | Oct-97 | 215/18866 | 1.59E-07 | 1.47E-06 | 6.26E-07 | AKT1/BIRC5/F3/MMP9/MYC/PPARG/PTGS2/TNF/VEGFA/XDH | 10 |
| GO:0045601 | regulation of endothelial cell differentiation | Jun-97 | 48/18866 | 1.63E-07 | 1.49E-06 | 6.37E-07 | CTNNB1/IKBKB/IL1B/TNF/VEGFA/XDH | 6 |
| GO:0051972 | regulation of telomerase activity | Jun-97 | 48/18866 | 1.63E-07 | 1.49E-06 | 6.37E-07 | CTNNB1/MAPK1/MAPK3/MYC/PPARG/TP53 | 6 |
| GO:2001239 | regulation of extrinsic apoptotic signaling pathway in absence of ligand | Jun-97 | 48/18866 | 1.63E-07 | 1.49E-06 | 6.37E-07 | AKT1/BCL2/BCL2L1/IL1A/IL1B/TNF | 6 |
| GO:0043536 | positive regulation of blood vessel endothelial cell migration | Jul-97 | 79/18866 | 1.63E-07 | 1.50E-06 | 6.37E-07 | AKT1/HMOX1/HSPB1/NOS3/PTGS2/TGFB1/VEGFA | 7 |
| GO:0033559 | unsaturated fatty acid metabolic process | Aug-97 | 118/18866 | 1.68E-07 | 1.53E-06 | 6.52E-07 | ALOX5/CYP1A1/CYP1A2/GSTM1/GSTP1/IL1B/MAPK3/PTGS2 | 8 |
| GO:0043535 | regulation of blood vessel endothelial cell migration | Sep-97 | 164/18866 | 1.69E-07 | 1.54E-06 | 6.56E-07 | AKT1/HMOX1/HSPB1/NOS3/PPARG/PTGS2/TGFB1/TNF/VEGFA | 9 |
| GO:0036294 | cellular response to decreased oxygen levels | Oct-97 | 218/18866 | 1.81E-07 | 1.64E-06 | 7.00E-07 | AKT1/BCL2/HMOX1/ICAM1/MYC/PPARD/PTEN/PTGS2/TP53/VEGFA | 10 |
| GO:0030850 | prostate gland development | Jun-97 | 49/18866 | 1.85E-07 | 1.67E-06 | 7.14E-07 | AR/CTNNB1/CYP19A1/ESR1/PTEN/RXRA | 6 |
| GO:0009408 | response to heat | Sep-97 | 166/18866 | 1.87E-07 | 1.69E-06 | 7.20E-07 | AKT1/CDKN1A/HMOX1/IL1A/MAPK1/MAPK3/NOS3/PTGS2/SOD1 | 9 |
| GO:2001242 | regulation of intrinsic apoptotic signaling pathway | Sep-97 | 166/18866 | 1.87E-07 | 1.69E-06 | 7.20E-07 | AKT1/BCL2/BCL2L1/CAV1/HSPB1/MMP9/PTGS2/SOD1/TP53 | 9 |
| GO:0060147 | regulation of posttranscriptional gene silencing | Aug-97 | 120/18866 | 1.91E-07 | 1.71E-06 | 7.30E-07 | EGFR/ESR1/IL6/PPARG/STAT3/TGFB1/TNF/TP53 | 8 |
| GO:0060966 | regulation of gene silencing by RNA | Aug-97 | 120/18866 | 1.91E-07 | 1.71E-06 | 7.30E-07 | EGFR/ESR1/IL6/PPARG/STAT3/TGFB1/TNF/TP53 | 8 |
| GO:0010721 | negative regulation of cell development | Dec-97 | 343/18866 | 1.92E-07 | 1.72E-06 | 7.31E-07 | BCL2/CTNNB1/F2/IL1B/IL6/PPARA/PTEN/SPP1/STAT3/TNF/TP53/VEGFA | 12 |
| GO:0016042 | lipid catabolic process | Dec-97 | 343/18866 | 1.92E-07 | 1.72E-06 | 7.31E-07 | ADIPOQ/AKT1/APOB/CYP19A1/CYP1A2/CYP3A4/IL1B/PIK3CG/PPARA/PPARD/SPP1/TNF | 12 |
| GO:0032612 | interleukin-1 production | Aug-97 | 121/18866 | 2.03E-07 | 1.81E-06 | 7.71E-07 | GSTP1/HSPB1/IFNG/IL10/IL1B/IL6/STAT3/TNF | 8 |
| GO:0120254 | olefinic compound metabolic process | Aug-97 | 121/18866 | 2.03E-07 | 1.81E-06 | 7.71E-07 | ALOX5/CYP19A1/CYP1A1/CYP1A2/GSTM1/GSTP1/MAPK3/PTGS2 | 8 |
| GO:0031652 | positive regulation of heat generation | Apr-97 | 11/18866 | 2.11E-07 | 1.86E-06 | 7.95E-07 | IL1B/PTGER3/PTGS2/TNF | 4 |
| GO:1903800 | positive regulation of production of miRNAs involved in gene silencing by miRNA | Apr-97 | 11/18866 | 2.11E-07 | 1.86E-06 | 7.95E-07 | EGFR/IL6/TGFB1/TP53 | 4 |
| GO:0022617 | extracellular matrix disassembly | Jul-97 | 82/18866 | 2.12E-07 | 1.87E-06 | 7.96E-07 | DPP4/IL6/MMP1/MMP2/MMP3/MMP9/TGFB1 | 7 |
| GO:0045471 | response to ethanol | Aug-97 | 122/18866 | 2.17E-07 | 1.91E-06 | 8.14E-07 | ADIPOQ/CAT/GSTP1/ICAM1/IL2/PTEN/SOD1/STAT3 | 8 |
| GO:0032868 | response to insulin | Nov-97 | 283/18866 | 2.26E-07 | 1.99E-06 | 8.47E-07 | ADIPOQ/AKT1/CAT/GSTP1/ICAM1/IL10/IL1B/PPARA/PPARG/PTEN/SREBF1 | 11 |
| GO:0009615 | response to virus | Dec-97 | 349/18866 | 2.32E-07 | 2.03E-06 | 8.65E-07 | APOB/BCL2/BCL2L1/CYP1A1/HSPB1/IFNG/IKBKB/IL1B/IL4/IL6/ODC1/TNF | 12 |
| GO:0048143 | astrocyte activation | May-97 | 27/18866 | 2.39E-07 | 2.09E-06 | 8.90E-07 | EGFR/IFNG/IL1B/IL6/TNF | 5 |
| GO:0051170 | import into nucleus | Sep-97 | 171/18866 | 2.41E-07 | 2.10E-06 | 8.97E-07 | AKT1/CDKN1A/IFNG/MAPK1/NFKBIA/PTGS2/STAT3/TGFB1/TP53 | 9 |
| GO:0007249 | I-kappaB kinase/NF-kappaB signaling | Nov-97 | 286/18866 | 2.51E-07 | 2.18E-06 | 9.30E-07 | ADIPOQ/AKT1/CTNNB1/ESR1/GSTP1/HMOX1/HSPB1/IKBKB/IL1B/NFKBIA/TNF | 11 |
| GO:0043542 | endothelial cell migration | Nov-97 | 286/18866 | 2.51E-07 | 2.18E-06 | 9.30E-07 | AKT1/DPP4/HMOX1/HSPB1/NOS3/PPARG/PTEN/PTGS2/TGFB1/TNF/VEGFA | 11 |
| GO:0006633 | fatty acid biosynthetic process | Sep-97 | 173/18866 | 2.66E-07 | 2.31E-06 | 9.83E-07 | ADIPOQ/ALOX5/CYP1A1/CYP1A2/CYP3A4/GSTM1/GSTP1/IL1B/PTGS2 | 9 |
| GO:0060485 | mesenchyme development | Nov-97 | 290/18866 | 2.89E-07 | 2.49E-06 | 1.06E-06 | BCL2/CTNNB1/ERBB3/IL1B/IL6/MAPK1/MAPK3/MYC/NOS3/PTEN/TGFB1 | 11 |
| GO:0010575 | positive regulation of vascular endothelial growth factor production | May-97 | 28/18866 | 2.90E-07 | 2.49E-06 | 1.06E-06 | IL1A/IL1B/IL6/PTGS2/TGFB1 | 5 |
| GO:0030194 | positive regulation of blood coagulation | May-97 | 28/18866 | 2.90E-07 | 2.49E-06 | 1.06E-06 | F2/F3/F7/SERPINE1/THBD | 5 |
| GO:1900048 | positive regulation of hemostasis | May-97 | 28/18866 | 2.90E-07 | 2.49E-06 | 1.06E-06 | F2/F3/F7/SERPINE1/THBD | 5 |
| GO:1901568 | fatty acid derivative metabolic process | Sep-97 | 175/18866 | 2.94E-07 | 2.51E-06 | 1.07E-06 | ALOX5/CYP1A1/CYP1A2/GSTM1/GSTP1/IL1B/MAPK3/PON1/PTGS2 | 9 |
| GO:0042509 | regulation of tyrosine phosphorylation of STAT protein | Jul-97 | 86/18866 | 2.94E-07 | 2.51E-06 | 1.07E-06 | CAV1/IFNG/IL2/IL4/IL6/STAT3/TNF | 7 |
| GO:0071621 | granulocyte chemotaxis | Aug-97 | 127/18866 | 2.96E-07 | 2.51E-06 | 1.07E-06 | CCL2/CXCL8/DPP4/IL1B/IL4/MAPK1/MAPK3/PIK3CG | 8 |
| GO:1905477 | positive regulation of protein localization to membrane | Aug-97 | 127/18866 | 2.96E-07 | 2.51E-06 | 1.07E-06 | AKT1/BCL2/EGFR/ERBB2/IFNG/MAPK8/TNF/TP53 | 8 |
| GO:0061756 | leukocyte adhesion to vascular endothelial cell | Jun-97 | 53/18866 | 2.98E-07 | 2.53E-06 | 1.08E-06 | ALOX5/ICAM1/IL6/SELE/TNF/VCAM1 | 6 |
| GO:0032386 | regulation of intracellular transport | Dec-97 | 358/18866 | 3.04E-07 | 2.57E-06 | 1.10E-06 | ADIPOQ/BCL2/ERBB2/IFNG/IL1B/MAPK1/MAPK3/MAPK8/PTGS2/SREBF1/TGFB1/TP53 | 12 |
| GO:0042368 | vitamin D biosynthetic process | Apr-97 | 12/18866 | 3.15E-07 | 2.65E-06 | 1.13E-06 | CYP3A4/IFNG/IL1B/TNF | 4 |
| GO:0060736 | prostate gland growth | Apr-97 | 12/18866 | 3.15E-07 | 2.65E-06 | 1.13E-06 | AR/CYP19A1/ESR1/PTEN | 4 |
| GO:0030857 | negative regulation of epithelial cell differentiation | Jun-97 | 54/18866 | 3.34E-07 | 2.81E-06 | 1.20E-06 | CAV1/CTNNB1/IFNG/MMP9/VEGFA/XDH | 6 |
| GO:0052548 | regulation of endopeptidase activity | 13/97 | 434/18866 | 3.41E-07 | 2.86E-06 | 1.22E-06 | AKT1/BIRC5/F3/MMP9/MYC/PPARG/PTGS2/SERPINE1/SLPI/STAT3/TNF/VEGFA/XDH | 13 |
| GO:0008209 | androgen metabolic process | May-97 | 29/18866 | 3.49E-07 | 2.90E-06 | 1.24E-06 | CYP19A1/CYP3A4/ESR1/HSD3B1/SPP1 | 5 |
| GO:0036296 | response to increased oxygen levels | May-97 | 29/18866 | 3.49E-07 | 2.90E-06 | 1.24E-06 | CAT/CAV1/CDKN1A/CYP1A1/PPARG | 5 |
| GO:0050820 | positive regulation of coagulation | May-97 | 29/18866 | 3.49E-07 | 2.90E-06 | 1.24E-06 | F2/F3/F7/SERPINE1/THBD | 5 |
| GO:0007260 | tyrosine phosphorylation of STAT protein | Jul-97 | 89/18866 | 3.73E-07 | 3.10E-06 | 1.32E-06 | CAV1/IFNG/IL2/IL4/IL6/STAT3/TNF | 7 |
| GO:0050796 | regulation of insulin secretion | Sep-97 | 181/18866 | 3.91E-07 | 3.24E-06 | 1.38E-06 | ALOX5/DPP4/IFNG/IL1B/IL6/NOS2/PPARD/SREBF1/TNF | 9 |
| GO:0090316 | positive regulation of intracellular protein transport | Sep-97 | 182/18866 | 4.09E-07 | 3.38E-06 | 1.44E-06 | BCL2/ERBB2/IFNG/IL1B/MAPK1/MAPK8/PTGS2/TGFB1/TP53 | 9 |
| GO:0060688 | regulation of morphogenesis of a branching structure | Jun-97 | 56/18866 | 4.17E-07 | 3.44E-06 | 1.47E-06 | AR/CTNNB1/ESR1/RXRA/TNF/VEGFA | 6 |
| GO:0045861 | negative regulation of proteolysis | Dec-97 | 369/18866 | 4.20E-07 | 3.46E-06 | 1.47E-06 | AKT1/BIRC5/F2/IL10/MMP9/PLAT/PTGS2/SERPINE1/SLPI/TNF/TP53/VEGFA | 12 |
| GO:2000116 | regulation of cysteine-type endopeptidase activity | Oct-97 | 239/18866 | 4.23E-07 | 3.48E-06 | 1.48E-06 | AKT1/BIRC5/F3/MMP9/MYC/PPARG/PTGS2/TNF/VEGFA/XDH | 10 |
| GO:0071347 | cellular response to interleukin-1 | Sep-97 | 183/18866 | 4.28E-07 | 3.51E-06 | 1.49E-06 | CCL2/CXCL8/ICAM1/IKBKB/IL1A/IL1B/IL6/MAPK3/NFKBIA | 9 |
| GO:2001235 | positive regulation of apoptotic signaling pathway | Sep-97 | 183/18866 | 4.28E-07 | 3.51E-06 | 1.49E-06 | BCL2/BCL2L1/CAV1/MAPK8/MMP9/PTEN/SOD1/TNF/TP53 | 9 |
| GO:0031650 | regulation of heat generation | Apr-97 | 13/18866 | 4.53E-07 | 3.70E-06 | 1.58E-06 | IL1B/PTGER3/PTGS2/TNF | 4 |
| GO:0001541 | ovarian follicle development | Jun-97 | 57/18866 | 4.64E-07 | 3.78E-06 | 1.61E-06 | BCL2/BCL2L1/ESR1/ICAM1/SOD1/VEGFA | 6 |
| GO:0002532 | production of molecular mediator involved in inflammatory response | Jul-97 | 93/18866 | 5.04E-07 | 4.09E-06 | 1.74E-06 | ALOX5/F2/IL6/NOS2/SERPINE1/STAT3/TNF | 7 |
| GO:0014909 | smooth muscle cell migration | Jul-97 | 93/18866 | 5.04E-07 | 4.09E-06 | 1.74E-06 | ADIPOQ/BCL2/GSTP1/IGFBP3/PLAT/PPARD/SERPINE1 | 7 |
| GO:0008217 | regulation of blood pressure | Sep-97 | 187/18866 | 5.14E-07 | 4.16E-06 | 1.77E-06 | ADIPOQ/AR/HMOX1/NOS2/NOS3/PPARA/PPARG/PTGS2/SOD1 | 9 |
| GO:0006959 | humoral immune response | Dec-97 | 377/18866 | 5.27E-07 | 4.26E-06 | 1.81E-06 | ALOX5/BCL2/CCL2/CRP/CXCL8/F2/IFNG/IL1B/IL6/LYZ/SLPI/TNF | 12 |
| GO:0051591 | response to cAMP | Jul-97 | 94/18866 | 5.42E-07 | 4.37E-06 | 1.86E-06 | ADIPOQ/AHR/FOS/PIK3CG/SREBF1/THBD/TYR | 7 |
| GO:0006469 | negative regulation of protein kinase activity | Oct-97 | 246/18866 | 5.51E-07 | 4.43E-06 | 1.89E-06 | ADIPOQ/AKT1/CASP3/CAV1/CDKN1A/GSTP1/HSPB1/IFNG/IL1B/PTEN | 10 |
| GO:0043534 | blood vessel endothelial cell migration | Sep-97 | 189/18866 | 5.62E-07 | 4.51E-06 | 1.92E-06 | AKT1/HMOX1/HSPB1/NOS3/PPARG/PTGS2/TGFB1/TNF/VEGFA | 9 |
| GO:0048008 | platelet-derived growth factor receptor signaling pathway | Jun-97 | 59/18866 | 5.71E-07 | 4.57E-06 | 1.95E-06 | ADIPOQ/F3/F7/PLAT/PTEN/VEGFA | 6 |
| GO:0008210 | estrogen metabolic process | May-97 | 32/18866 | 5.84E-07 | 4.67E-06 | 1.99E-06 | CYP19A1/CYP1A1/CYP1A2/CYP3A4/HSD3B1 | 5 |
| GO:0043401 | steroid hormone mediated signaling pathway | Aug-97 | 139/18866 | 5.90E-07 | 4.71E-06 | 2.01E-06 | AR/ESR1/NR3C1/NR3C2/PGR/PPARA/PPARD/RXRA | 8 |
| GO:0001701 | in utero embryonic development | Dec-97 | 383/18866 | 6.23E-07 | 4.95E-06 | 2.11E-06 | AKT1/APOB/AR/BCL2L1/CTNNB1/EGFR/IL10/MAPK1/NOS3/RXRA/TP53/VEGFA | 12 |
| GO:0060968 | regulation of gene silencing | Aug-97 | 140/18866 | 6.23E-07 | 4.95E-06 | 2.11E-06 | EGFR/ESR1/IL6/PPARG/STAT3/TGFB1/TNF/TP53 | 8 |
| GO:0001836 | release of cytochrome c from mitochondria | Jun-97 | 60/18866 | 6.32E-07 | 4.99E-06 | 2.13E-06 | AKT1/BCL2/BCL2L1/JUN/MMP9/TP53 | 6 |
| GO:0043388 | positive regulation of DNA binding | Jun-97 | 60/18866 | 6.32E-07 | 4.99E-06 | 2.13E-06 | CTNNB1/EGF/IFNG/MMP9/PPARG/TGFB1 | 6 |
| GO:1903428 | positive regulation of reactive oxygen species biosynthetic process | Jun-97 | 60/18866 | 6.32E-07 | 4.99E-06 | 2.13E-06 | AKT1/ICAM1/IFNG/IL1B/PTGS2/TNF | 6 |
| GO:0036473 | cell death in response to oxidative stress | Jul-97 | 97/18866 | 6.72E-07 | 5.27E-06 | 2.25E-06 | AKT1/BCL2/CTNNB1/HSPB1/IL10/MMP3/SOD1 | 7 |
| GO:0071674 | mononuclear cell migration | Jul-97 | 97/18866 | 6.72E-07 | 5.27E-06 | 2.25E-06 | CCL2/IL4/IL6/MAPK1/MAPK3/SERPINE1/TNF | 7 |
| GO:1901216 | positive regulation of neuron death | Jul-97 | 97/18866 | 6.72E-07 | 5.27E-06 | 2.25E-06 | CASP3/CTNNB1/FOS/IFNG/JUN/TNF/TP53 | 7 |
| GO:0043122 | regulation of I-kappaB kinase/NF-kappaB signaling | Oct-97 | 252/18866 | 6.87E-07 | 5.37E-06 | 2.29E-06 | ADIPOQ/AKT1/CTNNB1/ESR1/GSTP1/HMOX1/HSPB1/IKBKB/IL1B/TNF | 10 |
| GO:1902041 | regulation of extrinsic apoptotic signaling pathway via death domain receptors | Jun-97 | 61/18866 | 6.98E-07 | 5.45E-06 | 2.32E-06 | BCL2L1/HMOX1/ICAM1/NOS3/PTEN/SERPINE1 | 6 |
| GO:0051924 | regulation of calcium ion transport | Oct-97 | 253/18866 | 7.12E-07 | 5.55E-06 | 2.36E-06 | BCL2/CAV1/CCL2/CTNNB1/EGF/F2/ICAM1/NOS3/PIK3CG/PTGS2 | 10 |
| GO:0050864 | regulation of B cell activation | Sep-97 | 195/18866 | 7.31E-07 | 5.68E-06 | 2.42E-06 | AHR/BCL2/CASP3/CDKN1A/IL10/IL2/IL4/IL6/TGFB1 | 9 |
| GO:0014706 | striated muscle tissue development | Dec-97 | 389/18866 | 7.34E-07 | 5.69E-06 | 2.43E-06 | BCL2/CAV1/CTNNB1/ERBB3/FOS/MAPK1/PIM1/PPARA/PTEN/RXRA/TGFB1/VEGFA | 12 |
| GO:0031331 | positive regulation of cellular catabolic process | Dec-97 | 390/18866 | 7.54E-07 | 5.84E-06 | 2.49E-06 | AKT1/CAV1/EGF/HMOX1/IFNG/IL1B/IL4/IL6/MAPK3/PPARA/PTEN/TNF | 12 |
| GO:2000351 | regulation of endothelial cell apoptotic process | Jun-97 | 62/18866 | 7.70E-07 | 5.95E-06 | 2.54E-06 | CCL2/CD40LG/ICAM1/IL4/SERPINE1/TNF | 6 |
| GO:0010212 | response to ionizing radiation | Aug-97 | 144/18866 | 7.73E-07 | 5.95E-06 | 2.54E-06 | BCL2/BCL2L1/CASP3/CDKN1A/ICAM1/MYC/THBD/TP53 | 8 |
| GO:0048872 | homeostasis of number of cells | Oct-97 | 256/18866 | 7.93E-07 | 6.10E-06 | 2.60E-06 | AKT1/BCL2/CASP3/HMOX1/IL2/IL6/NOS3/SOD1/STAT3/VEGFA | 10 |
| GO:0045907 | positive regulation of vasoconstriction | May-97 | 34/18866 | 8.01E-07 | 6.15E-06 | 2.62E-06 | AKT1/CAV1/EGFR/ICAM1/PTGS2 | 5 |
| GO:0038127 | ERBB signaling pathway | Aug-97 | 145/18866 | 8.14E-07 | 6.24E-06 | 2.66E-06 | AKT1/EGF/EGFR/ERBB2/ERBB3/MAPK1/MMP9/TGFB1 | 8 |
| GO:0030072 | peptide hormone secretion | Oct-97 | 257/18866 | 8.22E-07 | 6.28E-06 | 2.68E-06 | ALOX5/DPP4/EGFR/IFNG/IL1B/IL6/NOS2/PPARD/SREBF1/TNF | 10 |
| GO:0002697 | regulation of immune effector process | 13/97 | 470/18866 | 8.38E-07 | 6.39E-06 | 2.73E-06 | CD40LG/F2/HMOX1/ICAM1/IFNG/IL10/IL1B/IL2/IL4/IL6/NOS2/TGFB1/TNF | 13 |
| GO:0042542 | response to hydrogen peroxide | Aug-97 | 146/18866 | 8.58E-07 | 6.48E-06 | 2.76E-06 | BCL2/CASP3/CAT/HMOX1/IL10/IL6/JUN/SOD1 | 8 |
| GO:0042362 | fat-soluble vitamin biosynthetic process | Apr-97 | 15/18866 | 8.58E-07 | 6.48E-06 | 2.76E-06 | CYP3A4/IFNG/IL1B/TNF | 4 |
| GO:0090594 | inflammatory response to wounding | Apr-97 | 15/18866 | 8.58E-07 | 6.48E-06 | 2.76E-06 | ALOX5/HMOX1/IL1A/TGFB1 | 4 |
| GO:1901550 | regulation of endothelial cell development | Apr-97 | 15/18866 | 8.58E-07 | 6.48E-06 | 2.76E-06 | IKBKB/IL1B/TNF/VEGFA | 4 |
| GO:1903140 | regulation of establishment of endothelial barrier | Apr-97 | 15/18866 | 8.58E-07 | 6.48E-06 | 2.76E-06 | IKBKB/IL1B/TNF/VEGFA | 4 |
| GO:0042102 | positive regulation of T cell proliferation | Jul-97 | 101/18866 | 8.85E-07 | 6.67E-06 | 2.84E-06 | CD40LG/IL1A/IL1B/IL2/IL4/IL6/VCAM1 | 7 |
| GO:0009755 | hormone-mediated signaling pathway | Sep-97 | 200/18866 | 9.03E-07 | 6.78E-06 | 2.89E-06 | AR/ESR1/NR3C1/NR3C2/PGR/PPARA/PPARD/PPARG/RXRA | 9 |
| GO:0017038 | protein import | Sep-97 | 200/18866 | 9.03E-07 | 6.78E-06 | 2.89E-06 | AKT1/CDKN1A/IFNG/MAPK1/NFKBIA/PTGS2/STAT3/TGFB1/TP53 | 9 |
| GO:0001885 | endothelial cell development | Jun-97 | 64/18866 | 9.31E-07 | 6.96E-06 | 2.97E-06 | CTNNB1/ICAM1/IKBKB/IL1B/TNF/VEGFA | 6 |
| GO:0033344 | cholesterol efflux | Jun-97 | 64/18866 | 9.31E-07 | 6.96E-06 | 2.97E-06 | ADIPOQ/APOB/CAV1/EGF/NFKBIA/PON1 | 6 |
| GO:1901653 | cellular response to peptide | Dec-97 | 398/18866 | 9.34E-07 | 6.97E-06 | 2.97E-06 | ADIPOQ/AKT1/CAV1/GSTP1/ICAM1/IL1B/PPARG/PTEN/SREBF1/STAT3/TP53/VCAM1 | 12 |
| GO:1903076 | regulation of protein localization to plasma membrane | Jul-97 | 102/18866 | 9.46E-07 | 7.04E-06 | 3.00E-06 | AKT1/AR/BCL2L1/EGFR/IFNG/TGFB1/TNF | 7 |
| GO:0043524 | negative regulation of neuron apoptotic process | Aug-97 | 149/18866 | 1.00E-06 | 7.44E-06 | 3.17E-06 | BCL2/BCL2L1/CCL2/ERBB3/HMOX1/IL10/JUN/SOD1 | 8 |
| GO:0010573 | vascular endothelial growth factor production | Jun-97 | 65/18866 | 1.02E-06 | 7.58E-06 | 3.23E-06 | IL1A/IL1B/IL6/PTGS2/TGFB1/TNF | 6 |
| GO:0097530 | granulocyte migration | Aug-97 | 150/18866 | 1.05E-06 | 7.79E-06 | 3.32E-06 | CCL2/CXCL8/DPP4/IL1B/IL4/MAPK1/MAPK3/PIK3CG | 8 |
| GO:0032147 | activation of protein kinase activity | Nov-97 | 331/18866 | 1.07E-06 | 7.86E-06 | 3.35E-06 | AKT1/CD40LG/EGF/EGFR/IL1B/IL4/MAPK1/MAPK3/SOD1/TNF/VEGFA | 11 |
| GO:0060562 | epithelial tube morphogenesis | Nov-97 | 331/18866 | 1.07E-06 | 7.86E-06 | 3.35E-06 | AR/BCL2/CASP3/CTNNB1/EGF/ESR1/MYC/PGR/TGFB1/TNF/VEGFA | 11 |
| GO:0042554 | superoxide anion generation | May-97 | 36/18866 | 1.08E-06 | 7.92E-06 | 3.38E-06 | CRP/EGFR/GSTP1/SOD1/TGFB1 | 5 |
| GO:0002367 | cytokine production involved in immune response | Jul-97 | 104/18866 | 1.08E-06 | 7.92E-06 | 3.38E-06 | HMOX1/IL10/IL1B/IL4/IL6/TGFB1/TNF | 7 |
| GO:0046824 | positive regulation of nucleocytoplasmic transport | Jun-97 | 66/18866 | 1.12E-06 | 8.19E-06 | 3.49E-06 | IFNG/IL1B/MAPK1/PTGS2/TGFB1/TP53 | 6 |
| GO:2000378 | negative regulation of reactive oxygen species metabolic process | Jun-97 | 66/18866 | 1.12E-06 | 8.19E-06 | 3.49E-06 | BCL2/CAV1/IL10/MMP3/STAT3/TP53 | 6 |
| GO:0062014 | negative regulation of small molecule metabolic process | Jul-97 | 105/18866 | 1.15E-06 | 8.40E-06 | 3.58E-06 | ADIPOQ/AKT1/PPARA/SOD1/STAT3/TGFB1/TP53 | 7 |
| GO:0015850 | organic hydroxy compound transport | Oct-97 | 268/18866 | 1.20E-06 | 8.69E-06 | 3.70E-06 | ADIPOQ/APOB/CAV1/CYP19A1/EGF/NFKBIA/PON1/PPARG/RXRA/SPP1 | 10 |
| GO:0033673 | negative regulation of kinase activity | Oct-97 | 268/18866 | 1.20E-06 | 8.69E-06 | 3.70E-06 | ADIPOQ/AKT1/CASP3/CAV1/CDKN1A/GSTP1/HSPB1/IFNG/IL1B/PTEN | 10 |
| GO:0034284 | response to monosaccharide | Sep-97 | 207/18866 | 1.20E-06 | 8.69E-06 | 3.70E-06 | ADIPOQ/CASP3/CAT/GSTP1/ICAM1/PPARD/PTEN/PTGS2/SREBF1 | 9 |
| GO:0046890 | regulation of lipid biosynthetic process | Sep-97 | 207/18866 | 1.20E-06 | 8.69E-06 | 3.70E-06 | ADIPOQ/AKT1/APOB/IFNG/IL1B/PTGS2/SOD1/SREBF1/TNF | 9 |
| GO:0050866 | negative regulation of cell activation | Sep-97 | 207/18866 | 1.20E-06 | 8.69E-06 | 3.70E-06 | CASP3/ERBB2/F2/HMOX1/IL10/IL2/IL4/NOS3/THBD | 9 |
| GO:0016202 | regulation of striated muscle tissue development | Aug-97 | 153/18866 | 1.22E-06 | 8.79E-06 | 3.75E-06 | BCL2/CTNNB1/ERBB3/MAPK1/PIM1/PPARA/PTEN/TGFB1 | 8 |
| GO:0045123 | cellular extravasation | Jun-97 | 67/18866 | 1.22E-06 | 8.79E-06 | 3.75E-06 | CCL2/ICAM1/PIK3CG/SELE/TNF/VCAM1 | 6 |
| GO:1900015 | regulation of cytokine production involved in inflammatory response | Jun-97 | 67/18866 | 1.22E-06 | 8.79E-06 | 3.75E-06 | ALOX5/F2/IL6/NOS2/STAT3/TNF | 6 |
| GO:0014812 | muscle cell migration | Jul-97 | 106/18866 | 1.23E-06 | 8.79E-06 | 3.75E-06 | ADIPOQ/BCL2/GSTP1/IGFBP3/PLAT/PPARD/SERPINE1 | 7 |
| GO:0032611 | interleukin-1 beta production | Jul-97 | 106/18866 | 1.23E-06 | 8.79E-06 | 3.75E-06 | GSTP1/HSPB1/IFNG/IL1B/IL6/STAT3/TNF | 7 |
| GO:0030212 | hyaluronan metabolic process | May-97 | 37/18866 | 1.24E-06 | 8.84E-06 | 3.77E-06 | AKT1/EGF/IL1B/PIM1/TGFB1 | 5 |
| GO:1904994 | regulation of leukocyte adhesion to vascular endothelial cell | May-97 | 37/18866 | 1.24E-06 | 8.84E-06 | 3.77E-06 | ALOX5/ICAM1/IL6/SELE/TNF | 5 |
| GO:0060537 | muscle tissue development | Dec-97 | 409/18866 | 1.24E-06 | 8.84E-06 | 3.77E-06 | BCL2/CAV1/CTNNB1/ERBB3/FOS/MAPK1/PIM1/PPARA/PTEN/RXRA/TGFB1/VEGFA | 12 |
| GO:0030258 | lipid modification | Oct-97 | 271/18866 | 1.33E-06 | 9.44E-06 | 4.02E-06 | ADIPOQ/AKT1/ALOX5/CYP1A1/CYP3A4/PIK3CG/PPARA/PPARD/PPARG/PTEN | 10 |
| GO:0032642 | regulation of chemokine production | Jun-97 | 68/18866 | 1.34E-06 | 9.46E-06 | 4.04E-06 | ADIPOQ/GSTP1/IL10/IL1B/IL6/TNF | 6 |
| GO:0050766 | positive regulation of phagocytosis | Jun-97 | 68/18866 | 1.34E-06 | 9.46E-06 | 4.04E-06 | CCL2/IFNG/IL1B/PPARG/SOD1/TNF | 6 |
| GO:0006109 | regulation of carbohydrate metabolic process | Sep-97 | 210/18866 | 1.35E-06 | 9.57E-06 | 4.08E-06 | ADIPOQ/AKT1/EGF/IFNG/IGFBP3/PPARA/STAT3/TGFB1/TP53 | 9 |
| GO:0071887 | leukocyte apoptotic process | Jul-97 | 108/18866 | 1.39E-06 | 9.82E-06 | 4.19E-06 | AKT1/CASP3/IL10/IL2/IL6/PTEN/TP53 | 7 |
| GO:1901861 | regulation of muscle tissue development | Aug-97 | 156/18866 | 1.42E-06 | 9.96E-06 | 4.25E-06 | BCL2/CTNNB1/ERBB3/MAPK1/PIM1/PPARA/PTEN/TGFB1 | 8 |
| GO:0014912 | negative regulation of smooth muscle cell migration | May-97 | 38/18866 | 1.42E-06 | 9.96E-06 | 4.25E-06 | ADIPOQ/GSTP1/IGFBP3/PPARD/SERPINE1 | 5 |
| GO:0032094 | response to food | May-97 | 38/18866 | 1.42E-06 | 9.96E-06 | 4.25E-06 | AKT1/CYP1A1/MPO/PPARA/SREBF1 | 5 |
| GO:0045930 | negative regulation of mitotic cell cycle | Nov-97 | 341/18866 | 1.42E-06 | 9.97E-06 | 4.25E-06 | BCL2/BCL2L1/CCL2/CDKN1A/CTNNB1/EGFR/IL10/PTEN/TGFB1/TNF/TP53 | 11 |
| GO:0045927 | positive regulation of growth | Oct-97 | 274/18866 | 1.47E-06 | 1.02E-05 | 4.37E-06 | AKT1/BCL2/EGFR/ERBB2/F2/IL2/MAPK1/PIM1/PPARD/VEGFA | 10 |
| GO:0002286 | T cell activation involved in immune response | Jul-97 | 109/18866 | 1.48E-06 | 1.03E-05 | 4.37E-06 | ICAM1/IFNG/IL2/IL4/IL6/STAT3/TP53 | 7 |
| GO:0009299 | mRNA transcription | Apr-97 | 17/18866 | 1.48E-06 | 1.03E-05 | 4.37E-06 | PPARD/SREBF1/STAT3/TP53 | 4 |
| GO:0030730 | sequestering of triglyceride | Apr-97 | 17/18866 | 1.48E-06 | 1.03E-05 | 4.37E-06 | IL1B/PPARA/PPARG/TNF | 4 |
| GO:0050665 | hydrogen peroxide biosynthetic process | Apr-97 | 17/18866 | 1.48E-06 | 1.03E-05 | 4.37E-06 | CYP1A1/CYP1A2/SOD1/STAT3 | 4 |
| GO:0043271 | negative regulation of ion transport | Aug-97 | 157/18866 | 1.48E-06 | 1.03E-05 | 4.37E-06 | AKT1/BCL2/CAV1/ICAM1/MMP9/NOS3/PTEN/PTGS2 | 8 |
| GO:0048634 | regulation of muscle organ development | Aug-97 | 157/18866 | 1.48E-06 | 1.03E-05 | 4.37E-06 | BCL2/CTNNB1/ERBB3/MAPK1/PIM1/PPARA/PTEN/TGFB1 | 8 |
| GO:0030073 | insulin secretion | Sep-97 | 213/18866 | 1.52E-06 | 1.05E-05 | 4.48E-06 | ALOX5/DPP4/IFNG/IL1B/IL6/NOS2/PPARD/SREBF1/TNF | 9 |
| GO:0002534 | cytokine production involved in inflammatory response | Jun-97 | 70/18866 | 1.59E-06 | 1.09E-05 | 4.66E-06 | ALOX5/F2/IL6/NOS2/STAT3/TNF | 6 |
| GO:0034504 | protein localization to nucleus | Oct-97 | 277/18866 | 1.62E-06 | 1.11E-05 | 4.74E-06 | AKT1/CDKN1A/F2/IFNG/MAPK1/NFKBIA/PTGS2/STAT3/TGFB1/TP53 | 10 |
| GO:0032652 | regulation of interleukin-1 production | Jul-97 | 111/18866 | 1.68E-06 | 1.15E-05 | 4.90E-06 | GSTP1/HSPB1/IFNG/IL10/IL6/STAT3/TNF | 7 |
| GO:0014823 | response to activity | Jun-97 | 71/18866 | 1.73E-06 | 1.18E-05 | 5.02E-06 | ADIPOQ/CAT/IL10/IL6/PPARD/PTEN | 6 |
| GO:0032371 | regulation of sterol transport | Jun-97 | 71/18866 | 1.73E-06 | 1.18E-05 | 5.02E-06 | ADIPOQ/CAV1/EGF/NFKBIA/PON1/PPARG | 6 |
| GO:0035690 | cellular response to drug | Jun-97 | 71/18866 | 1.73E-06 | 1.18E-05 | 5.02E-06 | ADIPOQ/EGFR/IL1B/MYC/NOS2/TP53 | 6 |
| GO:0042531 | positive regulation of tyrosine phosphorylation of STAT protein | Jun-97 | 71/18866 | 1.73E-06 | 1.18E-05 | 5.02E-06 | IFNG/IL2/IL4/IL6/STAT3/TNF | 6 |
| GO:0001938 | positive regulation of endothelial cell proliferation | Jul-97 | 113/18866 | 1.89E-06 | 1.28E-05 | 5.47E-06 | AKT1/F3/HMOX1/IL10/JUN/STAT3/VEGFA | 7 |
| GO:0010885 | regulation of cholesterol storage | Apr-97 | 18/18866 | 1.90E-06 | 1.29E-05 | 5.49E-06 | APOB/PPARA/PPARD/PPARG | 4 |
| GO:0051023 | regulation of immunoglobulin secretion | Apr-97 | 18/18866 | 1.90E-06 | 1.29E-05 | 5.49E-06 | CD40LG/IL2/IL6/TNF | 4 |
| GO:0016101 | diterpenoid metabolic process | Jul-97 | 114/18866 | 2.00E-06 | 1.35E-05 | 5.78E-06 | APOB/CYP1A1/CYP1A2/CYP3A4/EGFR/PPARD/RHO | 7 |
| GO:1902042 | negative regulation of extrinsic apoptotic signaling pathway via death domain receptors | May-97 | 41/18866 | 2.10E-06 | 1.41E-05 | 6.03E-06 | BCL2L1/HMOX1/ICAM1/NOS3/SERPINE1 | 5 |
| GO:0032388 | positive regulation of intracellular transport | Sep-97 | 222/18866 | 2.14E-06 | 1.44E-05 | 6.15E-06 | BCL2/ERBB2/IFNG/IL1B/MAPK1/MAPK8/PTGS2/TGFB1/TP53 | 9 |
| GO:0002699 | positive regulation of immune effector process | Sep-97 | 223/18866 | 2.22E-06 | 1.49E-05 | 6.37E-06 | HMOX1/IFNG/IL1B/IL2/IL4/IL6/NOS2/TGFB1/TNF | 9 |
| GO:0034250 | positive regulation of cellular amide metabolic process | Aug-97 | 167/18866 | 2.36E-06 | 1.58E-05 | 6.75E-06 | CASP3/ERBB2/IFNG/IL6/MAPK1/MAPK3/RXRA/TNF | 8 |
| GO:0045446 | endothelial cell differentiation | Jul-97 | 117/18866 | 2.39E-06 | 1.59E-05 | 6.80E-06 | CTNNB1/ICAM1/IKBKB/IL1B/TNF/VEGFA/XDH | 7 |
| GO:0032602 | chemokine production | Jun-97 | 75/18866 | 2.39E-06 | 1.59E-05 | 6.80E-06 | ADIPOQ/GSTP1/IL10/IL1B/IL6/TNF | 6 |
| GO:0033189 | response to vitamin A | Apr-97 | 19/18866 | 2.40E-06 | 1.59E-05 | 6.80E-06 | CAT/CYP1A1/PPARD/PPARG | 4 |
| GO:0060749 | mammary gland alveolus development | Apr-97 | 19/18866 | 2.40E-06 | 1.59E-05 | 6.80E-06 | AR/EGF/ESR1/VEGFA | 4 |
| GO:0061377 | mammary gland lobule development | Apr-97 | 19/18866 | 2.40E-06 | 1.59E-05 | 6.80E-06 | AR/EGF/ESR1/VEGFA | 4 |
| GO:0007569 | cell aging | Jul-97 | 118/18866 | 2.53E-06 | 1.67E-05 | 7.13E-06 | BCL2/CDKN1A/ICAM1/PTEN/SERPINE1/SOD1/TP53 | 7 |
| GO:0015918 | sterol transport | Jul-97 | 118/18866 | 2.53E-06 | 1.67E-05 | 7.13E-06 | ADIPOQ/APOB/CAV1/EGF/NFKBIA/PON1/PPARG | 7 |
| GO:0046688 | response to copper ion | May-97 | 43/18866 | 2.67E-06 | 1.77E-05 | 7.53E-06 | CYP1A1/CYP1A2/ICAM1/IL1A/SOD1 | 5 |
| GO:0032635 | interleukin-6 production | Aug-97 | 170/18866 | 2.69E-06 | 1.78E-05 | 7.58E-06 | IFNG/IL10/IL1A/IL1B/IL6/NOS2/STAT3/TNF | 8 |
| GO:1903201 | regulation of oxidative stress-induced cell death | Jun-97 | 77/18866 | 2.79E-06 | 1.84E-05 | 7.84E-06 | AKT1/CTNNB1/HSPB1/IL10/MMP3/SOD1 | 6 |
| GO:0046394 | carboxylic acid biosynthetic process | Nov-97 | 367/18866 | 2.90E-06 | 1.91E-05 | 8.13E-06 | ADIPOQ/ALOX5/CYP1A1/CYP1A2/CYP3A4/EGF/GSTM1/GSTP1/IL1B/PTGS2/TGFB1 | 11 |
| GO:0016053 | organic acid biosynthetic process | Nov-97 | 368/18866 | 2.98E-06 | 1.95E-05 | 8.32E-06 | ADIPOQ/ALOX5/CYP1A1/CYP1A2/CYP3A4/EGF/GSTM1/GSTP1/IL1B/PTGS2/TGFB1 | 11 |
| GO:0002544 | chronic inflammatory response | Apr-97 | 20/18866 | 2.99E-06 | 1.95E-05 | 8.32E-06 | CYP19A1/IL10/IL4/TNF | 4 |
| GO:0010875 | positive regulation of cholesterol efflux | Apr-97 | 20/18866 | 2.99E-06 | 1.95E-05 | 8.32E-06 | ADIPOQ/CAV1/NFKBIA/PON1 | 4 |
| GO:0010878 | cholesterol storage | Apr-97 | 20/18866 | 2.99E-06 | 1.95E-05 | 8.32E-06 | APOB/PPARA/PPARD/PPARG | 4 |
| GO:0032965 | regulation of collagen biosynthetic process | May-97 | 44/18866 | 3.00E-06 | 1.96E-05 | 8.34E-06 | F2/IL6/PPARD/PPARG/TGFB1 | 5 |
| GO:0002460 | adaptive immune response based on somatic recombination of immune receptors built from immunoglobulin superfamily domains | Nov-97 | 370/18866 | 3.14E-06 | 2.04E-05 | 8.70E-06 | CD40LG/CRP/ICAM1/IL10/IL1B/IL2/IL4/IL6/STAT3/TGFB1/TNF | 11 |
| GO:0051099 | positive regulation of binding | Aug-97 | 174/18866 | 3.20E-06 | 2.08E-05 | 8.88E-06 | CAV1/CTNNB1/EGF/IFNG/MMP9/PON1/PPARG/TGFB1 | 8 |
| GO:0071260 | cellular response to mechanical stimulus | Jun-97 | 79/18866 | 3.25E-06 | 2.10E-05 | 8.97E-06 | AKT1/EGFR/IL1B/MAPK3/MAPK8/PTGS2 | 6 |
| GO:1904375 | regulation of protein localization to cell periphery | Jul-97 | 123/18866 | 3.33E-06 | 2.16E-05 | 9.20E-06 | AKT1/AR/BCL2L1/EGFR/IFNG/TGFB1/TNF | 7 |
| GO:0014002 | astrocyte development | May-97 | 45/18866 | 3.36E-06 | 2.17E-05 | 9.25E-06 | EGFR/IFNG/IL1B/IL6/TNF | 5 |
| GO:0090311 | regulation of protein deacetylation | May-97 | 45/18866 | 3.36E-06 | 2.17E-05 | 9.25E-06 | IFNG/MAPK8/SREBF1/TP53/VEGFA | 5 |
| GO:0048568 | embryonic organ development | Dec-97 | 451/18866 | 3.42E-06 | 2.20E-05 | 9.38E-06 | AKT1/CTNNB1/CXCL8/EGFR/IL10/MAPK1/MAPK3/SOD1/TGFB1/TNF/TP53/VEGFA | 12 |
| GO:0002312 | B cell activation involved in immune response | Jun-97 | 80/18866 | 3.49E-06 | 2.24E-05 | 9.56E-06 | CD40LG/IL10/IL2/IL4/IL6/TGFB1 | 6 |
| GO:1900182 | positive regulation of protein localization to nucleus | Jun-97 | 80/18866 | 3.49E-06 | 2.24E-05 | 9.56E-06 | AKT1/F2/IFNG/MAPK1/PTGS2/TGFB1 | 6 |
| GO:0006721 | terpenoid metabolic process | Jul-97 | 124/18866 | 3.52E-06 | 2.25E-05 | 9.61E-06 | APOB/CYP1A1/CYP1A2/CYP3A4/EGFR/PPARD/RHO | 7 |
| GO:0009896 | positive regulation of catabolic process | Dec-97 | 454/18866 | 3.66E-06 | 2.33E-05 | 9.92E-06 | AKT1/CAV1/EGF/HMOX1/IFNG/IL1B/IL4/IL6/MAPK3/PPARA/PTEN/TNF | 12 |
| GO:0046777 | protein autophosphorylation | Sep-97 | 237/18866 | 3.66E-06 | 2.33E-05 | 9.92E-06 | ADIPOQ/AKT1/CAV1/EGFR/ERBB2/JUN/MAPK3/PIM1/VEGFA | 9 |
| GO:0009110 | vitamin biosynthetic process | Apr-97 | 21/18866 | 3.67E-06 | 2.33E-05 | 9.92E-06 | CYP3A4/IFNG/IL1B/TNF | 4 |
| GO:0010893 | positive regulation of steroid biosynthetic process | Apr-97 | 21/18866 | 3.67E-06 | 2.33E-05 | 9.92E-06 | IFNG/IL1B/SREBF1/TNF | 4 |
| GO:0048305 | immunoglobulin secretion | Apr-97 | 21/18866 | 3.67E-06 | 2.33E-05 | 9.92E-06 | CD40LG/IL2/IL6/TNF | 4 |
| GO:0061042 | vascular wound healing | Apr-97 | 21/18866 | 3.67E-06 | 2.33E-05 | 9.92E-06 | ALOX5/SERPINE1/TNF/VEGFA | 4 |
| GO:0090312 | positive regulation of protein deacetylation | Apr-97 | 21/18866 | 3.67E-06 | 2.33E-05 | 9.92E-06 | IFNG/SREBF1/TP53/VEGFA | 4 |
| GO:0008637 | apoptotic mitochondrial changes | Jul-97 | 125/18866 | 3.71E-06 | 2.35E-05 | 1.00E-05 | AKT1/BCL2/BCL2L1/JUN/MAPK8/MMP9/TP53 | 7 |
| GO:0010874 | regulation of cholesterol efflux | May-97 | 46/18866 | 3.76E-06 | 2.37E-05 | 1.01E-05 | ADIPOQ/CAV1/EGF/NFKBIA/PON1 | 5 |
| GO:0001937 | negative regulation of endothelial cell proliferation | Jun-97 | 81/18866 | 3.76E-06 | 2.37E-05 | 1.01E-05 | ALOX5/CAV1/CCL2/PPARG/TNF/XDH | 6 |
| GO:0010594 | regulation of endothelial cell migration | Sep-97 | 238/18866 | 3.79E-06 | 2.38E-05 | 1.02E-05 | AKT1/HMOX1/HSPB1/NOS3/PPARG/PTGS2/TGFB1/TNF/VEGFA | 9 |
| GO:1902930 | regulation of alcohol biosynthetic process | Jun-97 | 82/18866 | 4.04E-06 | 2.53E-05 | 1.08E-05 | APOB/IFNG/IL1B/SOD1/SREBF1/TNF | 6 |
| GO:0030225 | macrophage differentiation | May-97 | 47/18866 | 4.19E-06 | 2.62E-05 | 1.12E-05 | ADIPOQ/IFNG/MMP9/TGFB1/VEGFA | 5 |
| GO:0006909 | phagocytosis | Nov-97 | 382/18866 | 4.25E-06 | 2.66E-05 | 1.14E-05 | ADIPOQ/CCL2/CRP/IFNG/IL1B/MAPK1/MAPK3/PPARG/PTEN/SOD1/TNF | 11 |
| GO:0002718 | regulation of cytokine production involved in immune response | Jun-97 | 83/18866 | 4.33E-06 | 2.70E-05 | 1.15E-05 | HMOX1/IL10/IL1B/IL6/TGFB1/TNF | 6 |
| GO:0014855 | striated muscle cell proliferation | Jun-97 | 83/18866 | 4.33E-06 | 2.70E-05 | 1.15E-05 | MAPK1/PIM1/PPARD/PTEN/RXRA/STAT3 | 6 |
| GO:0014013 | regulation of gliogenesis | Jul-97 | 128/18866 | 4.34E-06 | 2.70E-05 | 1.15E-05 | CTNNB1/F2/IL1B/IL6/PPARG/TGFB1/TNF | 7 |
| GO:0001959 | regulation of cytokine-mediated signaling pathway | Aug-97 | 183/18866 | 4.66E-06 | 2.89E-05 | 1.23E-05 | ADIPOQ/CAV1/GSTP1/IFNG/IKBKB/IL6/PPARG/TNF | 8 |
| GO:0072330 | monocarboxylic acid biosynthetic process | Sep-97 | 246/18866 | 4.96E-06 | 3.07E-05 | 1.31E-05 | ADIPOQ/ALOX5/CYP1A1/CYP1A2/CYP3A4/GSTM1/GSTP1/IL1B/PTGS2 | 9 |
| GO:0010507 | negative regulation of autophagy | Jun-97 | 85/18866 | 4.98E-06 | 3.07E-05 | 1.31E-05 | AKT1/BCL2/HMOX1/IL10/STAT3/TP53 | 6 |
| GO:0043154 | negative regulation of cysteine-type endopeptidase activity involved in apoptotic process | Jun-97 | 85/18866 | 4.98E-06 | 3.07E-05 | 1.31E-05 | AKT1/BIRC5/MMP9/PTGS2/TNF/VEGFA | 6 |
| GO:0045844 | positive regulation of striated muscle tissue development | Jun-97 | 85/18866 | 4.98E-06 | 3.07E-05 | 1.31E-05 | BCL2/CTNNB1/ERBB3/MAPK1/PIM1/TGFB1 | 6 |
| GO:0048636 | positive regulation of muscle organ development | Jun-97 | 85/18866 | 4.98E-06 | 3.07E-05 | 1.31E-05 | BCL2/CTNNB1/ERBB3/MAPK1/PIM1/TGFB1 | 6 |
| GO:2000045 | regulation of G1/S transition of mitotic cell cycle | Aug-97 | 185/18866 | 5.05E-06 | 3.11E-05 | 1.32E-05 | AKT1/BCL2/CCL2/CDKN1A/CYP1A1/EGFR/PTEN/TP53 | 8 |
| GO:0002686 | negative regulation of leukocyte migration | May-97 | 49/18866 | 5.16E-06 | 3.17E-05 | 1.35E-05 | AKT1/CCL2/CYP19A1/DPP4/HMOX1 | 5 |
| GO:0010712 | regulation of collagen metabolic process | May-97 | 49/18866 | 5.16E-06 | 3.17E-05 | 1.35E-05 | F2/IL6/PPARD/PPARG/TGFB1 | 5 |
| GO:0010595 | positive regulation of endothelial cell migration | Jul-97 | 132/18866 | 5.33E-06 | 3.24E-05 | 1.38E-05 | AKT1/HMOX1/HSPB1/NOS3/PTGS2/TGFB1/VEGFA | 7 |
| GO:0014910 | regulation of smooth muscle cell migration | Jun-97 | 86/18866 | 5.33E-06 | 3.24E-05 | 1.38E-05 | ADIPOQ/BCL2/GSTP1/IGFBP3/PPARD/SERPINE1 | 6 |
| GO:0070664 | negative regulation of leukocyte proliferation | Jun-97 | 86/18866 | 5.33E-06 | 3.24E-05 | 1.38E-05 | CASP3/CRP/ERBB2/GSTP1/IL10/IL2 | 6 |
| GO:0097756 | negative regulation of blood vessel diameter | Jun-97 | 86/18866 | 5.33E-06 | 3.24E-05 | 1.38E-05 | AKT1/CAV1/CRP/EGFR/ICAM1/PTGS2 | 6 |
| GO:1901863 | positive regulation of muscle tissue development | Jun-97 | 86/18866 | 5.33E-06 | 3.24E-05 | 1.38E-05 | BCL2/CTNNB1/ERBB3/MAPK1/PIM1/TGFB1 | 6 |
| GO:0031281 | positive regulation of cyclase activity | Apr-97 | 23/18866 | 5.39E-06 | 3.27E-05 | 1.39E-05 | MAPK3/MAPK8/NOS2/NOS3 | 4 |
| GO:0051043 | regulation of membrane protein ectodomain proteolysis | Apr-97 | 23/18866 | 5.39E-06 | 3.27E-05 | 1.39E-05 | IFNG/IL10/IL1B/TNF | 4 |
| GO:0001774 | microglial cell activation | May-97 | 50/18866 | 5.71E-06 | 3.43E-05 | 1.46E-05 | IFNG/IL4/IL6/JUN/TNF | 5 |
| GO:0002269 | leukocyte activation involved in inflammatory response | May-97 | 50/18866 | 5.71E-06 | 3.43E-05 | 1.46E-05 | IFNG/IL4/IL6/JUN/TNF | 5 |
| GO:0030195 | negative regulation of blood coagulation | May-97 | 50/18866 | 5.71E-06 | 3.43E-05 | 1.46E-05 | F2/NOS3/PLAT/SERPINE1/THBD | 5 |
| GO:0045981 | positive regulation of nucleotide metabolic process | May-97 | 50/18866 | 5.71E-06 | 3.43E-05 | 1.46E-05 | IFNG/IL4/NOS2/NOS3/STAT3 | 5 |
| GO:1900544 | positive regulation of purine nucleotide metabolic process | May-97 | 50/18866 | 5.71E-06 | 3.43E-05 | 1.46E-05 | IFNG/IL4/NOS2/NOS3/STAT3 | 5 |
| GO:1904707 | positive regulation of vascular associated smooth muscle cell proliferation | May-97 | 50/18866 | 5.71E-06 | 3.43E-05 | 1.46E-05 | IL10/JUN/MMP2/MMP9/TNF | 5 |
| GO:0034754 | cellular hormone metabolic process | Jul-97 | 134/18866 | 5.88E-06 | 3.53E-05 | 1.50E-05 | CYP19A1/CYP1A1/CYP1A2/CYP3A4/ESR1/HSD3B1/SPP1 | 7 |
| GO:0014068 | positive regulation of phosphatidylinositol 3-kinase signaling | Jun-97 | 88/18866 | 6.10E-06 | 3.64E-05 | 1.55E-05 | CAT/ERBB3/F2/PIK3CG/PPARD/TNF | 6 |
| GO:0032755 | positive regulation of interleukin-6 production | Jun-97 | 88/18866 | 6.10E-06 | 3.64E-05 | 1.55E-05 | IFNG/IL1A/IL1B/IL6/STAT3/TNF | 6 |
| GO:0046889 | positive regulation of lipid biosynthetic process | Jun-97 | 88/18866 | 6.10E-06 | 3.64E-05 | 1.55E-05 | AKT1/IFNG/IL1B/PTGS2/SREBF1/TNF | 6 |
| GO:0042176 | regulation of protein catabolic process | Nov-97 | 397/18866 | 6.13E-06 | 3.65E-05 | 1.56E-05 | AKT1/CAV1/EGF/EGFR/IFNG/IL10/IL1B/NOS2/ODC1/PTEN/TNF | 11 |
| GO:0030308 | negative regulation of cell growth | Aug-97 | 190/18866 | 6.14E-06 | 3.65E-05 | 1.56E-05 | BCL2/CDKN1A/PPARA/PPARD/PPARG/SPP1/TGFB1/TP53 | 8 |
| GO:0003158 | endothelium development | Jul-97 | 135/18866 | 6.18E-06 | 3.66E-05 | 1.56E-05 | CTNNB1/ICAM1/IKBKB/IL1B/TNF/VEGFA/XDH | 7 |
| GO:0048565 | digestive tract development | Jul-97 | 135/18866 | 6.18E-06 | 3.66E-05 | 1.56E-05 | BCL2/CDKN1A/CTNNB1/CXCL8/CYP1A1/EGFR/TNF | 7 |
| GO:0002204 | somatic recombination of immunoglobulin genes involved in immune response | May-97 | 51/18866 | 6.31E-06 | 3.70E-05 | 1.58E-05 | CD40LG/IL10/IL2/IL4/TGFB1 | 5 |
| GO:0002208 | somatic diversification of immunoglobulins involved in immune response | May-97 | 51/18866 | 6.31E-06 | 3.70E-05 | 1.58E-05 | CD40LG/IL10/IL2/IL4/TGFB1 | 5 |
| GO:0045190 | isotype switching | May-97 | 51/18866 | 6.31E-06 | 3.70E-05 | 1.58E-05 | CD40LG/IL10/IL2/IL4/TGFB1 | 5 |
| GO:0045912 | negative regulation of carbohydrate metabolic process | May-97 | 51/18866 | 6.31E-06 | 3.70E-05 | 1.58E-05 | ADIPOQ/PPARA/STAT3/TGFB1/TP53 | 5 |
| GO:0048146 | positive regulation of fibroblast proliferation | May-97 | 51/18866 | 6.31E-06 | 3.70E-05 | 1.58E-05 | CDKN1A/EGFR/ESR1/JUN/MYC | 5 |
| GO:0071675 | regulation of mononuclear cell migration | May-97 | 51/18866 | 6.31E-06 | 3.70E-05 | 1.58E-05 | IL4/MAPK1/MAPK3/SERPINE1/TNF | 5 |
| GO:1900047 | negative regulation of hemostasis | May-97 | 51/18866 | 6.31E-06 | 3.70E-05 | 1.58E-05 | F2/NOS3/PLAT/SERPINE1/THBD | 5 |
| GO:0045926 | negative regulation of growth | Sep-97 | 254/18866 | 6.43E-06 | 3.75E-05 | 1.60E-05 | BCL2/CDKN1A/PPARA/PPARD/PPARG/PTEN/SPP1/TGFB1/TP53 | 9 |
| GO:2000027 | regulation of animal organ morphogenesis | Sep-97 | 254/18866 | 6.43E-06 | 3.75E-05 | 1.60E-05 | AR/BCL2/CTNNB1/ESR1/MYC/PIM1/RXRA/TNF/VEGFA | 9 |
| GO:0002719 | negative regulation of cytokine production involved in immune response | Apr-97 | 24/18866 | 6.45E-06 | 3.75E-05 | 1.60E-05 | HMOX1/IL10/TGFB1/TNF | 4 |
| GO:0010869 | regulation of receptor biosynthetic process | Apr-97 | 24/18866 | 6.45E-06 | 3.75E-05 | 1.60E-05 | ADIPOQ/IFNG/PPARA/PPARG | 4 |
| GO:0050995 | negative regulation of lipid catabolic process | Apr-97 | 24/18866 | 6.45E-06 | 3.75E-05 | 1.60E-05 | AKT1/IL1B/PIK3CG/TNF | 4 |
| GO:0030183 | B cell differentiation | Jul-97 | 137/18866 | 6.81E-06 | 3.95E-05 | 1.68E-05 | BCL2/CD40LG/IL10/IL4/IL6/TP53/VCAM1 | 7 |
| GO:1903038 | negative regulation of leukocyte cell-cell adhesion | Jul-97 | 137/18866 | 6.81E-06 | 3.95E-05 | 1.68E-05 | AKT1/CASP3/ERBB2/IL10/IL2/IL4/PPARA | 7 |
| GO:0048260 | positive regulation of receptor-mediated endocytosis | May-97 | 52/18866 | 6.95E-06 | 4.02E-05 | 1.71E-05 | EGF/IL4/SELE/SERPINE1/VEGFA | 5 |
| GO:0071622 | regulation of granulocyte chemotaxis | May-97 | 52/18866 | 6.95E-06 | 4.02E-05 | 1.71E-05 | CXCL8/DPP4/IL4/MAPK1/MAPK3 | 5 |
| GO:0031334 | positive regulation of protein-containing complex assembly | Sep-97 | 257/18866 | 7.07E-06 | 4.08E-05 | 1.74E-05 | ESR1/ICAM1/IFNG/MMP1/MMP3/TGFB1/TNF/TP53/VEGFA | 9 |
| GO:0002705 | positive regulation of leukocyte mediated immunity | Jul-97 | 138/18866 | 7.14E-06 | 4.12E-05 | 1.75E-05 | IL1B/IL2/IL4/IL6/NOS2/TGFB1/TNF | 7 |
| GO:0006898 | receptor-mediated endocytosis | Oct-97 | 328/18866 | 7.26E-06 | 4.18E-05 | 1.78E-05 | ACHE/ALB/APOB/CAV1/CXCL8/EGF/IL4/SELE/SERPINE1/VEGFA | 10 |
| GO:1901617 | organic hydroxy compound biosynthetic process | Sep-97 | 258/18866 | 7.29E-06 | 4.19E-05 | 1.79E-05 | APOB/CYP19A1/CYP3A4/IFNG/IL1B/SOD1/SREBF1/TNF/TYR | 9 |
| GO:0034103 | regulation of tissue remodeling | Jun-97 | 91/18866 | 7.41E-06 | 4.25E-05 | 1.81E-05 | EGFR/IL2/IL6/SPP1/TGFB1/TP53 | 6 |
| GO:0032731 | positive regulation of interleukin-1 beta production | May-97 | 53/18866 | 7.64E-06 | 4.37E-05 | 1.86E-05 | HSPB1/IFNG/IL6/STAT3/TNF | 5 |
| GO:0032964 | collagen biosynthetic process | May-97 | 53/18866 | 7.64E-06 | 4.37E-05 | 1.86E-05 | F2/IL6/PPARD/PPARG/TGFB1 | 5 |
| GO:0050927 | positive regulation of positive chemotaxis | Apr-97 | 25/18866 | 7.65E-06 | 4.37E-05 | 1.86E-05 | CXCL8/F3/F7/VEGFA | 4 |
| GO:0060759 | regulation of response to cytokine stimulus | Aug-97 | 196/18866 | 7.72E-06 | 4.39E-05 | 1.87E-05 | ADIPOQ/CAV1/GSTP1/IFNG/IKBKB/IL6/PPARG/TNF | 8 |
| GO:0071897 | DNA biosynthetic process | Aug-97 | 196/18866 | 7.72E-06 | 4.39E-05 | 1.87E-05 | ADIPOQ/CDKN1A/CTNNB1/MAPK1/MAPK3/MYC/PPARG/TP53 | 8 |
| GO:0007517 | muscle organ development | Nov-97 | 407/18866 | 7.76E-06 | 4.41E-05 | 1.88E-05 | BCL2/CAV1/CTNNB1/ERBB3/FOS/MAPK1/PIM1/PPARA/PTEN/RXRA/TGFB1 | 11 |
| GO:0031058 | positive regulation of histone modification | Jun-97 | 92/18866 | 7.89E-06 | 4.48E-05 | 1.91E-05 | CTNNB1/IL1B/MAPK3/SREBF1/TP53/VEGFA | 6 |
| GO:2000117 | negative regulation of cysteine-type endopeptidase activity | Jun-97 | 93/18866 | 8.40E-06 | 4.76E-05 | 2.03E-05 | AKT1/BIRC5/MMP9/PTGS2/TNF/VEGFA | 6 |
| GO:0019430 | removal of superoxide radicals | Apr-97 | 26/18866 | 9.00E-06 | 5.05E-05 | 2.15E-05 | MPO/NOS3/SOD1/TNF | 4 |
| GO:0030810 | positive regulation of nucleotide biosynthetic process | Apr-97 | 26/18866 | 9.00E-06 | 5.05E-05 | 2.15E-05 | IL4/NOS2/NOS3/STAT3 | 4 |
| GO:0034114 | regulation of heterotypic cell-cell adhesion | Apr-97 | 26/18866 | 9.00E-06 | 5.05E-05 | 2.15E-05 | ADIPOQ/IL10/IL1B/TNF | 4 |
| GO:0035902 | response to immobilization stress | Apr-97 | 26/18866 | 9.00E-06 | 5.05E-05 | 2.15E-05 | CYP1A1/CYP1A2/FOS/PPARG | 4 |
| GO:0042730 | fibrinolysis | Apr-97 | 26/18866 | 9.00E-06 | 5.05E-05 | 2.15E-05 | F2/PLAT/SERPINE1/THBD | 4 |
| GO:0050926 | regulation of positive chemotaxis | Apr-97 | 26/18866 | 9.00E-06 | 5.05E-05 | 2.15E-05 | CXCL8/F3/F7/VEGFA | 4 |
| GO:1900373 | positive regulation of purine nucleotide biosynthetic process | Apr-97 | 26/18866 | 9.00E-06 | 5.05E-05 | 2.15E-05 | IL4/NOS2/NOS3/STAT3 | 4 |
| GO:0050819 | negative regulation of coagulation | May-97 | 55/18866 | 9.19E-06 | 5.15E-05 | 2.19E-05 | F2/NOS3/PLAT/SERPINE1/THBD | 5 |
| GO:0010952 | positive regulation of peptidase activity | Aug-97 | 201/18866 | 9.28E-06 | 5.19E-05 | 2.21E-05 | CAV1/F3/MAPK3/MYC/PPARG/STAT3/TNF/XDH | 8 |
| GO:0006720 | isoprenoid metabolic process | Jul-97 | 144/18866 | 9.44E-06 | 5.27E-05 | 2.25E-05 | APOB/CYP1A1/CYP1A2/CYP3A4/EGFR/PPARD/RHO | 7 |
| GO:1990778 | protein localization to cell periphery | Oct-97 | 340/18866 | 9.93E-06 | 5.54E-05 | 2.36E-05 | ADIPOQ/AKT1/AR/BCL2L1/CAV1/EGFR/IFNG/IKBKB/TGFB1/TNF | 10 |
| GO:0002712 | regulation of B cell mediated immunity | May-97 | 56/18866 | 1.01E-05 | 5.55E-05 | 2.37E-05 | IL10/IL2/IL4/TGFB1/TNF | 5 |
| GO:0002889 | regulation of immunoglobulin mediated immune response | May-97 | 56/18866 | 1.01E-05 | 5.55E-05 | 2.37E-05 | IL10/IL2/IL4/TGFB1/TNF | 5 |
| GO:0010332 | response to gamma radiation | May-97 | 56/18866 | 1.01E-05 | 5.55E-05 | 2.37E-05 | BCL2/BCL2L1/CDKN1A/MYC/TP53 | 5 |
| GO:0016447 | somatic recombination of immunoglobulin gene segments | May-97 | 56/18866 | 1.01E-05 | 5.55E-05 | 2.37E-05 | CD40LG/IL10/IL2/IL4/TGFB1 | 5 |
| GO:0090183 | regulation of kidney development | May-97 | 56/18866 | 1.01E-05 | 5.55E-05 | 2.37E-05 | ADIPOQ/CTNNB1/MMP9/MYC/VEGFA | 5 |
| GO:1904645 | response to amyloid-beta | May-97 | 56/18866 | 1.01E-05 | 5.55E-05 | 2.37E-05 | ICAM1/MMP2/MMP3/MMP9/VCAM1 | 5 |
| GO:0006706 | steroid catabolic process | Apr-97 | 27/18866 | 1.05E-05 | 5.79E-05 | 2.47E-05 | CYP19A1/CYP1A2/CYP3A4/SPP1 | 4 |
| GO:0033081 | regulation of T cell differentiation in thymus | Apr-97 | 27/18866 | 1.05E-05 | 5.79E-05 | 2.47E-05 | ERBB2/IL1A/IL1B/SOD1 | 4 |
| GO:0072539 | T-helper 17 cell differentiation | Apr-97 | 27/18866 | 1.05E-05 | 5.79E-05 | 2.47E-05 | IL2/IL4/IL6/STAT3 | 4 |
| GO:0032651 | regulation of interleukin-1 beta production | Jun-97 | 97/18866 | 1.07E-05 | 5.88E-05 | 2.51E-05 | GSTP1/HSPB1/IFNG/IL6/STAT3/TNF | 6 |
| GO:0055123 | digestive system development | Jul-97 | 147/18866 | 1.08E-05 | 5.93E-05 | 2.53E-05 | BCL2/CDKN1A/CTNNB1/CXCL8/CYP1A1/EGFR/TNF | 7 |
| GO:1902806 | regulation of cell cycle G1/S phase transition | Aug-97 | 206/18866 | 1.11E-05 | 6.07E-05 | 2.59E-05 | AKT1/BCL2/CCL2/CDKN1A/CYP1A1/EGFR/PTEN/TP53 | 8 |
| GO:0050810 | regulation of steroid biosynthetic process | Jun-97 | 98/18866 | 1.14E-05 | 6.20E-05 | 2.64E-05 | APOB/IFNG/IL1B/SOD1/SREBF1/TNF | 6 |
| GO:2001243 | negative regulation of intrinsic apoptotic signaling pathway | Jun-97 | 98/18866 | 1.14E-05 | 6.20E-05 | 2.64E-05 | AKT1/BCL2/BCL2L1/HSPB1/MMP9/PTGS2 | 6 |
| GO:0031056 | regulation of histone modification | Jul-97 | 149/18866 | 1.18E-05 | 6.43E-05 | 2.74E-05 | CTNNB1/IL1B/MAPK3/MAPK8/SREBF1/TP53/VEGFA | 7 |
| GO:0002763 | positive regulation of myeloid leukocyte differentiation | May-97 | 58/18866 | 1.20E-05 | 6.49E-05 | 2.77E-05 | FOS/IFNG/JUN/TGFB1/TNF | 5 |
| GO:0019369 | arachidonic acid metabolic process | May-97 | 58/18866 | 1.20E-05 | 6.49E-05 | 2.77E-05 | ALOX5/CYP1A1/CYP1A2/MAPK3/PTGS2 | 5 |
| GO:0031294 | lymphocyte costimulation | May-97 | 58/18866 | 1.20E-05 | 6.49E-05 | 2.77E-05 | AKT1/CAV1/CD40LG/DPP4/IL4 | 5 |
| GO:0042742 | defense response to bacterium | Oct-97 | 348/18866 | 1.22E-05 | 6.57E-05 | 2.80E-05 | CRP/F2/IL10/IL6/LYZ/MPO/NOS2/SERPINE1/SLPI/TNF | 10 |
| GO:0002360 | T cell lineage commitment | Apr-97 | 28/18866 | 1.22E-05 | 6.57E-05 | 2.80E-05 | BCL2/IL6/STAT3/TP53 | 4 |
| GO:0071450 | cellular response to oxygen radical | Apr-97 | 28/18866 | 1.22E-05 | 6.57E-05 | 2.80E-05 | MPO/NOS3/SOD1/TNF | 4 |
| GO:0071451 | cellular response to superoxide | Apr-97 | 28/18866 | 1.22E-05 | 6.57E-05 | 2.80E-05 | MPO/NOS3/SOD1/TNF | 4 |
| GO:0072376 | protein activation cascade | Apr-97 | 28/18866 | 1.22E-05 | 6.57E-05 | 2.80E-05 | F10/F2/F3/F7 | 4 |
| GO:0072378 | blood coagulation, fibrin clot formation | Apr-97 | 28/18866 | 1.22E-05 | 6.57E-05 | 2.80E-05 | F10/F2/F3/F7 | 4 |
| GO:1902932 | positive regulation of alcohol biosynthetic process | Apr-97 | 28/18866 | 1.22E-05 | 6.57E-05 | 2.80E-05 | IFNG/IL1B/SREBF1/TNF | 4 |
| GO:0045580 | regulation of T cell differentiation | Jul-97 | 150/18866 | 1.23E-05 | 6.61E-05 | 2.82E-05 | ERBB2/IFNG/IL1A/IL1B/IL2/IL4/SOD1 | 7 |
| GO:0045807 | positive regulation of endocytosis | Jun-97 | 100/18866 | 1.28E-05 | 6.84E-05 | 2.91E-05 | EGF/IL4/PPARG/SELE/SERPINE1/VEGFA | 6 |
| GO:0006636 | unsaturated fatty acid biosynthetic process | May-97 | 59/18866 | 1.30E-05 | 6.95E-05 | 2.96E-05 | ALOX5/GSTM1/GSTP1/IL1B/PTGS2 | 5 |
| GO:0043525 | positive regulation of neuron apoptotic process | May-97 | 59/18866 | 1.30E-05 | 6.95E-05 | 2.96E-05 | CASP3/CTNNB1/JUN/TNF/TP53 | 5 |
| GO:0015908 | fatty acid transport | Jun-97 | 101/18866 | 1.35E-05 | 7.19E-05 | 3.06E-05 | AKT1/IL1B/NOS2/PPARA/PPARD/PPARG | 6 |
| GO:0042116 | macrophage activation | Jun-97 | 101/18866 | 1.35E-05 | 7.19E-05 | 3.06E-05 | IFNG/IL10/IL4/IL6/JUN/TNF | 6 |
| GO:0045639 | positive regulation of myeloid cell differentiation | Jun-97 | 101/18866 | 1.35E-05 | 7.19E-05 | 3.06E-05 | FOS/IFNG/JUN/STAT3/TGFB1/TNF | 6 |
| GO:0030278 | regulation of ossification | Aug-97 | 212/18866 | 1.37E-05 | 7.25E-05 | 3.09E-05 | ALOX5/BCL2/CTNNB1/IL6/MAPK1/MAPK3/TGFB1/TNF | 8 |
| GO:0051701 | interaction with host | Aug-97 | 212/18866 | 1.37E-05 | 7.25E-05 | 3.09E-05 | BCL2L1/CAV1/CTNNB1/CXCL8/DPP4/EGFR/ICAM1/RXRA | 8 |
| GO:0002822 | regulation of adaptive immune response based on somatic recombination of immune receptors built from immunoglobulin superfamily domains | Jul-97 | 153/18866 | 1.40E-05 | 7.39E-05 | 3.15E-05 | IL10/IL1B/IL2/IL4/IL6/TGFB1/TNF | 7 |
| GO:0006913 | nucleocytoplasmic transport | Oct-97 | 354/18866 | 1.41E-05 | 7.39E-05 | 3.15E-05 | AKT1/CDKN1A/IFNG/IL1B/MAPK1/NFKBIA/PTGS2/STAT3/TGFB1/TP53 | 10 |
| GO:0007263 | nitric oxide mediated signal transduction | Apr-97 | 29/18866 | 1.41E-05 | 7.39E-05 | 3.15E-05 | EGFR/NOS2/NOS3/VEGFA | 4 |
| GO:0031063 | regulation of histone deacetylation | Apr-97 | 29/18866 | 1.41E-05 | 7.39E-05 | 3.15E-05 | MAPK8/SREBF1/TP53/VEGFA | 4 |
| GO:0032373 | positive regulation of sterol transport | Apr-97 | 29/18866 | 1.41E-05 | 7.39E-05 | 3.15E-05 | ADIPOQ/CAV1/NFKBIA/PON1 | 4 |
| GO:0032376 | positive regulation of cholesterol transport | Apr-97 | 29/18866 | 1.41E-05 | 7.39E-05 | 3.15E-05 | ADIPOQ/CAV1/NFKBIA/PON1 | 4 |
| GO:0019229 | regulation of vasoconstriction | May-97 | 60/18866 | 1.41E-05 | 7.39E-05 | 3.15E-05 | AKT1/CAV1/EGFR/ICAM1/PTGS2 | 5 |
| GO:0032732 | positive regulation of interleukin-1 production | May-97 | 60/18866 | 1.41E-05 | 7.39E-05 | 3.15E-05 | HSPB1/IFNG/IL6/STAT3/TNF | 5 |
| GO:0050994 | regulation of lipid catabolic process | May-97 | 60/18866 | 1.41E-05 | 7.39E-05 | 3.15E-05 | AKT1/IL1B/PIK3CG/PPARA/TNF | 5 |
| GO:0097755 | positive regulation of blood vessel diameter | May-97 | 60/18866 | 1.41E-05 | 7.39E-05 | 3.15E-05 | EGFR/HMOX1/NOS3/PPARD/SOD1 | 5 |
| GO:0055024 | regulation of cardiac muscle tissue development | Jun-97 | 102/18866 | 1.43E-05 | 7.46E-05 | 3.18E-05 | ERBB3/MAPK1/PIM1/PPARA/PTEN/TGFB1 | 6 |
| GO:0072659 | protein localization to plasma membrane | Sep-97 | 281/18866 | 1.44E-05 | 7.53E-05 | 3.21E-05 | ADIPOQ/AKT1/AR/BCL2L1/EGFR/IFNG/IKBKB/TGFB1/TNF | 9 |
| GO:0002706 | regulation of lymphocyte mediated immunity | Jul-97 | 154/18866 | 1.46E-05 | 7.62E-05 | 3.25E-05 | IL10/IL1B/IL2/IL4/IL6/TGFB1/TNF | 7 |
| GO:0008630 | intrinsic apoptotic signaling pathway in response to DNA damage | Jun-97 | 103/18866 | 1.51E-05 | 7.84E-05 | 3.34E-05 | BCL2/BCL2L1/CDKN1A/HMOX1/TNF/TP53 | 6 |
| GO:0070498 | interleukin-1-mediated signaling pathway | Jun-97 | 103/18866 | 1.51E-05 | 7.84E-05 | 3.34E-05 | IKBKB/IL1A/IL1B/IL6/MAPK3/NFKBIA | 6 |
| GO:0070374 | positive regulation of ERK1 and ERK2 cascade | Aug-97 | 215/18866 | 1.51E-05 | 7.84E-05 | 3.34E-05 | CCL2/EGFR/ICAM1/JUN/MAPK3/PTEN/TGFB1/TNF | 8 |
| GO:0051169 | nuclear transport | Oct-97 | 357/18866 | 1.52E-05 | 7.85E-05 | 3.35E-05 | AKT1/CDKN1A/IFNG/IL1B/MAPK1/NFKBIA/PTGS2/STAT3/TGFB1/TP53 | 10 |
| GO:0002381 | immunoglobulin production involved in immunoglobulin mediated immune response | May-97 | 61/18866 | 1.53E-05 | 7.89E-05 | 3.36E-05 | CD40LG/IL10/IL2/IL4/TGFB1 | 5 |
| GO:0010574 | regulation of vascular endothelial growth factor production | May-97 | 61/18866 | 1.53E-05 | 7.89E-05 | 3.36E-05 | IL1A/IL1B/IL6/PTGS2/TGFB1 | 5 |
| GO:0033619 | membrane protein proteolysis | May-97 | 61/18866 | 1.53E-05 | 7.89E-05 | 3.36E-05 | IFNG/IL10/IL1B/TGFB1/TNF | 5 |
| GO:0090303 | positive regulation of wound healing | May-97 | 61/18866 | 1.53E-05 | 7.89E-05 | 3.36E-05 | F2/F3/F7/SERPINE1/THBD | 5 |
| GO:0010887 | negative regulation of cholesterol storage | Mar-97 | 10/18866 | 1.54E-05 | 7.89E-05 | 3.36E-05 | PPARA/PPARD/PPARG | 3 |
| GO:0033083 | regulation of immature T cell proliferation | Mar-97 | 10/18866 | 1.54E-05 | 7.89E-05 | 3.36E-05 | ERBB2/IL1A/IL1B | 3 |
| GO:0045348 | positive regulation of MHC class II biosynthetic process | Mar-97 | 10/18866 | 1.54E-05 | 7.89E-05 | 3.36E-05 | IFNG/IL10/IL4 | 3 |
| GO:0051918 | negative regulation of fibrinolysis | Mar-97 | 10/18866 | 1.54E-05 | 7.89E-05 | 3.36E-05 | F2/SERPINE1/THBD | 3 |
| GO:0000187 | activation of MAPK activity | Jul-97 | 156/18866 | 1.59E-05 | 8.14E-05 | 3.47E-05 | CD40LG/EGF/IL1B/MAPK1/MAPK3/SOD1/TNF | 7 |
| GO:0000303 | response to superoxide | Apr-97 | 30/18866 | 1.62E-05 | 8.27E-05 | 3.53E-05 | MPO/NOS3/SOD1/TNF | 4 |
| GO:0048147 | negative regulation of fibroblast proliferation | Apr-97 | 30/18866 | 1.62E-05 | 8.27E-05 | 3.53E-05 | GSTP1/MYC/PPARG/TP53 | 4 |
| GO:0060055 | angiogenesis involved in wound healing | Apr-97 | 30/18866 | 1.62E-05 | 8.27E-05 | 3.53E-05 | ALOX5/SERPINE1/TNF/VEGFA | 4 |
| GO:0002294 | CD4-positive, alpha-beta T cell differentiation involved in immune response | May-97 | 62/18866 | 1.66E-05 | 8.43E-05 | 3.59E-05 | IFNG/IL2/IL4/IL6/STAT3 | 5 |
| GO:0034113 | heterotypic cell-cell adhesion | May-97 | 62/18866 | 1.66E-05 | 8.43E-05 | 3.59E-05 | ADIPOQ/IL10/IL1B/TNF/VCAM1 | 5 |
| GO:2001244 | positive regulation of intrinsic apoptotic signaling pathway | May-97 | 62/18866 | 1.66E-05 | 8.43E-05 | 3.59E-05 | BCL2/BCL2L1/CAV1/SOD1/TP53 | 5 |
| GO:0002824 | positive regulation of adaptive immune response based on somatic recombination of immune receptors built from immunoglobulin superfamily domains | Jun-97 | 105/18866 | 1.69E-05 | 8.53E-05 | 3.63E-05 | IL1B/IL2/IL4/IL6/TGFB1/TNF | 6 |
| GO:0030301 | cholesterol transport | Jun-97 | 105/18866 | 1.69E-05 | 8.53E-05 | 3.63E-05 | ADIPOQ/APOB/CAV1/EGF/NFKBIA/PON1 | 6 |
| GO:1902106 | negative regulation of leukocyte differentiation | Jun-97 | 105/18866 | 1.69E-05 | 8.53E-05 | 3.63E-05 | ADIPOQ/CTNNB1/ERBB2/IL2/IL4/MYC | 6 |
| GO:0000082 | G1/S transition of mitotic cell cycle | Sep-97 | 287/18866 | 1.71E-05 | 8.61E-05 | 3.67E-05 | AKT1/BCL2/CCL2/CDKN1A/CYP1A1/EGFR/MYC/PTEN/TP53 | 9 |
| GO:0035051 | cardiocyte differentiation | Jul-97 | 158/18866 | 1.73E-05 | 8.70E-05 | 3.71E-05 | EGFR/MAPK1/MAPK3/PPARA/RXRA/TGFB1/VEGFA | 7 |
| GO:1903707 | negative regulation of hemopoiesis | Jul-97 | 158/18866 | 1.73E-05 | 8.70E-05 | 3.71E-05 | ADIPOQ/CTNNB1/ERBB2/IL2/IL4/MYC/NFKBIA | 7 |
| GO:0002708 | positive regulation of lymphocyte mediated immunity | Jun-97 | 106/18866 | 1.78E-05 | 8.93E-05 | 3.81E-05 | IL1B/IL2/IL4/IL6/TGFB1/TNF | 6 |
| GO:0046632 | alpha-beta T cell differentiation | Jun-97 | 106/18866 | 1.78E-05 | 8.93E-05 | 3.81E-05 | BCL2/IFNG/IL2/IL4/IL6/STAT3 | 6 |
| GO:1905269 | positive regulation of chromatin organization | Jun-97 | 106/18866 | 1.78E-05 | 8.93E-05 | 3.81E-05 | CTNNB1/IL1B/MAPK3/SREBF1/TP53/VEGFA | 6 |
| GO:0002287 | alpha-beta T cell activation involved in immune response | May-97 | 63/18866 | 1.80E-05 | 8.97E-05 | 3.83E-05 | IFNG/IL2/IL4/IL6/STAT3 | 5 |
| GO:0002293 | alpha-beta T cell differentiation involved in immune response | May-97 | 63/18866 | 1.80E-05 | 8.97E-05 | 3.83E-05 | IFNG/IL2/IL4/IL6/STAT3 | 5 |
| GO:0032675 | regulation of interleukin-6 production | Jul-97 | 159/18866 | 1.80E-05 | 8.97E-05 | 3.83E-05 | IFNG/IL10/IL1A/IL1B/IL6/STAT3/TNF | 7 |
| GO:0050777 | negative regulation of immune response | Jul-97 | 159/18866 | 1.80E-05 | 8.97E-05 | 3.83E-05 | HMOX1/IL10/IL2/IL4/PPARG/TGFB1/TNF | 7 |
| GO:0000305 | response to oxygen radical | Apr-97 | 31/18866 | 1.86E-05 | 9.21E-05 | 3.93E-05 | MPO/NOS3/SOD1/TNF | 4 |
| GO:0032770 | positive regulation of monooxygenase activity | Apr-97 | 31/18866 | 1.86E-05 | 9.21E-05 | 3.93E-05 | AKT1/IFNG/IL1B/TNF | 4 |
| GO:0045940 | positive regulation of steroid metabolic process | Apr-97 | 31/18866 | 1.86E-05 | 9.21E-05 | 3.93E-05 | IFNG/IL1B/SREBF1/TNF | 4 |
| GO:0072538 | T-helper 17 type immune response | Apr-97 | 31/18866 | 1.86E-05 | 9.21E-05 | 3.93E-05 | IL2/IL4/IL6/STAT3 | 4 |
| GO:0002449 | lymphocyte mediated immunity | Oct-97 | 366/18866 | 1.88E-05 | 9.30E-05 | 3.96E-05 | CD40LG/CRP/ICAM1/IL10/IL1B/IL2/IL4/IL6/TGFB1/TNF | 10 |
| GO:0032409 | regulation of transporter activity | Sep-97 | 291/18866 | 1.91E-05 | 9.42E-05 | 4.02E-05 | BCL2/CAV1/CCL2/IFNG/MMP9/PIM1/PON1/PPARG/PTEN | 9 |
| GO:0001523 | retinoid metabolic process | Jun-97 | 108/18866 | 1.98E-05 | 9.77E-05 | 4.17E-05 | APOB/CYP1A1/CYP1A2/CYP3A4/PPARD/RHO | 6 |
| GO:0008593 | regulation of Notch signaling pathway | Jun-97 | 108/18866 | 1.98E-05 | 9.77E-05 | 4.17E-05 | AKT1/EGF/EGFR/NFKBIA/NOS3/STAT3 | 6 |
| GO:0043467 | regulation of generation of precursor metabolites and energy | Jul-97 | 162/18866 | 2.03E-05 | 0.0001 | 4.27E-05 | AKT1/IFNG/IL4/NOS2/PPARA/STAT3/TP53 | 7 |
| GO:0044282 | small molecule catabolic process | Nov-97 | 452/18866 | 2.06E-05 | 0.0001 | 4.32E-05 | ADIPOQ/AKT1/CYP1A1/NOS2/NOS3/PON1/PPARA/PPARD/PTEN/TGFB1/TP53 | 11 |
| GO:0048645 | animal organ formation | May-97 | 65/18866 | 2.10E-05 | 0.0001 | 4.39E-05 | AR/CTNNB1/MAPK1/MAPK3/PIM1 | 5 |
| GO:0033079 | immature T cell proliferation | Mar-97 | 11/18866 | 2.11E-05 | 0.0001 | 4.39E-05 | ERBB2/IL1A/IL1B | 3 |
| GO:0060439 | trachea morphogenesis | Mar-97 | 11/18866 | 2.11E-05 | 0.0001 | 4.39E-05 | CTNNB1/MAPK1/MAPK3 | 3 |
| GO:0010165 | response to X-ray | Apr-97 | 32/18866 | 2.11E-05 | 0.0001 | 4.39E-05 | CASP3/CDKN1A/THBD/TP53 | 4 |
| GO:0038128 | ERBB2 signaling pathway | Apr-97 | 32/18866 | 2.11E-05 | 0.0001 | 4.39E-05 | EGF/EGFR/ERBB2/ERBB3 | 4 |
| GO:0097421 | liver regeneration | Apr-97 | 32/18866 | 2.11E-05 | 0.0001 | 4.39E-05 | EGFR/HMOX1/IL10/IL6 | 4 |
| GO:1901889 | negative regulation of cell junction assembly | Apr-97 | 32/18866 | 2.11E-05 | 0.0001 | 4.39E-05 | IKBKB/IL1B/PTEN/TNF | 4 |
| GO:0050768 | negative regulation of neurogenesis | Sep-97 | 295/18866 | 2.12E-05 | 0.0001 | 4.40E-05 | CTNNB1/F2/IL1B/IL6/PTEN/SPP1/STAT3/TNF/TP53 | 9 |
| GO:0050804 | modulation of chemical synaptic transmission | Nov-97 | 454/18866 | 2.15E-05 | 0.0001 | 4.45E-05 | ACHE/ADIPOQ/CCL2/EGFR/IL1B/MAPK1/PLAT/PTEN/PTGS2/STAT3/TNF | 11 |
| GO:0099177 | regulation of trans-synaptic signaling | Nov-97 | 455/18866 | 2.19E-05 | 0.00011 | 4.54E-05 | ACHE/ADIPOQ/CCL2/EGFR/IL1B/MAPK1/PLAT/PTEN/PTGS2/STAT3/TNF | 11 |
| GO:0046165 | alcohol biosynthetic process | Jul-97 | 164/18866 | 2.20E-05 | 0.00011 | 4.54E-05 | APOB/CYP3A4/IFNG/IL1B/SOD1/SREBF1/TNF | 7 |
| GO:0002821 | positive regulation of adaptive immune response | Jun-97 | 110/18866 | 2.20E-05 | 0.00011 | 4.54E-05 | IL1B/IL2/IL4/IL6/TGFB1/TNF | 6 |
| GO:0019932 | second-messenger-mediated signaling | Nov-97 | 456/18866 | 2.24E-05 | 0.00011 | 4.61E-05 | AHR/CXCL8/EGFR/ERBB3/NOS2/NOS3/PTGER3/SELE/TNF/VCAM1/VEGFA | 11 |
| GO:0002562 | somatic diversification of immune receptors via germline recombination within a single locus | May-97 | 66/18866 | 2.26E-05 | 0.00011 | 4.64E-05 | CD40LG/IL10/IL2/IL4/TGFB1 | 5 |
| GO:0016444 | somatic cell DNA recombination | May-97 | 66/18866 | 2.26E-05 | 0.00011 | 4.64E-05 | CD40LG/IL10/IL2/IL4/TGFB1 | 5 |
| GO:0016445 | somatic diversification of immunoglobulins | May-97 | 66/18866 | 2.26E-05 | 0.00011 | 4.64E-05 | CD40LG/IL10/IL2/IL4/TGFB1 | 5 |
| GO:0034440 | lipid oxidation | Jun-97 | 111/18866 | 2.32E-05 | 0.00011 | 4.75E-05 | ADIPOQ/AKT1/ALOX5/PPARA/PPARD/PPARG | 6 |
| GO:0048762 | mesenchymal cell differentiation | Aug-97 | 229/18866 | 2.38E-05 | 0.00011 | 4.88E-05 | BCL2/CTNNB1/IL1B/IL6/MAPK1/MAPK3/PTEN/TGFB1 | 8 |
| GO:0033028 | myeloid cell apoptotic process | Apr-97 | 33/18866 | 2.40E-05 | 0.00011 | 4.90E-05 | ADIPOQ/BCL2/IL6/PTEN | 4 |
| GO:0120255 | olefinic compound biosynthetic process | Apr-97 | 33/18866 | 2.40E-05 | 0.00011 | 4.90E-05 | ALOX5/CYP19A1/GSTM1/GSTP1 | 4 |
| GO:0045670 | regulation of osteoclast differentiation | May-97 | 67/18866 | 2.43E-05 | 0.00012 | 4.96E-05 | CTNNB1/FOS/IFNG/IL4/TNF | 5 |
| GO:0046822 | regulation of nucleocytoplasmic transport | Jun-97 | 112/18866 | 2.44E-05 | 0.00012 | 4.97E-05 | IFNG/IL1B/MAPK1/PTGS2/TGFB1/TP53 | 6 |
| GO:0001649 | osteoblast differentiation | Aug-97 | 231/18866 | 2.54E-05 | 0.00012 | 5.15E-05 | ACHE/AKT1/CAT/CTNNB1/IGFBP3/IL6/SPP1/TNF | 8 |
| GO:0048738 | cardiac muscle tissue development | Aug-97 | 231/18866 | 2.54E-05 | 0.00012 | 5.15E-05 | ERBB3/MAPK1/PIM1/PPARA/PTEN/RXRA/TGFB1/VEGFA | 8 |
| GO:0002819 | regulation of adaptive immune response | Jul-97 | 168/18866 | 2.57E-05 | 0.00012 | 5.21E-05 | IL10/IL1B/IL2/IL4/IL6/TGFB1/TNF | 7 |
| GO:1903522 | regulation of blood circulation | Sep-97 | 303/18866 | 2.62E-05 | 0.00012 | 5.31E-05 | AKT1/CAV1/EGFR/ICAM1/IL2/NOS3/PIK3CG/PTGS2/SREBF1 | 9 |
| GO:0033198 | response to ATP | Apr-97 | 34/18866 | 2.71E-05 | 0.00013 | 5.46E-05 | IL1B/PTEN/PTGS2/SOD1 | 4 |
| GO:0046685 | response to arsenic-containing substance | Apr-97 | 34/18866 | 2.71E-05 | 0.00013 | 5.46E-05 | CDKN1A/CYP1A1/HMOX1/PTEN | 4 |
| GO:0050715 | positive regulation of cytokine secretion | Apr-97 | 34/18866 | 2.71E-05 | 0.00013 | 5.46E-05 | IFNG/IL10/IL1A/TNF | 4 |
| GO:0060742 | epithelial cell differentiation involved in prostate gland development | Mar-97 | 12/18866 | 2.80E-05 | 0.00013 | 5.64E-05 | AR/CTNNB1/RXRA | 3 |
| GO:0072584 | caveolin-mediated endocytosis | Mar-97 | 12/18866 | 2.80E-05 | 0.00013 | 5.64E-05 | CAV1/MAPK1/MAPK3 | 3 |
| GO:0042698 | ovulation cycle | May-97 | 69/18866 | 2.81E-05 | 0.00013 | 5.64E-05 | CASP3/EGFR/ESR1/NOS3/PGR | 5 |
| GO:0002440 | production of molecular mediator of immune response | Sep-97 | 306/18866 | 2.83E-05 | 0.00013 | 5.67E-05 | CD40LG/HMOX1/IL10/IL1B/IL2/IL4/IL6/TGFB1/TNF | 9 |
| GO:0001654 | eye development | Oct-97 | 384/18866 | 2.83E-05 | 0.00013 | 5.67E-05 | ACHE/BCL2/CTNNB1/CYP1A1/EGFR/JUN/RHO/RXRA/STAT3/VEGFA | 10 |
| GO:0031623 | receptor internalization | Jun-97 | 115/18866 | 2.83E-05 | 0.00013 | 5.67E-05 | ACHE/CAV1/CXCL8/EGF/SELE/VEGFA | 6 |
| GO:0030307 | positive regulation of cell growth | Jul-97 | 171/18866 | 2.88E-05 | 0.00013 | 5.75E-05 | AKT1/BCL2/EGFR/ERBB2/F2/IL2/VEGFA | 7 |
| GO:0051302 | regulation of cell division | Jul-97 | 171/18866 | 2.88E-05 | 0.00013 | 5.75E-05 | BCL2L1/CAT/IL1A/IL1B/MYC/TGFB1/VEGFA | 7 |
| GO:0046620 | regulation of organ growth | Jun-97 | 116/18866 | 2.97E-05 | 0.00014 | 5.94E-05 | AKT1/MAPK1/PIM1/PPARA/PTEN/SOD1 | 6 |
| GO:0032374 | regulation of cholesterol transport | May-97 | 70/18866 | 3.01E-05 | 0.00014 | 6.00E-05 | ADIPOQ/CAV1/EGF/NFKBIA/PON1 | 5 |
| GO:0150063 | visual system development | Oct-97 | 388/18866 | 3.09E-05 | 0.00014 | 6.15E-05 | ACHE/BCL2/CTNNB1/CYP1A1/EGFR/JUN/RHO/RXRA/STAT3/VEGFA | 10 |
| GO:0033209 | tumor necrosis factor-mediated signaling pathway | Jul-97 | 173/18866 | 3.10E-05 | 0.00014 | 6.17E-05 | ADIPOQ/CD40LG/GSTP1/IKBKB/NFKBIA/TNF/TP53 | 7 |
| GO:0018107 | peptidyl-threonine phosphorylation | Jun-97 | 117/18866 | 3.12E-05 | 0.00015 | 6.20E-05 | AKT1/BCL2/EGF/MAPK1/MAPK8/TGFB1 | 6 |
| GO:0051346 | negative regulation of hydrolase activity | Nov-97 | 473/18866 | 3.13E-05 | 0.00015 | 6.21E-05 | AKT1/BIRC5/IKBKB/MMP9/NOS3/PTGS2/SERPINE1/SLPI/TNF/TP53/VEGFA | 11 |
| GO:0044843 | cell cycle G1/S phase transition | Sep-97 | 310/18866 | 3.13E-05 | 0.00015 | 6.21E-05 | AKT1/BCL2/CCL2/CDKN1A/CYP1A1/EGFR/MYC/PTEN/TP53 | 9 |
| GO:0015718 | monocarboxylic acid transport | Jul-97 | 174/18866 | 3.22E-05 | 0.00015 | 6.36E-05 | AKT1/IL1B/NOS2/PPARA/PPARD/PPARG/RXRA | 7 |
| GO:0002292 | T cell differentiation involved in immune response | May-97 | 71/18866 | 3.22E-05 | 0.00015 | 6.36E-05 | IFNG/IL2/IL4/IL6/STAT3 | 5 |
| GO:0061180 | mammary gland epithelium development | May-97 | 71/18866 | 3.22E-05 | 0.00015 | 6.36E-05 | AKT1/AR/ESR1/MAPK1/PGR | 5 |
| GO:0006805 | xenobiotic metabolic process | Jun-97 | 118/18866 | 3.27E-05 | 0.00015 | 6.46E-05 | AHR/CYP1A1/CYP1A2/CYP3A4/GSTM1/GSTP1 | 6 |
| GO:0002701 | negative regulation of production of molecular mediator of immune response | Apr-97 | 36/18866 | 3.41E-05 | 0.00016 | 6.70E-05 | HMOX1/IL10/TGFB1/TNF | 4 |
| GO:0033280 | response to vitamin D | Apr-97 | 36/18866 | 3.41E-05 | 0.00016 | 6.70E-05 | PIM1/PTGS2/SPP1/TYR | 4 |
| GO:0051973 | positive regulation of telomerase activity | Apr-97 | 36/18866 | 3.41E-05 | 0.00016 | 6.70E-05 | CTNNB1/MAPK1/MAPK3/MYC | 4 |
| GO:0045600 | positive regulation of fat cell differentiation | May-97 | 72/18866 | 3.45E-05 | 0.00016 | 6.77E-05 | AKT1/PIM1/PPARD/PPARG/PTGS2 | 5 |
| GO:0016241 | regulation of macroautophagy | Jul-97 | 176/18866 | 3.46E-05 | 0.00016 | 6.78E-05 | AKT1/CASP3/HMOX1/IL4/MAPK3/MAPK8/TP53 | 7 |
| GO:0048880 | sensory system development | Oct-97 | 394/18866 | 3.52E-05 | 0.00016 | 6.89E-05 | ACHE/BCL2/CTNNB1/CYP1A1/EGFR/JUN/RHO/RXRA/STAT3/VEGFA | 10 |
| GO:0002674 | negative regulation of acute inflammatory response | Mar-97 | 13/18866 | 3.63E-05 | 0.00017 | 7.06E-05 | GSTP1/IL4/PPARG | 3 |
| GO:0010623 | programmed cell death involved in cell development | Mar-97 | 13/18866 | 3.63E-05 | 0.00017 | 7.06E-05 | BCL2/IL1A/IL1B | 3 |
| GO:0030656 | regulation of vitamin metabolic process | Mar-97 | 13/18866 | 3.63E-05 | 0.00017 | 7.06E-05 | IFNG/IL1B/TNF | 3 |
| GO:0031953 | negative regulation of protein autophosphorylation | Mar-97 | 13/18866 | 3.63E-05 | 0.00017 | 7.06E-05 | ADIPOQ/CAV1/JUN | 3 |
| GO:0061043 | regulation of vascular wound healing | Mar-97 | 13/18866 | 3.63E-05 | 0.00017 | 7.06E-05 | ALOX5/SERPINE1/TNF | 3 |
| GO:0070227 | lymphocyte apoptotic process | May-97 | 73/18866 | 3.69E-05 | 0.00017 | 7.17E-05 | AKT1/IL10/IL2/PTEN/TP53 | 5 |
| GO:1900542 | regulation of purine nucleotide metabolic process | Jun-97 | 121/18866 | 3.77E-05 | 0.00017 | 7.32E-05 | IFNG/IL4/NOS2/NOS3/PPARA/STAT3 | 6 |
| GO:0045191 | regulation of isotype switching | Apr-97 | 37/18866 | 3.81E-05 | 0.00017 | 7.38E-05 | IL10/IL2/IL4/TGFB1 | 4 |
| GO:0051961 | negative regulation of nervous system development | Sep-97 | 319/18866 | 3.91E-05 | 0.00018 | 7.58E-05 | CTNNB1/F2/IL1B/IL6/PTEN/SPP1/STAT3/TNF/TP53 | 9 |
| GO:0050769 | positive regulation of neurogenesis | Nov-97 | 485/18866 | 3.93E-05 | 0.00018 | 7.60E-05 | BCL2/CTNNB1/IFNG/IL1B/IL2/IL6/PPARG/PTEN/TGFB1/TNF/VEGFA | 11 |
| GO:1903036 | positive regulation of response to wounding | May-97 | 74/18866 | 3.94E-05 | 0.00018 | 7.61E-05 | F2/F3/F7/SERPINE1/THBD | 5 |
| GO:0045619 | regulation of lymphocyte differentiation | Jul-97 | 181/18866 | 4.14E-05 | 0.00019 | 7.95E-05 | ERBB2/IFNG/IL1A/IL1B/IL2/IL4/SOD1 | 7 |
| GO:1905330 | regulation of morphogenesis of an epithelium | Jul-97 | 181/18866 | 4.14E-05 | 0.00019 | 7.95E-05 | AR/CTNNB1/ESR1/PTEN/RXRA/TNF/VEGFA | 7 |
| GO:0006140 | regulation of nucleotide metabolic process | Jun-97 | 123/18866 | 4.14E-05 | 0.00019 | 7.95E-05 | IFNG/IL4/NOS2/NOS3/PPARA/STAT3 | 6 |
| GO:0071466 | cellular response to xenobiotic stimulus | Jun-97 | 123/18866 | 4.14E-05 | 0.00019 | 7.95E-05 | AHR/CYP1A1/CYP1A2/CYP3A4/GSTM1/GSTP1 | 6 |
| GO:0045685 | regulation of glial cell differentiation | May-97 | 75/18866 | 4.20E-05 | 0.00019 | 8.06E-05 | CTNNB1/F2/IL6/PPARG/TGFB1 | 5 |
| GO:0050805 | negative regulation of synaptic transmission | May-97 | 75/18866 | 4.20E-05 | 0.00019 | 8.06E-05 | ACHE/ADIPOQ/IL1B/PTEN/PTGS2 | 5 |
| GO:0010661 | positive regulation of muscle cell apoptotic process | Apr-97 | 38/18866 | 4.24E-05 | 0.00019 | 8.07E-05 | IFNG/PPARG/PTEN/TP53 | 4 |
| GO:0030224 | monocyte differentiation | Apr-97 | 38/18866 | 4.24E-05 | 0.00019 | 8.07E-05 | JUN/MYC/PPARG/VEGFA | 4 |
| GO:0032733 | positive regulation of interleukin-10 production | Apr-97 | 38/18866 | 4.24E-05 | 0.00019 | 8.07E-05 | CD40LG/IL4/IL6/STAT3 | 4 |
| GO:0043029 | T cell homeostasis | Apr-97 | 38/18866 | 4.24E-05 | 0.00019 | 8.07E-05 | AKT1/BCL2/CASP3/IL2 | 4 |
| GO:1903131 | mononuclear cell differentiation | Apr-97 | 38/18866 | 4.24E-05 | 0.00019 | 8.07E-05 | JUN/MYC/PPARG/VEGFA | 4 |
| GO:2000144 | positive regulation of DNA-templated transcription, initiation | Apr-97 | 38/18866 | 4.24E-05 | 0.00019 | 8.07E-05 | CTNNB1/ESR1/JUN/TP53 | 4 |
| GO:0071346 | cellular response to interferon-gamma | Jul-97 | 182/18866 | 4.28E-05 | 0.00019 | 8.14E-05 | CCL2/ICAM1/IFNG/NOS2/PPARG/TP53/VCAM1 | 7 |
| GO:2001252 | positive regulation of chromosome organization | Jul-97 | 182/18866 | 4.28E-05 | 0.00019 | 8.14E-05 | CTNNB1/IL1B/MAPK1/MAPK3/SREBF1/TP53/VEGFA | 7 |
| GO:0002698 | negative regulation of immune effector process | Jun-97 | 124/18866 | 4.33E-05 | 0.00019 | 8.21E-05 | HMOX1/IL10/IL2/IL4/TGFB1/TNF | 6 |
| GO:0071229 | cellular response to acid chemical | May-97 | 76/18866 | 4.48E-05 | 0.0002 | 8.48E-05 | BCL2L1/EGFR/MMP2/TNF/VEGFA | 5 |
| GO:1903524 | positive regulation of blood circulation | May-97 | 76/18866 | 4.48E-05 | 0.0002 | 8.48E-05 | AKT1/CAV1/EGFR/ICAM1/PTGS2 | 5 |
| GO:0034605 | cellular response to heat | Jun-97 | 125/18866 | 4.53E-05 | 0.0002 | 8.56E-05 | CDKN1A/HMOX1/IL1A/MAPK1/MAPK3/PTGS2 | 6 |
| GO:0030213 | hyaluronan biosynthetic process | Mar-97 | 14/18866 | 4.60E-05 | 0.0002 | 8.67E-05 | EGF/IL1B/TGFB1 | 3 |
| GO:0051917 | regulation of fibrinolysis | Mar-97 | 14/18866 | 4.60E-05 | 0.0002 | 8.67E-05 | F2/SERPINE1/THBD | 3 |
| GO:0090399 | replicative senescence | Mar-97 | 14/18866 | 4.60E-05 | 0.0002 | 8.67E-05 | CDKN1A/SERPINE1/TP53 | 3 |
| GO:0032660 | regulation of interleukin-17 production | Apr-97 | 39/18866 | 4.70E-05 | 0.00021 | 8.84E-05 | IFNG/IL2/IL6/TGFB1 | 4 |
| GO:2000279 | negative regulation of DNA biosynthetic process | Apr-97 | 39/18866 | 4.70E-05 | 0.00021 | 8.84E-05 | ADIPOQ/CDKN1A/PPARG/TP53 | 4 |
| GO:0018210 | peptidyl-threonine modification | Jun-97 | 126/18866 | 4.74E-05 | 0.00021 | 8.89E-05 | AKT1/BCL2/EGF/MAPK1/MAPK8/TGFB1 | 6 |
| GO:0002200 | somatic diversification of immune receptors | May-97 | 77/18866 | 4.77E-05 | 0.00021 | 8.93E-05 | CD40LG/IL10/IL2/IL4/TGFB1 | 5 |
| GO:0014015 | positive regulation of gliogenesis | May-97 | 77/18866 | 4.77E-05 | 0.00021 | 8.93E-05 | IL1B/IL6/PPARG/TGFB1/TNF | 5 |
| GO:0046902 | regulation of mitochondrial membrane permeability | May-97 | 77/18866 | 4.77E-05 | 0.00021 | 8.93E-05 | BCL2/BCL2L1/MAPK8/STAT3/TP53 | 5 |
| GO:1900180 | regulation of protein localization to nucleus | Jun-97 | 127/18866 | 4.95E-05 | 0.00022 | 9.25E-05 | AKT1/F2/IFNG/MAPK1/PTGS2/TGFB1 | 6 |
| GO:0001895 | retina homeostasis | May-97 | 78/18866 | 5.08E-05 | 0.00022 | 9.45E-05 | ALB/HSPB1/LYZ/RHO/SOD1 | 5 |
| GO:0042310 | vasoconstriction | May-97 | 78/18866 | 5.08E-05 | 0.00022 | 9.45E-05 | AKT1/CAV1/EGFR/ICAM1/PTGS2 | 5 |
| GO:0043367 | CD4-positive, alpha-beta T cell differentiation | May-97 | 78/18866 | 5.08E-05 | 0.00022 | 9.45E-05 | IFNG/IL2/IL4/IL6/STAT3 | 5 |
| GO:0043409 | negative regulation of MAPK cascade | Jul-97 | 187/18866 | 5.09E-05 | 0.00022 | 9.45E-05 | ADIPOQ/AKT1/CAV1/GSTP1/IL1B/MYC/PTEN | 7 |
| GO:0071375 | cellular response to peptide hormone stimulus | Sep-97 | 330/18866 | 5.09E-05 | 0.00022 | 9.45E-05 | ADIPOQ/AKT1/CAV1/GSTP1/IL1B/PPARG/PTEN/SREBF1/STAT3 | 9 |
| GO:0034763 | negative regulation of transmembrane transport | Jun-97 | 128/18866 | 5.17E-05 | 0.00022 | 9.59E-05 | AKT1/CAV1/IL1B/MMP9/PTEN/TNF | 6 |
| GO:0002714 | positive regulation of B cell mediated immunity | Apr-97 | 40/18866 | 5.21E-05 | 0.00023 | 9.60E-05 | IL2/IL4/TGFB1/TNF | 4 |
| GO:0002891 | positive regulation of immunoglobulin mediated immune response | Apr-97 | 40/18866 | 5.21E-05 | 0.00023 | 9.60E-05 | IL2/IL4/TGFB1/TNF | 4 |
| GO:0042307 | positive regulation of protein import into nucleus | Apr-97 | 40/18866 | 5.21E-05 | 0.00023 | 9.60E-05 | IFNG/MAPK1/PTGS2/TGFB1 | 4 |
| GO:0045746 | negative regulation of Notch signaling pathway | Apr-97 | 40/18866 | 5.21E-05 | 0.00023 | 9.60E-05 | AKT1/EGF/EGFR/NFKBIA | 4 |
| GO:0140353 | lipid export from cell | Apr-97 | 40/18866 | 5.21E-05 | 0.00023 | 9.60E-05 | CYP19A1/IL1B/NOS2/SPP1 | 4 |
| GO:0043123 | positive regulation of I-kappaB kinase/NF-kappaB signaling | Jul-97 | 188/18866 | 5.26E-05 | 0.00023 | 9.69E-05 | ADIPOQ/AKT1/CTNNB1/HMOX1/IKBKB/IL1B/TNF | 7 |
| GO:0002576 | platelet degranulation | Jun-97 | 129/18866 | 5.40E-05 | 0.00023 | 9.94E-05 | ALB/EGF/SERPINE1/SOD1/TGFB1/VEGFA | 6 |
| GO:0003007 | heart morphogenesis | Aug-97 | 258/18866 | 5.55E-05 | 0.00024 | 0.0001 | CTNNB1/JUN/NOS3/PIM1/RXRA/TGFB1/TP53/VEGFA | 8 |
| GO:0010951 | negative regulation of endopeptidase activity | Aug-97 | 258/18866 | 5.55E-05 | 0.00024 | 0.0001 | AKT1/BIRC5/MMP9/PTGS2/SERPINE1/SLPI/TNF/VEGFA | 8 |
| GO:0051048 | negative regulation of secretion | Jul-97 | 190/18866 | 5.63E-05 | 0.00024 | 0.0001 | ADIPOQ/EGF/ERBB3/HMOX1/IL1B/PTGER3/SREBF1 | 7 |
| GO:0009410 | response to xenobiotic stimulus | Jun-97 | 130/18866 | 5.64E-05 | 0.00024 | 0.0001 | AHR/CYP1A1/CYP1A2/CYP3A4/GSTM1/GSTP1 | 6 |
| GO:0034349 | glial cell apoptotic process | Mar-97 | 15/18866 | 5.73E-05 | 0.00024 | 0.0001 | CASP3/CCL2/TP53 | 3 |
| GO:0035635 | entry of bacterium into host cell | Mar-97 | 15/18866 | 5.73E-05 | 0.00024 | 0.0001 | CAV1/CTNNB1/CXCL8 | 3 |
| GO:0045346 | regulation of MHC class II biosynthetic process | Mar-97 | 15/18866 | 5.73E-05 | 0.00024 | 0.0001 | IFNG/IL10/IL4 | 3 |
| GO:0051044 | positive regulation of membrane protein ectodomain proteolysis | Mar-97 | 15/18866 | 5.73E-05 | 0.00024 | 0.0001 | IFNG/IL1B/TNF | 3 |
| GO:0032945 | negative regulation of mononuclear cell proliferation | May-97 | 80/18866 | 5.74E-05 | 0.00024 | 0.0001 | CASP3/CRP/ERBB2/IL10/IL2 | 5 |
| GO:0048678 | response to axon injury | May-97 | 80/18866 | 5.74E-05 | 0.00024 | 0.0001 | BCL2/JUN/PTEN/SOD1/SPP1 | 5 |
| GO:0030890 | positive regulation of B cell proliferation | Apr-97 | 41/18866 | 5.75E-05 | 0.00024 | 0.0001 | BCL2/CDKN1A/IL2/IL4 | 4 |
| GO:0038083 | peptidyl-tyrosine autophosphorylation | Apr-97 | 41/18866 | 5.75E-05 | 0.00024 | 0.0001 | CAV1/EGFR/MAPK3/VEGFA | 4 |
| GO:2000008 | regulation of protein localization to cell surface | Apr-97 | 41/18866 | 5.75E-05 | 0.00024 | 0.0001 | AKT1/CTNNB1/EGF/TNF | 4 |
| GO:0010821 | regulation of mitochondrion organization | Jul-97 | 191/18866 | 5.82E-05 | 0.00025 | 0.00011 | AKT1/BCL2/BCL2L1/MAPK8/MMP9/SREBF1/TP53 | 7 |
| GO:0006766 | vitamin metabolic process | Jun-97 | 131/18866 | 5.89E-05 | 0.00025 | 0.00011 | CYP1A1/CYP3A4/IFNG/IL1B/PPARD/TNF | 6 |
| GO:0019218 | regulation of steroid metabolic process | Jun-97 | 131/18866 | 5.89E-05 | 0.00025 | 0.00011 | APOB/IFNG/IL1B/SOD1/SREBF1/TNF | 6 |
| GO:0001960 | negative regulation of cytokine-mediated signaling pathway | May-97 | 81/18866 | 6.09E-05 | 0.00026 | 0.00011 | ADIPOQ/CAV1/GSTP1/IL6/PPARG | 5 |
| GO:0050829 | defense response to Gram-negative bacterium | May-97 | 81/18866 | 6.09E-05 | 0.00026 | 0.00011 | F2/IL6/LYZ/NOS2/SERPINE1 | 5 |
| GO:0006509 | membrane protein ectodomain proteolysis | Apr-97 | 42/18866 | 6.33E-05 | 0.00027 | 0.00011 | IFNG/IL10/IL1B/TNF | 4 |
| GO:0048246 | macrophage chemotaxis | Apr-97 | 42/18866 | 6.33E-05 | 0.00027 | 0.00011 | CCL2/CYP19A1/MAPK1/MAPK3 | 4 |
| GO:1900371 | regulation of purine nucleotide biosynthetic process | Apr-97 | 42/18866 | 6.33E-05 | 0.00027 | 0.00011 | IL4/NOS2/NOS3/STAT3 | 4 |
| GO:1904591 | positive regulation of protein import | Apr-97 | 42/18866 | 6.33E-05 | 0.00027 | 0.00011 | IFNG/MAPK1/PTGS2/TGFB1 | 4 |
| GO:0019058 | viral life cycle | Sep-97 | 341/18866 | 6.55E-05 | 0.00028 | 0.00012 | BCL2/CAV1/CCL2/CXCL8/DPP4/EGFR/ICAM1/SLPI/TNF | 9 |
| GO:0007219 | Notch signaling pathway | Jul-97 | 195/18866 | 6.63E-05 | 0.00028 | 0.00012 | AKT1/EGF/EGFR/MYC/NFKBIA/NOS3/STAT3 | 7 |
| GO:1902275 | regulation of chromatin organization | Jul-97 | 195/18866 | 6.63E-05 | 0.00028 | 0.00012 | CTNNB1/IL1B/MAPK3/MAPK8/SREBF1/TP53/VEGFA | 7 |
| GO:0006816 | calcium ion transport | Oct-97 | 426/18866 | 6.77E-05 | 0.00028 | 0.00012 | BCL2/CAV1/CCL2/CTNNB1/EGF/F2/ICAM1/NOS3/PIK3CG/PTGS2 | 10 |
| GO:0001570 | vasculogenesis | May-97 | 83/18866 | 6.85E-05 | 0.00029 | 0.00012 | CAV1/CTNNB1/TGFB1/VEGFA/XDH | 5 |
| GO:0008631 | intrinsic apoptotic signaling pathway in response to oxidative stress | Apr-97 | 43/18866 | 6.95E-05 | 0.00029 | 0.00012 | AKT1/BCL2/HSPB1/SOD1 | 4 |
| GO:0030808 | regulation of nucleotide biosynthetic process | Apr-97 | 43/18866 | 6.95E-05 | 0.00029 | 0.00012 | IL4/NOS2/NOS3/STAT3 | 4 |
| GO:0071364 | cellular response to epidermal growth factor stimulus | Apr-97 | 43/18866 | 6.95E-05 | 0.00029 | 0.00012 | AKT1/EGFR/ERBB2/GSTP1 | 4 |
| GO:0019372 | lipoxygenase pathway | Mar-97 | 16/18866 | 7.03E-05 | 0.00029 | 0.00012 | ALOX5/PON1/PTGS2 | 3 |
| GO:0034116 | positive regulation of heterotypic cell-cell adhesion | Mar-97 | 16/18866 | 7.03E-05 | 0.00029 | 0.00012 | IL10/IL1B/TNF | 3 |
| GO:0045342 | MHC class II biosynthetic process | Mar-97 | 16/18866 | 7.03E-05 | 0.00029 | 0.00012 | IFNG/IL10/IL4 | 3 |
| GO:0045727 | positive regulation of translation | Jun-97 | 136/18866 | 7.25E-05 | 0.0003 | 0.00013 | ERBB2/IL6/MAPK1/MAPK3/RXRA/TNF | 6 |
| GO:0032620 | interleukin-17 production | Apr-97 | 44/18866 | 7.61E-05 | 0.00032 | 0.00013 | IFNG/IL2/IL6/TGFB1 | 4 |
| GO:0006970 | response to osmotic stress | May-97 | 85/18866 | 7.67E-05 | 0.00032 | 0.00014 | CASP3/EGFR/PTGS2/TNF/TP53 | 5 |
| GO:0110110 | positive regulation of animal organ morphogenesis | May-97 | 85/18866 | 7.67E-05 | 0.00032 | 0.00014 | AR/CTNNB1/MYC/PIM1/VEGFA | 5 |
| GO:0010466 | negative regulation of peptidase activity | Aug-97 | 272/18866 | 8.04E-05 | 0.00033 | 0.00014 | AKT1/BIRC5/MMP9/PTGS2/SERPINE1/SLPI/TNF/VEGFA | 8 |
| GO:0001776 | leukocyte homeostasis | May-97 | 86/18866 | 8.11E-05 | 0.00033 | 0.00014 | AKT1/BCL2/CASP3/IL2/IL6 | 5 |
| GO:0007272 | ensheathment of neurons | Jun-97 | 139/18866 | 8.18E-05 | 0.00034 | 0.00014 | AKT1/CTNNB1/ERBB2/PPARD/PTEN/SOD1 | 6 |
| GO:0008366 | axon ensheathment | Jun-97 | 139/18866 | 8.18E-05 | 0.00034 | 0.00014 | AKT1/CTNNB1/ERBB2/PPARD/PTEN/SOD1 | 6 |
| GO:0009746 | response to hexose | Jul-97 | 202/18866 | 8.27E-05 | 0.00034 | 0.00014 | ADIPOQ/CASP3/ICAM1/PPARD/PTEN/PTGS2/SREBF1 | 7 |
| GO:0034341 | response to interferon-gamma | Jul-97 | 202/18866 | 8.27E-05 | 0.00034 | 0.00014 | CCL2/ICAM1/IFNG/NOS2/PPARG/TP53/VCAM1 | 7 |
| GO:0002639 | positive regulation of immunoglobulin production | Apr-97 | 45/18866 | 8.32E-05 | 0.00034 | 0.00015 | IL2/IL4/IL6/TGFB1 | 4 |
| GO:0032722 | positive regulation of chemokine production | Apr-97 | 45/18866 | 8.32E-05 | 0.00034 | 0.00015 | ADIPOQ/IL1B/IL6/TNF | 4 |
| GO:0034198 | cellular response to amino acid starvation | Apr-97 | 45/18866 | 8.32E-05 | 0.00034 | 0.00015 | CDKN1A/MAPK1/MAPK3/MAPK8 | 4 |
| GO:0060443 | mammary gland morphogenesis | Apr-97 | 45/18866 | 8.32E-05 | 0.00034 | 0.00015 | AR/CAV1/ESR1/PGR | 4 |
| GO:0010715 | regulation of extracellular matrix disassembly | Mar-97 | 17/18866 | 8.50E-05 | 0.00035 | 0.00015 | DPP4/IL6/TGFB1 | 3 |
| GO:0031065 | positive regulation of histone deacetylation | Mar-97 | 17/18866 | 8.50E-05 | 0.00035 | 0.00015 | SREBF1/TP53/VEGFA | 3 |
| GO:0032966 | negative regulation of collagen biosynthetic process | Mar-97 | 17/18866 | 8.50E-05 | 0.00035 | 0.00015 | IL6/PPARD/PPARG | 3 |
| GO:0090185 | negative regulation of kidney development | Mar-97 | 17/18866 | 8.50E-05 | 0.00035 | 0.00015 | ADIPOQ/CTNNB1/MMP9 | 3 |
| GO:1905331 | negative regulation of morphogenesis of an epithelium | Mar-97 | 17/18866 | 8.50E-05 | 0.00035 | 0.00015 | CTNNB1/PTEN/TNF | 3 |
| GO:0051781 | positive regulation of cell division | May-97 | 87/18866 | 8.57E-05 | 0.00035 | 0.00015 | CAT/IL1A/IL1B/TGFB1/VEGFA | 5 |
| GO:0060761 | negative regulation of response to cytokine stimulus | May-97 | 87/18866 | 8.57E-05 | 0.00035 | 0.00015 | ADIPOQ/CAV1/GSTP1/IL6/PPARG | 5 |
| GO:0001889 | liver development | Jun-97 | 141/18866 | 8.86E-05 | 0.00036 | 0.00015 | CYP1A1/EGFR/HMOX1/IL10/IL6/JUN | 6 |
| GO:0090559 | regulation of membrane permeability | May-97 | 88/18866 | 9.05E-05 | 0.00037 | 0.00016 | BCL2/BCL2L1/MAPK8/STAT3/TP53 | 5 |
| GO:0032570 | response to progesterone | Apr-97 | 46/18866 | 9.08E-05 | 0.00037 | 0.00016 | CAV1/FOS/SREBF1/TGFB1 | 4 |
| GO:0043330 | response to exogenous dsRNA | Apr-97 | 46/18866 | 9.08E-05 | 0.00037 | 0.00016 | CAV1/MAPK1/MAPK3/NFKBIA | 4 |
| GO:2000142 | regulation of DNA-templated transcription, initiation | Apr-97 | 46/18866 | 9.08E-05 | 0.00037 | 0.00016 | CTNNB1/ESR1/JUN/TP53 | 4 |
| GO:0033044 | regulation of chromosome organization | Sep-97 | 356/18866 | 9.10E-05 | 0.00037 | 0.00016 | CTNNB1/IL1B/MAPK1/MAPK3/MAPK8/MYC/SREBF1/TP53/VEGFA | 9 |
| GO:0072073 | kidney epithelium development | Jun-97 | 142/18866 | 9.21E-05 | 0.00037 | 0.00016 | ADIPOQ/BCL2/CAT/CTNNB1/MYC/VEGFA | 6 |
| GO:0015980 | energy derivation by oxidation of organic compounds | Aug-97 | 278/18866 | 9.35E-05 | 0.00038 | 0.00016 | AKT1/CAT/CYP1A2/IFNG/IL4/MYC/NOS2/TP53 | 8 |
| GO:0045165 | cell fate commitment | Aug-97 | 278/18866 | 9.35E-05 | 0.00038 | 0.00016 | AR/BCL2/CASP3/CTNNB1/IL6/PPARG/STAT3/TP53 | 8 |
| GO:0022602 | ovulation cycle process | Apr-97 | 47/18866 | 9.89E-05 | 0.0004 | 0.00017 | CASP3/ESR1/NOS3/PGR | 4 |
| GO:0030574 | collagen catabolic process | Apr-97 | 47/18866 | 9.89E-05 | 0.0004 | 0.00017 | MMP1/MMP2/MMP3/MMP9 | 4 |
| GO:0045776 | negative regulation of blood pressure | Apr-97 | 47/18866 | 9.89E-05 | 0.0004 | 0.00017 | ADIPOQ/NOS2/NOS3/PPARA | 4 |
| GO:0046677 | response to antibiotic | Apr-97 | 47/18866 | 9.89E-05 | 0.0004 | 0.00017 | CASP3/CYP1A1/SOD1/TP53 | 4 |
| GO:0048806 | genitalia development | Apr-97 | 47/18866 | 9.89E-05 | 0.0004 | 0.00017 | AR/CTNNB1/CYP19A1/ESR1 | 4 |
| GO:0061008 | hepaticobiliary system development | Jun-97 | 144/18866 | 9.95E-05 | 0.0004 | 0.00017 | CYP1A1/EGFR/HMOX1/IL10/IL6/JUN | 6 |
| GO:0010713 | negative regulation of collagen metabolic process | Mar-97 | 18/18866 | 0.0001 | 0.0004 | 0.00017 | IL6/PPARD/PPARG | 3 |
| GO:0032740 | positive regulation of interleukin-17 production | Mar-97 | 18/18866 | 0.0001 | 0.0004 | 0.00017 | IL2/IL6/TGFB1 | 3 |
| GO:0035994 | response to muscle stretch | Mar-97 | 18/18866 | 0.0001 | 0.0004 | 0.00017 | FOS/JUN/NFKBIA | 3 |
| GO:0043217 | myelin maintenance | Mar-97 | 18/18866 | 0.0001 | 0.0004 | 0.00017 | AKT1/PTEN/SOD1 | 3 |
| GO:0071605 | monocyte chemotactic protein-1 production | Mar-97 | 18/18866 | 0.0001 | 0.0004 | 0.00017 | ADIPOQ/GSTP1/IL1B | 3 |
| GO:0071637 | regulation of monocyte chemotactic protein-1 production | Mar-97 | 18/18866 | 0.0001 | 0.0004 | 0.00017 | ADIPOQ/GSTP1/IL1B | 3 |
| GO:0150078 | positive regulation of neuroinflammatory response | Mar-97 | 18/18866 | 0.0001 | 0.0004 | 0.00017 | IL1B/IL6/TNF | 3 |
| GO:1900221 | regulation of amyloid-beta clearance | Mar-97 | 18/18866 | 0.0001 | 0.0004 | 0.00017 | IFNG/IL4/TNF | 3 |
| GO:0016054 | organic acid catabolic process | Aug-97 | 282/18866 | 0.0001 | 0.00041 | 0.00017 | ADIPOQ/AKT1/NOS2/NOS3/PON1/PPARA/PPARD/TGFB1 | 8 |
| GO:0046395 | carboxylic acid catabolic process | Aug-97 | 282/18866 | 0.0001 | 0.00041 | 0.00017 | ADIPOQ/AKT1/NOS2/NOS3/PON1/PPARA/PPARD/TGFB1 | 8 |
| GO:0002377 | immunoglobulin production | Jul-97 | 210/18866 | 0.00011 | 0.00042 | 0.00018 | CD40LG/IL10/IL2/IL4/IL6/TGFB1/TNF | 7 |
| GO:0030100 | regulation of endocytosis | Jul-97 | 210/18866 | 0.00011 | 0.00042 | 0.00018 | CAV1/EGF/IL4/PPARG/SELE/SERPINE1/VEGFA | 7 |
| GO:0045216 | cell-cell junction organization | Jul-97 | 210/18866 | 0.00011 | 0.00042 | 0.00018 | CAV1/CTNNB1/IKBKB/IL1B/TGFB1/TNF/VEGFA | 7 |
| GO:0001822 | kidney development | Aug-97 | 283/18866 | 0.00011 | 0.00042 | 0.00018 | ADIPOQ/BCL2/CAT/CTNNB1/MMP9/MYC/ODC1/VEGFA | 8 |
| GO:0046849 | bone remodeling | May-97 | 91/18866 | 0.00011 | 0.00042 | 0.00018 | CTNNB1/EGFR/IL6/SPP1/TGFB1 | 5 |
| GO:0060333 | interferon-gamma-mediated signaling pathway | May-97 | 91/18866 | 0.00011 | 0.00042 | 0.00018 | ICAM1/IFNG/PPARG/TP53/VCAM1 | 5 |
| GO:0097306 | cellular response to alcohol | May-97 | 91/18866 | 0.00011 | 0.00042 | 0.00018 | AHR/AKT1/CTNNB1/PPARG/PTEN | 5 |
| GO:0046631 | alpha-beta T cell activation | Jun-97 | 146/18866 | 0.00011 | 0.00042 | 0.00018 | BCL2/IFNG/IL2/IL4/IL6/STAT3 | 6 |
| GO:0031952 | regulation of protein autophosphorylation | Apr-97 | 48/18866 | 0.00011 | 0.00042 | 0.00018 | ADIPOQ/CAV1/JUN/VEGFA | 4 |
| GO:1904036 | negative regulation of epithelial cell apoptotic process | Apr-97 | 48/18866 | 0.00011 | 0.00042 | 0.00018 | HMOX1/ICAM1/IL4/SERPINE1 | 4 |
| GO:1990928 | response to amino acid starvation | Apr-97 | 48/18866 | 0.00011 | 0.00042 | 0.00018 | CDKN1A/MAPK1/MAPK3/MAPK8 | 4 |
| GO:0044409 | entry into host | Jun-97 | 147/18866 | 0.00011 | 0.00043 | 0.00019 | CAV1/CTNNB1/CXCL8/DPP4/EGFR/ICAM1 | 6 |
| GO:0045582 | positive regulation of T cell differentiation | May-97 | 92/18866 | 0.00011 | 0.00044 | 0.00019 | IFNG/IL1A/IL1B/IL2/IL4 | 5 |
| GO:0007254 | JNK cascade | Jul-97 | 213/18866 | 0.00012 | 0.00045 | 0.00019 | AKT1/CD40LG/EGFR/GSTP1/IL1B/MAPK8/TNF | 7 |
| GO:0010675 | regulation of cellular carbohydrate metabolic process | Jun-97 | 148/18866 | 0.00012 | 0.00045 | 0.00019 | ADIPOQ/AKT1/IGFBP3/PPARA/STAT3/TP53 | 6 |
| GO:0006692 | prostanoid metabolic process | Apr-97 | 49/18866 | 0.00012 | 0.00045 | 0.00019 | GSTM1/GSTP1/IL1B/PTGS2 | 4 |
| GO:0006693 | prostaglandin metabolic process | Apr-97 | 49/18866 | 0.00012 | 0.00045 | 0.00019 | GSTM1/GSTP1/IL1B/PTGS2 | 4 |
| GO:0031279 | regulation of cyclase activity | Apr-97 | 49/18866 | 0.00012 | 0.00045 | 0.00019 | MAPK3/MAPK8/NOS2/NOS3 | 4 |
| GO:0090199 | regulation of release of cytochrome c from mitochondria | Apr-97 | 49/18866 | 0.00012 | 0.00045 | 0.00019 | AKT1/BCL2L1/MMP9/TP53 | 4 |
| GO:0021700 | developmental maturation | Aug-97 | 287/18866 | 0.00012 | 0.00045 | 0.00019 | BCL2/CDKN1A/CTNNB1/MMP2/PGR/PPARG/PTEN/VEGFA | 8 |
| GO:0006006 | glucose metabolic process | Jul-97 | 214/18866 | 0.00012 | 0.00046 | 0.0002 | ADIPOQ/AKT1/IGFBP3/PPARA/PPARD/TNF/TP53 | 7 |
| GO:0010544 | negative regulation of platelet activation | Mar-97 | 19/18866 | 0.00012 | 0.00046 | 0.0002 | F2/NOS3/THBD | 3 |
| GO:0051546 | keratinocyte migration | Mar-97 | 19/18866 | 0.00012 | 0.00046 | 0.0002 | MMP9/PPARD/PTEN | 3 |
| GO:0060252 | positive regulation of glial cell proliferation | Mar-97 | 19/18866 | 0.00012 | 0.00046 | 0.0002 | IL1B/IL6/TNF | 3 |
| GO:0060438 | trachea development | Mar-97 | 19/18866 | 0.00012 | 0.00046 | 0.0002 | CTNNB1/MAPK1/MAPK3 | 3 |
| GO:2000696 | regulation of epithelial cell differentiation involved in kidney development | Mar-97 | 19/18866 | 0.00012 | 0.00046 | 0.0002 | ADIPOQ/CTNNB1/MMP9 | 3 |
| GO:0010660 | regulation of muscle cell apoptotic process | May-97 | 94/18866 | 0.00012 | 0.00048 | 0.0002 | HMOX1/IFNG/PPARG/PTEN/TP53 | 5 |
| GO:0010718 | positive regulation of epithelial to mesenchymal transition | Apr-97 | 50/18866 | 0.00013 | 0.00048 | 0.00021 | CTNNB1/IL1B/IL6/TGFB1 | 4 |
| GO:0050871 | positive regulation of B cell activation | Jun-97 | 151/18866 | 0.00013 | 0.0005 | 0.00021 | BCL2/CDKN1A/IL2/IL4/IL6/TGFB1 | 6 |
| GO:0045638 | negative regulation of myeloid cell differentiation | May-97 | 95/18866 | 0.00013 | 0.0005 | 0.00021 | ADIPOQ/CTNNB1/IL4/MYC/NFKBIA | 5 |
| GO:0097194 | execution phase of apoptosis | May-97 | 95/18866 | 0.00013 | 0.0005 | 0.00021 | AKT1/BCL2L1/CASP3/IL6/TP53 | 5 |
| GO:0072001 | renal system development | Aug-97 | 292/18866 | 0.00013 | 0.0005 | 0.00021 | ADIPOQ/BCL2/CAT/CTNNB1/MMP9/MYC/ODC1/VEGFA | 8 |
| GO:0002762 | negative regulation of myeloid leukocyte differentiation | Apr-97 | 51/18866 | 0.00014 | 0.00052 | 0.00022 | ADIPOQ/CTNNB1/IL4/MYC | 4 |
| GO:0006984 | ER-nucleus signaling pathway | Apr-97 | 51/18866 | 0.00014 | 0.00052 | 0.00022 | CCL2/CXCL8/SREBF1/TP53 | 4 |
| GO:0034976 | response to endoplasmic reticulum stress | Aug-97 | 294/18866 | 0.00014 | 0.00052 | 0.00022 | ALOX5/BCL2/BCL2L1/CAV1/CCL2/CXCL8/JUN/TP53 | 8 |
| GO:0051146 | striated muscle cell differentiation | Aug-97 | 295/18866 | 0.00014 | 0.00053 | 0.00023 | AKT1/BCL2/CASP3/IL4/PPARA/RXRA/TGFB1/VEGFA | 8 |
| GO:0002689 | negative regulation of leukocyte chemotaxis | Mar-97 | 20/18866 | 0.00014 | 0.00053 | 0.00023 | CCL2/CYP19A1/DPP4 | 3 |
| GO:0002902 | regulation of B cell apoptotic process | Mar-97 | 20/18866 | 0.00014 | 0.00053 | 0.00023 | IL10/IL2/PTEN | 3 |
| GO:0007252 | I-kappaB phosphorylation | Mar-97 | 20/18866 | 0.00014 | 0.00053 | 0.00023 | AKT1/IKBKB/TNF | 3 |
| GO:0051412 | response to corticosterone | Mar-97 | 20/18866 | 0.00014 | 0.00053 | 0.00023 | CDKN1A/FOS/HSD3B1 | 3 |
| GO:0051767 | nitric-oxide synthase biosynthetic process | Mar-97 | 20/18866 | 0.00014 | 0.00053 | 0.00023 | CCL2/GSTP1/IFNG | 3 |
| GO:0051769 | regulation of nitric-oxide synthase biosynthetic process | Mar-97 | 20/18866 | 0.00014 | 0.00053 | 0.00023 | CCL2/GSTP1/IFNG | 3 |
| GO:0060353 | regulation of cell adhesion molecule production | Mar-97 | 20/18866 | 0.00014 | 0.00053 | 0.00023 | CAV1/CXCL8/IL1B | 3 |
| GO:1900409 | positive regulation of cellular response to oxidative stress | Mar-97 | 20/18866 | 0.00014 | 0.00053 | 0.00023 | MMP3/SOD1/TNF | 3 |
| GO:1902004 | positive regulation of amyloid-beta formation | Mar-97 | 20/18866 | 0.00014 | 0.00053 | 0.00023 | CASP3/IFNG/TNF | 3 |
| GO:0006476 | protein deacetylation | May-97 | 97/18866 | 0.00014 | 0.00054 | 0.00023 | IFNG/MAPK8/SREBF1/TP53/VEGFA | 5 |
| GO:0035710 | CD4-positive, alpha-beta T cell activation | May-97 | 97/18866 | 0.00014 | 0.00054 | 0.00023 | IFNG/IL2/IL4/IL6/STAT3 | 5 |
| GO:0120162 | positive regulation of cold-induced thermogenesis | May-97 | 97/18866 | 0.00014 | 0.00054 | 0.00023 | ACHE/ADIPOQ/CAV1/IL4/VEGFA | 5 |
| GO:1901184 | regulation of ERBB signaling pathway | May-97 | 97/18866 | 0.00014 | 0.00054 | 0.00023 | AKT1/EGF/EGFR/ERBB2/MMP9 | 5 |
| GO:0002262 | myeloid cell homeostasis | Jun-97 | 154/18866 | 0.00014 | 0.00054 | 0.00023 | CASP3/HMOX1/IL6/SOD1/STAT3/VEGFA | 6 |
| GO:0031647 | regulation of protein stability | Aug-97 | 296/18866 | 0.00014 | 0.00054 | 0.00023 | BCL2/CASP3/CDKN1A/MAPK1/PIM1/PTEN/SREBF1/TP53 | 8 |
| GO:0042445 | hormone metabolic process | Jul-97 | 221/18866 | 0.00014 | 0.00054 | 0.00023 | CYP19A1/CYP1A1/CYP1A2/CYP3A4/ESR1/HSD3B1/SPP1 | 7 |
| GO:0000186 | activation of MAPKK activity | Apr-97 | 52/18866 | 0.00015 | 0.00055 | 0.00023 | EGF/EGFR/MAPK1/MAPK3 | 4 |
| GO:0031103 | axon regeneration | Apr-97 | 52/18866 | 0.00015 | 0.00055 | 0.00023 | BCL2/JUN/PTEN/SPP1 | 4 |
| GO:0060425 | lung morphogenesis | Apr-97 | 52/18866 | 0.00015 | 0.00055 | 0.00023 | CTNNB1/MAPK1/MAPK3/TNF | 4 |
| GO:0001657 | ureteric bud development | May-97 | 98/18866 | 0.00015 | 0.00056 | 0.00024 | BCL2/CAT/CTNNB1/MYC/VEGFA | 5 |
| GO:0010657 | muscle cell apoptotic process | May-97 | 98/18866 | 0.00015 | 0.00056 | 0.00024 | HMOX1/IFNG/PPARG/PTEN/TP53 | 5 |
| GO:0030316 | osteoclast differentiation | May-97 | 98/18866 | 0.00015 | 0.00056 | 0.00024 | CTNNB1/FOS/IFNG/IL4/TNF | 5 |
| GO:0045833 | negative regulation of lipid metabolic process | May-97 | 98/18866 | 0.00015 | 0.00056 | 0.00024 | AKT1/IL1B/PIK3CG/SOD1/TNF | 5 |
| GO:0070838 | divalent metal ion transport | Oct-97 | 471/18866 | 0.00015 | 0.00057 | 0.00024 | BCL2/CAV1/CCL2/CTNNB1/EGF/F2/ICAM1/NOS3/PIK3CG/PTGS2 | 10 |
| GO:0010717 | regulation of epithelial to mesenchymal transition | May-97 | 99/18866 | 0.00016 | 0.00058 | 0.00025 | CTNNB1/IL1B/IL6/PTEN/TGFB1 | 5 |
| GO:0043255 | regulation of carbohydrate biosynthetic process | May-97 | 99/18866 | 0.00016 | 0.00058 | 0.00025 | ADIPOQ/AKT1/EGF/PPARA/TGFB1 | 5 |
| GO:0072163 | mesonephric epithelium development | May-97 | 99/18866 | 0.00016 | 0.00058 | 0.00025 | BCL2/CAT/CTNNB1/MYC/VEGFA | 5 |
| GO:0072164 | mesonephric tubule development | May-97 | 99/18866 | 0.00016 | 0.00058 | 0.00025 | BCL2/CAT/CTNNB1/MYC/VEGFA | 5 |
| GO:0043331 | response to dsRNA | Apr-97 | 53/18866 | 0.00016 | 0.00058 | 0.00025 | CAV1/MAPK1/MAPK3/NFKBIA | 4 |
| GO:0045599 | negative regulation of fat cell differentiation | Apr-97 | 53/18866 | 0.00016 | 0.00058 | 0.00025 | ADIPOQ/IL6/TGFB1/TNF | 4 |
| GO:0045661 | regulation of myoblast differentiation | Apr-97 | 53/18866 | 0.00016 | 0.00058 | 0.00025 | IGFBP3/PPARD/TGFB1/TNF | 4 |
| GO:0045732 | positive regulation of protein catabolic process | Jul-97 | 225/18866 | 0.00016 | 0.0006 | 0.00025 | AKT1/CAV1/EGF/IFNG/IL1B/PTEN/TNF | 7 |
| GO:0002363 | alpha-beta T cell lineage commitment | Mar-97 | 21/18866 | 0.00016 | 0.0006 | 0.00026 | BCL2/IL6/STAT3 | 3 |
| GO:0030728 | ovulation | Mar-97 | 21/18866 | 0.00016 | 0.0006 | 0.00026 | NOS3/PGR/PTGS2 | 3 |
| GO:0050890 | cognition | Aug-97 | 302/18866 | 0.00017 | 0.00061 | 0.00026 | CASP3/EGFR/FOS/JUN/MAPK1/PTEN/PTGS2/TNF | 8 |
| GO:0090068 | positive regulation of cell cycle process | Aug-97 | 302/18866 | 0.00017 | 0.00061 | 0.00026 | AKT1/CDKN1A/CYP1A1/EGF/EGFR/IL1A/IL1B/TP53 | 8 |
| GO:0002702 | positive regulation of production of molecular mediator of immune response | May-97 | 100/18866 | 0.00017 | 0.00061 | 0.00026 | IL1B/IL2/IL4/IL6/TGFB1 | 5 |
| GO:0032869 | cellular response to insulin stimulus | Jul-97 | 226/18866 | 0.00017 | 0.00061 | 0.00026 | ADIPOQ/AKT1/GSTP1/IL1B/PPARG/PTEN/SREBF1 | 7 |
| GO:0006749 | glutathione metabolic process | Apr-97 | 54/18866 | 0.00017 | 0.00062 | 0.00027 | GSR/GSTM1/GSTP1/SOD1 | 4 |
| GO:0016064 | immunoglobulin mediated immune response | Jul-97 | 227/18866 | 0.00017 | 0.00062 | 0.00027 | CD40LG/CRP/IL10/IL2/IL4/TGFB1/TNF | 7 |
| GO:0072511 | divalent inorganic cation transport | Oct-97 | 478/18866 | 0.00017 | 0.00064 | 0.00027 | BCL2/CAV1/CCL2/CTNNB1/EGF/F2/ICAM1/NOS3/PIK3CG/PTGS2 | 10 |
| GO:0042692 | muscle cell differentiation | Sep-97 | 390/18866 | 0.00018 | 0.00066 | 0.00028 | AKT1/BCL2/CASP3/CTNNB1/IL4/PPARA/RXRA/TGFB1/VEGFA | 9 |
| GO:0000079 | regulation of cyclin-dependent protein serine/threonine kinase activity | May-97 | 102/18866 | 0.00018 | 0.00066 | 0.00028 | AKT1/CASP3/CDKN1A/EGFR/PTEN | 5 |
| GO:0051353 | positive regulation of oxidoreductase activity | Apr-97 | 55/18866 | 0.00018 | 0.00067 | 0.00028 | AKT1/IFNG/IL1B/TNF | 4 |
| GO:0019724 | B cell mediated immunity | Jul-97 | 230/18866 | 0.00019 | 0.00067 | 0.00029 | CD40LG/CRP/IL10/IL2/IL4/TGFB1/TNF | 7 |
| GO:0043369 | CD4-positive or CD8-positive, alpha-beta T cell lineage commitment | Mar-97 | 22/18866 | 0.00019 | 0.00069 | 0.00029 | BCL2/IL6/STAT3 | 3 |
| GO:0060352 | cell adhesion molecule production | Mar-97 | 22/18866 | 0.00019 | 0.00069 | 0.00029 | CAV1/CXCL8/IL1B | 3 |
| GO:0071379 | cellular response to prostaglandin stimulus | Mar-97 | 22/18866 | 0.00019 | 0.00069 | 0.00029 | AKT1/APOB/PPARG | 3 |
| GO:1902884 | positive regulation of response to oxidative stress | Mar-97 | 22/18866 | 0.00019 | 0.00069 | 0.00029 | MMP3/SOD1/TNF | 3 |
| GO:0043903 | regulation of symbiotic process | Jul-97 | 231/18866 | 0.00019 | 0.00069 | 0.00029 | BCL2/CAV1/CRP/CXCL8/JUN/SLPI/TNF | 7 |
| GO:0001823 | mesonephros development | May-97 | 103/18866 | 0.00019 | 0.00069 | 0.00029 | BCL2/CAT/CTNNB1/MYC/VEGFA | 5 |
| GO:0030593 | neutrophil chemotaxis | May-97 | 103/18866 | 0.00019 | 0.00069 | 0.00029 | CCL2/CXCL8/DPP4/IL1B/PIK3CG | 5 |
| GO:0048709 | oligodendrocyte differentiation | May-97 | 103/18866 | 0.00019 | 0.00069 | 0.00029 | CTNNB1/ERBB2/GSTP1/PPARG/PTEN | 5 |
| GO:0031214 | biomineral tissue development | Jun-97 | 163/18866 | 0.0002 | 0.00071 | 0.0003 | ALOX5/NOS3/PPARA/PTGS2/SPP1/TGFB1 | 6 |
| GO:0110148 | biomineralization | Jun-97 | 163/18866 | 0.0002 | 0.00071 | 0.0003 | ALOX5/NOS3/PPARA/PTGS2/SPP1/TGFB1 | 6 |
| GO:0031295 | T cell costimulation | Apr-97 | 56/18866 | 0.0002 | 0.00071 | 0.0003 | AKT1/CAV1/CD40LG/DPP4 | 4 |
| GO:0070228 | regulation of lymphocyte apoptotic process | Apr-97 | 56/18866 | 0.0002 | 0.00071 | 0.0003 | IL10/IL2/PTEN/TP53 | 4 |
| GO:0046661 | male sex differentiation | Jun-97 | 164/18866 | 0.0002 | 0.00073 | 0.00031 | AR/BCL2/BCL2L1/CTNNB1/ESR1/ICAM1 | 6 |
| GO:0045787 | positive regulation of cell cycle | Sep-97 | 396/18866 | 0.0002 | 0.00073 | 0.00031 | AKT1/CDKN1A/CYP1A1/EGF/EGFR/IL10/IL1A/IL1B/TP53 | 9 |
| GO:0045621 | positive regulation of lymphocyte differentiation | May-97 | 105/18866 | 0.00021 | 0.00075 | 0.00032 | IFNG/IL1A/IL1B/IL2/IL4 | 5 |
| GO:0048259 | regulation of receptor-mediated endocytosis | May-97 | 105/18866 | 0.00021 | 0.00075 | 0.00032 | EGF/IL4/SELE/SERPINE1/VEGFA | 5 |
| GO:0097006 | regulation of plasma lipoprotein particle levels | May-97 | 105/18866 | 0.00021 | 0.00075 | 0.00032 | ADIPOQ/ALB/APOB/HMOX1/MPO | 5 |
| GO:0019748 | secondary metabolic process | Apr-97 | 57/18866 | 0.00021 | 0.00075 | 0.00032 | BCL2/CYP1A1/CYP1A2/TYR | 4 |
| GO:0071398 | cellular response to fatty acid | Apr-97 | 57/18866 | 0.00021 | 0.00075 | 0.00032 | AKT1/APOB/PPARG/SREBF1 | 4 |
| GO:1905517 | macrophage migration | Apr-97 | 57/18866 | 0.00021 | 0.00075 | 0.00032 | CCL2/CYP19A1/MAPK1/MAPK3 | 4 |
| GO:0071695 | anatomical structure maturation | Jul-97 | 235/18866 | 0.00021 | 0.00075 | 0.00032 | BCL2/CDKN1A/CTNNB1/MMP2/PGR/PPARG/VEGFA | 7 |
| GO:0035743 | CD4-positive, alpha-beta T cell cytokine production | Mar-97 | 23/18866 | 0.00022 | 0.00077 | 0.00033 | IL1B/IL4/IL6 | 3 |
| GO:0072215 | regulation of metanephros development | Mar-97 | 23/18866 | 0.00022 | 0.00077 | 0.00033 | ADIPOQ/CTNNB1/MYC | 3 |
| GO:1900017 | positive regulation of cytokine production involved in inflammatory response | Mar-97 | 23/18866 | 0.00022 | 0.00077 | 0.00033 | IL6/STAT3/TNF | 3 |
| GO:0019233 | sensory perception of pain | May-97 | 106/18866 | 0.00022 | 0.00077 | 0.00033 | CCL2/IL10/MAPK1/MAPK3/PTGS2 | 5 |
| GO:0019395 | fatty acid oxidation | May-97 | 106/18866 | 0.00022 | 0.00077 | 0.00033 | ADIPOQ/AKT1/PPARA/PPARD/PPARG | 5 |
| GO:1904029 | regulation of cyclin-dependent protein kinase activity | May-97 | 106/18866 | 0.00022 | 0.00077 | 0.00033 | AKT1/CASP3/CDKN1A/EGFR/PTEN | 5 |
| GO:1900408 | negative regulation of cellular response to oxidative stress | Apr-97 | 58/18866 | 0.00023 | 0.0008 | 0.00034 | AKT1/CTNNB1/HSPB1/IL10 | 4 |
| GO:1903202 | negative regulation of oxidative stress-induced cell death | Apr-97 | 58/18866 | 0.00023 | 0.0008 | 0.00034 | AKT1/CTNNB1/HSPB1/IL10 | 4 |
| GO:0035601 | protein deacylation | May-97 | 107/18866 | 0.00023 | 0.0008 | 0.00034 | IFNG/MAPK8/SREBF1/TP53/VEGFA | 5 |
| GO:0055017 | cardiac muscle tissue growth | May-97 | 107/18866 | 0.00023 | 0.0008 | 0.00034 | MAPK1/PIM1/PPARA/PTEN/RXRA | 5 |
| GO:0045931 | positive regulation of mitotic cell cycle | Jun-97 | 168/18866 | 0.00023 | 0.00081 | 0.00035 | AKT1/CYP1A1/EGF/EGFR/IL1A/IL1B | 6 |
| GO:0038095 | Fc-epsilon receptor signaling pathway | Jun-97 | 169/18866 | 0.00024 | 0.00084 | 0.00036 | FOS/IKBKB/JUN/MAPK1/MAPK3/MAPK8 | 6 |
| GO:0052126 | movement in host environment | Jun-97 | 169/18866 | 0.00024 | 0.00084 | 0.00036 | CAV1/CTNNB1/CXCL8/DPP4/EGFR/ICAM1 | 6 |
| GO:0001658 | branching involved in ureteric bud morphogenesis | Apr-97 | 59/18866 | 0.00024 | 0.00084 | 0.00036 | BCL2/CTNNB1/MYC/VEGFA | 4 |
| GO:0031102 | neuron projection regeneration | Apr-97 | 59/18866 | 0.00024 | 0.00084 | 0.00036 | BCL2/JUN/PTEN/SPP1 | 4 |
| GO:0042306 | regulation of protein import into nucleus | Apr-97 | 59/18866 | 0.00024 | 0.00084 | 0.00036 | IFNG/MAPK1/PTGS2/TGFB1 | 4 |
| GO:0045454 | cell redox homeostasis | Apr-97 | 59/18866 | 0.00024 | 0.00084 | 0.00036 | GSR/MPO/NOS2/NOS3 | 4 |
| GO:0050707 | regulation of cytokine secretion | Apr-97 | 59/18866 | 0.00024 | 0.00084 | 0.00036 | IFNG/IL10/IL1A/TNF | 4 |
| GO:0071385 | cellular response to glucocorticoid stimulus | Apr-97 | 59/18866 | 0.00024 | 0.00084 | 0.00036 | EGFR/GSTP1/ICAM1/NR3C1 | 4 |
| GO:0010640 | regulation of platelet-derived growth factor receptor signaling pathway | Mar-97 | 24/18866 | 0.00025 | 0.00086 | 0.00037 | ADIPOQ/F3/F7 | 3 |
| GO:0044346 | fibroblast apoptotic process | Mar-97 | 24/18866 | 0.00025 | 0.00086 | 0.00037 | MYC/PIK3CG/TP53 | 3 |
| GO:0060444 | branching involved in mammary gland duct morphogenesis | Mar-97 | 24/18866 | 0.00025 | 0.00086 | 0.00037 | AR/ESR1/PGR | 3 |
| GO:1902993 | positive regulation of amyloid precursor protein catabolic process | Mar-97 | 24/18866 | 0.00025 | 0.00086 | 0.00037 | CASP3/IFNG/TNF | 3 |
| GO:0032653 | regulation of interleukin-10 production | Apr-97 | 60/18866 | 0.00026 | 0.00089 | 0.00038 | CD40LG/IL4/IL6/STAT3 | 4 |
| GO:0042093 | T-helper cell differentiation | Apr-97 | 60/18866 | 0.00026 | 0.00089 | 0.00038 | IL2/IL4/IL6/STAT3 | 4 |
| GO:1902883 | negative regulation of response to oxidative stress | Apr-97 | 60/18866 | 0.00026 | 0.00089 | 0.00038 | AKT1/CTNNB1/HSPB1/IL10 | 4 |
| GO:0006641 | triglyceride metabolic process | May-97 | 110/18866 | 0.00026 | 0.0009 | 0.00038 | APOB/CAT/CAV1/PIK3CG/SREBF1 | 5 |
| GO:0072009 | nephron epithelium development | May-97 | 110/18866 | 0.00026 | 0.0009 | 0.00038 | ADIPOQ/BCL2/CTNNB1/MYC/VEGFA | 5 |
| GO:1904659 | glucose transmembrane transport | May-97 | 110/18866 | 0.00026 | 0.0009 | 0.00038 | ADIPOQ/AKT1/IL1B/PPARD/TNF | 5 |
| GO:0001906 | cell killing | Jun-97 | 172/18866 | 0.00026 | 0.00091 | 0.00039 | F2/ICAM1/IFNG/IL4/LYZ/NOS2 | 6 |
| GO:0050806 | positive regulation of synaptic transmission | Jun-97 | 172/18866 | 0.00026 | 0.00091 | 0.00039 | CCL2/EGFR/MAPK1/PTEN/PTGS2/TNF | 6 |
| GO:0042303 | molting cycle | May-97 | 111/18866 | 0.00027 | 0.00093 | 0.0004 | BCL2/CTNNB1/EGFR/PTGS2/TNF | 5 |
| GO:0042633 | hair cycle | May-97 | 111/18866 | 0.00027 | 0.00093 | 0.0004 | BCL2/CTNNB1/EGFR/PTGS2/TNF | 5 |
| GO:0098732 | macromolecule deacylation | May-97 | 111/18866 | 0.00027 | 0.00093 | 0.0004 | IFNG/MAPK8/SREBF1/TP53/VEGFA | 5 |
| GO:0042593 | glucose homeostasis | Jul-97 | 245/18866 | 0.00027 | 0.00094 | 0.0004 | ADIPOQ/AKT1/ALOX5/ICAM1/IL6/PPARG/STAT3 | 7 |
| GO:1903078 | positive regulation of protein localization to plasma membrane | Apr-97 | 61/18866 | 0.00027 | 0.00094 | 0.0004 | AKT1/EGFR/IFNG/TNF | 4 |
| GO:0010469 | regulation of signaling receptor activity | Jun-97 | 174/18866 | 0.00028 | 0.00096 | 0.00041 | CCL2/EGF/IFNG/IL10/PTEN/SERPINE1 | 6 |
| GO:0033500 | carbohydrate homeostasis | Jul-97 | 246/18866 | 0.00028 | 0.00096 | 0.00041 | ADIPOQ/AKT1/ALOX5/ICAM1/IL6/PPARG/STAT3 | 7 |
| GO:0002053 | positive regulation of mesenchymal cell proliferation | Mar-97 | 25/18866 | 0.00028 | 0.00096 | 0.00041 | CTNNB1/MYC/VEGFA | 3 |
| GO:0002092 | positive regulation of receptor internalization | Mar-97 | 25/18866 | 0.00028 | 0.00096 | 0.00041 | EGF/SELE/VEGFA | 3 |
| GO:0045662 | negative regulation of myoblast differentiation | Mar-97 | 25/18866 | 0.00028 | 0.00096 | 0.00041 | PPARD/TGFB1/TNF | 3 |
| GO:0060571 | morphogenesis of an epithelial fold | Mar-97 | 25/18866 | 0.00028 | 0.00096 | 0.00041 | AR/CTNNB1/EGFR | 3 |
| GO:0071677 | positive regulation of mononuclear cell migration | Mar-97 | 25/18866 | 0.00028 | 0.00096 | 0.00041 | IL4/SERPINE1/TNF | 3 |
| GO:2000679 | positive regulation of transcription regulatory region DNA binding | Mar-97 | 25/18866 | 0.00028 | 0.00096 | 0.00041 | CTNNB1/IFNG/TGFB1 | 3 |
| GO:0002260 | lymphocyte homeostasis | Apr-97 | 62/18866 | 0.00029 | 0.00099 | 0.00042 | AKT1/BCL2/CASP3/IL2 | 4 |
| GO:0010803 | regulation of tumor necrosis factor-mediated signaling pathway | Apr-97 | 62/18866 | 0.00029 | 0.00099 | 0.00042 | ADIPOQ/GSTP1/IKBKB/TNF | 4 |
| GO:0055025 | positive regulation of cardiac muscle tissue development | Apr-97 | 62/18866 | 0.00029 | 0.00099 | 0.00042 | ERBB3/MAPK1/PIM1/TGFB1 | 4 |
| GO:0071384 | cellular response to corticosteroid stimulus | Apr-97 | 62/18866 | 0.00029 | 0.00099 | 0.00042 | EGFR/GSTP1/ICAM1/NR3C1 | 4 |
| GO:1904589 | regulation of protein import | Apr-97 | 62/18866 | 0.00029 | 0.00099 | 0.00042 | IFNG/MAPK1/PTGS2/TGFB1 | 4 |
| GO:0007009 | plasma membrane organization | May-97 | 113/18866 | 0.00029 | 0.001 | 0.00042 | AKT1/AR/CAV1/PTEN/SOD1 | 5 |
| GO:0035821 | modulation of process of other organism | May-97 | 113/18866 | 0.00029 | 0.001 | 0.00042 | BCL2L1/CRP/JUN/RXRA/SLPI | 5 |
| GO:1903510 | mucopolysaccharide metabolic process | May-97 | 113/18866 | 0.00029 | 0.001 | 0.00042 | AKT1/EGF/IL1B/PIM1/TGFB1 | 5 |
| GO:0008645 | hexose transmembrane transport | May-97 | 114/18866 | 0.00031 | 0.00104 | 0.00044 | ADIPOQ/AKT1/IL1B/PPARD/TNF | 5 |
| GO:0032613 | interleukin-10 production | Apr-97 | 63/18866 | 0.00031 | 0.00105 | 0.00045 | CD40LG/IL4/IL6/STAT3 | 4 |
| GO:0043010 | camera-type eye development | Aug-97 | 332/18866 | 0.00031 | 0.00106 | 0.00045 | ACHE/CTNNB1/CYP1A1/EGFR/JUN/RHO/RXRA/VEGFA | 8 |
| GO:0001783 | B cell apoptotic process | Mar-97 | 26/18866 | 0.00031 | 0.00106 | 0.00045 | IL10/IL2/PTEN | 3 |
| GO:0060740 | prostate gland epithelium morphogenesis | Mar-97 | 26/18866 | 0.00031 | 0.00106 | 0.00045 | AR/ESR1/RXRA | 3 |
| GO:1900739 | regulation of protein insertion into mitochondrial membrane involved in apoptotic signaling pathway | Mar-97 | 26/18866 | 0.00031 | 0.00106 | 0.00045 | BCL2/MAPK8/TP53 | 3 |
| GO:1900740 | positive regulation of protein insertion into mitochondrial membrane involved in apoptotic signaling pathway | Mar-97 | 26/18866 | 0.00031 | 0.00106 | 0.00045 | BCL2/MAPK8/TP53 | 3 |
| GO:0060419 | heart growth | May-97 | 115/18866 | 0.00032 | 0.00107 | 0.00046 | MAPK1/PIM1/PPARA/PTEN/RXRA | 5 |
| GO:0048469 | cell maturation | Jun-97 | 179/18866 | 0.00032 | 0.00109 | 0.00047 | BCL2/CDKN1A/CTNNB1/PGR/PPARG/VEGFA | 6 |
| GO:0045453 | bone resorption | Apr-97 | 64/18866 | 0.00033 | 0.00111 | 0.00047 | CTNNB1/EGFR/IL6/SPP1 | 4 |
| GO:0051926 | negative regulation of calcium ion transport | Apr-97 | 64/18866 | 0.00033 | 0.00111 | 0.00047 | BCL2/ICAM1/NOS3/PTGS2 | 4 |
| GO:0015749 | monosaccharide transmembrane transport | May-97 | 116/18866 | 0.00033 | 0.00111 | 0.00047 | ADIPOQ/AKT1/IL1B/PPARD/TNF | 5 |
| GO:0030518 | intracellular steroid hormone receptor signaling pathway | May-97 | 116/18866 | 0.00033 | 0.00111 | 0.00047 | AR/ESR1/NR3C1/NR3C2/PGR | 5 |
| GO:0019318 | hexose metabolic process | Jul-97 | 254/18866 | 0.00034 | 0.00114 | 0.00048 | ADIPOQ/AKT1/IGFBP3/PPARA/PPARD/TNF/TP53 | 7 |
| GO:0010950 | positive regulation of endopeptidase activity | Jun-97 | 181/18866 | 0.00034 | 0.00115 | 0.00049 | F3/MYC/PPARG/STAT3/TNF/XDH | 6 |
| GO:0031640 | killing of cells of other organism | Apr-97 | 65/18866 | 0.00035 | 0.00117 | 0.0005 | F2/IFNG/LYZ/NOS2 | 4 |
| GO:0051205 | protein insertion into membrane | Apr-97 | 65/18866 | 0.00035 | 0.00117 | 0.0005 | BCL2/EGFR/MAPK8/TP53 | 4 |
| GO:0060675 | ureteric bud morphogenesis | Apr-97 | 65/18866 | 0.00035 | 0.00117 | 0.0005 | BCL2/CTNNB1/MYC/VEGFA | 4 |
| GO:0045672 | positive regulation of osteoclast differentiation | Mar-97 | 27/18866 | 0.00035 | 0.00117 | 0.0005 | FOS/IFNG/TNF | 3 |
| GO:1900120 | regulation of receptor binding | Mar-97 | 27/18866 | 0.00035 | 0.00117 | 0.0005 | ADIPOQ/IL10/MMP9 | 3 |
| GO:1902175 | regulation of oxidative stress-induced intrinsic apoptotic signaling pathway | Mar-97 | 27/18866 | 0.00035 | 0.00117 | 0.0005 | AKT1/HSPB1/SOD1 | 3 |
| GO:0034219 | carbohydrate transmembrane transport | May-97 | 118/18866 | 0.00036 | 0.00119 | 0.00051 | ADIPOQ/AKT1/IL1B/PPARD/TNF | 5 |
| GO:0050868 | negative regulation of T cell activation | May-97 | 118/18866 | 0.00036 | 0.00119 | 0.00051 | CASP3/ERBB2/IL10/IL2/IL4 | 5 |
| GO:0043433 | negative regulation of DNA-binding transcription factor activity | Jun-97 | 183/18866 | 0.00037 | 0.00121 | 0.00052 | CAT/ESR1/HMOX1/IL10/NFKBIA/PIM1 | 6 |
| GO:0034394 | protein localization to cell surface | Apr-97 | 66/18866 | 0.00037 | 0.00123 | 0.00052 | AKT1/CTNNB1/EGF/TNF | 4 |
| GO:0050922 | negative regulation of chemotaxis | Apr-97 | 66/18866 | 0.00037 | 0.00123 | 0.00052 | CCL2/CYP19A1/DPP4/GSTP1 | 4 |
| GO:0072171 | mesonephric tubule morphogenesis | Apr-97 | 66/18866 | 0.00037 | 0.00123 | 0.00052 | BCL2/CTNNB1/MYC/VEGFA | 4 |
| GO:1901222 | regulation of NIK/NF-kappaB signaling | May-97 | 119/18866 | 0.00037 | 0.00123 | 0.00053 | EGFR/IL1B/NFKBIA/NR3C2/TNF | 5 |
| GO:0002695 | negative regulation of leukocyte activation | Jun-97 | 184/18866 | 0.00038 | 0.00124 | 0.00053 | CASP3/ERBB2/HMOX1/IL10/IL2/IL4 | 6 |
| GO:0038061 | NIK/NF-kappaB signaling | Jun-97 | 184/18866 | 0.00038 | 0.00124 | 0.00053 | AKT1/EGFR/IL1B/NFKBIA/NR3C2/TNF | 6 |
| GO:0046328 | regulation of JNK cascade | Jun-97 | 184/18866 | 0.00038 | 0.00124 | 0.00053 | AKT1/CD40LG/EGFR/GSTP1/IL1B/TNF | 6 |
| GO:0007611 | learning or memory | Jul-97 | 260/18866 | 0.00039 | 0.00129 | 0.00055 | CASP3/EGFR/FOS/JUN/MAPK1/PTEN/PTGS2 | 7 |
| GO:0050918 | positive chemotaxis | Apr-97 | 67/18866 | 0.00039 | 0.00129 | 0.00055 | CXCL8/F3/F7/VEGFA | 4 |
| GO:1903672 | positive regulation of sprouting angiogenesis | Apr-97 | 67/18866 | 0.00039 | 0.00129 | 0.00055 | HMOX1/IL10/PTGS2/VEGFA | 4 |
| GO:0045830 | positive regulation of isotype switching | Mar-97 | 28/18866 | 0.00039 | 0.00129 | 0.00055 | IL2/IL4/TGFB1 | 3 |
| GO:0045932 | negative regulation of muscle contraction | Mar-97 | 28/18866 | 0.00039 | 0.00129 | 0.00055 | PIK3CG/PTGS2/SOD1 | 3 |
| GO:0060512 | prostate gland morphogenesis | Mar-97 | 28/18866 | 0.00039 | 0.00129 | 0.00055 | AR/ESR1/RXRA | 3 |
| GO:1903579 | negative regulation of ATP metabolic process | Mar-97 | 28/18866 | 0.00039 | 0.00129 | 0.00055 | PPARA/STAT3/TP53 | 3 |
| GO:0034329 | cell junction assembly | Sep-97 | 434/18866 | 0.0004 | 0.0013 | 0.00056 | ACHE/BCL2/CAV1/CTNNB1/IKBKB/IL1B/PTEN/TNF/VEGFA | 9 |
| GO:0010906 | regulation of glucose metabolic process | May-97 | 121/18866 | 0.0004 | 0.00132 | 0.00056 | ADIPOQ/AKT1/IGFBP3/PPARA/TP53 | 5 |
| GO:0048639 | positive regulation of developmental growth | Jun-97 | 187/18866 | 0.00041 | 0.00134 | 0.00057 | AKT1/BCL2/MAPK1/PIM1/PPARD/VEGFA | 6 |
| GO:1904377 | positive regulation of protein localization to cell periphery | Apr-97 | 68/18866 | 0.00042 | 0.00136 | 0.00058 | AKT1/EGFR/IFNG/TNF | 4 |
| GO:1990266 | neutrophil migration | May-97 | 122/18866 | 0.00042 | 0.00136 | 0.00058 | CCL2/CXCL8/DPP4/IL1B/PIK3CG | 5 |
| GO:0071478 | cellular response to radiation | Jun-97 | 188/18866 | 0.00042 | 0.00138 | 0.00059 | BCL2L1/CDKN1A/MYC/PTGS2/RHO/TP53 | 6 |
| GO:0007173 | epidermal growth factor receptor signaling pathway | May-97 | 123/18866 | 0.00043 | 0.00141 | 0.0006 | AKT1/EGF/EGFR/MMP9/TGFB1 | 5 |
| GO:0007369 | gastrulation | Jun-97 | 189/18866 | 0.00043 | 0.00141 | 0.0006 | ADIPOQ/CTNNB1/IL10/MMP2/MMP9/TP53 | 6 |
| GO:0031330 | negative regulation of cellular catabolic process | Jul-97 | 265/18866 | 0.00044 | 0.00142 | 0.00061 | AKT1/BCL2/HMOX1/IL10/PIK3CG/STAT3/TP53 | 7 |
| GO:0019835 | cytolysis | Mar-97 | 29/18866 | 0.00044 | 0.00142 | 0.00061 | F2/LYZ/TGFB1 | 3 |
| GO:0033032 | regulation of myeloid cell apoptotic process | Mar-97 | 29/18866 | 0.00044 | 0.00142 | 0.00061 | ADIPOQ/BCL2/PTEN | 3 |
| GO:0050901 | leukocyte tethering or rolling | Mar-97 | 29/18866 | 0.00044 | 0.00142 | 0.00061 | SELE/TNF/VCAM1 | 3 |
| GO:0060038 | cardiac muscle cell proliferation | Apr-97 | 69/18866 | 0.00044 | 0.00142 | 0.00061 | MAPK1/PIM1/PTEN/RXRA | 4 |
| GO:0071230 | cellular response to amino acid stimulus | Apr-97 | 69/18866 | 0.00044 | 0.00142 | 0.00061 | BCL2L1/EGFR/MMP2/TNF | 4 |
| GO:2000573 | positive regulation of DNA biosynthetic process | Apr-97 | 69/18866 | 0.00044 | 0.00142 | 0.00061 | CTNNB1/MAPK1/MAPK3/MYC | 4 |
| GO:0009165 | nucleotide biosynthetic process | Jul-97 | 266/18866 | 0.00045 | 0.00145 | 0.00062 | IL4/MAPK1/NOS2/NOS3/PTGS2/STAT3/TGFB1 | 7 |
| GO:0010633 | negative regulation of epithelial cell migration | May-97 | 124/18866 | 0.00045 | 0.00146 | 0.00062 | IL4/PPARG/PTEN/TGFB1/TNF | 5 |
| GO:0006879 | cellular iron ion homeostasis | Apr-97 | 70/18866 | 0.00046 | 0.0015 | 0.00064 | HMOX1/IFNG/MYC/SOD1 | 4 |
| GO:1903578 | regulation of ATP metabolic process | May-97 | 125/18866 | 0.00047 | 0.00151 | 0.00064 | IFNG/IL4/PPARA/STAT3/TP53 | 5 |
| GO:0090596 | sensory organ morphogenesis | Jul-97 | 269/18866 | 0.00048 | 0.00154 | 0.00066 | BCL2/CTNNB1/MAPK1/MAPK3/SOD1/STAT3/VEGFA | 7 |
| GO:1901293 | nucleoside phosphate biosynthetic process | Jul-97 | 269/18866 | 0.00048 | 0.00154 | 0.00066 | IL4/MAPK1/NOS2/NOS3/PTGS2/STAT3/TGFB1 | 7 |
| GO:0001844 | protein insertion into mitochondrial membrane involved in apoptotic signaling pathway | Mar-97 | 30/18866 | 0.00048 | 0.00155 | 0.00066 | BCL2/MAPK8/TP53 | 3 |
| GO:0010758 | regulation of macrophage chemotaxis | Mar-97 | 30/18866 | 0.00048 | 0.00155 | 0.00066 | CYP19A1/MAPK1/MAPK3 | 3 |
| GO:0010800 | positive regulation of peptidyl-threonine phosphorylation | Mar-97 | 30/18866 | 0.00048 | 0.00155 | 0.00066 | EGF/MAPK1/TGFB1 | 3 |
| GO:0043457 | regulation of cellular respiration | Mar-97 | 30/18866 | 0.00048 | 0.00155 | 0.00066 | IFNG/IL4/NOS2 | 3 |
| GO:0071480 | cellular response to gamma radiation | Mar-97 | 30/18866 | 0.00048 | 0.00155 | 0.00066 | BCL2L1/CDKN1A/TP53 | 3 |
| GO:2000134 | negative regulation of G1/S transition of mitotic cell cycle | May-97 | 126/18866 | 0.00048 | 0.00155 | 0.00066 | BCL2/CCL2/CDKN1A/PTEN/TP53 | 5 |
| GO:0033627 | cell adhesion mediated by integrin | Apr-97 | 71/18866 | 0.00049 | 0.00157 | 0.00067 | DPP4/ICAM1/PIK3CG/SERPINE1 | 4 |
| GO:0042982 | amyloid precursor protein metabolic process | Apr-97 | 71/18866 | 0.00049 | 0.00157 | 0.00067 | ACHE/CASP3/IFNG/TNF | 4 |
| GO:2000181 | negative regulation of blood vessel morphogenesis | Jun-97 | 194/18866 | 0.0005 | 0.00159 | 0.00068 | ALOX5/CTNNB1/PPARG/SERPINE1/TNF/XDH | 6 |
| GO:0019079 | viral genome replication | May-97 | 127/18866 | 0.0005 | 0.0016 | 0.00068 | BCL2/CCL2/CXCL8/SLPI/TNF | 5 |
| GO:0043254 | regulation of protein-containing complex assembly | Sep-97 | 449/18866 | 0.00051 | 0.00162 | 0.00069 | ESR1/ICAM1/IFNG/MMP1/MMP3/TGFB1/TNF/TP53/VEGFA | 9 |
| GO:0006417 | regulation of translation | Sep-97 | 450/18866 | 0.00052 | 0.00165 | 0.0007 | AKT1/ERBB2/HSPB1/IL6/MAPK1/MAPK3/RXRA/STAT3/TNF | 9 |
| GO:0050663 | cytokine secretion | Apr-97 | 72/18866 | 0.00052 | 0.00165 | 0.0007 | IFNG/IL10/IL1A/TNF | 4 |
| GO:0002040 | sprouting angiogenesis | Jun-97 | 196/18866 | 0.00053 | 0.00167 | 0.00071 | AKT1/ALOX5/HMOX1/IL10/PTGS2/VEGFA | 6 |
| GO:0034368 | protein-lipid complex remodeling | Mar-97 | 31/18866 | 0.00053 | 0.00169 | 0.00072 | ALB/APOB/MPO | 3 |
| GO:0034369 | plasma lipoprotein particle remodeling | Mar-97 | 31/18866 | 0.00053 | 0.00169 | 0.00072 | ALB/APOB/MPO | 3 |
| GO:0071549 | cellular response to dexamethasone stimulus | Mar-97 | 31/18866 | 0.00053 | 0.00169 | 0.00072 | EGFR/ICAM1/NR3C1 | 3 |
| GO:1900745 | positive regulation of p38MAPK cascade | Mar-97 | 31/18866 | 0.00053 | 0.00169 | 0.00072 | IL1B/VEGFA/XDH | 3 |
| GO:0001818 | negative regulation of cytokine production | Aug-97 | 360/18866 | 0.00054 | 0.0017 | 0.00073 | ADIPOQ/F2/GSTP1/HMOX1/IFNG/IL10/TGFB1/TNF | 8 |
| GO:0071482 | cellular response to light stimulus | May-97 | 129/18866 | 0.00054 | 0.00171 | 0.00073 | CDKN1A/MYC/PTGS2/RHO/TP53 | 5 |
| GO:0009749 | response to glucose | Jun-97 | 197/18866 | 0.00054 | 0.00171 | 0.00073 | ADIPOQ/CASP3/ICAM1/PPARD/PTEN/SREBF1 | 6 |
| GO:0051966 | regulation of synaptic transmission, glutamatergic | Apr-97 | 73/18866 | 0.00054 | 0.00172 | 0.00073 | CCL2/EGFR/PTGS2/TNF | 4 |
| GO:0072078 | nephron tubule morphogenesis | Apr-97 | 73/18866 | 0.00054 | 0.00172 | 0.00073 | BCL2/CTNNB1/MYC/VEGFA | 4 |
| GO:1903747 | regulation of establishment of protein localization to mitochondrion | Apr-97 | 73/18866 | 0.00054 | 0.00172 | 0.00073 | BCL2/MAPK8/SREBF1/TP53 | 4 |
| GO:0022898 | regulation of transmembrane transporter activity | Jul-97 | 276/18866 | 0.00056 | 0.00176 | 0.00075 | BCL2/CAV1/CCL2/IFNG/MMP9/PIM1/PTEN | 7 |
| GO:0006635 | fatty acid beta-oxidation | Apr-97 | 74/18866 | 0.00057 | 0.00181 | 0.00077 | ADIPOQ/AKT1/PPARA/PPARD | 4 |
| GO:0051881 | regulation of mitochondrial membrane potential | Apr-97 | 74/18866 | 0.00057 | 0.00181 | 0.00077 | AKT1/BCL2/BCL2L1/SOD1 | 4 |
| GO:1901888 | regulation of cell junction assembly | Jun-97 | 200/18866 | 0.00058 | 0.00184 | 0.00078 | CAV1/IKBKB/IL1B/PTEN/TNF/VEGFA | 6 |
| GO:0034367 | protein-containing complex remodeling | Mar-97 | 32/18866 | 0.00059 | 0.00184 | 0.00078 | ALB/APOB/MPO | 3 |
| GO:0034694 | response to prostaglandin | Mar-97 | 32/18866 | 0.00059 | 0.00184 | 0.00078 | AKT1/APOB/PPARG | 3 |
| GO:0046320 | regulation of fatty acid oxidation | Mar-97 | 32/18866 | 0.00059 | 0.00184 | 0.00078 | AKT1/PPARA/PPARG | 3 |
| GO:0051385 | response to mineralocorticoid | Mar-97 | 32/18866 | 0.00059 | 0.00184 | 0.00078 | CDKN1A/FOS/HSD3B1 | 3 |
| GO:0051968 | positive regulation of synaptic transmission, glutamatergic | Mar-97 | 32/18866 | 0.00059 | 0.00184 | 0.00078 | CCL2/EGFR/PTGS2 | 3 |
| GO:0060603 | mammary gland duct morphogenesis | Mar-97 | 32/18866 | 0.00059 | 0.00184 | 0.00078 | AR/ESR1/PGR | 3 |
| GO:1902807 | negative regulation of cell cycle G1/S phase transition | May-97 | 132/18866 | 0.0006 | 0.00187 | 0.0008 | BCL2/CCL2/CDKN1A/PTEN/TP53 | 5 |
| GO:0035924 | cellular response to vascular endothelial growth factor stimulus | Apr-97 | 75/18866 | 0.0006 | 0.00189 | 0.0008 | AKT1/HSPB1/VEGFA/XDH | 4 |
| GO:0072088 | nephron epithelium morphogenesis | Apr-97 | 75/18866 | 0.0006 | 0.00189 | 0.0008 | BCL2/CTNNB1/MYC/VEGFA | 4 |
| GO:0072091 | regulation of stem cell proliferation | Apr-97 | 76/18866 | 0.00063 | 0.00198 | 0.00084 | CTNNB1/PIM1/TP53/VEGFA | 4 |
| GO:0043280 | positive regulation of cysteine-type endopeptidase activity involved in apoptotic process | May-97 | 134/18866 | 0.00064 | 0.002 | 0.00085 | F3/MYC/PPARG/TNF/XDH | 5 |
| GO:0010039 | response to iron ion | Mar-97 | 33/18866 | 0.00064 | 0.002 | 0.00085 | BCL2/CYP1A1/HMOX1 | 3 |
| GO:0010464 | regulation of mesenchymal cell proliferation | Mar-97 | 33/18866 | 0.00064 | 0.002 | 0.00085 | CTNNB1/MYC/VEGFA | 3 |
| GO:0045736 | negative regulation of cyclin-dependent protein serine/threonine kinase activity | Mar-97 | 33/18866 | 0.00064 | 0.002 | 0.00085 | CASP3/CDKN1A/PTEN | 3 |
| GO:1902003 | regulation of amyloid-beta formation | Mar-97 | 33/18866 | 0.00064 | 0.002 | 0.00085 | CASP3/IFNG/TNF | 3 |
| GO:0007422 | peripheral nervous system development | Apr-97 | 77/18866 | 0.00067 | 0.00207 | 0.00088 | AKT1/ERBB2/ERBB3/SOD1 | 4 |
| GO:0061333 | renal tubule morphogenesis | Apr-97 | 77/18866 | 0.00067 | 0.00207 | 0.00088 | BCL2/CTNNB1/MYC/VEGFA | 4 |
| GO:0072028 | nephron morphogenesis | Apr-97 | 77/18866 | 0.00067 | 0.00207 | 0.00088 | BCL2/CTNNB1/MYC/VEGFA | 4 |
| GO:0003012 | muscle system process | Sep-97 | 467/18866 | 0.00067 | 0.00208 | 0.00089 | CAV1/HMOX1/IL1B/NOS3/PIK3CG/PPARA/PTGER3/PTGS2/SOD1 | 9 |
| GO:1901343 | negative regulation of vasculature development | Jun-97 | 207/18866 | 0.0007 | 0.00216 | 0.00092 | ALOX5/CTNNB1/PPARG/SERPINE1/TNF/XDH | 6 |
| GO:0016242 | negative regulation of macroautophagy | Mar-97 | 34/18866 | 0.0007 | 0.00216 | 0.00092 | AKT1/HMOX1/TP53 | 3 |
| GO:0032212 | positive regulation of telomere maintenance via telomerase | Mar-97 | 34/18866 | 0.0007 | 0.00216 | 0.00092 | CTNNB1/MAPK1/MAPK3 | 3 |
| GO:0035633 | maintenance of blood-brain barrier | Mar-97 | 34/18866 | 0.0007 | 0.00216 | 0.00092 | IL6/PTGS2/VEGFA | 3 |
| GO:0043276 | anoikis | Mar-97 | 34/18866 | 0.0007 | 0.00216 | 0.00092 | AKT1/BCL2/CAV1 | 3 |
| GO:1902692 | regulation of neuroblast proliferation | Mar-97 | 34/18866 | 0.0007 | 0.00216 | 0.00092 | CTNNB1/TP53/VEGFA | 3 |
| GO:1904030 | negative regulation of cyclin-dependent protein kinase activity | Mar-97 | 34/18866 | 0.0007 | 0.00216 | 0.00092 | CASP3/CDKN1A/PTEN | 3 |
| GO:2000352 | negative regulation of endothelial cell apoptotic process | Mar-97 | 34/18866 | 0.0007 | 0.00216 | 0.00092 | ICAM1/IL4/SERPINE1 | 3 |
| GO:0042552 | myelination | May-97 | 137/18866 | 0.00071 | 0.00218 | 0.00093 | AKT1/CTNNB1/ERBB2/PTEN/SOD1 | 5 |
| GO:0006638 | neutral lipid metabolic process | May-97 | 138/18866 | 0.00073 | 0.00225 | 0.00096 | APOB/CAT/CAV1/PIK3CG/SREBF1 | 5 |
| GO:0006639 | acylglycerol metabolic process | May-97 | 138/18866 | 0.00073 | 0.00225 | 0.00096 | APOB/CAT/CAV1/PIK3CG/SREBF1 | 5 |
| GO:0010827 | regulation of glucose transmembrane transport | Apr-97 | 79/18866 | 0.00073 | 0.00225 | 0.00096 | ADIPOQ/AKT1/IL1B/TNF | 4 |
| GO:0016575 | histone deacetylation | Apr-97 | 79/18866 | 0.00073 | 0.00225 | 0.00096 | MAPK8/SREBF1/TP53/VEGFA | 4 |
| GO:0043407 | negative regulation of MAP kinase activity | Apr-97 | 79/18866 | 0.00073 | 0.00225 | 0.00096 | ADIPOQ/CAV1/GSTP1/IL1B | 4 |
| GO:0050672 | negative regulation of lymphocyte proliferation | Apr-97 | 79/18866 | 0.00073 | 0.00225 | 0.00096 | CASP3/ERBB2/IL10/IL2 | 4 |
| GO:0007435 | salivary gland morphogenesis | Mar-97 | 35/18866 | 0.00077 | 0.00233 | 0.00099 | EGFR/TGFB1/TNF | 3 |
| GO:0060251 | regulation of glial cell proliferation | Mar-97 | 35/18866 | 0.00077 | 0.00233 | 0.00099 | IL1B/IL6/TNF | 3 |
| GO:0071312 | cellular response to alkaloid | Mar-97 | 35/18866 | 0.00077 | 0.00233 | 0.00099 | BCL2L1/CASP3/ICAM1 | 3 |
| GO:0110111 | negative regulation of animal organ morphogenesis | Mar-97 | 35/18866 | 0.00077 | 0.00233 | 0.00099 | BCL2/CTNNB1/TNF | 3 |
| GO:1901030 | positive regulation of mitochondrial outer membrane permeabilization involved in apoptotic signaling pathway | Mar-97 | 35/18866 | 0.00077 | 0.00233 | 0.00099 | BCL2/MAPK8/TP53 | 3 |
| GO:1904031 | positive regulation of cyclin-dependent protein kinase activity | Mar-97 | 35/18866 | 0.00077 | 0.00233 | 0.00099 | AKT1/CDKN1A/EGFR | 3 |
| GO:0032204 | regulation of telomere maintenance | Apr-97 | 80/18866 | 0.00077 | 0.00234 | 0.001 | CTNNB1/MAPK1/MAPK3/MYC | 4 |
| GO:0055021 | regulation of cardiac muscle tissue growth | Apr-97 | 80/18866 | 0.00077 | 0.00234 | 0.001 | MAPK1/PIM1/PPARA/PTEN | 4 |
| GO:1901224 | positive regulation of NIK/NF-kappaB signaling | Apr-97 | 80/18866 | 0.00077 | 0.00234 | 0.001 | EGFR/IL1B/NR3C2/TNF | 4 |
| GO:0072655 | establishment of protein localization to mitochondrion | May-97 | 140/18866 | 0.00078 | 0.00237 | 0.00101 | AKT1/BCL2/MAPK8/SREBF1/TP53 | 5 |
| GO:0008584 | male gonad development | May-97 | 141/18866 | 0.00081 | 0.00244 | 0.00104 | AR/BCL2/BCL2L1/ESR1/ICAM1 | 5 |
| GO:0034644 | cellular response to UV | Apr-97 | 81/18866 | 0.00081 | 0.00244 | 0.00104 | CDKN1A/MYC/PTGS2/TP53 | 4 |
| GO:0044262 | cellular carbohydrate metabolic process | Jul-97 | 294/18866 | 0.00081 | 0.00244 | 0.00104 | ADIPOQ/AKT1/IGFBP3/PPARA/PTEN/STAT3/TP53 | 7 |
| GO:0010543 | regulation of platelet activation | Mar-97 | 36/18866 | 0.00083 | 0.0025 | 0.00107 | F2/NOS3/THBD | 3 |
| GO:0034405 | response to fluid shear stress | Mar-97 | 36/18866 | 0.00083 | 0.0025 | 0.00107 | AKT1/NOS3/PTGS2 | 3 |
| GO:0043368 | positive T cell selection | Mar-97 | 36/18866 | 0.00083 | 0.0025 | 0.00107 | BCL2/IL6/STAT3 | 3 |
| GO:0045742 | positive regulation of epidermal growth factor receptor signaling pathway | Mar-97 | 36/18866 | 0.00083 | 0.0025 | 0.00107 | AKT1/EGF/MMP9 | 3 |
| GO:1905332 | positive regulation of morphogenesis of an epithelium | Mar-97 | 36/18866 | 0.00083 | 0.0025 | 0.00107 | AR/CTNNB1/VEGFA | 3 |
| GO:0007006 | mitochondrial membrane organization | May-97 | 142/18866 | 0.00083 | 0.0025 | 0.00107 | BCL2/BCL2L1/MAPK8/STAT3/TP53 | 5 |
| GO:0007292 | female gamete generation | May-97 | 142/18866 | 0.00083 | 0.0025 | 0.00107 | BCL2/CTNNB1/NOS3/PGR/PTGS2 | 5 |
| GO:0046546 | development of primary male sexual characteristics | May-97 | 142/18866 | 0.00083 | 0.0025 | 0.00107 | AR/BCL2/BCL2L1/ESR1/ICAM1 | 5 |
| GO:0005996 | monosaccharide metabolic process | Jul-97 | 296/18866 | 0.00084 | 0.00252 | 0.00108 | ADIPOQ/AKT1/IGFBP3/PPARA/PPARD/TNF/TP53 | 7 |
| GO:0072006 | nephron development | May-97 | 143/18866 | 0.00086 | 0.00258 | 0.0011 | ADIPOQ/BCL2/CTNNB1/MYC/VEGFA | 5 |
| GO:0070585 | protein localization to mitochondrion | May-97 | 144/18866 | 0.00089 | 0.00266 | 0.00113 | AKT1/BCL2/MAPK8/SREBF1/TP53 | 5 |
| GO:0106106 | cold-induced thermogenesis | May-97 | 144/18866 | 0.00089 | 0.00266 | 0.00113 | ACHE/ADIPOQ/CAV1/IL4/VEGFA | 5 |
| GO:0120161 | regulation of cold-induced thermogenesis | May-97 | 144/18866 | 0.00089 | 0.00266 | 0.00113 | ACHE/ADIPOQ/CAV1/IL4/VEGFA | 5 |
| GO:0050792 | regulation of viral process | Jun-97 | 217/18866 | 0.00089 | 0.00268 | 0.00114 | BCL2/CRP/CXCL8/JUN/SLPI/TNF | 6 |
| GO:0090050 | positive regulation of cell migration involved in sprouting angiogenesis | Mar-97 | 37/18866 | 0.0009 | 0.00269 | 0.00115 | HMOX1/PTGS2/VEGFA | 3 |
| GO:0097242 | amyloid-beta clearance | Mar-97 | 37/18866 | 0.0009 | 0.00269 | 0.00115 | IFNG/IL4/TNF | 3 |
| GO:1904358 | positive regulation of telomere maintenance via telomere lengthening | Mar-97 | 37/18866 | 0.0009 | 0.00269 | 0.00115 | CTNNB1/MAPK1/MAPK3 | 3 |
| GO:2000273 | positive regulation of signaling receptor activity | Mar-97 | 37/18866 | 0.0009 | 0.00269 | 0.00115 | CCL2/EGF/IFNG | 3 |
| GO:0045913 | positive regulation of carbohydrate metabolic process | Apr-97 | 84/18866 | 0.00092 | 0.00276 | 0.00118 | AKT1/EGF/IFNG/PPARA | 4 |
| GO:0044344 | cellular response to fibroblast growth factor stimulus | May-97 | 146/18866 | 0.00094 | 0.00281 | 0.0012 | CCL2/CTNNB1/CXCL8/MAPK1/MAPK3 | 5 |
| GO:0007043 | cell-cell junction assembly | May-97 | 147/18866 | 0.00097 | 0.00289 | 0.00123 | CAV1/CTNNB1/IKBKB/IL1B/TNF | 5 |
| GO:0003156 | regulation of animal organ formation | Mar-97 | 38/18866 | 0.00098 | 0.00289 | 0.00123 | AR/CTNNB1/PIM1 | 3 |
| GO:0032885 | regulation of polysaccharide biosynthetic process | Mar-97 | 38/18866 | 0.00098 | 0.00289 | 0.00123 | AKT1/EGF/TGFB1 | 3 |
| GO:0048009 | insulin-like growth factor receptor signaling pathway | Mar-97 | 38/18866 | 0.00098 | 0.00289 | 0.00123 | AKT1/AR/IGFBP3 | 3 |
| GO:0071392 | cellular response to estradiol stimulus | Mar-97 | 38/18866 | 0.00098 | 0.00289 | 0.00123 | EGFR/ESR1/IL10 | 3 |
| GO:1901186 | positive regulation of ERBB signaling pathway | Mar-97 | 38/18866 | 0.00098 | 0.00289 | 0.00123 | AKT1/EGF/MMP9 | 3 |
| GO:1903670 | regulation of sprouting angiogenesis | May-97 | 148/18866 | 0.001 | 0.00297 | 0.00127 | ALOX5/HMOX1/IL10/PTGS2/VEGFA | 5 |
| GO:0019722 | calcium-mediated signaling | Jun-97 | 222/18866 | 0.00101 | 0.00298 | 0.00127 | CXCL8/EGFR/ERBB3/SELE/TNF/VCAM1 | 6 |
| GO:0006919 | activation of cysteine-type endopeptidase activity involved in apoptotic process | Apr-97 | 86/18866 | 0.00101 | 0.00298 | 0.00127 | F3/PPARG/TNF/XDH | 4 |
| GO:0009791 | post-embryonic development | Apr-97 | 86/18866 | 0.00101 | 0.00298 | 0.00127 | APOB/BCL2/CYP1A2/VEGFA | 4 |
| GO:0045445 | myoblast differentiation | Apr-97 | 86/18866 | 0.00101 | 0.00298 | 0.00127 | IGFBP3/PPARD/TGFB1/TNF | 4 |
| GO:2000106 | regulation of leukocyte apoptotic process | Apr-97 | 86/18866 | 0.00101 | 0.00298 | 0.00127 | IL10/IL2/PTEN/TP53 | 4 |
| GO:0006778 | porphyrin-containing compound metabolic process | Mar-97 | 39/18866 | 0.00105 | 0.00309 | 0.00132 | CYP1A1/CYP1A2/HMOX1 | 3 |
| GO:0007431 | salivary gland development | Mar-97 | 39/18866 | 0.00105 | 0.00309 | 0.00132 | EGFR/TGFB1/TNF | 3 |
| GO:0014037 | Schwann cell differentiation | Mar-97 | 39/18866 | 0.00105 | 0.00309 | 0.00132 | AKT1/ERBB3/SOD1 | 3 |
| GO:0034205 | amyloid-beta formation | Mar-97 | 39/18866 | 0.00105 | 0.00309 | 0.00132 | CASP3/IFNG/TNF | 3 |
| GO:0042092 | type 2 immune response | Mar-97 | 39/18866 | 0.00105 | 0.00309 | 0.00132 | IL10/IL4/IL6 | 3 |
| GO:0060416 | response to growth hormone | Mar-97 | 39/18866 | 0.00105 | 0.00309 | 0.00132 | AKT1/F7/STAT3 | 3 |
| GO:0001942 | hair follicle development | Apr-97 | 87/18866 | 0.00105 | 0.00309 | 0.00132 | BCL2/CTNNB1/EGFR/TNF | 4 |
| GO:0055072 | iron ion homeostasis | Apr-97 | 87/18866 | 0.00105 | 0.00309 | 0.00132 | HMOX1/IFNG/MYC/SOD1 | 4 |
| GO:0060420 | regulation of heart growth | Apr-97 | 87/18866 | 0.00105 | 0.00309 | 0.00132 | MAPK1/PIM1/PPARA/PTEN | 4 |
| GO:0035264 | multicellular organism growth | May-97 | 150/18866 | 0.00106 | 0.00312 | 0.00133 | AR/BCL2/SOD1/STAT3/TP53 | 5 |
| GO:0008643 | carbohydrate transport | May-97 | 151/18866 | 0.0011 | 0.0032 | 0.00137 | ADIPOQ/AKT1/IL1B/PPARD/TNF | 5 |
| GO:2001056 | positive regulation of cysteine-type endopeptidase activity | May-97 | 151/18866 | 0.0011 | 0.0032 | 0.00137 | F3/MYC/PPARG/TNF/XDH | 5 |
| GO:0016236 | macroautophagy | Jul-97 | 310/18866 | 0.0011 | 0.00321 | 0.00137 | AKT1/CASP3/HMOX1/IL4/MAPK3/MAPK8/TP53 | 7 |
| GO:0001837 | epithelial to mesenchymal transition | May-97 | 152/18866 | 0.00113 | 0.00329 | 0.0014 | CTNNB1/IL1B/IL6/PTEN/TGFB1 | 5 |
| GO:0002224 | toll-like receptor signaling pathway | May-97 | 152/18866 | 0.00113 | 0.00329 | 0.0014 | APOB/CAV1/ESR1/IKBKB/NFKBIA | 5 |
| GO:0045740 | positive regulation of DNA replication | Mar-97 | 40/18866 | 0.00113 | 0.0033 | 0.00141 | EGF/EGFR/JUN | 3 |
| GO:0090184 | positive regulation of kidney development | Mar-97 | 40/18866 | 0.00113 | 0.0033 | 0.00141 | ADIPOQ/MYC/VEGFA | 3 |
| GO:1902991 | regulation of amyloid precursor protein catabolic process | Mar-97 | 40/18866 | 0.00113 | 0.0033 | 0.00141 | CASP3/IFNG/TNF | 3 |
| GO:1903427 | negative regulation of reactive oxygen species biosynthetic process | Mar-97 | 40/18866 | 0.00113 | 0.0033 | 0.00141 | CAV1/IL10/STAT3 | 3 |
| GO:0010749 | regulation of nitric oxide mediated signal transduction | Feb-97 | 10/18866 | 0.00115 | 0.00331 | 0.00141 | EGFR/VEGFA | 2 |
| GO:0045713 | low-density lipoprotein particle receptor biosynthetic process | Feb-97 | 10/18866 | 0.00115 | 0.00331 | 0.00141 | ADIPOQ/PPARG | 2 |
| GO:0045792 | negative regulation of cell size | Feb-97 | 10/18866 | 0.00115 | 0.00331 | 0.00141 | AKT1/PTEN | 2 |
| GO:0048304 | positive regulation of isotype switching to IgG isotypes | Feb-97 | 10/18866 | 0.00115 | 0.00331 | 0.00141 | IL2/IL4 | 2 |
| GO:0060768 | regulation of epithelial cell proliferation involved in prostate gland development | Feb-97 | 10/18866 | 0.00115 | 0.00331 | 0.00141 | AR/CTNNB1 | 2 |
| GO:0071394 | cellular response to testosterone stimulus | Feb-97 | 10/18866 | 0.00115 | 0.00331 | 0.00141 | AR/SPP1 | 2 |
| GO:1900222 | negative regulation of amyloid-beta clearance | Feb-97 | 10/18866 | 0.00115 | 0.00331 | 0.00141 | IFNG/TNF | 2 |
| GO:2000320 | negative regulation of T-helper 17 cell differentiation | Feb-97 | 10/18866 | 0.00115 | 0.00331 | 0.00141 | IL2/IL4 | 2 |
| GO:0001892 | embryonic placenta development | Apr-97 | 89/18866 | 0.00115 | 0.00331 | 0.00141 | AKT1/EGFR/IL10/MAPK1 | 4 |
| GO:0022404 | molting cycle process | Apr-97 | 89/18866 | 0.00115 | 0.00331 | 0.00141 | BCL2/CTNNB1/EGFR/TNF | 4 |
| GO:0022405 | hair cycle process | Apr-97 | 89/18866 | 0.00115 | 0.00331 | 0.00141 | BCL2/CTNNB1/EGFR/TNF | 4 |
| GO:0071774 | response to fibroblast growth factor | May-97 | 153/18866 | 0.00116 | 0.00335 | 0.00143 | CCL2/CTNNB1/CXCL8/MAPK1/MAPK3 | 5 |
| GO:0042058 | regulation of epidermal growth factor receptor signaling pathway | Apr-97 | 90/18866 | 0.0012 | 0.00344 | 0.00146 | AKT1/EGF/EGFR/MMP9 | 4 |
| GO:0043470 | regulation of carbohydrate catabolic process | Apr-97 | 90/18866 | 0.0012 | 0.00344 | 0.00146 | IFNG/PPARA/STAT3/TP53 | 4 |
| GO:0098773 | skin epidermis development | Apr-97 | 90/18866 | 0.0012 | 0.00344 | 0.00146 | BCL2/CTNNB1/EGFR/TNF | 4 |
| GO:0051250 | negative regulation of lymphocyte activation | May-97 | 154/18866 | 0.0012 | 0.00344 | 0.00146 | CASP3/ERBB2/IL10/IL2/IL4 | 5 |
| GO:0007160 | cell-matrix adhesion | Jun-97 | 230/18866 | 0.00121 | 0.00346 | 0.00148 | BCL2/CTNNB1/PTEN/SERPINE1/VCAM1/VEGFA | 6 |
| GO:0002251 | organ or tissue specific immune response | Mar-97 | 41/18866 | 0.00122 | 0.00349 | 0.00149 | IL4/IL6/NOS2 | 3 |
| GO:0043267 | negative regulation of potassium ion transport | Mar-97 | 41/18866 | 0.00122 | 0.00349 | 0.00149 | CAV1/NOS3/PTEN | 3 |
| GO:0071548 | response to dexamethasone | Mar-97 | 41/18866 | 0.00122 | 0.00349 | 0.00149 | EGFR/ICAM1/NR3C1 | 3 |
| GO:1900117 | regulation of execution phase of apoptosis | Mar-97 | 41/18866 | 0.00122 | 0.00349 | 0.00149 | BCL2L1/IL6/TP53 | 3 |
| GO:1990845 | adaptive thermogenesis | May-97 | 155/18866 | 0.00123 | 0.00352 | 0.0015 | ACHE/ADIPOQ/CAV1/IL4/VEGFA | 5 |
| GO:0001656 | metanephros development | Apr-97 | 91/18866 | 0.00125 | 0.00356 | 0.00152 | ADIPOQ/BCL2/CTNNB1/MYC | 4 |
| GO:2000177 | regulation of neural precursor cell proliferation | Apr-97 | 91/18866 | 0.00125 | 0.00356 | 0.00152 | CTNNB1/EGF/TP53/VEGFA | 4 |
| GO:0031341 | regulation of cell killing | Apr-97 | 92/18866 | 0.0013 | 0.0037 | 0.00158 | ICAM1/IFNG/IL4/NOS2 | 4 |
| GO:0034383 | low-density lipoprotein particle clearance | Mar-97 | 42/18866 | 0.00131 | 0.00372 | 0.00159 | ADIPOQ/APOB/HMOX1 | 3 |
| GO:0090279 | regulation of calcium ion import | Mar-97 | 42/18866 | 0.00131 | 0.00372 | 0.00159 | CCL2/CTNNB1/EGF | 3 |
| GO:1904037 | positive regulation of epithelial cell apoptotic process | Mar-97 | 42/18866 | 0.00131 | 0.00372 | 0.00159 | CCL2/CD40LG/IL6 | 3 |
| GO:0007050 | cell cycle arrest | Jun-97 | 234/18866 | 0.00132 | 0.00375 | 0.0016 | CDKN1A/CXCL8/IFNG/MYC/TGFB1/TP53 | 6 |
| GO:0007519 | skeletal muscle tissue development | May-97 | 158/18866 | 0.00134 | 0.00381 | 0.00162 | BCL2/CAV1/CTNNB1/FOS/TGFB1 | 5 |
| GO:0030203 | glycosaminoglycan metabolic process | May-97 | 158/18866 | 0.00134 | 0.00381 | 0.00162 | AKT1/EGF/IL1B/PIM1/TGFB1 | 5 |
| GO:0045185 | maintenance of protein location | Apr-97 | 93/18866 | 0.00135 | 0.00383 | 0.00163 | AKT1/CAV1/IL10/NFKBIA | 4 |
| GO:0072080 | nephron tubule development | Apr-97 | 93/18866 | 0.00135 | 0.00383 | 0.00163 | BCL2/CTNNB1/MYC/VEGFA | 4 |
| GO:0007204 | positive regulation of cytosolic calcium ion concentration | Jul-97 | 322/18866 | 0.00137 | 0.00387 | 0.00165 | BCL2/CAV1/ESR1/F2/IL2/PIK3CG/PTGER3 | 7 |
| GO:0044242 | cellular lipid catabolic process | Jun-97 | 236/18866 | 0.00138 | 0.00389 | 0.00166 | ADIPOQ/AKT1/APOB/PIK3CG/PPARA/PPARD | 6 |
| GO:0003263 | cardioblast proliferation | Feb-97 | 11/18866 | 0.0014 | 0.00389 | 0.00166 | CTNNB1/PIM1 | 2 |
| GO:0003264 | regulation of cardioblast proliferation | Feb-97 | 11/18866 | 0.0014 | 0.00389 | 0.00166 | CTNNB1/PIM1 | 2 |
| GO:0006527 | arginine catabolic process | Feb-97 | 11/18866 | 0.0014 | 0.00389 | 0.00166 | NOS2/NOS3 | 2 |
| GO:0031284 | positive regulation of guanylate cyclase activity | Feb-97 | 11/18866 | 0.0014 | 0.00389 | 0.00166 | NOS2/NOS3 | 2 |
| GO:0033197 | response to vitamin E | Feb-97 | 11/18866 | 0.0014 | 0.00389 | 0.00166 | CAT/PPARG | 2 |
| GO:0033483 | gas homeostasis | Feb-97 | 11/18866 | 0.0014 | 0.00389 | 0.00166 | CAV1/GSTP1 | 2 |
| GO:0034115 | negative regulation of heterotypic cell-cell adhesion | Feb-97 | 11/18866 | 0.0014 | 0.00389 | 0.00166 | ADIPOQ/IL10 | 2 |
| GO:0035747 | natural killer cell chemotaxis | Feb-97 | 11/18866 | 0.0014 | 0.00389 | 0.00166 | CCL2/PIK3CG | 2 |
| GO:0042447 | hormone catabolic process | Feb-97 | 11/18866 | 0.0014 | 0.00389 | 0.00166 | CYP19A1/SPP1 | 2 |
| GO:0042789 | mRNA transcription by RNA polymerase II | Feb-97 | 11/18866 | 0.0014 | 0.00389 | 0.00166 | SREBF1/STAT3 | 2 |
| GO:0045899 | positive regulation of RNA polymerase II transcription preinitiation complex assembly | Feb-97 | 11/18866 | 0.0014 | 0.00389 | 0.00166 | ESR1/TP53 | 2 |
| GO:0051024 | positive regulation of immunoglobulin secretion | Feb-97 | 11/18866 | 0.0014 | 0.00389 | 0.00166 | IL2/IL6 | 2 |
| GO:0051712 | positive regulation of killing of cells of other organism | Feb-97 | 11/18866 | 0.0014 | 0.00389 | 0.00166 | IFNG/NOS2 | 2 |
| GO:0051974 | negative regulation of telomerase activity | Feb-97 | 11/18866 | 0.0014 | 0.00389 | 0.00166 | PPARG/TP53 | 2 |
| GO:0060525 | prostate glandular acinus development | Feb-97 | 11/18866 | 0.0014 | 0.00389 | 0.00166 | ESR1/RXRA | 2 |
| GO:0060767 | epithelial cell proliferation involved in prostate gland development | Feb-97 | 11/18866 | 0.0014 | 0.00389 | 0.00166 | AR/CTNNB1 | 2 |
| GO:0061307 | cardiac neural crest cell differentiation involved in heart development | Feb-97 | 11/18866 | 0.0014 | 0.00389 | 0.00166 | MAPK1/MAPK3 | 2 |
| GO:0061308 | cardiac neural crest cell development involved in heart development | Feb-97 | 11/18866 | 0.0014 | 0.00389 | 0.00166 | MAPK1/MAPK3 | 2 |
| GO:0070587 | regulation of cell-cell adhesion involved in gastrulation | Feb-97 | 11/18866 | 0.0014 | 0.00389 | 0.00166 | ADIPOQ/IL10 | 2 |
| GO:0070673 | response to interleukin-18 | Feb-97 | 11/18866 | 0.0014 | 0.00389 | 0.00166 | AKT1/ALOX5 | 2 |
| GO:2000317 | negative regulation of T-helper 17 type immune response | Feb-97 | 11/18866 | 0.0014 | 0.00389 | 0.00166 | IL2/IL4 | 2 |
| GO:0002369 | T cell cytokine production | Mar-97 | 43/18866 | 0.0014 | 0.00389 | 0.00166 | IL1B/IL4/IL6 | 3 |
| GO:0045124 | regulation of bone resorption | Mar-97 | 43/18866 | 0.0014 | 0.00389 | 0.00166 | EGFR/IL6/SPP1 | 3 |
| GO:0045778 | positive regulation of ossification | Apr-97 | 94/18866 | 0.00141 | 0.0039 | 0.00166 | ALOX5/CTNNB1/IL6/TGFB1 | 4 |
| GO:0060993 | kidney morphogenesis | Apr-97 | 94/18866 | 0.00141 | 0.0039 | 0.00166 | BCL2/CTNNB1/MYC/VEGFA | 4 |
| GO:0061326 | renal tubule development | Apr-97 | 95/18866 | 0.00146 | 0.00406 | 0.00173 | BCL2/CTNNB1/MYC/VEGFA | 4 |
| GO:0010463 | mesenchymal cell proliferation | Mar-97 | 44/18866 | 0.0015 | 0.00414 | 0.00176 | CTNNB1/MYC/VEGFA | 3 |
| GO:0031670 | cellular response to nutrient | Mar-97 | 44/18866 | 0.0015 | 0.00414 | 0.00176 | HMOX1/PIM1/PPARG | 3 |
| GO:0032881 | regulation of polysaccharide metabolic process | Mar-97 | 44/18866 | 0.0015 | 0.00414 | 0.00176 | AKT1/EGF/TGFB1 | 3 |
| GO:1903053 | regulation of extracellular matrix organization | Mar-97 | 44/18866 | 0.0015 | 0.00414 | 0.00176 | DPP4/IL6/TGFB1 | 3 |
| GO:1905521 | regulation of macrophage migration | Mar-97 | 44/18866 | 0.0015 | 0.00414 | 0.00176 | CYP19A1/MAPK1/MAPK3 | 3 |
| GO:0035249 | synaptic transmission, glutamatergic | Apr-97 | 96/18866 | 0.00152 | 0.0042 | 0.00179 | CCL2/EGFR/PTGS2/TNF | 4 |
| GO:0038093 | Fc receptor signaling pathway | Jun-97 | 241/18866 | 0.00153 | 0.00423 | 0.0018 | FOS/IKBKB/JUN/MAPK1/MAPK3/MAPK8 | 6 |
| GO:0010677 | negative regulation of cellular carbohydrate metabolic process | Mar-97 | 45/18866 | 0.0016 | 0.00439 | 0.00187 | ADIPOQ/PPARA/STAT3 | 3 |
| GO:0034105 | positive regulation of tissue remodeling | Mar-97 | 45/18866 | 0.0016 | 0.00439 | 0.00187 | EGFR/IL2/SPP1 | 3 |
| GO:0042771 | intrinsic apoptotic signaling pathway in response to DNA damage by p53 class mediator | Mar-97 | 45/18866 | 0.0016 | 0.00439 | 0.00187 | BCL2/CDKN1A/TP53 | 3 |
| GO:0045911 | positive regulation of DNA recombination | Mar-97 | 45/18866 | 0.0016 | 0.00439 | 0.00187 | IL2/IL4/TGFB1 | 3 |
| GO:0051204 | protein insertion into mitochondrial membrane | Mar-97 | 45/18866 | 0.0016 | 0.00439 | 0.00187 | BCL2/MAPK8/TP53 | 3 |
| GO:0070266 | necroptotic process | Mar-97 | 45/18866 | 0.0016 | 0.00439 | 0.00187 | CAV1/TNF/TP53 | 3 |
| GO:1901028 | regulation of mitochondrial outer membrane permeabilization involved in apoptotic signaling pathway | Mar-97 | 45/18866 | 0.0016 | 0.00439 | 0.00187 | BCL2/MAPK8/TP53 | 3 |
| GO:0031392 | regulation of prostaglandin biosynthetic process | Feb-97 | 12/18866 | 0.00167 | 0.00455 | 0.00194 | IL1B/PTGS2 | 2 |
| GO:0042178 | xenobiotic catabolic process | Feb-97 | 12/18866 | 0.00167 | 0.00455 | 0.00194 | CYP1A1/GSTM1 | 2 |
| GO:0060442 | branching involved in prostate gland morphogenesis | Feb-97 | 12/18866 | 0.00167 | 0.00455 | 0.00194 | ESR1/RXRA | 2 |
| GO:0061517 | macrophage proliferation | Feb-97 | 12/18866 | 0.00167 | 0.00455 | 0.00194 | MAPK1/MAPK3 | 2 |
| GO:0070586 | cell-cell adhesion involved in gastrulation | Feb-97 | 12/18866 | 0.00167 | 0.00455 | 0.00194 | ADIPOQ/IL10 | 2 |
| GO:0071639 | positive regulation of monocyte chemotactic protein-1 production | Feb-97 | 12/18866 | 0.00167 | 0.00455 | 0.00194 | ADIPOQ/IL1B | 2 |
| GO:0072540 | T-helper 17 cell lineage commitment | Feb-97 | 12/18866 | 0.00167 | 0.00455 | 0.00194 | IL6/STAT3 | 2 |
| GO:0097284 | hepatocyte apoptotic process | Feb-97 | 12/18866 | 0.00167 | 0.00455 | 0.00194 | BCL2L1/PIK3CG | 2 |
| GO:0150065 | regulation of deacetylase activity | Feb-97 | 12/18866 | 0.00167 | 0.00455 | 0.00194 | MAPK8/VEGFA | 2 |
| GO:0050830 | defense response to Gram-positive bacterium | Apr-97 | 99/18866 | 0.0017 | 0.00462 | 0.00197 | CRP/IL6/LYZ/TNF | 4 |
| GO:0051817 | modulation of process of other organism involved in symbiotic interaction | Apr-97 | 99/18866 | 0.0017 | 0.00462 | 0.00197 | BCL2L1/CRP/JUN/RXRA | 4 |
| GO:0010863 | positive regulation of phospholipase C activity | Mar-97 | 46/18866 | 0.0017 | 0.00462 | 0.00197 | EGFR/ESR1/SELE | 3 |
| GO:0017144 | drug metabolic process | Mar-97 | 46/18866 | 0.0017 | 0.00462 | 0.00197 | CYP1A1/CYP1A2/CYP3A4 | 3 |
| GO:0031641 | regulation of myelination | Mar-97 | 46/18866 | 0.0017 | 0.00462 | 0.00197 | AKT1/CTNNB1/PTEN | 3 |
| GO:0035987 | endodermal cell differentiation | Mar-97 | 46/18866 | 0.0017 | 0.00462 | 0.00197 | CTNNB1/MMP2/MMP9 | 3 |
| GO:0045581 | negative regulation of T cell differentiation | Mar-97 | 46/18866 | 0.0017 | 0.00462 | 0.00197 | ERBB2/IL2/IL4 | 3 |
| GO:1900744 | regulation of p38MAPK cascade | Mar-97 | 46/18866 | 0.0017 | 0.00462 | 0.00197 | IL1B/VEGFA/XDH | 3 |
| GO:0060538 | skeletal muscle organ development | May-97 | 168/18866 | 0.00176 | 0.00476 | 0.00203 | BCL2/CAV1/CTNNB1/FOS/TGFB1 | 5 |
| GO:0045069 | regulation of viral genome replication | Apr-97 | 100/18866 | 0.00177 | 0.00478 | 0.00204 | BCL2/CXCL8/SLPI/TNF | 4 |
| GO:0046486 | glycerolipid metabolic process | Aug-97 | 434/18866 | 0.00178 | 0.00482 | 0.00206 | ACHE/APOB/CAT/CAV1/PIK3CG/PON1/PTEN/SREBF1 | 8 |
| GO:0007157 | heterophilic cell-cell adhesion via plasma membrane cell adhesion molecules | Mar-97 | 47/18866 | 0.00181 | 0.00489 | 0.00208 | ICAM1/SELE/VCAM1 | 3 |
| GO:0035722 | interleukin-12-mediated signaling pathway | Mar-97 | 47/18866 | 0.00181 | 0.00489 | 0.00208 | IFNG/IL10/SOD1 | 3 |
| GO:0035850 | epithelial cell differentiation involved in kidney development | Mar-97 | 47/18866 | 0.00181 | 0.00489 | 0.00208 | ADIPOQ/CTNNB1/MMP9 | 3 |
| GO:0042572 | retinol metabolic process | Mar-97 | 47/18866 | 0.00181 | 0.00489 | 0.00208 | CYP1A1/CYP1A2/CYP3A4 | 3 |
| GO:0043303 | mast cell degranulation | Mar-97 | 47/18866 | 0.00181 | 0.00489 | 0.00208 | HMOX1/IL4/PIK3CG | 3 |
| GO:0032874 | positive regulation of stress-activated MAPK cascade | May-97 | 170/18866 | 0.00185 | 0.00498 | 0.00212 | CD40LG/IL1B/TNF/VEGFA/XDH | 5 |
| GO:2000241 | regulation of reproductive process | May-97 | 170/18866 | 0.00185 | 0.00498 | 0.00212 | AR/CTNNB1/ESR1/RXRA/VEGFA | 5 |
| GO:2000060 | positive regulation of ubiquitin-dependent protein catabolic process | Apr-97 | 102/18866 | 0.0019 | 0.00511 | 0.00218 | AKT1/CAV1/EGF/PTEN | 4 |
| GO:0071560 | cellular response to transforming growth factor beta stimulus | Jun-97 | 252/18866 | 0.00192 | 0.00515 | 0.0022 | CAV1/FOS/JUN/NR3C1/TGFB1/TP53 | 6 |
| GO:0002279 | mast cell activation involved in immune response | Mar-97 | 48/18866 | 0.00193 | 0.00515 | 0.0022 | HMOX1/IL4/PIK3CG | 3 |
| GO:0009409 | response to cold | Mar-97 | 48/18866 | 0.00193 | 0.00515 | 0.0022 | FOS/NFKBIA/PPARG | 3 |
| GO:0010799 | regulation of peptidyl-threonine phosphorylation | Mar-97 | 48/18866 | 0.00193 | 0.00515 | 0.0022 | EGF/MAPK1/TGFB1 | 3 |
| GO:0033628 | regulation of cell adhesion mediated by integrin | Mar-97 | 48/18866 | 0.00193 | 0.00515 | 0.0022 | DPP4/PIK3CG/SERPINE1 | 3 |
| GO:0043370 | regulation of CD4-positive, alpha-beta T cell differentiation | Mar-97 | 48/18866 | 0.00193 | 0.00515 | 0.0022 | IFNG/IL2/IL4 | 3 |
| GO:0060324 | face development | Mar-97 | 48/18866 | 0.00193 | 0.00515 | 0.0022 | MAPK1/MAPK3/MMP2 | 3 |
| GO:1900274 | regulation of phospholipase C activity | Mar-97 | 48/18866 | 0.00193 | 0.00515 | 0.0022 | EGFR/ESR1/SELE | 3 |
| GO:0006022 | aminoglycan metabolic process | May-97 | 172/18866 | 0.00195 | 0.0052 | 0.00222 | AKT1/EGF/IL1B/PIM1/TGFB1 | 5 |
| GO:0070304 | positive regulation of stress-activated protein kinase signaling cascade | May-97 | 172/18866 | 0.00195 | 0.0052 | 0.00222 | CD40LG/IL1B/TNF/VEGFA/XDH | 5 |
| GO:0002679 | respiratory burst involved in defense response | Feb-97 | 13/18866 | 0.00197 | 0.0052 | 0.00222 | MPO/PIK3CG | 2 |
| GO:0006703 | estrogen biosynthetic process | Feb-97 | 13/18866 | 0.00197 | 0.0052 | 0.00222 | CYP19A1/HSD3B1 | 2 |
| GO:0014842 | regulation of skeletal muscle satellite cell proliferation | Feb-97 | 13/18866 | 0.00197 | 0.0052 | 0.00222 | PPARD/STAT3 | 2 |
| GO:0016264 | gap junction assembly | Feb-97 | 13/18866 | 0.00197 | 0.0052 | 0.00222 | CAV1/IL1B | 2 |
| GO:0031053 | primary miRNA processing | Feb-97 | 13/18866 | 0.00197 | 0.0052 | 0.00222 | IL6/STAT3 | 2 |
| GO:0033089 | positive regulation of T cell differentiation in thymus | Feb-97 | 13/18866 | 0.00197 | 0.0052 | 0.00222 | IL1A/IL1B | 2 |
| GO:0033127 | regulation of histone phosphorylation | Feb-97 | 13/18866 | 0.00197 | 0.0052 | 0.00222 | IL1B/MAPK3 | 2 |
| GO:0043568 | positive regulation of insulin-like growth factor receptor signaling pathway | Feb-97 | 13/18866 | 0.00197 | 0.0052 | 0.00222 | AR/IGFBP3 | 2 |
| GO:0045602 | negative regulation of endothelial cell differentiation | Feb-97 | 13/18866 | 0.00197 | 0.0052 | 0.00222 | VEGFA/XDH | 2 |
| GO:0048302 | regulation of isotype switching to IgG isotypes | Feb-97 | 13/18866 | 0.00197 | 0.0052 | 0.00222 | IL2/IL4 | 2 |
| GO:0061029 | eyelid development in camera-type eye | Feb-97 | 13/18866 | 0.00197 | 0.0052 | 0.00222 | EGFR/JUN | 2 |
| GO:0072683 | T cell extravasation | Feb-97 | 13/18866 | 0.00197 | 0.0052 | 0.00222 | CCL2/ICAM1 | 2 |
| GO:0002042 | cell migration involved in sprouting angiogenesis | Apr-97 | 103/18866 | 0.00197 | 0.0052 | 0.00222 | AKT1/HMOX1/PTGS2/VEGFA | 4 |
| GO:0034766 | negative regulation of ion transmembrane transport | Apr-97 | 103/18866 | 0.00197 | 0.0052 | 0.00222 | AKT1/CAV1/MMP9/PTEN | 4 |
| GO:0006163 | purine nucleotide metabolic process | Aug-97 | 442/18866 | 0.002 | 0.00528 | 0.00225 | IFNG/IL4/NOS2/NOS3/PPARA/STAT3/TGFB1/XDH | 8 |
| GO:0021543 | pallium development | May-97 | 173/18866 | 0.002 | 0.00528 | 0.00225 | CASP3/CTNNB1/EGFR/HSD3B1/PTEN | 5 |
| GO:0002456 | T cell mediated immunity | Apr-97 | 104/18866 | 0.00204 | 0.00535 | 0.00228 | ICAM1/IL1B/IL4/IL6 | 4 |
| GO:0032649 | regulation of interferon-gamma production | Apr-97 | 104/18866 | 0.00204 | 0.00535 | 0.00228 | IL10/IL1B/IL2/TNF | 4 |
| GO:0043279 | response to alkaloid | Apr-97 | 104/18866 | 0.00204 | 0.00535 | 0.00228 | BCL2L1/CASP3/ICAM1/PPARG | 4 |
| GO:0048525 | negative regulation of viral process | Apr-97 | 104/18866 | 0.00204 | 0.00535 | 0.00228 | CRP/JUN/SLPI/TNF | 4 |
| GO:0002448 | mast cell mediated immunity | Mar-97 | 49/18866 | 0.00205 | 0.00535 | 0.00228 | HMOX1/IL4/PIK3CG | 3 |
| GO:0007595 | lactation | Mar-97 | 49/18866 | 0.00205 | 0.00535 | 0.00228 | CAV1/VEGFA/XDH | 3 |
| GO:0045058 | T cell selection | Mar-97 | 49/18866 | 0.00205 | 0.00535 | 0.00228 | BCL2/IL6/STAT3 | 3 |
| GO:0048641 | regulation of skeletal muscle tissue development | Mar-97 | 49/18866 | 0.00205 | 0.00535 | 0.00228 | BCL2/CTNNB1/TGFB1 | 3 |
| GO:0060711 | labyrinthine layer development | Mar-97 | 49/18866 | 0.00205 | 0.00535 | 0.00228 | AKT1/IL10/MAPK1 | 3 |
| GO:0071349 | cellular response to interleukin-12 | Mar-97 | 49/18866 | 0.00205 | 0.00535 | 0.00228 | IFNG/IL10/SOD1 | 3 |
| GO:0090151 | establishment of protein localization to mitochondrial membrane | Mar-97 | 49/18866 | 0.00205 | 0.00535 | 0.00228 | BCL2/MAPK8/TP53 | 3 |
| GO:1900087 | positive regulation of G1/S transition of mitotic cell cycle | Mar-97 | 49/18866 | 0.00205 | 0.00535 | 0.00228 | AKT1/CYP1A1/EGFR | 3 |
| GO:0090257 | regulation of muscle system process | Jun-97 | 256/18866 | 0.00208 | 0.00544 | 0.00232 | CAV1/NOS3/PIK3CG/PPARA/PTGS2/SOD1 | 6 |
| GO:1904062 | regulation of cation transmembrane transport | Jul-97 | 349/18866 | 0.00216 | 0.00561 | 0.00239 | CAV1/CCL2/F2/IFNG/MMP9/PIK3CG/PTEN | 7 |
| GO:0051607 | defense response to virus | Jun-97 | 258/18866 | 0.00216 | 0.00561 | 0.00239 | BCL2/BCL2L1/IFNG/IL1B/IL4/IL6 | 6 |
| GO:0071559 | response to transforming growth factor beta | Jun-97 | 258/18866 | 0.00216 | 0.00561 | 0.00239 | CAV1/FOS/JUN/NR3C1/TGFB1/TP53 | 6 |
| GO:0014009 | glial cell proliferation | Mar-97 | 50/18866 | 0.00217 | 0.00561 | 0.00239 | IL1B/IL6/TNF | 3 |
| GO:0042987 | amyloid precursor protein catabolic process | Mar-97 | 50/18866 | 0.00217 | 0.00561 | 0.00239 | CASP3/IFNG/TNF | 3 |
| GO:0045540 | regulation of cholesterol biosynthetic process | Mar-97 | 50/18866 | 0.00217 | 0.00561 | 0.00239 | APOB/SOD1/SREBF1 | 3 |
| GO:0046850 | regulation of bone remodeling | Mar-97 | 50/18866 | 0.00217 | 0.00561 | 0.00239 | EGFR/IL6/SPP1 | 3 |
| GO:0048546 | digestive tract morphogenesis | Mar-97 | 50/18866 | 0.00217 | 0.00561 | 0.00239 | BCL2/CTNNB1/EGFR | 3 |
| GO:0050873 | brown fat cell differentiation | Mar-97 | 50/18866 | 0.00217 | 0.00561 | 0.00239 | ADIPOQ/PIM1/PTGS2 | 3 |
| GO:0070671 | response to interleukin-12 | Mar-97 | 50/18866 | 0.00217 | 0.00561 | 0.00239 | IFNG/IL10/SOD1 | 3 |
| GO:0097300 | programmed necrotic cell death | Mar-97 | 50/18866 | 0.00217 | 0.00561 | 0.00239 | CAV1/TNF/TP53 | 3 |
| GO:0101023 | vascular endothelial cell proliferation | Mar-97 | 50/18866 | 0.00217 | 0.00561 | 0.00239 | CCL2/PPARG/STAT3 | 3 |
| GO:0106118 | regulation of sterol biosynthetic process | Mar-97 | 50/18866 | 0.00217 | 0.00561 | 0.00239 | APOB/SOD1/SREBF1 | 3 |
| GO:1903580 | positive regulation of ATP metabolic process | Mar-97 | 50/18866 | 0.00217 | 0.00561 | 0.00239 | IFNG/IL4/STAT3 | 3 |
| GO:1905562 | regulation of vascular endothelial cell proliferation | Mar-97 | 50/18866 | 0.00217 | 0.00561 | 0.00239 | CCL2/PPARG/STAT3 | 3 |
| GO:1901990 | regulation of mitotic cell cycle phase transition | Aug-97 | 448/18866 | 0.00217 | 0.00562 | 0.0024 | AKT1/BCL2/CCL2/CDKN1A/CYP1A1/EGFR/PTEN/TP53 | 8 |
| GO:0046942 | carboxylic acid transport | Jul-97 | 350/18866 | 0.00219 | 0.00567 | 0.00242 | AKT1/IL1B/NOS2/PPARA/PPARD/PPARG/RXRA | 7 |
| GO:0000018 | regulation of DNA recombination | Apr-97 | 107/18866 | 0.00226 | 0.00582 | 0.00248 | IL10/IL2/IL4/TGFB1 | 4 |
| GO:0010889 | regulation of sequestering of triglyceride | Feb-97 | 14/18866 | 0.00229 | 0.00582 | 0.00248 | PPARA/PPARG | 2 |
| GO:0014841 | skeletal muscle satellite cell proliferation | Feb-97 | 14/18866 | 0.00229 | 0.00582 | 0.00248 | PPARD/STAT3 | 2 |
| GO:0014854 | response to inactivity | Feb-97 | 14/18866 | 0.00229 | 0.00582 | 0.00248 | CAT/IL10 | 2 |
| GO:0014857 | regulation of skeletal muscle cell proliferation | Feb-97 | 14/18866 | 0.00229 | 0.00582 | 0.00248 | PPARD/STAT3 | 2 |
| GO:0031282 | regulation of guanylate cyclase activity | Feb-97 | 14/18866 | 0.00229 | 0.00582 | 0.00248 | NOS2/NOS3 | 2 |
| GO:0032310 | prostaglandin secretion | Feb-97 | 14/18866 | 0.00229 | 0.00582 | 0.00248 | IL1B/NOS2 | 2 |
| GO:0035112 | genitalia morphogenesis | Feb-97 | 14/18866 | 0.00229 | 0.00582 | 0.00248 | AR/CTNNB1 | 2 |
| GO:0036295 | cellular response to increased oxygen levels | Feb-97 | 14/18866 | 0.00229 | 0.00582 | 0.00248 | CAV1/PPARG | 2 |
| GO:0045591 | positive regulation of regulatory T cell differentiation | Feb-97 | 14/18866 | 0.00229 | 0.00582 | 0.00248 | IFNG/IL2 | 2 |
| GO:0045898 | regulation of RNA polymerase II transcription preinitiation complex assembly | Feb-97 | 14/18866 | 0.00229 | 0.00582 | 0.00248 | ESR1/TP53 | 2 |
| GO:0046321 | positive regulation of fatty acid oxidation | Feb-97 | 14/18866 | 0.00229 | 0.00582 | 0.00248 | PPARA/PPARG | 2 |
| GO:0048291 | isotype switching to IgG isotypes | Feb-97 | 14/18866 | 0.00229 | 0.00582 | 0.00248 | IL2/IL4 | 2 |
| GO:0048681 | negative regulation of axon regeneration | Feb-97 | 14/18866 | 0.00229 | 0.00582 | 0.00248 | PTEN/SPP1 | 2 |
| GO:0050930 | induction of positive chemotaxis | Feb-97 | 14/18866 | 0.00229 | 0.00582 | 0.00248 | CXCL8/VEGFA | 2 |
| GO:0051547 | regulation of keratinocyte migration | Feb-97 | 14/18866 | 0.00229 | 0.00582 | 0.00248 | MMP9/PTEN | 2 |
| GO:0051709 | regulation of killing of cells of other organism | Feb-97 | 14/18866 | 0.00229 | 0.00582 | 0.00248 | IFNG/NOS2 | 2 |
| GO:0072216 | positive regulation of metanephros development | Feb-97 | 14/18866 | 0.00229 | 0.00582 | 0.00248 | ADIPOQ/MYC | 2 |
| GO:1902894 | negative regulation of pri-miRNA transcription by RNA polymerase II | Feb-97 | 14/18866 | 0.00229 | 0.00582 | 0.00248 | PPARA/PPARD | 2 |
| GO:1903054 | negative regulation of extracellular matrix organization | Feb-97 | 14/18866 | 0.00229 | 0.00582 | 0.00248 | DPP4/TGFB1 | 2 |
| GO:2001279 | regulation of unsaturated fatty acid biosynthetic process | Feb-97 | 14/18866 | 0.00229 | 0.00582 | 0.00248 | IL1B/PTGS2 | 2 |
| GO:0032206 | positive regulation of telomere maintenance | Mar-97 | 51/18866 | 0.00229 | 0.00582 | 0.00248 | CTNNB1/MAPK1/MAPK3 | 3 |
| GO:0032873 | negative regulation of stress-activated MAPK cascade | Mar-97 | 51/18866 | 0.00229 | 0.00582 | 0.00248 | AKT1/GSTP1/MYC | 3 |
| GO:0035272 | exocrine system development | Mar-97 | 51/18866 | 0.00229 | 0.00582 | 0.00248 | EGFR/TGFB1/TNF | 3 |
| GO:0038084 | vascular endothelial growth factor signaling pathway | Mar-97 | 51/18866 | 0.00229 | 0.00582 | 0.00248 | HSPB1/VEGFA/XDH | 3 |
| GO:0050435 | amyloid-beta metabolic process | Mar-97 | 51/18866 | 0.00229 | 0.00582 | 0.00248 | CASP3/IFNG/TNF | 3 |
| GO:0070303 | negative regulation of stress-activated protein kinase signaling cascade | Mar-97 | 51/18866 | 0.00229 | 0.00582 | 0.00248 | AKT1/GSTP1/MYC | 3 |
| GO:0071354 | cellular response to interleukin-6 | Mar-97 | 51/18866 | 0.00229 | 0.00582 | 0.00248 | ICAM1/IL6/STAT3 | 3 |
| GO:0071827 | plasma lipoprotein particle organization | Mar-97 | 51/18866 | 0.00229 | 0.00582 | 0.00248 | ALB/APOB/MPO | 3 |
| GO:0015849 | organic acid transport | Jul-97 | 353/18866 | 0.0023 | 0.00583 | 0.00249 | AKT1/IL1B/NOS2/PPARA/PPARD/PPARG/RXRA | 7 |
| GO:0006939 | smooth muscle contraction | Apr-97 | 109/18866 | 0.00242 | 0.00612 | 0.00261 | CAV1/PTGER3/PTGS2/SOD1 | 4 |
| GO:0043618 | regulation of transcription from RNA polymerase II promoter in response to stress | Apr-97 | 109/18866 | 0.00242 | 0.00612 | 0.00261 | HMOX1/JUN/TP53/VEGFA | 4 |
| GO:0038066 | p38MAPK cascade | Mar-97 | 52/18866 | 0.00243 | 0.00612 | 0.00261 | IL1B/VEGFA/XDH | 3 |
| GO:2000677 | regulation of transcription regulatory region DNA binding | Mar-97 | 52/18866 | 0.00243 | 0.00612 | 0.00261 | CTNNB1/IFNG/TGFB1 | 3 |
| GO:2001238 | positive regulation of extrinsic apoptotic signaling pathway | Mar-97 | 52/18866 | 0.00243 | 0.00612 | 0.00261 | CAV1/PTEN/TNF | 3 |
| GO:0051480 | regulation of cytosolic calcium ion concentration | Jul-97 | 357/18866 | 0.00245 | 0.00618 | 0.00264 | BCL2/CAV1/ESR1/F2/IL2/PIK3CG/PTGER3 | 7 |
| GO:0031589 | cell-substrate adhesion | Jul-97 | 359/18866 | 0.00253 | 0.00638 | 0.00272 | BCL2/CTNNB1/PPARD/PTEN/SERPINE1/VCAM1/VEGFA | 7 |
| GO:0032210 | regulation of telomere maintenance via telomerase | Mar-97 | 53/18866 | 0.00256 | 0.00644 | 0.00275 | CTNNB1/MAPK1/MAPK3 | 3 |
| GO:0043124 | negative regulation of I-kappaB kinase/NF-kappaB signaling | Mar-97 | 53/18866 | 0.00256 | 0.00644 | 0.00275 | ADIPOQ/ESR1/GSTP1 | 3 |
| GO:0043392 | negative regulation of DNA binding | Mar-97 | 53/18866 | 0.00256 | 0.00644 | 0.00275 | HMOX1/JUN/NFKBIA | 3 |
| GO:0071320 | cellular response to cAMP | Mar-97 | 53/18866 | 0.00256 | 0.00644 | 0.00275 | ADIPOQ/AHR/PIK3CG | 3 |
| GO:0051149 | positive regulation of muscle cell differentiation | Apr-97 | 111/18866 | 0.00258 | 0.00649 | 0.00277 | BCL2/CTNNB1/IL4/TGFB1 | 4 |
| GO:0009404 | toxin metabolic process | Feb-97 | 15/18866 | 0.00263 | 0.00655 | 0.00279 | CYP1A1/CYP1A2 | 2 |
| GO:0010763 | positive regulation of fibroblast migration | Feb-97 | 15/18866 | 0.00263 | 0.00655 | 0.00279 | AKT1/TGFB1 | 2 |
| GO:0014856 | skeletal muscle cell proliferation | Feb-97 | 15/18866 | 0.00263 | 0.00655 | 0.00279 | PPARD/STAT3 | 2 |
| GO:0035745 | T-helper 2 cell cytokine production | Feb-97 | 15/18866 | 0.00263 | 0.00655 | 0.00279 | IL4/IL6 | 2 |
| GO:0045820 | negative regulation of glycolytic process | Feb-97 | 15/18866 | 0.00263 | 0.00655 | 0.00279 | PPARA/STAT3 | 2 |
| GO:0048308 | organelle inheritance | Feb-97 | 15/18866 | 0.00263 | 0.00655 | 0.00279 | MAPK1/MAPK3 | 2 |
| GO:0048313 | Golgi inheritance | Feb-97 | 15/18866 | 0.00263 | 0.00655 | 0.00279 | MAPK1/MAPK3 | 2 |
| GO:0070571 | negative regulation of neuron projection regeneration | Feb-97 | 15/18866 | 0.00263 | 0.00655 | 0.00279 | PTEN/SPP1 | 2 |
| GO:1900119 | positive regulation of execution phase of apoptosis | Feb-97 | 15/18866 | 0.00263 | 0.00655 | 0.00279 | IL6/TP53 | 2 |
| GO:2000402 | negative regulation of lymphocyte migration | Feb-97 | 15/18866 | 0.00263 | 0.00655 | 0.00279 | AKT1/CCL2 | 2 |
| GO:2001028 | positive regulation of endothelial cell chemotaxis | Feb-97 | 15/18866 | 0.00263 | 0.00655 | 0.00279 | HSPB1/VEGFA | 2 |
| GO:2001171 | positive regulation of ATP biosynthetic process | Feb-97 | 15/18866 | 0.00263 | 0.00655 | 0.00279 | IL4/STAT3 | 2 |
| GO:0006275 | regulation of DNA replication | Apr-97 | 112/18866 | 0.00267 | 0.00665 | 0.00283 | EGF/EGFR/JUN/TP53 | 4 |
| GO:0006754 | ATP biosynthetic process | Mar-97 | 54/18866 | 0.0027 | 0.00672 | 0.00286 | IL4/STAT3/TGFB1 | 3 |
| GO:1901185 | negative regulation of ERBB signaling pathway | Mar-97 | 54/18866 | 0.0027 | 0.00672 | 0.00286 | EGF/EGFR/ERBB2 | 3 |
| GO:2000179 | positive regulation of neural precursor cell proliferation | Mar-97 | 54/18866 | 0.0027 | 0.00672 | 0.00286 | CTNNB1/EGF/VEGFA | 3 |
| GO:0051147 | regulation of muscle cell differentiation | May-97 | 186/18866 | 0.00274 | 0.0068 | 0.0029 | BCL2/CTNNB1/IL4/PPARA/TGFB1 | 5 |
| GO:1903555 | regulation of tumor necrosis factor superfamily cytokine production | Apr-97 | 113/18866 | 0.00276 | 0.00684 | 0.00292 | ADIPOQ/GSTP1/IFNG/IL10 | 4 |
| GO:0006839 | mitochondrial transport | Jun-97 | 271/18866 | 0.00276 | 0.00684 | 0.00292 | BCL2/BCL2L1/MAPK8/SREBF1/STAT3/TP53 | 6 |
| GO:0045333 | cellular respiration | May-97 | 187/18866 | 0.0028 | 0.00694 | 0.00296 | CAT/CYP1A2/IFNG/IL4/NOS2 | 5 |
| GO:0009062 | fatty acid catabolic process | Apr-97 | 114/18866 | 0.00285 | 0.00701 | 0.00299 | ADIPOQ/AKT1/PPARA/PPARD | 4 |
| GO:0001706 | endoderm formation | Mar-97 | 55/18866 | 0.00285 | 0.00701 | 0.00299 | CTNNB1/MMP2/MMP9 | 3 |
| GO:0002931 | response to ischemia | Mar-97 | 55/18866 | 0.00285 | 0.00701 | 0.00299 | BCL2/CAV1/TP53 | 3 |
| GO:0032655 | regulation of interleukin-12 production | Mar-97 | 55/18866 | 0.00285 | 0.00701 | 0.00299 | CD40LG/IFNG/IL10 | 3 |
| GO:0032720 | negative regulation of tumor necrosis factor production | Mar-97 | 55/18866 | 0.00285 | 0.00701 | 0.00299 | ADIPOQ/GSTP1/IL10 | 3 |
| GO:0042304 | regulation of fatty acid biosynthetic process | Mar-97 | 55/18866 | 0.00285 | 0.00701 | 0.00299 | ADIPOQ/IL1B/PTGS2 | 3 |
| GO:0045620 | negative regulation of lymphocyte differentiation | Mar-97 | 55/18866 | 0.00285 | 0.00701 | 0.00299 | ERBB2/IL2/IL4 | 3 |
| GO:0070741 | response to interleukin-6 | Mar-97 | 55/18866 | 0.00285 | 0.00701 | 0.00299 | ICAM1/IL6/STAT3 | 3 |
| GO:0071825 | protein-lipid complex subunit organization | Mar-97 | 55/18866 | 0.00285 | 0.00701 | 0.00299 | ALB/APOB/MPO | 3 |
| GO:0097345 | mitochondrial outer membrane permeabilization | Mar-97 | 55/18866 | 0.00285 | 0.00701 | 0.00299 | BCL2/MAPK8/TP53 | 3 |
| GO:0032609 | interferon-gamma production | Apr-97 | 115/18866 | 0.00294 | 0.00722 | 0.00308 | IL10/IL1B/IL2/TNF | 4 |
| GO:0043620 | regulation of DNA-templated transcription in response to stress | Apr-97 | 115/18866 | 0.00294 | 0.00722 | 0.00308 | HMOX1/JUN/TP53/VEGFA | 4 |
| GO:0048640 | negative regulation of developmental growth | Apr-97 | 115/18866 | 0.00294 | 0.00722 | 0.00308 | CDKN1A/PPARA/PTEN/SPP1 | 4 |
| GO:0072521 | purine-containing compound metabolic process | Aug-97 | 472/18866 | 0.00299 | 0.00728 | 0.0031 | IFNG/IL4/NOS2/NOS3/PPARA/STAT3/TGFB1/XDH | 8 |
| GO:0002070 | epithelial cell maturation | Feb-97 | 16/18866 | 0.003 | 0.00728 | 0.0031 | CDKN1A/PGR | 2 |
| GO:0002295 | T-helper cell lineage commitment | Feb-97 | 16/18866 | 0.003 | 0.00728 | 0.0031 | IL6/STAT3 | 2 |
| GO:0002523 | leukocyte migration involved in inflammatory response | Feb-97 | 16/18866 | 0.003 | 0.00728 | 0.0031 | ALOX5/SELE | 2 |
| GO:0034374 | low-density lipoprotein particle remodeling | Feb-97 | 16/18866 | 0.003 | 0.00728 | 0.0031 | APOB/MPO | 2 |
| GO:0042994 | cytoplasmic sequestering of transcription factor | Feb-97 | 16/18866 | 0.003 | 0.00728 | 0.0031 | IL10/NFKBIA | 2 |
| GO:0051770 | positive regulation of nitric-oxide synthase biosynthetic process | Feb-97 | 16/18866 | 0.003 | 0.00728 | 0.0031 | CCL2/IFNG | 2 |
| GO:0060572 | morphogenesis of an epithelial bud | Feb-97 | 16/18866 | 0.003 | 0.00728 | 0.0031 | AR/CTNNB1 | 2 |
| GO:0070886 | positive regulation of calcineurin-NFAT signaling cascade | Feb-97 | 16/18866 | 0.003 | 0.00728 | 0.0031 | ERBB3/TNF | 2 |
| GO:0071380 | cellular response to prostaglandin E stimulus | Feb-97 | 16/18866 | 0.003 | 0.00728 | 0.0031 | AKT1/PPARG | 2 |
| GO:0071391 | cellular response to estrogen stimulus | Feb-97 | 16/18866 | 0.003 | 0.00728 | 0.0031 | AR/ESR1 | 2 |
| GO:0097091 | synaptic vesicle clustering | Feb-97 | 16/18866 | 0.003 | 0.00728 | 0.0031 | CTNNB1/PTEN | 2 |
| GO:0106058 | positive regulation of calcineurin-mediated signaling | Feb-97 | 16/18866 | 0.003 | 0.00728 | 0.0031 | ERBB3/TNF | 2 |
| GO:1900121 | negative regulation of receptor binding | Feb-97 | 16/18866 | 0.003 | 0.00728 | 0.0031 | ADIPOQ/IL10 | 2 |
| GO:1900451 | positive regulation of glutamate receptor signaling pathway | Feb-97 | 16/18866 | 0.003 | 0.00728 | 0.0031 | CCL2/IFNG | 2 |
| GO:1903358 | regulation of Golgi organization | Feb-97 | 16/18866 | 0.003 | 0.00728 | 0.0031 | MAPK1/MAPK3 | 2 |
| GO:1905050 | positive regulation of metallopeptidase activity | Feb-97 | 16/18866 | 0.003 | 0.00728 | 0.0031 | MAPK3/STAT3 | 2 |
| GO:0046916 | cellular transition metal ion homeostasis | Apr-97 | 116/18866 | 0.00303 | 0.00736 | 0.00314 | HMOX1/IFNG/MYC/SOD1 | 4 |
| GO:0072676 | lymphocyte migration | Apr-97 | 117/18866 | 0.00312 | 0.00758 | 0.00323 | AKT1/CCL2/ICAM1/PIK3CG | 4 |
| GO:0016525 | negative regulation of angiogenesis | May-97 | 192/18866 | 0.00314 | 0.00761 | 0.00324 | ALOX5/CTNNB1/PPARG/SERPINE1/TNF | 5 |
| GO:0002090 | regulation of receptor internalization | Mar-97 | 57/18866 | 0.00315 | 0.00762 | 0.00325 | EGF/SELE/VEGFA | 3 |
| GO:0032615 | interleukin-12 production | Mar-97 | 57/18866 | 0.00315 | 0.00762 | 0.00325 | CD40LG/IFNG/IL10 | 3 |
| GO:0045840 | positive regulation of mitotic nuclear division | Mar-97 | 57/18866 | 0.00315 | 0.00762 | 0.00325 | EGF/IL1A/IL1B | 3 |
| GO:0061005 | cell differentiation involved in kidney development | Mar-97 | 57/18866 | 0.00315 | 0.00762 | 0.00325 | ADIPOQ/CTNNB1/MMP9 | 3 |
| GO:1903556 | negative regulation of tumor necrosis factor superfamily cytokine production | Mar-97 | 57/18866 | 0.00315 | 0.00762 | 0.00325 | ADIPOQ/GSTP1/IL10 | 3 |
| GO:0008544 | epidermis development | Aug-97 | 477/18866 | 0.00319 | 0.00771 | 0.00329 | BCL2/CASP3/CTNNB1/EGFR/PPARA/PPARD/SOD1/TNF | 8 |
| GO:1903749 | positive regulation of establishment of protein localization to mitochondrion | Mar-97 | 58/18866 | 0.00331 | 0.008 | 0.00341 | BCL2/MAPK8/TP53 | 3 |
| GO:0010822 | positive regulation of mitochondrion organization | Apr-97 | 119/18866 | 0.00332 | 0.008 | 0.00341 | BCL2/MAPK8/MMP9/TP53 | 4 |
| GO:0051153 | regulation of striated muscle cell differentiation | Apr-97 | 119/18866 | 0.00332 | 0.008 | 0.00341 | BCL2/IL4/PPARA/TGFB1 | 4 |
| GO:0071706 | tumor necrosis factor superfamily cytokine production | Apr-97 | 119/18866 | 0.00332 | 0.008 | 0.00341 | ADIPOQ/GSTP1/IFNG/IL10 | 4 |
| GO:0006978 | DNA damage response, signal transduction by p53 class mediator resulting in transcription of p21 class mediator | Feb-97 | 17/18866 | 0.00338 | 0.00807 | 0.00344 | CDKN1A/TP53 | 2 |
| GO:0010224 | response to UV-B | Feb-97 | 17/18866 | 0.00338 | 0.00807 | 0.00344 | BCL2/CDKN1A | 2 |
| GO:0015732 | prostaglandin transport | Feb-97 | 17/18866 | 0.00338 | 0.00807 | 0.00344 | IL1B/NOS2 | 2 |
| GO:0030540 | female genitalia development | Feb-97 | 17/18866 | 0.00338 | 0.00807 | 0.00344 | CYP19A1/ESR1 | 2 |
| GO:0030889 | negative regulation of B cell proliferation | Feb-97 | 17/18866 | 0.00338 | 0.00807 | 0.00344 | CASP3/IL10 | 2 |
| GO:0032682 | negative regulation of chemokine production | Feb-97 | 17/18866 | 0.00338 | 0.00807 | 0.00344 | GSTP1/IL10 | 2 |
| GO:0042953 | lipoprotein transport | Feb-97 | 17/18866 | 0.00338 | 0.00807 | 0.00344 | APOB/PPARG | 2 |
| GO:0043117 | positive regulation of vascular permeability | Feb-97 | 17/18866 | 0.00338 | 0.00807 | 0.00344 | TGFB1/VEGFA | 2 |
| GO:0045623 | negative regulation of T-helper cell differentiation | Feb-97 | 17/18866 | 0.00338 | 0.00807 | 0.00344 | IL2/IL4 | 2 |
| GO:0045986 | negative regulation of smooth muscle contraction | Feb-97 | 17/18866 | 0.00338 | 0.00807 | 0.00344 | PTGS2/SOD1 | 2 |
| GO:0071850 | mitotic cell cycle arrest | Feb-97 | 17/18866 | 0.00338 | 0.00807 | 0.00344 | CDKN1A/TP53 | 2 |
| GO:0090336 | positive regulation of brown fat cell differentiation | Feb-97 | 17/18866 | 0.00338 | 0.00807 | 0.00344 | PIM1/PTGS2 | 2 |
| GO:1903209 | positive regulation of oxidative stress-induced cell death | Feb-97 | 17/18866 | 0.00338 | 0.00807 | 0.00344 | MMP3/SOD1 | 2 |
| GO:1904355 | positive regulation of telomere capping | Feb-97 | 17/18866 | 0.00338 | 0.00807 | 0.00344 | MAPK1/MAPK3 | 2 |
| GO:2000136 | regulation of cell proliferation involved in heart morphogenesis | Feb-97 | 17/18866 | 0.00338 | 0.00807 | 0.00344 | CTNNB1/PIM1 | 2 |
| GO:2000811 | negative regulation of anoikis | Feb-97 | 17/18866 | 0.00338 | 0.00807 | 0.00344 | BCL2/CAV1 | 2 |
| GO:0016579 | protein deubiquitination | Jun-97 | 283/18866 | 0.00342 | 0.00814 | 0.00347 | AR/ESR1/MYC/NFKBIA/PTEN/TP53 | 6 |
| GO:0043270 | positive regulation of ion transport | Jun-97 | 284/18866 | 0.00347 | 0.00827 | 0.00352 | AKT1/CAV1/CCL2/F2/IFNG/IL1B | 6 |
| GO:0043030 | regulation of macrophage activation | Mar-97 | 59/18866 | 0.00348 | 0.00827 | 0.00352 | IL10/IL4/IL6 | 3 |
| GO:0046456 | icosanoid biosynthetic process | Mar-97 | 59/18866 | 0.00348 | 0.00827 | 0.00352 | ALOX5/IL1B/PTGS2 | 3 |
| GO:0060043 | regulation of cardiac muscle cell proliferation | Mar-97 | 59/18866 | 0.00348 | 0.00827 | 0.00352 | MAPK1/PIM1/PTEN | 3 |
| GO:0006164 | purine nucleotide biosynthetic process | May-97 | 197/18866 | 0.0035 | 0.00832 | 0.00355 | IL4/NOS2/NOS3/STAT3/TGFB1 | 5 |
| GO:0002065 | columnar/cuboidal epithelial cell differentiation | Apr-97 | 121/18866 | 0.00353 | 0.00837 | 0.00357 | CDKN1A/RXRA/SERPINE1/SOD1 | 4 |
| GO:1901987 | regulation of cell cycle phase transition | Aug-97 | 486/18866 | 0.00357 | 0.00848 | 0.00362 | AKT1/BCL2/CCL2/CDKN1A/CYP1A1/EGFR/PTEN/TP53 | 8 |
| GO:0043312 | neutrophil degranulation | Aug-97 | 487/18866 | 0.00362 | 0.00858 | 0.00366 | ALOX5/CAT/GSTP1/LYZ/MAPK1/MMP9/MPO/SLPI | 8 |
| GO:0042752 | regulation of circadian rhythm | Apr-97 | 122/18866 | 0.00363 | 0.0086 | 0.00367 | MAPK8/PPARA/PPARG/TP53 | 4 |
| GO:0010823 | negative regulation of mitochondrion organization | Mar-97 | 60/18866 | 0.00365 | 0.00863 | 0.00368 | AKT1/BCL2L1/TP53 | 3 |
| GO:1902808 | positive regulation of cell cycle G1/S phase transition | Mar-97 | 60/18866 | 0.00365 | 0.00863 | 0.00368 | AKT1/CYP1A1/EGFR | 3 |
| GO:0060047 | heart contraction | Jun-97 | 287/18866 | 0.00366 | 0.00865 | 0.00369 | CAV1/IL2/NOS3/PIK3CG/SOD1/SREBF1 | 6 |
| GO:0034765 | regulation of ion transmembrane transport | Aug-97 | 489/18866 | 0.00371 | 0.00877 | 0.00374 | AKT1/CAV1/CCL2/F2/IFNG/MMP9/PIK3CG/PTEN | 8 |
| GO:0002283 | neutrophil activation involved in immune response | Aug-97 | 490/18866 | 0.00375 | 0.00887 | 0.00378 | ALOX5/CAT/GSTP1/LYZ/MAPK1/MMP9/MPO/SLPI | 8 |
| GO:0042772 | DNA damage response, signal transduction resulting in transcription | Feb-97 | 18/18866 | 0.00379 | 0.00893 | 0.00381 | CDKN1A/TP53 | 2 |
| GO:0044872 | lipoprotein localization | Feb-97 | 18/18866 | 0.00379 | 0.00893 | 0.00381 | APOB/PPARG | 2 |
| GO:0061323 | cell proliferation involved in heart morphogenesis | Feb-97 | 18/18866 | 0.00379 | 0.00893 | 0.00381 | CTNNB1/PIM1 | 2 |
| GO:0070230 | positive regulation of lymphocyte apoptotic process | Feb-97 | 18/18866 | 0.00379 | 0.00893 | 0.00381 | IL10/TP53 | 2 |
| GO:0071318 | cellular response to ATP | Feb-97 | 18/18866 | 0.00379 | 0.00893 | 0.00381 | PTGS2/SOD1 | 2 |
| GO:2000641 | regulation of early endosome to late endosome transport | Feb-97 | 18/18866 | 0.00379 | 0.00893 | 0.00381 | MAPK1/MAPK3 | 2 |
| GO:0002221 | pattern recognition receptor signaling pathway | May-97 | 201/18866 | 0.00381 | 0.00895 | 0.00382 | APOB/CAV1/ESR1/IKBKB/NFKBIA | 5 |
| GO:0033013 | tetrapyrrole metabolic process | Mar-97 | 61/18866 | 0.00382 | 0.00895 | 0.00382 | CYP1A1/CYP1A2/HMOX1 | 3 |
| GO:0045576 | mast cell activation | Mar-97 | 61/18866 | 0.00382 | 0.00895 | 0.00382 | HMOX1/IL4/PIK3CG | 3 |
| GO:0045843 | negative regulation of striated muscle tissue development | Mar-97 | 61/18866 | 0.00382 | 0.00895 | 0.00382 | PPARA/PTEN/TGFB1 | 3 |
| GO:0046324 | regulation of glucose import | Mar-97 | 61/18866 | 0.00382 | 0.00895 | 0.00382 | ADIPOQ/AKT1/TNF | 3 |
| GO:0046888 | negative regulation of hormone secretion | Mar-97 | 61/18866 | 0.00382 | 0.00895 | 0.00382 | ADIPOQ/IL1B/SREBF1 | 3 |
| GO:1902110 | positive regulation of mitochondrial membrane permeability involved in apoptotic process | Mar-97 | 61/18866 | 0.00382 | 0.00895 | 0.00382 | BCL2/MAPK8/TP53 | 3 |
| GO:1904356 | regulation of telomere maintenance via telomere lengthening | Mar-97 | 61/18866 | 0.00382 | 0.00895 | 0.00382 | CTNNB1/MAPK1/MAPK3 | 3 |
| GO:0016311 | dephosphorylation | Aug-97 | 492/18866 | 0.00385 | 0.009 | 0.00384 | BCL2/IFNG/IGFBP3/IKBKB/PON1/PTEN/TGFB1/TNF | 8 |
| GO:0007179 | transforming growth factor beta receptor signaling pathway | May-97 | 202/18866 | 0.00389 | 0.00911 | 0.00388 | CAV1/FOS/JUN/TGFB1/TP53 | 5 |
| GO:0001952 | regulation of cell-matrix adhesion | Apr-97 | 125/18866 | 0.00396 | 0.00924 | 0.00394 | BCL2/PTEN/SERPINE1/VEGFA | 4 |
| GO:0035270 | endocrine system development | Apr-97 | 125/18866 | 0.00396 | 0.00924 | 0.00394 | AKT1/IL6/MAPK1/MAPK3 | 4 |
| GO:0043500 | muscle adaptation | Apr-97 | 125/18866 | 0.00396 | 0.00924 | 0.00394 | HMOX1/IL1B/NOS3/PPARA | 4 |
| GO:0007405 | neuroblast proliferation | Mar-97 | 62/18866 | 0.004 | 0.00932 | 0.00397 | CTNNB1/TP53/VEGFA | 3 |
| GO:0010518 | positive regulation of phospholipase activity | Mar-97 | 62/18866 | 0.004 | 0.00932 | 0.00397 | EGFR/ESR1/SELE | 3 |
| GO:0048635 | negative regulation of muscle organ development | Mar-97 | 62/18866 | 0.004 | 0.00932 | 0.00397 | PPARA/PTEN/TGFB1 | 3 |
| GO:0055007 | cardiac muscle cell differentiation | Apr-97 | 126/18866 | 0.00407 | 0.00948 | 0.00404 | PPARA/RXRA/TGFB1/VEGFA | 4 |
| GO:0046622 | positive regulation of organ growth | Mar-97 | 63/18866 | 0.00419 | 0.00972 | 0.00414 | AKT1/MAPK1/PIM1 | 3 |
| GO:0070059 | intrinsic apoptotic signaling pathway in response to endoplasmic reticulum stress | Mar-97 | 63/18866 | 0.00419 | 0.00972 | 0.00414 | BCL2/BCL2L1/TP53 | 3 |
| GO:1902686 | mitochondrial outer membrane permeabilization involved in programmed cell death | Mar-97 | 63/18866 | 0.00419 | 0.00972 | 0.00414 | BCL2/MAPK8/TP53 | 3 |
| GO:2000514 | regulation of CD4-positive, alpha-beta T cell activation | Mar-97 | 63/18866 | 0.00419 | 0.00972 | 0.00414 | IFNG/IL2/IL4 | 3 |
| GO:0051053 | negative regulation of DNA metabolic process | Apr-97 | 127/18866 | 0.00419 | 0.00972 | 0.00414 | ADIPOQ/CDKN1A/PPARG/TP53 | 4 |
| GO:1903052 | positive regulation of proteolysis involved in cellular protein catabolic process | Apr-97 | 127/18866 | 0.00419 | 0.00972 | 0.00414 | AKT1/CAV1/EGF/PTEN | 4 |
| GO:0002922 | positive regulation of humoral immune response | Feb-97 | 19/18866 | 0.00423 | 0.00972 | 0.00414 | IL1B/TNF | 2 |
| GO:0010663 | positive regulation of striated muscle cell apoptotic process | Feb-97 | 19/18866 | 0.00423 | 0.00972 | 0.00414 | PTEN/TP53 | 2 |
| GO:0010666 | positive regulation of cardiac muscle cell apoptotic process | Feb-97 | 19/18866 | 0.00423 | 0.00972 | 0.00414 | PTEN/TP53 | 2 |
| GO:0010759 | positive regulation of macrophage chemotaxis | Feb-97 | 19/18866 | 0.00423 | 0.00972 | 0.00414 | MAPK1/MAPK3 | 2 |
| GO:0034393 | positive regulation of smooth muscle cell apoptotic process | Feb-97 | 19/18866 | 0.00423 | 0.00972 | 0.00414 | IFNG/PPARG | 2 |
| GO:0043373 | CD4-positive, alpha-beta T cell lineage commitment | Feb-97 | 19/18866 | 0.00423 | 0.00972 | 0.00414 | IL6/STAT3 | 2 |
| GO:0044320 | cellular response to leptin stimulus | Feb-97 | 19/18866 | 0.00423 | 0.00972 | 0.00414 | PTEN/STAT3 | 2 |
| GO:0045780 | positive regulation of bone resorption | Feb-97 | 19/18866 | 0.00423 | 0.00972 | 0.00414 | EGFR/SPP1 | 2 |
| GO:0046852 | positive regulation of bone remodeling | Feb-97 | 19/18866 | 0.00423 | 0.00972 | 0.00414 | EGFR/SPP1 | 2 |
| GO:0048245 | eosinophil chemotaxis | Feb-97 | 19/18866 | 0.00423 | 0.00972 | 0.00414 | CCL2/IL4 | 2 |
| GO:0070989 | oxidative demethylation | Feb-97 | 19/18866 | 0.00423 | 0.00972 | 0.00414 | CYP1A2/CYP3A4 | 2 |
| GO:1902176 | negative regulation of oxidative stress-induced intrinsic apoptotic signaling pathway | Feb-97 | 19/18866 | 0.00423 | 0.00972 | 0.00414 | AKT1/HSPB1 | 2 |
| GO:2000010 | positive regulation of protein localization to cell surface | Feb-97 | 19/18866 | 0.00423 | 0.00972 | 0.00414 | AKT1/TNF | 2 |
| GO:2000319 | regulation of T-helper 17 cell differentiation | Feb-97 | 19/18866 | 0.00423 | 0.00972 | 0.00414 | IL2/IL4 | 2 |
| GO:0072089 | stem cell proliferation | Apr-97 | 128/18866 | 0.00431 | 0.0099 | 0.00422 | CTNNB1/PIM1/TP53/VEGFA | 4 |
| GO:0003015 | heart process | Jun-97 | 297/18866 | 0.00432 | 0.00992 | 0.00423 | CAV1/IL2/NOS3/PIK3CG/SOD1/SREBF1 | 6 |
| GO:0002437 | inflammatory response to antigenic stimulus | Mar-97 | 64/18866 | 0.00438 | 0.01001 | 0.00427 | ICAM1/IL10/TNF | 3 |
| GO:0042130 | negative regulation of T cell proliferation | Mar-97 | 64/18866 | 0.00438 | 0.01001 | 0.00427 | CASP3/ERBB2/IL10 | 3 |
| GO:0046637 | regulation of alpha-beta T cell differentiation | Mar-97 | 64/18866 | 0.00438 | 0.01001 | 0.00427 | IFNG/IL2/IL4 | 3 |
| GO:0051898 | negative regulation of protein kinase B signaling | Mar-97 | 64/18866 | 0.00438 | 0.01001 | 0.00427 | AKT1/PTEN/XDH | 3 |
| GO:0070265 | necrotic cell death | Mar-97 | 64/18866 | 0.00438 | 0.01001 | 0.00427 | CAV1/TNF/TP53 | 3 |
| GO:1901862 | negative regulation of muscle tissue development | Mar-97 | 64/18866 | 0.00438 | 0.01001 | 0.00427 | PPARA/PTEN/TGFB1 | 3 |
| GO:0072522 | purine-containing compound biosynthetic process | May-97 | 208/18866 | 0.00441 | 0.01008 | 0.0043 | IL4/NOS2/NOS3/STAT3/TGFB1 | 5 |
| GO:0034101 | erythrocyte homeostasis | Apr-97 | 129/18866 | 0.00443 | 0.01012 | 0.00432 | CASP3/HMOX1/STAT3/VEGFA | 4 |
| GO:0035303 | regulation of dephosphorylation | May-97 | 209/18866 | 0.0045 | 0.01027 | 0.00438 | IFNG/IGFBP3/IKBKB/TGFB1/TNF | 5 |
| GO:0070646 | protein modification by small protein removal | Jun-97 | 300/18866 | 0.00454 | 0.01035 | 0.00441 | AR/ESR1/MYC/NFKBIA/PTEN/TP53 | 6 |
| GO:0006940 | regulation of smooth muscle contraction | Mar-97 | 65/18866 | 0.00457 | 0.0104 | 0.00443 | CAV1/PTGS2/SOD1 | 3 |
| GO:0009206 | purine ribonucleoside triphosphate biosynthetic process | Mar-97 | 65/18866 | 0.00457 | 0.0104 | 0.00443 | IL4/STAT3/TGFB1 | 3 |
| GO:0032729 | positive regulation of interferon-gamma production | Mar-97 | 65/18866 | 0.00457 | 0.0104 | 0.00443 | IL1B/IL2/TNF | 3 |
| GO:0035794 | positive regulation of mitochondrial membrane permeability | Mar-97 | 65/18866 | 0.00457 | 0.0104 | 0.00443 | BCL2/MAPK8/TP53 | 3 |
| GO:0090181 | regulation of cholesterol metabolic process | Mar-97 | 65/18866 | 0.00457 | 0.0104 | 0.00443 | APOB/SOD1/SREBF1 | 3 |
| GO:0034764 | positive regulation of transmembrane transport | May-97 | 210/18866 | 0.00459 | 0.01043 | 0.00445 | ADIPOQ/AKT1/CCL2/F2/IFNG | 5 |
| GO:0003206 | cardiac chamber morphogenesis | Apr-97 | 131/18866 | 0.00468 | 0.01057 | 0.00451 | NOS3/RXRA/TGFB1/TP53 | 4 |
| GO:0010508 | positive regulation of autophagy | Apr-97 | 131/18866 | 0.00468 | 0.01057 | 0.00451 | HMOX1/IFNG/IL4/MAPK3 | 4 |
| GO:0046887 | positive regulation of hormone secretion | Apr-97 | 131/18866 | 0.00468 | 0.01057 | 0.00451 | CYP19A1/EGFR/PPARD/SPP1 | 4 |
| GO:0002726 | positive regulation of T cell cytokine production | Feb-97 | 20/18866 | 0.00468 | 0.01057 | 0.00451 | IL1B/IL6 | 2 |
| GO:0006525 | arginine metabolic process | Feb-97 | 20/18866 | 0.00468 | 0.01057 | 0.00451 | NOS2/NOS3 | 2 |
| GO:0019373 | epoxygenase P450 pathway | Feb-97 | 20/18866 | 0.00468 | 0.01057 | 0.00451 | CYP1A1/CYP1A2 | 2 |
| GO:0031998 | regulation of fatty acid beta-oxidation | Feb-97 | 20/18866 | 0.00468 | 0.01057 | 0.00451 | AKT1/PPARA | 2 |
| GO:0045019 | negative regulation of nitric oxide biosynthetic process | Feb-97 | 20/18866 | 0.00468 | 0.01057 | 0.00451 | CAV1/IL10 | 2 |
| GO:0097709 | connective tissue replacement | Feb-97 | 20/18866 | 0.00468 | 0.01057 | 0.00451 | IL1A/TGFB1 | 2 |
| GO:1904406 | negative regulation of nitric oxide metabolic process | Feb-97 | 20/18866 | 0.00468 | 0.01057 | 0.00451 | CAV1/IL10 | 2 |
| GO:2001169 | regulation of ATP biosynthetic process | Feb-97 | 20/18866 | 0.00468 | 0.01057 | 0.00451 | IL4/STAT3 | 2 |
| GO:0009145 | purine nucleoside triphosphate biosynthetic process | Mar-97 | 66/18866 | 0.00477 | 0.01076 | 0.00459 | IL4/STAT3/TGFB1 | 3 |
| GO:1902108 | regulation of mitochondrial membrane permeability involved in apoptotic process | Mar-97 | 66/18866 | 0.00477 | 0.01076 | 0.00459 | BCL2/MAPK8/TP53 | 3 |
| GO:1905207 | regulation of cardiocyte differentiation | Mar-97 | 66/18866 | 0.00477 | 0.01076 | 0.00459 | EGFR/PPARA/TGFB1 | 3 |
| GO:0016051 | carbohydrate biosynthetic process | May-97 | 213/18866 | 0.00487 | 0.01097 | 0.00468 | ADIPOQ/AKT1/EGF/PPARA/TGFB1 | 5 |
| GO:0002548 | monocyte chemotaxis | Mar-97 | 67/18866 | 0.00498 | 0.01119 | 0.00477 | CCL2/IL6/SERPINE1 | 3 |
| GO:0072678 | T cell migration | Mar-97 | 67/18866 | 0.00498 | 0.01119 | 0.00477 | CCL2/ICAM1/PIK3CG | 3 |
| GO:1905710 | positive regulation of membrane permeability | Mar-97 | 67/18866 | 0.00498 | 0.01119 | 0.00477 | BCL2/MAPK8/TP53 | 3 |
| GO:0045995 | regulation of embryonic development | Apr-97 | 134/18866 | 0.00507 | 0.01138 | 0.00485 | ADIPOQ/AR/CTNNB1/IL10 | 4 |
| GO:0046718 | viral entry into host cell | Apr-97 | 134/18866 | 0.00507 | 0.01138 | 0.00485 | CAV1/DPP4/EGFR/ICAM1 | 4 |
| GO:0002693 | positive regulation of cellular extravasation | Feb-97 | 21/18866 | 0.00516 | 0.01145 | 0.00488 | ICAM1/SELE | 2 |
| GO:0010310 | regulation of hydrogen peroxide metabolic process | Feb-97 | 21/18866 | 0.00516 | 0.01145 | 0.00488 | MMP3/STAT3 | 2 |
| GO:0010829 | negative regulation of glucose transmembrane transport | Feb-97 | 21/18866 | 0.00516 | 0.01145 | 0.00488 | IL1B/TNF | 2 |
| GO:0030878 | thyroid gland development | Feb-97 | 21/18866 | 0.00516 | 0.01145 | 0.00488 | MAPK1/MAPK3 | 2 |
| GO:0035357 | peroxisome proliferator activated receptor signaling pathway | Feb-97 | 21/18866 | 0.00516 | 0.01145 | 0.00488 | PPARG/RXRA | 2 |
| GO:0036499 | PERK-mediated unfolded protein response | Feb-97 | 21/18866 | 0.00516 | 0.01145 | 0.00488 | CCL2/CXCL8 | 2 |
| GO:0043371 | negative regulation of CD4-positive, alpha-beta T cell differentiation | Feb-97 | 21/18866 | 0.00516 | 0.01145 | 0.00488 | IL2/IL4 | 2 |
| GO:0043651 | linoleic acid metabolic process | Feb-97 | 21/18866 | 0.00516 | 0.01145 | 0.00488 | ALOX5/GSTP1 | 2 |
| GO:0046827 | positive regulation of protein export from nucleus | Feb-97 | 21/18866 | 0.00516 | 0.01145 | 0.00488 | IL1B/TP53 | 2 |
| GO:0051797 | regulation of hair follicle development | Feb-97 | 21/18866 | 0.00516 | 0.01145 | 0.00488 | CTNNB1/TNF | 2 |
| GO:0060065 | uterus development | Feb-97 | 21/18866 | 0.00516 | 0.01145 | 0.00488 | CYP19A1/ESR1 | 2 |
| GO:0060716 | labyrinthine layer blood vessel development | Feb-97 | 21/18866 | 0.00516 | 0.01145 | 0.00488 | AKT1/MAPK1 | 2 |
| GO:0090201 | negative regulation of release of cytochrome c from mitochondria | Feb-97 | 21/18866 | 0.00516 | 0.01145 | 0.00488 | AKT1/BCL2L1 | 2 |
| GO:0140467 | integrated stress response signaling | Feb-97 | 21/18866 | 0.00516 | 0.01145 | 0.00488 | CCL2/CXCL8 | 2 |
| GO:1900543 | negative regulation of purine nucleotide metabolic process | Feb-97 | 21/18866 | 0.00516 | 0.01145 | 0.00488 | PPARA/STAT3 | 2 |
| GO:1903055 | positive regulation of extracellular matrix organization | Feb-97 | 21/18866 | 0.00516 | 0.01145 | 0.00488 | IL6/TGFB1 | 2 |
| GO:1903204 | negative regulation of oxidative stress-induced neuron death | Feb-97 | 21/18866 | 0.00516 | 0.01145 | 0.00488 | CTNNB1/IL10 | 2 |
| GO:1904886 | beta-catenin destruction complex disassembly | Feb-97 | 21/18866 | 0.00516 | 0.01145 | 0.00488 | CAV1/CTNNB1 | 2 |
| GO:2000269 | regulation of fibroblast apoptotic process | Feb-97 | 21/18866 | 0.00516 | 0.01145 | 0.00488 | PIK3CG/TP53 | 2 |
| GO:0016239 | positive regulation of macroautophagy | Mar-97 | 68/18866 | 0.00519 | 0.01149 | 0.0049 | HMOX1/IL4/MAPK3 | 3 |
| GO:0034381 | plasma lipoprotein particle clearance | Mar-97 | 68/18866 | 0.00519 | 0.01149 | 0.0049 | ADIPOQ/APOB/HMOX1 | 3 |
| GO:0040014 | regulation of multicellular organism growth | Mar-97 | 68/18866 | 0.00519 | 0.01149 | 0.0049 | BCL2/SOD1/STAT3 | 3 |
| GO:0071479 | cellular response to ionizing radiation | Mar-97 | 68/18866 | 0.00519 | 0.01149 | 0.0049 | BCL2L1/CDKN1A/TP53 | 3 |
| GO:0090287 | regulation of cellular response to growth factor stimulus | Jun-97 | 310/18866 | 0.00531 | 0.01176 | 0.00501 | CAV1/CTNNB1/IL1B/TGFB1/TP53/XDH | 6 |
| GO:0046034 | ATP metabolic process | Jun-97 | 311/18866 | 0.00539 | 0.01193 | 0.00509 | IFNG/IL4/PPARA/STAT3/TGFB1/TP53 | 6 |
| GO:0007004 | telomere maintenance via telomerase | Mar-97 | 69/18866 | 0.0054 | 0.01195 | 0.00509 | CTNNB1/MAPK1/MAPK3 | 3 |
| GO:0019730 | antimicrobial humoral response | Apr-97 | 137/18866 | 0.00548 | 0.01211 | 0.00516 | CXCL8/F2/LYZ/SLPI | 4 |
| GO:0055076 | transition metal ion homeostasis | Apr-97 | 138/18866 | 0.00562 | 0.0124 | 0.00529 | HMOX1/IFNG/MYC/SOD1 | 4 |
| GO:0072329 | monocarboxylic acid catabolic process | Apr-97 | 138/18866 | 0.00562 | 0.0124 | 0.00529 | ADIPOQ/AKT1/PPARA/PPARD | 4 |
| GO:0045655 | regulation of monocyte differentiation | Feb-97 | 22/18866 | 0.00565 | 0.01243 | 0.0053 | JUN/MYC | 2 |
| GO:0045980 | negative regulation of nucleotide metabolic process | Feb-97 | 22/18866 | 0.00565 | 0.01243 | 0.0053 | PPARA/STAT3 | 2 |
| GO:0050765 | negative regulation of phagocytosis | Feb-97 | 22/18866 | 0.00565 | 0.01243 | 0.0053 | ADIPOQ/PTEN | 2 |
| GO:0051000 | positive regulation of nitric-oxide synthase activity | Feb-97 | 22/18866 | 0.00565 | 0.01243 | 0.0053 | AKT1/TNF | 2 |
| GO:1901685 | glutathione derivative metabolic process | Feb-97 | 22/18866 | 0.00565 | 0.01243 | 0.0053 | GSTM1/GSTP1 | 2 |
| GO:1901687 | glutathione derivative biosynthetic process | Feb-97 | 22/18866 | 0.00565 | 0.01243 | 0.0053 | GSTM1/GSTP1 | 2 |
| GO:2000316 | regulation of T-helper 17 type immune response | Feb-97 | 22/18866 | 0.00565 | 0.01243 | 0.0053 | IL2/IL4 | 2 |
| GO:0035304 | regulation of protein dephosphorylation | Apr-97 | 139/18866 | 0.00576 | 0.01266 | 0.0054 | IGFBP3/IKBKB/TGFB1/TNF | 4 |
| GO:0009201 | ribonucleoside triphosphate biosynthetic process | Mar-97 | 71/18866 | 0.00585 | 0.01284 | 0.00547 | IL4/STAT3/TGFB1 | 3 |
| GO:0051785 | positive regulation of nuclear division | Mar-97 | 71/18866 | 0.00585 | 0.01284 | 0.00547 | EGF/IL1A/IL1B | 3 |
| GO:0016485 | protein processing | May-97 | 223/18866 | 0.0059 | 0.01294 | 0.00552 | CASP3/F3/F7/PLAT/SERPINE1 | 5 |
| GO:0042440 | pigment metabolic process | Mar-97 | 72/18866 | 0.00608 | 0.01332 | 0.00568 | BCL2/HMOX1/TYR | 3 |
| GO:0070988 | demethylation | Mar-97 | 72/18866 | 0.00608 | 0.01332 | 0.00568 | CYP1A1/CYP1A2/CYP3A4 | 3 |
| GO:0002052 | positive regulation of neuroblast proliferation | Feb-97 | 23/18866 | 0.00617 | 0.0134 | 0.00571 | CTNNB1/VEGFA | 2 |
| GO:0006582 | melanin metabolic process | Feb-97 | 23/18866 | 0.00617 | 0.0134 | 0.00571 | BCL2/TYR | 2 |
| GO:0010288 | response to lead ion | Feb-97 | 23/18866 | 0.00617 | 0.0134 | 0.00571 | CAT/PTGS2 | 2 |
| GO:0030539 | male genitalia development | Feb-97 | 23/18866 | 0.00617 | 0.0134 | 0.00571 | AR/CTNNB1 | 2 |
| GO:0031639 | plasminogen activation | Feb-97 | 23/18866 | 0.00617 | 0.0134 | 0.00571 | PLAT/SERPINE1 | 2 |
| GO:0032799 | low-density lipoprotein receptor particle metabolic process | Feb-97 | 23/18866 | 0.00617 | 0.0134 | 0.00571 | ADIPOQ/PPARG | 2 |
| GO:0043586 | tongue development | Feb-97 | 23/18866 | 0.00617 | 0.0134 | 0.00571 | CTNNB1/EGFR | 2 |
| GO:0045649 | regulation of macrophage differentiation | Feb-97 | 23/18866 | 0.00617 | 0.0134 | 0.00571 | ADIPOQ/TGFB1 | 2 |
| GO:0045723 | positive regulation of fatty acid biosynthetic process | Feb-97 | 23/18866 | 0.00617 | 0.0134 | 0.00571 | IL1B/PTGS2 | 2 |
| GO:0051220 | cytoplasmic sequestering of protein | Feb-97 | 23/18866 | 0.00617 | 0.0134 | 0.00571 | IL10/NFKBIA | 2 |
| GO:0072677 | eosinophil migration | Feb-97 | 23/18866 | 0.00617 | 0.0134 | 0.00571 | CCL2/IL4 | 2 |
| GO:1901522 | positive regulation of transcription from RNA polymerase II promoter involved in cellular response to chemical stimulus | Feb-97 | 23/18866 | 0.00617 | 0.0134 | 0.00571 | TP53/VEGFA | 2 |
| GO:1903077 | negative regulation of protein localization to plasma membrane | Feb-97 | 23/18866 | 0.00617 | 0.0134 | 0.00571 | BCL2L1/TGFB1 | 2 |
| GO:1903589 | positive regulation of blood vessel endothelial cell proliferation involved in sprouting angiogenesis | Feb-97 | 23/18866 | 0.00617 | 0.0134 | 0.00571 | HMOX1/VEGFA | 2 |
| GO:2000810 | regulation of bicellular tight junction assembly | Feb-97 | 23/18866 | 0.00617 | 0.0134 | 0.00571 | IKBKB/TNF | 2 |
| GO:2001020 | regulation of response to DNA damage stimulus | May-97 | 226/18866 | 0.00623 | 0.01353 | 0.00577 | BCL2/BCL2L1/EGFR/MYC/TP53 | 5 |
| GO:0010517 | regulation of phospholipase activity | Mar-97 | 73/18866 | 0.00632 | 0.0137 | 0.00584 | EGFR/ESR1/SELE | 3 |
| GO:0051155 | positive regulation of striated muscle cell differentiation | Mar-97 | 73/18866 | 0.00632 | 0.0137 | 0.00584 | BCL2/IL4/TGFB1 | 3 |
| GO:0006470 | protein dephosphorylation | Jun-97 | 323/18866 | 0.00646 | 0.01399 | 0.00597 | BCL2/IGFBP3/IKBKB/PTEN/TGFB1/TNF | 6 |
| GO:0001759 | organ induction | Feb-97 | 24/18866 | 0.00671 | 0.01445 | 0.00616 | AR/CTNNB1 | 2 |
| GO:0010804 | negative regulation of tumor necrosis factor-mediated signaling pathway | Feb-97 | 24/18866 | 0.00671 | 0.01445 | 0.00616 | ADIPOQ/GSTP1 | 2 |
| GO:0034695 | response to prostaglandin E | Feb-97 | 24/18866 | 0.00671 | 0.01445 | 0.00616 | AKT1/PPARG | 2 |
| GO:0036003 | positive regulation of transcription from RNA polymerase II promoter in response to stress | Feb-97 | 24/18866 | 0.00671 | 0.01445 | 0.00616 | TP53/VEGFA | 2 |
| GO:0042738 | exogenous drug catabolic process | Feb-97 | 24/18866 | 0.00671 | 0.01445 | 0.00616 | CYP1A2/CYP3A4 | 2 |
| GO:0044321 | response to leptin | Feb-97 | 24/18866 | 0.00671 | 0.01445 | 0.00616 | PTEN/STAT3 | 2 |
| GO:0044550 | secondary metabolite biosynthetic process | Feb-97 | 24/18866 | 0.00671 | 0.01445 | 0.00616 | CYP1A2/TYR | 2 |
| GO:0051349 | positive regulation of lyase activity | Feb-97 | 24/18866 | 0.00671 | 0.01445 | 0.00616 | NOS2/NOS3 | 2 |
| GO:0090335 | regulation of brown fat cell differentiation | Feb-97 | 24/18866 | 0.00671 | 0.01445 | 0.00616 | PIM1/PTGS2 | 2 |
| GO:2000209 | regulation of anoikis | Feb-97 | 24/18866 | 0.00671 | 0.01445 | 0.00616 | BCL2/CAV1 | 2 |
| GO:0006695 | cholesterol biosynthetic process | Mar-97 | 75/18866 | 0.00681 | 0.01463 | 0.00624 | APOB/SOD1/SREBF1 | 3 |
| GO:0030858 | positive regulation of epithelial cell differentiation | Mar-97 | 75/18866 | 0.00681 | 0.01463 | 0.00624 | ADIPOQ/CTNNB1/SERPINE1 | 3 |
| GO:0032418 | lysosome localization | Mar-97 | 75/18866 | 0.00681 | 0.01463 | 0.00624 | HMOX1/IL4/PIK3CG | 3 |
| GO:0046323 | glucose import | Mar-97 | 75/18866 | 0.00681 | 0.01463 | 0.00624 | ADIPOQ/AKT1/TNF | 3 |
| GO:1902653 | secondary alcohol biosynthetic process | Mar-97 | 75/18866 | 0.00681 | 0.01463 | 0.00624 | APOB/SOD1/SREBF1 | 3 |
| GO:0051592 | response to calcium ion | Apr-97 | 147/18866 | 0.007 | 0.01504 | 0.00641 | CAV1/EGFR/FOS/JUN | 4 |
| GO:0006278 | RNA-dependent DNA biosynthetic process | Mar-97 | 76/18866 | 0.00706 | 0.01514 | 0.00645 | CTNNB1/MAPK1/MAPK3 | 3 |
| GO:0033555 | multicellular organismal response to stress | Mar-97 | 76/18866 | 0.00706 | 0.01514 | 0.00645 | BCL2/DPP4/PTEN | 3 |
| GO:0060193 | positive regulation of lipase activity | Mar-97 | 76/18866 | 0.00706 | 0.01514 | 0.00645 | EGFR/ESR1/SELE | 3 |
| GO:0046639 | negative regulation of alpha-beta T cell differentiation | Feb-97 | 25/18866 | 0.00727 | 0.01553 | 0.00662 | IL2/IL4 | 2 |
| GO:0060330 | regulation of response to interferon-gamma | Feb-97 | 25/18866 | 0.00727 | 0.01553 | 0.00662 | IFNG/PPARG | 2 |
| GO:0060334 | regulation of interferon-gamma-mediated signaling pathway | Feb-97 | 25/18866 | 0.00727 | 0.01553 | 0.00662 | IFNG/PPARG | 2 |
| GO:1904376 | negative regulation of protein localization to cell periphery | Feb-97 | 25/18866 | 0.00727 | 0.01553 | 0.00662 | BCL2L1/TGFB1 | 2 |
| GO:2001026 | regulation of endothelial cell chemotaxis | Feb-97 | 25/18866 | 0.00727 | 0.01553 | 0.00662 | HSPB1/VEGFA | 2 |
| GO:0032413 | negative regulation of ion transmembrane transporter activity | Mar-97 | 77/18866 | 0.00732 | 0.01564 | 0.00667 | CAV1/MMP9/PTEN | 3 |
| GO:1903364 | positive regulation of cellular protein catabolic process | Apr-97 | 149/18866 | 0.00734 | 0.01567 | 0.00668 | AKT1/CAV1/EGF/PTEN | 4 |
| GO:0035148 | tube formation | Apr-97 | 150/18866 | 0.00751 | 0.01602 | 0.00683 | CASP3/CTNNB1/TGFB1/VEGFA | 4 |
| GO:0090263 | positive regulation of canonical Wnt signaling pathway | Apr-97 | 150/18866 | 0.00751 | 0.01602 | 0.00683 | CAV1/EGF/EGFR/TGFB1 | 4 |
| GO:0000271 | polysaccharide biosynthetic process | Mar-97 | 78/18866 | 0.00759 | 0.01615 | 0.00689 | AKT1/EGF/TGFB1 | 3 |
| GO:0060395 | SMAD protein signal transduction | Mar-97 | 78/18866 | 0.00759 | 0.01615 | 0.00689 | FOS/JUN/TGFB1 | 3 |
| GO:0072332 | intrinsic apoptotic signaling pathway by p53 class mediator | Mar-97 | 78/18866 | 0.00759 | 0.01615 | 0.00689 | BCL2/CDKN1A/TP53 | 3 |
| GO:0016331 | morphogenesis of embryonic epithelium | Apr-97 | 151/18866 | 0.00769 | 0.01635 | 0.00697 | AR/CASP3/CTNNB1/TGFB1 | 4 |
| GO:0002227 | innate immune response in mucosa | Feb-97 | 26/18866 | 0.00785 | 0.01655 | 0.00706 | IL4/NOS2 | 2 |
| GO:0006309 | apoptotic DNA fragmentation | Feb-97 | 26/18866 | 0.00785 | 0.01655 | 0.00706 | CASP3/IL6 | 2 |
| GO:0032743 | positive regulation of interleukin-2 production | Feb-97 | 26/18866 | 0.00785 | 0.01655 | 0.00706 | IL1A/IL1B | 2 |
| GO:0032967 | positive regulation of collagen biosynthetic process | Feb-97 | 26/18866 | 0.00785 | 0.01655 | 0.00706 | F2/TGFB1 | 2 |
| GO:0042737 | drug catabolic process | Feb-97 | 26/18866 | 0.00785 | 0.01655 | 0.00706 | CYP1A2/CYP3A4 | 2 |
| GO:0043567 | regulation of insulin-like growth factor receptor signaling pathway | Feb-97 | 26/18866 | 0.00785 | 0.01655 | 0.00706 | AR/IGFBP3 | 2 |
| GO:0048643 | positive regulation of skeletal muscle tissue development | Feb-97 | 26/18866 | 0.00785 | 0.01655 | 0.00706 | BCL2/CTNNB1 | 2 |
| GO:0051123 | RNA polymerase II preinitiation complex assembly | Feb-97 | 26/18866 | 0.00785 | 0.01655 | 0.00706 | ESR1/TP53 | 2 |
| GO:0072202 | cell differentiation involved in metanephros development | Feb-97 | 26/18866 | 0.00785 | 0.01655 | 0.00706 | ADIPOQ/CTNNB1 | 2 |
| GO:1903649 | regulation of cytoplasmic transport | Feb-97 | 26/18866 | 0.00785 | 0.01655 | 0.00706 | MAPK1/MAPK3 | 2 |
| GO:1904353 | regulation of telomere capping | Feb-97 | 26/18866 | 0.00785 | 0.01655 | 0.00706 | MAPK1/MAPK3 | 2 |
| GO:1905523 | positive regulation of macrophage migration | Feb-97 | 26/18866 | 0.00785 | 0.01655 | 0.00706 | MAPK1/MAPK3 | 2 |
| GO:0006446 | regulation of translational initiation | Mar-97 | 79/18866 | 0.00786 | 0.01655 | 0.00706 | HSPB1/RXRA/TNF | 3 |
| GO:0007492 | endoderm development | Mar-97 | 79/18866 | 0.00786 | 0.01655 | 0.00706 | CTNNB1/MMP2/MMP9 | 3 |
| GO:0009205 | purine ribonucleoside triphosphate metabolic process | Mar-97 | 79/18866 | 0.00786 | 0.01655 | 0.00706 | IL4/STAT3/TGFB1 | 3 |
| GO:0031016 | pancreas development | Mar-97 | 79/18866 | 0.00786 | 0.01655 | 0.00706 | AKT1/CTNNB1/IL6 | 3 |
| GO:0070373 | negative regulation of ERK1 and ERK2 cascade | Mar-97 | 79/18866 | 0.00786 | 0.01655 | 0.00706 | ADIPOQ/GSTP1/PTEN | 3 |
| GO:0007605 | sensory perception of sound | Apr-97 | 152/18866 | 0.00786 | 0.01656 | 0.00706 | BIRC5/CASP3/ICAM1/SOD1 | 4 |
| GO:0042391 | regulation of membrane potential | Jul-97 | 443/18866 | 0.00788 | 0.01658 | 0.00707 | AKT1/BCL2/BCL2L1/CAV1/JUN/PTEN/SOD1 | 7 |
| GO:0060070 | canonical Wnt signaling pathway | Jun-97 | 339/18866 | 0.0081 | 0.01704 | 0.00727 | CAV1/CTNNB1/EGF/EGFR/PTEN/TGFB1 | 6 |
| GO:0003151 | outflow tract morphogenesis | Mar-97 | 80/18866 | 0.00813 | 0.01709 | 0.00729 | CTNNB1/JUN/VEGFA | 3 |
| GO:0006110 | regulation of glycolytic process | Mar-97 | 80/18866 | 0.00813 | 0.01709 | 0.00729 | IFNG/PPARA/STAT3 | 3 |
| GO:0061351 | neural precursor cell proliferation | Apr-97 | 154/18866 | 0.00823 | 0.01727 | 0.00736 | CTNNB1/EGF/TP53/VEGFA | 4 |
| GO:0010833 | telomere maintenance via telomere lengthening | Mar-97 | 81/18866 | 0.00841 | 0.01763 | 0.00752 | CTNNB1/MAPK1/MAPK3 | 3 |
| GO:0016126 | sterol biosynthetic process | Mar-97 | 81/18866 | 0.00841 | 0.01763 | 0.00752 | APOB/SOD1/SREBF1 | 3 |
| GO:0021766 | hippocampus development | Mar-97 | 81/18866 | 0.00841 | 0.01763 | 0.00752 | CASP3/HSD3B1/PTEN | 3 |
| GO:0002026 | regulation of the force of heart contraction | Feb-97 | 27/18866 | 0.00845 | 0.01763 | 0.00752 | CAV1/NOS3 | 2 |
| GO:0009065 | glutamine family amino acid catabolic process | Feb-97 | 27/18866 | 0.00845 | 0.01763 | 0.00752 | NOS2/NOS3 | 2 |
| GO:0009651 | response to salt stress | Feb-97 | 27/18866 | 0.00845 | 0.01763 | 0.00752 | TNF/TP53 | 2 |
| GO:0010714 | positive regulation of collagen metabolic process | Feb-97 | 27/18866 | 0.00845 | 0.01763 | 0.00752 | F2/TGFB1 | 2 |
| GO:0050996 | positive regulation of lipid catabolic process | Feb-97 | 27/18866 | 0.00845 | 0.01763 | 0.00752 | IL1B/PPARA | 2 |
| GO:1903203 | regulation of oxidative stress-induced neuron death | Feb-97 | 27/18866 | 0.00845 | 0.01763 | 0.00752 | CTNNB1/IL10 | 2 |
| GO:1905208 | negative regulation of cardiocyte differentiation | Feb-97 | 27/18866 | 0.00845 | 0.01763 | 0.00752 | EGFR/PPARA | 2 |
| GO:2000353 | positive regulation of endothelial cell apoptotic process | Feb-97 | 27/18866 | 0.00845 | 0.01763 | 0.00752 | CCL2/CD40LG | 2 |
| GO:0008360 | regulation of cell shape | Apr-97 | 156/18866 | 0.0086 | 0.01791 | 0.00764 | CCL2/F2/ICAM1/VEGFA | 4 |
| GO:0030902 | hindbrain development | Apr-97 | 156/18866 | 0.0086 | 0.01791 | 0.00764 | BCL2/CTNNB1/EGF/TP53 | 4 |
| GO:0048592 | eye morphogenesis | Apr-97 | 156/18866 | 0.0086 | 0.01791 | 0.00764 | BCL2/CTNNB1/STAT3/VEGFA | 4 |
| GO:0009142 | nucleoside triphosphate biosynthetic process | Mar-97 | 82/18866 | 0.0087 | 0.01812 | 0.00773 | IL4/STAT3/TGFB1 | 3 |
| GO:1903900 | regulation of viral life cycle | Apr-97 | 157/18866 | 0.00879 | 0.01829 | 0.0078 | BCL2/CXCL8/SLPI/TNF | 4 |
| GO:0048041 | focal adhesion assembly | Mar-97 | 83/18866 | 0.009 | 0.0187 | 0.00797 | BCL2/PTEN/VEGFA | 3 |
| GO:2000243 | positive regulation of reproductive process | Mar-97 | 83/18866 | 0.009 | 0.0187 | 0.00797 | AR/CTNNB1/VEGFA | 3 |
| GO:0003180 | aortic valve morphogenesis | Feb-97 | 28/18866 | 0.00907 | 0.01872 | 0.00798 | NOS3/TGFB1 | 2 |
| GO:0022011 | myelination in peripheral nervous system | Feb-97 | 28/18866 | 0.00907 | 0.01872 | 0.00798 | AKT1/SOD1 | 2 |
| GO:0031069 | hair follicle morphogenesis | Feb-97 | 28/18866 | 0.00907 | 0.01872 | 0.00798 | BCL2/CTNNB1 | 2 |
| GO:0031645 | negative regulation of nervous system process | Feb-97 | 28/18866 | 0.00907 | 0.01872 | 0.00798 | IL10/PTEN | 2 |
| GO:0032292 | peripheral nervous system axon ensheathment | Feb-97 | 28/18866 | 0.00907 | 0.01872 | 0.00798 | AKT1/SOD1 | 2 |
| GO:0033598 | mammary gland epithelial cell proliferation | Feb-97 | 28/18866 | 0.00907 | 0.01872 | 0.00798 | ESR1/MAPK1 | 2 |
| GO:0045686 | negative regulation of glial cell differentiation | Feb-97 | 28/18866 | 0.00907 | 0.01872 | 0.00798 | CTNNB1/F2 | 2 |
| GO:0071280 | cellular response to copper ion | Feb-97 | 28/18866 | 0.00907 | 0.01872 | 0.00798 | CYP1A1/CYP1A2 | 2 |
| GO:0090200 | positive regulation of release of cytochrome c from mitochondria | Feb-97 | 28/18866 | 0.00907 | 0.01872 | 0.00798 | MMP9/TP53 | 2 |
| GO:1905563 | negative regulation of vascular endothelial cell proliferation | Feb-97 | 28/18866 | 0.00907 | 0.01872 | 0.00798 | CCL2/PPARG | 2 |
| GO:1990776 | response to angiotensin | Feb-97 | 28/18866 | 0.00907 | 0.01872 | 0.00798 | CAV1/PTGS2 | 2 |
| GO:2000191 | regulation of fatty acid transport | Feb-97 | 28/18866 | 0.00907 | 0.01872 | 0.00798 | AKT1/IL1B | 2 |
| GO:0006874 | cellular calcium ion homeostasis | Jul-97 | 456/18866 | 0.00915 | 0.01889 | 0.00805 | BCL2/CAV1/ESR1/F2/IL2/PIK3CG/PTGER3 | 7 |
| GO:2000058 | regulation of ubiquitin-dependent protein catabolic process | Apr-97 | 159/18866 | 0.00918 | 0.01893 | 0.00807 | AKT1/CAV1/EGF/PTEN | 4 |
| GO:0014031 | mesenchymal cell development | Mar-97 | 84/18866 | 0.00929 | 0.01916 | 0.00817 | BCL2/MAPK1/MAPK3 | 3 |
| GO:1901991 | negative regulation of mitotic cell cycle phase transition | May-97 | 251/18866 | 0.00958 | 0.01973 | 0.00841 | BCL2/CCL2/CDKN1A/PTEN/TP53 | 5 |
| GO:0000723 | telomere maintenance | Apr-97 | 161/18866 | 0.00958 | 0.01973 | 0.00841 | CTNNB1/MAPK1/MAPK3/MYC | 4 |
| GO:0009199 | ribonucleoside triphosphate metabolic process | Mar-97 | 85/18866 | 0.0096 | 0.01974 | 0.00842 | IL4/STAT3/TGFB1 | 3 |
| GO:0070509 | calcium ion import | Mar-97 | 85/18866 | 0.0096 | 0.01974 | 0.00842 | CCL2/CTNNB1/EGF | 3 |
| GO:0001773 | myeloid dendritic cell activation | Feb-97 | 29/18866 | 0.0097 | 0.01982 | 0.00845 | IL10/IL4 | 2 |
| GO:0001782 | B cell homeostasis | Feb-97 | 29/18866 | 0.0097 | 0.01982 | 0.00845 | BCL2/CASP3 | 2 |
| GO:0019048 | modulation by virus of host process | Feb-97 | 29/18866 | 0.0097 | 0.01982 | 0.00845 | BCL2L1/RXRA | 2 |
| GO:0036336 | dendritic cell migration | Feb-97 | 29/18866 | 0.0097 | 0.01982 | 0.00845 | ALOX5/PIK3CG | 2 |
| GO:0042634 | regulation of hair cycle | Feb-97 | 29/18866 | 0.0097 | 0.01982 | 0.00845 | CTNNB1/TNF | 2 |
| GO:0045671 | negative regulation of osteoclast differentiation | Feb-97 | 29/18866 | 0.0097 | 0.01982 | 0.00845 | CTNNB1/IL4 | 2 |
| GO:0045822 | negative regulation of heart contraction | Feb-97 | 29/18866 | 0.0097 | 0.01982 | 0.00845 | IL2/PIK3CG | 2 |
| GO:0048679 | regulation of axon regeneration | Feb-97 | 29/18866 | 0.0097 | 0.01982 | 0.00845 | PTEN/SPP1 | 2 |
| GO:0048873 | homeostasis of number of cells within a tissue | Feb-97 | 29/18866 | 0.0097 | 0.01982 | 0.00845 | BCL2/NOS3 | 2 |
| GO:0060441 | epithelial tube branching involved in lung morphogenesis | Feb-97 | 29/18866 | 0.0097 | 0.01982 | 0.00845 | CTNNB1/TNF | 2 |
| GO:0071624 | positive regulation of granulocyte chemotaxis | Feb-97 | 29/18866 | 0.0097 | 0.01982 | 0.00845 | CXCL8/IL4 | 2 |
| GO:1901017 | negative regulation of potassium ion transmembrane transporter activity | Feb-97 | 29/18866 | 0.0097 | 0.01982 | 0.00845 | CAV1/PTEN | 2 |
| GO:2000108 | positive regulation of leukocyte apoptotic process | Feb-97 | 29/18866 | 0.0097 | 0.01982 | 0.00845 | IL10/TP53 | 2 |
| GO:0009144 | purine nucleoside triphosphate metabolic process | Mar-97 | 87/18866 | 0.01023 | 0.02085 | 0.00889 | IL4/STAT3/TGFB1 | 3 |
| GO:0030512 | negative regulation of transforming growth factor beta receptor signaling pathway | Mar-97 | 87/18866 | 0.01023 | 0.02085 | 0.00889 | CAV1/TGFB1/TP53 | 3 |
| GO:0090049 | regulation of cell migration involved in sprouting angiogenesis | Mar-97 | 87/18866 | 0.01023 | 0.02085 | 0.00889 | HMOX1/PTGS2/VEGFA | 3 |
| GO:0001516 | prostaglandin biosynthetic process | Feb-97 | 30/18866 | 0.01036 | 0.021 | 0.00895 | IL1B/PTGS2 | 2 |
| GO:0033137 | negative regulation of peptidyl-serine phosphorylation | Feb-97 | 30/18866 | 0.01036 | 0.021 | 0.00895 | CAV1/PTEN | 2 |
| GO:0035066 | positive regulation of histone acetylation | Feb-97 | 30/18866 | 0.01036 | 0.021 | 0.00895 | IL1B/MAPK3 | 2 |
| GO:0036475 | neuron death in response to oxidative stress | Feb-97 | 30/18866 | 0.01036 | 0.021 | 0.00895 | CTNNB1/IL10 | 2 |
| GO:0046457 | prostanoid biosynthetic process | Feb-97 | 30/18866 | 0.01036 | 0.021 | 0.00895 | IL1B/PTGS2 | 2 |
| GO:0061082 | myeloid leukocyte cytokine production | Feb-97 | 30/18866 | 0.01036 | 0.021 | 0.00895 | HMOX1/TGFB1 | 2 |
| GO:0070168 | negative regulation of biomineral tissue development | Feb-97 | 30/18866 | 0.01036 | 0.021 | 0.00895 | NOS3/TGFB1 | 2 |
| GO:0070723 | response to cholesterol | Feb-97 | 30/18866 | 0.01036 | 0.021 | 0.00895 | F7/TGFB1 | 2 |
| GO:0110150 | negative regulation of biomineralization | Feb-97 | 30/18866 | 0.01036 | 0.021 | 0.00895 | NOS3/TGFB1 | 2 |
| GO:1900027 | regulation of ruffle assembly | Feb-97 | 30/18866 | 0.01036 | 0.021 | 0.00895 | CAV1/ICAM1 | 2 |
| GO:1904837 | beta-catenin-TCF complex assembly | Feb-97 | 30/18866 | 0.01036 | 0.021 | 0.00895 | CTNNB1/MYC | 2 |
| GO:0008016 | regulation of heart contraction | May-97 | 256/18866 | 0.01037 | 0.021 | 0.00895 | CAV1/IL2/NOS3/PIK3CG/SREBF1 | 5 |
| GO:0010256 | endomembrane system organization | Jul-97 | 468/18866 | 0.01046 | 0.02116 | 0.00902 | AKT1/AR/CAV1/MAPK1/MAPK3/PTEN/SOD1 | 7 |
| GO:0016570 | histone modification | Jul-97 | 468/18866 | 0.01046 | 0.02116 | 0.00902 | CTNNB1/IL1B/MAPK3/MAPK8/SREBF1/TP53/VEGFA | 7 |
| GO:0055074 | calcium ion homeostasis | Jul-97 | 468/18866 | 0.01046 | 0.02116 | 0.00902 | BCL2/CAV1/ESR1/F2/IL2/PIK3CG/PTGER3 | 7 |
| GO:0032410 | negative regulation of transporter activity | Mar-97 | 88/18866 | 0.01055 | 0.02132 | 0.00909 | CAV1/MMP9/PTEN | 3 |
| GO:0007178 | transmembrane receptor protein serine/threonine kinase signaling pathway | Jun-97 | 359/18866 | 0.01056 | 0.02134 | 0.0091 | CAV1/FOS/JUN/MAPK3/TGFB1/TP53 | 6 |
| GO:0007093 | mitotic cell cycle checkpoint | Apr-97 | 166/18866 | 0.01063 | 0.02145 | 0.00915 | BCL2L1/CDKN1A/TGFB1/TP53 | 4 |
| GO:1903531 | negative regulation of secretion by cell | Apr-97 | 166/18866 | 0.01063 | 0.02145 | 0.00915 | ADIPOQ/HMOX1/IL1B/SREBF1 | 4 |
| GO:0021537 | telencephalon development | May-97 | 259/18866 | 0.01086 | 0.0219 | 0.00934 | CASP3/CTNNB1/EGFR/HSD3B1/PTEN | 5 |
| GO:0043506 | regulation of JUN kinase activity | Mar-97 | 89/18866 | 0.01088 | 0.0219 | 0.00934 | CD40LG/GSTP1/TNF | 3 |
| GO:0043537 | negative regulation of blood vessel endothelial cell migration | Mar-97 | 89/18866 | 0.01088 | 0.0219 | 0.00934 | PPARG/TGFB1/TNF | 3 |
| GO:1903845 | negative regulation of cellular response to transforming growth factor beta stimulus | Mar-97 | 89/18866 | 0.01088 | 0.0219 | 0.00934 | CAV1/TGFB1/TP53 | 3 |
| GO:0014044 | Schwann cell development | Feb-97 | 31/18866 | 0.01104 | 0.02211 | 0.00943 | AKT1/SOD1 | 2 |
| GO:0045737 | positive regulation of cyclin-dependent protein serine/threonine kinase activity | Feb-97 | 31/18866 | 0.01104 | 0.02211 | 0.00943 | AKT1/EGFR | 2 |
| GO:0045948 | positive regulation of translational initiation | Feb-97 | 31/18866 | 0.01104 | 0.02211 | 0.00943 | RXRA/TNF | 2 |
| GO:0048710 | regulation of astrocyte differentiation | Feb-97 | 31/18866 | 0.01104 | 0.02211 | 0.00943 | F2/IL6 | 2 |
| GO:0060261 | positive regulation of transcription initiation from RNA polymerase II promoter | Feb-97 | 31/18866 | 0.01104 | 0.02211 | 0.00943 | ESR1/TP53 | 2 |
| GO:0060914 | heart formation | Feb-97 | 31/18866 | 0.01104 | 0.02211 | 0.00943 | CTNNB1/PIM1 | 2 |
| GO:1902230 | negative regulation of intrinsic apoptotic signaling pathway in response to DNA damage | Feb-97 | 31/18866 | 0.01104 | 0.02211 | 0.00943 | BCL2/BCL2L1 | 2 |
| GO:1902253 | regulation of intrinsic apoptotic signaling pathway by p53 class mediator | Feb-97 | 31/18866 | 0.01104 | 0.02211 | 0.00943 | BCL2/TP53 | 2 |
| GO:1905476 | negative regulation of protein localization to membrane | Feb-97 | 31/18866 | 0.01104 | 0.02211 | 0.00943 | BCL2L1/TGFB1 | 2 |
| GO:2000515 | negative regulation of CD4-positive, alpha-beta T cell activation | Feb-97 | 31/18866 | 0.01104 | 0.02211 | 0.00943 | IL2/IL4 | 2 |
| GO:0042475 | odontogenesis of dentin-containing tooth | Mar-97 | 91/18866 | 0.01155 | 0.02308 | 0.00984 | CTNNB1/PPARA/SERPINE1 | 3 |
| GO:0045682 | regulation of epidermis development | Mar-97 | 91/18866 | 0.01155 | 0.02308 | 0.00984 | CTNNB1/PPARD/TNF | 3 |
| GO:0051899 | membrane depolarization | Mar-97 | 91/18866 | 0.01155 | 0.02308 | 0.00984 | BCL2/CAV1/JUN | 3 |
| GO:1904063 | negative regulation of cation transmembrane transport | Mar-97 | 91/18866 | 0.01155 | 0.02308 | 0.00984 | CAV1/MMP9/PTEN | 3 |
| GO:0048863 | stem cell differentiation | May-97 | 264/18866 | 0.01173 | 0.02332 | 0.00994 | ESR1/MAPK1/MAPK3/STAT3/TP53 | 5 |
| GO:0002724 | regulation of T cell cytokine production | Feb-97 | 32/18866 | 0.01174 | 0.02332 | 0.00994 | IL1B/IL6 | 2 |
| GO:0003176 | aortic valve development | Feb-97 | 32/18866 | 0.01174 | 0.02332 | 0.00994 | NOS3/TGFB1 | 2 |
| GO:0035767 | endothelial cell chemotaxis | Feb-97 | 32/18866 | 0.01174 | 0.02332 | 0.00994 | HSPB1/VEGFA | 2 |
| GO:0042744 | hydrogen peroxide catabolic process | Feb-97 | 32/18866 | 0.01174 | 0.02332 | 0.00994 | CAT/MPO | 2 |
| GO:0043304 | regulation of mast cell degranulation | Feb-97 | 32/18866 | 0.01174 | 0.02332 | 0.00994 | HMOX1/IL4 | 2 |
| GO:0045589 | regulation of regulatory T cell differentiation | Feb-97 | 32/18866 | 0.01174 | 0.02332 | 0.00994 | IFNG/IL2 | 2 |
| GO:0048730 | epidermis morphogenesis | Feb-97 | 32/18866 | 0.01174 | 0.02332 | 0.00994 | BCL2/CTNNB1 | 2 |
| GO:0050869 | negative regulation of B cell activation | Feb-97 | 32/18866 | 0.01174 | 0.02332 | 0.00994 | CASP3/IL10 | 2 |
| GO:0070570 | regulation of neuron projection regeneration | Feb-97 | 32/18866 | 0.01174 | 0.02332 | 0.00994 | PTEN/SPP1 | 2 |
| GO:0071295 | cellular response to vitamin | Feb-97 | 32/18866 | 0.01174 | 0.02332 | 0.00994 | PIM1/PPARG | 2 |
| GO:1903321 | negative regulation of protein modification by small protein conjugation or removal | Mar-97 | 92/18866 | 0.01189 | 0.02361 | 0.01006 | AKT1/CAV1/CTNNB1 | 3 |
| GO:1905897 | regulation of response to endoplasmic reticulum stress | Mar-97 | 92/18866 | 0.01189 | 0.02361 | 0.01006 | ALOX5/BCL2L1/CAV1 | 3 |
| GO:0032412 | regulation of ion transmembrane transporter activity | May-97 | 265/18866 | 0.01191 | 0.02361 | 0.01007 | CAV1/CCL2/IFNG/MMP9/PTEN | 5 |
| GO:0016569 | covalent chromatin modification | Jul-97 | 481/18866 | 0.01203 | 0.02385 | 0.01017 | CTNNB1/IL1B/MAPK3/MAPK8/SREBF1/TP53/VEGFA | 7 |
| GO:0007409 | axonogenesis | Jul-97 | 482/18866 | 0.01216 | 0.02409 | 0.01027 | BCL2/ERBB2/MAPK1/MAPK3/PTEN/SPP1/VEGFA | 7 |
| GO:0006937 | regulation of muscle contraction | Apr-97 | 173/18866 | 0.01222 | 0.0242 | 0.01032 | CAV1/PIK3CG/PTGS2/SOD1 | 4 |
| GO:0050954 | sensory perception of mechanical stimulus | Apr-97 | 173/18866 | 0.01222 | 0.0242 | 0.01032 | BIRC5/CASP3/ICAM1/SOD1 | 4 |
| GO:0032088 | negative regulation of NF-kappaB transcription factor activity | Mar-97 | 93/18866 | 0.01225 | 0.0242 | 0.01032 | CAT/IL10/NFKBIA | 3 |
| GO:0051702 | interaction with symbiont | Mar-97 | 93/18866 | 0.01225 | 0.0242 | 0.01032 | CRP/F2/JUN | 3 |
| GO:0061097 | regulation of protein tyrosine kinase activity | Mar-97 | 93/18866 | 0.01225 | 0.0242 | 0.01032 | CAV1/EGF/ERBB3 | 3 |
| GO:0001975 | response to amphetamine | Feb-97 | 33/18866 | 0.01245 | 0.02446 | 0.01043 | ICAM1/SOD1 | 2 |
| GO:0002691 | regulation of cellular extravasation | Feb-97 | 33/18866 | 0.01245 | 0.02446 | 0.01043 | ICAM1/SELE | 2 |
| GO:0002861 | regulation of inflammatory response to antigenic stimulus | Feb-97 | 33/18866 | 0.01245 | 0.02446 | 0.01043 | IL10/TNF | 2 |
| GO:0033006 | regulation of mast cell activation involved in immune response | Feb-97 | 33/18866 | 0.01245 | 0.02446 | 0.01043 | HMOX1/IL4 | 2 |
| GO:0042573 | retinoic acid metabolic process | Feb-97 | 33/18866 | 0.01245 | 0.02446 | 0.01043 | CYP1A1/CYP3A4 | 2 |
| GO:0048384 | retinoic acid receptor signaling pathway | Feb-97 | 33/18866 | 0.01245 | 0.02446 | 0.01043 | PPARG/RXRA | 2 |
| GO:0061311 | cell surface receptor signaling pathway involved in heart development | Feb-97 | 33/18866 | 0.01245 | 0.02446 | 0.01043 | CTNNB1/TGFB1 | 2 |
| GO:0090022 | regulation of neutrophil chemotaxis | Feb-97 | 33/18866 | 0.01245 | 0.02446 | 0.01043 | CXCL8/DPP4 | 2 |
| GO:1901380 | negative regulation of potassium ion transmembrane transport | Feb-97 | 33/18866 | 0.01245 | 0.02446 | 0.01043 | CAV1/PTEN | 2 |
| GO:1905048 | regulation of metallopeptidase activity | Feb-97 | 33/18866 | 0.01245 | 0.02446 | 0.01043 | MAPK3/STAT3 | 2 |
| GO:2000758 | positive regulation of peptidyl-lysine acetylation | Feb-97 | 33/18866 | 0.01245 | 0.02446 | 0.01043 | IL1B/MAPK3 | 2 |
| GO:0003205 | cardiac chamber development | Apr-97 | 174/18866 | 0.01246 | 0.02446 | 0.01043 | NOS3/RXRA/TGFB1/TP53 | 4 |
| GO:0032200 | telomere organization | Apr-97 | 174/18866 | 0.01246 | 0.02446 | 0.01043 | CTNNB1/MAPK1/MAPK3/MYC | 4 |
| GO:0030641 | regulation of cellular pH | Mar-97 | 94/18866 | 0.0126 | 0.0247 | 0.01053 | BCL2/MAPK1/MAPK3 | 3 |
| GO:0034308 | primary alcohol metabolic process | Mar-97 | 94/18866 | 0.0126 | 0.0247 | 0.01053 | CYP1A1/CYP1A2/CYP3A4 | 3 |
| GO:1901992 | positive regulation of mitotic cell cycle phase transition | Mar-97 | 94/18866 | 0.0126 | 0.0247 | 0.01053 | AKT1/CYP1A1/EGFR | 3 |
| GO:0010921 | regulation of phosphatase activity | Apr-97 | 175/18866 | 0.01271 | 0.02488 | 0.01061 | IFNG/IGFBP3/IKBKB/TNF | 4 |
| GO:1901988 | negative regulation of cell cycle phase transition | May-97 | 270/18866 | 0.01282 | 0.0251 | 0.0107 | BCL2/CCL2/CDKN1A/PTEN/TP53 | 5 |
| GO:0030111 | regulation of Wnt signaling pathway | Jun-97 | 375/18866 | 0.01288 | 0.0252 | 0.01075 | CAV1/CTNNB1/EGF/EGFR/ESR1/TGFB1 | 6 |
| GO:0007589 | body fluid secretion | Mar-97 | 95/18866 | 0.01297 | 0.02533 | 0.0108 | CAV1/VEGFA/XDH | 3 |
| GO:0070167 | regulation of biomineral tissue development | Mar-97 | 95/18866 | 0.01297 | 0.02533 | 0.0108 | ALOX5/NOS3/TGFB1 | 3 |
| GO:0110149 | regulation of biomineralization | Mar-97 | 95/18866 | 0.01297 | 0.02533 | 0.0108 | ALOX5/NOS3/TGFB1 | 3 |
| GO:0000737 | DNA catabolic process, endonucleolytic | Feb-97 | 34/18866 | 0.01319 | 0.02564 | 0.01093 | CASP3/IL6 | 2 |
| GO:0007202 | activation of phospholipase C activity | Feb-97 | 34/18866 | 0.01319 | 0.02564 | 0.01093 | EGFR/SELE | 2 |
| GO:0010762 | regulation of fibroblast migration | Feb-97 | 34/18866 | 0.01319 | 0.02564 | 0.01093 | AKT1/TGFB1 | 2 |
| GO:0042311 | vasodilation | Feb-97 | 34/18866 | 0.01319 | 0.02564 | 0.01093 | NOS3/SOD1 | 2 |
| GO:0048566 | embryonic digestive tract development | Feb-97 | 34/18866 | 0.01319 | 0.02564 | 0.01093 | CXCL8/TNF | 2 |
| GO:0055022 | negative regulation of cardiac muscle tissue growth | Feb-97 | 34/18866 | 0.01319 | 0.02564 | 0.01093 | PPARA/PTEN | 2 |
| GO:0055094 | response to lipoprotein particle | Feb-97 | 34/18866 | 0.01319 | 0.02564 | 0.01093 | AKT1/PPARG | 2 |
| GO:0061117 | negative regulation of heart growth | Feb-97 | 34/18866 | 0.01319 | 0.02564 | 0.01093 | PPARA/PTEN | 2 |
| GO:0070102 | interleukin-6-mediated signaling pathway | Feb-97 | 34/18866 | 0.01319 | 0.02564 | 0.01093 | IL6/STAT3 | 2 |
| GO:0072503 | cellular divalent inorganic cation homeostasis | Jul-97 | 492/18866 | 0.01348 | 0.0262 | 0.01117 | BCL2/CAV1/ESR1/F2/IL2/PIK3CG/PTGER3 | 7 |
| GO:0046634 | regulation of alpha-beta T cell activation | Mar-97 | 97/18866 | 0.01371 | 0.02663 | 0.01135 | IFNG/IL2/IL4 | 3 |
| GO:0009112 | nucleobase metabolic process | Feb-97 | 35/18866 | 0.01394 | 0.02695 | 0.01149 | MAPK1/XDH | 2 |
| GO:0030262 | apoptotic nuclear changes | Feb-97 | 35/18866 | 0.01394 | 0.02695 | 0.01149 | CASP3/IL6 | 2 |
| GO:0032735 | positive regulation of interleukin-12 production | Feb-97 | 35/18866 | 0.01394 | 0.02695 | 0.01149 | CD40LG/IFNG | 2 |
| GO:0034390 | smooth muscle cell apoptotic process | Feb-97 | 35/18866 | 0.01394 | 0.02695 | 0.01149 | IFNG/PPARG | 2 |
| GO:0034391 | regulation of smooth muscle cell apoptotic process | Feb-97 | 35/18866 | 0.01394 | 0.02695 | 0.01149 | IFNG/PPARG | 2 |
| GO:0036314 | response to sterol | Feb-97 | 35/18866 | 0.01394 | 0.02695 | 0.01149 | F7/TGFB1 | 2 |
| GO:0045066 | regulatory T cell differentiation | Feb-97 | 35/18866 | 0.01394 | 0.02695 | 0.01149 | IFNG/IL2 | 2 |
| GO:0060674 | placenta blood vessel development | Feb-97 | 35/18866 | 0.01394 | 0.02695 | 0.01149 | AKT1/MAPK1 | 2 |
| GO:0070884 | regulation of calcineurin-NFAT signaling cascade | Feb-97 | 35/18866 | 0.01394 | 0.02695 | 0.01149 | ERBB3/TNF | 2 |
| GO:0048010 | vascular endothelial growth factor receptor signaling pathway | Mar-97 | 98/18866 | 0.01409 | 0.02723 | 0.01161 | HSPB1/IL1B/VEGFA | 3 |
| GO:0098742 | cell-cell adhesion via plasma-membrane adhesion molecules | May-97 | 277/18866 | 0.01419 | 0.0274 | 0.01168 | ADIPOQ/ICAM1/IL10/SELE/VCAM1 | 5 |
| GO:0008361 | regulation of cell size | Apr-97 | 181/18866 | 0.01422 | 0.02745 | 0.01171 | AKT1/PTEN/SPP1/VEGFA | 4 |
| GO:0007044 | cell-substrate junction assembly | Mar-97 | 99/18866 | 0.01448 | 0.02793 | 0.01191 | BCL2/PTEN/VEGFA | 3 |
| GO:0090288 | negative regulation of cellular response to growth factor stimulus | Apr-97 | 182/18866 | 0.01449 | 0.02793 | 0.01191 | CAV1/TGFB1/TP53/XDH | 4 |
| GO:0001569 | branching involved in blood vessel morphogenesis | Feb-97 | 36/18866 | 0.01471 | 0.02826 | 0.01205 | CTNNB1/VEGFA | 2 |
| GO:0010092 | specification of animal organ identity | Feb-97 | 36/18866 | 0.01471 | 0.02826 | 0.01205 | AR/CTNNB1 | 2 |
| GO:0031128 | developmental induction | Feb-97 | 36/18866 | 0.01471 | 0.02826 | 0.01205 | AR/CTNNB1 | 2 |
| GO:0045622 | regulation of T-helper cell differentiation | Feb-97 | 36/18866 | 0.01471 | 0.02826 | 0.01205 | IL2/IL4 | 2 |
| GO:0071402 | cellular response to lipoprotein particle stimulus | Feb-97 | 36/18866 | 0.01471 | 0.02826 | 0.01205 | AKT1/PPARG | 2 |
| GO:0106056 | regulation of calcineurin-mediated signaling | Feb-97 | 36/18866 | 0.01471 | 0.02826 | 0.01205 | ERBB3/TNF | 2 |
| GO:2000310 | regulation of NMDA receptor activity | Feb-97 | 36/18866 | 0.01471 | 0.02826 | 0.01205 | CCL2/IFNG | 2 |
| GO:0030177 | positive regulation of Wnt signaling pathway | Apr-97 | 183/18866 | 0.01475 | 0.02833 | 0.01208 | CAV1/EGF/EGFR/TGFB1 | 4 |
| GO:0022900 | electron transport chain | Apr-97 | 184/18866 | 0.01502 | 0.02883 | 0.01229 | CYP19A1/CYP1A2/GSR/XDH | 4 |
| GO:0010522 | regulation of calcium ion transport into cytosol | Mar-97 | 101/18866 | 0.01527 | 0.02928 | 0.01248 | BCL2/CAV1/F2 | 3 |
| GO:0060191 | regulation of lipase activity | Mar-97 | 101/18866 | 0.01527 | 0.02928 | 0.01248 | EGFR/ESR1/SELE | 3 |
| GO:2001257 | regulation of cation channel activity | Apr-97 | 185/18866 | 0.0153 | 0.02931 | 0.0125 | CAV1/CCL2/IFNG/MMP9 | 4 |
| GO:0006921 | cellular component disassembly involved in execution phase of apoptosis | Feb-97 | 37/18866 | 0.0155 | 0.0296 | 0.01262 | CASP3/IL6 | 2 |
| GO:0045730 | respiratory burst | Feb-97 | 37/18866 | 0.0155 | 0.0296 | 0.01262 | MPO/PIK3CG | 2 |
| GO:0060260 | regulation of transcription initiation from RNA polymerase II promoter | Feb-97 | 37/18866 | 0.0155 | 0.0296 | 0.01262 | ESR1/TP53 | 2 |
| GO:0070897 | transcription preinitiation complex assembly | Feb-97 | 37/18866 | 0.0155 | 0.0296 | 0.01262 | ESR1/TP53 | 2 |
| GO:0071542 | dopaminergic neuron differentiation | Feb-97 | 37/18866 | 0.0155 | 0.0296 | 0.01262 | CTNNB1/VEGFA | 2 |
| GO:0090218 | positive regulation of lipid kinase activity | Feb-97 | 37/18866 | 0.0155 | 0.0296 | 0.01262 | F2/TGFB1 | 2 |
| GO:1905314 | semi-lunar valve development | Feb-97 | 37/18866 | 0.0155 | 0.0296 | 0.01262 | NOS3/TGFB1 | 2 |
| GO:0150115 | cell-substrate junction organization | Mar-97 | 102/18866 | 0.01568 | 0.02992 | 0.01276 | BCL2/PTEN/VEGFA | 3 |
| GO:0050821 | protein stabilization | Apr-97 | 187/18866 | 0.01585 | 0.03024 | 0.01289 | CDKN1A/PIM1/PTEN/TP53 | 4 |
| GO:0006885 | regulation of pH | Mar-97 | 103/18866 | 0.01609 | 0.03065 | 0.01307 | BCL2/MAPK1/MAPK3 | 3 |
| GO:1901570 | fatty acid derivative biosynthetic process | Mar-97 | 103/18866 | 0.01609 | 0.03065 | 0.01307 | ALOX5/IL1B/PTGS2 | 3 |
| GO:0002385 | mucosal immune response | Feb-97 | 38/18866 | 0.01631 | 0.031 | 0.01322 | IL4/NOS2 | 2 |
| GO:0019433 | triglyceride catabolic process | Feb-97 | 38/18866 | 0.01631 | 0.031 | 0.01322 | APOB/PIK3CG | 2 |
| GO:0044003 | modulation by symbiont of host process | Feb-97 | 38/18866 | 0.01631 | 0.031 | 0.01322 | BCL2L1/RXRA | 2 |
| GO:0060045 | positive regulation of cardiac muscle cell proliferation | Feb-97 | 38/18866 | 0.01631 | 0.031 | 0.01322 | MAPK1/PIM1 | 2 |
| GO:1903523 | negative regulation of blood circulation | Feb-97 | 38/18866 | 0.01631 | 0.031 | 0.01322 | IL2/PIK3CG | 2 |
| GO:0007200 | phospholipase C-activating G protein-coupled receptor signaling pathway | Mar-97 | 104/18866 | 0.01651 | 0.03135 | 0.01337 | ESR1/F2/PTGER3 | 3 |
| GO:0006575 | cellular modified amino acid metabolic process | Apr-97 | 190/18866 | 0.01671 | 0.03172 | 0.01353 | GSR/GSTM1/GSTP1/SOD1 | 4 |
| GO:0001662 | behavioral fear response | Feb-97 | 39/18866 | 0.01713 | 0.03238 | 0.01381 | BCL2/DPP4 | 2 |
| GO:0010831 | positive regulation of myotube differentiation | Feb-97 | 39/18866 | 0.01713 | 0.03238 | 0.01381 | BCL2/IL4 | 2 |
| GO:0030866 | cortical actin cytoskeleton organization | Feb-97 | 39/18866 | 0.01713 | 0.03238 | 0.01381 | IKBKB/TNF | 2 |
| GO:0033146 | regulation of intracellular estrogen receptor signaling pathway | Feb-97 | 39/18866 | 0.01713 | 0.03238 | 0.01381 | AR/ESR1 | 2 |
| GO:0045022 | early endosome to late endosome transport | Feb-97 | 39/18866 | 0.01713 | 0.03238 | 0.01381 | MAPK1/MAPK3 | 2 |
| GO:0046326 | positive regulation of glucose import | Feb-97 | 39/18866 | 0.01713 | 0.03238 | 0.01381 | ADIPOQ/AKT1 | 2 |
| GO:0046329 | negative regulation of JNK cascade | Feb-97 | 39/18866 | 0.01713 | 0.03238 | 0.01381 | AKT1/GSTP1 | 2 |
| GO:0071709 | membrane assembly | Feb-97 | 39/18866 | 0.01713 | 0.03238 | 0.01381 | CAV1/PTEN | 2 |
| GO:1902229 | regulation of intrinsic apoptotic signaling pathway in response to DNA damage | Feb-97 | 39/18866 | 0.01713 | 0.03238 | 0.01381 | BCL2/BCL2L1 | 2 |
| GO:0071868 | cellular response to monoamine stimulus | Mar-97 | 106/18866 | 0.01736 | 0.03277 | 0.01397 | ADIPOQ/MAPK1/MAPK3 | 3 |
| GO:0071870 | cellular response to catecholamine stimulus | Mar-97 | 106/18866 | 0.01736 | 0.03277 | 0.01397 | ADIPOQ/MAPK1/MAPK3 | 3 |
| GO:0051604 | protein maturation | May-97 | 293/18866 | 0.01767 | 0.03333 | 0.01421 | CASP3/F3/F7/PLAT/SERPINE1 | 5 |
| GO:0010596 | negative regulation of endothelial cell migration | Mar-97 | 107/18866 | 0.01779 | 0.03354 | 0.0143 | PPARG/TGFB1/TNF | 3 |
| GO:0043266 | regulation of potassium ion transport | Mar-97 | 107/18866 | 0.01779 | 0.03354 | 0.0143 | CAV1/NOS3/PTEN | 3 |
| GO:0002209 | behavioral defense response | Feb-97 | 40/18866 | 0.01798 | 0.03377 | 0.0144 | BCL2/DPP4 | 2 |
| GO:0016572 | histone phosphorylation | Feb-97 | 40/18866 | 0.01798 | 0.03377 | 0.0144 | IL1B/MAPK3 | 2 |
| GO:0030501 | positive regulation of bone mineralization | Feb-97 | 40/18866 | 0.01798 | 0.03377 | 0.0144 | ALOX5/TGFB1 | 2 |
| GO:0046636 | negative regulation of alpha-beta T cell activation | Feb-97 | 40/18866 | 0.01798 | 0.03377 | 0.0144 | IL2/IL4 | 2 |
| GO:0048713 | regulation of oligodendrocyte differentiation | Feb-97 | 40/18866 | 0.01798 | 0.03377 | 0.0144 | CTNNB1/PPARG | 2 |
| GO:0050691 | regulation of defense response to virus by host | Feb-97 | 40/18866 | 0.01798 | 0.03377 | 0.0144 | IL1B/IL4 | 2 |
| GO:0072210 | metanephric nephron development | Feb-97 | 40/18866 | 0.01798 | 0.03377 | 0.0144 | ADIPOQ/CTNNB1 | 2 |
| GO:0060828 | regulation of canonical Wnt signaling pathway | May-97 | 295/18866 | 0.01814 | 0.03405 | 0.01452 | CAV1/CTNNB1/EGF/EGFR/TGFB1 | 5 |
| GO:0001909 | leukocyte mediated cytotoxicity | Mar-97 | 108/18866 | 0.01824 | 0.03419 | 0.01458 | F2/ICAM1/NOS2 | 3 |
| GO:0006024 | glycosaminoglycan biosynthetic process | Mar-97 | 108/18866 | 0.01824 | 0.03419 | 0.01458 | EGF/IL1B/TGFB1 | 3 |
| GO:0032526 | response to retinoic acid | Mar-97 | 108/18866 | 0.01824 | 0.03419 | 0.01458 | PPARG/RXRA/SREBF1 | 3 |
| GO:0021761 | limbic system development | Mar-97 | 109/18866 | 0.01868 | 0.03501 | 0.01493 | CASP3/HSD3B1/PTEN | 3 |
| GO:0033574 | response to testosterone | Feb-97 | 41/18866 | 0.01884 | 0.03522 | 0.01502 | AR/SPP1 | 2 |
| GO:0042596 | fear response | Feb-97 | 41/18866 | 0.01884 | 0.03522 | 0.01502 | BCL2/DPP4 | 2 |
| GO:0046825 | regulation of protein export from nucleus | Feb-97 | 41/18866 | 0.01884 | 0.03522 | 0.01502 | IL1B/TP53 | 2 |
| GO:2000826 | regulation of heart morphogenesis | Feb-97 | 41/18866 | 0.01884 | 0.03522 | 0.01502 | CTNNB1/PIM1 | 2 |
| GO:0002831 | regulation of response to biotic stimulus | Jun-97 | 409/18866 | 0.01896 | 0.03543 | 0.01511 | IFNG/IKBKB/IL1B/IL4/MAPK3/PPARG | 6 |
| GO:0006310 | DNA recombination | May-97 | 299/18866 | 0.0191 | 0.03566 | 0.0152 | CD40LG/IL10/IL2/IL4/TGFB1 | 5 |
| GO:0009141 | nucleoside triphosphate metabolic process | Mar-97 | 110/18866 | 0.01914 | 0.03566 | 0.0152 | IL4/STAT3/TGFB1 | 3 |
| GO:0032680 | regulation of tumor necrosis factor production | Mar-97 | 110/18866 | 0.01914 | 0.03566 | 0.0152 | ADIPOQ/GSTP1/IL10 | 3 |
| GO:0071867 | response to monoamine | Mar-97 | 110/18866 | 0.01914 | 0.03566 | 0.0152 | ADIPOQ/MAPK1/MAPK3 | 3 |
| GO:0071869 | response to catecholamine | Mar-97 | 110/18866 | 0.01914 | 0.03566 | 0.0152 | ADIPOQ/MAPK1/MAPK3 | 3 |
| GO:1901989 | positive regulation of cell cycle phase transition | Mar-97 | 110/18866 | 0.01914 | 0.03566 | 0.0152 | AKT1/CYP1A1/EGFR | 3 |
| GO:0010907 | positive regulation of glucose metabolic process | Feb-97 | 42/18866 | 0.01971 | 0.0366 | 0.01561 | AKT1/PPARA | 2 |
| GO:0021795 | cerebral cortex cell migration | Feb-97 | 42/18866 | 0.01971 | 0.0366 | 0.01561 | CTNNB1/EGFR | 2 |
| GO:0046006 | regulation of activated T cell proliferation | Feb-97 | 42/18866 | 0.01971 | 0.0366 | 0.01561 | CASP3/IL2 | 2 |
| GO:0046621 | negative regulation of organ growth | Feb-97 | 42/18866 | 0.01971 | 0.0366 | 0.01561 | PPARA/PTEN | 2 |
| GO:0071470 | cellular response to osmotic stress | Feb-97 | 42/18866 | 0.01971 | 0.0366 | 0.01561 | CASP3/PTGS2 | 2 |
| GO:0098927 | vesicle-mediated transport between endosomal compartments | Feb-97 | 42/18866 | 0.01971 | 0.0366 | 0.01561 | MAPK1/MAPK3 | 2 |
| GO:1902622 | regulation of neutrophil migration | Feb-97 | 42/18866 | 0.01971 | 0.0366 | 0.01561 | CXCL8/DPP4 | 2 |
| GO:0016052 | carbohydrate catabolic process | Apr-97 | 200/18866 | 0.01977 | 0.0367 | 0.01565 | IFNG/PPARA/STAT3/TP53 | 4 |
| GO:0005976 | polysaccharide metabolic process | Mar-97 | 112/18866 | 0.02006 | 0.03722 | 0.01587 | AKT1/EGF/TGFB1 | 3 |
| GO:1901605 | alpha-amino acid metabolic process | Apr-97 | 202/18866 | 0.02042 | 0.03787 | 0.01615 | NOS2/NOS3/ODC1/TYR | 4 |
| GO:0001953 | negative regulation of cell-matrix adhesion | Feb-97 | 43/18866 | 0.02061 | 0.03801 | 0.01621 | PTEN/SERPINE1 | 2 |
| GO:0002823 | negative regulation of adaptive immune response based on somatic recombination of immune receptors built from immunoglobulin superfamily domains | Feb-97 | 43/18866 | 0.02061 | 0.03801 | 0.01621 | IL2/IL4 | 2 |
| GO:0006308 | DNA catabolic process | Feb-97 | 43/18866 | 0.02061 | 0.03801 | 0.01621 | CASP3/IL6 | 2 |
| GO:0010470 | regulation of gastrulation | Feb-97 | 43/18866 | 0.02061 | 0.03801 | 0.01621 | ADIPOQ/IL10 | 2 |
| GO:0040019 | positive regulation of embryonic development | Feb-97 | 43/18866 | 0.02061 | 0.03801 | 0.01621 | AR/CTNNB1 | 2 |
| GO:0050850 | positive regulation of calcium-mediated signaling | Feb-97 | 43/18866 | 0.02061 | 0.03801 | 0.01621 | ERBB3/TNF | 2 |
| GO:0090207 | regulation of triglyceride metabolic process | Feb-97 | 43/18866 | 0.02061 | 0.03801 | 0.01621 | PIK3CG/SREBF1 | 2 |
| GO:1901031 | regulation of response to reactive oxygen species | Feb-97 | 43/18866 | 0.02061 | 0.03801 | 0.01621 | IL10/TNF | 2 |
| GO:1903146 | regulation of autophagy of mitochondrion | Feb-97 | 43/18866 | 0.02061 | 0.03801 | 0.01621 | SREBF1/TP53 | 2 |
| GO:2000648 | positive regulation of stem cell proliferation | Feb-97 | 43/18866 | 0.02061 | 0.03801 | 0.01621 | CTNNB1/VEGFA | 2 |
| GO:0030004 | cellular monovalent inorganic cation homeostasis | Mar-97 | 114/18866 | 0.02101 | 0.03873 | 0.01651 | BCL2/MAPK1/MAPK3 | 3 |
| GO:0032411 | positive regulation of transporter activity | Mar-97 | 114/18866 | 0.02101 | 0.03873 | 0.01651 | CCL2/IFNG/PON1 | 3 |
| GO:0006023 | aminoglycan biosynthetic process | Mar-97 | 115/18866 | 0.0215 | 0.03948 | 0.01683 | EGF/IL1B/TGFB1 | 3 |
| GO:0006096 | glycolytic process | Mar-97 | 115/18866 | 0.0215 | 0.03948 | 0.01683 | IFNG/PPARA/STAT3 | 3 |
| GO:0033003 | regulation of mast cell activation | Feb-97 | 44/18866 | 0.02151 | 0.03948 | 0.01683 | HMOX1/IL4 | 2 |
| GO:0033173 | calcineurin-NFAT signaling cascade | Feb-97 | 44/18866 | 0.02151 | 0.03948 | 0.01683 | ERBB3/TNF | 2 |
| GO:0044091 | membrane biogenesis | Feb-97 | 44/18866 | 0.02151 | 0.03948 | 0.01683 | CAV1/PTEN | 2 |
| GO:0045684 | positive regulation of epidermis development | Feb-97 | 44/18866 | 0.02151 | 0.03948 | 0.01683 | PPARD/TNF | 2 |
| GO:0045687 | positive regulation of glial cell differentiation | Feb-97 | 44/18866 | 0.02151 | 0.03948 | 0.01683 | PPARG/TGFB1 | 2 |
| GO:0097178 | ruffle assembly | Feb-97 | 44/18866 | 0.02151 | 0.03948 | 0.01683 | CAV1/ICAM1 | 2 |
| GO:1901985 | positive regulation of protein acetylation | Feb-97 | 44/18866 | 0.02151 | 0.03948 | 0.01683 | IL1B/MAPK3 | 2 |
| GO:0006757 | ATP generation from ADP | Mar-97 | 116/18866 | 0.02199 | 0.04029 | 0.01718 | IFNG/PPARA/STAT3 | 3 |
| GO:0032640 | tumor necrosis factor production | Mar-97 | 116/18866 | 0.02199 | 0.04029 | 0.01718 | ADIPOQ/GSTP1/IL10 | 3 |
| GO:1903828 | negative regulation of cellular protein localization | Mar-97 | 116/18866 | 0.02199 | 0.04029 | 0.01718 | ADIPOQ/BCL2L1/TGFB1 | 3 |
| GO:0001974 | blood vessel remodeling | Feb-97 | 45/18866 | 0.02244 | 0.04094 | 0.01745 | NOS3/TGFB1 | 2 |
| GO:0003197 | endocardial cushion development | Feb-97 | 45/18866 | 0.02244 | 0.04094 | 0.01745 | ERBB3/NOS3 | 2 |
| GO:0010828 | positive regulation of glucose transmembrane transport | Feb-97 | 45/18866 | 0.02244 | 0.04094 | 0.01745 | ADIPOQ/AKT1 | 2 |
| GO:0031018 | endocrine pancreas development | Feb-97 | 45/18866 | 0.02244 | 0.04094 | 0.01745 | AKT1/IL6 | 2 |
| GO:0035315 | hair cell differentiation | Feb-97 | 45/18866 | 0.02244 | 0.04094 | 0.01745 | CTNNB1/SOD1 | 2 |
| GO:0043114 | regulation of vascular permeability | Feb-97 | 45/18866 | 0.02244 | 0.04094 | 0.01745 | TGFB1/VEGFA | 2 |
| GO:0052372 | modulation by symbiont of entry into host | Feb-97 | 45/18866 | 0.02244 | 0.04094 | 0.01745 | CAV1/CXCL8 | 2 |
| GO:0055026 | negative regulation of cardiac muscle tissue development | Feb-97 | 45/18866 | 0.02244 | 0.04094 | 0.01745 | PPARA/PTEN | 2 |
| GO:1904646 | cellular response to amyloid-beta | Feb-97 | 45/18866 | 0.02244 | 0.04094 | 0.01745 | ICAM1/VCAM1 | 2 |
| GO:0030282 | bone mineralization | Mar-97 | 117/18866 | 0.02248 | 0.04097 | 0.01747 | ALOX5/PTGS2/TGFB1 | 3 |
| GO:0043666 | regulation of phosphoprotein phosphatase activity | Mar-97 | 117/18866 | 0.02248 | 0.04097 | 0.01747 | IGFBP3/IKBKB/TNF | 3 |
| GO:0008543 | fibroblast growth factor receptor signaling pathway | Mar-97 | 118/18866 | 0.02299 | 0.04187 | 0.01785 | CTNNB1/MAPK1/MAPK3 | 3 |
| GO:0001754 | eye photoreceptor cell differentiation | Feb-97 | 46/18866 | 0.02338 | 0.04245 | 0.0181 | STAT3/VEGFA | 2 |
| GO:0010761 | fibroblast migration | Feb-97 | 46/18866 | 0.02338 | 0.04245 | 0.0181 | AKT1/TGFB1 | 2 |
| GO:0014014 | negative regulation of gliogenesis | Feb-97 | 46/18866 | 0.02338 | 0.04245 | 0.0181 | CTNNB1/F2 | 2 |
| GO:0032309 | icosanoid secretion | Feb-97 | 46/18866 | 0.02338 | 0.04245 | 0.0181 | IL1B/NOS2 | 2 |
| GO:0032692 | negative regulation of interleukin-1 production | Feb-97 | 46/18866 | 0.02338 | 0.04245 | 0.0181 | GSTP1/IL10 | 2 |
| GO:0048066 | developmental pigmentation | Feb-97 | 46/18866 | 0.02338 | 0.04245 | 0.0181 | BCL2/TYR | 2 |
| GO:0050798 | activated T cell proliferation | Feb-97 | 46/18866 | 0.02338 | 0.04245 | 0.0181 | CASP3/IL2 | 2 |
| GO:0050808 | synapse organization | Jun-97 | 433/18866 | 0.02427 | 0.04404 | 0.01878 | ACHE/CTNNB1/ERBB2/IL10/PTEN/TNF | 6 |
| GO:0010559 | regulation of glycoprotein biosynthetic process | Feb-97 | 47/18866 | 0.02434 | 0.04404 | 0.01878 | BCL2/CTNNB1 | 2 |
| GO:0014003 | oligodendrocyte development | Feb-97 | 47/18866 | 0.02434 | 0.04404 | 0.01878 | GSTP1/PTEN | 2 |
| GO:0014075 | response to amine | Feb-97 | 47/18866 | 0.02434 | 0.04404 | 0.01878 | ICAM1/SOD1 | 2 |
| GO:0032369 | negative regulation of lipid transport | Feb-97 | 47/18866 | 0.02434 | 0.04404 | 0.01878 | AKT1/EGF | 2 |
| GO:0043300 | regulation of leukocyte degranulation | Feb-97 | 47/18866 | 0.02434 | 0.04404 | 0.01878 | HMOX1/IL4 | 2 |
| GO:0048599 | oocyte development | Feb-97 | 47/18866 | 0.02434 | 0.04404 | 0.01878 | BCL2/CTNNB1 | 2 |
| GO:0030218 | erythrocyte differentiation | Mar-97 | 121/18866 | 0.02453 | 0.04436 | 0.01891 | CASP3/STAT3/VEGFA | 3 |
| GO:0051928 | positive regulation of calcium ion transport | Mar-97 | 122/18866 | 0.02506 | 0.04529 | 0.01931 | CAV1/CCL2/F2 | 3 |
| GO:0002820 | negative regulation of adaptive immune response | Feb-97 | 48/18866 | 0.02532 | 0.04558 | 0.01943 | IL2/IL4 | 2 |
| GO:0008089 | anterograde axonal transport | Feb-97 | 48/18866 | 0.02532 | 0.04558 | 0.01943 | HSPB1/SOD1 | 2 |
| GO:0046461 | neutral lipid catabolic process | Feb-97 | 48/18866 | 0.02532 | 0.04558 | 0.01943 | APOB/PIK3CG | 2 |
| GO:0046464 | acylglycerol catabolic process | Feb-97 | 48/18866 | 0.02532 | 0.04558 | 0.01943 | APOB/PIK3CG | 2 |
| GO:0055010 | ventricular cardiac muscle tissue morphogenesis | Feb-97 | 48/18866 | 0.02532 | 0.04558 | 0.01943 | RXRA/TGFB1 | 2 |
| GO:0055023 | positive regulation of cardiac muscle tissue growth | Feb-97 | 48/18866 | 0.02532 | 0.04558 | 0.01943 | MAPK1/PIM1 | 2 |
| GO:0070169 | positive regulation of biomineral tissue development | Feb-97 | 48/18866 | 0.02532 | 0.04558 | 0.01943 | ALOX5/TGFB1 | 2 |
| GO:0110151 | positive regulation of biomineralization | Feb-97 | 48/18866 | 0.02532 | 0.04558 | 0.01943 | ALOX5/TGFB1 | 2 |
| GO:0051651 | maintenance of location in cell | Apr-97 | 216/18866 | 0.02535 | 0.04562 | 0.01945 | AKT1/ALB/CAV1/F2 | 4 |
| GO:0042471 | ear morphogenesis | Mar-97 | 123/18866 | 0.02559 | 0.04603 | 0.01962 | MAPK1/MAPK3/SOD1 | 3 |
| GO:0010810 | regulation of cell-substrate adhesion | Apr-97 | 217/18866 | 0.02573 | 0.04626 | 0.01972 | BCL2/PTEN/SERPINE1/VEGFA | 4 |
| GO:0046031 | ADP metabolic process | Mar-97 | 124/18866 | 0.02613 | 0.04695 | 0.02002 | IFNG/PPARA/STAT3 | 3 |
| GO:0006111 | regulation of gluconeogenesis | Feb-97 | 49/18866 | 0.02631 | 0.04707 | 0.02007 | ADIPOQ/PPARA | 2 |
| GO:0032663 | regulation of interleukin-2 production | Feb-97 | 49/18866 | 0.02631 | 0.04707 | 0.02007 | IL1A/IL1B | 2 |
| GO:0042059 | negative regulation of epidermal growth factor receptor signaling pathway | Feb-97 | 49/18866 | 0.02631 | 0.04707 | 0.02007 | EGF/EGFR | 2 |
| GO:0042149 | cellular response to glucose starvation | Feb-97 | 49/18866 | 0.02631 | 0.04707 | 0.02007 | BCL2/TP53 | 2 |
| GO:0048512 | circadian behavior | Feb-97 | 49/18866 | 0.02631 | 0.04707 | 0.02007 | PTEN/TP53 | 2 |
| GO:0070231 | T cell apoptotic process | Feb-97 | 49/18866 | 0.02631 | 0.04707 | 0.02007 | AKT1/TP53 | 2 |
| GO:0097720 | calcineurin-mediated signaling | Feb-97 | 49/18866 | 0.02631 | 0.04707 | 0.02007 | ERBB3/TNF | 2 |
| GO:0120178 | steroid hormone biosynthetic process | Feb-97 | 49/18866 | 0.02631 | 0.04707 | 0.02007 | CYP19A1/HSD3B1 | 2 |
| GO:1990090 | cellular response to nerve growth factor stimulus | Feb-97 | 49/18866 | 0.02631 | 0.04707 | 0.02007 | AKT1/PTEN | 2 |
| GO:0000075 | cell cycle checkpoint | Apr-97 | 219/18866 | 0.0265 | 0.04738 | 0.0202 | BCL2L1/CDKN1A/TGFB1/TP53 | 4 |
| GO:0001838 | embryonic epithelial tube formation | Mar-97 | 125/18866 | 0.02668 | 0.04768 | 0.02033 | CASP3/CTNNB1/TGFB1 | 3 |
| GO:0002711 | positive regulation of T cell mediated immunity | Feb-97 | 50/18866 | 0.02731 | 0.0487 | 0.02076 | IL1B/IL6 | 2 |
| GO:0007622 | rhythmic behavior | Feb-97 | 50/18866 | 0.02731 | 0.0487 | 0.02076 | PTEN/TP53 | 2 |
| GO:0071715 | icosanoid transport | Feb-97 | 50/18866 | 0.02731 | 0.0487 | 0.02076 | IL1B/NOS2 | 2 |
| GO:1901571 | fatty acid derivative transport | Feb-97 | 50/18866 | 0.02731 | 0.0487 | 0.02076 | IL1B/NOS2 | 2 |
| GO:2000725 | regulation of cardiac muscle cell differentiation | Feb-97 | 50/18866 | 0.02731 | 0.0487 | 0.02076 | PPARA/TGFB1 | 2 |
| GO:0001704 | formation of primary germ layer | Mar-97 | 127/18866 | 0.02778 | 0.04949 | 0.0211 | CTNNB1/MMP2/MMP9 | 3 |
| GO:0017015 | regulation of transforming growth factor beta receptor signaling pathway | Mar-97 | 127/18866 | 0.02778 | 0.04949 | 0.0211 | CAV1/TGFB1/TP53 | 3 |

| **Supplementary file4: Table S5. The detailed information of GO enrichment analysis of cellular component (CC)** | | | | | | | |  |
| --- | --- | --- | --- | --- | --- | --- | --- | --- |
| **ID** | **Description** | **Gene Ratio** | **BgRatio** | **p-value** | **p.adjust** | **qvalue** | **geneID** | **Count** |
| GO:0031983 | vesicle lumen | 14/97 | 328/19559 | 8.43E-10 | 6.99E-08 | 5.09E-08 | ALB/ALOX5/APOB/CAT/EGF/EGFR/GSTP1/LYZ/MAPK1/MPO/SERPINE1/SLPI/TGFB1/VEGFA | 14 |
| GO:0045121 | membrane raft | 14/97 | 329/19559 | 8.77E-10 | 6.99E-08 | 5.09E-08 | CASP3/CAV1/CTNNB1/DPP4/EGFR/HMOX1/ICAM1/IKBKB/MAPK1/MAPK3/NOS3/PTGS2/SELE/TNF | 14 |
| GO:0098857 | membrane microdomain | 14/97 | 330/19559 | 9.12E-10 | 6.99E-08 | 5.09E-08 | CASP3/CAV1/CTNNB1/DPP4/EGFR/HMOX1/ICAM1/IKBKB/MAPK1/MAPK3/NOS3/PTGS2/SELE/TNF | 14 |
| GO:0098589 | membrane region | 14/97 | 343/19559 | 1.50E-09 | 8.64E-08 | 6.29E-08 | CASP3/CAV1/CTNNB1/DPP4/EGFR/HMOX1/ICAM1/IKBKB/MAPK1/MAPK3/NOS3/PTGS2/SELE/TNF | 14 |
| GO:0005901 | caveola | Aug-97 | 82/19559 | 7.19E-09 | 3.31E-07 | 2.41E-07 | CAV1/CTNNB1/HMOX1/MAPK1/MAPK3/NOS3/PTGS2/SELE | 8 |
| GO:0034774 | secretory granule lumen | Dec-97 | 322/19559 | 6.59E-08 | 2.48E-06 | 1.80E-06 | ALB/ALOX5/CAT/EGF/GSTP1/LYZ/MAPK1/MPO/SERPINE1/SLPI/TGFB1/VEGFA | 12 |
| GO:0060205 | cytoplasmic vesicle lumen | Dec-97 | 326/19559 | 7.54E-08 | 2.48E-06 | 1.80E-06 | ALB/ALOX5/CAT/EGF/GSTP1/LYZ/MAPK1/MPO/SERPINE1/SLPI/TGFB1/VEGFA | 12 |
| GO:0044853 | plasma membrane raft | Aug-97 | 113/19559 | 9.11E-08 | 2.62E-06 | 1.91E-06 | CAV1/CTNNB1/HMOX1/MAPK1/MAPK3/NOS3/PTGS2/SELE | 8 |
| GO:0062023 | collagen-containing extracellular matrix | Nov-97 | 427/19559 | 8.71E-06 | 0.00022 | 0.00016 | ACHE/ADIPOQ/F2/F3/F7/ICAM1/MMP2/MMP9/SERPINE1/SLPI/TGFB1 | 11 |
| GO:0090575 | RNA polymerase II transcription regulator complex | Jul-97 | 161/19559 | 1.55E-05 | 0.00036 | 0.00026 | CTNNB1/FOS/JUN/PPARG/RXRA/STAT3/TP53 | 7 |
| GO:0031093 | platelet alpha granule lumen | May-97 | 67/19559 | 2.05E-05 | 0.00043 | 0.00031 | ALB/EGF/SERPINE1/TGFB1/VEGFA | 5 |
| GO:0005788 | endoplasmic reticulum lumen | Sep-97 | 308/19559 | 2.25E-05 | 0.00043 | 0.00031 | ALB/APOB/F10/F2/F7/IGFBP3/IL6/PTGS2/SPP1 | 9 |
| GO:0031091 | platelet alpha granule | May-97 | 91/19559 | 8.98E-05 | 0.00159 | 0.00116 | ALB/EGF/SERPINE1/TGFB1/VEGFA | 5 |
| GO:0005925 | focal adhesion | Sep-97 | 415/19559 | 0.00022 | 0.0035 | 0.00254 | CAT/CAV1/CTNNB1/DPP4/EGFR/HSPB1/ICAM1/MAPK1/MAPK3 | 9 |
| GO:0009897 | external side of plasma membrane | Sep-97 | 417/19559 | 0.00023 | 0.0035 | 0.00254 | CD40LG/F10/F2/F3/GSR/ICAM1/THBD/TNF/VCAM1 | 9 |
| GO:0030055 | cell-substrate junction | Sep-97 | 423/19559 | 0.00025 | 0.00364 | 0.00265 | CAT/CAV1/CTNNB1/DPP4/EGFR/HSPB1/ICAM1/MAPK1/MAPK3 | 9 |
| GO:0101002 | ficolin-1-rich granule | May-97 | 124/19559 | 0.00038 | 0.00488 | 0.00355 | ALOX5/CAT/GSTP1/MAPK1/MMP9 | 5 |
| GO:1904813 | ficolin-1-rich granule lumen | May-97 | 124/19559 | 0.00038 | 0.00488 | 0.00355 | ALOX5/CAT/GSTP1/MAPK1/MMP9 | 5 |
| GO:0005635 | nuclear envelope | Sep-97 | 473/19559 | 0.00057 | 0.00691 | 0.00503 | ALOX5/BCL2/BCL2L1/EGFR/MAPK3/PTGER3/PTGS2/SREBF1/TP53 | 9 |
| GO:0005667 | transcription regulator complex | Aug-97 | 413/19559 | 0.00104 | 0.01148 | 0.00835 | AHR/CTNNB1/FOS/JUN/PPARG/RXRA/STAT3/TP53 | 8 |
| GO:0031970 | organelle envelope lumen | Apr-97 | 90/19559 | 0.00105 | 0.01148 | 0.00835 | ALOX5/CAT/HSD3B1/SOD1 | 4 |
| GO:0045177 | apical part of cell | Aug-97 | 433/19559 | 0.0014 | 0.01466 | 0.01067 | CTNNB1/DPP4/EGFR/ERBB2/ERBB3/PLAT/PTEN/VCAM1 | 8 |
| GO:0043209 | myelin sheath | Mar-97 | 47/19559 | 0.00164 | 0.01637 | 0.01191 | BCL2/ERBB2/PTEN | 3 |
| GO:0005796 | Golgi lumen | Apr-97 | 103/19559 | 0.00173 | 0.01655 | 0.01204 | F10/F2/F7/TGFB1 | 4 |
| GO:0009925 | basal plasma membrane | Mar-97 | 51/19559 | 0.00207 | 0.01907 | 0.01387 | EGFR/ERBB2/ERBB3 | 3 |
| GO:0005769 | early endosome | Jul-97 | 377/19559 | 0.00272 | 0.02406 | 0.01751 | APOB/CAV1/EGFR/ERBB2/MAPK1/MAPK3/VCAM1 | 7 |
| GO:0031143 | pseudopodium | Feb-97 | 18/19559 | 0.00354 | 0.03013 | 0.02192 | MAPK1/MAPK3 | 2 |
| GO:0031965 | nuclear membrane | Jun-97 | 301/19559 | 0.00387 | 0.03182 | 0.02316 | ALOX5/BCL2/BCL2L1/EGFR/PTGS2/TP53 | 6 |
| GO:0071682 | endocytic vesicle lumen | Feb-97 | 20/19559 | 0.00436 | 0.03408 | 0.0248 | APOB/MPO | 2 |
| GO:0030139 | endocytic vesicle | Jun-97 | 313/19559 | 0.00468 | 0.03408 | 0.0248 | APOB/CAV1/DPP4/EGFR/MPO/NOS3 | 6 |
| GO:0005777 | peroxisome | Apr-97 | 137/19559 | 0.00483 | 0.03408 | 0.0248 | CAT/NOS2/SOD1/XDH | 4 |
| GO:0042579 | microbody | Apr-97 | 137/19559 | 0.00483 | 0.03408 | 0.0248 | CAT/NOS2/SOD1/XDH | 4 |
| GO:0045178 | basal part of cell | Mar-97 | 69/19559 | 0.00489 | 0.03408 | 0.0248 | EGFR/ERBB2/ERBB3 | 3 |
| GO:0031233 | intrinsic component of external side of plasma membrane | Feb-97 | 24/19559 | 0.00626 | 0.0416 | 0.03027 | F10/F3 | 2 |
| GO:0072562 | blood microparticle | Apr-97 | 148/19559 | 0.00633 | 0.0416 | 0.03027 | ALB/F2/PON1/TGFB1 | 4 |
| GO:0005758 | mitochondrial intermembrane space | Mar-97 | 80/19559 | 0.00737 | 0.0471 | 0.03428 | CAT/HSD3B1/SOD1 | 3 |
| GO:0034364 | high-density lipoprotein particle | Feb-97 | 27/19559 | 0.00788 | 0.04899 | 0.03565 | APOB/PON1 | 2 |

|  |  | |  |  |  |  |  |  |  |  |  |  |
| --- | --- | --- | --- | --- | --- | --- | --- | --- | --- | --- | --- | --- |
| **Supplementary file4: Table S6. The detailed information of GO enrichment analysis of molecular function (MF)** | | | | | | | | | | |  |  |
| **ID** | | **Description** | | | **Gene Ratio** | **BgRatio** | **p-value** | **p.adjust** | **qvalue** | **geneID** | **Count** | |
| GO:0004879 | | nuclear receptor activity | | | Dec-97 | 52/18352 | 4.08E-17 | 7.14E-15 | 4.34E-15 | AHR/AR/ESR1/NR3C1/NR3C2/PGR/PPARA/PPARD/PPARG/RXRA/SREBF1/STAT3 | 12 |  |
| GO:0098531 | | ligand-activated transcription factor activity | | | Dec-97 | 52/18352 | 4.08E-17 | 7.14E-15 | 4.34E-15 | AHR/AR/ESR1/NR3C1/NR3C2/PGR/PPARA/PPARD/PPARG/RXRA/SREBF1/STAT3 | 12 |  |
| GO:0005125 | | cytokine activity | | | 15/97 | 235/18352 | 1.59E-12 | 1.86E-10 | 1.13E-10 | ADIPOQ/CCL2/CD40LG/CXCL8/IFNG/IL10/IL1A/IL1B/IL2/IL4/IL6/SPP1/TGFB1/TNF/VEGFA | 15 |  |
| GO:0005126 | | cytokine receptor binding | | | 15/97 | 271/18352 | 1.23E-11 | 1.08E-09 | 6.55E-10 | CASP3/CCL2/CD40LG/CXCL8/IFNG/IL10/IL1A/IL1B/IL2/IL4/IL6/STAT3/TGFB1/TNF/VEGFA | 15 |  |
| GO:0003707 | | steroid hormone receptor activity | | | Jul-97 | 26/18352 | 5.60E-11 | 3.92E-09 | 2.38E-09 | ESR1/NR3C1/NR3C2/PGR/PPARA/PPARD/RXRA | 7 |  |
| GO:0048018 | | receptor ligand activity | | | 18/97 | 487/18352 | 7.57E-11 | 4.41E-09 | 2.68E-09 | ADIPOQ/CCL2/CD40LG/CXCL8/DPP4/EGF/F2/IFNG/IL10/IL1A/IL1B/IL2/IL4/IL6/SPP1/TGFB1/TNF/VEGFA | 18 |  |
| GO:0030546 | | signaling receptor activator activity | | | 18/97 | 492/18352 | 8.94E-11 | 4.47E-09 | 2.71E-09 | ADIPOQ/CCL2/CD40LG/CXCL8/DPP4/EGF/F2/IFNG/IL10/IL1A/IL1B/IL2/IL4/IL6/SPP1/TGFB1/TNF/VEGFA | 18 |  |
| GO:0019902 | | phosphatase binding | | | Dec-97 | 194/18352 | 4.50E-10 | 1.97E-08 | 1.20E-08 | AKT1/BCL2/CTNNB1/EGFR/ERBB2/MAPK1/MAPK3/PPARA/PPARG/SOD1/STAT3/TP53 | 12 |  |
| GO:0020037 | | heme binding | | | Oct-97 | 138/18352 | 3.00E-09 | 1.17E-07 | 7.09E-08 | CAT/CYP19A1/CYP1A1/CYP1A2/CYP3A4/HMOX1/MPO/NOS2/NOS3/PTGS2 | 10 |  |
| GO:0017171 | | serine hydrolase activity | | | Nov-97 | 191/18352 | 5.31E-09 | 1.86E-07 | 1.13E-07 | ACHE/DPP4/F10/F2/F3/F7/MMP1/MMP2/MMP3/MMP9/PLAT | 11 |  |
| GO:0046906 | | tetrapyrrole binding | | | Oct-97 | 148/18352 | 5.92E-09 | 1.88E-07 | 1.14E-07 | CAT/CYP19A1/CYP1A1/CYP1A2/CYP3A4/HMOX1/MPO/NOS2/NOS3/PTGS2 | 10 |  |
| GO:0001223 | | transcription coactivator binding | | | Jun-97 | 29/18352 | 8.03E-09 | 2.34E-07 | 1.42E-07 | AHR/AR/ESR1/PGR/PPARA/PPARD | 6 |  |
| GO:0004252 | | serine-type endopeptidase activity | | | Oct-97 | 169/18352 | 2.12E-08 | 5.70E-07 | 3.47E-07 | DPP4/F10/F2/F3/F7/MMP1/MMP2/MMP3/MMP9/PLAT | 10 |  |
| GO:0140297 | | DNA-binding transcription factor binding | | | 13/97 | 347/18352 | 3.52E-08 | 8.81E-07 | 5.35E-07 | BCL2/CTNNB1/ESR1/FOS/HSPB1/JUN/MYC/NFKBIA/PPARA/PPARD/PPARG/RXRA/STAT3 | 13 |  |
| GO:0008236 | | serine-type peptidase activity | | | Oct-97 | 187/18352 | 5.54E-08 | 1.27E-06 | 7.73E-07 | DPP4/F10/F2/F3/F7/MMP1/MMP2/MMP3/MMP9/PLAT | 10 |  |
| GO:0070851 | | growth factor receptor binding | | | Sep-97 | 141/18352 | 5.81E-08 | 1.27E-06 | 7.73E-07 | EGF/IL10/IL1A/IL1B/IL2/IL4/IL6/PTEN/VEGFA | 9 |  |
| GO:0019903 | | protein phosphatase binding | | | Sep-97 | 149/18352 | 9.36E-08 | 1.93E-06 | 1.17E-06 | AKT1/BCL2/CTNNB1/EGFR/ERBB2/PPARG/SOD1/STAT3/TP53 | 9 |  |
| GO:0070491 | | repressing transcription factor binding | | | Jul-97 | 74/18352 | 1.25E-07 | 2.43E-06 | 1.47E-06 | BCL2/CTNNB1/MYC/PPARA/PPARD/PPARG/STAT3 | 7 |  |
| GO:0061629 | | RNA polymerase II-specific DNA-binding transcription factor binding | | | Nov-97 | 267/18352 | 1.66E-07 | 3.05E-06 | 1.85E-06 | CTNNB1/ESR1/FOS/HSPB1/JUN/NFKBIA/PPARA/PPARD/PPARG/RXRA/STAT3 | 11 |  |
| GO:0016705 | | oxidoreductase activity, acting on paired donors, with incorporation or reduction of molecular oxygen | | | Sep-97 | 162/18352 | 1.92E-07 | 3.35E-06 | 2.04E-06 | CYP19A1/CYP1A1/CYP1A2/CYP3A4/HMOX1/NOS2/NOS3/PTGS2/TYR | 9 |  |
| GO:0001221 | | transcription cofactor binding | | | Jun-97 | 51/18352 | 2.77E-07 | 4.62E-06 | 2.81E-06 | AHR/AR/ESR1/PGR/PPARA/PPARD | 6 |  |
| GO:0016209 | | antioxidant activity | | | Jul-97 | 86/18352 | 3.54E-07 | 5.63E-06 | 3.42E-06 | ALB/CAT/GSR/GSTP1/MPO/PTGS2/SOD1 | 7 |  |
| GO:0002020 | | protease binding | | | Aug-97 | 137/18352 | 6.49E-07 | 9.88E-06 | 6.00E-06 | BCL2/CASP3/DPP4/F3/PTEN/SERPINE1/TNF/TP53 | 8 |  |
| GO:0004497 | | monooxygenase activity | | | Jul-97 | 101/18352 | 1.06E-06 | 1.55E-05 | 9.41E-06 | CYP19A1/CYP1A1/CYP1A2/CYP3A4/NOS2/NOS3/TYR | 7 |  |
| GO:0005496 | | steroid binding | | | Jul-97 | 106/18352 | 1.47E-06 | 2.06E-05 | 1.25E-05 | AR/CAV1/CYP3A4/ESR1/NR3C1/NR3C2/PGR | 7 |  |
| GO:0031406 | | carboxylic acid binding | | | Sep-97 | 212/18352 | 1.83E-06 | 2.47E-05 | 1.50E-05 | ADIPOQ/ALB/GSTP1/NOS2/NOS3/PPARD/PPARG/RXRA/SELE | 9 |  |
| GO:0008083 | | growth factor activity | | | Aug-97 | 162/18352 | 2.30E-06 | 2.98E-05 | 1.81E-05 | EGF/F2/IL10/IL2/IL4/IL6/TGFB1/VEGFA | 8 |  |
| GO:0001091 | | RNA polymerase II general transcription initiation factor binding | | | Apr-97 | 19/18352 | 2.67E-06 | 3.34E-05 | 2.03E-05 | AHR/AR/ESR1/TP53 | 4 |  |
| GO:0043177 | | organic acid binding | | | Sep-97 | 224/18352 | 2.88E-06 | 3.48E-05 | 2.12E-05 | ADIPOQ/ALB/GSTP1/NOS2/NOS3/PPARD/PPARG/RXRA/SELE | 9 |  |
| GO:0070330 | | aromatase activity | | | Apr-97 | 25/18352 | 8.52E-06 | 9.94E-05 | 6.04E-05 | CYP19A1/CYP1A1/CYP1A2/CYP3A4 | 4 |  |
| GO:0005507 | | copper ion binding | | | May-97 | 60/18352 | 1.61E-05 | 0.00018 | 0.00011 | ALB/IL1A/SOD1/TP53/TYR | 5 |  |
| GO:0016725 | | oxidoreductase activity, acting on CH or CH2 groups | | | Mar-97 | 10/18352 | 1.67E-05 | 0.00018 | 0.00011 | CYP1A2/CYP3A4/XDH | 3 |  |
| GO:0004175 | | endopeptidase activity | | | Nov-97 | 440/18352 | 2.07E-05 | 0.00022 | 0.00013 | CASP3/DPP4/F10/F2/F3/F7/MMP1/MMP2/MMP3/MMP9/PLAT | 11 |  |
| GO:0001103 | | RNA polymerase II repressing transcription factor binding | | | Apr-97 | 35/18352 | 3.39E-05 | 0.00034 | 0.00021 | PPARA/PPARD/PPARG/STAT3 | 4 |  |
| GO:0016712 | | oxidoreductase activity, acting on paired donors, with incorporation or reduction of molecular oxygen, reduced flavin or flavoprotein as one donor, and incorporation of one atom of oxygen | | | Apr-97 | 35/18352 | 3.39E-05 | 0.00034 | 0.00021 | CYP19A1/CYP1A1/CYP1A2/CYP3A4 | 4 |  |
| GO:0019825 | | oxygen binding | | | Apr-97 | 36/18352 | 3.79E-05 | 0.00037 | 0.00022 | ALB/CYP19A1/CYP1A1/CYP3A4 | 4 |  |
| GO:0033293 | | monocarboxylic acid binding | | | May-97 | 72/18352 | 3.93E-05 | 0.00037 | 0.00023 | ALB/GSTP1/PPARD/PPARG/RXRA | 5 |  |
| GO:0008395 | | steroid hydroxylase activity | | | Apr-97 | 38/18352 | 4.72E-05 | 0.00043 | 0.00026 | CYP19A1/CYP1A1/CYP1A2/CYP3A4 | 4 |  |
| GO:0005504 | | fatty acid binding | | | Apr-97 | 39/18352 | 5.23E-05 | 0.00047 | 0.00029 | ALB/GSTP1/PPARD/PPARG | 4 |  |
| GO:0033613 | | activating transcription factor binding | | | May-97 | 80/18352 | 6.53E-05 | 0.00057 | 0.00035 | CTNNB1/FOS/JUN/MYC/PPARG | 5 |  |
| GO:0140296 | | general transcription initiation factor binding | | | Apr-97 | 44/18352 | 8.47E-05 | 0.00072 | 0.00044 | AHR/AR/ESR1/TP53 | 4 |  |
| GO:0016709 | | oxidoreductase activity, acting on paired donors, with incorporation or reduction of molecular oxygen, NAD(P)H as one donor, and incorporation of one atom of oxygen | | | Apr-97 | 45/18352 | 9.25E-05 | 0.00077 | 0.00047 | CYP1A1/CYP3A4/NOS2/NOS3 | 4 |  |
| GO:0001046 | | core promoter sequence-specific DNA binding | | | Apr-97 | 46/18352 | 0.0001 | 0.00082 | 0.0005 | FOS/MYC/NR3C1/TP53 | 4 |  |
| GO:0051117 | | ATPase binding | | | May-97 | 88/18352 | 0.0001 | 0.00082 | 0.0005 | AR/CAV1/EGFR/ESR1/PGR | 5 |  |
| GO:0005178 | | integrin binding | | | Jun-97 | 144/18352 | 0.00012 | 0.0009 | 0.00055 | CD40LG/EGFR/ICAM1/IL1B/SPP1/VCAM1 | 6 |  |
| GO:0019207 | | kinase regulator activity | | | Jul-97 | 216/18352 | 0.00015 | 0.00111 | 0.00068 | CASP3/CDKN1A/EGF/ERBB3/GSTP1/HSPB1/IL2 | 7 |  |
| GO:0005506 | | iron ion binding | | | Jun-97 | 151/18352 | 0.00015 | 0.00111 | 0.00068 | ALOX5/CYP19A1/CYP1A1/CYP1A2/CYP3A4/XDH | 6 |  |
| GO:0004707 | | MAP kinase activity | | | Mar-97 | 20/18352 | 0.00015 | 0.00111 | 0.00068 | MAPK1/MAPK3/MAPK8 | 3 |  |
| GO:0004601 | | peroxidase activity | | | Apr-97 | 52/18352 | 0.00016 | 0.00117 | 0.00071 | CAT/GSTP1/MPO/PTGS2 | 4 |  |
| GO:0050661 | | NADP binding | | | Apr-97 | 54/18352 | 0.00019 | 0.00133 | 0.00081 | CAT/GSR/NOS2/NOS3 | 4 |  |
| GO:0016922 | | nuclear receptor binding | | | May-97 | 101/18352 | 0.0002 | 0.00135 | 0.00082 | CTNNB1/ESR1/PPARG/RXRA/STAT3 | 5 |  |
| GO:0016684 | | oxidoreductase activity, acting on peroxide as acceptor | | | Apr-97 | 56/18352 | 0.00022 | 0.00147 | 0.00089 | CAT/GSTP1/MPO/PTGS2 | 4 |  |
| GO:0008144 | | drug binding | | | May-97 | 104/18352 | 0.00023 | 0.00149 | 0.00091 | ALB/GSTP1/PPARA/PPARD/PPARG | 5 |  |
| GO:0046982 | | protein heterodimerization activity | | | Aug-97 | 321/18352 | 0.0003 | 0.00195 | 0.00118 | AHR/BCL2/BCL2L1/CAV1/ERBB2/ERBB3/IKBKB/TP53 | 8 |  |
| GO:0001098 | | basal transcription machinery binding | | | Apr-97 | 72/18352 | 0.00057 | 0.00355 | 0.00216 | AHR/AR/ESR1/TP53 | 4 |  |
| GO:0001099 | | basal RNA polymerase II transcription machinery binding | | | Apr-97 | 72/18352 | 0.00057 | 0.00355 | 0.00216 | AHR/AR/ESR1/TP53 | 4 |  |
| GO:0030291 | | protein serine/threonine kinase inhibitor activity | | | Mar-97 | 31/18352 | 0.00058 | 0.00355 | 0.00216 | CASP3/CDKN1A/HSPB1 | 3 |  |
| GO:0051721 | | protein phosphatase 2A binding | | | Mar-97 | 32/18352 | 0.00064 | 0.00383 | 0.00233 | AKT1/BCL2/TP53 | 3 |  |
| GO:0035258 | | steroid hormone receptor binding | | | Apr-97 | 81/18352 | 0.00089 | 0.00521 | 0.00317 | CTNNB1/ESR1/PPARG/STAT3 | 4 |  |
| GO:0050660 | | flavin adenine dinucleotide binding | | | Apr-97 | 81/18352 | 0.00089 | 0.00521 | 0.00317 | GSR/NOS2/NOS3/XDH | 4 |  |
| GO:0035257 | | nuclear hormone receptor binding | | | May-97 | 144/18352 | 0.001 | 0.00567 | 0.00345 | CTNNB1/ESR1/PPARG/RXRA/STAT3 | 5 |  |
| GO:0031625 | | ubiquitin protein ligase binding | | | Jul-97 | 297/18352 | 0.001 | 0.00567 | 0.00345 | BCL2/CDKN1A/EGFR/ERBB3/JUN/NFKBIA/TP53 | 7 |  |
| GO:0001094 | | TFIID-class transcription factor complex binding | | | Feb-97 | 10/18352 | 0.00121 | 0.00662 | 0.00402 | AHR/TP53 | 2 |  |
| GO:0004955 | | prostaglandin receptor activity | | | Feb-97 | 10/18352 | 0.00121 | 0.00662 | 0.00402 | PPARG/PTGER3 | 2 |  |
| GO:0042277 | | peptide binding | | | Jul-97 | 308/18352 | 0.00124 | 0.00668 | 0.00406 | ACHE/CAT/GSTM1/GSTP1/NFKBIA/PPARG/RXRA | 7 |  |
| GO:0019209 | | kinase activator activity | | | Apr-97 | 89/18352 | 0.00127 | 0.00673 | 0.00409 | CDKN1A/EGF/ERBB3/IL2 | 4 |  |
| GO:0030331 | | estrogen receptor binding | | | Mar-97 | 42/18352 | 0.00142 | 0.00717 | 0.00435 | CTNNB1/ESR1/PPARG | 3 |  |
| GO:0051879 | | Hsp90 protein binding | | | Mar-97 | 42/18352 | 0.00142 | 0.00717 | 0.00435 | AHR/CYP1A1/NR3C1 | 3 |  |
| GO:0044389 | | ubiquitin-like protein ligase binding | | | Jul-97 | 316/18352 | 0.00144 | 0.00717 | 0.00435 | BCL2/CDKN1A/EGFR/ERBB3/JUN/NFKBIA/TP53 | 7 |  |
| GO:0004954 | | prostanoid receptor activity | | | Feb-97 | 11/18352 | 0.00147 | 0.00717 | 0.00435 | PPARG/PTGER3 | 2 |  |
| GO:0051400 | | BH domain binding | | | Feb-97 | 11/18352 | 0.00147 | 0.00717 | 0.00435 | BCL2/BCL2L1 | 2 |  |
| GO:0070513 | | death domain binding | | | Feb-97 | 11/18352 | 0.00147 | 0.00717 | 0.00435 | BCL2/BCL2L1 | 2 |  |
| GO:0004712 | | protein serine/threonine/tyrosine kinase activity | | | Mar-97 | 45/18352 | 0.00173 | 0.00823 | 0.005 | AKT1/MAPK1/MAPK3 | 3 |  |
| GO:0004861 | | cyclin-dependent protein serine/threonine kinase inhibitor activity | | | Feb-97 | 12/18352 | 0.00176 | 0.00823 | 0.005 | CASP3/CDKN1A | 2 |  |
| GO:0043295 | | glutathione binding | | | Feb-97 | 12/18352 | 0.00176 | 0.00823 | 0.005 | GSTM1/GSTP1 | 2 |  |
| GO:0001102 | | RNA polymerase II activating transcription factor binding | | | Mar-97 | 47/18352 | 0.00196 | 0.00903 | 0.00549 | CTNNB1/FOS/JUN | 3 |  |
| GO:0016653 | | oxidoreductase activity, acting on NAD(P)H, heme protein as acceptor | | | Feb-97 | 13/18352 | 0.00208 | 0.00923 | 0.00561 | NOS2/NOS3 | 2 |  |
| GO:1900750 | | oligopeptide binding | | | Feb-97 | 13/18352 | 0.00208 | 0.00923 | 0.00561 | GSTM1/GSTP1 | 2 |  |
| GO:0032813 | | tumor necrosis factor receptor superfamily binding | | | Mar-97 | 48/18352 | 0.00208 | 0.00923 | 0.00561 | CASP3/CD40LG/TNF | 3 |  |
| GO:0004674 | | protein serine/threonine kinase activity | | | Aug-97 | 435/18352 | 0.00215 | 0.00939 | 0.0057 | AKT1/EGFR/IKBKB/MAPK1/MAPK3/MAPK8/PIK3CG/PIM1 | 8 |  |
| GO:0051087 | | chaperone binding | | | Apr-97 | 104/18352 | 0.00225 | 0.00973 | 0.00591 | ALB/BIRC5/SOD1/TP53 | 4 |  |
| GO:0070888 | | E-box binding | | | Mar-97 | 50/18352 | 0.00234 | 0.01 | 0.00608 | AHR/MYC/PPARG | 3 |  |
| GO:0051427 | | hormone receptor binding | | | May-97 | 177/18352 | 0.00249 | 0.01048 | 0.00637 | CTNNB1/ESR1/PPARG/RXRA/STAT3 | 5 |  |
| GO:0004222 | | metalloendopeptidase activity | | | Apr-97 | 108/18352 | 0.00258 | 0.01076 | 0.00654 | MMP1/MMP2/MMP3/MMP9 | 4 |  |
| GO:1901681 | | sulfur compound binding | | | Jun-97 | 262/18352 | 0.00267 | 0.01101 | 0.00669 | APOB/F2/GSTM1/GSTP1/MPO/VEGFA | 6 |  |
| GO:0004953 | | icosanoid receptor activity | | | Feb-97 | 15/18352 | 0.00278 | 0.01104 | 0.00671 | PPARG/PTGER3 | 2 |  |
| GO:0005161 | | platelet-derived growth factor receptor binding | | | Feb-97 | 15/18352 | 0.00278 | 0.01104 | 0.00671 | PTEN/VEGFA | 2 |  |
| GO:0036041 | | long-chain fatty acid binding | | | Feb-97 | 15/18352 | 0.00278 | 0.01104 | 0.00671 | PPARD/PPARG | 2 |  |
| GO:0009055 | | electron transfer activity | | | Apr-97 | 111/18352 | 0.00285 | 0.01122 | 0.00681 | CYP19A1/CYP1A2/GSR/XDH | 4 |  |
| GO:0019887 | | protein kinase regulator activity | | | May-97 | 185/18352 | 0.00301 | 0.0117 | 0.00711 | CASP3/CDKN1A/EGF/ERBB3/HSPB1 | 5 |  |
| GO:0005149 | | interleukin-1 receptor binding | | | Feb-97 | 16/18352 | 0.00316 | 0.01203 | 0.00731 | IL1A/IL1B | 2 |  |
| GO:0010181 | | FMN binding | | | Feb-97 | 16/18352 | 0.00316 | 0.01203 | 0.00731 | NOS2/NOS3 | 2 |  |
| GO:0001085 | | RNA polymerase II transcription factor binding | | | Mar-97 | 56/18352 | 0.00324 | 0.01219 | 0.00741 | AR/CTNNB1/TP53 | 3 |  |
| GO:0043621 | | protein self-association | | | Mar-97 | 57/18352 | 0.00341 | 0.01255 | 0.00762 | ACHE/PPARG/TP53 | 3 |  |
| GO:0050840 | | extracellular matrix binding | | | Mar-97 | 57/18352 | 0.00341 | 0.01255 | 0.00762 | ACHE/SPP1/VEGFA | 3 |  |
| GO:0097110 | | scaffold protein binding | | | Mar-97 | 60/18352 | 0.00394 | 0.01436 | 0.00872 | IKBKB/MAPK3/NOS3 | 3 |  |
| GO:0004708 | | MAP kinase kinase activity | | | Feb-97 | 18/18352 | 0.004 | 0.01445 | 0.00878 | MAPK1/MAPK3 | 2 |  |
| GO:0033218 | | amide binding | | | Jul-97 | 381/18352 | 0.00408 | 0.01458 | 0.00886 | ACHE/CAT/GSTM1/GSTP1/NFKBIA/PPARG/RXRA | 7 |  |
| GO:0004714 | | transmembrane receptor protein tyrosine kinase activity | | | Mar-97 | 61/18352 | 0.00413 | 0.01459 | 0.00886 | EGFR/ERBB2/ERBB3 | 3 |  |
| GO:0030296 | | protein tyrosine kinase activator activity | | | Feb-97 | 19/18352 | 0.00446 | 0.01561 | 0.00948 | EGF/ERBB3 | 2 |  |
| GO:0004860 | | protein kinase inhibitor activity | | | Mar-97 | 65/18352 | 0.00493 | 0.01709 | 0.01039 | CASP3/CDKN1A/HSPB1 | 3 |  |
| GO:0050750 | | low-density lipoprotein particle receptor binding | | | Feb-97 | 21/18352 | 0.00544 | 0.01866 | 0.01134 | APOB/CRP | 2 |  |
| GO:0019210 | | kinase inhibitor activity | | | Mar-97 | 69/18352 | 0.00583 | 0.01977 | 0.01201 | CASP3/CDKN1A/HSPB1 | 3 |  |
| GO:0019838 | | growth factor binding | | | Apr-97 | 136/18352 | 0.00588 | 0.01977 | 0.01201 | EGFR/ERBB2/ERBB3/IGFBP3 | 4 |  |
| GO:0033691 | | sialic acid binding | | | Feb-97 | 22/18352 | 0.00596 | 0.01987 | 0.01207 | ADIPOQ/SELE | 2 |  |
| GO:0042379 | | chemokine receptor binding | | | Mar-97 | 70/18352 | 0.00607 | 0.02003 | 0.01217 | CCL2/CXCL8/STAT3 | 3 |  |
| GO:0070412 | | R-SMAD binding | | | Feb-97 | 23/18352 | 0.00651 | 0.02129 | 0.01293 | FOS/JUN | 2 |  |
| GO:0001618 | | virus receptor activity | | | Mar-97 | 74/18352 | 0.00708 | 0.02272 | 0.0138 | DPP4/EGFR/ICAM1 | 3 |  |
| GO:0140272 | | exogenous protein binding | | | Mar-97 | 74/18352 | 0.00708 | 0.02272 | 0.0138 | DPP4/EGFR/ICAM1 | 3 |  |
| GO:0004364 | | glutathione transferase activity | | | Feb-97 | 25/18352 | 0.00766 | 0.02416 | 0.01468 | GSTM1/GSTP1 | 2 |  |
| GO:0016702 | | oxidoreductase activity, acting on single donors with incorporation of molecular oxygen, incorporation of two atoms of oxygen | | | Feb-97 | 25/18352 | 0.00766 | 0.02416 | 0.01468 | ALOX5/PTGS2 | 2 |  |
| GO:0016701 | | oxidoreductase activity, acting on single donors with incorporation of molecular oxygen | | | Feb-97 | 26/18352 | 0.00827 | 0.0254 | 0.01543 | ALOX5/PTGS2 | 2 |  |
| GO:0017025 | | TBP-class protein binding | | | Feb-97 | 26/18352 | 0.00827 | 0.0254 | 0.01543 | AHR/ESR1 | 2 |  |
| GO:0070325 | | lipoprotein particle receptor binding | | | Feb-97 | 26/18352 | 0.00827 | 0.0254 | 0.01543 | APOB/CRP | 2 |  |
| GO:0046332 | | SMAD binding | | | Mar-97 | 79/18352 | 0.00847 | 0.02578 | 0.01566 | CTNNB1/FOS/JUN | 3 |  |
| GO:0001227 | | DNA-binding transcription repressor activity, RNA polymerase II-specific | | | Jun-97 | 335/18352 | 0.00871 | 0.02619 | 0.01591 | JUN/MYC/NR3C1/PPARA/PPARD/PPARG | 6 |  |
| GO:0019199 | | transmembrane receptor protein kinase activity | | | Mar-97 | 80/18352 | 0.00877 | 0.02619 | 0.01591 | EGFR/ERBB2/ERBB3 | 3 |  |
| GO:0001217 | | DNA-binding transcription repressor activity | | | Jun-97 | 336/18352 | 0.00884 | 0.02619 | 0.01591 | JUN/MYC/NR3C1/PPARA/PPARD/PPARG | 6 |  |
| GO:0001968 | | fibronectin binding | | | Feb-97 | 27/18352 | 0.00891 | 0.02619 | 0.01591 | IGFBP3/VEGFA | 2 |  |
| GO:0030295 | | protein kinase activator activity | | | Mar-97 | 82/18352 | 0.00938 | 0.02735 | 0.01662 | CDKN1A/EGF/ERBB3 | 3 |  |
| GO:0042562 | | hormone binding | | | Mar-97 | 84/18352 | 0.01001 | 0.02896 | 0.0176 | AR/EGFR/NR3C1 | 3 |  |
| GO:0008013 | | beta-catenin binding | | | Mar-97 | 85/18352 | 0.01034 | 0.02942 | 0.01788 | AR/CTNNB1/ESR1 | 3 |  |
| GO:0051219 | | phosphoprotein binding | | | Mar-97 | 85/18352 | 0.01034 | 0.02942 | 0.01788 | MAPK1/MAPK3/PLAT | 3 |  |
| GO:0005164 | | tumor necrosis factor receptor binding | | | Feb-97 | 31/18352 | 0.01164 | 0.03258 | 0.0198 | CD40LG/TNF | 2 |  |
| GO:0051059 | | NF-kappaB binding | | | Feb-97 | 31/18352 | 0.01164 | 0.03258 | 0.0198 | NFKBIA/PPARD | 2 |  |
| GO:0008201 | | heparin binding | | | Apr-97 | 169/18352 | 0.01239 | 0.03442 | 0.02091 | APOB/F2/MPO/VEGFA | 4 |  |
| GO:0005501 | | retinoid binding | | | Feb-97 | 36/18352 | 0.0155 | 0.04238 | 0.02575 | RHO/RXRA | 2 |  |
| GO:0097718 | | disordered domain specific binding | | | Feb-97 | 36/18352 | 0.0155 | 0.04238 | 0.02575 | CTNNB1/TP53 | 2 |  |
| GO:0004857 | | enzyme inhibitor activity | | | Jun-97 | 383/18352 | 0.01602 | 0.04346 | 0.0264 | BIRC5/CASP3/CDKN1A/HSPB1/SERPINE1/SLPI | 6 |  |
| GO:0019840 | | isoprenoid binding | | | Feb-97 | 37/18352 | 0.01633 | 0.04396 | 0.02671 | RHO/RXRA | 2 |  |
| GO:0032451 | | demethylase activity | | | Feb-97 | 38/18352 | 0.01718 | 0.0459 | 0.02788 | CYP1A1/CYP1A2 | 2 |  |
| GO:0008237 | | metallopeptidase activity | | | Apr-97 | 189/18352 | 0.01798 | 0.04749 | 0.02885 | MMP1/MMP2/MMP3/MMP9 | 4 |  |
| GO:0016248 | | channel inhibitor activity | | | Feb-97 | 39/18352 | 0.01805 | 0.04749 | 0.02885 | BCL2/CAV1 | 2 |  |
