# Supplementary material for: Network Pharmacology-Based Investigation on the Mechanism of the JinGuanLan Formula in Treating Acne Vulgaris
Source: Evid Based Complement Alternat Med. 2022 Jul 13;2022:6944792. doi: 10.1155/2022/6944792 (PMC9300327; doi:10.1155/2022/6944792)
Supplement: Supplementary Materials — Supplementary file 1, Tables S1 and S2: the basic information of all active compounds and related targets. Supplementary file 2, Table S3; and Supplementary file 3, Figure S1: the detailed information of the potential target genes of acne vulgaris. Supplementary file 4, Tables 4, S5, and S6: the detailed information of GO enrichment analysis for BP, CC, and MF. Supplementary file 5, Table S7: the detailed information of screened KEGG pathways. [file 6944792.f1.zip › 6944792.f1/Supplementary file5, Table S7 The detailed information of screened KEGG pathways..docx]

| **Supplementary file5 : Table S7. KEGG enriched pathways** | | | | | | | | |  |
| --- | --- | --- | --- | --- | --- | --- | --- | --- | --- |
| **ID** | **Description** | **GeneRatio** | **BgRatio** | **pvalue** | **p.adjust** | **qvalue** | **gene Name** | **Count** | |
| hsa04933 | AGE-RAGE signaling pathway in diabetic complications | 25/93 | 100/8081 | 1.31E-27 | 2.90E-25 | 8.54E-26 | AKT1/BCL2/CASP3/CCL2/CXCL8/F3/ICAM1/IL1A/IL1B/IL6/JUN/MAPK1/MAPK3/MAPK8/MMP2/NOS3/PIM1/SELE/SERPINE1/STAT3/TGFB1/THBD/TNF/VCAM1/VEGFA | | 25 |
| hsa05418 | Fluid shear stress and atherosclerosis | 26/93 | 139/8081 | 3.60E-25 | 4.00E-23 | 1.18E-23 | AKT1/BCL2/CAV1/CCL2/CTNNB1/FOS/GSTM1/GSTP1/HMOX1/ICAM1/IFNG/IKBKB/IL1A/IL1B/JUN/MAPK8/MMP2/MMP9/NOS3/PLAT/SELE/THBD/TNF/TP53/VCAM1/VEGFA | | 26 |
| hsa04657 | IL-17 signaling pathway | 19/93 | 94/8081 | 3.81E-19 | 2.82E-17 | 8.30E-18 | CASP3/CCL2/CXCL8/FOS/IFNG/IKBKB/IL1B/IL4/IL6/JUN/MAPK1/MAPK3/MAPK8/MMP1/MMP3/MMP9/NFKBIA/PTGS2/TNF | | 19 |
| hsa05142 | Chagas disease | 19/93 | 102/8081 | 1.98E-18 | 1.10E-16 | 3.22E-17 | AKT1/CCL2/CXCL8/FOS/IFNG/IKBKB/IL10/IL1B/IL2/IL6/JUN/MAPK1/MAPK3/MAPK8/NFKBIA/NOS2/SERPINE1/TGFB1/TNF | | 19 |
| hsa04668 | TNF signaling pathway | 19/93 | 112/8081 | 1.27E-17 | 5.63E-16 | 1.65E-16 | AKT1/CASP3/CCL2/FOS/ICAM1/IKBKB/IL1B/IL6/JUN/MAPK1/MAPK3/MAPK8/MMP3/MMP9/NFKBIA/PTGS2/SELE/TNF/VCAM1 | | 19 |
| hsa05215 | Prostate cancer | 18/93 | 97/8081 | 1.83E-17 | 6.78E-16 | 1.99E-16 | AKT1/AR/BCL2/CDKN1A/CTNNB1/EGF/EGFR/ERBB2/GSTP1/IKBKB/MAPK1/MAPK3/MMP3/MMP9/NFKBIA/PLAT/PTEN/TP53 | | 18 |
| hsa05145 | Toxoplasmosis | 18/93 | 112/8081 | 2.71E-16 | 8.58E-15 | 2.52E-15 | AKT1/ALOX5/BCL2/BCL2L1/CASP3/CD40LG/IFNG/IKBKB/IL10/MAPK1/MAPK3/MAPK8/NFKBIA/NOS2/PIK3CG/STAT3/TGFB1/TNF | | 18 |
| hsa05219 | Bladder cancer | 13/93 | 41/8081 | 3.54E-16 | 9.83E-15 | 2.89E-15 | CDKN1A/CXCL8/EGF/EGFR/ERBB2/MAPK1/MAPK3/MMP1/MMP2/MMP9/MYC/TP53/VEGFA | | 13 |
| hsa05161 | Hepatitis B | 20/93 | 162/8081 | 1.02E-15 | 2.52E-14 | 7.40E-15 | AKT1/BCL2/BIRC5/CASP3/CDKN1A/CXCL8/FOS/IKBKB/IL6/JUN/MAPK1/MAPK3/MAPK8/MMP9/MYC/NFKBIA/STAT3/TGFB1/TNF/TP53 | | 20 |
| hsa05210 | Colorectal cancer | 16/93 | 86/8081 | 1.25E-15 | 2.78E-14 | 8.19E-15 | AKT1/BCL2/BIRC5/CASP3/CDKN1A/CTNNB1/EGF/EGFR/FOS/JUN/MAPK1/MAPK3/MAPK8/MYC/TGFB1/TP53 | | 16 |
| hsa05167 | Kaposi sarcoma-associated herpesvirus infection | 20/93 | 193/8081 | 3.16E-14 | 6.38E-13 | 1.88E-13 | AKT1/CASP3/CDKN1A/CTNNB1/CXCL8/FOS/ICAM1/IKBKB/IL6/JUN/MAPK1/MAPK3/MAPK8/MYC/NFKBIA/PIK3CG/PTGS2/STAT3/TP53/VEGFA | | 20 |
| hsa04659 | Th17 cell differentiation | 16/93 | 107/8081 | 4.65E-14 | 8.60E-13 | 2.53E-13 | AHR/FOS/IFNG/IKBKB/IL1B/IL2/IL4/IL6/JUN/MAPK1/MAPK3/MAPK8/NFKBIA/RXRA/STAT3/TGFB1 | | 16 |
| hsa04066 | HIF-1 signaling pathway | 16/93 | 109/8081 | 6.28E-14 | 1.07E-12 | 3.15E-13 | AKT1/BCL2/CDKN1A/EGF/EGFR/ERBB2/HMOX1/IFNG/IL6/MAPK1/MAPK3/NOS2/NOS3/SERPINE1/STAT3/VEGFA | | 16 |
| hsa05212 | Pancreatic cancer | 14/93 | 76/8081 | 1.00E-13 | 1.59E-12 | 4.68E-13 | AKT1/BCL2L1/CDKN1A/EGF/EGFR/ERBB2/IKBKB/MAPK1/MAPK3/MAPK8/STAT3/TGFB1/TP53/VEGFA | | 14 |
| hsa05140 | Leishmaniasis | 14/93 | 77/8081 | 1.21E-13 | 1.80E-12 | 5.28E-13 | FOS/IFNG/IL10/IL1A/IL1B/IL4/JUN/MAPK1/MAPK3/NFKBIA/NOS2/PTGS2/TGFB1/TNF | | 14 |
| hsa05144 | Malaria | Dec-93 | 50/8081 | 2.21E-13 | 3.07E-12 | 9.02E-13 | CCL2/CD40LG/CXCL8/ICAM1/IFNG/IL10/IL1B/IL6/SELE/TGFB1/TNF/VCAM1 | | 12 |
| hsa05163 | Human cytomegalovirus infection | 20/93 | 225/8081 | 5.95E-13 | 7.77E-12 | 2.28E-12 | AKT1/CASP3/CCL2/CDKN1A/CTNNB1/CXCL8/EGFR/IKBKB/IL1B/IL6/MAPK1/MAPK3/MYC/NFKBIA/PTGER3/PTGS2/STAT3/TNF/TP53/VEGFA | | 20 |
| hsa05205 | Proteoglycans in cancer | 19/93 | 205/8081 | 1.15E-12 | 1.42E-11 | 4.18E-12 | AKT1/CASP3/CAV1/CDKN1A/CTNNB1/EGFR/ERBB2/ERBB3/ESR1/MAPK1/MAPK3/MMP2/MMP9/MYC/STAT3/TGFB1/TNF/TP53/VEGFA | | 19 |
| hsa05160 | Hepatitis C | 17/93 | 157/8081 | 1.62E-12 | 1.90E-11 | 5.58E-12 | AKT1/CASP3/CDKN1A/CTNNB1/EGF/EGFR/IFNG/IKBKB/MAPK1/MAPK3/MYC/NFKBIA/PPARA/RXRA/STAT3/TNF/TP53 | | 17 |
| hsa05323 | Rheumatoid arthritis | 14/93 | 93/8081 | 1.85E-12 | 2.05E-11 | 6.04E-12 | CCL2/CXCL8/FOS/ICAM1/IFNG/IL1A/IL1B/IL6/JUN/MMP1/MMP3/TGFB1/TNF/VEGFA | | 14 |
| hsa05133 | Pertussis | 13/93 | 76/8081 | 2.22E-12 | 2.34E-11 | 6.89E-12 | CASP3/CXCL8/FOS/IL10/IL1A/IL1B/IL6/JUN/MAPK1/MAPK3/MAPK8/NOS2/TNF | | 13 |
| hsa01521 | EGFR tyrosine kinase inhibitor resistance | 13/93 | 79/8081 | 3.73E-12 | 3.75E-11 | 1.10E-11 | AKT1/BCL2/BCL2L1/EGF/EGFR/ERBB2/ERBB3/IL6/MAPK1/MAPK3/PTEN/STAT3/VEGFA | | 13 |
| hsa01522 | Endocrine resistance | 14/93 | 98/8081 | 3.89E-12 | 3.75E-11 | 1.10E-11 | AKT1/BCL2/CDKN1A/EGFR/ERBB2/ESR1/FOS/JUN/MAPK1/MAPK3/MAPK8/MMP2/MMP9/TP53 | | 14 |
| hsa04660 | T cell receptor signaling pathway | 14/93 | 104/8081 | 8.96E-12 | 8.29E-11 | 2.44E-11 | AKT1/CD40LG/FOS/IFNG/IKBKB/IL10/IL2/IL4/JUN/MAPK1/MAPK3/MAPK8/NFKBIA/TNF | | 14 |
| hsa04932 | Non-alcoholic fatty liver disease | 16/93 | 150/8081 | 9.93E-12 | 8.82E-11 | 2.59E-11 | ADIPOQ/AKT1/CASP3/CXCL8/FOS/IKBKB/IL1A/IL1B/IL6/JUN/MAPK8/PPARA/RXRA/SREBF1/TGFB1/TNF | | 16 |
| hsa04926 | Relaxin signaling pathway | 15/93 | 129/8081 | 1.33E-11 | 1.14E-10 | 3.35E-11 | AKT1/EGFR/FOS/JUN/MAPK1/MAPK3/MAPK8/MMP1/MMP2/MMP9/NFKBIA/NOS2/NOS3/TGFB1/VEGFA | | 15 |
| hsa01524 | Platinum drug resistance | Dec-93 | 73/8081 | 2.70E-11 | 2.22E-10 | 6.51E-11 | AKT1/BCL2/BCL2L1/BIRC5/CASP3/CDKN1A/ERBB2/GSTM1/GSTP1/MAPK1/MAPK3/TP53 | | 12 |
| hsa05222 | Small cell lung cancer | 13/93 | 92/8081 | 2.79E-11 | 2.22E-10 | 6.51E-11 | AKT1/BCL2/BCL2L1/CASP3/CDKN1A/IKBKB/MYC/NFKBIA/NOS2/PTEN/PTGS2/RXRA/TP53 | | 13 |
| hsa05135 | Yersinia infection | 15/93 | 137/8081 | 3.21E-11 | 2.46E-10 | 7.23E-11 | AKT1/CCL2/CXCL8/FOS/IKBKB/IL10/IL1B/IL2/IL6/JUN/MAPK1/MAPK3/MAPK8/NFKBIA/TNF | | 15 |
| hsa05166 | Human T-cell leukemia virus 1 infection | 18/93 | 219/8081 | 3.74E-11 | 2.68E-10 | 7.88E-11 | AKT1/BCL2L1/CDKN1A/FOS/ICAM1/IKBKB/IL2/IL6/JUN/MAPK1/MAPK3/MAPK8/MYC/NFKBIA/PTEN/TGFB1/TNF/TP53 | | 18 |
| hsa05213 | Endometrial cancer | Nov-93 | 58/8081 | 3.74E-11 | 2.68E-10 | 7.88E-11 | AKT1/CDKN1A/CTNNB1/EGF/EGFR/ERBB2/MAPK1/MAPK3/MYC/PTEN/TP53 | | 11 |
| hsa05162 | Measles | 15/93 | 139/8081 | 3.97E-11 | 2.75E-10 | 8.09E-11 | AKT1/BCL2/BCL2L1/CASP3/FOS/IKBKB/IL1A/IL1B/IL2/IL6/JUN/MAPK8/NFKBIA/STAT3/TP53 | | 15 |
| hsa04151 | PI3K-Akt signaling pathway | 22/93 | 354/8081 | 4.77E-11 | 3.21E-10 | 9.44E-11 | AKT1/BCL2/BCL2L1/CDKN1A/EGF/EGFR/ERBB2/ERBB3/IKBKB/IL2/IL4/IL6/MAPK1/MAPK3/MYC/NOS3/PIK3CG/PTEN/RXRA/SPP1/TP53/VEGFA | | 22 |
| hsa04010 | MAPK signaling pathway | 20/93 | 294/8081 | 8.37E-11 | 5.47E-10 | 1.61E-10 | AKT1/CASP3/EGF/EGFR/ERBB2/ERBB3/FOS/HSPB1/IKBKB/IL1A/IL1B/JUN/MAPK1/MAPK3/MAPK8/MYC/TGFB1/TNF/TP53/VEGFA | | 20 |
| hsa05224 | Breast cancer | 15/93 | 147/8081 | 8.92E-11 | 5.66E-10 | 1.66E-10 | AKT1/CDKN1A/CTNNB1/EGF/EGFR/ERBB2/ESR1/FOS/JUN/MAPK1/MAPK3/MYC/PGR/PTEN/TP53 | | 15 |
| hsa05171 | Coronavirus disease - COVID-19 | 18/93 | 232/8081 | 9.78E-11 | 6.03E-10 | 1.77E-10 | CCL2/CXCL8/EGFR/F2/FOS/IKBKB/IL1B/IL2/IL6/JUN/MAPK1/MAPK3/MAPK8/MMP1/MMP3/NFKBIA/STAT3/TNF | | 18 |
| hsa04620 | Toll-like receptor signaling pathway | 13/93 | 104/8081 | 1.37E-10 | 7.84E-10 | 2.31E-10 | AKT1/CXCL8/FOS/IKBKB/IL1B/IL6/JUN/MAPK1/MAPK3/MAPK8/NFKBIA/SPP1/TNF | | 13 |
| hsa04625 | C-type lectin receptor signaling pathway | 13/93 | 104/8081 | 1.37E-10 | 7.84E-10 | 2.31E-10 | AKT1/IKBKB/IL10/IL1B/IL2/IL6/JUN/MAPK1/MAPK3/MAPK8/NFKBIA/PTGS2/TNF | | 13 |
| hsa05321 | Inflammatory bowel disease | Nov-93 | 65/8081 | 1.38E-10 | 7.84E-10 | 2.31E-10 | IFNG/IL10/IL1A/IL1B/IL2/IL4/IL6/JUN/STAT3/TGFB1/TNF | | 11 |
| hsa04380 | Osteoclast differentiation | 14/93 | 128/8081 | 1.57E-10 | 8.71E-10 | 2.56E-10 | AKT1/FOS/IFNG/IKBKB/IL1A/IL1B/JUN/MAPK1/MAPK3/MAPK8/NFKBIA/PPARG/TGFB1/TNF | | 14 |
| hsa04068 | FoxO signaling pathway | 14/93 | 131/8081 | 2.15E-10 | 1.16E-09 | 3.42E-10 | AKT1/CAT/CDKN1A/EGF/EGFR/IKBKB/IL10/IL6/MAPK1/MAPK3/MAPK8/PTEN/STAT3/TGFB1 | | 14 |
| hsa05235 | PD-L1 expression and PD-1 checkpoint pathway in cancer | Dec-93 | 89/8081 | 3.00E-10 | 1.59E-09 | 4.67E-10 | AKT1/EGF/EGFR/FOS/IFNG/IKBKB/JUN/MAPK1/MAPK3/NFKBIA/PTEN/STAT3 | | 12 |
| hsa04210 | Apoptosis | 14/93 | 136/8081 | 3.56E-10 | 1.84E-09 | 5.41E-10 | AKT1/BCL2/BCL2L1/BIRC5/CASP3/FOS/IKBKB/JUN/MAPK1/MAPK3/MAPK8/NFKBIA/TNF/TP53 | | 14 |
| hsa05132 | Salmonella infection | 17/93 | 249/8081 | 2.51E-09 | 1.27E-08 | 3.72E-09 | AKT1/BCL2/CASP3/CTNNB1/CXCL8/FOS/IKBKB/IL1B/IL6/JUN/MAPK1/MAPK3/MAPK8/MYC/NFKBIA/PIK3CG/TNF | | 17 |
| hsa04012 | ErbB signaling pathway | Nov-93 | 85/8081 | 2.72E-09 | 1.34E-08 | 3.95E-09 | AKT1/CDKN1A/EGF/EGFR/ERBB2/ERBB3/JUN/MAPK1/MAPK3/MAPK8/MYC | | 11 |
| hsa04630 | JAK-STAT signaling pathway | 14/93 | 162/8081 | 3.63E-09 | 1.75E-08 | 5.16E-09 | AKT1/BCL2/BCL2L1/CDKN1A/EGF/EGFR/IFNG/IL10/IL2/IL4/IL6/MYC/PIM1/STAT3 | | 14 |
| hsa05225 | Hepatocellular carcinoma | 14/93 | 168/8081 | 5.84E-09 | 2.76E-08 | 8.11E-09 | AKT1/BCL2L1/CDKN1A/CTNNB1/EGFR/GSTM1/GSTP1/HMOX1/MAPK1/MAPK3/MYC/PTEN/TGFB1/TP53 | | 14 |
| hsa05143 | African trypanosomiasis | Aug-93 | 37/8081 | 6.66E-09 | 3.02E-08 | 8.87E-09 | ICAM1/IFNG/IL10/IL1B/IL6/SELE/TNF/VCAM1 | | 8 |
| hsa05216 | Thyroid cancer | Aug-93 | 37/8081 | 6.66E-09 | 3.02E-08 | 8.87E-09 | CDKN1A/CTNNB1/MAPK1/MAPK3/MYC/PPARG/RXRA/TP53 | | 8 |
| hsa05164 | Influenza A | 14/93 | 171/8081 | 7.35E-09 | 3.23E-08 | 9.51E-09 | AKT1/CASP3/CCL2/CXCL8/ICAM1/IFNG/IKBKB/IL1A/IL1B/IL6/MAPK1/MAPK3/NFKBIA/TNF | | 14 |
| hsa05223 | Non-small cell lung cancer | Oct-93 | 72/8081 | 7.43E-09 | 3.23E-08 | 9.51E-09 | AKT1/CDKN1A/EGF/EGFR/ERBB2/MAPK1/MAPK3/RXRA/STAT3/TP53 | | 10 |
| hsa05206 | MicroRNAs in cancer | 18/93 | 310/8081 | 1.06E-08 | 4.52E-08 | 1.33E-08 | BCL2/CASP3/CDKN1A/EGFR/ERBB2/ERBB3/HMOX1/IKBKB/MAPK1/MAPK3/MMP9/MYC/PIM1/PTEN/PTGS2/STAT3/TP53/VEGFA | | 18 |
| hsa05226 | Gastric cancer | 13/93 | 149/8081 | 1.23E-08 | 5.15E-08 | 1.52E-08 | AKT1/BCL2/CDKN1A/CTNNB1/EGF/EGFR/ERBB2/MAPK1/MAPK3/MYC/RXRA/TGFB1/TP53 | | 13 |
| hsa05220 | Chronic myeloid leukemia | Oct-93 | 76/8081 | 1.27E-08 | 5.24E-08 | 1.54E-08 | AKT1/BCL2L1/CDKN1A/IKBKB/MAPK1/MAPK3/MYC/NFKBIA/TGFB1/TP53 | | 10 |
| hsa05152 | Tuberculosis | 14/93 | 180/8081 | 1.43E-08 | 5.76E-08 | 1.69E-08 | AKT1/BCL2/CASP3/IFNG/IL10/IL1A/IL1B/IL6/MAPK1/MAPK3/MAPK8/NOS2/TGFB1/TNF | | 14 |
| hsa04218 | Cellular senescence | 13/93 | 156/8081 | 2.15E-08 | 8.51E-08 | 2.50E-08 | AKT1/CDKN1A/CXCL8/IGFBP3/IL1A/IL6/MAPK1/MAPK3/MYC/PTEN/SERPINE1/TGFB1/TP53 | | 13 |
| hsa04064 | NF-kappa B signaling pathway | Nov-93 | 104/8081 | 2.38E-08 | 9.27E-08 | 2.73E-08 | BCL2/BCL2L1/CD40LG/CXCL8/ICAM1/IKBKB/IL1B/NFKBIA/PTGS2/TNF/VCAM1 | | 11 |
| hsa04931 | Insulin resistance | Nov-93 | 108/8081 | 3.55E-08 | 1.36E-07 | 3.99E-08 | AKT1/IKBKB/IL6/MAPK8/NFKBIA/NOS3/PPARA/PTEN/SREBF1/STAT3/TNF | | 11 |
| hsa04915 | Estrogen signaling pathway | Dec-93 | 138/8081 | 4.87E-08 | 1.83E-07 | 5.38E-08 | AKT1/BCL2/EGFR/ESR1/FOS/JUN/MAPK1/MAPK3/MMP2/MMP9/NOS3/PGR | | 12 |
| hsa04510 | Focal adhesion | 14/93 | 201/8081 | 5.81E-08 | 2.15E-07 | 6.32E-08 | AKT1/BCL2/CAV1/CTNNB1/EGF/EGFR/ERBB2/JUN/MAPK1/MAPK3/MAPK8/PTEN/SPP1/VEGFA | | 14 |
| hsa05221 | Acute myeloid leukemia | Sep-93 | 67/8081 | 5.91E-08 | 2.15E-07 | 6.32E-08 | AKT1/IKBKB/MAPK1/MAPK3/MPO/MYC/PIM1/PPARD/STAT3 | | 9 |
| hsa05169 | Epstein-Barr virus infection | 14/93 | 202/8081 | 6.19E-08 | 2.22E-07 | 6.51E-08 | AKT1/BCL2/CASP3/CDKN1A/ICAM1/IKBKB/IL6/JUN/MAPK8/MYC/NFKBIA/STAT3/TNF/TP53 | | 14 |
| hsa04920 | Adipocytokine signaling pathway | Sep-93 | 69/8081 | 7.69E-08 | 2.71E-07 | 7.96E-08 | ADIPOQ/AKT1/IKBKB/MAPK8/NFKBIA/PPARA/RXRA/STAT3/TNF | | 9 |
| hsa04658 | Th1 and Th2 cell differentiation | Oct-93 | 92/8081 | 8.28E-08 | 2.87E-07 | 8.44E-08 | FOS/IFNG/IKBKB/IL2/IL4/JUN/MAPK1/MAPK3/MAPK8/NFKBIA | | 10 |
| hsa04621 | NOD-like receptor signaling pathway | 13/93 | 181/8081 | 1.26E-07 | 4.31E-07 | 1.27E-07 | BCL2/BCL2L1/CCL2/CXCL8/IKBKB/IL1B/IL6/JUN/MAPK1/MAPK3/MAPK8/NFKBIA/TNF | | 13 |
| hsa05146 | Amoebiasis | Oct-93 | 102/8081 | 2.23E-07 | 7.49E-07 | 2.20E-07 | CASP3/CXCL8/HSPB1/IFNG/IL10/IL1B/IL6/NOS2/TGFB1/TNF | | 10 |
| hsa05202 | Transcriptional misregulation in cancer | 13/93 | 192/8081 | 2.52E-07 | 8.34E-07 | 2.45E-07 | BCL2L1/CDKN1A/CXCL8/IGFBP3/IL6/MMP3/MMP9/MPO/MYC/PLAT/PPARG/RXRA/TP53 | | 13 |
| hsa05130 | Pathogenic Escherichia coli infection | 13/93 | 197/8081 | 3.39E-07 | 1.11E-06 | 3.25E-07 | CASP3/CXCL8/F2/FOS/IKBKB/IL1B/IL6/JUN/MAPK1/MAPK3/MAPK8/NFKBIA/TNF | | 13 |
| hsa05131 | Shigellosis | 14/93 | 246/8081 | 7.05E-07 | 2.27E-06 | 6.67E-07 | AKT1/BCL2/BCL2L1/CXCL8/EGFR/IKBKB/IL1B/JUN/MAPK1/MAPK3/MAPK8/NFKBIA/TNF/TP53 | | 14 |
| hsa05230 | Central carbon metabolism in cancer | Aug-93 | 70/8081 | 1.19E-06 | 3.79E-06 | 1.11E-06 | AKT1/EGFR/ERBB2/MAPK1/MAPK3/MYC/PTEN/TP53 | | 8 |
| hsa05218 | Melanoma | Aug-93 | 72/8081 | 1.49E-06 | 4.65E-06 | 1.37E-06 | AKT1/CDKN1A/EGF/EGFR/MAPK1/MAPK3/PTEN/TP53 | | 8 |
| hsa04115 | p53 signaling pathway | Aug-93 | 73/8081 | 1.65E-06 | 5.10E-06 | 1.50E-06 | BCL2/BCL2L1/CASP3/CDKN1A/IGFBP3/PTEN/SERPINE1/TP53 | | 8 |
| hsa05214 | Glioma | Aug-93 | 75/8081 | 2.04E-06 | 6.19E-06 | 1.82E-06 | AKT1/CDKN1A/EGF/EGFR/MAPK1/MAPK3/PTEN/TP53 | | 8 |
| hsa05330 | Allograft rejection | Jun-93 | 38/8081 | 4.05E-06 | 1.22E-05 | 3.57E-06 | CD40LG/IFNG/IL10/IL2/IL4/TNF | | 6 |
| hsa04370 | VEGF signaling pathway | Jul-93 | 59/8081 | 4.46E-06 | 1.32E-05 | 3.88E-06 | AKT1/HSPB1/MAPK1/MAPK3/NOS3/PTGS2/VEGFA | | 7 |
| hsa05165 | Human papillomavirus infection | 15/93 | 331/8081 | 4.78E-06 | 1.39E-05 | 4.10E-06 | AKT1/CASP3/CDKN1A/CTNNB1/EGF/EGFR/IKBKB/MAPK1/MAPK3/PTEN/PTGS2/SPP1/TNF/TP53/VEGFA | | 15 |
| hsa05170 | Human immunodeficiency virus 1 infection | Dec-93 | 212/8081 | 5.00E-06 | 1.44E-05 | 4.24E-06 | AKT1/BCL2/BCL2L1/CASP3/FOS/IKBKB/JUN/MAPK1/MAPK3/MAPK8/NFKBIA/TNF | | 12 |
| hsa05332 | Graft-versus-host disease | Jun-93 | 42/8081 | 7.42E-06 | 2.11E-05 | 6.21E-06 | IFNG/IL1A/IL1B/IL2/IL6/TNF | | 6 |
| hsa04071 | Sphingolipid signaling pathway | Sep-93 | 119/8081 | 8.19E-06 | 2.27E-05 | 6.68E-06 | AKT1/BCL2/MAPK1/MAPK3/MAPK8/NOS3/PTEN/TNF/TP53 | | 9 |
| hsa04722 | Neurotrophin signaling pathway | Sep-93 | 119/8081 | 8.19E-06 | 2.27E-05 | 6.68E-06 | AKT1/BCL2/IKBKB/JUN/MAPK1/MAPK3/MAPK8/NFKBIA/TP53 | | 9 |
| hsa04664 | Fc epsilon RI signaling pathway | Jul-93 | 68/8081 | 1.17E-05 | 3.20E-05 | 9.39E-06 | AKT1/ALOX5/IL4/MAPK1/MAPK3/MAPK8/TNF | | 7 |
| hsa04930 | Type II diabetes mellitus | Jun-93 | 46/8081 | 1.28E-05 | 3.44E-05 | 1.01E-05 | ADIPOQ/IKBKB/MAPK1/MAPK3/MAPK8/TNF | | 6 |
| hsa05211 | Renal cell carcinoma | Jul-93 | 69/8081 | 1.29E-05 | 3.44E-05 | 1.01E-05 | AKT1/CDKN1A/JUN/MAPK1/MAPK3/TGFB1/VEGFA | | 7 |
| hsa04917 | Prolactin signaling pathway | Jul-93 | 70/8081 | 1.41E-05 | 3.70E-05 | 1.09E-05 | AKT1/ESR1/FOS/MAPK1/MAPK3/MAPK8/STAT3 | | 7 |
| hsa05120 | Epithelial cell signaling in Helicobacter pylori infection | Jul-93 | 70/8081 | 1.41E-05 | 3.70E-05 | 1.09E-05 | CASP3/CXCL8/EGFR/IKBKB/JUN/MAPK8/NFKBIA | | 7 |
| hsa05231 | Choline metabolism in cancer | Aug-93 | 98/8081 | 1.53E-05 | 3.95E-05 | 1.16E-05 | AKT1/EGF/EGFR/FOS/JUN/MAPK1/MAPK3/MAPK8 | | 8 |
| hsa04672 | Intestinal immune network for IgA production | Jun-93 | 49/8081 | 1.85E-05 | 4.73E-05 | 1.39E-05 | CD40LG/IL10/IL2/IL4/IL6/TGFB1 | | 6 |
| hsa04371 | Apelin signaling pathway | Sep-93 | 137/8081 | 2.56E-05 | 6.46E-05 | 1.90E-05 | AKT1/MAPK1/MAPK3/NOS2/NOS3/PIK3CG/PLAT/SERPINE1/SPP1 | | 9 |
| hsa04215 | Apoptosis - multiple species | May-93 | 32/8081 | 2.85E-05 | 7.12E-05 | 2.09E-05 | BCL2/BCL2L1/BIRC5/CASP3/MAPK8 | | 5 |
| hsa04662 | B cell receptor signaling pathway | Jul-93 | 82/8081 | 4.01E-05 | 9.90E-05 | 2.91E-05 | AKT1/FOS/IKBKB/JUN/MAPK1/MAPK3/NFKBIA | | 7 |
| hsa05134 | Legionellosis | Jun-93 | 57/8081 | 4.47E-05 | 0.00011 | 3.20E-05 | CASP3/CXCL8/IL1B/IL6/NFKBIA/TNF | | 6 |
| hsa04610 | Complement and coagulation cascades | Jul-93 | 85/8081 | 5.06E-05 | 0.00012 | 3.59E-05 | F10/F2/F3/F7/PLAT/SERPINE1/THBD | | 7 |
| hsa04921 | Oxytocin signaling pathway | Sep-93 | 154/8081 | 6.44E-05 | 0.00015 | 4.52E-05 | CDKN1A/EGFR/FOS/JUN/MAPK1/MAPK3/NOS3/PIK3CG/PTGS2 | | 9 |
| hsa04919 | Thyroid hormone signaling pathway | Aug-93 | 121/8081 | 7.05E-05 | 0.00017 | 4.90E-05 | AKT1/CTNNB1/ESR1/MAPK1/MAPK3/MYC/RXRA/TP53 | | 8 |
| hsa04940 | Type I diabetes mellitus | May-93 | 43/8081 | 0.00012 | 0.00029 | 8.48E-05 | IFNG/IL1A/IL1B/IL2/TNF | | 5 |
| hsa04060 | Cytokine-cytokine receptor interaction | Dec-93 | 295/8081 | 0.00013 | 0.00031 | 8.97E-05 | CCL2/CD40LG/CXCL8/IFNG/IL10/IL1A/IL1B/IL2/IL4/IL6/TGFB1/TNF | | 12 |
| hsa04928 | Parathyroid hormone synthesis, secretion and action | Jul-93 | 106/8081 | 0.00021 | 0.00047 | 0.00014 | BCL2/CDKN1A/EGFR/FOS/MAPK1/MAPK3/RXRA | | 7 |
| hsa03320 | PPAR signaling pathway | Jun-93 | 76/8081 | 0.00023 | 0.00051 | 0.00015 | ADIPOQ/MMP1/PPARA/PPARD/PPARG/RXRA | | 6 |
| hsa04913 | Ovarian steroidogenesis | May-93 | 51/8081 | 0.00028 | 0.00063 | 0.00018 | ALOX5/CYP19A1/CYP1A1/HSD3B1/PTGS2 | | 5 |
| hsa05010 | Alzheimer disease | 13/93 | 369/8081 | 0.00028 | 0.00063 | 0.00019 | AKT1/CASP3/CTNNB1/IKBKB/IL1A/IL1B/IL6/MAPK1/MAPK3/MAPK8/NOS2/PTGS2/TNF | | 13 |
| hsa04072 | Phospholipase D signaling pathway | Aug-93 | 148/8081 | 0.00029 | 0.00063 | 0.00019 | AKT1/CXCL8/EGF/EGFR/F2/MAPK1/MAPK3/PIK3CG | | 8 |
| hsa04725 | Cholinergic synapse | Jul-93 | 113/8081 | 0.00031 | 0.00066 | 0.0002 | ACHE/AKT1/BCL2/FOS/MAPK1/MAPK3/PIK3CG | | 7 |
| hsa05022 | Pathways of neurodegeneration - multiple diseases | 15/93 | 475/8081 | 0.00031 | 0.00067 | 0.0002 | BCL2/BCL2L1/CASP3/CAT/CTNNB1/IL1A/IL1B/IL6/MAPK1/MAPK3/MAPK8/NOS2/PTGS2/SOD1/TNF | | 15 |
| hsa04062 | Chemokine signaling pathway | Sep-93 | 192/8081 | 0.00034 | 0.00073 | 0.00022 | AKT1/CCL2/CXCL8/IKBKB/MAPK1/MAPK3/NFKBIA/PIK3CG/STAT3 | | 9 |
| hsa05204 | Chemical carcinogenesis | Jun-93 | 83/8081 | 0.00037 | 0.00077 | 0.00023 | CYP1A1/CYP1A2/CYP3A4/GSTM1/GSTP1/PTGS2 | | 6 |
| hsa01523 | Antifolate resistance | Apr-93 | 31/8081 | 0.00041 | 0.00085 | 0.00025 | IKBKB/IL1B/IL6/TNF | | 4 |
| hsa05310 | Asthma | Apr-93 | 31/8081 | 0.00041 | 0.00085 | 0.00025 | CD40LG/IL10/IL4/TNF | | 4 |
| hsa04935 | Growth hormone synthesis, secretion and action | Jul-93 | 119/8081 | 0.00042 | 0.00086 | 0.00025 | AKT1/FOS/IGFBP3/MAPK1/MAPK3/MAPK8/STAT3 | | 7 |
| hsa00140 | Steroid hormone biosynthesis | May-93 | 61/8081 | 0.00065 | 0.00132 | 0.00039 | CYP19A1/CYP1A1/CYP1A2/CYP3A4/HSD3B1 | | 5 |
| hsa04912 | GnRH signaling pathway | Jun-93 | 93/8081 | 0.00067 | 0.00136 | 0.0004 | EGFR/JUN/MAPK1/MAPK3/MAPK8/MMP2 | | 6 |
| hsa04350 | TGF-beta signaling pathway | Jun-93 | 94/8081 | 0.00071 | 0.00143 | 0.00042 | IFNG/MAPK1/MAPK3/MYC/TGFB1/TNF | | 6 |
| hsa04024 | cAMP signaling pathway | Sep-93 | 216/8081 | 0.00081 | 0.0016 | 0.00047 | AKT1/FOS/JUN/MAPK1/MAPK3/MAPK8/NFKBIA/PPARA/PTGER3 | | 9 |
| hsa04140 | Autophagy - animal | Jul-93 | 137/8081 | 0.00097 | 0.00191 | 0.00056 | AKT1/BCL2/BCL2L1/MAPK1/MAPK3/MAPK8/PTEN | | 7 |
| hsa04061 | Viral protein interaction with cytokine and cytokine receptor | Jun-93 | 100/8081 | 0.00099 | 0.00192 | 0.00057 | CCL2/CXCL8/IL10/IL2/IL6/TNF | | 6 |
| hsa05020 | Prion disease | Oct-93 | 273/8081 | 0.00112 | 0.00216 | 0.00063 | CASP3/CAV1/IL1A/IL1B/IL6/MAPK1/MAPK3/MAPK8/SOD1/TNF | | 10 |
| hsa04622 | RIG-I-like receptor signaling pathway | May-93 | 70/8081 | 0.00122 | 0.00233 | 0.00068 | CXCL8/IKBKB/MAPK8/NFKBIA/TNF | | 5 |
| hsa04520 | Adherens junction | May-93 | 71/8081 | 0.0013 | 0.00246 | 0.00072 | CTNNB1/EGFR/ERBB2/MAPK1/MAPK3 | | 5 |
| hsa04014 | Ras signaling pathway | Sep-93 | 232/8081 | 0.00134 | 0.00252 | 0.00074 | AKT1/BCL2L1/EGF/EGFR/IKBKB/MAPK1/MAPK3/MAPK8/VEGFA | | 9 |
| hsa00980 | Metabolism of xenobiotics by cytochrome P450 | May-93 | 78/8081 | 0.00197 | 0.00368 | 0.00108 | CYP1A1/CYP1A2/CYP3A4/GSTM1/GSTP1 | | 5 |
| hsa04934 | Cushing syndrome | Jul-93 | 155/8081 | 0.00199 | 0.00368 | 0.00108 | AHR/CDKN1A/CTNNB1/EGFR/HSD3B1/MAPK1/MAPK3 | | 7 |
| hsa00983 | Drug metabolism - other enzymes | May-93 | 80/8081 | 0.00221 | 0.00405 | 0.00119 | CYP3A4/GSTM1/GSTP1/MPO/XDH | | 5 |
| hsa04217 | Necroptosis | Jul-93 | 159/8081 | 0.0023 | 0.00418 | 0.00123 | BCL2/IFNG/IL1A/IL1B/MAPK8/STAT3/TNF | | 7 |
| hsa05203 | Viral carcinogenesis | Aug-93 | 204/8081 | 0.00233 | 0.00421 | 0.00124 | CASP3/CDKN1A/JUN/MAPK1/MAPK3/NFKBIA/STAT3/TP53 | | 8 |
| hsa04611 | Platelet activation | Jun-93 | 124/8081 | 0.00297 | 0.00532 | 0.00156 | AKT1/F2/MAPK1/MAPK3/NOS3/PIK3CG | | 6 |
| hsa05320 | Autoimmune thyroid disease | Apr-93 | 53/8081 | 0.00313 | 0.00556 | 0.00163 | CD40LG/IL10/IL2/IL4 | | 4 |
| hsa04211 | Longevity regulating pathway | May-93 | 89/8081 | 0.00351 | 0.00619 | 0.00182 | ADIPOQ/AKT1/CAT/PPARG/TP53 | | 5 |
| hsa04650 | Natural killer cell mediated cytotoxicity | Jun-93 | 131/8081 | 0.0039 | 0.00682 | 0.002 | CASP3/ICAM1/IFNG/MAPK1/MAPK3/TNF | | 6 |
| hsa00480 | Glutathione metabolism | Apr-93 | 57/8081 | 0.00408 | 0.00707 | 0.00208 | GSR/GSTM1/GSTP1/ODC1 | | 4 |
| hsa04910 | Insulin signaling pathway | Jun-93 | 137/8081 | 0.00485 | 0.00835 | 0.00246 | AKT1/IKBKB/MAPK1/MAPK3/MAPK8/SREBF1 | | 6 |
| hsa05416 | Viral myocarditis | Apr-93 | 60/8081 | 0.0049 | 0.00837 | 0.00246 | CASP3/CAV1/CD40LG/ICAM1 | | 4 |
| hsa04640 | Hematopoietic cell lineage | May-93 | 99/8081 | 0.00554 | 0.00938 | 0.00276 | IL1A/IL1B/IL4/IL6/TNF | | 5 |
| hsa04914 | Progesterone-mediated oocyte maturation | May-93 | 100/8081 | 0.00578 | 0.00971 | 0.00286 | AKT1/MAPK1/MAPK3/MAPK8/PGR | | 5 |
| hsa04623 | Cytosolic DNA-sensing pathway | Apr-93 | 63/8081 | 0.00583 | 0.00974 | 0.00286 | IKBKB/IL1B/IL6/NFKBIA | | 4 |
| hsa04550 | Signaling pathways regulating pluripotency of stem cells | Jun-93 | 143/8081 | 0.00597 | 0.00989 | 0.00291 | AKT1/CTNNB1/MAPK1/MAPK3/MYC/STAT3 | | 6 |
| hsa04929 | GnRH secretion | Apr-93 | 64/8081 | 0.00617 | 0.01015 | 0.00298 | AKT1/MAPK1/MAPK3/SPP1 | | 4 |
| hsa04020 | Calcium signaling pathway | Aug-93 | 240/8081 | 0.00625 | 0.0102 | 0.003 | EGF/EGFR/ERBB2/ERBB3/NOS2/NOS3/PTGER3/VEGFA | | 8 |
| hsa04137 | Mitophagy - animal | Apr-93 | 68/8081 | 0.00764 | 0.01238 | 0.00364 | BCL2L1/JUN/MAPK8/TP53 | | 4 |
| hsa04960 | Aldosterone-regulated sodium reabsorption | Mar-93 | 37/8081 | 0.00864 | 0.0139 | 0.00409 | MAPK1/MAPK3/NR3C2 | | 3 |
| hsa04150 | mTOR signaling pathway | Jun-93 | 155/8081 | 0.00875 | 0.01398 | 0.00411 | AKT1/IKBKB/MAPK1/MAPK3/PTEN/TNF | | 6 |
| hsa00982 | Drug metabolism - cytochrome P450 | Apr-93 | 72/8081 | 0.00932 | 0.01478 | 0.00434 | CYP1A2/CYP3A4/GSTM1/GSTP1 | | 4 |
| hsa04670 | Leukocyte transendothelial migration | May-93 | 114/8081 | 0.00994 | 0.01565 | 0.0046 | CTNNB1/ICAM1/MMP2/MMP9/VCAM1 | | 5 |
| hsa04310 | Wnt signaling pathway | Jun-93 | 160/8081 | 0.01015 | 0.01586 | 0.00466 | CTNNB1/JUN/MAPK8/MYC/PPARD/TP53 | | 6 |
| hsa04726 | Serotonergic synapse | May-93 | 115/8081 | 0.0103 | 0.01598 | 0.0047 | ALOX5/CASP3/MAPK1/MAPK3/PTGS2 | | 5 |
| hsa04015 | Rap1 signaling pathway | Jul-93 | 210/8081 | 0.01039 | 0.01602 | 0.00471 | AKT1/CTNNB1/EGF/EGFR/MAPK1/MAPK3/VEGFA | | 7 |
| hsa05168 | Herpes simplex virus 1 infection | Dec-93 | 498/8081 | 0.0114 | 0.01745 | 0.00513 | AKT1/BCL2/BCL2L1/CASP3/CCL2/IFNG/IKBKB/IL1B/IL6/NFKBIA/TNF/TP53 | | 12 |
| hsa00380 | Tryptophan metabolism | Mar-93 | 42/8081 | 0.01225 | 0.01863 | 0.00548 | CAT/CYP1A1/CYP1A2 | | 3 |
| hsa04146 | Peroxisome | Apr-93 | 83/8081 | 0.01513 | 0.02286 | 0.00672 | CAT/NOS2/SOD1/XDH | | 4 |
| hsa04540 | Gap junction | Apr-93 | 88/8081 | 0.01839 | 0.02759 | 0.00811 | EGF/EGFR/MAPK1/MAPK3 | | 4 |
| hsa00330 | Arginine and proline metabolism | Mar-93 | 51/8081 | 0.02064 | 0.03075 | 0.00904 | NOS2/NOS3/ODC1 | | 3 |
| hsa00220 | Arginine biosynthesis | Feb-93 | 22/8081 | 0.02607 | 0.03858 | 0.01134 | NOS2/NOS3 | | 2 |
| hsa04923 | Regulation of lipolysis in adipocytes | Mar-93 | 57/8081 | 0.02761 | 0.0406 | 0.01193 | AKT1/PTGER3/PTGS2 | | 3 |
| hsa04916 | Melanogenesis | Apr-93 | 101/8081 | 0.0288 | 0.04206 | 0.01236 | CTNNB1/MAPK1/MAPK3/TYR | | 4 |
| hsa04261 | Adrenergic signaling in cardiomyocytes | May-93 | 150/8081 | 0.02907 | 0.04219 | 0.0124 | AKT1/BCL2/MAPK1/MAPK3/PIK3CG | | 5 |
| hsa04213 | Longevity regulating pathway - multiple species | Mar-93 | 62/8081 | 0.03426 | 0.04932 | 0.0145 | AKT1/CAT/SOD1 | | 3 |
| hsa04390 | Hippo signaling pathway | May-93 | 157/8081 | 0.03443 | 0.04932 | 0.0145 | BIRC5/CTNNB1/MYC/SERPINE1/TGFB1 | | 5 |
